# Asymmetric synthesis of *N*-bridged [3.3.1] ring systems by phosphonium salt/Lewis acid relay catalysis

**DOI:** 10.1038/s41467-022-28001-8

**Published:** 2022-01-18

**Authors:** Jian-Ping Tan, Kehan Li, Boming Shen, Cheng Zhuang, Zanjiao Liu, Kai Xiao, Peiyuan Yu, Bing Yi, Xiaoyu Ren, Tianli Wang

**Affiliations:** 1 Key Laboratory of Green Chemistry & Technology of Ministry of Education, College of Chemistry, Sichuan University, Chengdu, P. R. China; 2 Hunan Province Key Laboratory of Environmental Catalysis and Waste Recycling, College of Materials and Chemical Engineering, Hunan Institute of Engineering, Xiangtan, P. R. China; 3 Department of Chemistry and Shenzhen Grubbs Institute, Southern University of Science and Technology, Shenzhen, 518055 P. R. China; 4 Precision Medicine Research Center & Sichuan Provincial Key Laboratory of Precision Medicine, West China Hospital, Sichuan University, Chengdu, 610041 P. R. China; 5 Beijing National Laboratory for Molecular Sciences, Beijing, 100190 China

**Keywords:** Synthetic chemistry methodology, Organocatalysis

## Abstract

Optically pure pseudo-natural products (PNPs), particularly exemplified by azabicyclo[3.3.1]nonane molecules and their analogs provide an attractive platform for structure−activity relationship studies, and also lead new compound discovery in drug development. However, there are currently no examples of guiding catalytic asymmetric strategies available to construct such important PN-scaffolds, thus limiting their broad use. Here, we report a general and modular method for constructing these pseudo-natural *N*-bridged [3.3.1] ring systems via cascade process by bifunctional phosphonium salt/Lewis acid relay catalysis. A wide variety of substrates bearing an assortment of functional groups (59 examples) are compatible with this protocol. Other features include a [3 + 2] cyclization/ring-opening/Friedel-Crafts cascade pathway, excellent reactivities and stereoselectivities, easily available starting materials, step economy and scalability. The obtained enantioenriched products showed potential of preliminary anticancer activities. Insights gained from our studies are expected to advance general efforts towards the catalytic synthesis of challenging even unprecedented chiral PNPs, offering new opportunities for bioactive small-molecule discovery.

## Abstract (web summary)

Optically pure pseudo-natural products (PNPs) bearing N-bridged [3.3.1] ring systerm provide a platform for structure−activity relationship studies, but currently no examples of catalytic asymmetric strategies are available to construct such scaffolds. Here the authors show a general method for constructing such N-bridged [3.3.1] ring systems via a bifunctional phosphonium salt/Lewis acid relay catalysis.

## Introduction

**Fig. 1:**
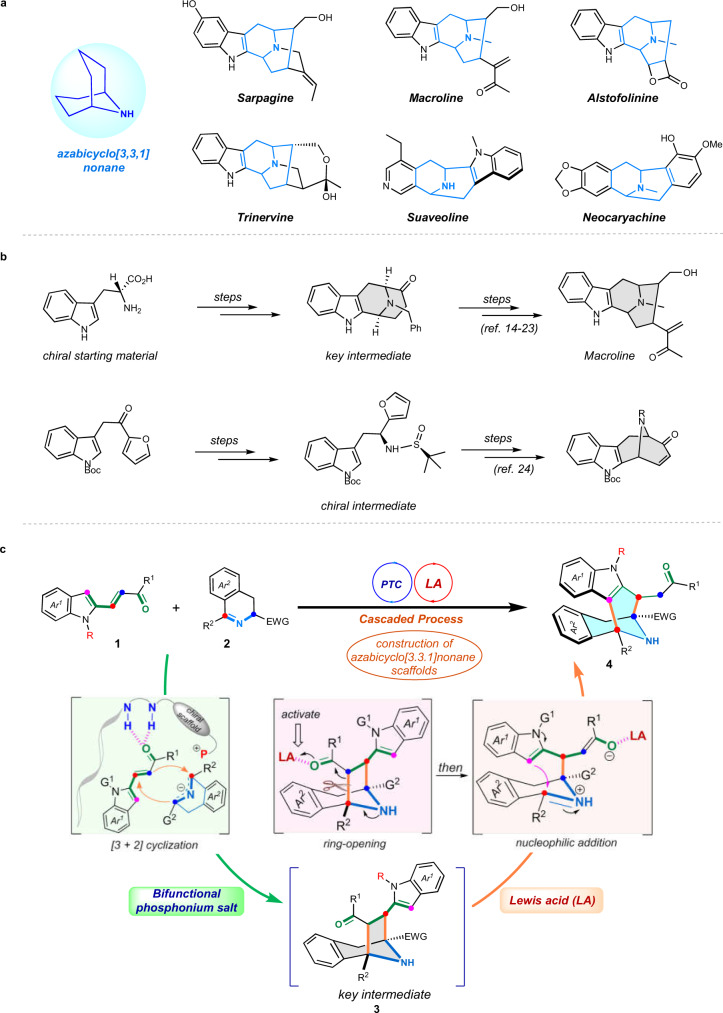
Chiral aryl-fused azabicyclo[3.3.1]nonane compounds and their synthesis. **a** Representative bioactive molecules containing aryl-fused azabicyclo[3.3.1]nonane framework. **b** Reported total synthesis strategies for preparing chiral azabicyclo[3.3.1]nonane scaffolds. **c** The first catalytic asymmetric approach to construct azabicyclo[3.3.1]nonane molecules by metal-free relay catalysis (this work).

Chiral azabicyclo[3.3.1]nonane-containing molecules, especially these aryl-fused *N*-bridged [3.3.1] ring systems that have highly functionalized and complex architectures represent an important and unique family of (pseudo-)natural products, which have arguably been subject to extensive structural, pharmacological, biosynthetic, and synthetic investigations owing to their multitudinous bioactive and medicinal properties^1–3^. Most of their family members as exemplified by Sarpagine, Macroline, Suaveoline, Trinervine, etc. (Fig. 1a), which belong to the group of monoterpenoid indole alkaloids^4–7^, are mainly isolated from infrequent natural plants and particularly have antiproliferative activity and antitumor effect, thus playing key roles in the treatment of Alzheimer’s disease (AD), Parkinson’s disease, etc.^8–10^. Consequently, as an important subject of biology-oriented synthesis (BIOS)^11,12^, these *N*-bridged [3.3.1] ring systems are arguably one of the most versatile classes of target scaffolds towards developing and discovering small-molecule drugs and/or their leading compounds, which have been fascinated and pursued by both synthetic and pharmaceutical chemists for decades^7,13,14^. Synthetic access to these (pseudo-)natural compounds was pioneered by Cook and co-workers, who have accomplished very elegant total synthesis of several indole alkaloids with this structural feature by using Dieckmann condensation to construct the *N*-bridged framework^13–16^. Besides, efficient strategies also include biomimetic synthesis and olefin metathesis by the Martin group^17,18^, a [5 + 2] cycloaddition/ring enlargement by the Gaich group^19,20^, and other methods^21–23^. However, despite these advances, most of the existing methods require tediously long steps including functional-group pre-installations and transformations, harsh reaction conditions, and complicated operations. Moreover, only sporadic examples that disclosed the synthesis of chiral azabicyclo[3.3.1]nonane compounds have been available so far (Fig. 1b), which utterly relied on utilizing chiral auxiliary^24^ or employing chiral starting materials^14–23^, respectively. To the best of our knowledge, the catalytic asymmetric protocol for constructing such ring systems has never been reported to date, which remains a tremendous challenge and long-standing assignment in modern synthetic chemistry. Of note, these eight-membered *N*-bridged ring systems are particularly difficult to prepare due to their high strain energies, lacking of effective catalytic systems to overcome the negative enthalpic and entropic challenges of such organic reactions.

From the perspective of efficiency and practicability of chemical synthesis, one essential objective is to convert readily available and inexpensive starting materials into structurally complex and multi-functional molecules with a perfect stereocontrol^25^. In this context, asymmetric organocatalysis that greatly enhances the synthetic toolbox by complementing metal-based and enzymatic methodologies offers powerful solutions to this endeavor^26^, which is conducive to expanding the diversity of chiral molecules for modulating biological targets, thus providing more opportunities for drug discovery and development^27^. Among the well-known asymmetric organocatalytic systems, asymmetric phase-transfer catalysis (PTC)^28^ particularly involving phosphonium salts as catalysts provide a powerful and versatile tool for the enantioselective synthesis of diverse chiral molecules^29–32^. The groups of Maruoka, Ooi, and Zhao have pioneered to make significant contributions in this field^33–37^. Recently, our group has developed a series of multi-/bifunctional phosphonium salt catalysts and demonstrated their applications in a variety of asymmetric synthetic reactions^38–41^. In connection to the above synthetic challenge and our disclosure of phosphonium salts and their successful applications in asymmetric synthesis, we envisioned that the employment of highly tunable bifunctional phosphonium salts together with metal-free Lewis acid catalysts may result in a practical cascade transformation towards constructing these synthetically challenging azabicyclo[3.3.1]nonane scaffolds.

Herein, we describe an asymmetric synthesis of pseudo-natural *N*-bridged [3.3.1] ring systems via a novel cascade approach by chiral phosphonium salt/Lewis acid relay catalysis. Starting from commonly available indole-based α,β-unsaturated ketones, and cyclic azomethine ylides, this cascade reaction proceeded along a [3 + 2] cyclization/ring-opening/Friedel-Crafts pathway. The stereocontrol in the phosphonium salt-promoted cyclization step is realized via the synergistic effects of ion-pair and hydrogen-bonding between both reactants and cationic catalysts, respectively. DFT investigations provided insights into the Lewis acid-promoted ring-opening/ring-expansion process. Moreover, preliminary biological activity investigations indicated that these azabicyclo[3.3.1]nonane-based molecules are potential antitumor agents (Fig. 1c).

## Results

### Reaction development

Our general approach to address the above issue is depicted in Fig. 1c. We initially designed and synthesized indole-based α,β-unsaturated ketones **1**, and cyclic azomethine ylides **2** as starting materials. We hypothesized that an asymmetric 1,3-dipolar cyclization between these two types of reactants would proceed by phosphonium salt catalysis, generating chiral indole-fused tropane intermediates **3**^41^. Next, metal-free Lewis acid catalysts such as BF_3_ would promote a ring-opening process of this key intermediate via coordination of the Lewis acid to the carbonyl group. Subsequently, an intramolecular Friedel–Crafts-type reaction could occur, in which the electron-rich indole moiety serves as the nucleophile to attack the iminium unit, thus furnishing the desired *N*-bridged [3.3.1] ring systems **4**. While 2-alkenylindole compounds have been well utilized in organic synthesis^42^, the employment of 2-indolylethylene ketones in the asymmetric cascade process begins with a dipolar cyclization, in which these substrates initially serve as C2-sythons, is virtually unexplored^43^. Of note, the crucial asymmetric induction in this cascade should come from the first cyclization step, and thus would be expected to originate from the ion-pairing and H-bonding capability of the chiral phosphonium catalyst. Whereas the idea of constructing optically pure pseudo-natural *N*-bridged [3.3.1] rings by metal-free relay catalysis that involves a chiral phosphonium salt catalyst cooperated with a Lewis acid was very appealing to us, we were mindful that the catalytic asymmetric cascade between 2-alkenylindoles and cyclic azomethine ylides for producing these rigid azabicyclo[3.3.1]nonane scaffolds is extremely challenging, and moreover the catalytic asymmetric protocol towards synthesizing such pseudo-natural products has never been accomplished so far.

**Fig. 2:**
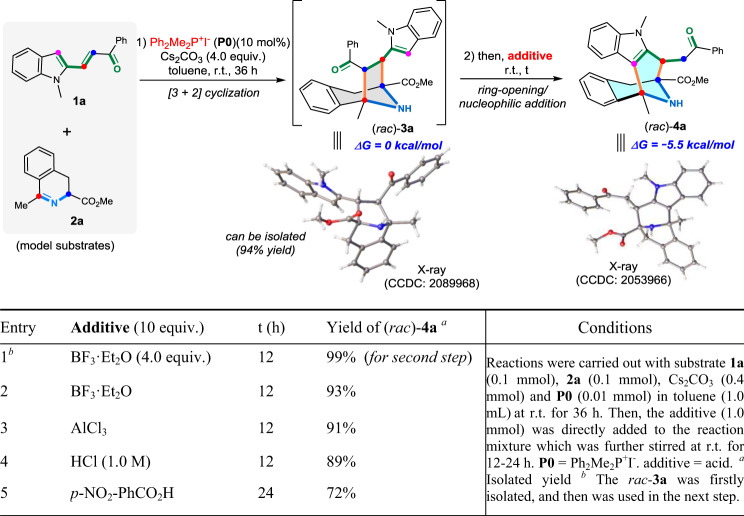
Initial investigations on the feasibility of this cascade process. The annulation reaction between N-methylated 2-alkenylindole **1a** and benzyl-fused cyclic azomethine ylide **2a** in the presence of racemic dimethyldiphenylphosphonium iodide catalyst **P0** under standard PTC conditions was tested, and then different types of acids towards promoting the further ring-opening/ring-expansion process was envaluated.

With this consideration in mind, we initially evaluated the feasibility of this hypothesis by choosing the cascade reaction between *N*-methylated 2-alkenylindole **1a** and benzyl-fused cyclic azomethine ylide **2a** in the presence of racemic dimethyldiphenylphosphonium iodide catalyst **P0** under standard PTC conditions as the model reaction. As expected, the dipolar [3 + 2] cyclization product *rac*-**3a** that was identified by X-ray crystallographic analysis (CCDC 2089968) was readily generated and isolated in 94% yield (Fig. 2). It is noteworthy that this seven-membered *N*-bridged [3.2.1] ring product *rac*-**3a** could be completely converted into a new eight-membered *N*-bridged [3.3.1] ring product *rac*-**4a** (CCDC 2053966) in the presence of 4.0 equivalents of BF_3_·Et_2_O at r.t. for 12 h (entry 1). More importantly, the crude reaction mixture that mainly contained *N*-bridged [3.2.1] ring intermediate could be in situ converted and thus afforded eight-membered *N*-bridged [3.3.1] ring product *rac*-**4a** by directly adding either Lewis acids such as BF_3_·Et_2_O or AlCl_3_ (entries 2 and 3) or Brønsted acids such as hydrochloric acid or *p*-nitrobenzoic acid (entries 4 and 5). Also, DFT computational results show that the free energy difference between **3a** and **4a** is 5.5 kcal/mol, which indicates that the ring-opening/ring-expansion process is thermodynamically favored since the seven-membered intermediate **3a** is less stable than the final eight-membered product **4a**.

### Condition optimization

**Fig. 3:**
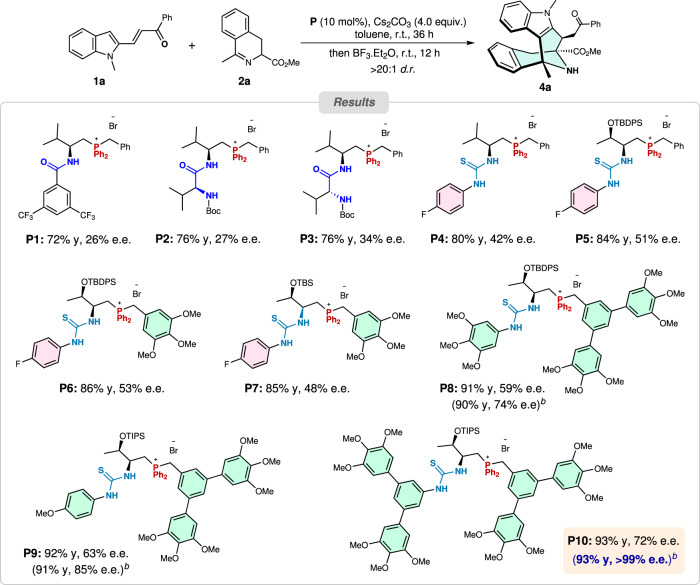
Selected optimization studies on bifunctional chiral phosphonium salts. ^*a*^Reaction condition: substrates **1a** (0.10 mmol) and **2a** (0.12 mmol) in the presence of **P** (0.01 mmol) and Cs_2_CO_3_ (0.40 mmol) in toluene (2.0 mL) at r.t. for 36 h; then BF_3_·Et_2_O (1.0 mmol) was directly added to the reaction mixture which was further stirred at r.t. for 12 h. ^*b*^Optimal conditions: the reaction was performed with 6.0 equiv. of Cs_2_CO_3_ in Et_2_O at −20 °C for 72 h; and then BF_3_.Et_2_O was directly added at r.t. for further stirring 12 h (see the SI for more details).

Encouraged by the initial results, we next explored the asymmetric version of this cascade reaction. For the development of effective catalysts, given our recent progress in developing phosphonium-involved bifunctional catalytic systems^38–41^, we chose amide-, dipeptide- and thiourea-derived phosphonium salts as candidate catalysts because of their representative hydrogen-bonding and ion-pairing features. Pleasingly, all tested phosphonium salt catalysts were effective in promoting this reaction, furnishing the desired *N*-bridged azabicyclo[3.3.1]nonane product in high yields and excellent diastereoselectivities (all > 20:1 *d.r*.) (Fig. 3). When phosphonium salts **P1** bearing an amide framework were employed, the reaction proceeded smoothly, affording the desired product in good yield and diastereoselectivity, albeit with low enantioselectivity. Delightedly, while dipeptide-based phosphonium salts **P2–3** that particularly bear double H-bonding-donating moieties were used, the ee value was slightly improved. At last, thiourea-based phosphonium salt **P4** was found to be more effective in promoting this reaction, affording the product with a slightly higher ee value. Further screening of other analogous catalysts **P5**−**10** identified that **P10** could catalyze this cascade process with excellent efficiency (93% yield) and moderate enantioselectivity (72% e.e.) at room temperature. Finally, optimization of other reaction parameters, such as solvent, base, temperature, chiral catalyst loading, and the cooperative Lewis acid catalysts, concluded that the best result (93% yield, > 20:1 *d.r*. and > 99% e.e.) could be obtained when the reaction was run at −20 °C with 10 mol% of catalyst **P10** and 6.0 equivalents of Cs_2_CO_3_ in ether for 72 h, with the subsequent addition of 10.0 equivalents of BF_3_·Et_2_O at r.t. for stirring another 12 h (see Supplementary Tables S2–S6 for more details).

### Substrates scope exploration

**Fig. 4:**
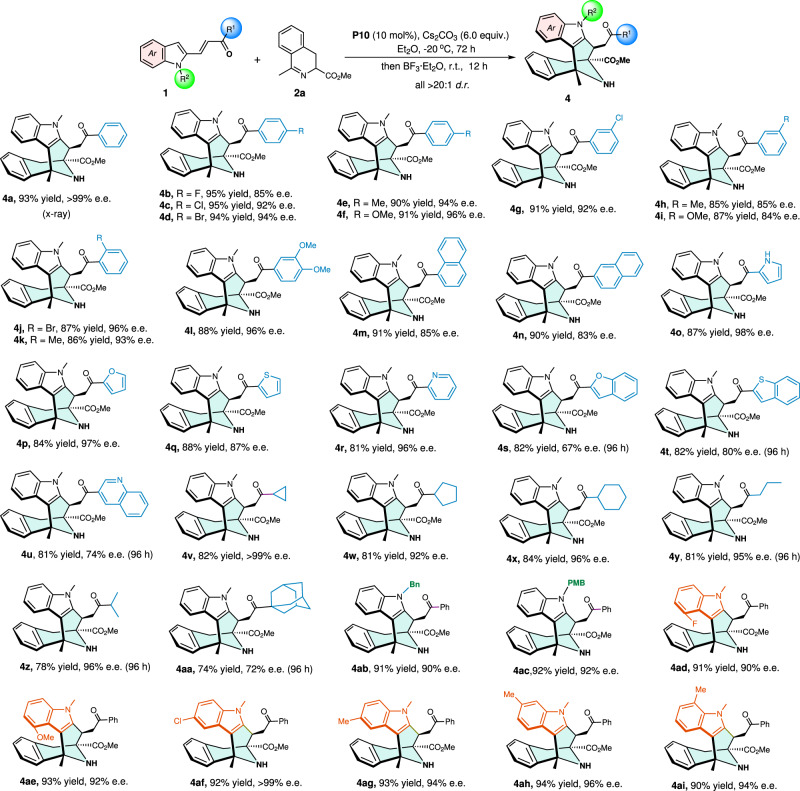
Scope with respect to 2-vinylindoles. Unless other noticed, reactions were performed with **1** (0.10 mmol), **2a** (0.12 mmol), **P10** (0.01 mmol), and Cs_2_CO_3_ (0.60 mmol) in Et_2_O (2.0 mL) at −20 °C for 72 h; then BF_3_·Et_2_O (1.0 mmol) was directly added to the reaction mixture which was further stirred at r.t. for 12 h. Isolated yield. The e.e. value was determined by HPLC analysis on a chiral stationary phase.

Encouraged by the above exciting results, we proceeded to establish the substrate scope for this novel cascade reaction. Firstly, the scope of 2-vinylindoles was assessed by using cyclic azomethine ylide **2a** as the reacting partner under the above optimal conditions. As shown in Fig. 4, various aryl-substituted 2-vinylindoles regardless of the positions and electronic properties of the substituents on the phenyl ring were perfectly compatible with the reaction conditions, providing the corresponding products (**4a**–**n**) in high yields (85−95%) with excellent diastereoselectivities (>20:1 *d.r*.) and good to excellent enantioselectivities (83−99% e.e.) Notably, 2-vinylindoles containing heteroaromatic rings such as pyrrole, furan, thiophene, pyridine, benzofuran, benzothiophene, and benzopyridine were also proven to be ideal substrates, affording the desired products (**4o**–**u**) in high yields with satisfying stereoselectivities. Importantly, alkyl-substituted 2-vinylindoles were also well-tolerated in this reaction, producing the corresponding products (**4v**–**aa**) with excellent yields and good stereoselectivities excepting for the 1-adamantane-substituted product **4aa** with a slightly lower ee value, which may be attributed to the steric hindrance of the adamantane group. Also, good yields and excellent stereoselectivities (**4ab** and **4ac**) were obtained from these reactions, in which the protective group on the nitrogen-atom of substrates was changed. In addition, the substituents of the indole rings did not affect the reactivities and stereoselectivities of the cascade reaction, affording the corresponding products **4ad**−**i** in high yields and excellent *dr* and ee values in all cases.

**Fig. 5:**
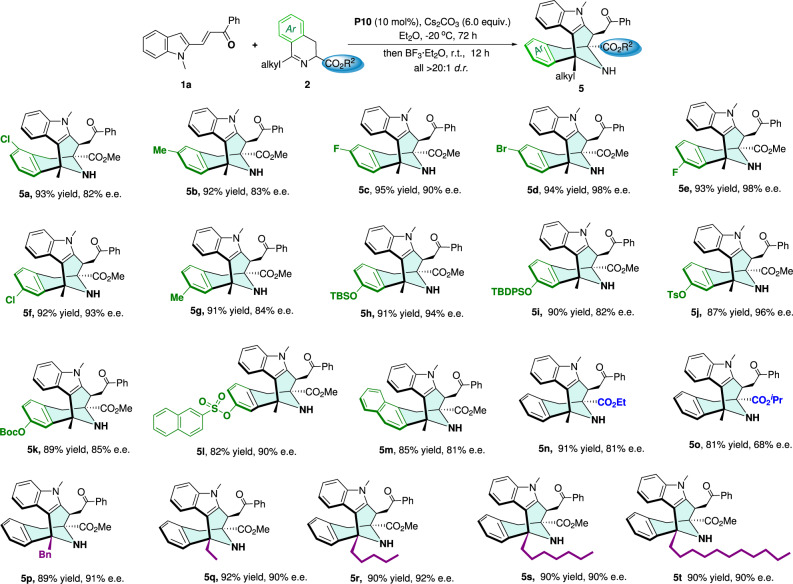
Scope with respect to cyclic azomethine ylides. Reactions were performed with **1a** (0.10 mmol), **2** (0.12 mmol), **P10** (0.01 mmol), and Cs_2_CO_3_ (0.60 mmol) in Et_2_O (2.0 mL) at −20 °C for 72 h; then BF_3_·Et_2_O (1.0 mmol) was directly added to the reaction mixture which was further stirred at r.t. for 12 h. Isolated yield. The e.e. value was determined by HPLC analysis on a chiral stationary phase.

Subsequently, we further evaluated the scope of the cyclic azomethine ylides **2** (Fig. 5). Delightfully, an array of cyclic azomethine ylides with electron-donating or electron-withdrawing substituents at the 6- or 7-position of the phenyl ring were found to be suitable reaction partners, furnishing the desired products **5a**–**l** with good yields (82–95%) and high ee values (82–98%). Alternatively, the azomethine ylide that was fused by the naphthyl group was also proven to be tolerated for this cascade system, providing the corresponding product (**5m**) in good stereoselectivities. Also, the cyclic azomethine ylides with different esters were suitable substrates for affording the desired products (**5n** and **5o**). It was worth mentioning that the cyclic azomethine ylides bearing different alkyl substituents could be compatible with the standard conditions, affording the products (**5p**–**t**) in high yields with excellent diastereo- and enantioselectivities.

### Synthetic applications

**Fig. 6:**
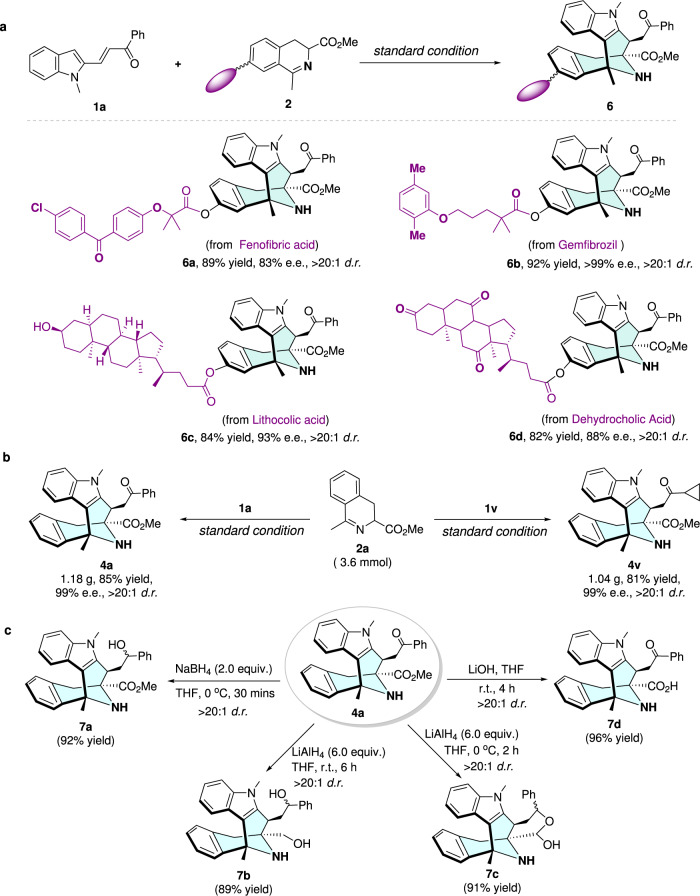
Synthetic applications. **a** Late-stage diversification. **b** Gram-scale preparations. **c** Product transformations.

In order to evaluate the practicality of this cascade protocol, the cyclic azomethine ylide species that have been installed on different drug molecule skeletons were employed as starting materials. All these supported cyclic azomethine ylides could also serve as effective substrates, constructing the corresponding cascade products **6a**–**d** in good yields, satisfying enantioselectivities, and excellent diastereoselectivities (Fig. 6a). Moreover, this synthetic protocol is equally robust for gram-scale synthesis with no erosion of both yields and stereoselectivities (Fig. 6b). In view of the importance of these azabicyclo[3,3,1]nonane scaffolds in natural products and pharmaceutical chemistry, the synthesis of diverse azabicyclo[3,3,1]nonane-containing molecules is of great interest. As illustrated in Fig. 6c, the eight-membered product **4a** could be easily transformed into different classes of highly functionalized bioactive compounds (**7a**–**d**) that bear intrinsically synthetic challenges.

### Biological activity study

Particularly, we were intrigued by the potential biological activities of these chiral pseudo-natural *N*-bridged [3.3.1] ring systems. Thus the cytotoxic effects of these randomly selected products against a panel of cancer cell lines including HCT116 human colon cancer cells, BB4 human lung cancer cells, and A375 human melanoma cells were screened by Cell Counting Kit-8 (CCK-8) assay. Our preliminary studies demonstrated that two of them exhibited noticeable cytotoxicity against cancer cells at the concentration of 20 μM (see Supplementary Table S13 for details). Next, the half-maximal inhibitory concentration (IC_50_) values of these two active compounds against the above cancer cell lines, together with two other cancer cell lines (HeLa human cervical carcinoma cells and NCIN87 human gastric carcinoma cells), were further examined. As illustrated in Supplementary Fig. S4 and Supplementary Table S14, the two representative products (**4o** and **4s**) demonstrated concentration-dependent cytotoxicity against all the tested cancer cell lines. Particularly, in some cancer cells such as HCT116, BB4, and HeLa, the cytotoxicity of these compounds was similar to that of classical chemotherapeutic drugs such as cisplatin and irinotecan. Of note, both of them were less cytotoxic towards normal cells (HUVEC, human umbilical vein endothelial cells). These preliminary results indicate that these pseudonatural *N*-bridged [3.3.1] ring systems show great potential for further development into anticancer agents.

### Mechanistic investigations

**Fig. 7:**
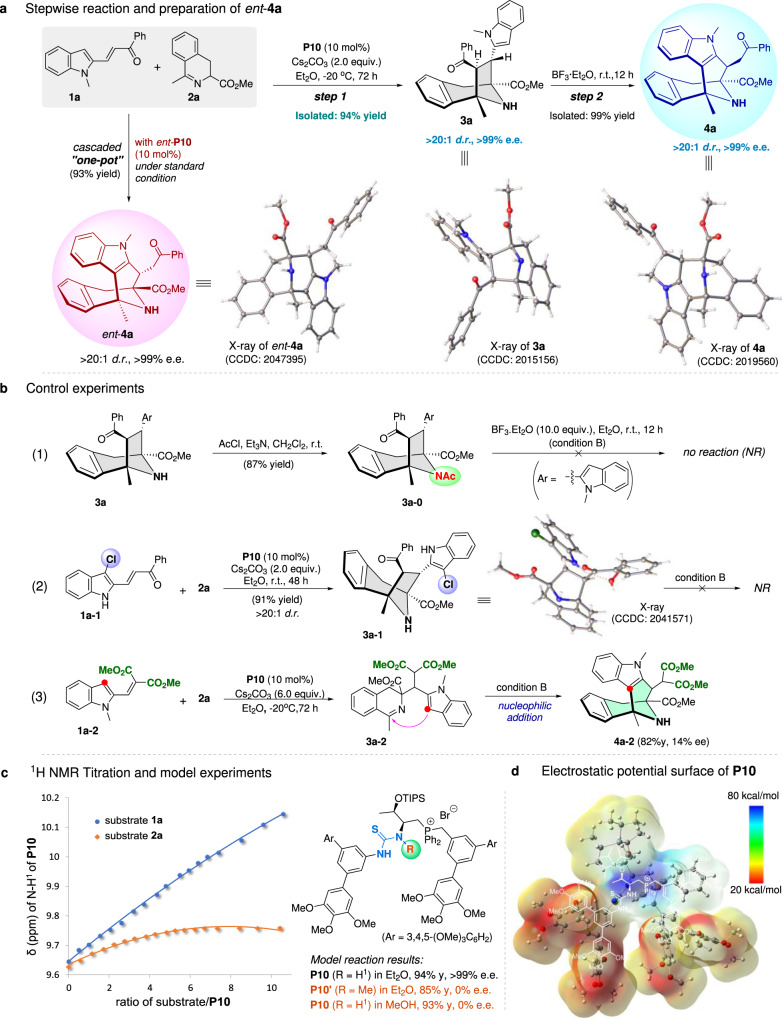
Mechanistic investigations. **a** Stepwise reaction and preparation of *ent*-**4a**. **b.** Control experiments. **c** ^1^H NMR titration and model experiments(sample size, *n* = 18). **d** Electrostatic potential surface (ESP) of catalyst **P10**.

Next, we carried out a series of experiments to understand the reaction mechanism. Firstly, the model cascade reaction between 2-indole-vinyl ketone **1a** and cyclic azomethine ylide **2a** was performed in the presence of catalyst **P10** under standard PTC conditions, and chiral key intermediate **3a** (CCDC 2015156) was obtained and fully characterized (94% yield, >99% e.e., and >20:1 d.r.). This seven-membered product **3a** could be quantitatively transformed into chiral eight-membered product **4a** (CCDC 2019560) via a Lewis acid BF_3_·Et_2_O promoted ring-opening/Friedel–Crafts cascade process without any loss of the stereoselectivities. While the phosphonium *ent*-**P10** was utilized as a catalyst, the model reaction proceeded efficiently to form the corresponding product *ent*-**4a** (CCDC 2047395) with excellent enantioselectivity under the standard “one-pot” conditions (Fig. 7a). These preliminary results clearly demonstrated that the desired [3.3.1] cyclic product **4a** was generated from the key [3.2.1] cyclic intermediate **3a** via cascade process, but not directly constructed via formal [3 + 3] cyclization between **1a** and 1, 3-dipole **2a**. Then, various control experiments were further carried out (Fig. 7b). In particular, while the NH-unit of **3a** was protected to form the *N*-acetyl [3.2.1] cyclic intermediate **3a–0**, this compound could not be converted into the ring-expanding [3.3.1] cyclic system under the above Lewis acid catalytic conditions, which was probably due to the failure of the acid-promoting ring-opening step. When the C3-position of indole moiety of substrate **1a** was substituted by a Cl-substituent, the corresponding [3 + 2] cyclization product **3a-1** (CCDC 2041571) was generated; however, the subsequent ring-opening/ring-expanding process of this seven-membered intermediate could not occur, suggesting that the C3-nucleophilicity of indole moiety was crucial for this cascade. Alternatively, a related 2-indole-alkenyl ester **1a-2** was used as the reactant under the standard PTC conditions, the Michael addition product **3a-2** was obtained, which could also be fully transformed into eight-membered [3.3.1] ring system **4a-2** via intramolecular Friedel-Crafts-type reaction by Lewis acid catalysis, suggesting that the Friedel–Crafts process during this cascade was legitimately existing. In addition, ^1^H NMR titration and model experiments were performed for getting further mechanistic insights (Fig. 7c). Generally, titration of 2-indole-vinyl ketone substrate **1a** to the optimal catalyst **P10** led to a distinct change in the position of thiourea-NH^1^ signal of catalyst, while titration of cyclic azomethine ylide **2a** to this catalyst **P10** led to a less pronounced change (Fig. 7c, left, also see Supplementary Fig. S7 for more details). When methylated phosphonium salt **P10’** was used as the catalyst, the reaction became slightly slower and the enantioselectivities decreased to 0% ee. In addition, when the reaction was performed in methanol, a racemic product was obtained (Fig. 7c, right). These results offered clear clues that the ion-pairing and H-bonding effects are essential for enhancing the reactivity and stereoselectivities of this cascade reaction. Furthermore, bifunctional phosphonium salt catalyst **P10** particularly possesses a semi-enclosed cavity with an electropositive region as demonstrated by its computed electrostatic potential (ESP) map (Fig. 7d), which would be advantageous to control the stereoselectivities of the initial [3 + 2] cyclization.

**Fig. 8:**
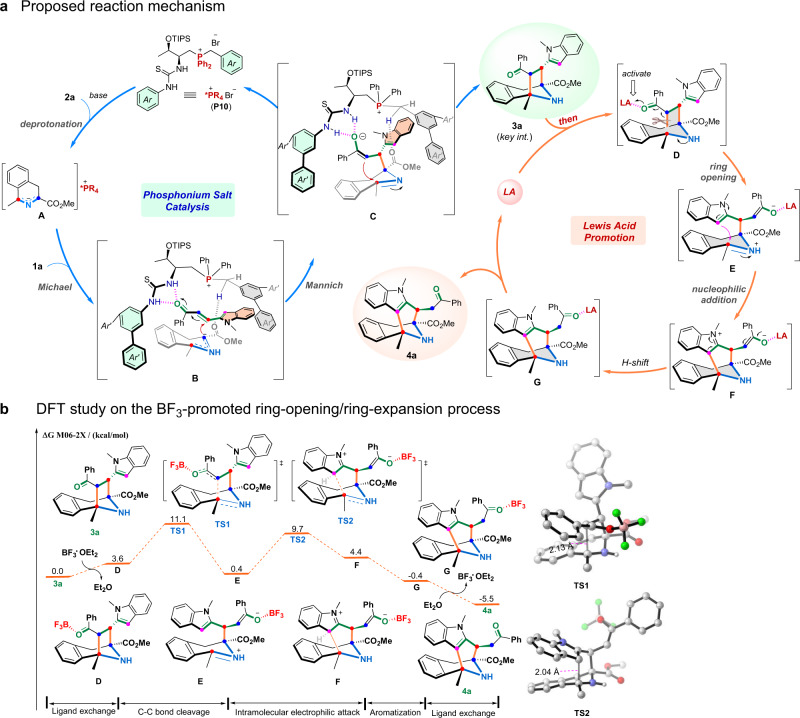
Proposed reaction mechanism and DFT study. **a** Proposed reaction mechanism. **b** Free-energy profiles for the BF_3_-catalyzed ring-opening/Friedel–Crafts cascade process. Energy values are given in kcal/mol. Bond lengths are given in angstrom.

On the basis of the above results, a plausible reaction mechanism of this reaction was proposed in Fig. 8a, which illuminated that a complex cascade process containing [3 + 2] cyclization, ring-opening, and intramolecular Friedel–Crafts-type reaction was involved in the catalytic cycle. The initial [3 + 2] cyclization in this cascade is likely to follow the general mechanism described in the literature^44^. Firstly, the reaction is initiated by the base-triggered deprotonation of cyclic azomethine ylide **2a** to give the anionic intermediate **A** that might be stabilized by phosphonium cation. Then, the Michael addition of intermediate **A** to 2-indole-vinyl ketone **1a** occurred via model **B**, and subsequently, the intramolecular Mannich process proceeded via model **C**, thus providing the key intermediate **3a**. Next, the intermediate **3a** was activated by Lewis acid catalyst to form **D**, which would result in the ring-opening process for affording intermediate **E**. Then, the intramolecular nucleophilic addition of the indole moiety to the imine unit occurred, furnishing the eight-membered *N*-bridged [3.3.1] ring intermediate **F**, and aromatization of **F** could smoothly proceed to form **G**, thus finally furnishing the desired cascade product **4a** with simultaneously releasing the Lewis acid catalyst.

To further understand the mechanism of this cascade reaction particularly on the process of acid-catalyzed ring-opening/Friedel-Crafts cascade process of intermediate **3a**, we performed density functional theory (DFT) calculations (see Supplementary Information for computational details). In our calculations, seven-membered ring complex **3a** was chosen as the model reactant, which can deliver azabicyclo[3.3.1]nonane derivative **4a** as observed experimentally, where **3a** was set as the relative zero point for computed Gibbs free energies of various intermediates and transition states. As depicted in Fig. 8b, ligand exchange between boron trifluoride ethyl ether complex and substrate **3a** gives carbonyl-coordinated complex **D** with endergonic energy of 3.6 kcal/mol, which is attributed to the slightly weaker coordination ability of the carbonyl group as compared to ethyl ether. In complex **D**, the coordination with the Lewis acidic boron trifluoride catalyst facilitates electron density flow from the N–H moiety to the carbonyl moiety in substrate **3a**, which significantly activates the C−C bond. The heterolytic cleavage of the C–C bond then smoothly occurs via transition state **TS1** with an energy barrier of 7.5 kcal/mol (11.1 kcal/mol relative to **3a**), which produces iminium species **E**. In **TS1**, the length of the breaking C–C bond is 2.13 Å. Subsequently, the intramolecular Friedel–Crafts-type reaction proceeds via transition state **TS2** with an energy barrier of 9.3 kcal/mol, generating cationic species **F**. In **TS2**, the length of the forming C–C bond is 2.04 Å. Re-aromatization and enol-keto tautomerization of **F** through proton transfers subsequently occur to afford the eight-membered ring complex **G**. This process is presumably facile and likely to be solvent-assisted, thus the transition states were not computed. Finally, the ligand exchange with diethyl ether solvent yields azabicyclo[3.3.1]nonane product **4a** with the regeneration of active catalytic species (BF_3_·Et_2_O), which is exergonic by 5.1 kcal/mol, thus completing the catalytic cycle. Of note, the calculated results show that the free energy difference between **3a** and **4a** is 5.5 kcal/mol, which indicates that the ring-opening/ring-expansion process is thermodynamically favored since the seven-membered intermediate **3a** is less stable than the eight-membered product **4a**. The calculations indicate that the C–C bond-breaking step is likely the rate-determining step in the right catalytic cycle, and the overall activation free energy for this reaction is 11.1 kcal/mol. Besides, we carried out additional DFT studies on the stereoselectivity-determining transition states (Supplementary Fig. S9), and the results listed in Supplementary Fig. S16 suggested that (*S,S,S,S*)-**3a** was the favorable cycloaddition product, which was in agreement with the experimental observation (see Supplementary Information for more details).

## Discussion

In summary, we have developed an unprecedented catalytic asymmetric protocol for the synthesis of chiral *N*-bridged [3.3.1] ring systems, which is an important family of pseudo-natural products that bear an intrinsically synthetic challenge. This is enabled by a fascinating cascade reaction via bifunctional phosphonium salt/Lewis acid relay catalysis. Given the easy availability of both 2-indole-vinyl ketone and cyclic azomethine ylide starting materials together with the simple-operating and transition-metal-free conditions, this protocol provides a general and modular platform to access a great diversity of pseudo-natural azabicyclo[3.3.1]nonane compounds and their analogs with high chemical yields and excellent stereoselectivities. Preliminary biological activity studies indicate that these *N*-bridged [3.3.1] molecules are potential anticancer agents. Experimental and computational mechanistic investigations revealed that it proceeded through a complex [3 + 2] cyclization/ring-opening/Friedel–Crafts cascade process enabled by phosphonium salt/Lewis acid metal-free relay catalysis. This study is expected to stimulate a more systematic exploration of the related organic synthesis, particularly toward the synthesis of diverse chiral pseudo-natural products for drug discovery.

## Methods

General procedure for the catalytic asymmetric synthesis of *N*-bridged [3.3.1] products. To a dried round bottle flask with a magnetic stirring bar were added 1a (26.1 mg, 0.10 mmol) and 2a (24.4 mg, 0.12 mmol), followed by the addition of Cs_2_CO_3_ (195 mg, 0.6 mmol) and catalyst P10 (13.9 mg, 0.01 mol), followed by the addition of Et_2_O (2.0 mL). The reaction mixture was stirred at −20 °C for 72 h, and TLC show that the reaction was completed. Then, BF_3_·Et_2_O (1.0 mmol) was added directly. After being stirred at room temperature for another 12 h, the reaction was quenched by saturated NaHCO_3_(aq) and extracted by ethyl acetate three times, and the combined organic phase was concentrated in vacuo, and the crude residue was purified by column chromatography on silica gel (hexane/ethyl acetate = 3/1) to afford target product 4a.

### Reporting summary

Further information on research design is available in the Nature Research Reporting Summary linked to this article.

## Supplementary information

Supplementary Information

Reporting Summary

## Data Availability

Data relating to the characterization data of materials and products, general methods, optimization studies, mechanistic studies, mass spectrometry, HPLC and NMR spectra, computational studies, and biological activity studies are available in the Supplementary Information. The X-ray crystallographic coordinates for structures of *rac*-**3a**, *rac*-**4a**, **3a**, **4a**, *ent*-**4a**, **3a-1** reported in this article have been deposited and available free of charge from the Cambridge Crystallographic Data Centre (CCDC), under deposition CCDC numbers 2089968, 2053966, 2015156, 2019560, 2047395, and 2041571, respectively. All other data are available from the corresponding author upon request.
