# Supplementary material for: Asymmetric synthesis of N-bridged [3.3.1] ring systems by phosphonium salt/Lewis acid relay catalysis
Source: Nat Commun. 2022 Jan 18;13:357. doi: 10.1038/s41467-022-28001-8 (PMC8766524; doi:10.1038/s41467-022-28001-8)
Supplement: Supplementary file 1 — Supplementary Information [file 41467_2022_28001_MOESM1_ESM.pdf]

*Supplementary Information for*

# Asymmetric synthesis of *N*-bridged [3.3.1] ring systems by phosphonium salt/Lewis acid relay catalysis

**Jian-Ping Tan,[1,3,5] Kehan Li,[1,5] Boming Shen,[2,5] Cheng Zhuang,[4] Zanjiao Liu,[1] Kai Xiao,[4] Peiyuan Yu,*[,2] Bing Yi,*[,3] Xiaoyu Ren,[1] and Tianli Wang*[,1]**

[1] *Key Laboratory of Green Chemistry & Technology of Ministry of Education, College of Chemistry, Sichuan University, Chengdu 610064, P. R. China.*

[2] *Department of Chemistry and Shenzhen Grubbs Institute, Southern University of Science and Technology, Shenzhen 518055, P. R. China.*

[3] *Hunan Province Key Laboratory of Environmental Catalysis and Waste Recycling, College of Materials and Chemical Engineering, Hunan Institute of Engineering, Xiangtan 411104,PR China.*

[4] *Precision Medicine Research Center & Sichuan Provincial Key Laboratory of Precision Medicine, West China Hospital, Sichuan University, Chengdu, 610041, P. R. China*

[5] *These authors contributed equally to this work*

e-mail: yupy@sustech.edu.cn; bingyi2004@126.com; wangtl@scu.edu.cn.

## Supplementary Methods

1. **General information --------------------------------------------------------------S2**
2. **Initial studies on reaction feasibility ----------------------------------------S3**
3. **Optimization of reaction conditions ----------------------------------------S3**
4. **Preparation of 2-vinylindoles and cyclic azomethine ylides-----------S7**
5. **Preparation of bifunctional phosphonium salts----------------------------S19**
6. **Procedure for the synthesis of *N*-bridged [3.3.1] products --------------S25**
7. **Late-stage diversification ---------------------------------------------------S101**
8. **Gram-scale preparations and synthetic applications -------------------S111**
9. **Determination of absolute configuration of products--------------------S115**
10. **Bioactive investigations------------------------------------------------------S124**
11. **Mechanism studies -------------------------------------------------------------S127**
12. **Computational investigations ----------------------------------------------S142**
13. **NMR spectra ---------------------------------------------------------------------S194**

**Supplementary References--------------------------------------------------------S300**

# Supplementary Methods

## 1. General information

All the starting materials were obtained from commercial sources and used without further purification unless otherwise stated. $^1H$ and $^{13}C$ NMR spectra were recorded on a Bruker AVANCE III HD (400 MHz) spectrometer in $CDCl_3$ or $CD_3OD$. Chemical shifts (δ) are reported in ppm, and the residual solvent peak was used as an internal reference ($CDCl_3$: δ 7.26 ppm $^1H$; δ 77.16 ppm $^{13}C$; $CD_3OD$: δ = 3.31 ppm $^1H$; δ 49.00 ppm $^{13}C$; Acetone-*d6*: δ 2.09 ppm $^1H$; δ 29.84 ppm $^{13}C$ ). Multiplicity was indicated as follows: s (singlet), d (doublet), t (triplet), q (quartet), m (multiplet), dd (doublet of doublet), br s (broad singlet). Coupling constants (*J*) were reported in Hertz (Hz). All high resolution mass spectra were obtained on a Thermo LTQ mass spectrometer. For thin layer chromatography (TLC), Merck pre-coated TLC plates (Merck 60 F254) were used, and compounds were visualized with a UV light at 254 nm. Further visualization was achieved by staining with iodine, followed by heating on a hot plate. Flash chromatographic separations were performed on Merck 60 (0.040–0.063 mm) mesh silica gel. Diastereomeric ratios were determined by crude $^1H$-NMR or HPLC analysis. Enantiomeric excesses were determined by HPLC analysis using chiral column described below in detail. Optical rotations were measured with polarimeter.

All the 2-vinylindoles **1** were synthesized following the methods reported in the literature,[1] and cyclic azomethine ylides **2** were synthesized following the previous reported literature.[2] All the phosphonium salt catalysts **P1-10** used in this study were prepared via a P-alkylation reaction of our previously reported organophosphines according to the known procedures.[3] The structure and absolute configurations of [3 + 2] cycloaddition product was assigned by X-ray crystallographic analysis of the single crystal of chiral **3a** (Supplementary Table S7); and moreover the structure and absolute configurations of the *N*-bridged [3.3.1] cyclic products were assigned by X-ray crystallographic analysis of the single crystal of chiral **4a** (Supplementary Table S8). Besides, the X-ray structures of compounds *rac*-**3a** (Supplementary Table S9), *rac*-**4a** (Supplementary Table S10), *ent*-**4a** (Supplementary Table S11), and **3a-1** (Supplementary Table S12) were also obtained, respectively.

## 2. Initial studies on reaction feasibility

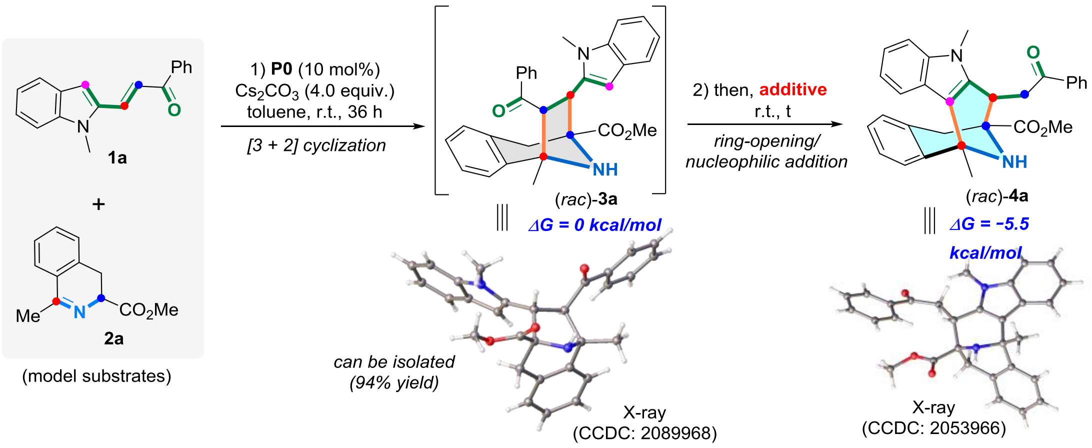


**Supplementary Table S1.** Studies on the reaction of acid promoted ring-opening/Friedel-Crafts cascade process.[a]

| entry | additive (10.0 equiv.) | T (°C), t (h) | Yield **(4a)**[b] |
|---|---|---|---|
| 1[c] | $BF_3$ ·$Et_2O$ (4.0 equiv.) | rt, 12 h | 99% (2nd step) |
| **2** | **$BF_3$ ·$Et_2O$** | **rt, 12 h** | **93%** |
| 3 | $AlCl_3$ | rt, 12 h | 91% |
| 4 | HCl (1M) | rt, 12 h | 89% |
| 5 | *p*-$NO_2$-benzoic acid | rt, 24 h | 72% |

[a]Reactions were carried out with substrate **1a** (0.1 mmol), **2a** (0.12 mmol), $Cs_2CO_3$ (0.4 mmol) and **P0** (0.01 mmol) in toluene (2.0 mL) at r.t. for 36 h. Then, the additive (1.0 mmol, 10.0 equiv.) was directly added to the reaction mixture which was further stirred at r.t.for 12-24 h. **P0** = $Ph_2Me_2P^+I^-$. [b] Isolated yield. [c] The *rac*-**3a** was firstly isolated, and then was used in the next step.

## 3. Optimization of reaction conditions

**Supplementary Table S2.** Screening chiral bifunctional phosphonium salts catalyst.[a]

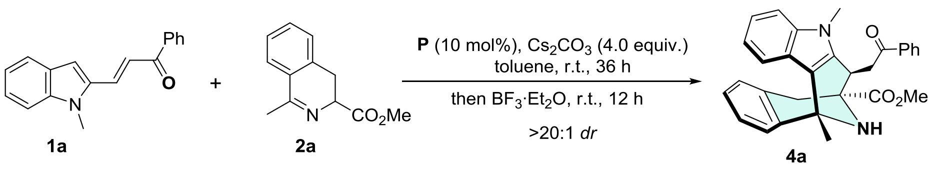

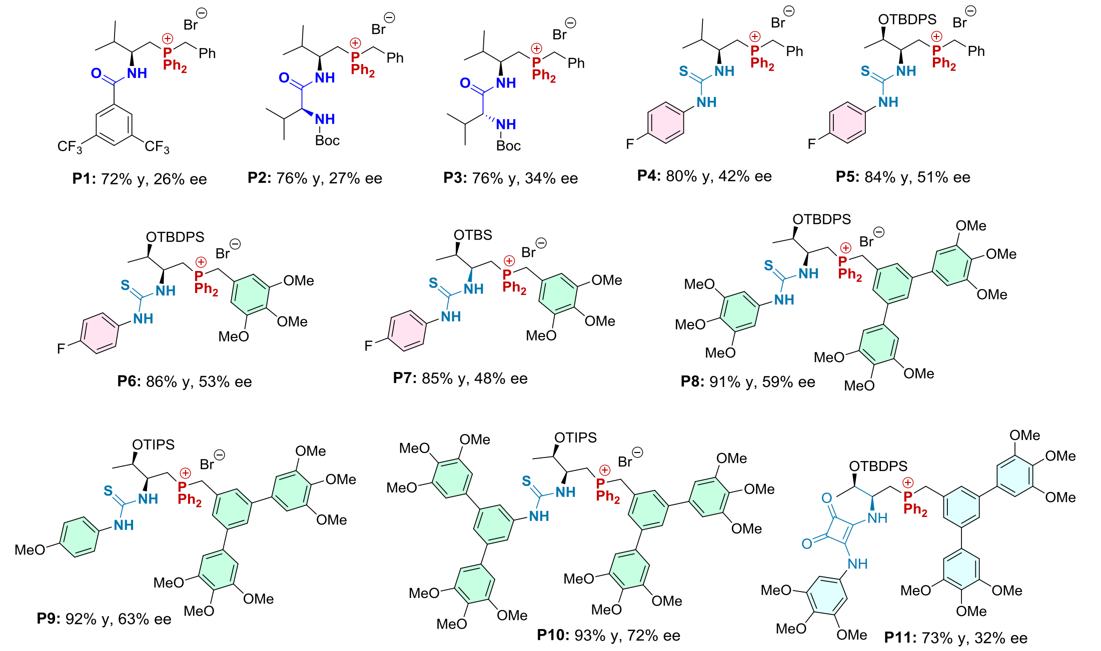


[a]Reaction conditions: **1a** (0.10 mmol) and **2a** (0.12 mmol) in the presence of **P** (0.01 mmol) and $Cs_2CO_3$ (0.40 mmol) in toluene (2.0 mL) at r.t. for 36 h; then $BF_3 \cdot Et_2O$ (1.0 mmol) was directly added to the reaction mixture which was further stirred at r.t. for 12 hours. All *dr* values were determined by $^1$H NMR and HPLC analysis. Yields of isolated products of **4a**. All ee values were determined by HPLC analysis on a chiral stationary phase. (TBS = *tert*-butylbimethylsilyl, TBDPS = *tert*-butylbiphenylsilyl, TIPS = triisopropylsilyl)

**Supplementary Table S3.** Screening of the solvent.[a]

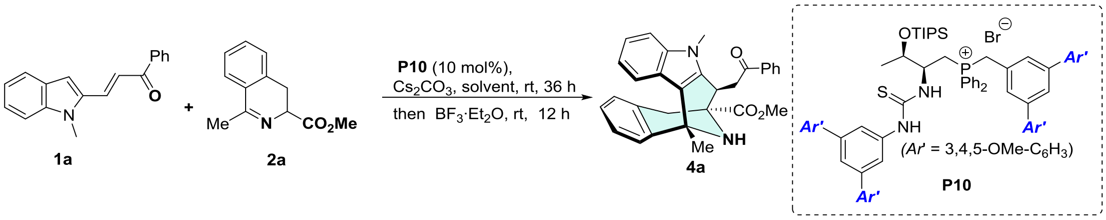


| entry | solvent | yield (%)[b] | ee (%)[c] | *dr* |
|---|---|---|---|---|
| 1 | hexane | 81 | 73 | >20:1 |
| 2 | PE (b.p.60-90 °C) | 85 | 80 | >20:1 |
| 3 | *c*-pentane | 83 | 78 | >20:1 |
| 4 | *c*-hexane | 85 | 74 | >20:1 |
| 5 | *n*-pentane | 79 | 51 | >20:1 |
| 6 | *n*-heptane | 85 | 70 | >20:1 |
| **7** | *n*-octane | 80 | 79 | >20:1 |

| | | | | |
|---|---|---|---|---|
| 8 | toluene | 84 | 81 | >20:1 |
| **9** | **$Et_2O$** | **92** | **84** | **>20:1** |
| 10 | $CH_2Cl_2$ | 75 | 18 | >20:1 |
| 11 | $CHCl_3$ | 95 | 56 | >20:1 |
| 12 | THF | 90 | 74 | >20:1 |
| 13 | xylene | 85 | 80 | >20:1 |
| 14 | *m*-xylene | 84 | 67 | >20:1 |
| 15 | MTBE | 85 | 82 | >20:1 |

[a]Reaction conditions: **1a** (0.10 mmol) and **2a** (0.12 mmol) in the presence of **P10** (0.01 mmol) and $Cs_2CO_3$ (0.40 mmol) in solvent (2.0 mL) at r.t. for 36 hours; then $BF_3 \cdot Et_2O$ (1.0 mmol) was directly added to the reaction mixture which was further stirred at r.t. for 12 hours. All *dr* values were determined by $^1H$ NMR and HPLC analysis. [b]Yields of isolated products of **4a**. [c]Determined by HPLC analysis on a chiral stationary phase. PE = petroleum ether. MTBE = methyl tert-butyl ether.

**Supplementary Table S4.** Screening of the base [a]

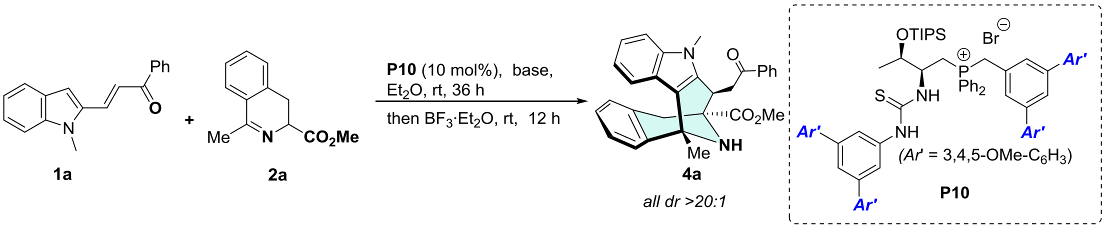


| entry | base | x.(equiv.) | yield (%)[b] | ee (%)[c] |
|---|---|---|---|---|
| 1 | $K_2CO_3$ | 4.0 | 80 | 83 |
| 2 | $K_3PO_4$ | 4.0 | 85 | 80 |
| 3 | $K_3PO_4.3H_2O$ | 4.0 | 90 | 78 |
| 4 | $K_3PO_4.7H_2O$ | 4.0 | 91 | 71 |
| 5 | $Na_2CO_3$ | 4.0 | 81 | 78 |
| 6 | $Cs_2CO_3$ | 4.0 | 92 | 84 |
| 7 | KOH | 4.0 | 95 | 13 |
| 8 | NaOH | 4.0 | 95 | 8 |
| 9 | $Et_3N$ | 4.0 | <10 | ND |
| 10 | DABCO | 4.0 | <10 | ND |

| | | | | |
|---|---|---|---|---|
| 11 | PhONa | 4.0 | 95 | 23 |
| 12 | $Na_3PO_4.12H_2O$ | 4.0 | 95 | 46 |
| 13 | $Cs_2CO_3$ | 1.0 | 71 | 72 |
| 14 | $Cs_2CO_3$ | 2.0 | 82 | 80 |
| 15 | $Cs_2CO_3$ | 3.0 | 84 | 81 |
| **16** | **$Cs_2CO_3$** | **6.0** | **93** | **88** |
| 17 | $Cs_2CO_3$ | 8.0 | 94 | 85 |
| 18 | $Cs_2CO_3$ | 10.0 | 95 | 80 |

[a] Reaction conditions: **1a** (0.10 mmol) and **2a** (0.12 mmol) in the presence of **P10** (0.01 mmol) and base (x.0 mmol) in $Et_2O$ (2.0 mL) at r.t. for 36 hours; then $BF_3 \cdot Et_2O$ (1.0 mmol) was directly added to the reaction mixture which was further stirred at r.t. for 12 hours. All *dr* values were determined by $^1H$ NMR and HPLC analysis. [b] Yields of isolated products of **4a**. [c] Determined by HPLC analysis on a chiral stationary phase. DABCO = 1,4-Diazabicyclo[2.2.2]octane. ND = Not detected.

**Supplementary Table S5**. Screening of temperature and cat.loading [a]

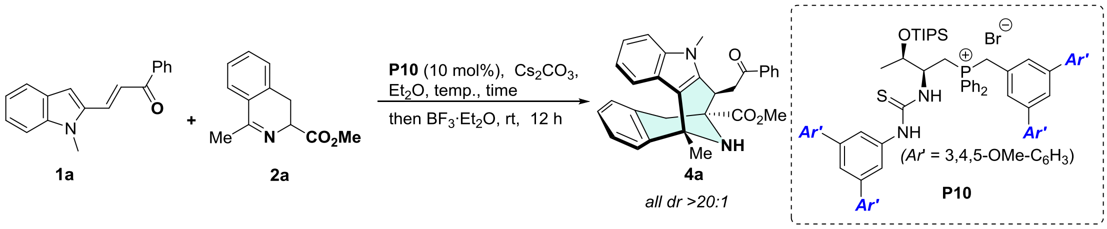


| entry | temp. | cat. loading | time (h) | yield (%)[b] | ee (%)[c] |
|---|---|---|---|---|---|
| 1 | rt | 10 mol% | 32 | 95 | 86 |
| 2 | 0°C | 10 mol% | 48 | 90 | 91 |
| 3 | -10°C | 10 mol% | 72 | 92 | 92 |
| **4** | **-20°C** | **10 mol%** | **72** | **93** | **>99** |
| 5 | -30°C | 10 mol% | 72 | <20 | ND |
| 6 | -10°C | 5 mol% | 72 | 86 | 90 |
| 7 | rt | 0 | 32 | 84 | 0 |

[a] Reaction conditions: **1a** (0.10 mmol) and **2a** (0.12 mmol) in the presence of **P10** (0.01 mmol) and $Cs_2CO_3$ (6.0 mmol) in $Et_2O$ (2.0 mL) at indicated temperature for indicated hours; then $BF_3 \cdot Et_2O$ (1.0 mmol) was directly added to the reaction mixture which was further stirred at r.t. for another 12 hours. All *dr* values were determined by $^1H$ NMR and HPLC analysis. [b] Yields of isolated products of **4a**. [c] Determined by HPLC analysis on a chiral stationary phase. ND = Not detected.

**Supplementary Table S6.** In view of the acid promoted ring-opening/Friedel-Crafts cascade process [a]

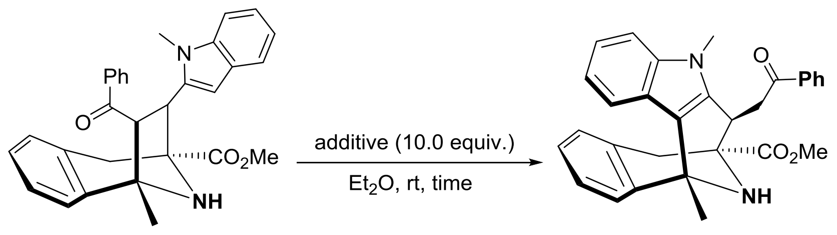


**3a**
99% ee, >20:1 dr

**4a**

| entry | additive | time(h) | yield(%)[b] | ee (%) [c] | *dr* [d] |
|---|---|---|---|---|---|
| 1 | *P*-$NO_2$-benzoic acid | 24 | 72 | 99 | >20:1 |
| 2 | *L*-proline | 24 | 71 | 99 | >20:1 |
| 3 | *D*-proline | 24 | 73 | 99 | >20:1 |
| 4 | (*R*)-binaphthyl phosphate | 24 | 70 | 98 | >20:1 |
| 5 | (*S*)-binaphthyl phosphate | 24 | 73 | 99 | >20:1 |
| **6** | $BF_3$ ·$Et_2O$ | **12** | **99** | **99** | **>20:1** |
| 7 | $AlCl_3$ | 12 | 91 | 98 | >20:1 |
| 8 | HCl(1M) | 12 | 89 | 98 | >20:1 |
| 9 | $ZnCl_2$ | 24 | 82 | 98 | >20:1 |
| 10 | $Cu(OTf)_2$ | 24 | 77 | 98 | >20:1 |
| 11 [e] | $BF_3$ ·$Et_2O$ | 48 | 58 | 99 | >20:1 |

[a] Unless otherwise stated, all reactions were performed with **3a** ( 0.05 mmol, 99% ee, >20;1 dr) in $Et_2O$ (1.0 mL) under indicated additive(0.5 mmol, 10.0 equiv.) at room temperature. [b] Yields of isolated products. [c] Determined by HPLC analysis on a chiral stationary phase. [d] Determined by $^1$HNMR and HPLC analysis on a chiral stationary phase. [e.] 1.0 equiv. $BF_3$ ·$Et_2O$ was used.

# 4. Preparation of 2-vinylindoles and cyclic azomethine ylides

## 4.1. Preparation of 2-vinyl indolyl ketones

All the 2-vinyl indolyl ketone substrates in this study were listed as follow (Supplementary Figure S1), 2-vinyl indolyl ketones **1a-1ai** were synthesized by according to the reported procedure in the literature .[1] The total yields of these compouns were 52-87%. Most of these 2-vinyl indolyl ketones were known

compounds and the unknown compounds **1o, 1r-1w, 1z-1aa, 1ad-1ah** were fully characterized.

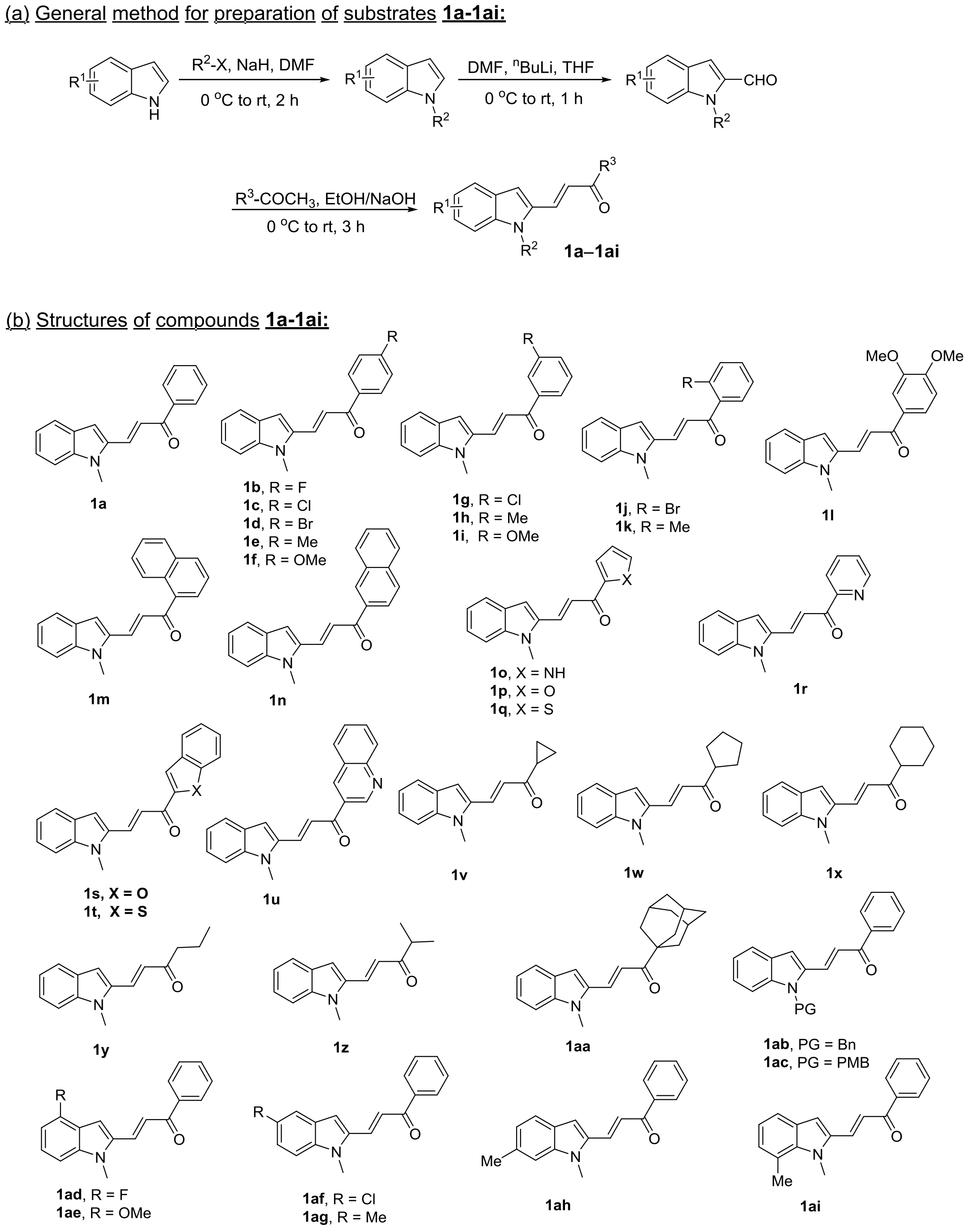


**Supplementary Figure S1**. Synthesis of 2-vinyl indolyl ketones **1a-1ai**[1].

**General procedure:** To a DMF (10 mL) solution of indole (5 mmol) was added NaH (60% dispersion in mineral oil, 7.5 mmol) at 0 ℃. The reaction mixture was stirred at the same temperature for 20 min. Then, alkyl halide (7.5 mmol) was added. The

reaction mixture was stirred at room temperature until complete consumption of starting material (detected by TLC). The reaction mixture was quenched with $NH_4Cl$, extracted with $CH_2Cl_2$ (3 × 20 mL). The combined organic layer was washed with brine, dried over sodium sulfate, filtered and evaporated. Purification via a flash column chromatography (hexanes/ EtOAc = 20/1) of the crude product provided the N-alkyl-1H-indole.

To a stirrred solution of N-alkyl-1H-indole (5 mmol) in anhydrous THF (10 mL) was added $^{n}$BuLi (6 mmol) dropwise at 0 ℃. Then, the reaction mixture was stirred up to 30 min followed by anhydrous DMF (7.5 mmol) was added dropwise. The reaction mixture was allow to warm to room temperature for 1 h. Then the reaction was quenched with $NH_4Cl$, extracted with $CH_2Cl_2$ (3 × 20 mL). The combined organic layer was washed with brine, dried over sodium sulfate, filtered and evaporated. Purification of crude product via a flash column chromatography (hexanes/EA = 30/1) provided the 1-methylindole-2-carbaldehyde.

In a 25 ml round bottom flask, ketones (2 mmol) was dissovleed in ethanol (9 mL), then 1 M NaOH (1 mL) was solwed added at 0 $^{o}$C and stirred at room temperature for 1 h. Then indole-2-carboxaldehyde derivative (2 mmol) was added, and stirred at room temperature until large mounts of yellow solids emerge from the solution. Filtrated the solution, the yellow solids was washed with EtOH to get 2-vinyl indolyl ketones product, which was further dried under vacuum.

**A. Characterization of the unknown 2-vinyl indolyl ketones**

**(E)-3-(1-methyl-1H-indol-2-yl)-1-(1H-pyrrol-2-yl)prop-2-en-1-one（1o）**

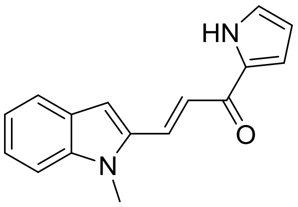


**1o**

A yellow solid; 76% yield, $^1$H NMR (400 MHz, $CDCl_3$) δ 9.91 (s, 1H), 7.97 (d, *J* = 15.2 Hz, 1H), 7.64 (d, *J* = 8.0 Hz, 1H), 7.44 (d, *J* = 15.2 Hz, 1H), 7.34 (d, *J* = 8.4 Hz, 1H), 7.29 (dd, *J* = 6.8, 1.2 Hz, 1H), 7.15 – 7.10 (m, 4H), 6.38 (dt, *J* = 4.0, 2.4 Hz, 1H), 3.87 (s, 3H); $^{13}$C NMR (100 MHz, $CDCl_3$) δ 178.45, 139.34, 135.96, 133.39, 130.18,

127.70, 125.53, 123.78, 122.30, 121.52, 120.62, 116.38, 111.21, 109.80, 103.57, 30.13; HRMS (ESI) *m/z* $C_{16}H_{14}N_2O$ calcd for $[M+H]^+$ = 251.1179, found = 251.1177.

**(E)-3-(1-methyl-1H-indol-2-yl)-1-(pyridin-2-yl)prop-2-en-1-one（1r）**

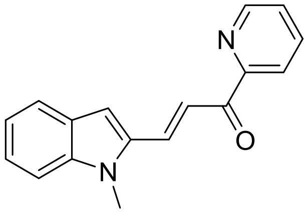

**1r**

A yellow solid; 80% yield, $^1$H NMR (400 MHz, $CDCl_3$) δ 8.68 (ddd, *J* = 4.8, 1.6, 0.8 Hz, 1H), 8.29 (d, *J* = 15.6 Hz, 1H), 8.13 (dd, *J* = 7.6, 0.8 Hz, 1H), 8.01 (d, *J* = 15.6 Hz, 1H), 7.79 (td, *J* = 7.6, 1.6 Hz, 1H), 7.57 (d, *J* = 8.0 Hz, 1H), 7.40 (ddd, *J* = 7.6, 4.8, 1.2 Hz, 1H), 7.24 – 7.19 (m, 2H), 7.17 (s, 1H), 7.07 – 7.03 (m, 1H), 3.81(s, 3H); $^{13}$C NMR (100 MHz, $CDCl_3$) δ 188.97, 154.33, 148.95, 139.59, 137.07, 136.08, 132.48, 127.68, 126.90, 124.06, 122.95, 121.73, 120.82, 120.59, 109.79, 105.19, 30.15; HRMS (ESI) *m/z* $C_{17}H_{14}N_2O$ calcd for $[M+H]^+$ = 263.1179, found = 263.1180.

**(E)-1-(benzofuran-2-yl)-3-(1-methyl-1H-indol-2-yl)prop-2-en-1-one (1s)**

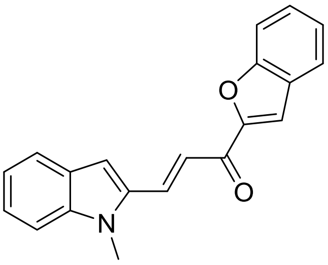

**1s**

A yellow solid; 83% yield, $^1$H NMR (400 MHz, $CDCl_3$) δ 8.07 (d, *J* = 15.2 Hz, 1H), 7.74 (d, *J* = 7.6 Hz, 1H), 7.68 – 7.63 (m, 4H), 7.52 – 7.48 (m, 1H), 7.33 (t, *J* = 7.4 Hz, 2H), 7.29 (dd, *J* = 8.4, 0.8 Hz, 1H), 7.23 (s, 1H), 7.14 (ddd, *J* = 8.0, 6.4, 1.2 Hz, 1H), 3.89 (s, 3H); $^{13}$C NMR (100 MHz, $CDCl_3$) δ 179.25, 155.92, 154.02, 139.71, 135.61, 132.34, 128.32, 127.67, 127.50, 124.35, 124.07, 123.36, 121.78, 121.05, 120.82, 113.09, 112.59, 109.93, 104.84, 30.18; HRMS (ESI) *m/z* $C_{20}H_{15}NO_2$ calcd for $[M+H]^+$ = 302.1176, found = 302.1172.

**(E)-3-(1-methyl-1H-indol-2-yl)-1-(quinolin-3-yl)prop-2-en-1-one(1u)**

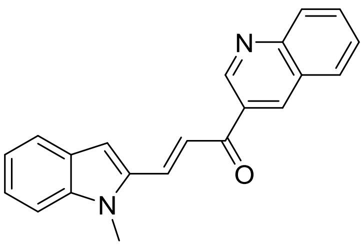

**1u**

A yellow solid; 81%yield, $^1$H NMR (400 MHz, $CDCl_3$) δ 9.53 (d, *J* = 2.0 Hz, 1H), 8.79 (d, *J* = 2.0 Hz, 1H), 8.19 (d, *J* = 8.4 Hz, 1H), 8.08 (d, *J* = 15.2 Hz, 1H), 7.98 (d, *J* = 7.6 Hz, 1H), 7.85 (ddd, *J* = 8.8, 6.8, 1.2 Hz, 1H), 7.71 (d, *J* = 15.2 Hz, 1H), 7.67-7.63 (m, 2H), 7.34 (d, *J* = 8.0 Hz, 1H), 7.32 – 7.28 (m, 1H), 7.22 (s, 1H), 7.14 (ddd, *J* = 8.0, 6.4, 1.2 Hz, 1H), 3.88 (s, 3H); $^{13}$C NMR (100 MHz, $CDCl_3$) δ 187.90, 149.80, 149.56, 139.73, 137.16, 135.46, 133.44, 132.00, 130.63, 129.62, 129.43, 127.68, 127.61, 127.08, 124.46, 121.82, 120.90, 120.80, 109.95, 104.86, 30.17; HRMS (ESI) *m/z* $C_{21}H_{16}N_2O$ calcd for $[M+H]^+$ = 313.1335, found = 313.1332.

**(E)-1-cyclopropyl-3-(1-methyl-1H-indol-2-yl)prop-2-en-1-one(1v)**

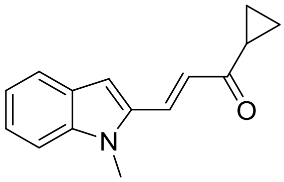

**1v**

A yellow solid; 85% yield, $^1$H NMR (400 MHz, $CDCl_3$) δ 7.73 (d, *J* = 15.6 Hz, 1H), 7.62 (d, *J* = 8.0 Hz, 1H), 7.32 (d, *J* = 8.0 Hz, 1H), 7.28 (dd, *J* = 6.8, 0.8 Hz, 1H), 7.14 –7.10 (m, 1H), 7.03 (s, 1H), 6.98 (d, *J* = 15.6 Hz, 1H), 3.84 (s, 1H), 2.22–2.16 (m, 1H), 1.21–1.13 (m, 2H), 1.00 (dq, *J* = 11.2, 3.4 Hz, 2H); $^{13}$C NMR (100 MHz, $CDCl_3$) δ 199.55, 139.43, 135.43, 129.79, 127.62, 125.94, 123.84, 121.56, 120.63, 109.77, 103.89, 30.16, 20.77, 11.66; HRMS (ESI) *m/z* $C_{15}H_{15}NO$ calcd for $[M+H]^+$ = 226.1226, found = 226.1224.

**(E)-1-cyclopentyl-3-(1-methyl-1H-indol-2-yl)prop-2-en-1-one(1w)**

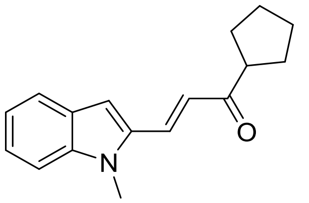

**1w**

A yellow solid; 81% yield, $^{1}$H NMR (400 MHz, $CDCl_3$) δ 7.68 (d, *J* = 15.6 Hz, 1H), 7.55 (d, *J* = 8.0 Hz, 1H), 7.24 – 7.18 (m, 2H), 7.09 – 7.02 (m, 1H), 6.95 (s, 1H), 6.82 (d, *J* = 15.6 Hz, 1H), 3.75 (s, 3H), 3.04-3.01 (m, 1H), 1.87 – 1.80 (m, 4H), 1.67 – 1.59 (m, 4H); $^{13}$C NMR (100 MHz, $CDCl_3$) δ 201.97, 139.33, 135.46, 130.16, 127.58, 125.32, 123.75, 121.48, 120.58, 109.74, 103.68, 50.70, 30.09, 29.33, 26.30; HRMS (ESI) *m/z* $C_{17}H_{19}NO$ calcd for $[M+H]^+$ = 254.1539, found = 254.1541.

**(E)-4-methyl-1-(1-methyl-1H-indol-2-yl)pent-1-en-3-one(1z)**

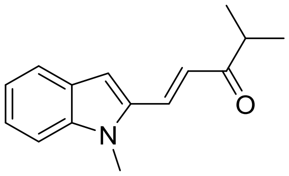

**1z**

A yellow solid; 79% yield, $^{1}$H NMR (400 MHz, $CDCl_3$) δ 7.77 (d, *J* = 15.6 Hz, 1H), 7.62 (d, *J* = 8.0 Hz, 1H), 7.32 (dd, *J* = 8.4, 0.8 Hz, 1H), 7.29 – 7.25 (m, 1H), 7.12 (ddd, *J* = 8.0, 6.8, 1.2 Hz, 1H), 7.03 (s, 1H), 6.92 (d, *J* = 15.6 Hz, 1H), 3.83 (s, 3H), 2.91-2.84 (m, 1H), 1.21 (d, *J* = 7.0 Hz, 6H); $^{13}$C NMR (100 MHz, $CDCl_3$) δ 203.25, 139.36, 135.47, 130.42, 127.59, 124.19, 123.82, 121.52, 120.62, 109.77, 103.74, 40.20, 30.12, 18.59; HRMS (ESI) *m/z* $C_{15}H_{17}NO$ calcd for $[M+H]^+$ = 228.1383 found = 228.1381.

**(E)-1-((3r,5r,7r)-adamantan-1-yl)-3-(1-methyl-1H-indol-2-yl)prop-2-en-1-one(1aa)**

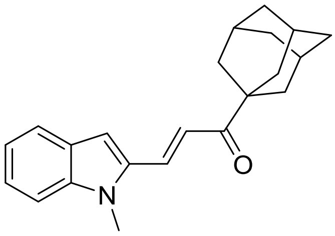

**1aa**

A yellow solid; 80% yield, $^1$H NMR (400 MHz, $CDCl_3$) δ 7.88 (d, *J* = 15.2 Hz, 1H), 7.67 (d, *J* = 8.0 Hz, 1H), 7.38 (d, *J* = 8.0 Hz, 1H), 7.35 – 7.30 (m, 2H), 7.18 (ddd, *J* = 8.0, 6.8, 0.8 Hz, 1H), 7.11 (s, 1H), 3.89 (s, 3H), 2.17 (s, 3H), 1.98 (d, *J* = 2.8 Hz, 6H), 1.89-1.80 (m, 6H); $^{13}$C NMR (100 MHz, $CDCl_3$) δ 203.60, 139.24, 135.93, 130.85, 127.62, 123.65, 121.42, 120.73, 120.55, 109.75, 103.25, 45.52, 38.27, 36.74, 30.06, 28.13; HRMS (ESI) *m/z* $C_{22}H_{25}NO$ calcd for $[M+H]^+$ = 320.2009, found = 320.2004.

**(E)-3-(4-fluoro-1-methyl-1H-indol-2-yl)-1-phenylprop-2-en-1-one(1ad)**

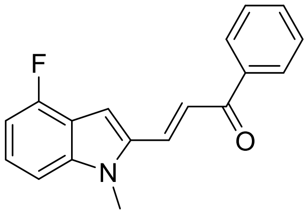


**1ad**

A yellow solid; 84% yield, $^1$H NMR (400 MHz, $CDCl_3$) δ 8.06 (dd, *J* = 8.0, 1.2 Hz, 2H), 7.96 (d, *J* = 15.2 Hz, 1H), 7.68 (d, *J* = 15.2 Hz, 1H), 7.63 – 7.59 (m, 1H), 7.55 – 7.51 (m, 2H), 7.20 (s, 1H), 7.22– 7.17 (m, 1H), 7.11 (d, *J* = 8.4 Hz, 1H), 6.79 (dd, *J* = 10.0, 8.0, 1H), 3.87 (s, 3H); $^{13}$C NMR (100 MHz,) δ 189.45, 156.74 (d, *J* = 249.4 Hz), 141.74 (d, *J* = 10.6 Hz), 138.20, 135.90, 133.11, 131.97, 128.86, 128.56, 124.36 (d, *J* = 7.8 Hz), 122.47, 117.28 (d, *J* = 22.8 Hz), 105.98 (d, *J* = 3.8 Hz), 105.14 (d, *J* = 18.8 Hz), 99.73, 30.64; $^{19}$F NMR (376 MHz, $CDCl_3$) δ -122.11; HRMS (ESI) *m/z* $C_{18}H_{15}FNO$ calcd for $[M+H]^+$ = 280.1132, found = 280.1130.

**(E)-3-(4-methoxy-1-methyl-1H-indol-2-yl)-1-phenylprop-2-en-1-one(1ae)**

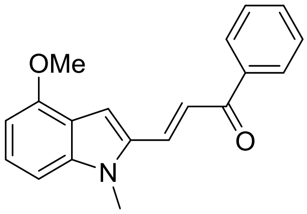


**1ae**

A yellow solid; 85% yield, $^1$H NMR (400 MHz, $CDCl_3$) δ 8.06 – 8.04 (m, 2H), 7.98 (d, *J* = 15.2 Hz, 1H), 7.64 (d, *J* = 15.2 Hz, 1H), 7.62 – 7.57 (m, 1H), 7.52 (t, *J* = 7.4 Hz , 2H), 7.28 (s, 1H), 7.21 (t, *J* = 8.0 Hz, 1H), 6.95 (d, *J* = 8.4 Hz, 1H), 6.51 (d, *J* = 7.8 Hz, 1H), 3.97 (s, 3H), 3.85 (s, 3H); $^{13}$C NMR (100 MHz, $CDCl_3$) δ 189.58, 154.00, 140.95, 138.49, 134.65, 132.87, 132.54, 128.78, 128.49, 125.12, 121.01, 118.99,

103.10, 101.83, 99.90, 55.50, 30.46; HRMS (ESI) *m/z* $C_{19}H_{17}NO_2$ calcd for $[M+H]^+$ = 292.1332, found = 292.1331.

**(E)-3-(1,5-dimethyl-1H-indol-2-yl)-1-phenylprop-2-en-1-one(1ag)**

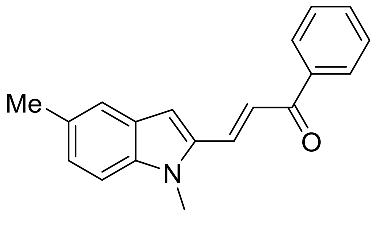


**1ag**

A yellow solid; 84%yield, $^1H$ NMR (400 MHz, $CDCl_3$) δ 8.06 – 8.04 (m, 2H), 7.99 (d, *J* = 15.2 Hz, 1H), 7.62 (d, *J* = 15.2 Hz, 1H), 7.62-7.58 (m, 1H), 7.54 – 7.50 (m, 2H), 7.41 (s, 1H), 7.23 (d, *J* = 8.4 Hz, 1H), 7.12 (dd, *J* = 8.4, 1.2 Hz, 1H), 7.06 (s, 1H), 3.86 (s, 3H), 2.45 (s, 3H); $^{13}C$ NMR (100 MHz, $CDCl_3$) δ 189.70, 138.49, 138.14, 135.80, 132.90, 132.78, 130.07, 128.80, 128.53, 127.89, 126.04, 121.34, 121.01, 109.58, 103.64, 30.21, 21.54; HRMS (ESI) *m/z* $C_{19}H_{17}NO$ calcd for $[M+H]^+$ = 276.1383, found = 276.1381.

**(E)-3-(1,6-dimethyl-1H-indol-2-yl)-1-phenylprop-2-en-1-one(1ah)**

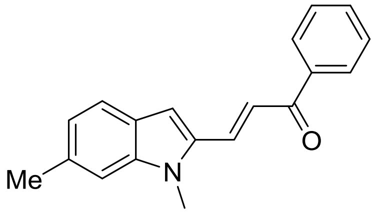


**1ah**

A yellow solid; 87% yield, $^1H$ NMR (400 MHz, $CDCl_3$) δ 8.05 (d, *J* = 7.2 Hz, 1H), 7.99 (d, *J* = 15.2 Hz, 1H), 7.62 – 7.59 (m, 2H), 7.58 – 7.50 (m, 3H), 7.12 (d, *J* = 6.4 Hz, 1H), 6.97 (d, *J* = 8.0 Hz, 1H), 3.85 (s, 3H), 2.51 (s, 3H); $^{13}C$ NMR (100 MHz, $CDCl_3$) δ 189.65, 140.04, 138.51, 135.32, 134.36, 132.84, 132.80, 128.76, 128.48, 125.58, 122.80, 121.30, 120.92, 109.69, 104.34, 30.06, 22.30; HRMS (ESI) *m/z* $C_{19}H_{17}NO$ calcd for $[M+H]^+$ = 276.1383, found = 276.1384.

## 4.2. Preparation of cyclic azomethine ylides

All the cyclic azomethine ylide substrates in this study were listed as follow (Supplementary Figure S2), all the cyclic azomethine ylides were synthesized by

according to the reported procedure in the literature.[2] Most of these cyclic azomethine ylides were known compounds and the unknown compounds **2d-2e, 2g, 2l, 2r-2t** were fully characterized.

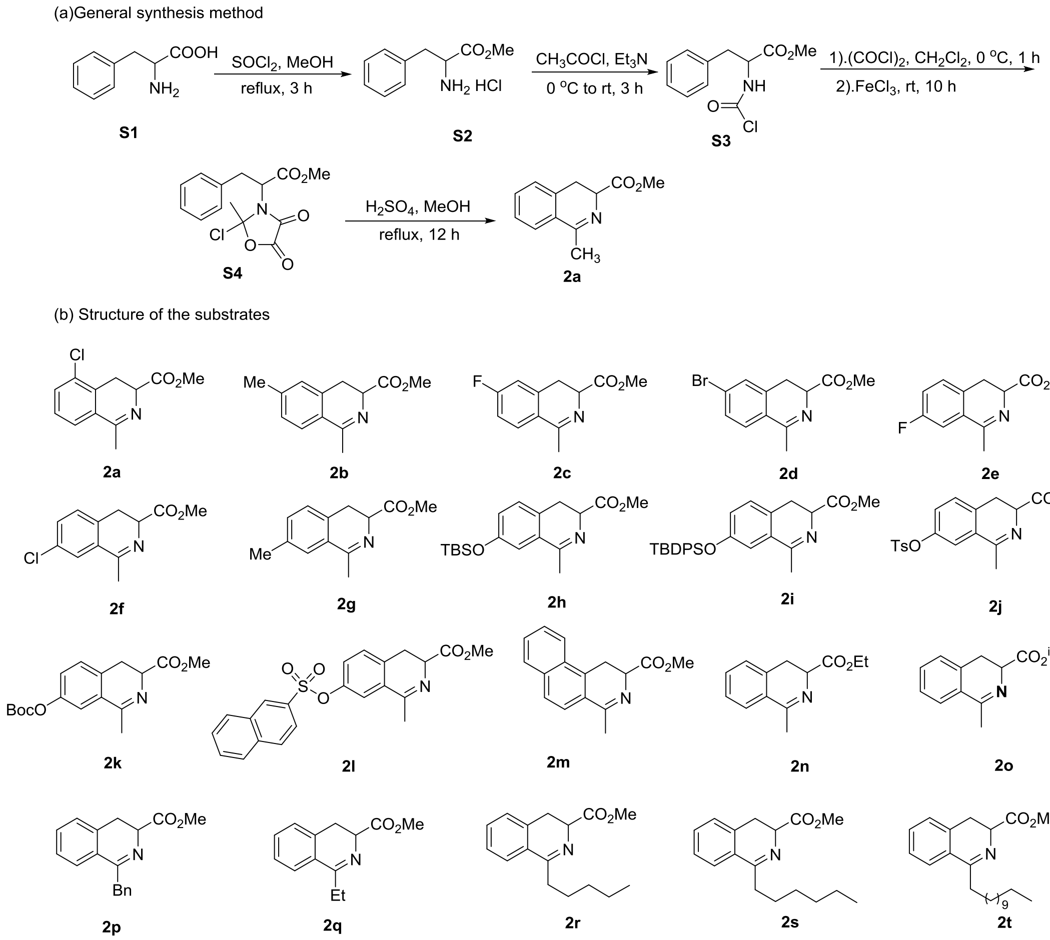


**Supplementary Figure S2**. Substrates of cyclic azomethine ylides in this study

**General procedures:** A solution of DL-Phenylalanine **S1** (1.65 g, 10.0 mmol) in MeOH (100 mL) was placed in 0 °C. Then $SOCl_2$ (2.0 equiv.) was added by dropwise slowly. The reaction mixture was reflux for 3 h. After that, the reaction mixture was cooled to room temperature and the solvent was evaporated under reduced pressure to result the crude product **S2** and used directly in the next step without purification.

A solution of **S2** (10 mmol) in $CH_2Cl_2$ (100 mL) was placed in 0 °C, then triethylamine (20 mmol) was added slowly. After that, acetyl chloride (15 mmol) was added by dropwise slowly. The reaction mixture was stirred at room temperature for 3 h and $H_2O$ was added dropwise to quench the reaction. The resulting solution was extracted with $CH_2Cl_2$ (3×10 mL). Then the combined organic phases were washed

with brine and dried over anhydrous $Na_2SO_4$. The solvent was removed to give the crude product **S3** and used directly in the next step without purification.

To a solution of **S3** (10 mmol) in $CH_2Cl_2$ (100 mL) at 0 $^oC$, then oxalyl chloride (15 mmol) was added slowly. The reaction mixture was stirred for 1 h, then $FeCl_3$ (15 mmol) was added, the mixture was warmed to room temperature and stirred for 8 h, then quenched by $H_2O$. The resulting solution was extracted with $CH_2Cl_2$ (3×10 mL). Then the combined organic phases were washed with brine and dried over anhydrous $Na_2SO_4$. The solvent was removed to give the crude product **S4** used directly in the next step without purification.

The crude product **S4** (10 mmol) in MeOH (100 mL) was placed in 0 $^oC$, then conc. $H_2SO_4$ (5 ml) was added by dropwise slowly. Then, the reaction mixture was stirred to reflux for 12 h. After that, the reaction mixture was cooled to room temperature and the solvent was evaporated under reduced pressure. The residue was added $H_2O$ and basified with concentrated ammonium hydroxide. The resulting solution was extracted with $CH_2Cl_2$ (3×10 mL). Then the combined organic phases were washed with brine and dried over anhydrous $Na_2SO_4$. The solvent was removed to give the crude product which was purified by flash column chromatography to afford the desired compound **1a**. All the cyclic azomethine ylides were synthesized by following this method with 31-82% total yields and were listed in Supplementary Figure S2**.**

**A. Characterization of the unknown cyclic azomethine ylides**

**<u>Methyl 6-bromo-1-methyl-3,4-dihydroisoquinoline-3-carboxylate(2d)</u>**

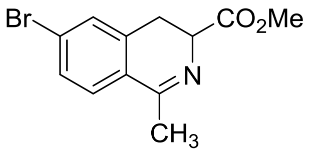


**2d**

A yellow liquid; 75% yield, $^1H$ NMR (400 MHz, $CDCl_3$) δ 7.39 (dd, *J* = 8.4, 2.0 Hz, 1H), 7.33 – 7.31 (m, 2H), 4.18 – 4.11 (m, 1H), 3.76 (s, 3H), 2.89 (d, *J* = 9.4 Hz, 2H), 2.38 (d, *J* = 2.0 Hz, 3H); $^{13}C$ NMR (100 MHz, $CDCl_3$) δ 172.85, 164.98, 137.57, 130.65, 130.56, 127.89, 127.15, 125.31, 59.55, 52.52, 28.04, 23.28; HRMS (ESI) *m/z* $C_{12}H_{12}NO_2Br$ calcd for $[M+H]^+$ = 282.0124, found = 282.0123.

**<u>Methyl 7-fluoro-1-methyl-3,4-dihydroisoquinoline-3-carboxylate(2e)</u>**

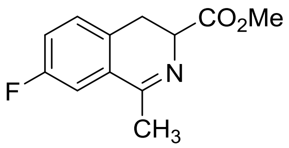

**2e**

A yellow liquid; $^{1}H$ NMR (400 MHz, $CDCl_3$) δ 7.48 – 7.44 (m, 1H), 6.95 – 6.85 (m, 2H), 4.13 (d, *J* = 9.2 Hz, 1H), 3.77 – 3.75 (m, 3H), 2,93 – 2.89 (m, 2H), 2.38 – 2.37 (m, 3H); $^{13}C$ NMR (101 MHz, $CDCl_3$) δ 173.00, 165.12, 163.70 (d, *J* = 218.6 Hz), 138.59 (d, *J* = 8.8 Hz), 127.98 (d, *J* = 9.0 Hz), 125.67 (d, *J* = 2.8 Hz), 114.71 (d, *J* = 22.0 Hz), 114.19 (d, *J* = 22.0 Hz), 59.52, 52.48, 28.49, 23.43; $^{19}F$ NMR (376 MHz, $CDCl_3$) δ -113.98; HRMS (ESI) *m/z* $C_{12}H_{12}NO_2F$ calcd for $[M+H]^+$ = 222.0907, found = 222.0906.

**Methyl 4,6-dimethyl-1,2-dihydronaphthalene-2-carboxylate (2g)**

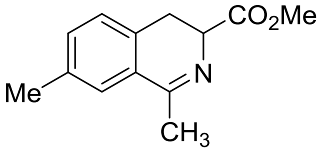

**2g**

A yellow liquid; 80% yield, $^{1}H$ NMR (400 MHz, $CDCl_3$) δ 7.26 (s, 1H), 7.12 (d, *J* = 7.2 Hz, 1H), 7.03 (d, *J* = 7.6 Hz, 1H), 4.13 – 4.08 (m, 1H), 3.74 (s, 3H), 2.86 – 2.83 (m, 2H), 2.38 (s, 3H), 2.30 (s, 3H); $^{13}C$ NMR (100 MHz, $CDCl_3$) δ 173.26 , 165.79, 136.82, 132.30, 131.63, 128.92 127.28, 126.17, 59.89, 52.23, 27.92, 23.22, 21.09; HRMS (ESI) *m/z* $C_{13}H_{15}NO_2$ calcd for $[M+H]^+$ = 218.1176, found = 218.1174.

**Methyl-1-methyl-7-((naphthalen-2-ylsulfonyl)oxy)-3,4-dihydroisoquinoline-3-carboxylate(2l)**

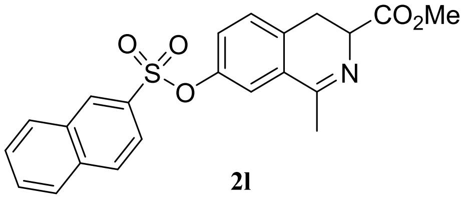

**2l**

A yellow solid; 67% yield, $^{1}H$ NMR (400 MHz, $CDCl_3$) δ 8.34 (s, 1H), 7.99 (d, *J* = 8.8 Hz, 1H), 7.91 (t, *J* = 8.4 Hz, 2H), 7.84 (d, *J* = 7.2 Hz, 1H), 7.70 – 7.59 (m, 2H), 7.09 (d, *J* = 8.4 Hz, 1H), 7.04 (d, *J* = 2.0 Hz, 1H), 6.98 – 6.95 (m, 1H), 4.15 – 4.10 (m, 1H), 3.77 (s, 3H), 2.94 – 2.80 (m, 2H), 2.15 (s, 3H); $^{13}C$ NMR (100 MHz, $CDCl_3$) δ 172.92, 164.53, 148.55, 135.45, 134.44, 131.79, 131.69, 130.67, 130.07, 129.88, 129.71, 129.41, 128.90, 128.11, 128.05, 124.96, 122.77, 119.87, 59.73, 52.60, 27.66 ,

23.08; HRMS (ESI) *m/z* $C_{22}H_{19}NO_5S$ calcd for $[M+H]^+$ = 410.1059, found = 410.1057.

**Methyl 1-pentyl-3,4-dihydroisoquinoline-3-carboxylate(2r)**

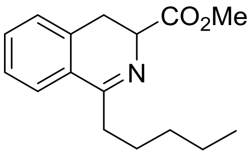


**2r**

A yellow liquid; 64% yield, $^1$H NMR (400 MHz, $CDCl_3$) δ 7.49 (dd, *J* = 7.2, 1.2 Hz, 1H), 7.35 – 7.26 (m, 2H), 7.18 (dd, *J* = 7.2, 1.2 Hz, 1H), 4.18 (t, *J* = 9.4 Hz, 1H), 3.76 (s, 3H), 2.91 (d, *J* = 9.2 Hz, 2H), 2.85 – 2.67 (m, 2H), 1.68 – 1.60 (m, 2H), 1.40 – 1.27 (m, 4H), 0.86 (t, *J* = 7.0 Hz, 3H); $^{13}$C NMR (100 MHz, $CDCl_3$) δ 173.25, 168.77, 135.85, 130.77, 128.50, 127.70, 127.26, 125.31, 59.68, 52.19, 36.08, 31.70, 28.53, 27.18, 22.40, 13.95; HRMS (ESI) *m/z* $C_{16}H_{21}NO_2$ calcd for $[M+H]^+$ = 260.1647, found = 260.1648.

**methyl 1-hexyl-3,4-dihydroisoquinoline-3-carboxylate(2s)**

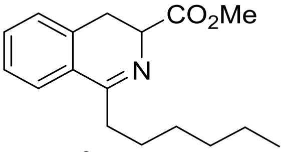


**2s**

A yellow liquid; 68% yield, $^1$H NMR (400 MHz, $CDCl_3$) δ 7.49 (d, *J* = 7.6 Hz, 1H), 7.34 – 7.28 (m, 2H), 7.18 (d, *J* = 7.2 Hz, 1H), 4.18 (t, *J* = 9.2 Hz, 1H), 3.76 (s, 3H), 2.91 (d, *J* = 9.6 Hz, 2H), 2.85 – 2.67 (m, 2H), 1.67 – 1.60 (m, 2H), 1.38 – 1.25 (m, 8H), 0.85 (t, *J* = 6.2 Hz, 3H); $^{13}$C NMR (100 MHz, $CDCl_3$) δ 173.07, 168.60, 135.69, 130.59, 128.34, 127.53, 127.09, 125.15, 59.52, 52.02, 35.95, 31.51, 29.28, 28.84, 28.37, 27.33, 22.37, 13.83; HRMS (ESI) *m/z* $C_{17}H_{23}NO_2$ calcd for $[M+H]^+$ = 274.1804, found = 274.1801.

**Methyl 1-undecyl-3,4-dihydroisoquinoline-3-carboxylate(2t)**

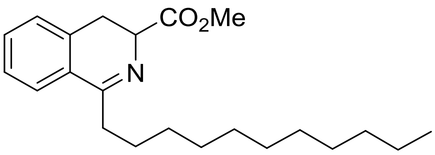


**2t**

A yellow liquid; 55% yield, $^1$H NMR (400 MHz, $CDCl_3$) δ 7.48 (d, *J* = 7.2 Hz, 1H), 7.34 – 7.17 (m, 2H), 7.18 (d, *J* = 6.8 Hz, 1H), 4.20 (t, *J* = 9.0 Hz, 1H), 3.76 (s, 3H),

2.91 (d, $J$ = 9.2 Hz, 2H), 2.84 – 2.67 (m, 2H), 1.64 – 1.59 (m, 21H), 1.22 (s, 16H), 0.85 (t, $J$ = 6.0 Hz, 3H); $^{13}$C NMR (100 MHz, $CDCl_3$) δ 173.29, 169.03, 135.89, 130.83, 128.53, 127.74, 127.29, 125.41, 59.65, 52.28, 36.18, 31.87, 29.57, 29.54, 29.51, 29.38, 29.30, 28.59, 27.60, 22.64, 14.07; HRMS (ESI) *m/z* $C_{22}H_{33}NO_2$ calcd for $[M+H]^+$ = 344.2586, found = 344.2584.

## 5. Preparation of bifunctional phosphonium salts

All the phosphonium salt catalysts in this studies were listed in Supplementary Figure S3, which were prepared by following our previously reported procedures.[3] **P1-P6** were known compounds. The unknown compounds **P7, P8, P9, P10, P11, P10', *ent*-P10** were fully characterized.

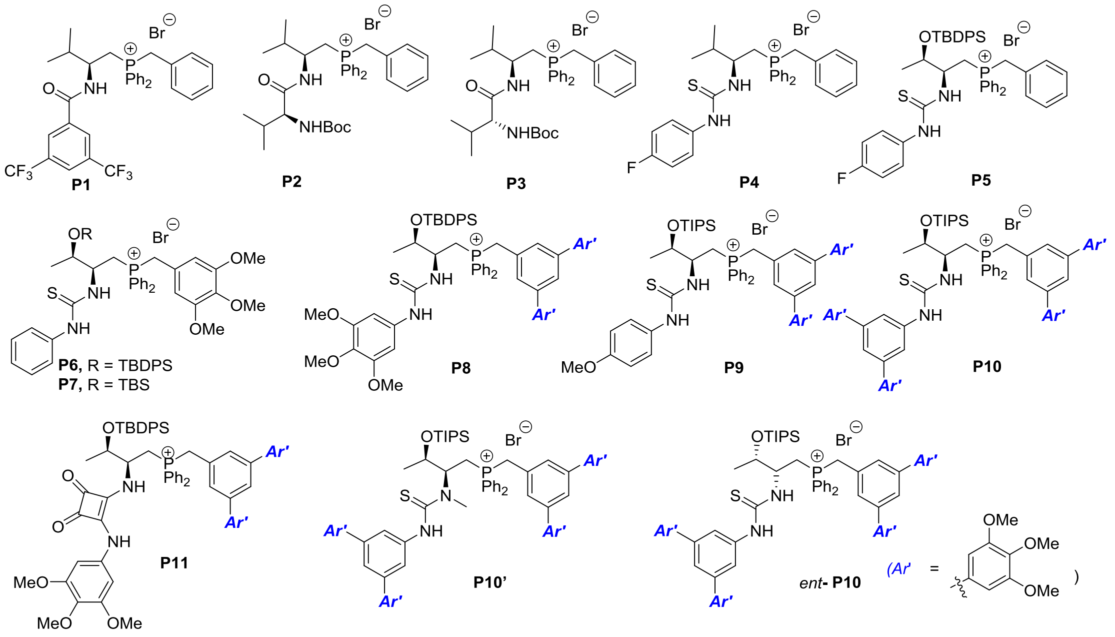


**Supplementary Figure S3.** Bifunctional phosphonium salt catalysts in this study.

### A. Characterization of the unknown phosphonium salts

**<u>((2S,3R)-3-((tert-butyldimethylsilyl)oxy)-2-(3-(4-fluorophenyl)thioureido)butyl)diphenyl(3,4,5-trimethoxybenzyl)phosphonium bromide (P7)</u>**

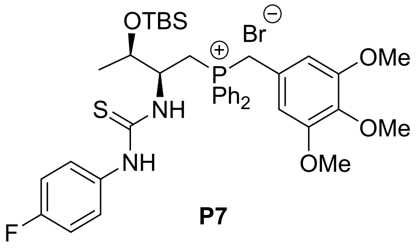

**P7**

A white solid; $^1H$ NMR (400 MHz, $CDCl_3$) δ 9.41 (d, *J* = 8.4 Hz, 1H), 9.15 (s, 1H), 7.90 (dd, *J* = 12.0, 7.2 Hz, 2H), 7.74 (q, *J* = 6.4 Hz, 2H), 7.69 – 7.61 (m, 4H), 7.60 – 7.54 (m, 2H), 7.46 (dd, *J* = 8.8, 4.8 Hz, 2H), 6.92 (t, *J* = 8.6, 2H), 6.06 (d, *J* = 2.4 Hz, 2H), 5.11 (t, *J* = 14.6 Hz, 1H), 4.20 – 4.13 (m, 2H), 3.86 – 3.74 (m, 2H), 3.71 (s, 3H), 3.47 (s, 6H), 2.90 (t, *J* = 14.4 Hz, 1H), 1.24 (d, *J* = 6.4 Hz, 3H), 0.74 (s, 9H), 0.00 (s, 3H), -0.10 (s, 3H); $^{13}C$ NMR (100 MHz, $CDCl_3$) δ 181.16, 161.23, 158.80, 153.33 (d, *J* = 3.6 Hz), 137.99 (d, *J* = 4.6 Hz), 135.06 (dd, *J* = 6.8, 2.8 Hz), 134.90 (d, *J* = 2.8 Hz), 134.23 (d, *J* = 9.4 Hz), 133.76 (d, *J* = 8.8 Hz), 130.06 (dd, *J* = 18.6, 12.2 Hz), 125.95 (d, *J* = 8.2 Hz), 122.42 (d, *J* = 9.6 Hz), 118.07, 117.12 (d, *J* = 20.8 Hz), 116.20 , 115.04 (d, *J* = 22.6 Hz), 107.67 (d, *J* = 5.6 Hz), 68.20 (d, *J* = 12.8 Hz), 60.93 (d, *J* = 2.3 Hz), 56.03, 52.80 (d, *J* = 4.0 Hz), 29.79 (d, *J* = 44.2 Hz), 25.85, 21.89 (d, *J* = 51.5 Hz), 17.94, 17.08, -4.74 (d, *J* = 29.0 Hz); $^{31}P$ NMR (162 MHz, $CDCl_3$) δ 26.38; HRMS (ESI) *m/z* calcd for $C_{39}H_{51}FN_2O_4PSSi^+$ $[M\text{-}Br]^+$ = 721.3055, found = 721.3050.

**((2S,3R)-3-((tert-butyldiphenylsilyl)oxy)-2-(3-(3,4,5-trimethoxyphenyl)thioureido)butyl)((3,3'',4,4'',5,5''-hexamethoxy-[1,1':3',1''-terphenyl]-5'-yl)methyl)diphenylphosphonium bromide (P8)**

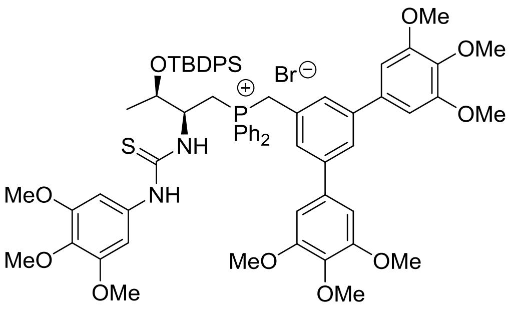


**P8**

A white solid; $^1H$ NMR (400 MHz, $CDCl_3$) δ 9.40 (d, *J* = 8.4 Hz, 1H), 9.34 (s, 1H), 8.09 (dd, *J* = 12.2, 7.8 Hz, 2H), 7.89 – 7.77 (m, 4H), 7.77 – 7.61 (m, 10H), 7.48 (t, *J* = 7.0 Hz, 2H), 7.44 (d, *J* = 7.6 Hz, 2H), 7.40 (d, *J* = 7.6 Hz, 3H), 6.99 (s, 2H), 6.64 (s, 4H), 5.48 (t, *J* = 14.6 Hz, 2H), 4.57 (t, *J* = 14.4 Hz, 1H), 4.40 (s, 1H), 4.22 – 4.14 (m, 1H), 3.98 (s, 18H), 3.87 (s, 3H), 3.84 (s, 6H), 3.15(t, *J* = 14.2 Hz, 1H ), 1.27 (d, *J* = 6.0 Hz, 3H), 1.05 (s, 9H); $^{13}C$ NMR (100 MHz, $CDCl_3$) δ 180.35, 153.42, 152.50, 142.46 (d, *J* = 3.1 Hz), 138.12, 135.62, 135.38, 134.91, 134.77, 134.04 (d, *J* = 9.4 Hz), 133.66 (d, *J* = 8.9 Hz), 133.26, 133.00, 130.01 (d, *J* = 12.3 Hz), 129.77 (d, *J* = 11.5 Hz), 128.49 (d, *J* = 9.0 Hz), 127.85 (d, *J* = 5.3 Hz), 127.66 (d, *J* = 7.4 Hz), 125.55 (d,

*J* = 3.4 Hz), 117.71, 116.86, 116.78, 115.96, 104.27, 100.91, 69.60 (d, *J* = 12.8 Hz), 60.84, 60.24, 56.33, 55.36 (d, *J* = 3.5Hz), 29.26 (d, *J* = 43.9 Hz), 26.99, 22.68 (d, *J* = 52.4 Hz),, 20.92, 19.05 , 16.96, 14.08; $^{31}$P NMR (162 MHz, $CDCl_3$) δ 26.33; HRMS (ESI) *m/z* calcd for $C_{67}H_{76}N_2O_{10}PSSi^+$ $[M\text{-}Br]^+$ = 1159.4722, found = 1159.4721.

**((3,3'',4,4'',5,5''-hexamethoxy-[1,1':3',1''-terphenyl]-5'-yl)methyl)((2S,3R)-2-(3-(4-methoxyphenyl)thioureido)-3-((triisopropylsilyl)oxy)butyl)diphenylphosphonium bromide (P9)**

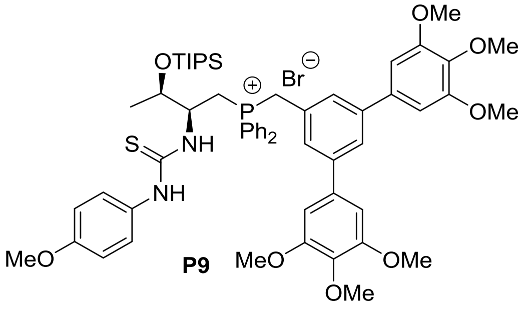

**P9**

A white solid; $^{1}$H NMR (400 MHz, $CDCl_3$) δ 9.33 (s, 1H), 8.95 (s, 1H), 7.99 (dd, *J* = 12.4, 7.6 Hz, 2H), 7.74 –7.67(m, 3H), 7.66 – 7.59 (m, 3H), 7.54-7.48 (m, 3H), 7.32 (d, *J* = 8.8 Hz, 2H), 7.07 (s, 2H), 6.73 (d, *J* = 9.2 Hz, 2H), 6.48 (s, 4H), 5.36 (t, *J* = 14.8 Hz, 1H), 5.26 – 5.14 (m, 1H), 4.46 (t, *J* = 14.4 Hz, 1H), 4.27 – 4.24 (m, 1H), 4.03 – 3.93 (m, 1H), 3.82 (d, *J* = 4.0 Hz, 18H), 3.68 (s, 3H), 2.95 (t, *J* = 14.0 Hz, 1H), 1.31 (d, *J* = 6.4 Hz, 3H), 0.91-0.88 (m, 21H); $^{13}$C NMR (100 MHz, $CDCl_3$) δ 181.29, 157.01, 153.41, 142.40 (d, *J* = 3.2 Hz), 138.08, 135.45, 134.92 (d, *J* = 2.4 Hz), 134.74 (d, *J* = 2.6 Hz), 134.21 (d, *J* = 9.4 Hz), 133.74 (d, *J* = 8.8 Hz), 131.89 (dd, *J* = 10.2, 6.5 Hz), 130.05 (d, *J* = 12.2 Hz), 129.67 (d, *J* = 12.2 Hz), 128.61 (d, *J* = 9.2 Hz), 128.51 (dd, *J* = 19.8, 10.6 Hz), 127.90 (d, *J* = 5.8 Hz), 126.08, 125.48 (d, *J* = 3.2 Hz) , 117.83, 116.96 (d, *J* = 3.0 Hz), 116.13, 113.47, 104.27, 68.25 (d, *J* = 12.8 Hz), 60.84 (d, *J* = 1.8 Hz), 56.34 (d, *J* = 1.8 Hz), 55.26, 52.87, 29.04 (d, *J* = 44.0 Hz), 22.16 (d, *J* = 52.2 Hz), 17.94 (d, *J* = 11.2 Hz), 17.24, 12.01; $^{31}$P NMR (162 MHz, $CDCl_3$) δ 27.13; HRMS (ESI) *m/z* calcd for $C_{58}H_{74}N_2O_8PSSi^+$ $[M\text{-}Br]^+$ = 1017.4667, found = 1017.4662.

**((3,3'',4,4'',5,5''-hexamethoxy-[1,1':3',1''-terphenyl]-5'-yl)methyl)((2S,3R)-2-(3-(3,3'',4,4'',5,5''-hexamethoxy-[1,1':3',1''-terphenyl]-5'-yl)thioureido)-3-((triisopropylsilyl)oxy)butyl)diphenylphosphonium bromide (P10)**

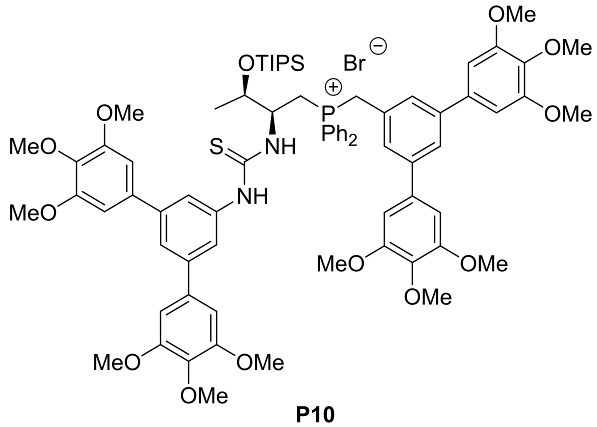


**P10**

A white solid; $^{1}$H NMR (400 MHz, $CDCl_3$) δ 9.64 (s, 1H), 9.40 (s, 1H), 8.00 (dd, *J* = 12.4, 7.6 Hz, 2H), 7.76 (s, 2H), 7.71 (dd, *J* = 12.0, 7.6 Hz, 4H), 7.64 (d, *J* = 6.4 Hz, 2H), 7.54 (td, *J* = 7.4, 2.8 Hz, 2H), 7.48 (s, 1H), 7.36 (s, 1H), 7.07 (s, 2H), 6.75 (s, 4H), 6.45 (s, 4H), 5.36 (t, *J* = 14.2 Hz, 1H), 5.22 (td, *J* = 12.8, 4.0 Hz, 1H), 4.44 (t, *J* = 14.2 Hz, 1H), 4.31 (s, 1H), 3.99 (t, *J* = 11.6 Hz, 1H), 3.82 – 3.80 (m, 36H), 2.99 (t, *J* = 14.0 Hz, 1H), 1.35 (d, *J* = 6.0 Hz, 3H), 0.90 (s, 21H); $^{13}$C NMR (100 MHz, $CDCl_3$) δ 180.74, 153.39, 153.22, 142.41 (d, *J* = 3.0 Hz), 141.86, 139.62 , 138.11, 137.57, 136.81, 135.29, 134.89 (d, *J* = 9.4 Hz), 134.18 (d, *J* = 9.4 Hz), 133.70 (d, *J* = 8.8 Hz), 130.07 (d, *J* = 12.2 Hz), 129.72 (d, *J* = 12.2 Hz), 128.40 (d, *J* = 9.2 Hz), 127.83 (d, *J* = 5.4 Hz), 125.48, 122.17, 121.35, 117.70, 116.82, 116.00, 104.59, 104.22, 68.10 (d, *J* = 12.6 Hz), 60.77, 56.29 (d, *J* = 1.4 Hz), 56.12 (d, *J* = 1.2 Hz), 52.69 (d, *J* = 4.0 Hz), 29.24 (d, *J* = 44.2 Hz), 22.20, 21.98, 17.99, 17.87, 17.23, 12.01; $^{31}$P NMR (162 MHz, $CDCl_3$) δ 27.02; HRMS (ESI) *m/z* calcd for $C_{75}H_{92}N_2O_{13}PSSi^+$ $[M\text{-}Br]^+$ = 1319.5822, found = 1319.5818.

**<u>((2S,3R)-3-((tert-butyldiphenylsilyl)oxy)-2-((3,4-dioxo-2-((3,4,5-trimethoxyphenyl)amino)cyclobut-1-en-1-yl)amino)butyl)((3,3'',4,4'',5,5''-hexamethoxy-[1,1':3',1''-terphenyl]-5'-yl)methyl)diphenylphosphonium bromide (P11)</u>**

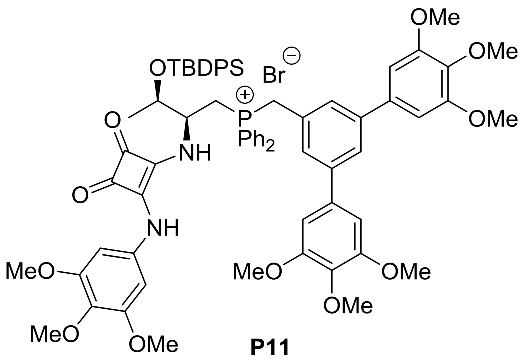


**P11**

A white solid; $^1$H NMR (400 MHz, $CDCl_3$) δ 9.75 (s, 1H), 8.74 (d, *J* = 7.2 Hz, 1H), 7.82 (dd, *J* = 12.4, 7.6 Hz, 2H), 7.72 – 7.64 (m, 2H), 7.63 – 7.44 (m, 12H), 7.36 – 7.26 (m, 2H), 7.16-7.24 (m, 6H), 6.94 (d, *J* = 4.8 Hz, 2H), 6.55 (s, 4H), 5.03 (t, *J* = 14.6 Hz, 1H), 4.56 (d, *J* = 10.4 Hz, 1H), 4.23 (t, *J* = 13.8 Hz, 1H), 3.98 (s, 1H), 3.76-3.85 (m, 24H), 3.70-3.72 (m, 3H), 2.81 – 2.64 (m, 1H), 1.07 (d, *J* = 5.9 Hz, 3H), 0.90 (s, 9H).; $^{13}$C NMR (101 MHz, $CDCl_3$) δ 182.03, 180.30, 167.13, 164.82, 153.48 (d, *J* = 5.2 Hz), 142.85, 138.21, 135.66 (d, *J* = 4.2 Hz), 135.19, 135.14, 133.76 (d, *J* = 9.6 Hz), 133.45, 133.36, 132.83, 132.68, 130.32 (d, *J* = 4.8 Hz), 130.20 (d, *J* = 4.5 Hz), 129.89 (d, *J* = 3.9 Hz), 127.86, 127.66, 127.62, 127.56, 125.97, 116.85, 116.74, 115.97 (d, *J* = 9.2 Hz), 104.29, 95.63, 71.89 (d, *J* = 12.4 Hz), 60.84, 60.78, 56.36, 56.03, 53.65 (d, *J* = 4.0 Hz), 30.92, 30.45, 26.92, 21.05 (d, *J* = 54.4 Hz), 18.90, 17.64, 14.06; $^{31}$P NMR (162 MHz, $CDCl_3$) δ 23.26; HRMS (ESI) *m/z* calcd for $C_{70}H_{76}N_2O_{12}PSi^+$ $[M-Br]^+$ = 1195.4900, found = 1195.4907.

**((2S,3R)-2-(3-(3,3'',4,4'',5,5''-hexamethoxy-[1,1':3',1''-terphenyl]-5'-yl)-1-methylthioureido)-3-((triisopropylsilyl)oxy)butyl)((3,3'',4,4'',5,5''-hexamethoxy-[1,1':3',1''-terphenyl]-5'-yl)methyl)diphenylphosphonium bromide (P10')**

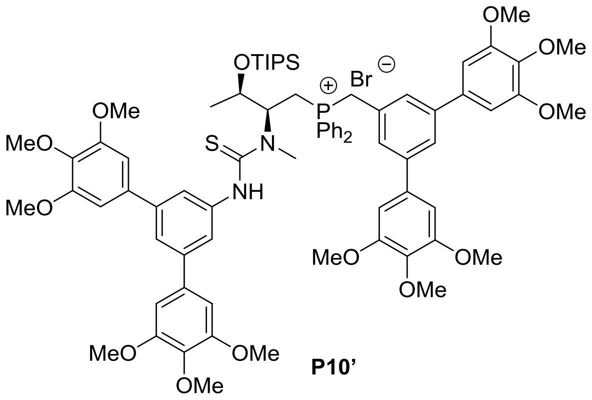

**P10'**

A white solid; $^1$H NMR (400 MHz, $CDCl_3$) δ 8.22 (dd, *J* = 12.0, 8.0 Hz, 2H), 8.14 (s, 1H), 7.80 (dd, *J* = 12.0, 8.0 Hz, 2H), 7.75 – 7.71 (m, 4H), 7.62 – 7.57 (m, 5H), 7.45 (s, 2H), 7.42 (s, 1H), 6.84 (s, 4H), 6.60 (s, 5H), 5.70 (t, *J* = 14.4 Hz, 1H), 4.92 (dd, *J* = 26.6, 11.4 Hz, 1H), 4.39 (t, *J* = 14.0 Hz, 1H), 4.14 – 4.08 (m, 1H), 3.84 (dd, *J* = 4.8, 2.4 Hz, 36H), 3.46 (q, *J* = 7.2 Hz, 1H), 3.13 (s, 3H), 3.07 (t, *J* = 15.6 Hz, 1H), 1.24 (d, *J* = 6.8 Hz, 3H), 1.20-1.14 (m, 2H), 0.98 – 0.92 (m, 18H); $^{13}$C NMR (100 MHz, $CDCl_3$) δ 182.42, 153.56, 153.45, 142.34 (d, *J* = 3.0 Hz), 141.57, 140.26, 138.18, 137.69, 137.06, 135.51, 135.06, 134.86 (d, *J* = 3.2 Hz), 133.99 (d, *J* = 9.2 Hz), 133.80 (d, *J* = 8.6 Hz), 132.15, 132.05, 129.85 (d, *J* = 12.2 Hz), 128.63, 128.57, 128.11 (d, *J*

= 4.2 Hz), 125.29, 123.85, 122.59, 104.79, 104.37, 65.89, 60.97, 58.35, 58.27, 56.53, 56.38, 35.29, 19.71, 18.29, 18.11, 15.32, 12.67; $^{31}$P NMR (162 MHz, $CDCl_3$) δ 24.14; HRMS (ESI) *m/z* calcd for $C_{76}H_{94}N_2O_{13}PSSi^+$ $[M-Br]^+$ = 1333.5978, found = 1333.5969.

**((3,3'',4,4'',5,5''-hexamethoxy-[1,1':3',1''-terphenyl]-5'-yl)methyl)((2R,3S)-2-(3-(3,3'',4,4'',5,5''-hexamethoxy-[1,1':3',1''-terphenyl]-5'-yl)thioureido)-3-((triisopropylsilyl)oxy)butyl)diphenylphosphonium bromide (*ent*-P10)**

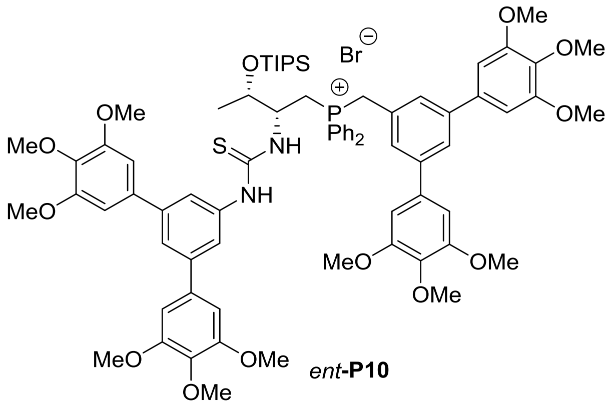

*ent*-**P10**

A white solid; $^{1}$H NMR (400 MHz, $CDCl_3$) δ 9.42 (s, 1H), 9.27 (s, 1H), 7.73 (s 2H), 7.76 (s, 2H), 7.70 – 7.64 (m, 4H), 7.59 (dd, *J* = 8.6, 5.8 Hz, 2H), 7.52 (dd, *J* = 9.0, 6.2 Hz, 2H), 7.46 (s, 1H), 7.32 (s, 1H), 7.08 (s, 2H), 6.72 (s, 4H), 6.45 (s, 4H), 5.32 – 5.19 (m, 2H), 4.37 (t, *J* = 14.2 Hz, 1H), 4.26 (s, 1H), 4.00 – 3.94 (m, 1H), 3.77 (d, *J* = 7.6 Hz, 36H), 3.00 (t, *J* = 14.2 Hz, 1H), 1.31 (d, *J* = 6.0 Hz, 3H), 0.91-0.86 (m, 21H); $^{13}$C NMR (100 MHz, $CDCl_3$) δ 180.47, 153.06 (d, *J* = 14.6 Hz), 142.09, 141.54, 139.48, 137.80, 137.31, 136.60, 135.11, 134.60, 133.95 (d, *J* = 9.2 Hz), 133.53 (d, *J* = 9.2 Hz), 131.61(d, *J* = 4.0 Hz), 129.82 (d, *J* = 12.2 Hz), 129.52 (d, *J* = 12.2 Hz), 128.21 (d, *J* = 8.6 Hz), 127.60 (d, *J* = 4.6 Hz), 125.12 (d, *J* = 4.2 Hz), 121.72 (d, *J* = 1.8 Hz), 121.01, 117.33, 116.72, 116.48, 115.90, 104.33, 103.92, 60.50 (d, *J* = 2.4 Hz), 59.94, 56.05, 55.86, 52.45, 20.60, 17.77, 17.65, 17.03, 13.82, 11.82; $^{31}$P NMR (162 MHz, $CDCl_3$) δ 26.47; HRMS (ESI) *m/z* calcd for $C_{75}H_{92}N_2O_{13}PSSi^+$ $[M-Br]^+$ = 1319.5822, found = 1319.5825.

## 6. Procedure for the synthesis of *N*-bridged [3.3.1] products

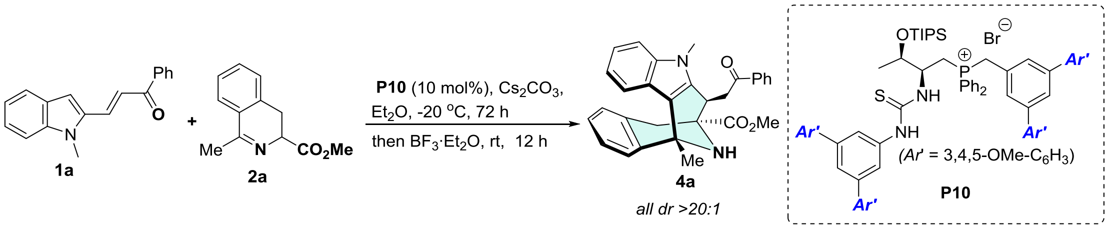


**General procedures:** To a dried round bottle flask with a magnetic stirring bar were added **1a** (26.1 mg, 0.10 mmol) and **2a** (24.4 mg, 0.12 mmol), followed by the addition of $Cs_2CO_3$ ( 195 mg, 0.6 mmol ) and catalyst **P10** (13.9 mg, 0.01 mol), followed by the addition of $Et_2O$ (2.0 mL). The reaction mixture was stirred at -20 $^oC$ for 72 h, and TLC show that the reaction was completed. Then, $BF_3$ $Et_2O$ (1.0 mmol) was added directly. After stirred at room temperature for another 12 h, the reaction was quenched by saturated $NaHCO_3$ (aq) and extracted by ethyl acetate three times, and the combined organic phase was concentrated *in vacuo*, and the crude residue was purified by column chromatography on silica gel (hexane/ethyl acetate = 3/1) to afford target product **4a**.

**Methyl(*6S,7S,13R*)-8,13-dimethyl-7-(2-oxo-2-phenylethyl)-5,7,8,13-tetrahydro-6H-6,13-epiminobenzo[4,5]cycloocta[1,2-b]indole-6-carboxylate (4a)**

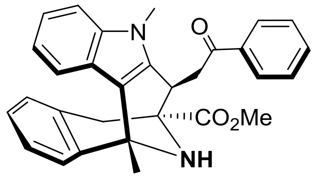


**4a**

A white solid; 43.1 mg, 93% yield; m.p. = 173.6-174.4 $^oC$, $[\alpha]^{25}_D$ = -95.64 (*c* 0.47, $CHCl_3$); *dr* >20:1; $^1H$ NMR (400 MHz, $CDCl_3$) δ 8.02 (d, *J* = 7.2 Hz, 2H), 7.70 (d, *J* = 7.2 Hz, 1H), 7.65 (d, *J* = 7.8 Hz, 1H), 7.57 (dd, *J* = 10.4, 4.2 Hz, 1H), 7.47 (t, *J* = 7.6 Hz, 1H), 7.25 – 7.18 (m, 2H), 7.14 (t, *J* = 7.6 Hz, 1H), 7.10 – 7.03 (m, 2H), 6.95 (d, *J* = 7.4 Hz, 1H), 4.24 (dd, *J* = 6.1, 3.0 Hz, 1H), 3.97 (dd, *J* = 18.0, 6.2 Hz, 1H), 3.70 (s, 3H), 3.51 (s, 3H), 3.30 (d, *J* = 17.8 Hz, 1H), 3.02 (dd, *J* = 18.0, 3.0 Hz, 1H), 2.74 (d, *J* = 17.8 Hz, 1H), 2.35 (s, 1H), 2.18 (s, 2H).; $^{13}C$ NMR (100 MHz, $CDCl_3$) δ 198.66, 175.09, 145.88, 137.81, 136.82, 136.25, 133.76, 133.52, 129.04, 128.83, 128.42, 126.30, 126.18, 124.15, 121.77, 121.18, 112.52, 109.29, 61.74, 53.76, 52.43, 41.10,

39.83, 37.41, 29.45, 25.10; HRMS (ESI) *m/z* calcd for $C_{30}H_{28}N_2O_3$ $[M+H]^+$ = 465.2173, found = 465.2174; The ee value was >99%, $t_R$ (major) = 11.4 min, $t_R$ (minor) = 17.9 min (Chiralcel IA, λ = 254 nm, 10% i-PrOH/hexane, flow rate = 1.0 mL/min).

<Chromatogram>

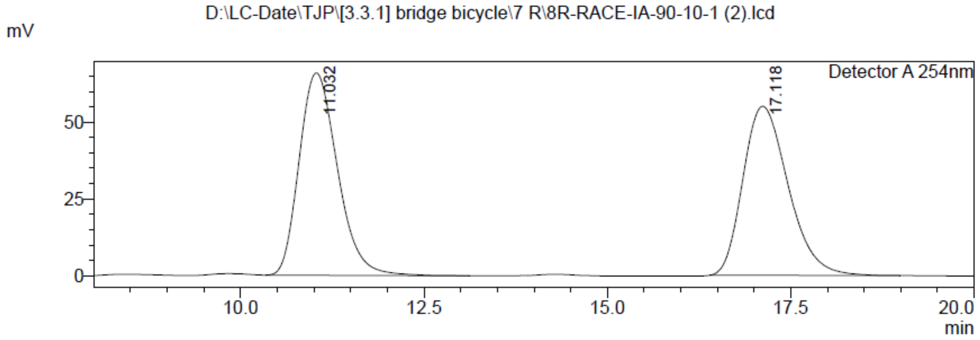


<Peak Table>

Detector A 254nm

| Peak# | Ret. Time | Height | Height% | Conc. | Area | Area% |
|---|---|---|---|---|---|---|
| 1 | 11.032 | 65910 | 54.469 | 50.287 | 2393672 | 50.287 |
| 2 | 17.118 | 55095 | 45.531 | 49.713 | 2366386 | 49.713 |
| Total | | 121006 | 100.000 | | 4760058 | 100.000 |

Racemic **4a**

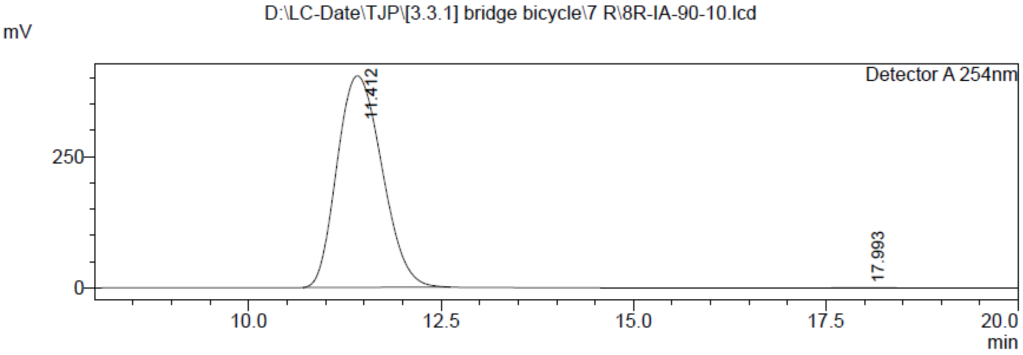


<Peak Table>

Detector A 254nm

| Peak# | Ret. Time | Height | Height% | Conc. | Area | Area% |
|---|---|---|---|---|---|---|
| 1 | 11.412 | 402867 | 99.837 | 99.807 | 16371201 | 99.807 |
| 2 | 17.993 | 659 | 0.163 | 0.193 | 31715 | 0.193 |
| Total | | 403526 | 100.000 | | 16402917 | 100.000 |

Enantiomerically enriched **4a**

**Methyl(*6S,7S,13R*)-7-(2-(4-fluorophenyl)-2-oxoethyl)-8,13-dimethyl-5,7,8,13-tetrahydro-6H-6,13-epiminobenzo[4,5]cycloocta[1,2-b]indole-6-carboxylate(4b)**

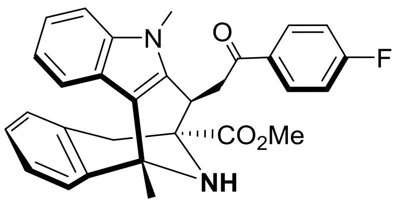

**4b**

A white solid; 45.8 mg, 95% yield; m.p. =128.3-129.2 °C, $[\alpha]^{25}_{D}$ = -60.65 (*c* 0.31, $CHCl_3$); *dr* >20:1; $^1$H NMR (400 MHz, $CDCl_3$) δ 8.02 (dd, *J* = 11.0, 5.4 Hz, 2H), 7.68 (d, *J* = 6.4 Hz, 1H), 7.62 (d, *J* = 8.0 Hz, 1H), 7.20 (t, *J* = 8.2 Hz, 2H), 7.14 – 7.10 (m, 3H), 7.05 (td, *J* = 7.6, 0.8 Hz, 2H), 6.94 (d, *J* = 7.6 Hz, 1H), 4.19 (dd, *J* = 6.4, 3.2 Hz, 1H), 3.90 (dd, *J* = 18.0, 6.4 Hz, 1H), 3.67 (s, 3H), 3.51 (s, 3H), 3.27 (d, *J* = 18.0 Hz, 1H), 3.00 (dd, *J* = 18.0, 3.2 Hz, 1H), 2.71 (d, *J* = 18.0 Hz, 1H), 2.15 (s, 3H); $^{13}$C NMR (100 MHz, $CDCl_3$) δ 197.12, 175.06, 145.84, 136.93 (d, *J* = 178.8 Hz), 133.71, 133.28 (d, *J* = 3.0 Hz), 131.15, 131.06, 129.05, 126.28 (d, *J* = 11.2 Hz), 124.13, 121.78, 121.25, 119.37, 119.32, 115.93 (d, *J* = 21.8 Hz), 112.54, 109.30. 61.73, 53.79, 52.46, 41.02, 39.90, 37.37, 29.46, 25.09; $^{19}$F NMR (376 MHz, CDCl3) δ -104.68. HRMS (ESI) *m/z* calcd for $C_{30}H_{27}FN_2O_3$ $[M+H]^+$ = 483.2078, found = 483.2079; The ee value was 85%, $t_R$ (major) = 11.3 min, $t_R$ (minor) = 18.0 min (Chiralcel IA, λ = 254 nm, 10% i-PrOH/hexane, flow rate = 1.0 mL/min).

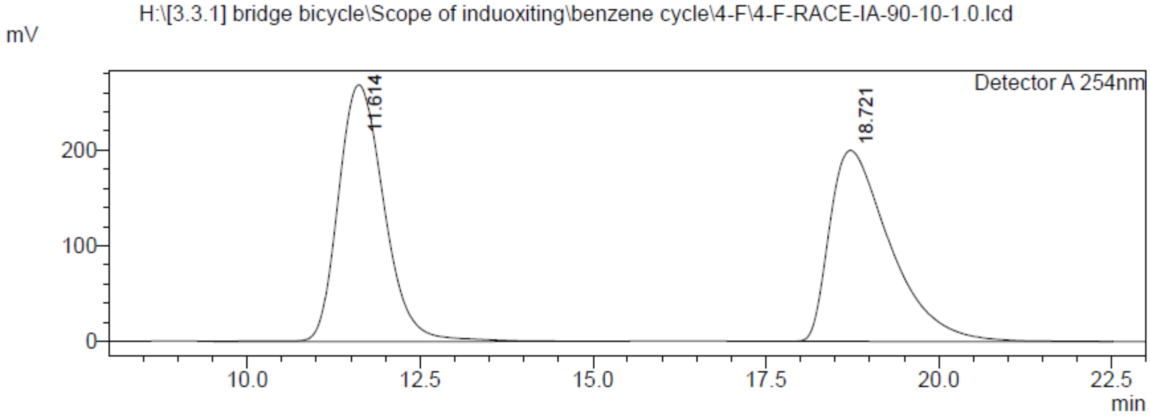


Detector A 254nm

| Peak# | Ret. Time | Height | Height% | Area | Area% |
|---|---|---|---|---|---|
| 1 | 11.614 | 268454 | 57.323 | 12361936 | 50.291 |
| 2 | 18.721 | 199862 | 42.677 | 12219012 | 49.709 |
| Total | | 468316 | 100.000 | 24580947 | 100.000 |

Racemic **4b**

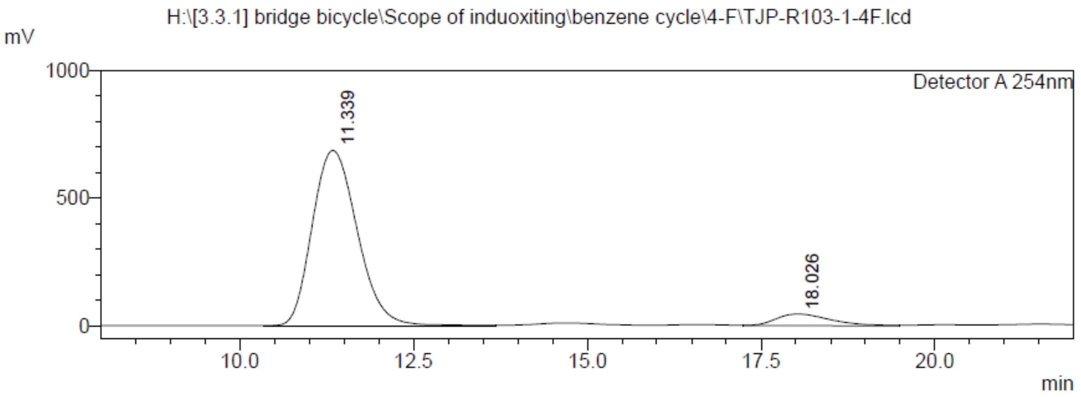


Detector A 254nm

| Peak# | Ret. Time | Height | Height% | Area | Area% |
|---|---|---|---|---|---|
| 1 | 11.339 | 685826 | 93.718 | 30912259 | 92.561 |
| 2 | 18.026 | 45970 | 6.282 | 2484415 | 7.439 |
| Total | | 731796 | 100.000 | 33396674 | 100.000 |

Enantiomerically enriched **4b**

**Methyl(6S,7S,13R)-7-(2-(4-chlorophenyl)-2-oxoethyl)-8,13-dimethyl-5,7,8,13-tetrahydro-6H-6,13-epiminobenzo[4,5]cycloocta[1,2-b]indole-6-carboxylate (4c)**

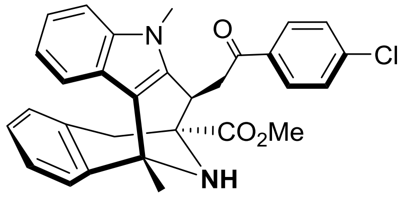


**4c**

A white solid; 47.3 mg, 95% yield; m.p. = 88.5-89.7 °C, $[\alpha]^{25}_{D}$ = -40.15 (*c* 0.18, $CHCl_3$); *dr* >20:1; $^1$H NMR (400 MHz, $CDCl_3$) δ 7.93 (d, *J* = 8.4 Hz, 2H), 7.68 (d, *J* = 7.6 Hz, 1H), 7.62 (d, *J* = 8.0 Hz, 1H), 7.42 (d, *J* = 8.8 Hz, 2H), 7.20 (t, *J* = 8.4 Hz, 2H), 7.12 (t, *J* = 8.0 Hz, 1H), 7.05 (q, *J* = 7.2 Hz, 2H), 6.93 (d, *J* = 7.6 Hz, 1H), 4.18 (dd, *J* = 6.0, 3.2 Hz, 1H), 3.89 (dd, *J* = 18.0, 6.0 Hz, 1H), 3.66 (s, 3H), 3.51 (s, 3H), 3.26 (d, *J* = 18.0 Hz, 1H), 2.99 (dd, *J* = 18.0, 3.2 Hz, 1H), 2.70 (d, *J* = 18.0 Hz, 1H), 2.15 (s, 3H); $^{13}$C NMR (100 MHz, $CDCl_3$) δ 197.52, 175.04, 145.82, 140.04, 137.83, 135.98, 135.15, 133.69, 129.84, 129.15, 129.05, 126.34, 126.23, 124.13, 121.79, 121.27, 119.38, 119.33, 112.56, 109.30, 61.74, 53.79, 52.49, 41.08, 39.86, 37.36, 29.46, 25.09; HRMS (ESI) *m/z* calcd for $C_{30}H_{27}ClN_2O_3$ $[M+H]^+$ = 499.1783, found = 499.1786; The ee value was 92%, $t_R$ (major) = 11.9 min, $t_R$ (minor) = 16.7 min (Chiralcel IA, λ = 254 nm, 10% i-PrOH/hexane, flow rate = 1.0 mL/min).

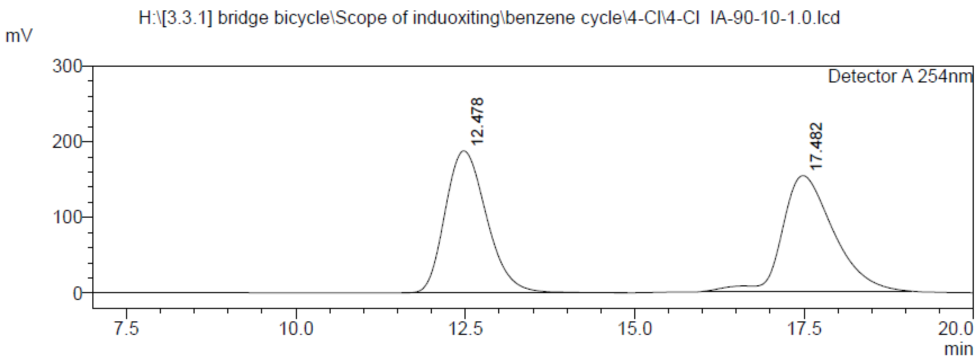


Detector A 254nm

| Peak# | Ret. Time | Height | Height% | Area | Area% |
|---|---|---|---|---|---|
| 1 | 12.478 | 187461 | 55.132 | 7991607 | 49.933 |
| 2 | 17.482 | 152560 | 44.868 | 8012938 | 50.067 |
| Total | | 340021 | 100.000 | 16004545 | 100.000 |

Racemic **4c**

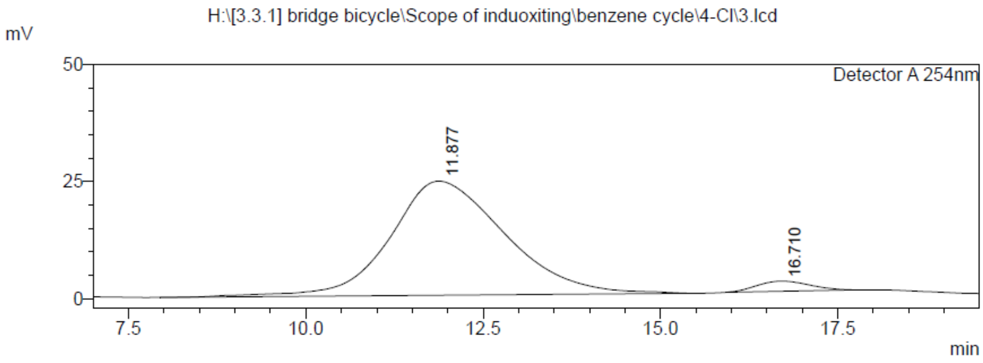


Detector A 254nm

| Peak# | Ret. Time | Height | Height% | Area | Area% |
|---|---|---|---|---|---|
| 1 | 11.877 | 24341 | 91.890 | 2760369 | 96.107 |
| 2 | 16.710 | 2148 | 8.110 | 111807 | 3.893 |
| Total | | 26489 | 100.000 | 2872176 | 100.000 |

Enantiomerically enriched **4c**

**Methyl(6S,7S,13R)-7-(2-(4-bromophenyl)-2-oxoethyl)-8,13-dimethyl-5,7,8,13-tetrahydro-6H-6,13-epiminobenzo[4,5]cycloocta[1,2-b]indole-6-carboxylate (4d)**

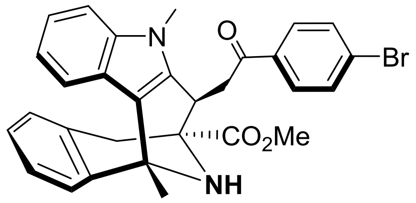


**4d**

A white solid; 50.9 mg, 94% yield; m.p. = 170.2-170.9 °C, $[\alpha]^{25}_{D}$ = -37.64 (*c* 0.34, $CHCl_3$); *dr* >20:1; $^1$H NMR (400 MHz, $CDCl_3$) δ 7.86 (d, *J* = 8.8 Hz, 2H), 7.69 (d, *J*

= 7.6 Hz, 1H), 7.63 (d, $J$ = 8.0 Hz, 1H), 7.60 (d, $J$ = 8.8 Hz, 2H), 7.20 (t, $J$ = 8.2 Hz, 2H), 7.15 – 7.11 (m, 1H), 7.05 (td, $J$ = 7.4, 1.0 Hz, 2H), 6.94 (d, $J$ = 7.6 Hz, 1H), 4.19 (dd, $J$ = 6.4, 3.0 Hz, 1H), 3.90 (dd, $J$ = 18.0, 6.4 Hz, 1H), 3.67 (s, 3H), 3.52 (s, 3H), 3.27 (d, $J$ = 18.0 Hz, 1H), 2.99 (dd, $J$ = 18.0, 3.0 Hz, 1H), 2.71 (d, $J$ = 18.0 Hz, 1H), 2.31 (s, 1H), 2.16 (s, 3H); $^{13}$C NMR (100 MHz, $CDCl_3$) δ 197.71, 175.02, 145.81, 137.82, 135.95, 135.54, 133.67, 132.13, 129.93, 129.04, 128.78, 126.34, 126.23,, 124.12, 121.78, 121.27, 119.38, 119.32, 112.56, 109.30, 61.73, 53.79, 52.48, 41.06, 39.84, 37.35, 29.45, 25.08; HRMS (ESI) *m/z* calcd for $C_{30}H_{27}BrN_2O_3$ $[M+H]^+$ = 543.1278, found = 543.1279; The ee value was 94%, $t_R$ (major) = 13.4 min, $t_R$ (minor) = 16.8 min (Chiralcel IA, λ = 254 nm, 10% i-PrOH/hexane, flow rate = 1.0 mL/min).

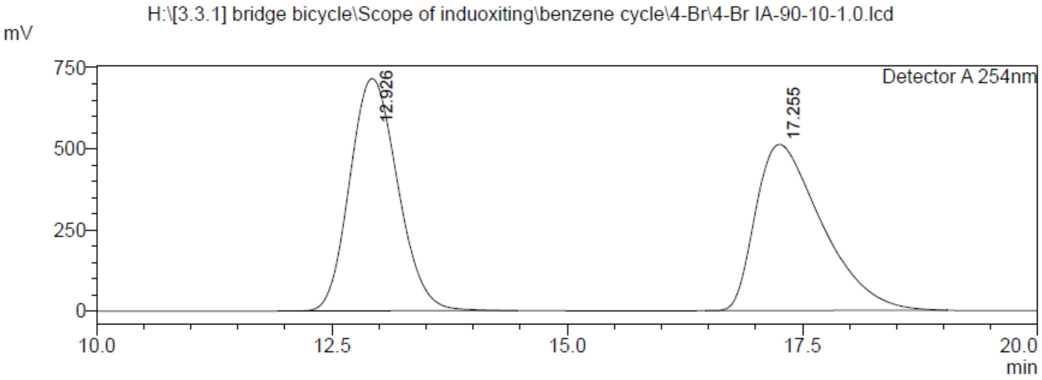


Detector A 254nm

| Peak# | Ret. Time | Height | Height% | Area | Area% |
|---|---|---|---|---|---|
| 1 | 12.926 | 715446 | 58.304 | 25346918 | 50.219 |
| 2 | 17.255 | 511661 | 41.696 | 25125596 | 49.781 |
| Total | | 1227107 | 100.000 | 50472514 | 100.000 |

Racemic **4d**

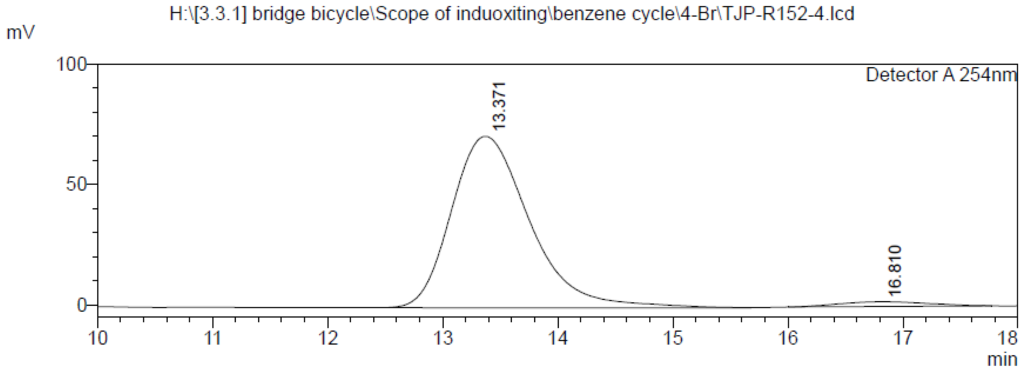


Detector A 254nm

| Peak# | Ret. Time | Height | Height% | Area | Area% |
|---|---|---|---|---|---|
| 1 | 13.371 | 71167 | 97.385 | 3367511 | 96.932 |
| 2 | 16.810 | 1911 | 2.615 | 106590 | 3.068 |
| Total | | 73078 | 100.000 | 3474101 | 100.000 |

Enantiomerically enriched **4d**

**Methyl(6S,7S,13R)-8,13-dimethyl-7-(2-oxo-2-(p-tolyl)ethyl)-5,7,8,13-tetrahydro-**

**6H-6,13-epiminobenzo[4,5]cycloocta[1,2-b]indole-6-carboxylate (4e)**

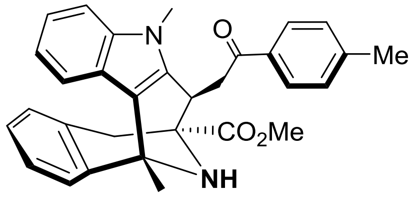


**4e**

A white solid; 43.0 mg, 90% yield; m.p. = 180.2-181.6 °C, $[\alpha]^{25}_{D}$ = -64.24 (*c* 0.25, $CHCl_3$); *dr* >20:1; $^1$H NMR (400 MHz, $CDCl_3$) δ 7.90 (d, *J* = 8.4 Hz, 2H), 7.68 (d, *J* = 6.8 Hz, 1H), 7.63 (d, *J* = 7.6 Hz, 1H), 7.25 (d, *J* = 7.2 Hz, 2H), 7.20 (dd, *J* = 12.2, 7.8 Hz, 2H), 7.14 – 7.10 (m, 1H), 7.07 – 7.03 (m, 2H), 6.93 (d, *J* = 7.8 Hz, 1H), 4.21 (dd, *J* = 6.2, 3.0 Hz, 1H), 3.92 (dd, *J* = 18.0, 6.2 Hz, 1H), 3.68 (s, 3H), 3.48 (s, 3H), 3.27 (d, *J* = 18.0 Hz, 1H), 2.96 (dd, *J* = 18.0, 3.0 Hz, 1H), 2.72 (d, *J* = 18.0 Hz, 1H), 2.40 (s, 3H), 2.16 (s, 3H); $^{13}$C NMR (100 MHz, $CDCl_3$) δ 198.25, 175.11, 145.90, 144.41, 137.81, 136.36, 134.36, 133.79, 129.52, 129.03, 128.56, 126.28, 126.16, 124.15, 121.77, 121.14, 119.28, 112.49, 109.28, 61.73, 53.76, 52.41, 40.93, 39.89, 37.43, 29.46, 25.13, 21.79; HRMS (ESI) *m/z* calcd for $C_{31}H_{30}N_2O_3$ $[M+H]^+$ = 479.2329, found = 479.2329; The ee value was 94%, $t_R$ (major) = 12.1 min, $t_R$ (minor) = 16.5 min (Chiralcel IA, λ = 254 nm, 10% i-PrOH/hexane, flow rate = 1.0 mL/min).

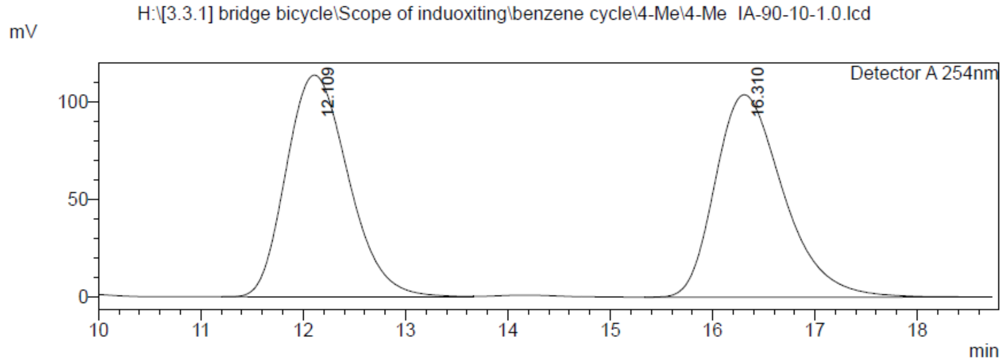


Detector A 254nm

| Peak# | Ret. Time | Height | Height% | Area | Area% |
|---|---|---|---|---|---|
| 1 | 12.109 | 113558 | 52.282 | 4810102 | 49.983 |
| 2 | 16.310 | 103645 | 47.718 | 4813316 | 50.017 |
| Total | | 217203 | 100.000 | 9623418 | 100.000 |

Racemic **4e**

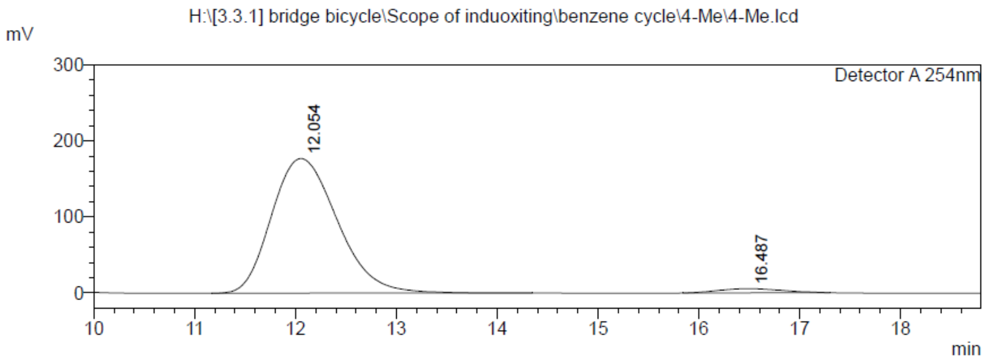


Detector A 254nm

| Peak# | Ret. Time | Height | Height% | Area | Area% |
|---|---|---|---|---|---|
| 1 | 12.054 | 176988 | 96.999 | 8036270 | 97.113 |
| 2 | 16.487 | 5475 | 3.001 | 238862 | 2.887 |
| Total | | 182463 | 100.000 | 8275132 | 100.000 |

Enantiomerically enriched **4e**

**Methyl(6S,7S,13R)-7-(2-(4-methoxyphenyl)-2-oxoethyl)-8,13-dimethyl-5,7,8,13-tetrahydro-6H-6,13-epiminobenzo[4,5]cycloocta[1,2-b]indole-6-carboxylate (4f)**

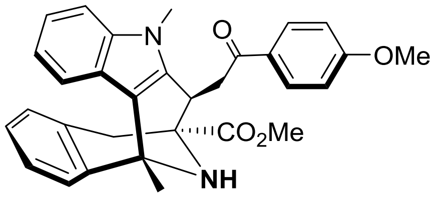


**4f**

A white solid; 44.9 mg, 91% yield; m.p. = 150.4-151.2 °C, $[\alpha]^{25}_{D}$ = -68.56 (*c* 0.28, $CHCl_3$); *dr* >20:1; $^1H$ NMR (400 MHz, $CDCl_3$) δ 8.00 (d, *J* = 8.8 Hz, 2H), 7.69 (d, *J* = 7.4 Hz, 1H), 7.63 (d, *J* = 8.0 Hz, 1H), 7.20 (t, *J* = 9.4 Hz, 2H), 7.12 (t, *J* = 7.2 Hz, 1H), 7.06 (d, *J* = 6.4 Hz, 2H), 7.03 (d, *J* = 6.8 Hz, 2H), 6.95 – 6.92 (m, 3H), 4.21 (dd, *J* =5.8, 3.0 Hz, 1H), 3.92 (dd, *J* = 17.8, 5.8 Hz, 1H), 3.86 (s, 3H), 3.68 (s, 1H), 3.48 (s, 1H), 3.28 (d, *J* = 17.8 Hz, 1H), 2.94 (dd, *J* = 17.8, 3.0 Hz, 1H), 2.73 (d, *J* = 17.8 Hz, 1H), 2.18 (s, 3H).; $^{13}C$ NMR (100 MHz, $CDCl_3$) δ 197.16, 175.11, 163.86, 145.89, 137.80, 136.35, 133.79, 130.76, 129.92, 129.02, 126.26, 126.14, 124.14, 121.75, 121.11, 119.26, 113.95, 112.46, 109.27, 61.68, 55.62, 53.75, 52.38, 40.61, 40.07, 37.44, 29.44, 25.12; HRMS (ESI) *m/z* calcd for $C_{31}H_{30}N_2O_4$ $[M+H]^+$ = 495.2278, found = 495.2275; The ee value was 96%, $t_R$ (major) = 16.7 min, $t_R$ (minor) = 21.8 min (Chiralcel IA, λ = 254 nm, 10% i-PrOH/hexane, flow rate = 1.0 mL/min).

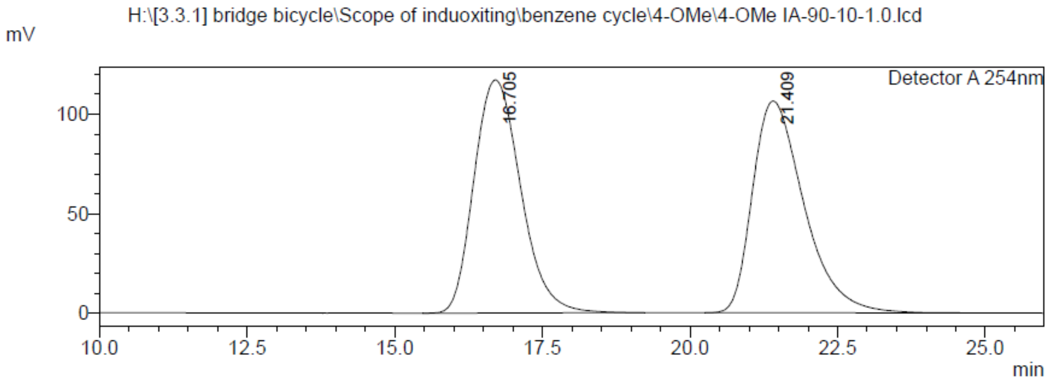


Detector A 254nm

| Peak# | Ret. Time | Height | Height% | Area | Area% |
|---|---|---|---|---|---|
| 1 | 16.705 | 117036 | 52.404 | 6447995 | 49.989 |
| 2 | 21.409 | 106298 | 47.596 | 6450768 | 50.011 |
| Total | | 223334 | 100.000 | 12898764 | 100.000 |

Racemic **4f**

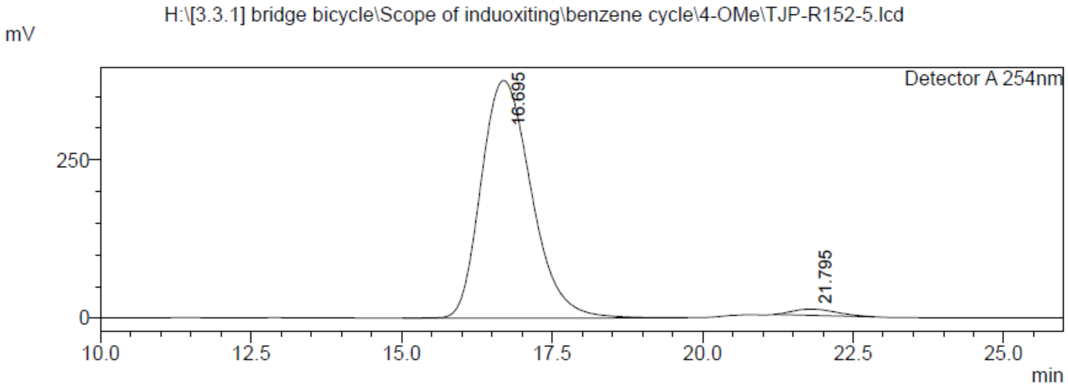


Detector A 254nm

| Peak# | Ret. Time | Height | Height% | Area | Area% |
|---|---|---|---|---|---|
| 1 | 16.695 | 375455 | 97.450 | 21865062 | 97.793 |
| 2 | 21.795 | 9824 | 2.550 | 493551 | 2.207 |
| Total | | 385280 | 100.000 | 22358613 | 100.000 |

Enantiomerically enriched **4f**

**Methyl(6S,7S,13R)-7-(2-(3-chlorophenyl)-2-oxoethyl)-8,13-dimethyl-5,7,8,13-tetrahydro-6H-6,13-epiminobenzo[4,5]cycloocta[1,2-b]indole-6-carboxylate (4g)**

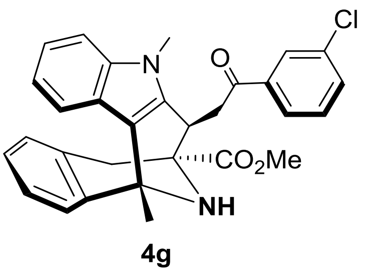


**4g**

A white solid; 45.3 mg, 91% yield; m.p. = 130.6-131.4 °C, $[\alpha]^{25}_{D}$ = -30.68 (*c* 0.38, $CHCl_3$); *dr* >20:1; $^1$H NMR (400 MHz, $CDCl_3$) δ 7.96 (t, *J* = 1.8 Hz, 1H), 7.84 (d, *J* = 7.8 Hz, 1H), 7.68 (d, *J* = 7.4 Hz, 1H), 7.62 (d, *J* = 8.0 Hz, 1H), 7.52 (ddd, *J* = 8.0, 2.0, 1.0 Hz, 1H), 7.38 (t, *J* = 8.0 Hz, 2H), 7.19 (t, *J* = 7.2 Hz, 2H), 7.16 – 7.10 (m, 1H), 7.04 (td, *J* = 8.2, 1.0 Hz, 2H), 6.94 (d, *J* = 7.4 Hz, 1H), 4.18 (dd, *J* = 6.0, 3.4 Hz, 1H), 3.86 (dd, *J* = 17.9, 6.0 Hz, 1H), 3.65 (s, 3H), 3.56 (s, 3H), 3.28 (d, *J* = 17.8 Hz, 1H), 3.05 (dd, *J* = 17.9, 3.4 Hz, 1H), 2.70 (d, *J* = 17.8 Hz, 1H), 2.15 (s, 3H). $^{13}$C NMR (100 MHz, $CDCl_3$) δ 197.41, 175.00, 145.81, 138.35, 137.83, 135.86, 135.20, 133.67, 133.39, 130.13, 129.05, 128.40, 126.52, 126.34, 126.23, 124.13, 121.78, 121.28, 119.38, 119.33, 112.58, 109.30, 61.76, 53.81, 52.55, 41.38, 39.70, 37.35, 29.48, 25.05; HRMS (ESI) *m/z* calcd for $C_{30}H_{27}ClN_2O_3$ $[M+H]^+$ = 499.1783, found = 499.1787; The ee value was 92%, $t_R$ (major) = 9.8 min, $t_R$ (minor) = 14.3 min (Chiralcel IA, λ = 254 nm, 10% i-PrOH/hexane, flow rate = 1.0 mL/min).

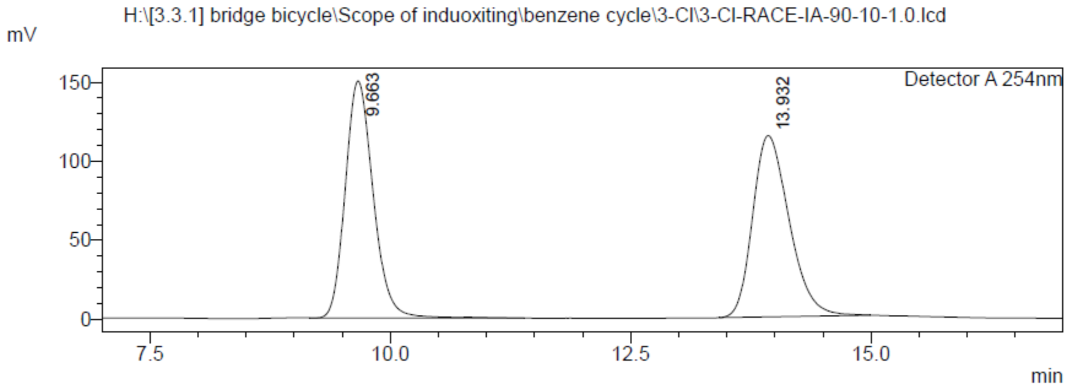


Detector A 254nm

| Peak# | Ret. Time | Height | Height% | Area | Area% |
|---|---|---|---|---|---|
| 1 | 9.663 | 149851 | 56.649 | 3041759 | 50.090 |
| 2 | 13.932 | 114674 | 43.351 | 3030847 | 49.910 |
| Total | | 264526 | 100.000 | 6072606 | 100.000 |

Racemic **4g**

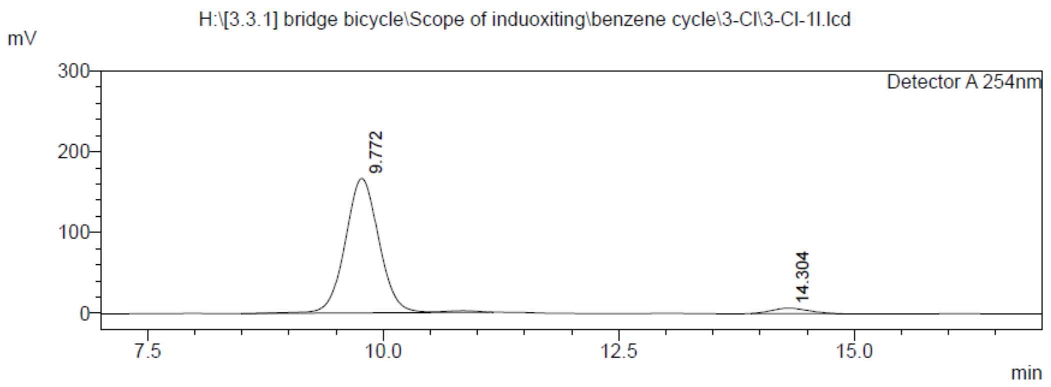


Detector A 254nm

| Peak# | Ret. Time | Height | Height% | Area | Area% |
|---|---|---|---|---|---|
| 1 | 9.772 | 166316 | 96.187 | 4194578 | 95.937 |
| 2 | 14.304 | 6593 | 3.813 | 177643 | 4.063 |
| Total | | 172909 | 100.000 | 4372220 | 100.000 |

Enantiomerically enriched **4g**

**Methyl(6S,7S,13R)-8,13-dimethyl-7-(2-oxo-2-(m-tolyl)ethyl)-5,7,8,13-tetrahydro-6H-6,13-epiminobenzo[4,5]cycloocta[1,2-b]indole-6-carboxylate (4h)**

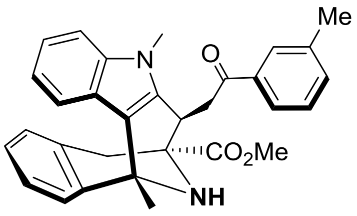


**4h**

A white solid; 40.6 mg, 85% yield; m.p. = 161.2-161.9 $^{o}$C, $[\alpha]^{25}_{D}$ = -24.54 (*c* 0.32, $CHCl_3$); *dr* >20:1; $^{1}$H NMR (400 MHz, $CDCl_3$) δ 7.80 (d, *J* = 9.2 Hz, 2H), 7.70 (d, *J* = 6.8 Hz, 1H), 7.64 (d, *J* = 8.0 Hz, 1H), 7.37 – 7.32 (m, 2H), 7.20 (t, *J* = 7.8 Hz, 2H), 7.15– 7.11 (m, 1H), 7.06 (td, *J* = 7.8, 1.2 Hz, 2H), 6.95 (d, *J* = 7.2 Hz, 1H), 4.23 (dd, *J* = 6.0, 3.2 Hz, 1H), 3.89 (dd, *J* = 18.0, 6.0 Hz, 1H), 3.67 (s, 3H), 3.54 (s, 3H), 3.30 (d, *J* = 18.0 Hz, 1H), 3.06 (dd, *J* = 18.0, 3.2 Hz, 1H), 2.74 (d, *J* = 18.0 Hz, 1H), 2.39 (s, 3H), 2.35 (s, 1H), 2.17 (s, 3H); $^{13}$C NMR (100 MHz, $CDCl_3$) δ 198.72, 175.09, 145.88, 138.61, 137.80, 136.83, 136.31, 134.26, 133.78, 129.04, 128.91, 128.68, 126.29, 126.16, 125.59, 124.15, 121.75, 121.16, 119.29, 112.46, 109.28, 61.77, 53.77, 52.47 , 41.33, 39.68, 37.42, 29.46, 25.09, 21.44; HRMS (ESI) *m/z* calcd for $C_{31}H_{30}N_2O_3$ $[M+H]^+$ = 479.2329, found = 479.2332; The ee value was 85%, $t_R$ (major) = 9.8 min, $t_R$ (minor) = 14.3 min (Chiralcel IA, λ = 254 nm, 10% i-PrOH/hexane, flow rate = 1.0 mL/min).

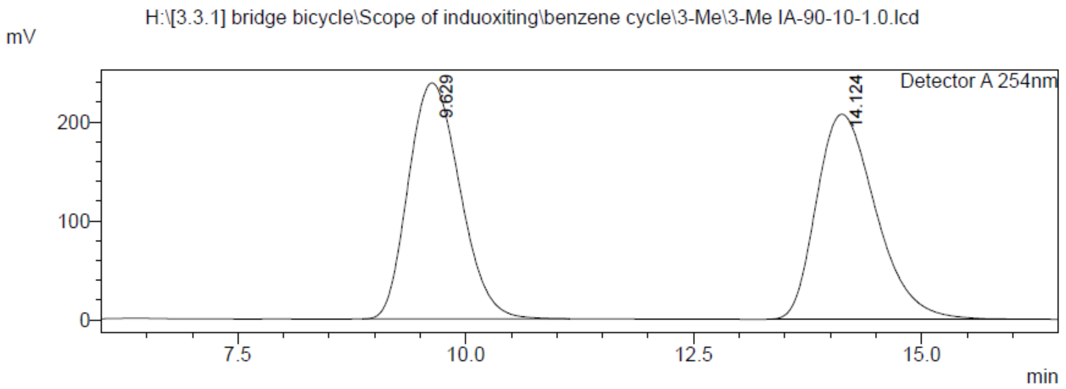


Detector A 254nm

| Peak# | Ret. Time | Height | Height% | Area | Area% |
|---|---|---|---|---|---|
| 1 | 9.629 | 238495 | 53.486 | 9375914 | 49.902 |
| 2 | 14.124 | 207404 | 46.514 | 9412690 | 50.098 |
| Total | | 445899 | 100.000 | 18788604 | 100.000 |

Racemic **4h**

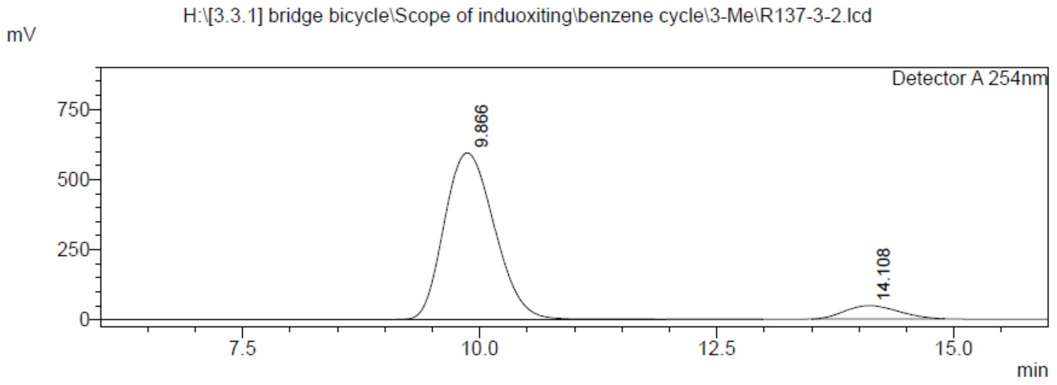


Detector A 254nm

| Peak# | Ret. Time | Height | Height% | Area | Area% |
|---|---|---|---|---|---|
| 1 | 9.866 | 594340 | 92.656 | 21965610 | 92.259 |
| 2 | 14.108 | 47109 | 7.344 | 1843087 | 7.741 |
| Total | | 641449 | 100.000 | 23808697 | 100.000 |

Enantiomerically enriched **4h**

**Methyl(6S,7S,13R)-7-(2-(3-methoxyphenyl)-2-oxoethyl)-8,13-dimethyl-5,7,8,13-tetrahydro-6H-6,13-epiminobenzo[4,5]cycloocta[1,2-b]indole-6-carboxylate(4i)**

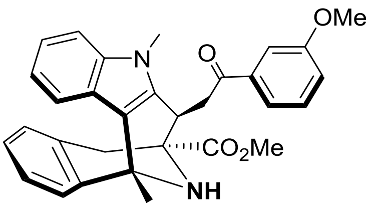


**4i**

A white solid; 42.9 mg, 87% yield; m.p. = 140.3-141.2 $^{o}$C, $[\alpha]^{25}_{D}$ = -31.25 (*c* 0.22, $CHCl_3$); *dr* >20:1; $^1$H NMR (400 MHz, $CDCl_3$) δ 7.69 (d, *J* = 7.6 Hz, 1H), 7.63 (d, *J*

= 8.0 Hz, 1H), 7.56 (d, $J$ = 8.0 Hz, 1H), 7.52 (d, $J$ = 1.8 Hz, 1H), 7.36 (t, $J$ = 8.0 Hz, 1H), 7.22 – 7.18 (m, 2H), 7.15 – 7.10 (m, 2H), 7.05 (q, $J$ = 6.8 Hz, 2H), 6.94 (d, $J$ = 7.4 Hz, 1H), 4.21 (dd, $J$ = 6.0, 3.2 Hz, 1H), 3.91 (dd, $J$ = 18.0, 6.0 Hz, 1H), 3.85 (s, 3H), 3.67 (s, 3H), 3.52 (s, 3H), 3.28 (d, $J$ = 18.0 Hz, 1H), 3.01 (dd, $J$ = 18.0, 3.2 Hz, 1H), 2.72 (d, $J$ = 18.0 Hz, 1H), 2.16 (s, 3H); $^{13}$C NMR (100 MHz, $CDCl_3$) δ 198.53 , 175.06, 160.02, 145.88, 138.22, 137.81, 136.24, 133.76, 129.84, 129.04, 126.30, 126.18, 124.15, 121.77, 121.19, 121.13, 120.03, 119.32, 119.30, 112.54, 112.51, 109.29, 61.75, 55.60, 53.77, 52.47, 41.32, 39.90, 37.42, 29.47, 25.09; HRMS (ESI) *m/z* calcd for $C_{31}H_{30}N_2O_4$ $[M+H]^+$ = 495.2278, found = 495.2277; The ee value was 84%, $t_R$ (major) = 12.8 min, $t_R$ (minor) = 16.0 min (Chiralcel IA, λ = 254 nm, 10% i-PrOH/hexane, flow rate = 1.0 mL/min).

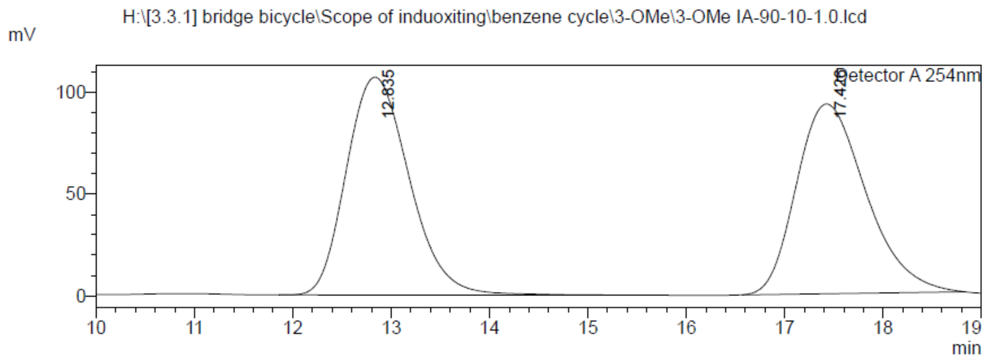


Detector A 254nm

| Peak# | Ret. Time | Height | Height% | Area | Area% |
|---|---|---|---|---|---|
| 1 | 12.835 | 107225 | 53.432 | 4779406 | 50.946 |
| 2 | 17.426 | 93450 | 46.568 | 4601841 | 49.054 |
| Total | | 200675 | 100.000 | 9381247 | 100.000 |

Racemic **4i**

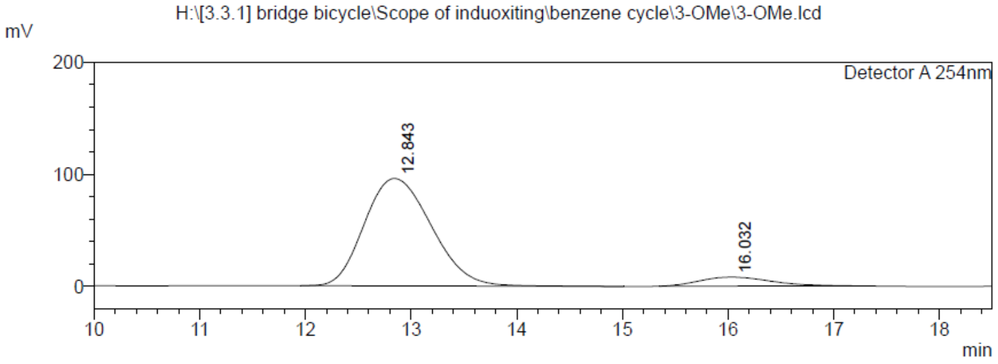


Detector A 254nm

| Peak# | Ret. Time | Height | Height% | Area | Area% |
|---|---|---|---|---|---|
| 1 | 12.843 | 95787 | 92.248 | 4221881 | 91.782 |
| 2 | 16.032 | 8049 | 7.752 | 378019 | 8.218 |
| Total | | 103836 | 100.000 | 4599901 | 100.000 |

Enantiomerically enriched **4i**

**Methyl(6S,7S,13R)-7-(2-(2-bromophenyl)-2-oxoethyl)-8,13-dimethyl-5,7,8,13-tetrahydro-6H-6,13-epiminobenzo[4,5]cycloocta[1,2-b]indole-6-carboxylate(4j)**

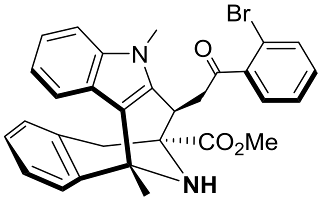


**4j**

A white solid; 47.1 mg, 87% yield; m.p. = 67.5-68.8 $^{o}$C, $[\alpha]^{25}_{D}$ = -11.23 (*c* 0.26, $CHCl_3$); *dr* >20:1; $^{1}$H NMR (400 MHz, $CDCl_3$) δ 7.68 (d, *J* = 7.2 Hz, 1H), 7.64 – 7.60 (m, 2H), 7.44 (dd, *J* = 7.6, 1.8 Hz, 1H), 7.36 (td, *J* = 7.2, 1.0 Hz, 1H), 7.30 (dd, *J* = 8.0, 2.0 Hz, 1H), 7.23 (d, 7.6 Hz, 1H), 7.20 (d, 7.6 Hz, 1H)，7.17 – 7.13 (m, 1H), 7.06 (td, *J* = 8.0, 1.0 Hz, 2H), 6.96 (d, *J* = 7.2 Hz, 1H), 4.24 (dd, *J* = 5.6, 3.2 Hz, 1H), 3.76 (s, 3H), 3.74 (dd, *J* =19.6, 5.6 Hz, 1H), 3.72 (s, 3H), 3.34 (d, *J* = 18.0 Hz, 1H), 3.21 (dd, *J* = 19.6, 3.2 Hz, 1H), 2.75 (d, *J* = 18.0 Hz, 1H), 2.12 (s, 3H); $^{13}$C NMR (100 MHz, $CDCl_3$) δ 201.29 , 175.20, 145.82, 140.94, 137.80, 136.21, 134.03, 133.75, 131.92, 129.04, 128.85, 127.58, 126.31, 126.19, 124.14, 121.71, 121.21, 119.33, 119.30, 118.89, 112.31, 109.33, 61.87, 53.76, 52.78, 46.00, 38.49, 37.33, 29.59, 25.01; HRMS (ESI) *m/z* calcd for $C_{30}H_{27}BrN_2O_3$ $[M+H]^+$ = 543.1278, found = 543.1276; The ee value was 96%, $t_R$ (major) = 11.1 min, $t_R$ (minor) = 20.1 min (Chiralcel IA, λ = 254 nm, 10% i-PrOH/hexane, flow rate = 1.0 mL/min).

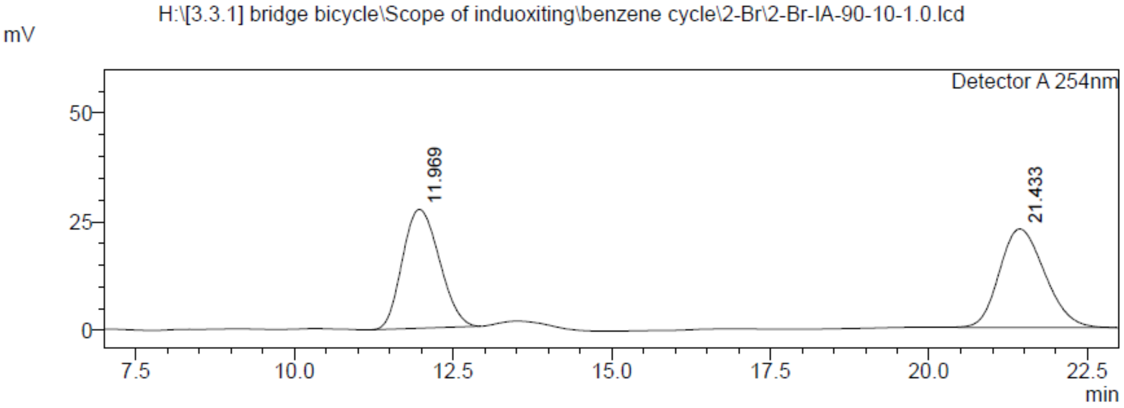


Detector A 254nm

| Peak# | Ret. Time | Height | Height% | Area | Area% |
|---|---|---|---|---|---|
| 1 | 11.969 | 27321 | 54.659 | 1111115 | 49.573 |
| 2 | 21.433 | 22663 | 45.341 | 1130235 | 50.427 |
| Total | | 49984 | 100.000 | 2241350 | 100.000 |

Racemic **4j**

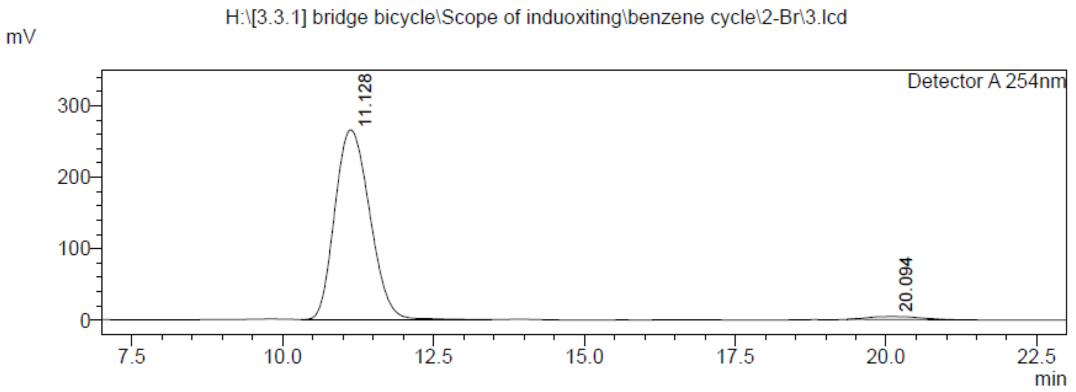


Detector A 254nm

| Peak# | Ret. Time | Height | Height% | Area | Area% |
|---|---|---|---|---|---|
| 1 | 11.128 | 265175 | 98.392 | 10684462 | 97.735 |
| 2 | 20.094 | 4333 | 1.608 | 247623 | 2.265 |
| Total | | 269509 | 100.000 | 10932085 | 100.000 |

Enantiomerically enriched **4j**

**Methyl(6S,7S,13R)-8,13-dimethyl-7-(2-oxo-2-(o-tolyl)ethyl)-5,7,8,13-tetrahydro-6H-6,13-epiminobenzo[4,5]cycloocta[1,2-b]indole-6-carboxylate(4k)**

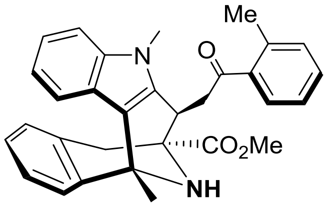


**4k**

A white solid; 41.1 mg, 86% yield; m.p. = 152.1-153.5 °C, $[\alpha]^{25}_D$ = -15.40 (*c* 0.24, $CHCl_3$); *dr* >20:1; $^1$H NMR (400 MHz, $CDCl_3$) δ 7.68 (d, *J* = 7.0 Hz, 1H), 7.63 (t, *J* = 7.6 Hz, 2H), 7.37 (td, *J* = 7.6, 1.2 Hz, 1H), 7.26 – 7.23 (m, 3H), 7.23 – 7.17 (m, 2H), 7.14 (dd, *J* = 7.0, 1.2 Hz, 1H), 7.09 – 7.01 (m, 2H), 6.94 (d, *J* = 7.6 Hz, 1H), 4.24 (dd, *J* = 5.8, 3.0 Hz, 1H), 3.84 (dd, *J* = 18.6, 5.8 Hz, 1H), 3.70 (s, 3H), 3.59 (s, 3H), 3.29 (d, *J* = 17.8 Hz, 1H), 3.00 (dd, *J* = 18.6, 3.0 Hz, 1H), 2.74 (d, *J* = 17.8 Hz, 1H), 2.59 (s, 3H), 2.13 (s, 3H).;$^{13}$C NMR (100 MHz, $CDCl_3$) δ 201.76, 175.24, 145.88, 138.61, 137.81, 137.33, 136.54, 133.80, 132.19, 131.84, 129.20, 129.04, 126.29, 126.17, 125.99, 124.18, 121.76, 121.16, 119.31, 119.30, 112.40, 109.29, 61.90, 53.76, 52.50, 44.29, 39.41, 37.50, 29.49, 25.09, 21.72; HRMS (ESI) *m/z* calcd for $C_{31}H_{30}N_2O_3$ $[M+H]^+$ = 479.2329, found = 479.2331; The ee value was 93%, $t_R$ (major) = 9.6 min, $t_R$ (minor) = 12.3 min (Chiralcel IA, λ = 254 nm, 10% i-PrOH/hexane, flow rate = 1.0 mL/min).

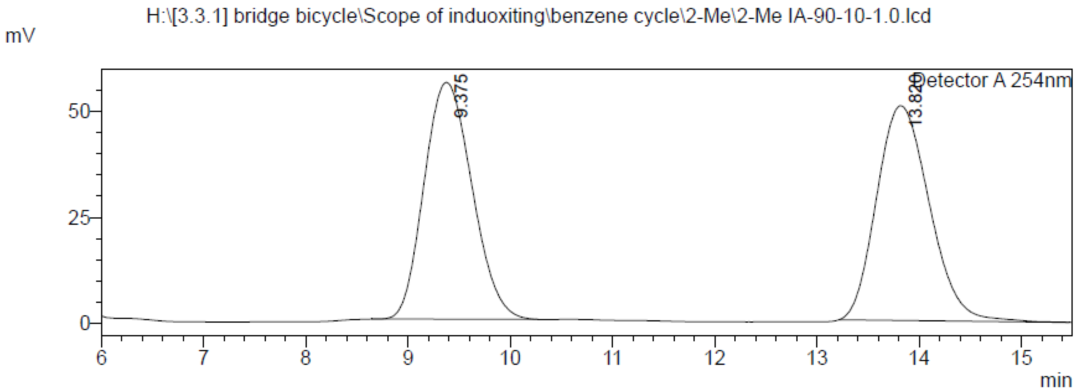


Detector A 254nm

| Peak# | Ret. Time | Height | Height% | Area | Area% |
|---|---|---|---|---|---|
| 1 | 9.375 | 55785 | 52.455 | 1821178 | 50.073 |
| 2 | 13.820 | 50563 | 47.545 | 1815896 | 49.927 |
| Total | | 106348 | 100.000 | 3637074 | 100.000 |

Racemic **4k**

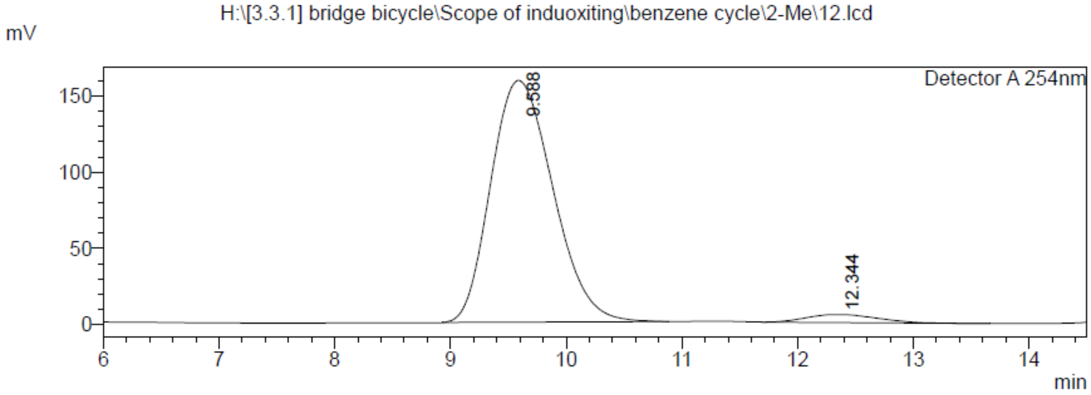


Detector A 254nm

| Peak# | Ret. Time | Height | Height% | Area | Area% |
|---|---|---|---|---|---|
| 1 | 9.588 | 158809 | 96.812 | 5933518 | 96.512 |
| 2 | 12.344 | 5229 | 3.188 | 214423 | 3.488 |
| Total | | 164037 | 100.000 | 6147941 | 100.000 |

Enantiomerically enriched **4k**

**Methyl(6S,7S,13R)-7-(2-(3,4-dimethoxyphenyl)-2-oxoethyl)-8,13-dimethyl-5,7,8,13-tetrahydro-6H-6,13-epiminobenzo[4,5]cycloocta[1,2-b]indole-6-carboxylate (4l)**

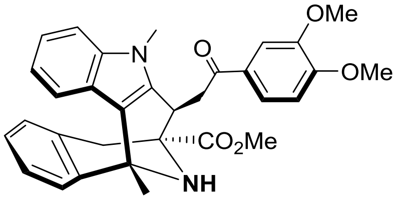


**4l**

A white solid; 46.1 mg, 88% yield; m.p. = 76.5-77.8 °C, $[\alpha]^{25}_{D}$ = -57.94 (*c* 0.29, $CHCl_3$); *dr* >20:1; $^1H$ NMR (400 MHz, $CDCl_3$) δ 7.69 (d, *J* = 7.2 Hz, 1H), 7.66 – 7.59 (m, 2H), 7.57 (d, *J* = 2.0 Hz, 1H), 7.20 (t, *J* = 8.0 Hz, 2H), 7.13 (t, *J* = 7.6 Hz, 1H),

7.05 (qd, $J$ = 7.0, 1.0 Hz, 2H), 6.94 (d, $J$ = 7.4 Hz, 1H), 6.86 (d, $J$ = 8.4 Hz, 1H), 4.19 (dd, $J$ = 6.0, 3.6 Hz, 1H), 3.93 (s, 6H), 3.87 (dd, $J$ = 17.6, 6.0 Hz, 1H), 3.68 (s, 3H), 3.51 (s, 3H), 3.28 (d, $J$ = 17.6 Hz, 1H), 2.99 (dd, $J$ = 17.6, 3.6 Hz, 1H), 2.73 (d, $J$ = 17.6 Hz, 1H), 2.17 (s, 3H); $^{13}$C NMR (100 MHz, $CDCl_3$) δ 197.37, 175.10, 153.67, 149.19, 145.87, 137.79, 136.30, 133.74, 130.07, 129.02, 126.28, 126.15, 124.12, 123.36, 121.74, 121.16, 119.29, 119.25, 112.43, 110.27, 110.17, 109.28, 61.69, 56.20, 56.12, 53.75, 52.41, 40.57, 40.25, 37.45, 29.44, 25.12; HRMS (ESI) *m/z* calcd for $C_{32}H_{32}N_2O_5$ $[M+H]^+$ = 525.2384, found = 525.2382; The ee value was 96%, $t_R$ (major) = 17.6 min, $t_R$ (minor) = 21.2 min (Chiralcel IA, λ = 254 nm, 10% i-PrOH/hexane, flow rate = 1.0 mL/min).

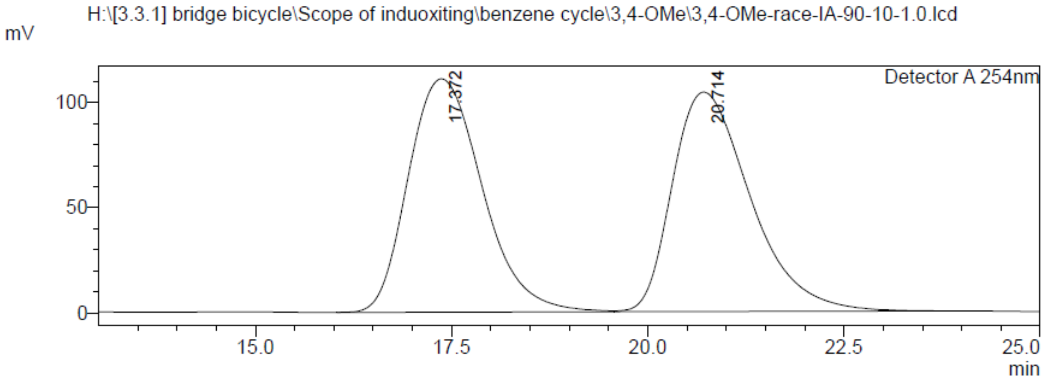


Detector A 254nm

| Peak# | Ret. Time | Height | Height% | Area | Area% |
|---|---|---|---|---|---|
| 1 | 17.372 | 110854 | 51.542 | 7251595 | 50.183 |
| 2 | 20.714 | 104222 | 48.458 | 7198580 | 49.817 |
| Total | | 215076 | 100.000 | 14450176 | 100.000 |

Racemic **4l**

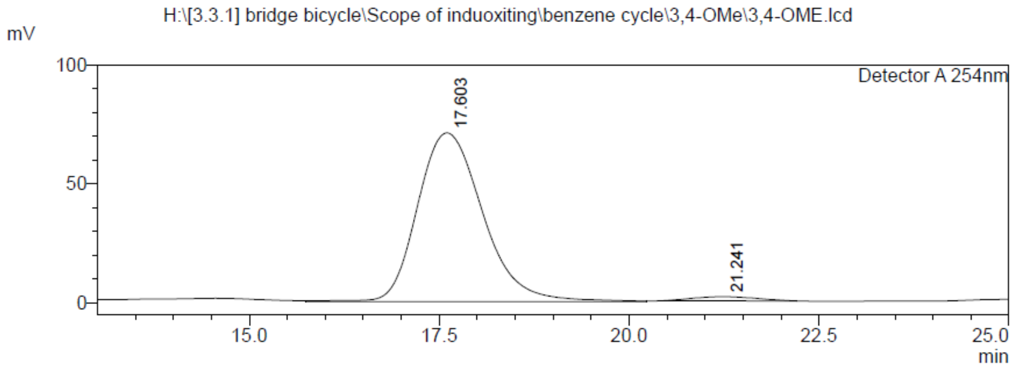


Detector A 254nm

| Peak# | Ret. Time | Height | Height% | Area | Area% |
|---|---|---|---|---|---|
| 1 | 17.603 | 70962 | 97.642 | 4250320 | 97.702 |
| 2 | 21.241 | 1714 | 2.358 | 99976 | 2.298 |
| Total | | 72675 | 100.000 | 4350296 | 100.000 |

Enantiomerically enriched **4l**

**Methyl(6S,7S,13R)-8,13-dimethyl-7-(2-(naphthalen-1-yl)-2-oxoethyl)-5,7,8,13-tetrahydro-6H-6,13-epiminobenzo[4,5]cycloocta[1,2-b]indole-6-carboxylate (4m)**

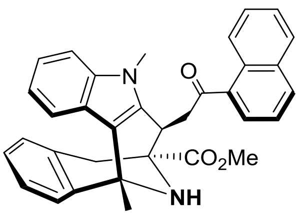


**4m**

A white solid; 46.7 mg, 91% yield; m.p. = 140.5-141.7 °C, $[\alpha]^{25}_{D}$ = -41.11 (*c* 0.31, $CHCl_3$); *dr* >20:1; $^1$H NMR (400 MHz, $CDCl_3$) δ 8.83 (d, *J* = 8.8 Hz, 1H), 8.00 (d, *J* = 8.2 Hz, 1H), 7.96 – 7.87 (m, 2H), 7.70 (d, *J* = 7.0 Hz, 1H), 7.68 – 7.62 (m, 2H), 7.57 (ddd, *J* = 8.0, 6.9, 1.1 Hz, 1H), 7.47 (dd, *J* = 8.0, 7.6 Hz, 1H), 7.22 (dd, *J* = 15.5, 7.8 Hz, 2H), 7.17 – 7.11 (m, 1H), 7.06 (td, *J* = 7.6, 1.0 Hz, 2H), 6.96 (d, *J* = 7.6 Hz, 1H), 4.36 (dd, *J* = 5.8, 3.0 Hz, 1H), 4.01 (dd, *J* = 18.6, 5.8 Hz, 1H), 3.76 (s, 3H), 3.54 (s, 3H), 3.32 (d, *J* = 17.8 Hz, 1H), 3.20 (dd, *J* = 18.6, 3.0 Hz, 1H), 2.79 (d, *J* = 17.8 Hz, 1H), 2.16 (s, 3H); $^{13}$C NMR (100 MHz, $CDCl_3$) δ 201.84, 175.31, 145.90, 137.86, 136.45, 135.05, 134.15, 133.79, 133.44, 130.40, 129.07, 128.90, 128.72, 128.29, 126.64, 126.32, 126.21, 125.95, 124.59, 124.20, 121.79, 121.19, 119.34, 119.31, 112.51, 109.33, 61.91, 53.80, 52.67, 44.85, 39.65, 37.52, 29.56, 25.11; HRMS (ESI) *m/z* calcd for $C_{34}H_{30}N_2O_3$ $[M+H]^+$ = 515.2329, found = 515.2325; The ee value was 85%, $t_R$ (major) = 16.6 min, $t_R$ (minor) = 11.0 min (Chiralcel IB, λ = 254 nm, 10% i-PrOH/hexane, flow rate = 1.0 mL/min).

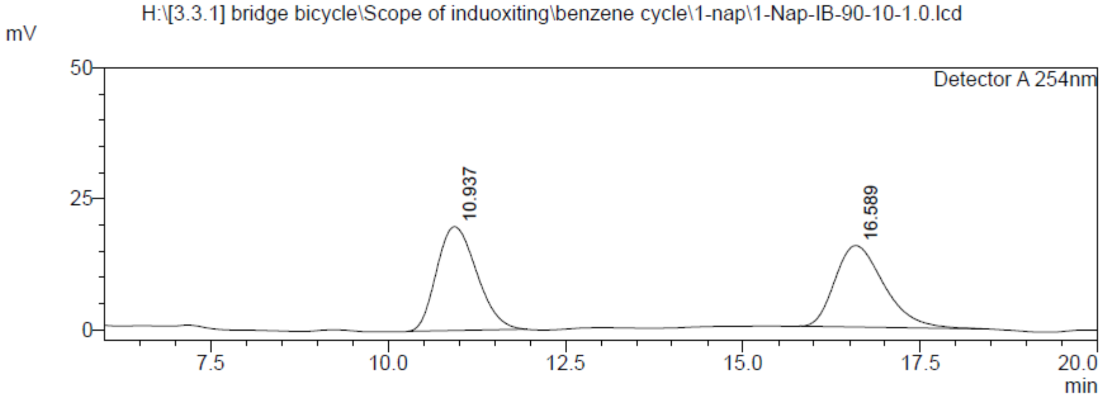


Detector A 254nm

| Peak# | Ret. Time | Height | Height% | Area | Area% |
|---|---|---|---|---|---|
| 1 | 10.937 | 19808 | 56.034 | 780325 | 51.053 |
| 2 | 16.589 | 15542 | 43.966 | 748150 | 48.947 |
| Total | | 35350 | 100.000 | 1528475 | 100.000 |

Racemic **4m**

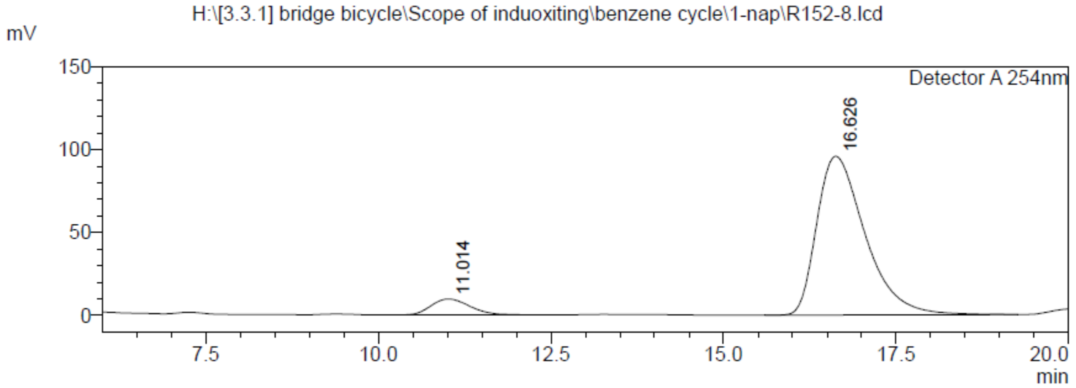


Detector A 254nm

| Peak# | Ret. Time | Height | Height% | Area | Area% |
|---|---|---|---|---|---|
| 1 | 11.014 | 9562 | 9.083 | 372290 | 7.370 |
| 2 | 16.626 | 95711 | 90.917 | 4679150 | 92.630 |
| Total | | 105273 | 100.000 | 5051440 | 100.000 |

Enantiomerically enriched **4m**

**Methyl(6S,7S,13R)-8,13-dimethyl-7-(2-(naphthalen-2-yl)-2-oxoethyl)-5,7,8,13-tetrahydro-6H-6,13-epiminobenzo[4,5]cycloocta[1,2-b]indole-6-carboxylate(4n)**

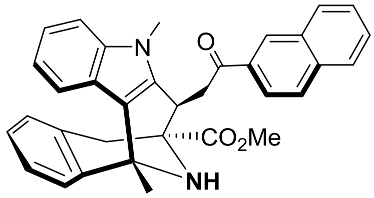


**4n**

A white solid; 46.2 mg, 90% yield; m.p. = 143.5-144.2 °C, $[\alpha]^{25}_{D}$ = -5.65 (*c* 0.45, $CHCl_3$); *dr* >20:1; $^1$H NMR (400 MHz, $CDCl_3$) δ 8.47 (s, 1H), 8.09 (dd, *J* = 8.6, 1.8 Hz, 1H), 7.94 (d, *J* = 8.0 Hz, 1H), 7.90 (d, *J* = 8.8 Hz, 1H), 7.87 (d, *J* = 8.0 Hz, 1H), 7.70 (d, *J* = 7.2 Hz, 1H), 7.64 (d, *J* = 8.0 Hz, 1H), 7.62 – 7.58 (m, 1H), 7.56 – 7.52 (m, 1H), 7.20 (t, *J* = 7.8 Hz, 2H), 7.14 – 7.12 (m, 1H), 7.05 (td, *J* = 8.8, 1.2 Hz, 2H), 6.95 (d, *J* = 7.2 Hz, 1H), 4.29 (dd, *J* =6.0, 3.2 Hz, 1H), 4.07 (dd, *J* = 18.0, 6.0 Hz, 1H), 3.70 (s, 3H), 3.49 (s, 3H), 3.30 (d, *J* = 17.6 Hz, 1H), 3.16 (dd, *J* = 18.0, 3.2 Hz, 1H), 2.75 (d, *J* = 17.6 Hz, 1H), 2.19 (s, 3H); $^{13}$C NMR (100 MHz, $CDCl_3$) δ 198.47 , 175.12, 145.87, 137.85, 136.30, 135.83, 134.12, 133.78, 132.64, 130.36, 129.89, 129.07, 128.81, 128.71, 127.86, 126.99, 126.33, 126.21, 124.17, 123.94, 121.80, 121.21, 119.34, 119.32, 112.54, 109.31, 61.85, 53.83, 52.51, 41.29, 39.87, 37.46, 29.53, 25.16; HRMS (ESI) *m/z* calcd for $C_{34}H_{30}N_2O_3$ $[M+H]^+$ = 515.2329, found = 515.2327; The ee value was 83%, $t_R$ (major) = 22.0 min, $t_R$ (minor) = 13.5 min (Chiralcel IB, λ = 254 nm, 10% i-PrOH/hexane, flow rate = 1.0 mL/min).

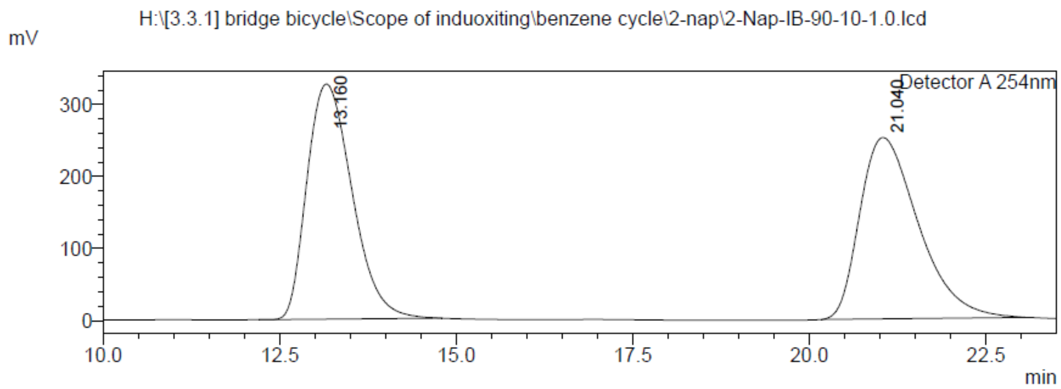


Detector A 254nm

| Peak# | Ret. Time | Height | Height% | Area | Area% |
|---|---|---|---|---|---|
| 1 | 13.160 | 326500 | 56.450 | 14564713 | 50.012 |
| 2 | 21.040 | 251888 | 43.550 | 14557746 | 49.988 |
| Total | | 578388 | 100.000 | 29122459 | 100.000 |

Racemic **4n**

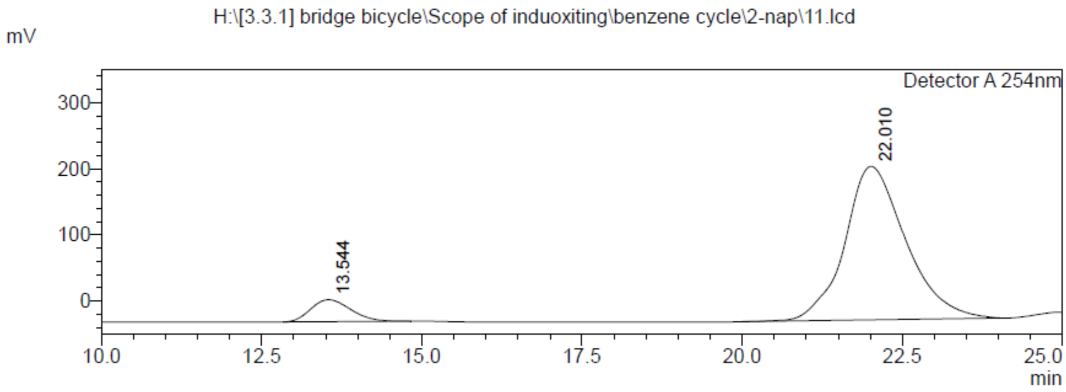


Detector A 254nm

| Peak# | Ret. Time | Height | Height% | Area | Area% |
|---|---|---|---|---|---|
| 1 | 13.544 | 33259 | 12.534 | 1442277 | 8.557 |
| 2 | 22.010 | 232102 | 87.466 | 15413061 | 91.443 |
| Total | | 265361 | 100.000 | 16855338 | 100.000 |

Enantiomerically enriched **4n**

**Methyl(6S,7S,13R)-8,13-dimethyl-7-(2-oxo-2-(1H-pyrrol-2-yl)ethyl)-5,7,8,13-tetrahydro-6H-6,13-epiminobenzo[4,5]cycloocta[1,2-b]indole-6-carboxylate(4o)**

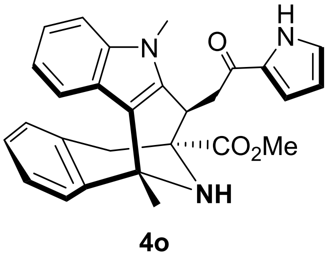


**4o**

A white solid; 39.4 mg, 87% yield; m.p. = 80.1-82.5 °C, $[\alpha]^{25}_{D}$ = -25.12 (*c* 0.24, $CHCl_3$); *dr* >20:1; $^1H$ NMR (400 MHz, $CDCl_3$) δ 9.53 (s, 1H), 7.68 (d, *J* = 7.6 Hz,

1H), 7.62 (d, $J$ = 8.0 Hz, 1H), 7.20 (dd, $J$ = 15.4, 7.8 Hz, 2H), 7.13 (t, $J$ = 7.4 Hz, 1H), 7.08 – 7.00 (m, 3H), 6.92 (d, $J$ = 6.6 Hz, 2H), 6.26 (dd, $J$ = 6.2, 2.6 Hz, 1H), 4.08 (dd, $J$ = 6.2, 3.0 Hz, 1H), 3.71 (s, 2H), 3.69 – 3.63 (m, 1H), 3.45 (s, 3H), 3.22 (d, $J$ = 17.8 Hz, 1H), 2.86 (dd, $J$ = 16.8, 3.0 Hz, 1H), 2.69 (d, $J$ = 17.8 Hz, 1H), 2.15 (s, 3H), 1.70 (s, 1H).;.$^{13}$C NMR (100 MHz, $CDCl_3$) δ 188.83, 175.03, 145.95, 137.83, 136.16, 133.74, 132.07, 129.01, 126.26, 126.17, 125.19, 124.14, 121.79, 121.13, 119.30, 119.26, 117.34, 112.56, 111.21, 109.27, 61.53, 53.73, 52.17, 40.63, 40.34, 37.62, 29.39, 25.12; HRMS (ESI) *m/z* calcd for $C_{28}H_{27}N_3O_3$ $[M+H]^+$ = 454.2125, found = 454.2128; The ee value was 98%, $t_R$ (major) = 15.8 min, $t_R$ (minor) = 31.1 min (Chiralcel IE, λ = 254 nm, 10% i-PrOH/hexane, flow rate = 1.0 mL/min).

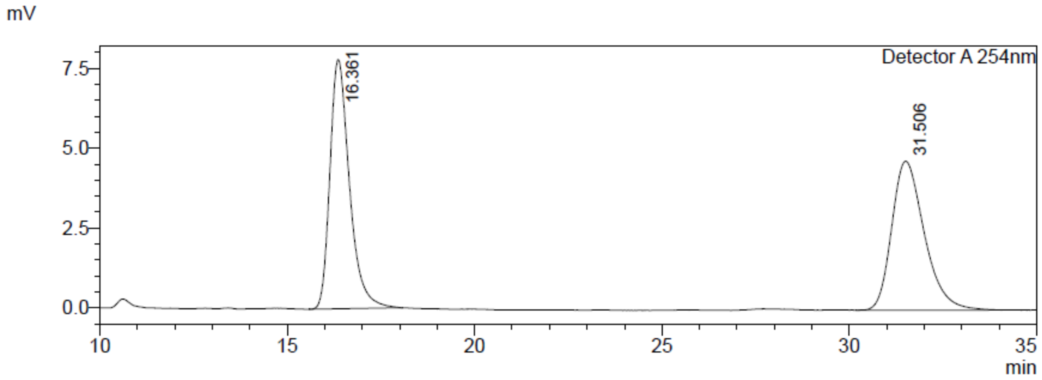


**<Peak Table>**

Detector A 254nm

| Peak# | Ret. Time | Height | Height% | Conc. | Area | Area% |
|---|---|---|---|---|---|---|
| 1 | 16.361 | 7810 | 62.583 | 50.024 | 283032 | 50.024 |
| 2 | 31.506 | 4669 | 37.417 | 49.976 | 282765 | 49.976 |
| Total | | 12479 | 100.000 | | 565797 | 100.000 |

Racemic **4o**

H:\[3.3.1] bridge bicycle\Scope of induoxiting\hetrocycle and alkyl\pyrrol\TJP-R134-5-2.lcd

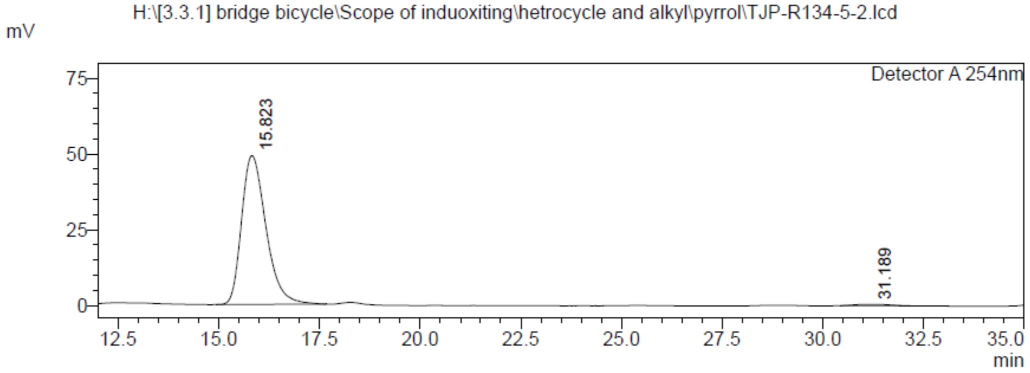


Detector A 254nm

| Peak# | Ret. Time | Height | Height% | Area | Area% |
|---|---|---|---|---|---|
| 1 | 15.823 | 49127 | 99.059 | 2072037 | 98.727 |
| 2 | 31.189 | 467 | 0.941 | 26712 | 1.273 |
| Total | | 49594 | 100.000 | 2098748 | 100.000 |

Enantiomerically enriched **4o**

**Methyl(6S,7S,13R)-7-(2-(furan-2-yl)-2-oxoethyl)-8,13-dimethyl-5,7,8,13-tetrahydro-6H-6,13-epiminobenzo[4,5]cycloocta[1,2-b]indole-6-carboxylate(4p)**

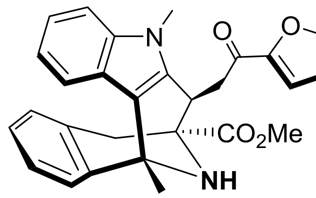


**4p**

A white solid; 38.1 mg, 84% yield; m.p. =170.6-171.4 °C, $[\alpha]^{25}_{D}$ = -25.68 (*c* 0.44, $CHCl_3$); *dr* >20:1; $^1H$ NMR (400 MHz, $CDCl_3$) δ 7.69 (d, *J* = 7.6 Hz, 1H), 7.63 (d, *J* = 8.0 Hz, 1H), 7.60 (d, *J* = 1.0 Hz, 1H), 7.25 – 7.17 (m, 3H), 7.14 (dd, *J* = 11.0, 4.0 Hz, 1H), 7.05 (dt, *J* = 12.4, 6.0 Hz, 2H), 6.93 (d, *J* = 7.4 Hz, 1H), 6.52 (dd, *J* = 3.2, 1.6 Hz, 1H), 4.11 (dd, *J* = 6.4, 3.2 Hz, 1H), 3.74 (dd, *J* = 17.2, 6.4 Hz, 1H), 3.71 (s, 3H), 3.55 (s, 3H), 3.23 (d, *J* = 17.8 Hz, 1H), 2.94 (dd, *J* = 17.2, 3.2 Hz, 1H), 2.69 (d, *J* = 17.8 Hz, 1H), 2.15 (s, 3H); $^{13}C$ NMR (100 MHz, $CDCl_3$) δ 187.53, 174.96, 152.56, 147.05, 145.87, 137.84, 135.85, 133.65, 129.02, 126.28, 126.19, 124.10, 121.76, 121.19, 119.30, 118.18, 112.61, 112.50, 109.30, 61.53, 53.72, 52.29, 41.12, 39.83, 37.44, 29.42, 25.07; HRMS (ESI) *m/z* calcd for $C_{28}H_{26}N_2O_4$ $[M+H]^+$ = 455.1965, found = 455.1964; The ee value was 97%, $t_R$ (major) = 15.3 min, $t_R$ (minor) = 20.7 min (Chiralcel IB, λ = 254 nm, 10% i-PrOH/hexane, flow rate = 1.0 mL/min).

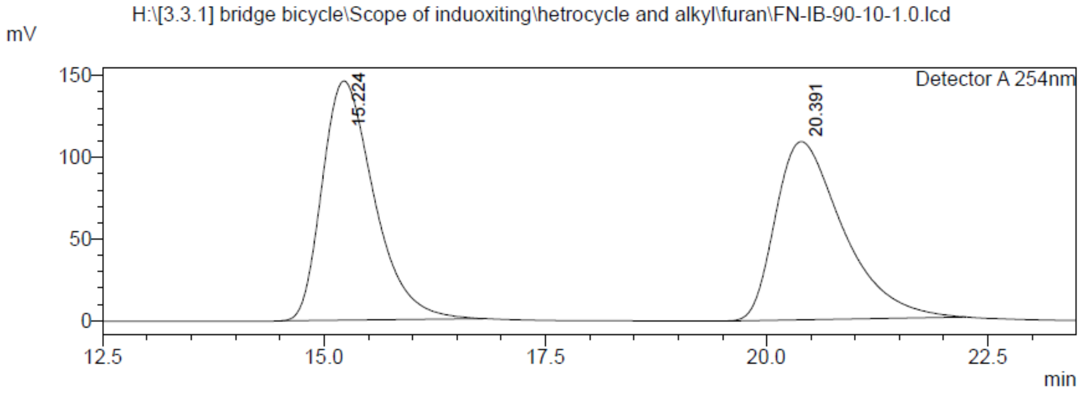


Detector A 254nm

| Peak# | Ret. Time | Height | Height% | Area | Area% |
|---|---|---|---|---|---|
| 1 | 15.224 | 146295 | 57.318 | 5975965 | 50.686 |
| 2 | 20.391 | 108940 | 42.682 | 5814313 | 49.314 |
| Total | | 255235 | 100.000 | 11790278 | 100.000 |

Racemic **4p**

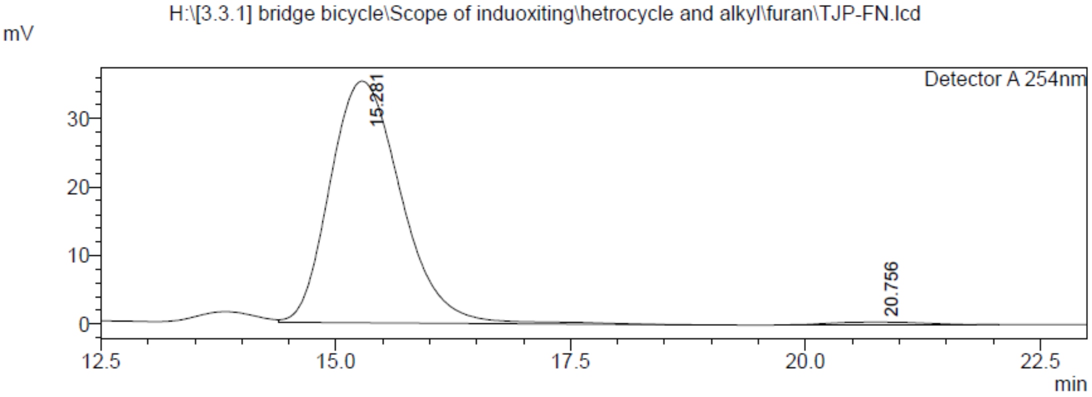


Detector A 254nm

| Peak# | Ret. Time | Height | Height% | Area | Area% |
|---|---|---|---|---|---|
| 1 | 15.281 | 35285 | 98.806 | 1866650 | 98.450 |
| 2 | 20.756 | 426 | 1.194 | 29390 | 1.550 |
| Total | | 35712 | 100.000 | 1896039 | 100.000 |

Enantiomerically enriched **4p**

**Methyl(6S,7S,13R)-8,13-dimethyl-7-(2-oxo-2-(thiophen-2-yl)ethyl)-5,7,8,13-tetrahydro-6H-6,13-epiminobenzo[4,5]cycloocta[1,2-b]indole-6-carboxylate (4q)**

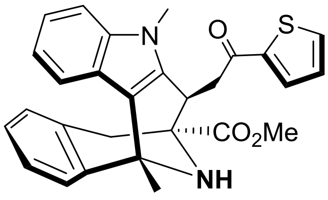


**4q**

A pale yellow solid; 41.3 mg, 88% yield; m.p. =188.5-189.6 °C, $[\alpha]^{25}_D$ = -35.23 (*c* 0.26, $CHCl_3$); *dr* >20:1; $^1$H NMR (400 MHz, $CDCl_3$) δ 7.71 (dd, *J* = 9.6, 5.6 Hz, 2H), 7.64 (t, *J* = 6.0 Hz, 2H), 7.21 (dd, *J* = 12.8, 7.8 Hz, 2H), 7.16 – 7.09 (m, 2H), 7.06 (dd, *J* = 12.8, 7.2 Hz, 2H), 6.94 (d, *J* = 7.2 Hz, 1H), 4.14 (dd, *J* = 6.0, 3.2 Hz, 1H), 3.83 (dd, *J* = 17.2, 6.0 Hz, 1H), 3.71 (s, 3H), 3.52 (s, 3H), 3.25 (d, *J* = 18.0 Hz, 1H), 3.01 (dd, *J* = 17.2, 3.0 Hz, 1H), 2.70 (d, *J* = 18.0 Hz, 1H), 2.17 (s, 3H); $^{13}$C NMR (100 MHz, $CDCl_3$) δ 191.75, 175.00, 145.87, 144.42, 137.84, 135.85, 134.53, 133.66, 132.86, 129.03, 128.48, 126.29, 126.19, 124.09, 121.78, 121.20, 119.30, 112.62, 109.30, 61.53, 53.72, 52.30, 41.78, 40.24, 37.40, 29.43, 25.09; HRMS (ESI) *m/z* calcd for $C_{28}H_{26}N_2O_3S$ $[M+H]^+$ = 471.1737, found = 471.1739; The ee value was 87%, $t_R$ (major) = 17.0 min, $t_R$ (minor) = 20.7 min (Chiralcel IC, λ = 254 nm, 5% i-PrOH/hexane, flow rate = 1.0 mL/min).

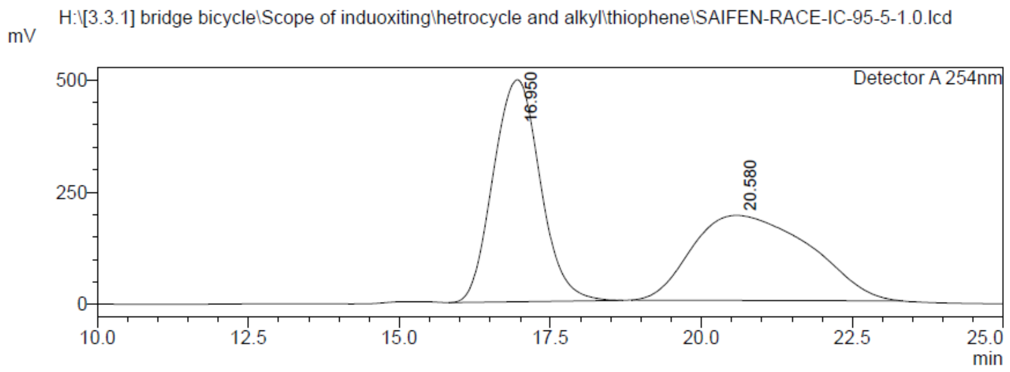


Detector A 254nm

| Peak# | Ret. Time | Height | Height% | Area | Area% |
|---|---|---|---|---|---|
| 1 | 16.950 | 494288 | 72.310 | 26810438 | 50.896 |
| 2 | 20.580 | 189283 | 27.690 | 25866685 | 49.104 |
| Total | | 683570 | 100.000 | 52677123 | 100.000 |

Racemic **4q**

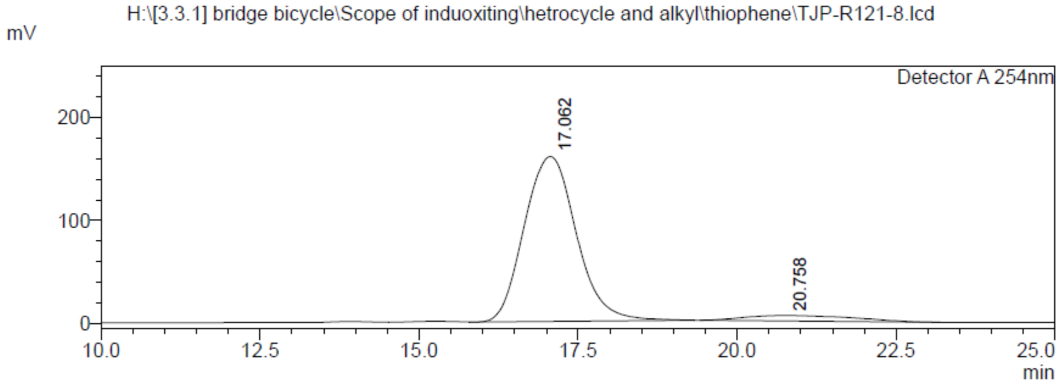


Detector A 254nm

| Peak# | Ret. Time | Height | Height% | Area | Area% |
|---|---|---|---|---|---|
| 1 | 17.062 | 160061 | 96.847 | 9128792 | 93.648 |
| 2 | 20.758 | 5211 | 3.153 | 619207 | 6.352 |
| Total | | 165272 | 100.000 | 9748000 | 100.000 |

Enantiomerically enriched **4q**

**Methyl(6S,7S,13R)-8,13-dimethyl-7-(2-oxo-2-(pyridin-2-yl)ethyl)-5,7,8,13-tetrahydro-6H-6,13-epiminobenzo[4,5]cycloocta[1,2-b]indole-6-carboxylate(4r)**

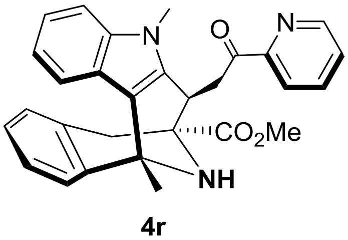


**4r**

A pale yellow solid; 37.7 mg, 81% yield; m.p. =181.9-182.5 °C, $[\alpha]^{25}_{D}$ = -21.22 (*c* 0.18, $CHCl_3$); *dr* >20:1; $^1$H NMR (400 MHz, $CDCl_3$) δ 8.67 (ddd, *J* = 4.8, 1.8, 0.8 Hz, 1H), 8.11 (dt, *J* = 8.0, 1.0 Hz, 1H), 7.86 (td, *J* = 7.8, 1.8 Hz, 1H), 7.67 (dd, *J* = 7.8, 0.8

Hz, 1H), 7.62 (d, *J* = 8.0 Hz, 1H), 7.47 (ddd, *J* = 7.6, 4.7, 1.2 Hz, 1H), 7.24 (d, *J* = 8.2 Hz, 1H), 7.18 (t, *J* = 7.6 Hz, 1H), 7.15 – 7.10 (m, 1H), 7.07 – 6.98 (m, 2H), 6.91 (d, *J* = 7.6 Hz, 1H), 4.22 (t, *J* = 8.4 Hz, 1H), 4.19 – 4.14 (m, 1H), 3.80 (s, 3H), 3.51 (s, 3H), 3.30 – 3.25 (m, 1H), 3.25 – 3.20 (m, 1H), 2.70 (d, *J* = 17.8 Hz, 1H), 2.13 (s, 3H).; $^{13}C$ NMR (100 MHz, $CDCl_3$) δ 200.30, 175.07, 153.35, 149.11, 145.96, 137.84, 137.01, 136.28, 133.81, 128.96, 127.25, 126.18, 126.10, 124.22, 121.99, 121.69, 121.05, 119.32, 119.19, 112.61, 109.21, 61.49, 53.75, 52.19 , 40.53, 40.51, 37.80, 29.46, 24.95; HRMS (ESI) *m/z* calcd for $C_{29}H_{27}N_3O_3$ $[M+H]^+$ = 466.2125, found = 466.2123; The ee value was 96%, $t_R$ (major) = 15.7 min, $t_R$ (minor) = 18.0 min (Chiralcel IB, λ = 254 nm, 10% i-PrOH/hexane, flow rate = 1.0 mL/min).

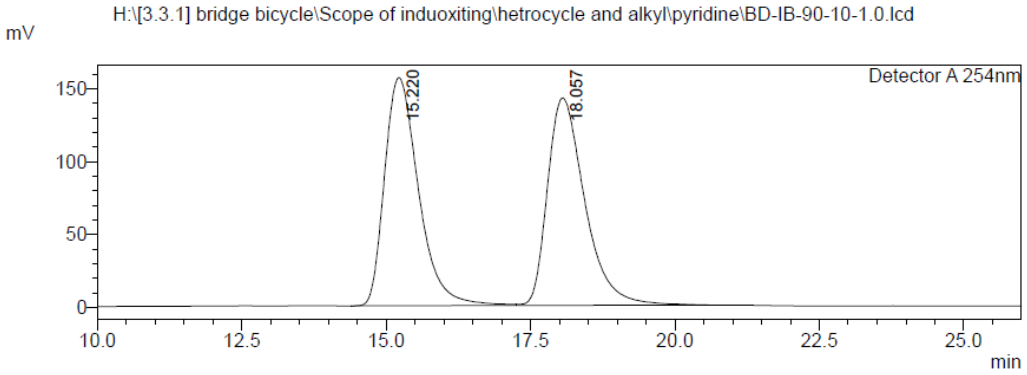


Detector A 254nm

| Peak# | Ret. Time | Height | Height% | Area | Area% |
|---|---|---|---|---|---|
| 1 | 15.220 | 156298 | 52.361 | 6407615 | 50.029 |
| 2 | 18.057 | 142201 | 47.639 | 6400186 | 49.971 |
| Total | | 298499 | 100.000 | 12807801 | 100.000 |

Racemic **4r**

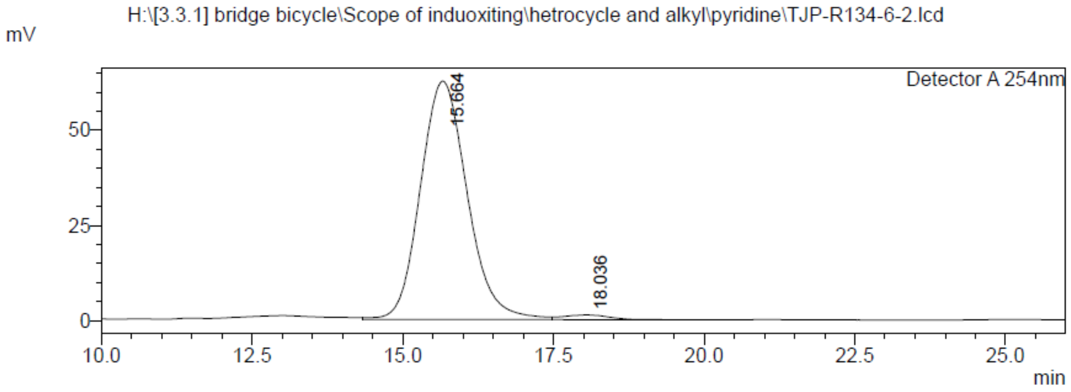


Detector A 254nm

| Peak# | Ret. Time | Height | Height% | Area | Area% |
|---|---|---|---|---|---|
| 1 | 15.664 | 62685 | 98.033 | 3393808 | 98.094 |
| 2 | 18.036 | 1258 | 1.967 | 65950 | 1.906 |
| Total | | 63943 | 100.000 | 3459758 | 100.000 |

Enantiomerically enriched **4r**

**Methyl(6S,7S,13R)-7-(2-(benzofuran-2-yl)-2-oxoethyl)-8,13-dimethyl-5,7,8,13-tetrahydro-6H-6,13-epiminobenzo[4,5]cycloocta[1,2-b]indole-6-carboxylate(4s)**

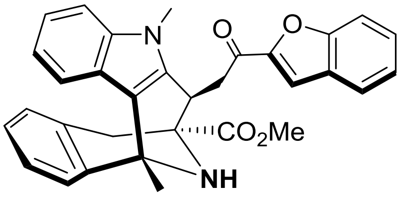


**4s**

A pale yellow solid; 41.3 mg, 82% yield; m.p. = 208.0-208.9 °C, $[\alpha]^{25}_{D}$ = -5.78 (*c* 0.40, $CHCl_3$); *dr* >20:1; $^{1}H$ NMR (400 MHz, $CDCl_3$) δ 7.69 (d, *J* = 7.6 Hz, 2H), 7.62 (d, *J* = 8.0 Hz, 1H), 7.58 (d, *J* = 8.4 Hz, 1H), 7.55 (s, 1H), 7.48 (t, *J* = 8.0 Hz, 1H), 7.31 (t, *J* = 7.6 Hz, 1H), 7.21 (dd, *J* = 16.0, 8.0 Hz, 2H), 7.13 (t, *J* = 7.6 Hz, 1H), 7.05 (q, *J* = 7.2 Hz, 2H), 6.93 (d, *J* = 7.2 Hz, 1H), 4.17 (dd, *J* = 6.2, 3.0 Hz, 1H), 3.87 (dd, *J* = 17.4, 6.2 Hz, 1H), 3.74 (s, 3H), 3.55 (s, 3H), 3.24 (d, *J* = 18.0 Hz, 1H), 3.07 (dd, *J* = 17.4, 3.0 Hz, 1H), 2.70 (d, *J* = 18.0 Hz, 1H), 2.15 (s, 3H); $^{13}C$ NMR (100 MHz, $CDCl_3$) δ 189.42, 174.97, 155.97, 152.39, 145.85, 137.88, 135.77, 133.63, 129.06, 128.64, 127.10, 126.33, 126.23, 124.13, 123.59, 121.79, 121.27, 119.35, 113.96, 112.68, 109.34, 61.63, 53.79, 52.44, 41.69, 39.77, 37.45, 29.50, 25.08; HRMS (ESI) *m/z* calcd for $C_{32}H_{28}N_2O_4$ $[M+H]^+$ = 505.2122, found = 505.2118; The ee value was 67%, $t_R$ (major) = 24.6 min, $t_R$ (minor) = 15.8 min (Chiralcel IB, λ = 254 nm, 10% i-PrOH/hexane, flow rate = 1.0 mL/min).

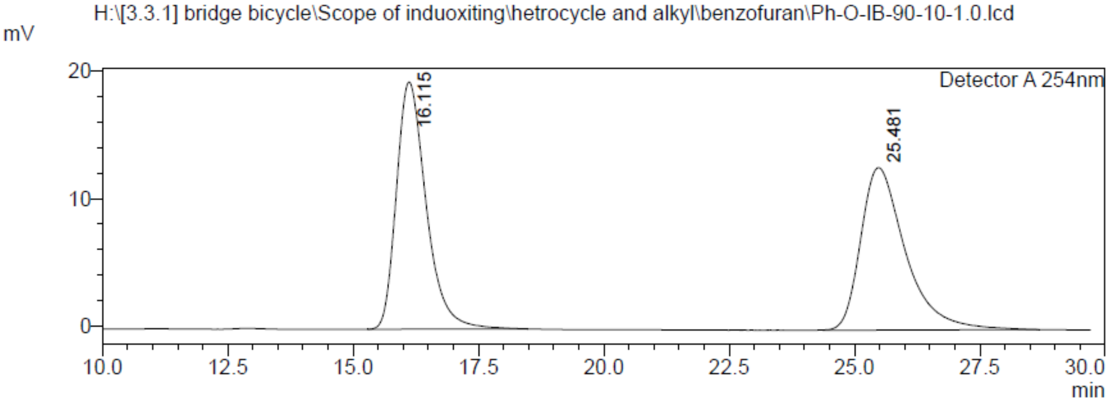


Detector A 254nm

| Peak# | Ret. Time | Height | Height% | Area | Area% |
|---|---|---|---|---|---|
| 1 | 16.115 | 19344 | 60.352 | 800740 | 50.327 |
| 2 | 25.481 | 12708 | 39.648 | 790319 | 49.673 |
| Total | | 32053 | 100.000 | 1591058 | 100.000 |

Racemic **4s**

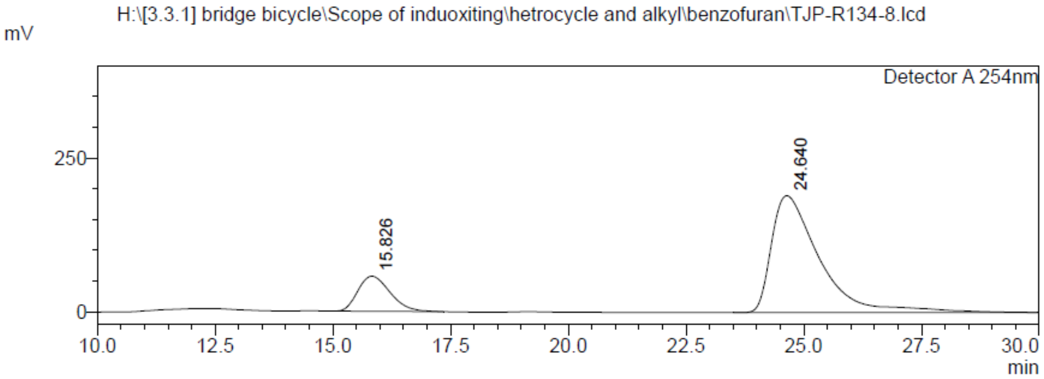


Detector A 254nm

| Peak# | Ret. Time | Height | Height% | Area | Area% |
|---|---|---|---|---|---|
| 1 | 15.826 | 56907 | 23.045 | 2709979 | 16.709 |
| 2 | 24.640 | 190035 | 76.955 | 13508497 | 83.291 |
| Total | | 246941 | 100.000 | 16218476 | 100.000 |

Enantiomerically enriched **4s**

**Methyl(6S,7S,13R)-7-(2-(benzo[b]thiophen-2-yl)-2-oxoethyl)-8,13-dimethyl-5,7,8,13-tetrahydro-6H-6,13-epiminobenzo[4,5]cycloocta[1,2-b]indole-6-carboxylate(4t)**

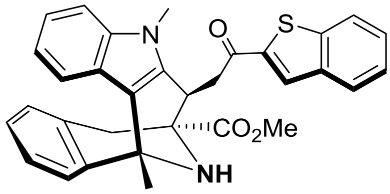


**4t**

A pale yellow solid; 42.6 mg, 82% yield; m.p. =202.1-203.7 °C, $[\alpha]^{25}_{D}$ = -9.89 (*c* 0.35, $CHCl_3$); *dr* >20:1; $^1$H NMR (400 MHz, $CDCl_3$) δ 7.98 (s, 1H), 7.87 (dd, *J* = 7.8, 4.0 Hz, 2H), 7.71 (d, *J* = 7.2 Hz, 1H), 7.65 (d, *J* = 8.0 Hz, 1H), 7.50 – 7.46 (m, 1H), 7.41 (t, *J* = 7.6 Hz, 1H), 7.22 (dd, *J* = 13.6, 7.8 Hz, 2H), 7.16 – 7.13 (m, 1H), 7.06 (qd, *J* = 7.2, 0.8 Hz, 2H), 6.95 (d, *J* = 7.6 Hz, 1H), 4.19 (dd, *J* = 6.4, 3.0 Hz, 1H), 4.00 (dd, *J* = 17.2, 6.4 Hz, 1H), 3.75 (s, 3H), 3.52 (s, 3H), 3.26 (d, *J* = 18.0 Hz 1H), 3.06 (dd, *J* = 17.2, 3.0 Hz, 1H), 2.73 (d, *J* = 18.0 Hz, 1H), 2.19 (s, 3H); $^{13}$C NMR (100 MHz, $CDCl_3$) δ 193.29, 175.00, 145.83, 143.73, 142.88, 139.26, 137.85, 135.71, 133.61, 130.11, 129.04, 127.78, 126.38, 126.32, 126.22, 125.20, 124.08, 123.06, 121.80, 121.25, 119.34, 119.30, 112.71, 109.33, 61.56, 53.76, 52.41, 41.55, 40.30, 37.37, 29.46, 25.12; HRMS (ESI) *m/z* calcd for $C_{32}H_{28}N_2O_3S$ $[M+H]^+$ = 521.1893, found = 521.1895; The ee value was 80%, $t_R$ (major) = 14.4 min, $t_R$

(minor) = 17.9 min (Chiralcel IC, λ = 254 nm, 10% i-PrOH/hexane, flow rate = 1.0 mL/min).

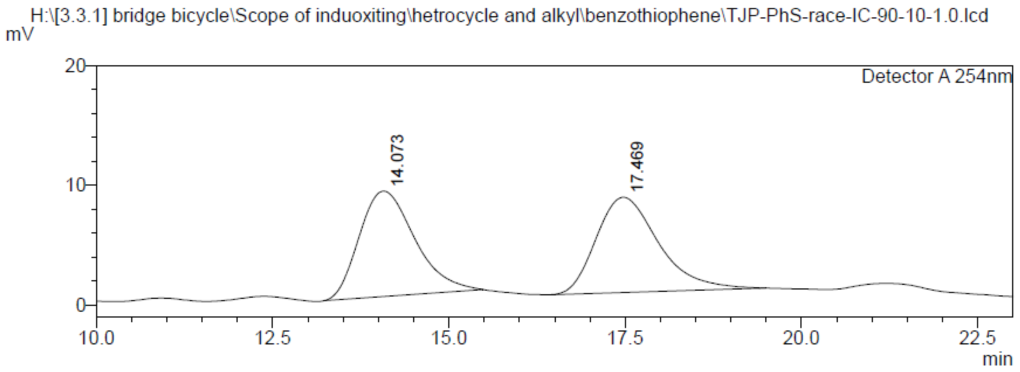


Detector A 254nm

| Peak# | Ret. Time | Height | Height% | Area | Area% |
|---|---|---|---|---|---|
| 1 | 14.073 | 8814 | 52.566 | 484042 | 49.597 |
| 2 | 17.469 | 7954 | 47.434 | 491916 | 50.403 |
| Total | | 16767 | 100.000 | 975958 | 100.000 |

Racemic **4t**

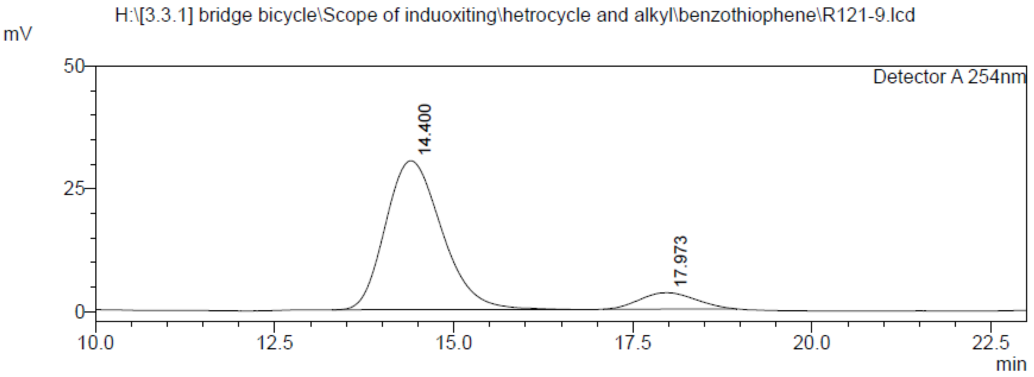


Detector A 254nm

| Peak# | Ret. Time | Height | Height% | Area | Area% |
|---|---|---|---|---|---|
| 1 | 14.400 | 30305 | 90.084 | 1659415 | 90.200 |
| 2 | 17.973 | 3336 | 9.916 | 180283 | 9.800 |
| Total | | 33641 | 100.000 | 1839698 | 100.000 |

Enantiomerically enriched **4t**

**Methyl(6S,7S,13R)-8,13-dimethyl-7-(2-oxo-2-(quinolin-3-yl)ethyl)-5,7,8,13-tetrahydro-6H-6,13-epiminobenzo[4,5]cycloocta[1,2-b]indole-6-carboxylate(4u)**

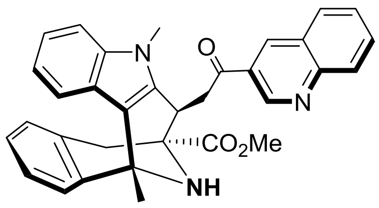


**4u**

A white solid; 41.7 mg, 81% yield; m.p. =75.1-76.4 $^{o}$C, $[\alpha]^{25}_{D}$ = -13.25 (*c* 0.21, $CHCl_3$); *dr* >20:1; $^1$H NMR (400 MHz, $CDCl_3$) δ 9.49 (d, *J* = 2.2 Hz, 1H), 8.65 (d, *J* = 2.0 Hz, 1H), 8.15 (d, *J* = 8.4 Hz, 1H), 7.89 – 7.80 (m, 2H), 7.69 (d, *J* = 7.6 Hz, 1H), 7.60 (dd, *J* = 11.6, 4.5 Hz, 2H), 7.25 – 7.17 (m, 2H), 7.15 – 7.10 (m, 1H), 7.05 (ddd, *J* = 15.1, 7.7, 1.0 Hz, 2H), 6.95 (d, *J* = 7.4 Hz, 1H), 6.95 (d, *J* = 7.4 Hz, 1H), 4.26 (dd, *J* = 6.0, 3.6 Hz, 1H), 4.01 (dd, *J* = 17.6, 6.0 Hz, 1H), 3.69 (s, 3H), 3.56 (s, 3H), 3.30 (d, *J* = 17.8 Hz, 1H), 3.23 (dd, *J* = 17.6, 3.6 Hz, 1H), 2.74 (d, *J* = 17.8 Hz, 1H), 2.17 (s, 3H).; $^{13}$C NMR (100 MHz, $CDCl_3$) δ 197.62, 174.99, 149.89, 149.17, 145.74, 137.85, 137.58, 135.70, 133.60, 132.28, 129.53, 129.50, 129.06, 128.95, 127.72, 126.78, 126.37, 126.26, 124.10, 121.78, 121.35, 119.44, 119.32, 112.65, 109.33, 61.78, 53.86, 52.61, 41.52, 39.75, 37.37, 29.54, 25.05; HRMS (ESI) *m/z* calcd for $C_{33}H_{29}N_3O_3$ $[M+H]^+$ = 516.2282, found = 516.2180; The ee value was 74%, $t_R$ (major) = 63.3 min, $t_R$ (minor) = 57.8 min (Chiralcel IB, λ = 254 nm, 10% i-PrOH/hexane, flow rate = 1.0 mL/min).

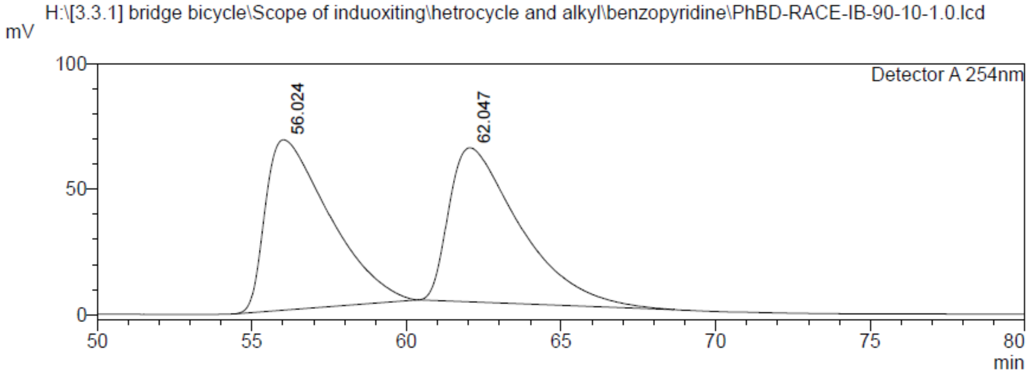


Detector A 254nm

| Peak# | Ret. Time | Height | Height% | Area | Area% |
|---|---|---|---|---|---|
| 1 | 56.024 | 67873 | 52.507 | 9819500 | 49.889 |
| 2 | 62.047 | 61392 | 47.493 | 9863387 | 50.111 |
| Total | | 129266 | 100.000 | 19682887 | 100.000 |

Racemic **4u**

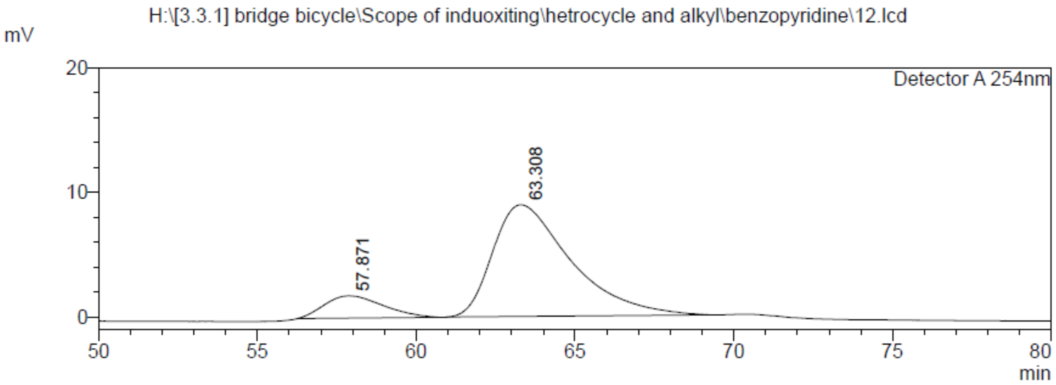


Detector A 254nm

| Peak# | Ret. Time | Height | Height% | Area | Area% |
|---|---|---|---|---|---|
| 1 | 57.871 | 1798 | 16.713 | 230514 | 13.061 |
| 2 | 63.308 | 8960 | 83.287 | 1534403 | 86.939 |
| Total | | 10757 | 100.000 | 1764916 | 100.000 |

Enantiomerically enriched **4u**

**Methyl(6S,7S,13R)-7-(2-cyclopropyl-2-oxoethyl)-8,13-dimethyl-5,7,8,13-tetrahydro-6H-6,13-epiminobenzo[4,5]cycloocta[1,2-b]indole-6-carboxylate(4v)**

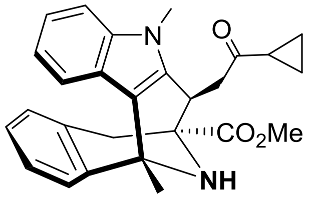


**4v**

A white solid; 35.1 mg, 82% yield; m.p. = 216.2-216.9 $^{o}$C, $[\alpha]^{25}_{D}$ = -65.50 (*c* 0.40, $CHCl_3$); *dr* >20:1; $^{1}$H NMR (400 MHz, $CDCl_3$) δ 7.67 (d, *J* = 7.6 Hz, 1H), 7.61 (d, *J* = 8.0 Hz, 1H), 7.23 (d, *J* = 8.4 Hz, 1H), 7.19 (t, *J* = 7.6 Hz, 1H), 7.15 – 7.11 (m, 1H), 7.07 – 7.01 (m, 2H), 6.93 (d, *J* = 7.6 Hz, 1H), 4.00 (dd, *J* = 5.8, 3.2 Hz, 1H), 3.78 (s, 3H), 3.62 (s, 3H), 3.34 (dd, *J* = 18.2, 5.8 Hz, 1H), 3.27 (d, *J* = 18.0 Hz, 1H), 2.79 (dd, *J* = 18.2, 3.2 Hz, 1H), 2.67 (d, *J* = 18.0 Hz, 1H), 2.13 (s, 3H), 1.92-1.85 (m, 1H), 1.08 – 1.06 (m, 2H), 0.92 – 0.87 (m, 2H); $^{13}$C NMR (100 MHz, $CDCl_3$) δ 208.89, 175.14 , 145.85, 137.80, 136.26, 133.77, 129.01, 126.27, 126.15, 124.17, 121.70, 121.13, 119.29, 119.27, 112.26, 109.27, 61.71, 53.73, 52.57, 45.93, 38.99, 37.38, 29.43, 25.05, 21.22, 11.21, 10.90; HRMS (ESI) *m/z* calcd for $C_{27}H_{28}N_2O_3$ $[M+H]^+$ = 429.2173, found = 429.2175; The ee value was >99%, $t_R$ (major) = 9.1 min, $t_R$ (minor) = 18.1 min (Chiralcel IA, λ = 254 nm, 10% i-PrOH/hexane, flow rate = 1.0 mL/min).

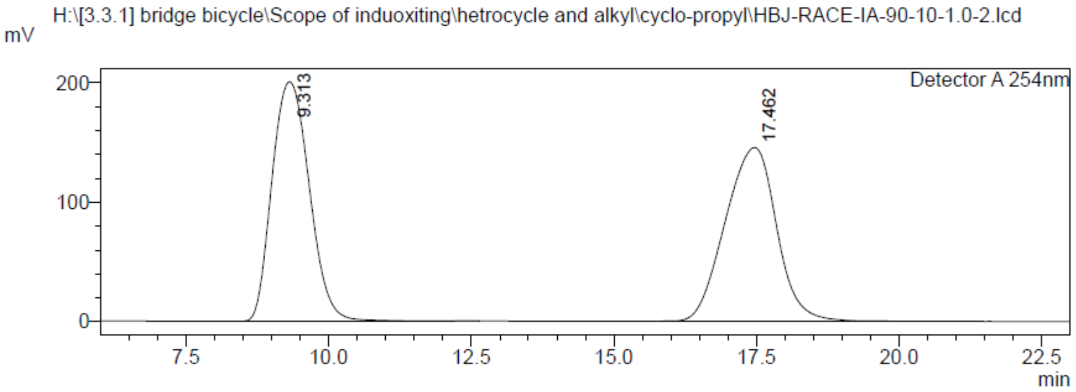


Detector A 254nm

| Peak# | Ret. Time | Height | Height% | Area | Area% |
|---|---|---|---|---|---|
| 1 | 9.313 | 200560 | 57.945 | 9171954 | 50.006 |
| 2 | 17.462 | 145563 | 42.055 | 9169899 | 49.994 |
| Total | | 346124 | 100.000 | 18341853 | 100.000 |

Racemic **4v**

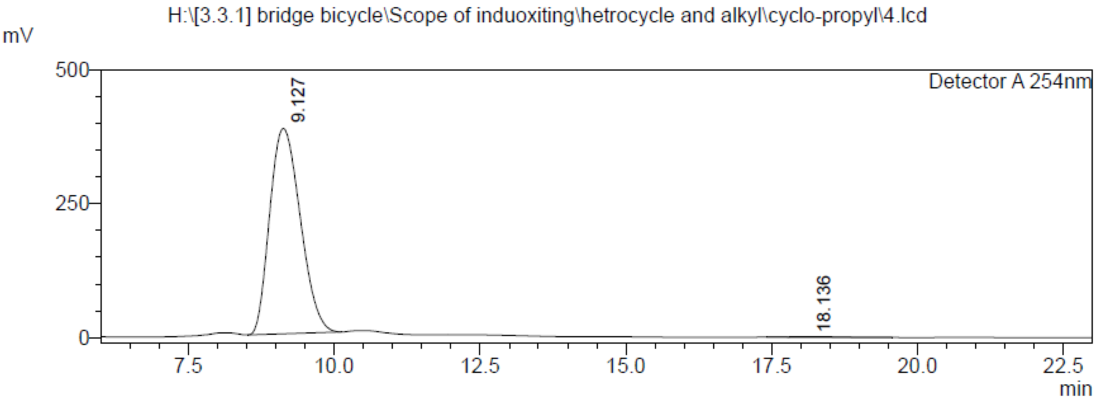


Detector A 254nm

| Peak# | Ret. Time | Height | Height% | Area | Area% |
|---|---|---|---|---|---|
| 1 | 9.127 | 383457 | 99.767 | 14005764 | 99.607 |
| 2 | 18.136 | 895 | 0.233 | 55267 | 0.393 |
| Total | | 384353 | 100.000 | 14061031 | 100.000 |

Enantiomerically enriched **4v**

**Methyl(6S,7S,13R)-7-(2-cyclopentyl-2-oxoethyl)-8,13-dimethyl-5,7,8,13-tetrahydro-6H-6,13-epiminobenzo[4,5]cycloocta[1,2-b]indole-6-carboxylate(4w)**

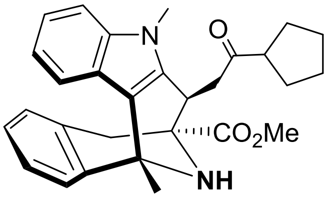


**4w**

A colorless oil; 36.9 mg, 81% yield; $[\alpha]^{25}_{D}$ = -19.38 (*c* 0.32, $CHCl_3$); *dr* >20:1; $^{1}H$ NMR (400 MHz, $CDCl_3$) δ 7.66 (d, *J* = 7.2 Hz, 1H), 7.61 (d, *J* = 7.6 Hz, 1H), 7.22 (d, *J* = 8.0 Hz, 1H), 7.18 (t, *J* = 7.6 Hz, 1H), 7.15 – 7.10 (m, 1H), 7.04 (qd, *J* = 7.2, 1.2 Hz, 2H), 6.93 (d, *J* = 7.6 Hz, 1H), 4.02 (dd, *J* = 5.6, 3.6 Hz, 1H), 3.76 (s, 3H), 3.61 (s, 3H), 3.26 (d, 18.0 Hz, 1H), 3.25 (dd, *J* = 18.4, 5.6 Hz, 1H), 2.84 – 2.76 (m, 1H), 2.72 (dd, *J* = 18.4, 3.6 Hz, 1H), 2.67 (d, *J* = 18.0 Hz, 1H), 2.12 (s, 3H), 1.82 – 1.77 (m, 3H), 1.68 – 1.56 (m, 5H); $^{13}C$ NMR (100 MHz, $CDCl_3$) δ 210.97, 175.16, 145.87, 137.81 , 136.55, 133.80, 129.02, 126.28, 126.15, 124.20, 121.68, 121.14, 119.32, 119.28, 112.15, 109.29, 61.76, 53.76, 52.58, 52.00, 44.96, 38.73, 37.44, 29.46, 29.35, 28.66 , 26.15, 26.04, 25.05; HRMS (ESI) *m/z* calcd for $C_{29}H_{32}N_2O_3$ $[M+H]^+$ = 457.2486, found = 457.2491; The ee value was 92%, $t_R$ (major) = 8.3 min, $t_R$ (minor) = 12.9 min (Chiralcel IA, λ = 254 nm, 10% i-PrOH/hexane, flow rate = 1.0 mL/min).

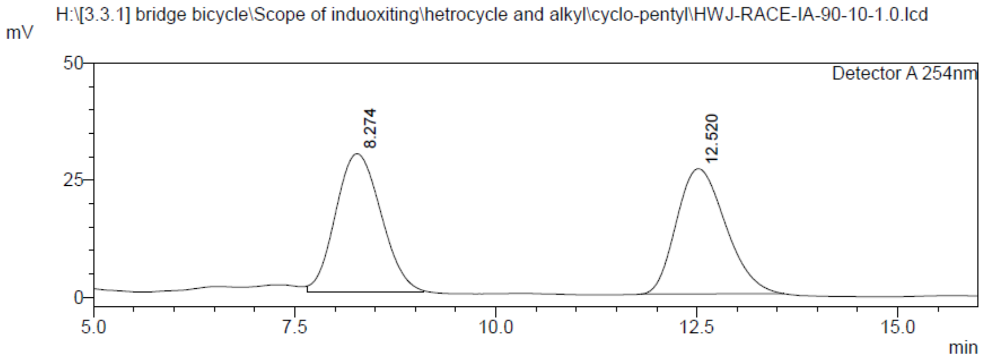


Detector A 254nm

| Peak# | Ret. Time | Height | Height% | Area | Area% |
|---|---|---|---|---|---|
| 1 | 8.274 | 29521 | 52.490 | 1162495 | 50.518 |
| 2 | 12.520 | 26720 | 47.510 | 1138652 | 49.482 |
| Total | | 56241 | 100.000 | 2301147 | 100.000 |

Racemic **4w**

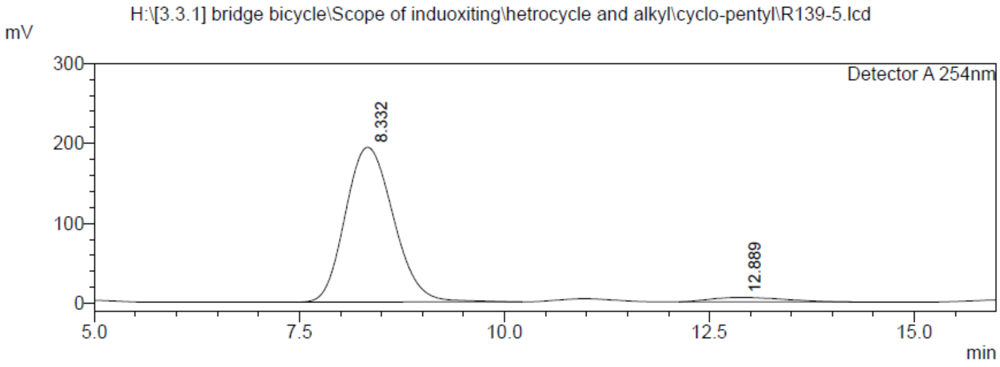


Detector A 254nm

| Peak# | Ret. Time | Height | Height% | Area | Area% |
|---|---|---|---|---|---|
| 1 | 8.332 | 193550 | 97.250 | 7899201 | 95.940 |
| 2 | 12.889 | 5474 | 2.750 | 334241 | 4.060 |
| Total | | 199024 | 100.000 | 8233443 | 100.000 |

Enantiomerically enriched **4w**

**Methyl(6S,7S,13R)-7-(2-cyclohexyl-2-oxoethyl)-8,13-dimethyl-5,7,8,13-tetrahydro-6H-6,13-epiminobenzo[4,5]cycloocta[1,2-b]indole-6-carboxylate(4x)**

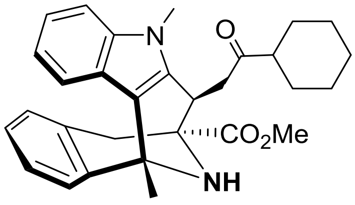

**4x**

A colorless oil; 39.5 mg, 84% yield; $[\alpha]^{25}_{D}$ = -15.56 (*c* 0.22, $CHCl_3$); *dr* >20:1; $^1H$ NMR (400 MHz, $CDCl_3$) δ 7.66 (d, *J* = 7.6 Hz, 1H), 7.60 (d, *J* = 7.6 Hz, 1H), 7.19(dd, *J* = 18.0, 8.0 Hz, 2H), 7.12 (t, *J* = 7.6 Hz, 2H), 7.03 (q, *J* = 6.8 Hz, 2H), 6.92 (d, *J* = 7.2 Hz, 1H), 4.02 (dd, *J* = 5.6, 3.2 Hz, 1H), 3.75 (s, 3H), 3.60 (s, 3H), 3.27 (dd, *J* = 18.8, 5.6 Hz, 1H), 3.26 (d, *J* = 18.4 Hz, 1H), 2.68 (d, *J* = 18.4 Hz, 1H), 2.66 (dd, *J* = 18.8, 3.2 Hz, 1H), 2.11(s, 3H), 2.32 – 2.25 (m, 1H), 1.85 – 1.74 (m, 4H), 1.34 – 1.20 (m, 6H); $^{13}C$ NMR (100 MHz, $CDCl_3$) δ 211.67, 175.17, 145.86, 137.78, 136.65, 133.82, 129.02, 126.29, 124.19, 121.69, 121.12, 119.31, 119.27, 112.10, 109.27, 100.13, 61.79, 53.76, 52.60, 51.30, 43.98, 38.31, 37.43, 29.42, 28.84, 28.49, 25.93, 25.85, 25.70, 25.06; HRMS (ESI) *m/z* calcd for $C_{30}H_{34}N_2O_3$ $[M+H]^+$ = 471.2642, found = 471.2641; The ee value was 96%, $t_R$ (major) = 8.3 min, $t_R$ (minor) = 13.8 min (Chiralcel IA, λ = 254 nm, 10% i-PrOH/hexane, flow rate = 1.0 mL/min).

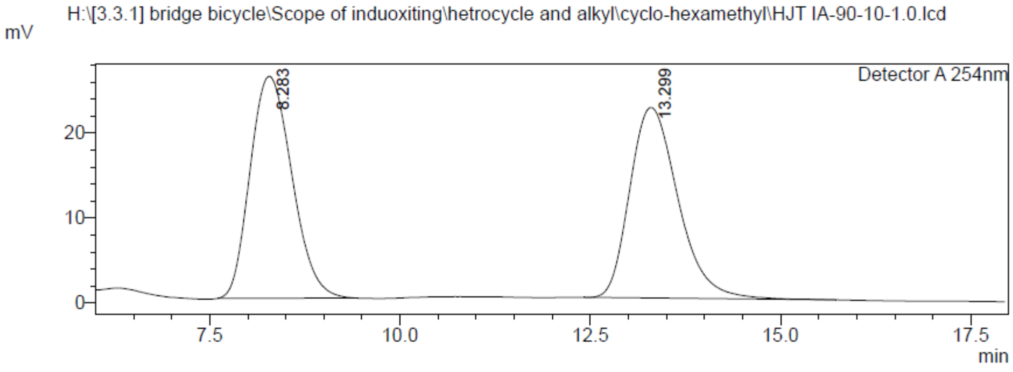


Detector A 254nm

| Peak# | Ret. Time | Height | Height% | Area | Area% |
|---|---|---|---|---|---|
| 1 | 8.283 | 26080 | 53.794 | 998408 | 50.206 |
| 2 | 13.299 | 22402 | 46.206 | 990216 | 49.794 |
| Total | | 48482 | 100.000 | 1988624 | 100.000 |

Racemic **4x**

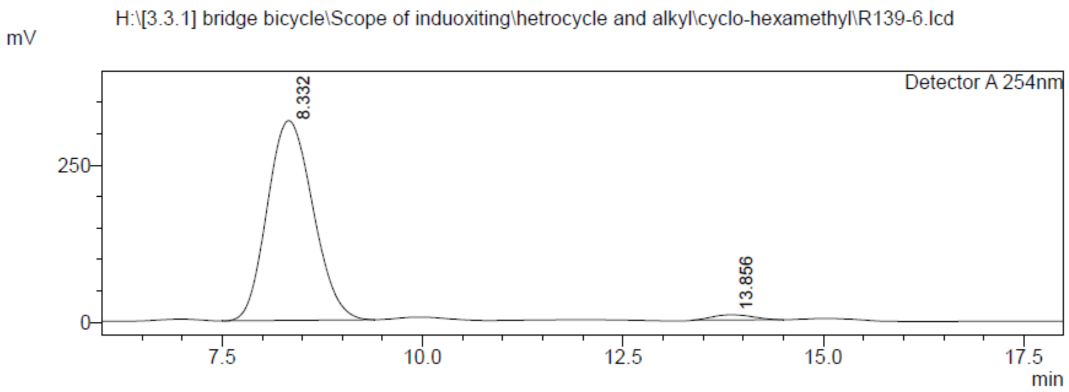


Detector A 254nm

| Peak# | Ret. Time | Height | Height% | Area | Area% |
|---|---|---|---|---|---|
| 1 | 8.332 | 317846 | 97.432 | 12611534 | 97.748 |
| 2 | 13.856 | 8379 | 2.568 | 290567 | 2.252 |
| Total | | 326225 | 100.000 | 12902101 | 100.000 |

Enantiomerically enriched **4x**

**Methyl(6S,7S,13R)-8,13-dimethyl-7-(2-oxopentyl)-5,7,8,13-tetrahydro-6H-6,13-epiminobenzo[4,5]cycloocta[1,2-b]indole-6-carboxylate(4y)**

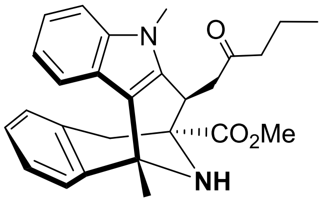


**4y**

A white solid; 34.8 mg, 81% yield; m.p. =150.5-152.0 °C, $[\alpha]^{25}_{D}$ = -12.50 (*c* 0.32, $CHCl_3$); *dr* >20:1; $^1$H NMR (400 MHz, $CDCl_3$) δ 7.66 (d, *J* = 7.2 Hz, 1H), 7.61 (d, *J* = 8.0 Hz, 1H), 7.23 (d, *J* = 8.0 Hz, 1H), 7.19 (t, *J* = 7.6 Hz, 1H), 7.15 – 7.11 (m, 1H), 7.04 (qd, *J* = 7.2, 1.0 Hz, 2H), 6.93 (d, *J* = 7.6 Hz, 1H), 4.00 (dd, *J* = 6.0, 3.6 Hz, 1H), 3.77 (s, 3H), 3.63 (s, 3H), 3.27 (d, *J* = 18.4 Hz, 1H), 3.23 (dd, *J* = 18.6, 6.0 Hz, 1H), 2.67(d, *J* = 18.4 Hz, 1H), 2.64(dd, *J* = 18.6, 3.6 Hz, 1H), 2.36 (td, *J* = 7.6, 2.8 Hz, 2H), 2.11 (s, 3H), 1.62 (q, *J* = 7.6 Hz, 2H), 0.93 (t, *J* = 7.6 Hz, 3H); $^{13}$C NMR (100 MHz, $CDCl_3$) δ 208.93, 175.14, 145.82, 137.79, 136.28, 133.75, 129.01, 126.29, 126.16, 124.17, 121.69, 121.16, 119.33, 119.28, 112.22, 109.28, 61.69, 53.74, 52.56, 45.71, 45.48, 38.79, 37.37, 29.41, 25.03, 17.41, 13.88; HRMS (ESI) *m/z* calcd for $C_{27}H_{30}N_2O_3$ $[M+H]^+$ = 431.2329, found = 431.2329; The ee value was 95%, $t_R$ (major) = 7.9 min, $t_R$ (minor) = 10.2 min (Chiralcel IA, λ = 254 nm, 10% i-PrOH/hexane, flow rate = 1.0 mL/min).

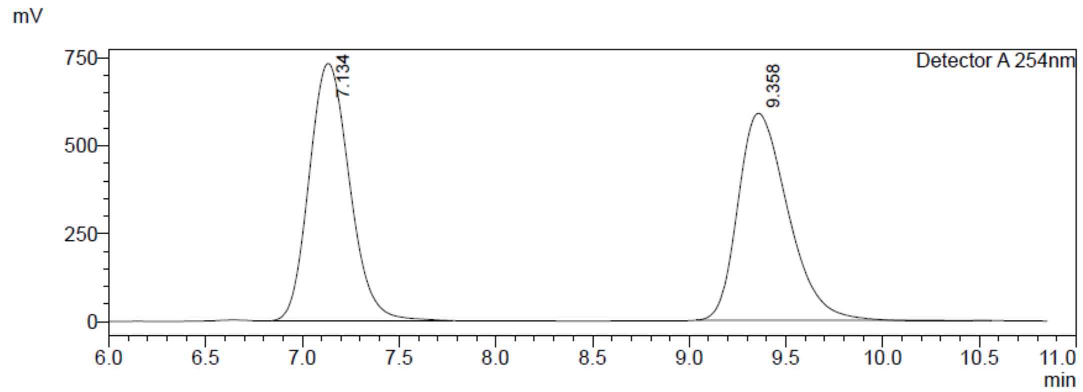


**<Peak Table>**

Detector A 254nm

| Peak# | Ret. Time | Height | Height% | Conc. | Area | Area% |
|---|---|---|---|---|---|---|
| 1 | 7.134 | 730057 | 55.394 | 50.161 | 10584138 | 50.161 |
| 2 | 9.358 | 587881 | 44.606 | 49.839 | 10516021 | 49.839 |
| Total | | 1317938 | 100.000 | | 21100159 | 100.000 |

Racemic **4y**

H:\[3.3.1] bridge bicycle\Scope of induoxiting\hetrocycle and alkyl\n-propyl\TJP-R152-13.lcd

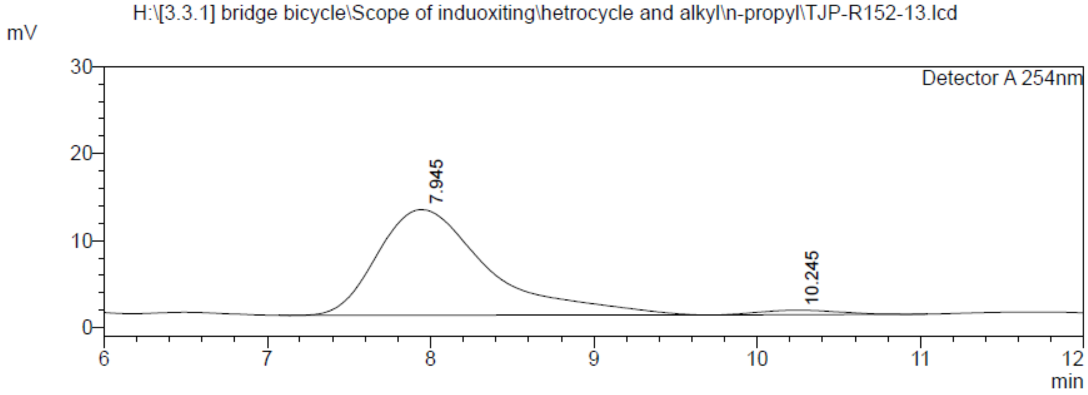


Detector A 254nm

| Peak# | Ret. Time | Height | Height% | Area | Area% |
|---|---|---|---|---|---|
| 1 | 7.945 | 12138 | 96.048 | 573635 | 97.218 |
| 2 | 10.245 | 499 | 3.952 | 16417 | 2.782 |
| Total | | 12638 | 100.000 | 590052 | 100.000 |

Enantiomerically enriched **4y**

**Methyl(6S,7S,13R)-8,13-dimethyl-7-(3-methyl-2-oxobutyl)-5,7,8,13-tetrahydro-6H-6,13-epiminobenzo[4,5]cycloocta[1,2-b]indole-6-carboxylate(4z)**

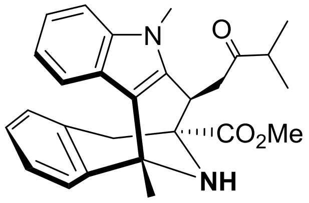


**4z**

A white solid; 33.5 mg, 78% yield; m.p. =185.5-186.9 $^{o}$C, $[\alpha]^{25}_{D}$ = -22.54 (*c* 0.22, $CHCl_3$); *dr* >20:1; $^{1}$H NMR (400 MHz, $CDCl_3$) δ 7.66 (d, *J* = 6.8 Hz, 1H), 7.60 (d, *J* = 7.6 Hz, 1H), 7.22 (d, *J* = 8.4 Hz, 1H), 7.18 (t, *J* = 7.6 Hz, 1H), 7.14 – 7.10 (m, 1H),

7.04 (td, $J$ = 7.8, 1.2 Hz, 2H), 6.92 (d, $J$ = 7.2 Hz, 1H), 4.02 (dd, $J$ = 5.6, 3.6 Hz, 1H), 3.76 (s, 3H), 3.69 (s, 1H), 3.60 (s, 3H), 3.26 (dd, $J$ = 18.8, 5.6 Hz, 1H), 3.25 (d, $J$ = 17.0 Hz, 1H), 2.71(dd, $J$ = 18.8, 3.6 Hz, 1H), 2.67 (d, $J$ = 17.0 Hz), 2.55 (dt, $J$ = 13.6, 6.8 Hz, 1H), 2.11 (s, 3H), 1.09 (dd, $J$ = 9.8, 6.8 Hz, 6H); $^{13}$C NMR (100 MHz, $CDCl_3$) δ 212.47, 175.14, 145.87, 137.82, 136.56, 133.79, 129.03, 126.30, 126.17, 124.20, 121.70, 121.16, 119.34, 119.29, 112.14, 109.29, 61.79, 53.77, 52.59, 43.73, 41.38, 38.54, 37.44, 29.43, 25.05, 18.50, 18.19; HRMS (ESI) *m/z* calcd for $C_{27}H_{30}N_2O_3$ $[M+H]^+$ = 431.2329, found = 431.2332; The ee value was 96%, $t_R$ (major) = 7.6 min, $t_R$ (minor) = 11.1 min (Chiralcel IA, λ = 254 nm, 10% i-PrOH/hexane, flow rate = 1.0 mL/min).

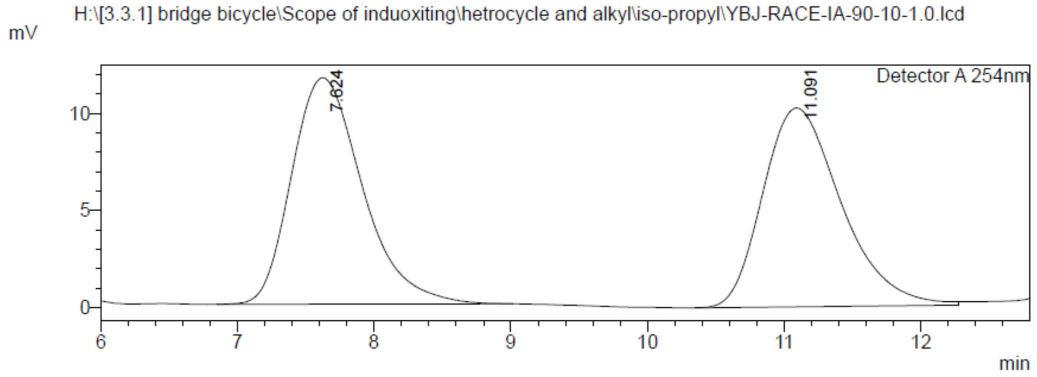


Detector A 254nm

| Peak# | Ret. Time | Height | Height% | Area | Area% |
|---|---|---|---|---|---|
| 1 | 7.624 | 11641 | 53.257 | 419717 | 50.746 |
| 2 | 11.091 | 10218 | 46.743 | 407370 | 49.254 |
| Total | | 21859 | 100.000 | 827087 | 100.000 |

Racemic **4z**

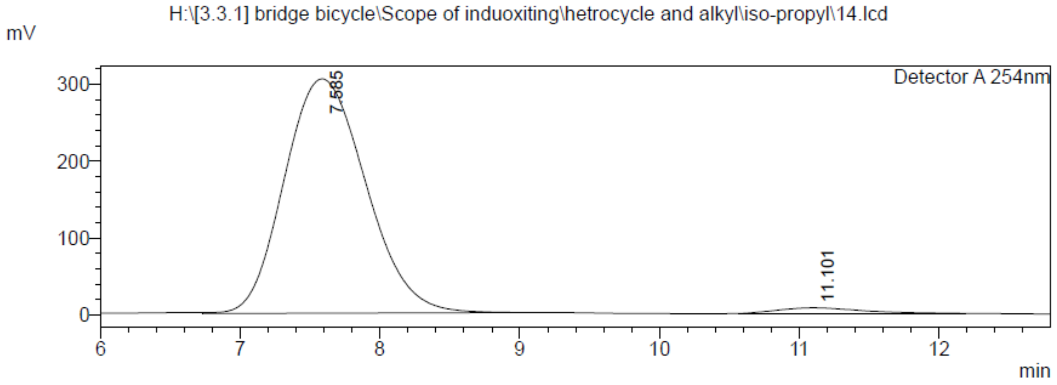


Detector A 254nm

| Peak# | Ret. Time | Height | Height% | Area | Area% |
|---|---|---|---|---|---|
| 1 | 7.585 | 304746 | 97.722 | 12347896 | 97.684 |
| 2 | 11.101 | 7103 | 2.278 | 292742 | 2.316 |
| Total | | 311849 | 100.000 | 12640639 | 100.000 |

Enantiomerically enriched **4z**

**Methyl(6S,7S,13R)-7-(2-((1R,3S)-adamantan-1-yl)-2-oxoethyl)-8,13-dimethyl-5,7,8,13-tetrahydro-6H-6,13-epiminobenzo[4,5]cycloocta[1,2-b]indole-6-carboxylate (4aa)**

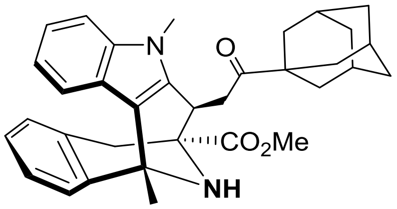


**4aa**

A white solid; 38.6 mg, 74% yield; m.p. =101.5-102.3 $^{o}$C, $[\alpha]^{25}_{D}$ = -32.54 (*c* 0.21, $CHCl_3$); *dr* >20:1; $^1$H NMR (400 MHz, $CDCl_3$) δ 7.66 (d, *J* = 6.8 Hz, 1H), 7.61 (d, *J* = 8.0 Hz, 1H), 7.19 (dd, *J* = 16.6, 8.0 Hz, 2H), 7.14 – 7.10 (m, 1H), 7.07 – 7.01 (m, 2H), 6.93 (d, *J* = 7.6 Hz, 1H), 4.10 (dd, *J* = 5.2, 3.2 Hz, 1H), 3.75 (s, 3H), 3.56 (s, 3H), 3.30 (dd, *J* = 19.6, 5.2 Hz, 2H), 3.28 (d, *J* = 17.6 Hz, 1H), 2.70 (d, *J* = 17.6 Hz, 1H), 2.68 (dd, *J* = 19.6, 3.2 Hz, 1H)., 2.13 (s, 3H), 2.03 (s, 3H), 1.80 (d, *J* = 2.4 Hz, 6H), 1.71 (q, *J* = 12.4 Hz, 6H); $^{13}$C NMR (100 MHz, $CDCl_3$) δ 213.05, 175.12, 145.91, 137.75, 137.13, 133.90, 129.01, 126.25, 126.11, 124.21, 121.66, 121.07, 119.29, 119.26, 112.00, 109.25, 61.88, 53.76, 52.62, 46.46, 39.97, 38.56, 37.65, 37.53, 36.61, 29.46, 28.09, 25.06; HRMS (ESI) *m/z* calcd for $C_{34}H_{38}N_2O_3$ $[M+H]^+$ = 523.2955, found = 523.2953; The ee value was 72%, $t_R$ (major) = 9.2 min, $t_R$ (minor) = 18.4 min (Chiralcel IA, λ = 254 nm, 10% i-PrOH/hexane, flow rate = 1.0 mL/min).

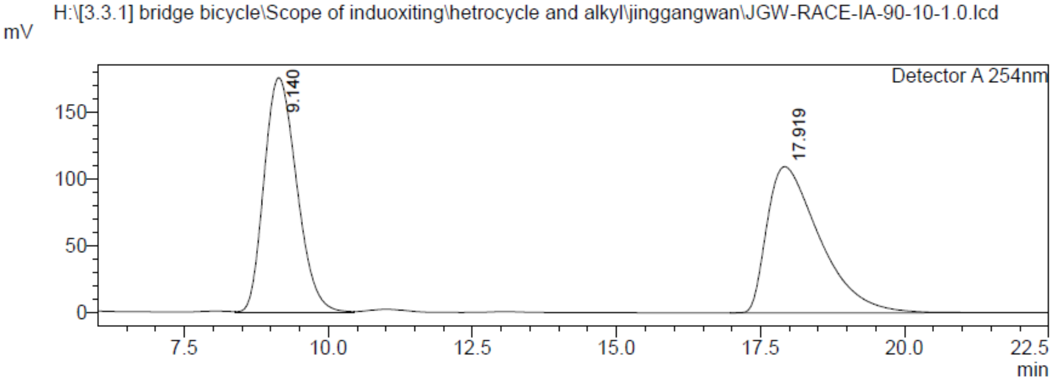


Detector A 254nm

| Peak# | Ret. Time | Height | Height% | Area | Area% |
|---|---|---|---|---|---|
| 1 | 9.140 | 175689 | 61.659 | 6964599 | 49.977 |
| 2 | 17.919 | 109247 | 38.341 | 6970926 | 50.023 |
| Total | | 284936 | 100.000 | 13935525 | 100.000 |

Racemic **4aa**

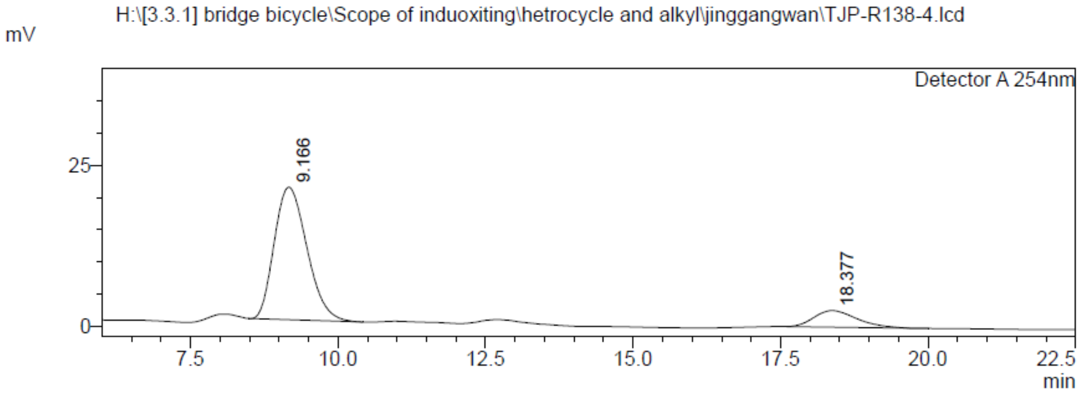


Detector A 254nm

| Peak# | Ret. Time | Height | Height% | Area | Area% |
|---|---|---|---|---|---|
| 1 | 9.166 | 20507 | 88.871 | 792006 | 85.764 |
| 2 | 18.377 | 2568 | 11.129 | 131463 | 14.236 |
| Total | | 23076 | 100.000 | 923469 | 100.000 |

Enantiomerically enriched **4aa**

**Methyl-(6S,7S,13R)-8-benzyl-13-methyl-7-(2-oxo-2-phenylethyl)-5,7,8,13-tetrahydro-6H-6,13-epiminobenzo[4,5]cycloocta[1,2-b]indole-6-carboxylate(4ab)**

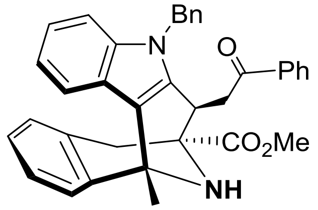


**4ab**

A white solid; 49.1 mg, 91% yield; m.p.=129.5-130.6 °C, $[\alpha]^{25}_{D}$ = -35.89 (*c* 0.35, $CHCl_3$); *dr* >20:1; $^1$H NMR (400 MHz, $CDCl_3$) δ 7.83 (d, *J* = 7.2 Hz, 1H), 7.72 (d, *J* = 7.4 Hz, 1H), 7.68 (dd, *J* = 6.2, 3.0 Hz, 1H), 7.52 (t, *J* = 7.4 Hz, 1H), 7.39 (t, *J* = 7.8 Hz, 2H), 7.22 (t, *J* = 7.6 Hz, 1H), 7.15 – 7.05 (m, 7H), 6.94 (d, *J* = 7.8 Hz, 1H), 6.85 (d, *J* = 7.0 Hz, 2H), 5.41 (dd, *J* = 24.0, 17.2 Hz, 2H), 4.16 (dd, *J* = 6.0, 2.4 Hz, 1H), 3.85 (dd, *J* = 18.4, 6.0 Hz, 1H), 3.43 (s, 3H), 3.22 (d, *J* = 17.6 Hz, 1H), 2.86 (dd, *J* = 18.4, 2.4 Hz, 1H), 2.63 (d, *J* = 17.6 Hz, 1H), 2.19 (s, 3H); HRMS (ESI) *m/z* calcd for $C_{36}H_{32}N_2O_3$ $[M+H]^+$ = 541.2486, found = 541.2485; The ee value was 90%, $t_R$ (major) =12.1 min, $t_R$ (minor) = 21.2 min (Chiralcel IA, λ = 254 nm, 10% i-PrOH/hexane, flow rate = 1.0 mL/min).

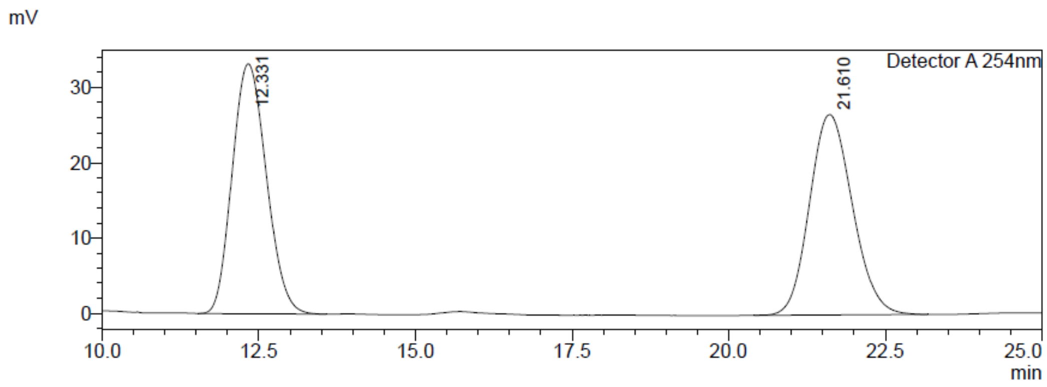


**<Peak Table>**

Detector A 254nm

| Peak# | Ret. Time | Height | Height% | Conc. | Area | Area% |
|---|---|---|---|---|---|---|
| 1 | 12.331 | 33194 | 55.503 | 49.858 | 1261441 | 49.858 |
| 2 | 21.610 | 26611 | 44.497 | 50.142 | 1268606 | 50.142 |
| Total | | 59805 | 100.000 | | 2530047 | 100.000 |

Racemic **4ab**

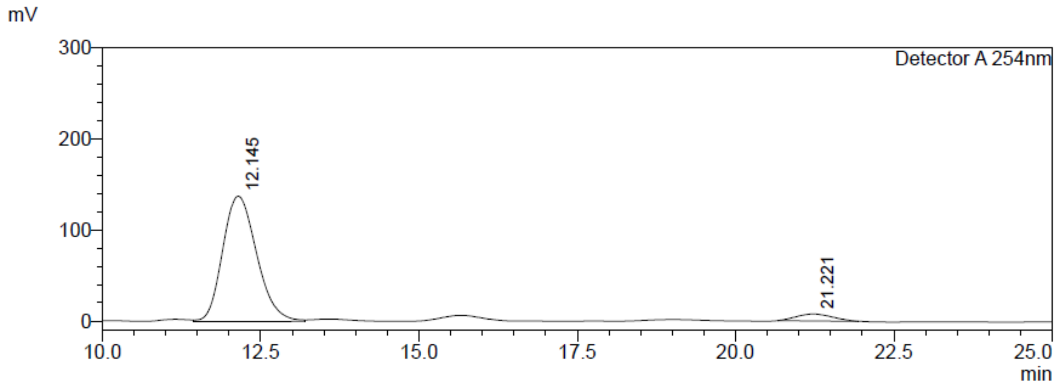


**<Peak Table>**

Detector A 254nm

| Peak# | Ret. Time | Height | Height% | Conc. | Area | Area% |
|---|---|---|---|---|---|---|
| 1 | 12.145 | 137352 | 94.841 | 94.561 | 5141747 | 94.561 |
| 2 | 21.221 | 7472 | 5.159 | 5.439 | 295736 | 5.439 |
| Total | | 144824 | 100.000 | | 5437483 | 100.000 |

Enantiomerically enriched **4ab**

**Methyl(6S,7S,13R)-8-(4-methoxybenzyl)-13-methyl-7-(2-oxo-2-phenylethyl)-5,7,8,13-tetrahydro-6H-6,13-epiminobenzo[4,5]cycloocta[1,2-b]indole-6-carboxylate(4ac)**

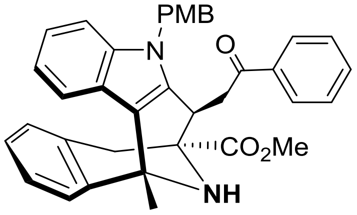


**4ac**

A white solid; 52.4 mg, 92% yield; m.p. =146.6-148.3 $^{o}$C, $[\alpha]^{25}_{D}$ = -22.14 (*c* 0.25, $CHCl_3$); *dr* >20:1; $^{1}$H NMR (400 MHz, $CDCl_3$) δ 7.83 (d, *J* = 7.2 Hz, 2H), 7.71 (d, *J* = 7.2 Hz, 1H), 7.67 (dd, *J* = 6.4, 2.4 Hz, 1H), 7.52 (t, *J* = 7.2 Hz, 1H), 7.39 (t, *J* = 7.6 Hz, 2H), 7.22 (t, *J* = 7.6 Hz, 1H), 7.17 (dd, *J* = 6.6, 2.2 Hz, 1H), 7.09 – 7.03 (m, 3H), 6.94 (d, *J* = 7.6 Hz, 1H), 6.80 (d, *J* = 8.8 Hz, 2H), 6.63 (d, *J* = 8.8 Hz, 2H), 5.33 (dd, *J* = 16.8, 26.4 Hz, 2H), 4.17 (dd, *J* = 6.0, 2.8 Hz, 1H), 3.81 (dd, *J* = 18.4, 6.0 Hz, 1H), 3.62 (s, 3H), 3.45 (s, 3H), 3.23 (d, *J* = 18.0 Hz, 1H), 2.86 (dd, *J* = 18.4, 2.8 Hz, 1H), 2.63 (d, *J* = 18.0 Hz, 1H), 2.18 (s, 3H), 1.59 (s, 1H); $^{13}$C NMR (100 MHz, $CDCl_3$) δ 198.25, 175.02, 158.72, 145.91, 137.69, 136.75, 136.42, 133.92, 133.22, 129.78, 129.05, 128.59, 128.24, 127.31, 126.33, 126.20, 124.37, 121.83, 121.43, 119.51, 119.41, 114.12, 113.07, 110.04, 61.81, 55.22, 53.83, 52.38, 45.62, 41.34, 39.40, 37.22, 25.11; HRMS (ESI) *m/z* calcd for $C_{37}H_{34}N_2O_4$ $[M+H]^+$ = 571.2591, found = 571.2590; The ee value was 92%, $t_R$ (major) = 90.2 min, $t_R$ (minor) = 106.0 min (Chiralcel IE, λ = 254 nm, 5% i-PrOH/hexane, flow rate = 1.0 mL/min).

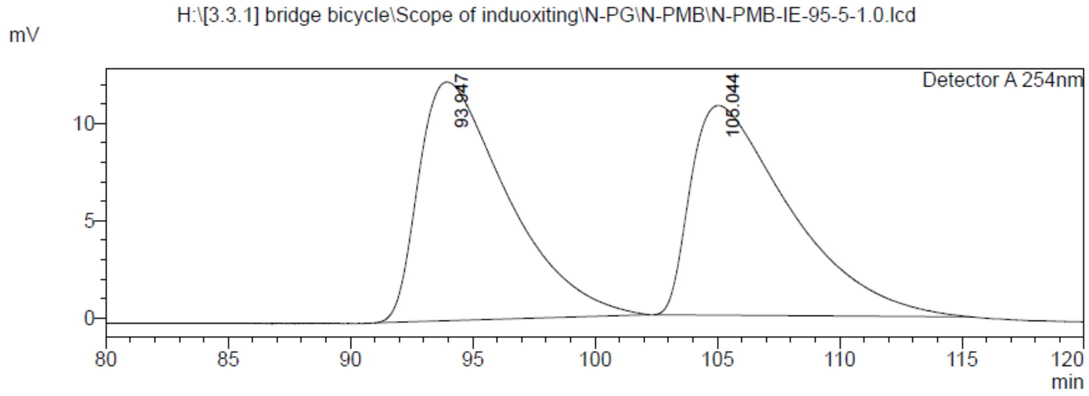


Detector A 254nm

| Peak# | Ret. Time | Height | Height% | Area | Area% |
|---|---|---|---|---|---|
| 1 | 93.947 | 12250 | 53.254 | 3095873 | 50.418 |
| 2 | 105.044 | 10753 | 46.746 | 3044551 | 49.582 |
| Total | | 23002 | 100.000 | 6140424 | 100.000 |

Racemic **4ac**

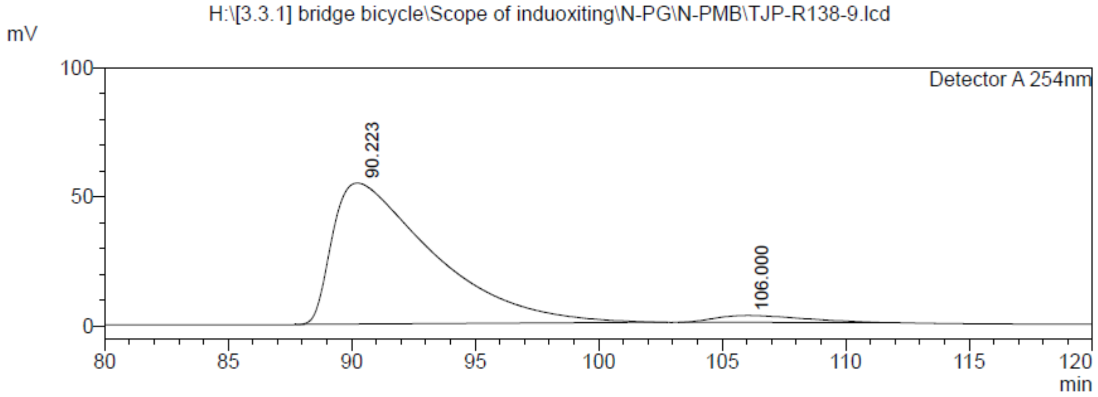


Detector A 254nm

| Peak# | Ret. Time | Height | Height% | Area | Area% |
|---|---|---|---|---|---|
| 1 | 90.223 | 54666 | 95.312 | 15513585 | 95.764 |
| 2 | 106.000 | 2689 | 4.688 | 686146 | 4.236 |
| Total | | 57355 | 100.000 | 16199732 | 100.000 |

Enantiomerically enriched **4ac**

**Methyl(6S,7S,13R)-12-fluoro-8,13-dimethyl-7-(2-oxo-2-phenylethyl)-5,7,8,13-tetrahydro-6H-6,13-epiminobenzo[4,5]cycloocta[1,2-b]indole-6-carboxylate(4ad)**

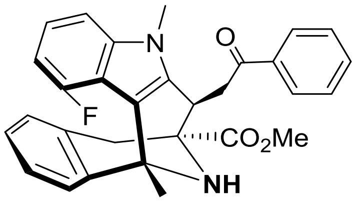


**4ad**

A white solid; 43.8 mg, 91% yield; m.p. =183.6-184.8 °C, $[\alpha]^{25}_{D}$ = -32.35 (*c* 0.41, $CHCl_3$); *dr* >20:1; $^1$H NMR (400 MHz, $CDCl_3$) δ 8.01 (d, *J* = 7.2 Hz, 2H), 7.74 (dd, *J* = 7.6, 3.2 Hz, 1H), 7.57 (t, *J* = 7.2 Hz, 1H), 7.46 (t, *J* = 7.6 Hz, 2H), 7.20 (t, *J* = 7.6 Hz, 1H), 7.07 (t, *J* = 7.4 Hz, 1H), 7.05 – 6.99 (m, 2H), 6.98 (d, *J* = 8.0 Hz, 1H), 6.94 (d, *J* = 7.4 Hz, 1H), 6.70 (ddd, *J* = 11.8, 7.6, 0.8 Hz, 1H), 4.20 (dd, *J* = 6.0, 2.8 Hz, 1H), 4.00 (dd, *J* = 18.0, 6.0 Hz, 1H), 3.68 (s, 3H), 3.48 (s, 3H), 3.27 (d, *J* = 17.6 Hz, 1H), 2.98 (dd, *J* = 18.0, 2.8 Hz, 1H), 2.70 (d, *J* = 17.6 Hz, 1H), 2.19 (s, 3H); $^{13}$C NMR (100 MHz, $CDCl_3$) δ 198.64, 174.94, 155.85 (d, *J* = 246.1 Hz), 146.01, 140.78, 140.65, 136.77, 136.69 , 133.63, 133.57, 128.85, 128.69, 128.43, 126.21 (d, *J* = 12.8 Hz), 123.20 (d, *J* = 4.2 Hz), 121.76 (d, *J* = 8.4 Hz), 112.97, 112.06, 105.28, 105.07, 61.70, 53.82, 52.43, 39.89, 37.36, 29.90, 26.15 (d, *J* = 10.8 Hz); $^{19}$F NMR (376 MHz, CDCl3) δ -113.82; HRMS (ESI) *m/z* calcd for $C_{30}H_{27}FN_2O_3$ $[M+H]^+$ = 483.2078,

found = 483.2079; The ee value was 90%, $t_R$ (major) = 9.6 min, $t_R$ (minor) = 12.3 min (Chiralcel IE, λ = 254 nm, 5% i-PrOH/hexane, flow rate = 1.0 mL/min).

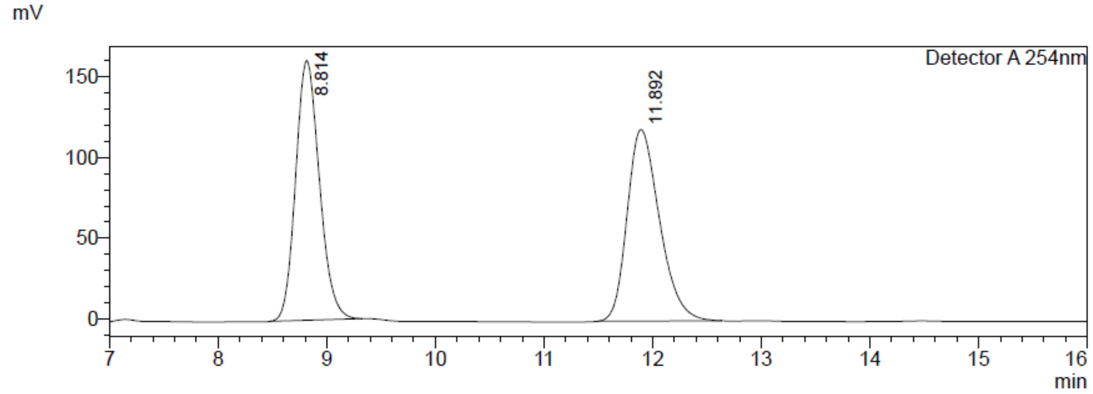


<Peak Table>

Detector A 254nm

| Peak# | Ret. Time | Height | Height% | Conc. | Area | Area% |
|---|---|---|---|---|---|---|
| 1 | 8.814 | 160976 | 57.530 | 49.927 | 2456558 | 49.927 |
| 2 | 11.892 | 118837 | 42.470 | 50.073 | 2463729 | 50.073 |
| Total | | 279813 | 100.000 | | 4920287 | 100.000 |

Racemic **4ad**

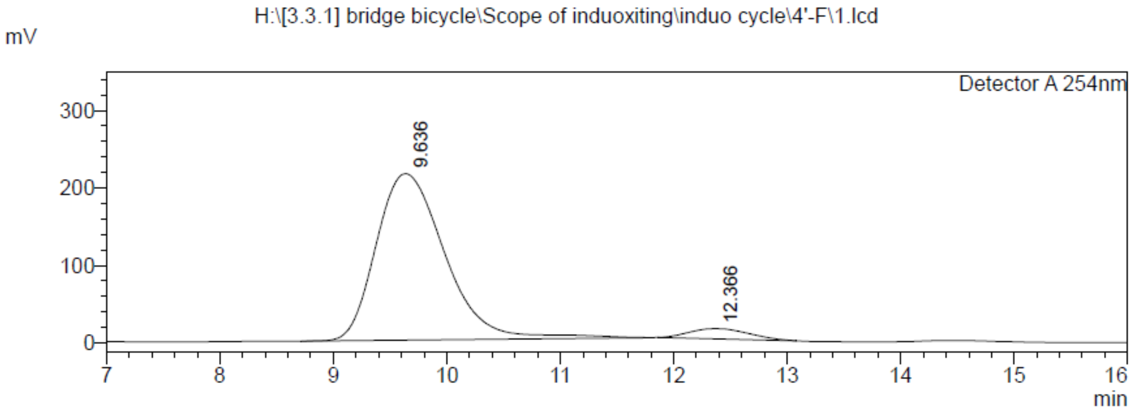


Detector A 254nm

| Peak# | Ret. Time | Height | Height% | Area | Area% |
|---|---|---|---|---|---|
| 1 | 9.636 | 215039 | 94.164 | 9074263 | 94.955 |
| 2 | 12.366 | 13328 | 5.836 | 482112 | 5.045 |
| Total | | 228368 | 100.000 | 9556375 | 100.000 |

Enantiomerically enriched **4ad**

**methyl(6S,7S,13R)-12-methoxy-8,13-dimethyl-7-(2-oxo-2-phenylethyl)-5,7,8,13-tetrahydro-6H-6,13-epiminobenzo[4,5]cycloocta[1,2-b]indole-6-carboxylate(4ae)**

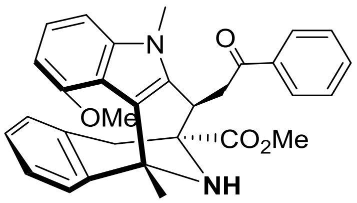

**4ae**

A white solid; 45.9 mg, 93% yield; m.p. =85.6-86.9 °C, $[\alpha]^{25}_{D}$ = -26.12 (*c* 0.24, $CHCl_3$); *dr* >20:1; $^1$H NMR (400 MHz, $CDCl_3$) δ 8.01 (d, *J* = 7.2 Hz, 2H), 7.71 (d, *J* = 7.6 Hz, 1H), 7.56 (t, *J* = 7.2 Hz, 1H), 7.46 (t, *J* = 8.0 Hz, 2H), 7.17 (t, *J* = 7.6 Hz, 1H), 7.04 (t, *J* = 8.0 Hz, 2H), 6.92 (d, *J* = 8.4 Hz, 1H), 6.83 (d, *J* = 8.0 Hz, 1H), 6.46 (d, *J* = 7.8 Hz, 1H), 4.18 (dd, *J* = 6.4, 2.4 Hz, 1H), 4.02 (dd, *J* = 18.0, 6.4 Hz, 1H), 3.92 (s, 3H), 3.66 (s, 3H), 3.46 (s, 3H), 3.24 (d, *J* = 17.6 Hz, 1H), 2.99 (dd, *J* = 18.0, 2.4 Hz, 1H), 2.70 (d, *J* = 17.6 Hz, 1H), 2.25 (s, 3H); $^{13}$C NMR (100 MHz, $CDCl_3$) δ 198.85, 175.16, 153.35, 146.82, 139.57, 136.89, 135.40, 133.99, 133.47, 128.81, 128.58, 128.43, 125.94, 125.42, 123.91, 122.09, 115.01, 112.95, 102.47, 100.19, 61.70, 54.83, 54.48, 52.33, 40.90, 39.97, 37.44, 29.69, 26.91; HRMS (ESI) *m/z* calcd for $C_{31}H_{30}N_2O_4$ $[M+H]^+$ = 495.2278, found = 495.2277; The ee value was 92%, $t_R$ (major) = 8.9 min, $t_R$ (minor) = 11.8 min (Chiralcel IA, λ = 254 nm, 10% i-PrOH/hexane, flow rate = 1.0 mL/min).

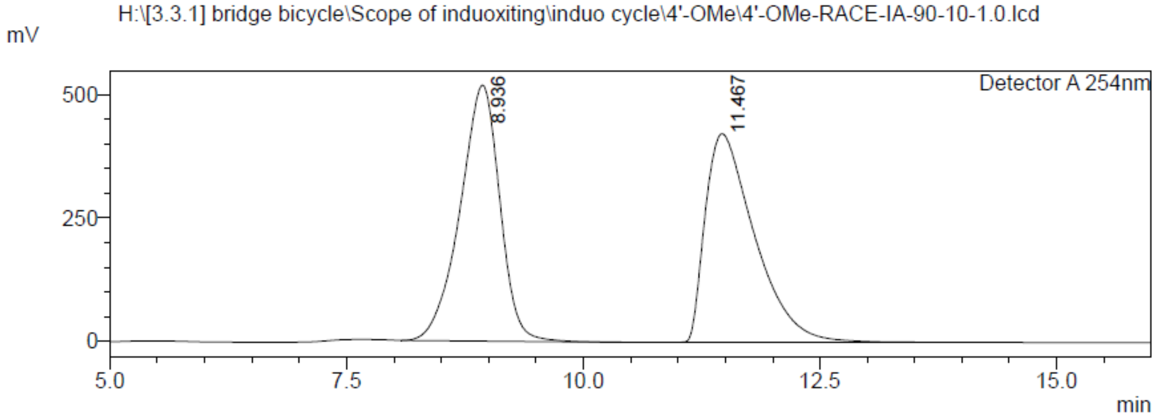


Detector A 254nm

| Peak# | Ret. Time | Height | Height% | Area | Area% |
|---|---|---|---|---|---|
| 1 | 8.936 | 518939 | 55.081 | 15208074 | 49.696 |
| 2 | 11.467 | 423198 | 44.919 | 15393901 | 50.304 |
| Total | | 942138 | 100.000 | 30601975 | 100.000 |

Racemic **4ae**

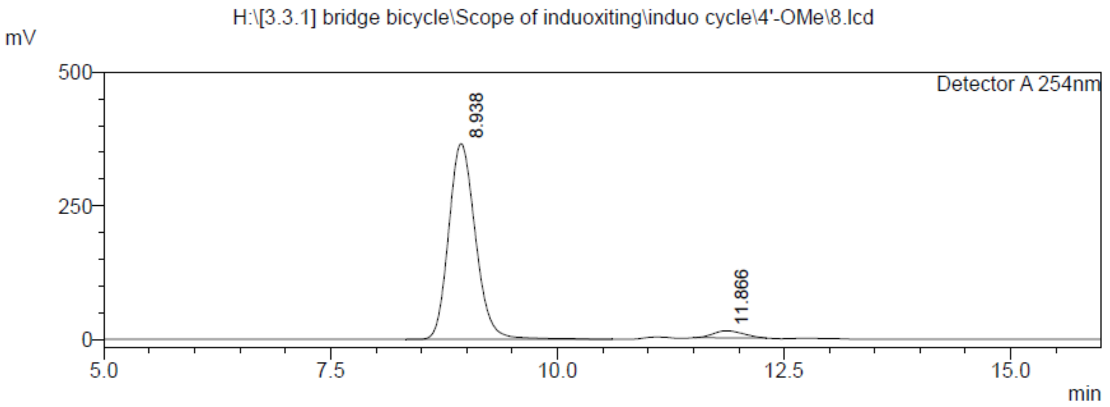


Detector A 254nm

| Peak# | Ret. Time | Height | Height% | Area | Area% |
|---|---|---|---|---|---|
| 1 | 8.938 | 366051 | 96.505 | 7544218 | 96.060 |
| 2 | 11.866 | 13255 | 3.495 | 309462 | 3.940 |
| Total | | 379306 | 100.000 | 7853680 | 100.000 |

Enantiomerically enriched **4ae**

**Methyl(6S,7S,13R)-11-chloro-8,13-dimethyl-7-(2-oxo-2-phenylethyl)-5,7,8,13-tetrahydro-6H-6,13-epiminobenzo[4,5]cycloocta[1,2-b]indole-6-carboxylate(4af)**

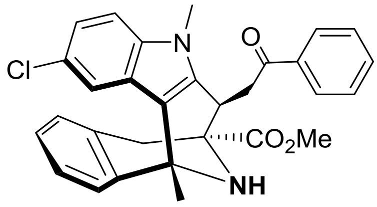


**4af**

A white solid; 45.8 mg, 92% yield; m.p. =200.5-201.4 $^{o}$C, $[\alpha]^{25}_{D}$ = -25.00 (*c* 0.42, $CHCl_3$); *dr* >20:1; $^{1}$H NMR (400 MHz, $CDCl_3$) δ 7.99 (d, *J* = 7.2 Hz, 2H), 7.67 (d, *J* = 7.6 Hz, 1H), 7.58 – 7.54 (m, 2H), 7.46 (t, *J* = 7.2 Hz, 2H), 7.23 (t, *J* = 7.6 Hz, 1H), 7.11 –7.04 (m, 3H), 6.95 (d, *J* = 7.2 Hz, 1H), 4.20 (dd, *J* = 6.0, 3.2 Hz, 1H), 3.93 (dd, *J* = 18.0, 6.0 Hz, 1H), 3.66 (s, 3H), 3.49 (s, 3H), 3.27 (d, *J* = 18.0 Hz, 1H), 2.99 (dd, *J* = 18.0, 3.2 Hz, 1H), 2.70 (d, *J* = 18.0 Hz, 1H), 2.33 (s, 1H), 2.12 (s, 3H); $^{13}$C NMR (100 MHz, $CDCl_3$) δ 198.55, 174.90, 145.52, 137.71, 136.73, 136.28, 133.60, 129.09, 128.85, 128.41, 126.48, 126.43, 125.06, 125.04, 121.75, 121.37, 118.61, 112.40, 110.23, 61.66, 53.60, 52.48, 41.00, 39.94, 37.35, 29.63, 25.01; HRMS (ESI) *m/z* calcd for $C_{30}H_{27}ClN_2O_3$ $[M+H]^+$ = 499.1783, found = 499.1782; The ee value was >99%, $t_R$ (major) = 10.7 min, $t_R$ (minor) = 28.4 min (Chiralcel IA, λ = 254 nm, 10% i-PrOH/hexane, flow rate = 1.0 mL/min).

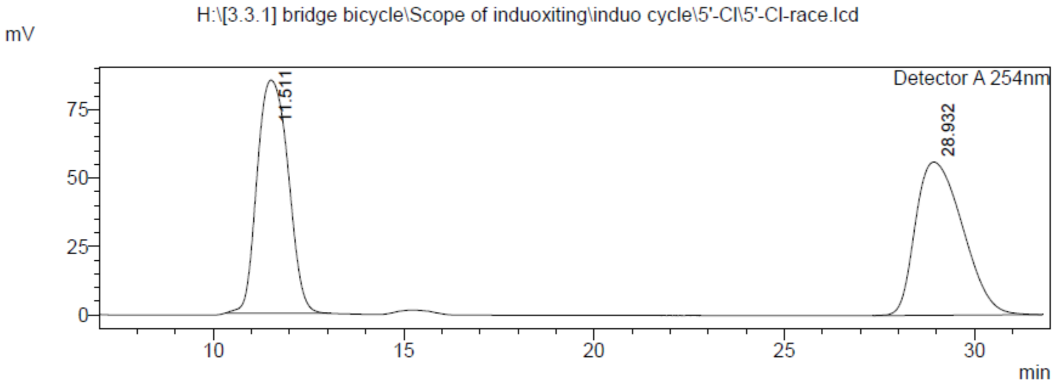


Detector A 254nm

| Peak# | Ret. Time | Height | Height% | Area | Area% |
|---|---|---|---|---|---|
| 1 | 11.511 | 85177 | 60.347 | 4848659 | 50.087 |
| 2 | 28.932 | 55968 | 39.653 | 4831859 | 49.913 |
| Total | | 141144 | 100.000 | 9680518 | 100.000 |

Racemic **4af**

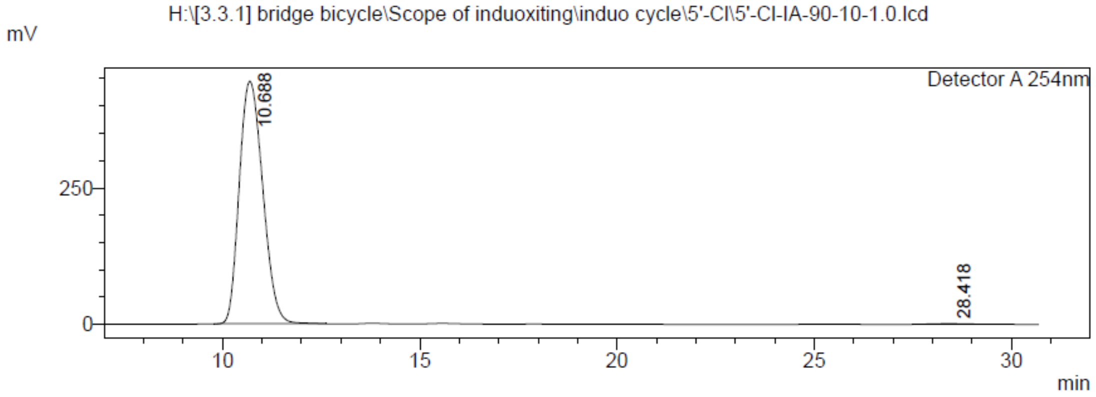


Detector A 254nm

| Peak# | Ret. Time | Height | Height% | Area | Area% |
|---|---|---|---|---|---|
| 1 | 10.688 | 443433 | 99.657 | 18371796 | 99.574 |
| 2 | 28.418 | 1525 | 0.343 | 78661 | 0.426 |
| Total | | 444957 | 100.000 | 18450457 | 100.000 |

Enantiomerically enriched **4af**

**Methyl(6S,7S,13R)-8,11,13-trimethyl-7-(2-oxo-2-phenylethyl)-5,7,8,13-tetrahydro-6H-6,13-epiminobenzo[4,5]cycloocta[1,2-b]indole-6-carboxylate(4ag)**

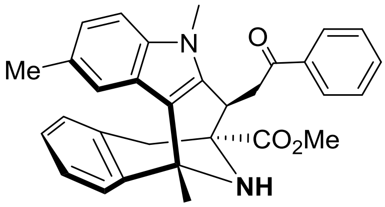


**4ag**

A white solid; 44.5 mg, 93% yield; m.p. =171.9-172.5 °C, $[\alpha]^{25}_{D}$ = -31.10 (*c* 0.24, $CHCl_3$); *dr* >20:1; $^1$H NMR (400 MHz, $CDCl_3$) δ 8.00 (d, *J* = 7.2 Hz, 2H), 7.70 (d, *J*

= 7.6 Hz, 1H), 7.57 (t, *J* = 7.6 Hz, 1H), 7.46 (t, *J* = 7.6 Hz, 2H), 7.39 (s, 1H), 7.22 (t, *J* = 7.6 Hz, 1H), 7.08 (dd, *J* = 18.2, 7.8 Hz, 2H), 6.95 (dd, *J* = 7.2, 4.6 Hz, 2H), 4.21 (dd, *J* = 6.0, 2.8 Hz, 1H), 3.95 (dd, *J* = 18.0, 6.0 Hz, 1H), 3.65 (s, 3H), 3.50 (s, 3H), 3.28 (d, *J* = 18.0 Hz, 1H), 3.00 (dd, *J* = 18.0, 2.8 Hz, 1H), 2.73 (d, *J* = 18.0 Hz, 1H), 2.43 (s, 3H), 2.35 (s, 1H), 2.16 (s, 3H); $^{13}$C NMR (100 MHz, $CDCl_3$) δ 198.70, 175.13, 145.98, 136.85, 136.30, 130.24, 133.79, 133.48, 129.03, 128.81, 128.50, 128.41, 126.25, 126.17, 124.33, 122.67, 121.78, 119.02, 111.88, 108.98, 61.74, 53.78, 52.41, 41.09, 39.85, 37.42, 29.46, 25.14, 21.73; HRMS (ESI) *m/z* calcd for $C_{30}H_{30}N_2O_3$ $[M+H]^+$ = 479.2329, found = 479.2327; The ee value was 94%, $t_R$ (major) = 9.6 min, $t_R$ (minor) = 13.1 min (Chiralcel IA, λ = 254 nm, 10% i-PrOH/hexane, flow rate = 1.0 mL/min).

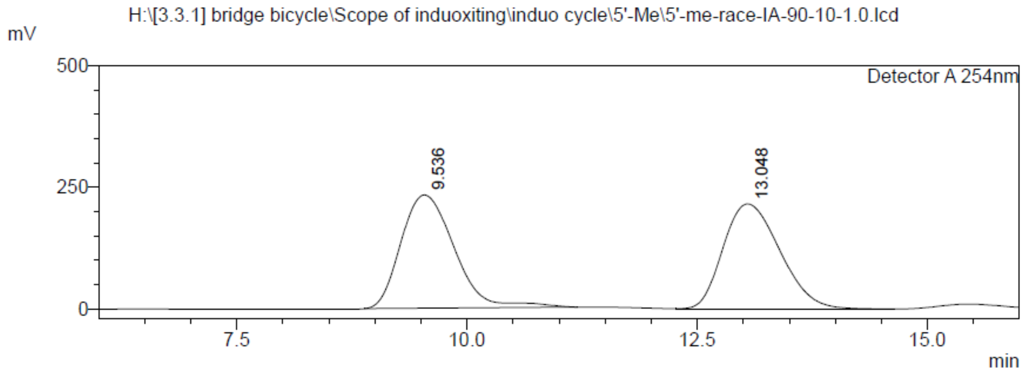


Detector A 254nm

| Peak# | Ret. Time | Height | Height% | Area | Area% |
|---|---|---|---|---|---|
| 1 | 9.536 | 232607 | 51.838 | 9397386 | 50.186 |
| 2 | 13.048 | 216116 | 48.162 | 9327595 | 49.814 |
| Total | | 448723 | 100.000 | 18724981 | 100.000 |

Racemic **4ag**

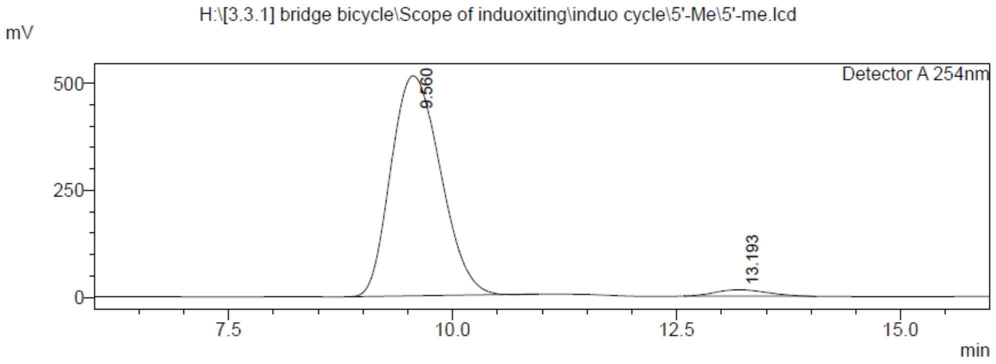


Detector A 254nm

| Peak# | Ret. Time | Height | Height% | Area | Area% |
|---|---|---|---|---|---|
| 1 | 9.560 | 515226 | 97.185 | 19861221 | 97.085 |
| 2 | 13.193 | 14923 | 2.815 | 596343 | 2.915 |
| Total | | 530149 | 100.000 | 20457564 | 100.000 |

Enantiomerically enriched **4ag**

**Methyl(6S,7S,13R)-8,10,13-trimethyl-7-(2-oxo-2-phenylethyl)-5,7,8,13-tetrahydro-6H-6,13-epiminobenzo[4,5]cycloocta[1,2-b]indole-6-carboxylate(4ah)**

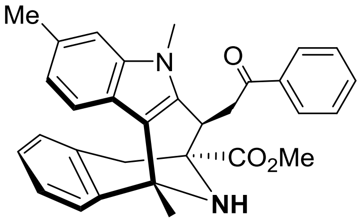


**4ah**

A white solid; 44.9 mg, 94% yield; m.p. =215.2-216.6 °C, $[\alpha]^{25}_{D}$ = -56.40 (*c* 0.25, $CHCl_3$); *dr* >20:1; $^{1}$H NMR (400 MHz, $CDCl_3$) δ 8.00 (d, *J* = 7.2 Hz, 2H), 7.67 (d, *J* = 7.2 Hz, 1H), 7.57 (t, *J* = 7.6 Hz, 1H), 7.50 (d, *J* = 8.0 Hz, 1H), 7.46 (t, *J* = 8.0 Hz, 2H), 7.18 (t, *J* = 7.6 Hz, 1H), 7.04 (t, *J* = 7.0 Hz, 1H), 7.01 (s, 1H), 6.93 (d, *J* = 7.6 Hz, 1H), 6.88 (d, *J* = 7.2 Hz, 1H), 4.19 (dd, *J* = 6.0, 2.8 Hz, 1H), 3.94 (dd, *J* = 18.0, 6.0 Hz, 1H), 3.65 (s, 3H), 3.49 (s, 3H), 3.27 (d, *J* = 17.6 Hz, 1H), 3.00 (dd, *J* = 18.0, 2.8 Hz, 1H), 2.72 (d, *J* = 17.6 Hz, 1H), 2.42 (s, 3H), 2.32 (s, 1H), 2.14 (s, 3H); $^{13}$C NMR (100 MHz, $CDCl_3$) δ 198.74, 175.14, 145.93, 138.24, 136.86, 135.56, 133.78, 133.49 , 131.10, 129.02, 128.82, 128.42, 126.25, 126.13, 121.95, 121.73, 120.94, 118.93, 112.40, 109.40, 61.79, 53.75, 52.41, 41.11, 39.83, 37.42, 29.38, 25.11, 21.83; HRMS (ESI) *m/z* calcd for $C_{31}H_{30}N_2O_3$ $[M+H]^+$ = 479.2329, found = 479.2330; The ee value was 96%, $t_R$ (major) = 10.6 min, $t_R$ (minor) = 15.5 min (Chiralcel IA, λ = 254 nm, 10% i-PrOH/hexane, flow rate = 1.0 mL/min).

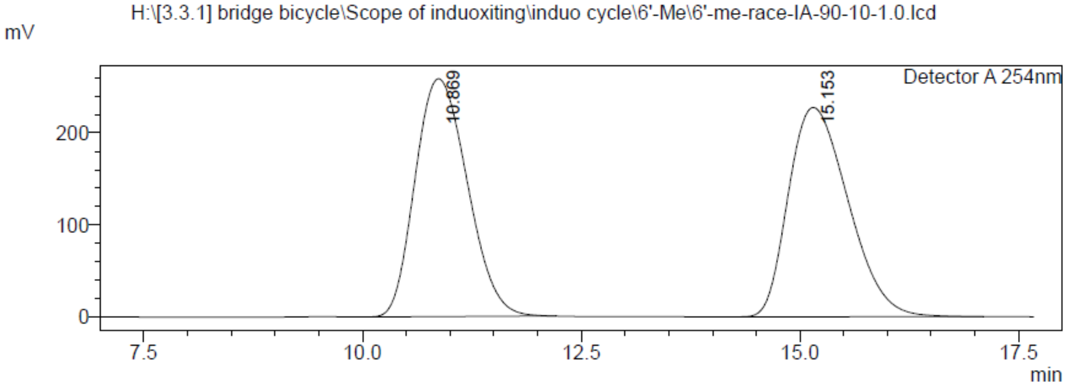


Detector A 254nm

| Peak# | Ret. Time | Height | Height% | Area | Area% |
|---|---|---|---|---|---|
| 1 | 10.869 | 257987 | 53.184 | 10733985 | 49.850 |
| 2 | 15.153 | 227098 | 46.816 | 10798481 | 50.150 |
| Total | | 485085 | 100.000 | 21532466 | 100.000 |

Racemic **4ah**

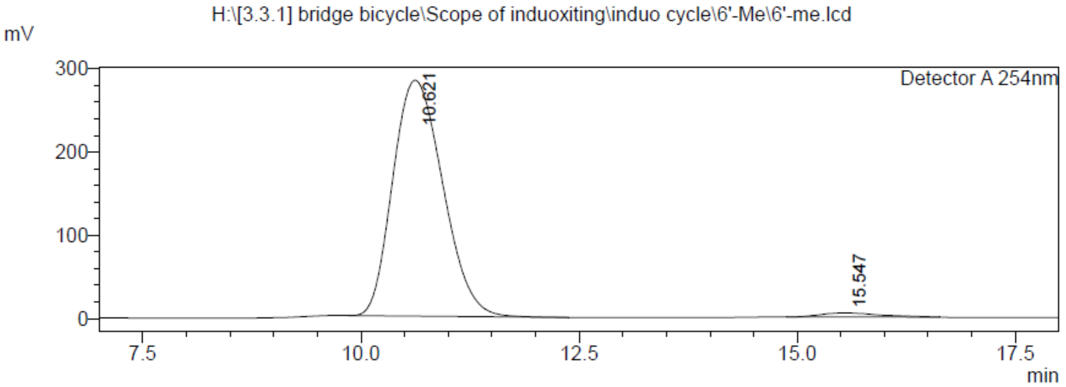


Detector A 254nm

| Peak# | Ret. Time | Height | Height% | Area | Area% |
|---|---|---|---|---|---|
| 1 | 10.621 | 283490 | 98.274 | 11326617 | 98.017 |
| 2 | 15.547 | 4980 | 1.726 | 229176 | 1.983 |
| Total | | 288470 | 100.000 | 11555794 | 100.000 |

Enantiomerically enriched **4ah**

**methyl(6S,7S,13R)-8,9,13-trimethyl-7-(2-oxo-2-phenylethyl)-5,7,8,13-tetrahydro-6H-6,13-epiminobenzo[4,5]cycloocta[1,2-b]indole-6-carboxylate(4ai)**

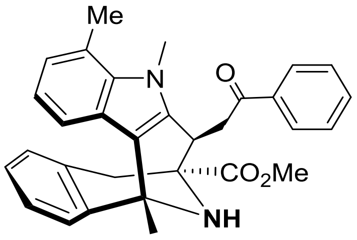


**4ai**

A white solid; 43.0 mg, 90% yield; m.p. =174.1-175.2 °C, $[\alpha]^{25}_{D}$ = -52.10 (*c* 0.35, $CHCl_3$); *dr* >20:1; $^{1}H$ NMR (400 MHz, $CDCl_3$) δ 8.01 (d, *J* = 7.2 Hz, 2H), 7.69 (d, *J* = 7.6 Hz, 1H), 7.57 (t, *J* = 7.2 Hz, 1H), 7.50 – 7.45 (m, 3H), 7.20 (t, *J* = 7.6 Hz, 1H), 7.06 (td, *J* = 7.6, 0.8 Hz, 1H), 6.94 (d, *J* = 7.6 Hz, 1H), 6.91 (t, *J* = 8.0 Hz, 1H), 6.82 (d, *J* = 7.2 Hz, 1H), 4.19 (dd, *J* = 6.4, 2.8 Hz, 1H), 4.00 (dd, *J* = 18.0, 6.4 Hz, 1H), 3.96 (s, 3H), 3.49 (s, 3H), 3.28 (d, *J* = 18.0 Hz, 1H), 2.98 (dd, *J* = 18.0, 2.8 Hz, 1H), 2.72 (d, *J* = 18.0 Hz, 1H), 2.70 (s, 3H), 2.31 (s, 1H), 2.14 (s, 3H); $^{13}C$ NMR (100 MHz, $CDCl_3$) δ 198.66, 175.09, 145.79, 136.82, 136.69, 136.64, 133.80, 133.49, 129.03, 128.81, 128.41, 126.26, 126.12, 124.98, 124.30, 121.83, 121.36, 119.41, 117.39, 112.50, 61.70, 53.69, 52.39, 40.96, 39.89, 37.43, 32.61, 25.00, 20.46; HRMS (ESI) *m/z* calcd for $C_{31}H_{30}N_2O_3$ $[M+H]^+$ = 479.2329, found = 479.2328; The ee value was 94%, $t_R$ (major) = 10.5 min, $t_R$ (minor) = 13.5 min (Chiralcel IA, λ = 254 nm, 10% i-PrOH/hexane, flow rate = 1.0 mL/min).

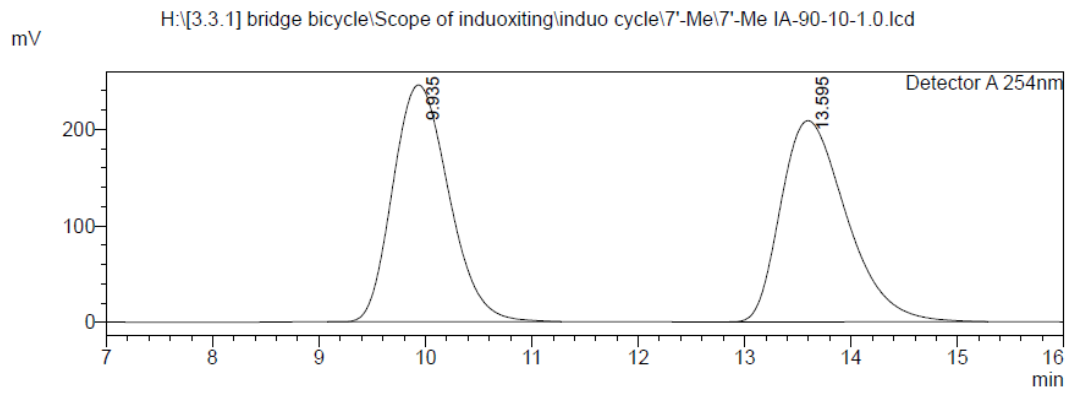


Detector A 254nm

| Peak# | Ret. Time | Height | Height% | Area | Area% |
|---|---|---|---|---|---|
| 1 | 9.935 | 245254 | 54.064 | 8950079 | 50.009 |
| 2 | 13.595 | 208381 | 45.936 | 8946896 | 49.991 |
| Total | | 453635 | 100.000 | 17896975 | 100.000 |

Racemic **4ai**

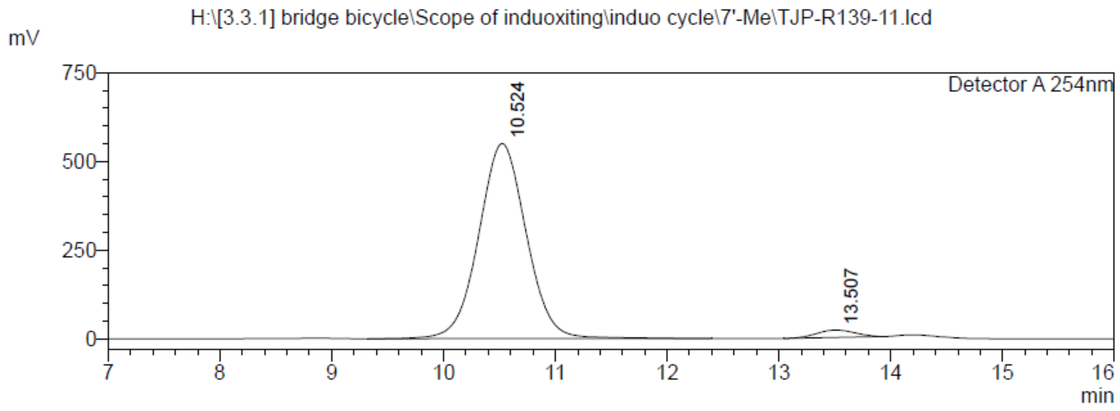


Detector A 254nm

| Peak# | Ret. Time | Height | Height% | Area | Area% |
|---|---|---|---|---|---|
| 1 | 10.524 | 549770 | 96.354 | 16288955 | 97.057 |
| 2 | 13.507 | 20802 | 3.646 | 493838 | 2.943 |
| Total | | 570572 | 100.000 | 16782793 | 100.000 |

Enantiomerically enriched **4ai**

**Methyl(6S,7S,13R)-4-chloro-8,13-dimethyl-7-(2-oxo-2-phenylethyl)-5,7,8,13-tetrahydro-6H-6,13-epiminobenzo[4,5]cycloocta[1,2-b]indole-6-carboxylate(5a)**

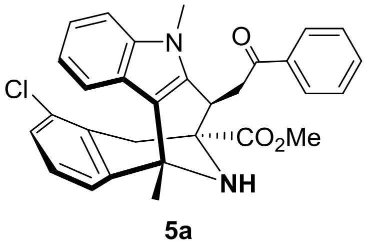


**5a**

A white solid; 46.3 mg, 93% yield; m.p. =195.8-197.2 $^{o}$C, $[\alpha]^{25}_{D}$ = -20,02 (*c* 0.75,

$CHCl_3$); *dr* >20:1; $^1$H NMR (400 MHz, $CDCl_3$) δ 7.99 (d, *J* = 7.2 Hz, 2H),7.61 – 7.55 (m, 3H), 7.46 (t, *J* = 7.6 Hz, 2H), 7.22 (d, *J* = 8.0 Hz, 1H), 7.16 – 7.11 (m, 3H), 7.04 (t, *J* = 7.6 Hz, 1H), 4.26 (dd, *J* = 6.4, 3.2 Hz, 1H), 3.93 (dd, *J* = 18.0, 6.4 Hz, 1H), 3.69 (s, 3H), 3.58 (d, *J* = 18.8 Hz, 1H), 3.52 (s, 3H), 2.97 (dd, *J* = 18.0, 3.2 Hz, 1H), 2.53 (d, *J* = 18.8 Hz, 1H), 2.17 (s, 3H); $^{13}$C NMR (100 MHz, $CDCl_3$) δ 198.34, 174.92, 147.66, 137.82, 136.77, 136.11, 134.24, 133.57, 131.76, 128.86, 128.40, 127.05, 126.71, 123.90, 121.34, 120.16, 119.43, 119.14, 112.77, 109.40, 61.37, 53.74, 52.59, 41.21, 39.66, 34.74, 29.47, 25.07; HRMS (ESI) *m/z* calcd for $C_{30}H_{27}ClN_2O_3$ $[M+H]^+$ = 499.1973, found = 499.1978; The ee value was 82%, $t_R$ (major) = 15.4 min, $t_R$ (minor) = 14.1 min (Chiralcel IA, λ = 254 nm, 5% i-PrOH/hexane, flow rate = 1.0 mL/min).

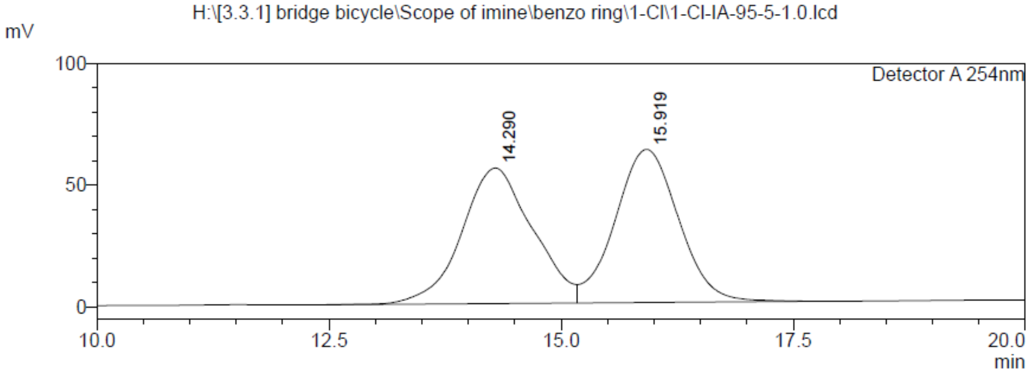


Detector A 254nm

| Peak# | Ret. Time | Height | Height% | Area | Area% |
|---|---|---|---|---|---|
| 1 | 14.290 | 55663 | 46.949 | 2952599 | 49.623 |
| 2 | 15.919 | 62899 | 53.051 | 2997473 | 50.377 |
| Total | | 118562 | 100.000 | 5950072 | 100.000 |

Racemic **5a**

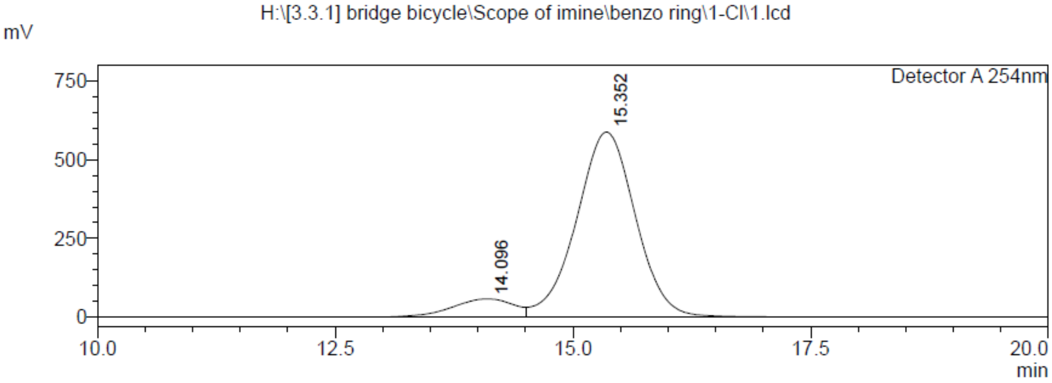


Detector A 254nm

| Peak# | Ret. Time | Height | Height% | Area | Area% |
|---|---|---|---|---|---|
| 1 | 14.096 | 57741 | 8.958 | 2594792 | 9.269 |
| 2 | 15.352 | 586865 | 91.042 | 25400626 | 90.731 |
| Total | | 644606 | 100.000 | 27995419 | 100.000 |

Enantiomerically enriched **5a**

**Methyl(6S,7S,13R)-4,8,13-trimethyl-7-(2-oxo-2-phenylethyl)-5,7,8,13-tetrahydro-6H-6,13-epiminobenzo[4,5]cycloocta[1,2-b]indole-6-carboxylate (5b)**

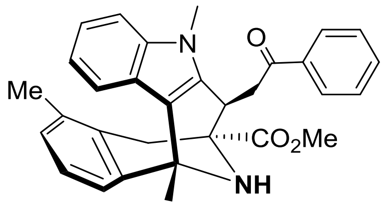


**5b**

A white solid; 43.9 mg, 92% yield; m.p. =74.2-74.3 °C, $[\alpha]^{25}_{D}$ = -19,05 (*c* 0.72, $CHCl_3$); *dr* >20:1; $^1H$ NMR (400 MHz, $CDCl_3$) δ 7.99 (d, *J* = 7.2 Hz, 2H), 7.61 (d, *J* = 8.0 Hz, 1H), 7.55 (t, *J* = 7.6 Hz, 2H), 7.45 (t, *J* = 8.0 Hz, 2H), 7.20 (d, *J* = 8.0 Hz, 1H), 7.11 (td, *J* = 7.2, 1.2 Hz, 1H), 7.01 (dd, *J* = 16.0, 8.0 Hz, 2H), 6.76 (s, 1H), 4.20 (dd, *J* = 6.0, 3.2 Hz, 1H), 3.94 (dd, *J* = 18.0, 6.0 Hz, 1H), 3.67 (s, 3H), 3.48 (s, 3H), 3.22 (d, *J* = 18.0 Hz, 1H), 3.00 (dd, *J* = 18.0, 3.2 Hz, 1H), 2.69 (d, *J* = 18.0 Hz, 1H), 2.21 (s, 3H), 2.13 (s, 3H); $^{13}C$ NMR (100 MHz, $CDCl_3$) δ 198.71, 175.13, 143.09, 137.84, 136.85, 136.09, 135.77, 133.62, 133.49, 129.72, 128.81, 128.42, 126.71, 124.14, 121.67, 121.15, 119.29, 112.79, 109.27, 61.79, 53.59, 52.39, 41.15, 39.85, 37.41, 29.44, 25.11, 20.99; HRMS (ESI) *m/z* calcd for $C_{31}H_{30}N_2O_3$ $[M+H]^+$ = 479.2329, found = 479.2333; The ee value was 83%, $t_R$ (major) = 9.0 min, $t_R$ (minor) = 14.3 min (Chiralcel IA, λ = 254 nm, 10% i-PrOH/hexane, flow rate = 1.0 mL/min).

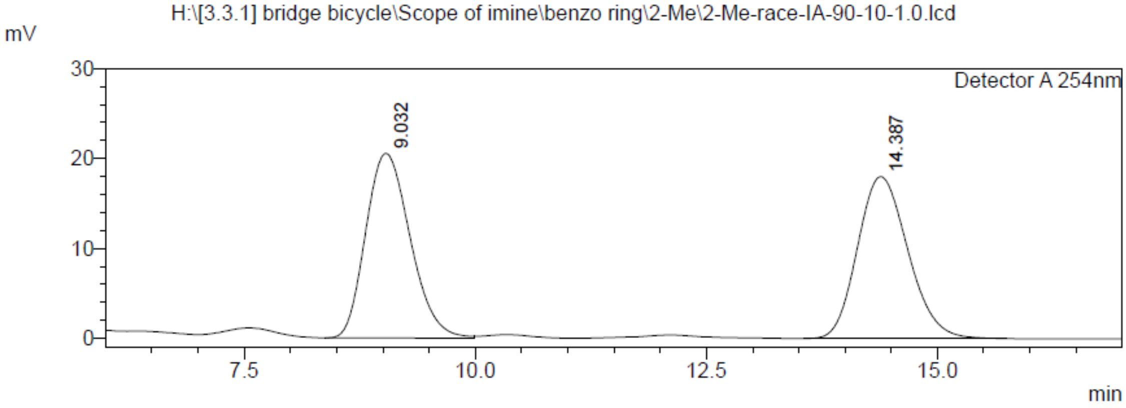


Detector A 254nm

| Peak# | Ret. Time | Height | Height% | Area | Area% |
|---|---|---|---|---|---|
| 1 | 9.032 | 20541 | 53.276 | 687521 | 50.150 |
| 2 | 14.387 | 18015 | 46.724 | 683408 | 49.850 |
| Total | | 38556 | 100.000 | 1370929 | 100.000 |

Racemic **5b**

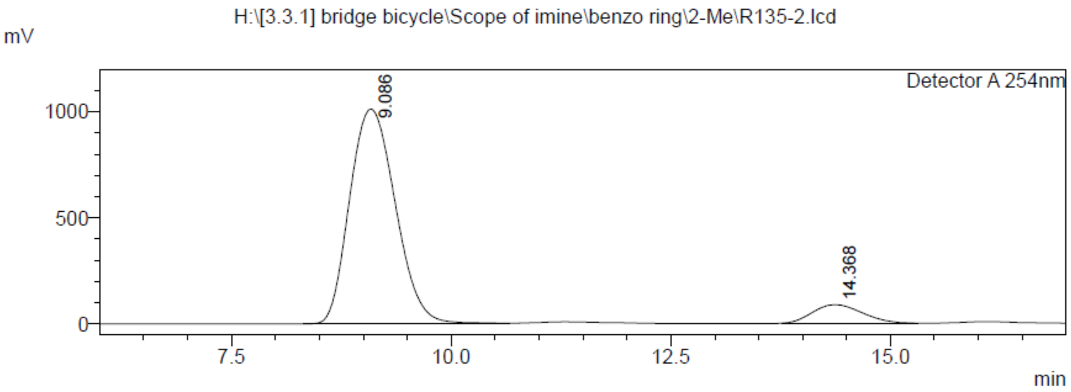


Detector A 254nm

| Peak# | Ret. Time | Height | Height% | Area | Area% |
|---|---|---|---|---|---|
| 1 | 9.086 | 1012638 | 92.034 | 36740177 | 91.406 |
| 2 | 14.368 | 87651 | 7.966 | 3454145 | 8.594 |
| Total | | 1100289 | 100.000 | 40194321 | 100.000 |

Enantiomerically enriched **5b**

**Methyl(6S,7S,13R)-3-fluoro-8,13-dimethyl-7-(2-oxo-2-phenylethyl)-5,7,8,13-tetrahydro-6H-6,13-epiminobenzo[4,5]cycloocta[1,2-b]indole-6-carboxylate(5c)**

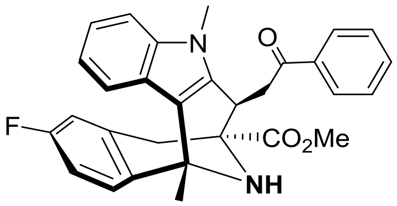


**5c**

A white solid; 45.7 mg, 95% yield; m.p. =148.5-150.2 °C, $[\alpha]^{25}_D$ = -45,07 (*c* 0.32, $CHCl_3$); *dr* >20:1; $^1$H NMR (400 MHz, $CDCl_3$) δ 7.99 (d, *J* = 7.2 Hz, 2H), 7.62 (dd, *J* = 8.4, 5.6 Hz, 1H), 7.60 – 7.56 (m, 2H), 7.46 (t, *J* = 8.0 Hz, 2H), 7.22 (d, *J* = 8.0 Hz, 1H), 7.14 (td, *J* = 7.2, 1.2 Hz, 1H), 7.05 (td, *J* = 8.0, 0.8 Hz, 1H), 6.87 (td, *J* = 8.4, 2.4 Hz, 1H), 6.66 (dd, *J* = 9.2, 2.4 Hz, 1H), 4.21 (dd, *J* = 6.4, 2.8 Hz, 1H), 3.94 (dd, *J* = 18.0, 6.4 Hz, 1H), 3.68 (s, 3H), 3.50 (s, 3H), 3.26 (d, *J* = 17.6 Hz, 1H), 2.99 (dd, *J* = 18.0, 2.8 Hz, 1H), 2.72 (d, *J* = 17.6 Hz, 1H), 2.14 (s, 3H); $^{13}$C NMR (100MHz, $CDCl_3$) δ 198.49, 174.87, 161.27 (d, *J* = 241.4 Hz), 141.73, 137.83, 136.77, 136.15 (d, *J* = 5.0 Hz), 136.06, 133.57, 128.85, 128.41, 123.98, 123.11 (d, *J* = 8.4 Hz), 121.32, 119.41, 119.13, 115.71 (d, *J* = 21.2 Hz), 112.56 (d, *J* = 11.2 Hz), 112.41, 109.38, 61.55, 53.49, 52.52, 41.03, 39.76, 37.55, 29.46, 25.22;. $^{19}$F NMR (376 MHz, $CDCl_3$) δ -107.50; HRMS (ESI) *m/z* calcd for $C_{30}H_{27}FN_2O_3$ $[M+H]^+$ = 483.2078, found = 483.2080; The ee value was 90%, $t_R$ (major) = 11.4 min, $t_R$ (minor) = 31.6 min (Chiralcel IA, λ = 254 nm, 10% i-PrOH/hexane, flow rate = 1.0 mL/min).

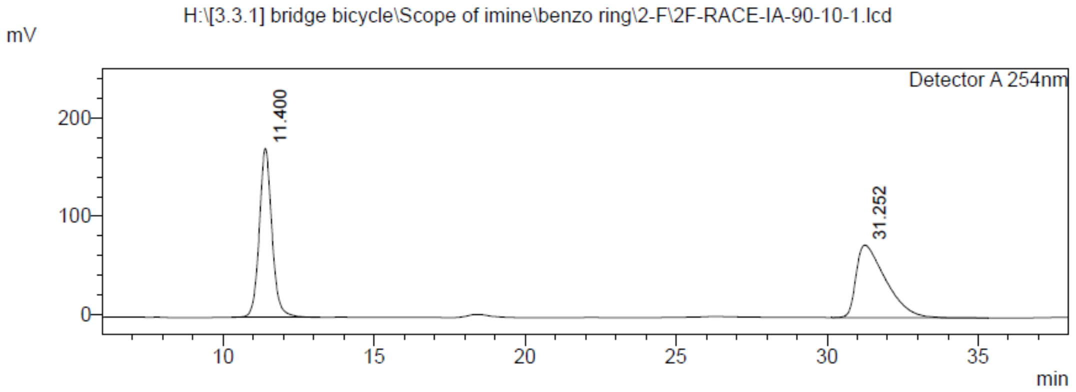


Detector A 254nm

| Peak# | Ret. Time | Height | Height% | Area | Area% |
|---|---|---|---|---|---|
| 1 | 11.400 | 171496 | 69.959 | 5012778 | 49.978 |
| 2 | 31.252 | 73642 | 30.041 | 5017241 | 50.022 |
| Total | | 245139 | 100.000 | 10030020 | 100.000 |

Racemic **5c**

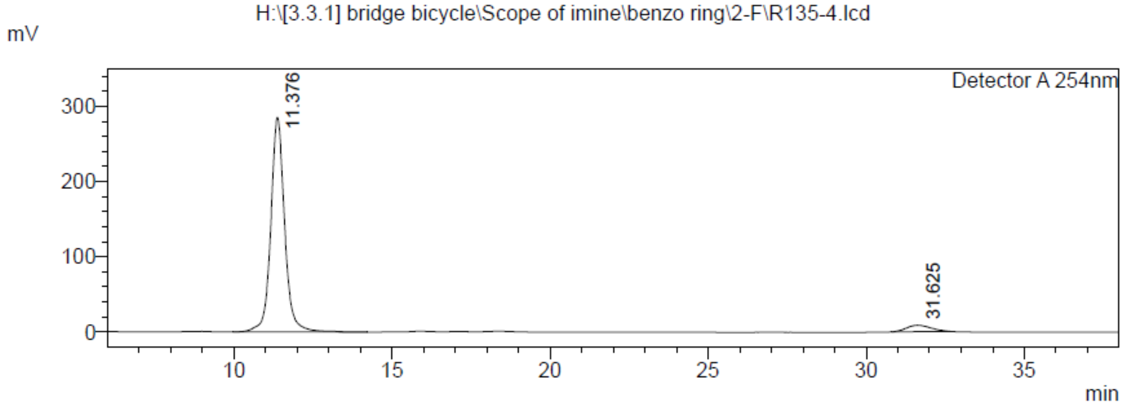


Detector A 254nm

| Peak# | Ret. Time | Height | Height% | Area | Area% |
|---|---|---|---|---|---|
| 1 | 11.376 | 284997 | 97.088 | 8676584 | 95.044 |
| 2 | 31.625 | 8548 | 2.912 | 452394 | 4.956 |
| Total | | 293546 | 100.000 | 9128978 | 100.000 |

Enantiomerically enriched **5c**

**Methyl(6S,7S,13R)-3-bromo-8,13-dimethyl-7-(2-oxo-2-phenylethyl)-5,7,8,13-tetrahydro-6H-6,13-epiminobenzo[4,5]cycloocta[1,2-b]indole-6-carboxylate(5d)**

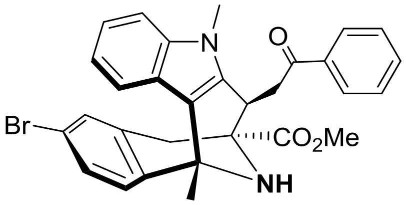


**5d**

A white solid; 50.9 mg, 94% yield; m.p. =192.3-193.2 $^{o}$C, $[\alpha]^{25}_{D}$ = -52,79 (*c* 0.42,

CHCl$_3$); *dr* >20:1; $^1$H NMR (400 MHz, CDCl$_3$) δ 8.00 (d, *J* = 7.2 Hz, 2H), 7.59 – 7.54 (m, 3H), 7.46 (t, *J* = 7.6 Hz, 2H), 7.31 (dd, *J* = 8.4, 1.6 Hz, 1H), 7.23 (d, *J* = 8.0 Hz, 1H), 7.14 (td, *J* = 7.2, 1.2 Hz, 1H), 7.09 (d, *J* = 0.8 Hz, 1H), 7.05 (t, *J* = 7.6 Hz, 1H), 4.21 (dd, *J* = 6.0, 2.8 Hz, 1H), 3.94 (dd, *J* = 18.0, 6.0 Hz, 1H), 3.68 (s, 3H), 3.50 (s, 3H), 3.24 (d, *J* = 18.0 Hz, 1H), 3.00 (dd, *J* = 18.0, 2.8 Hz, 1H), 2.71 (d, *J* = 18.0 Hz, 1H), 2.13 (s, 3H); $^{13}$C NMR (100 MHz, CDCl$_3$) δ 198.46, 174.75, 144.92, 137.83, 136.75, 136.36, 126.21, 133.57, 131.76, 129.03, 128.85, 128.40, 123.92, 123.41, 121.37, 119.77, 119.46, 119.05, 112.03, 109.42, 61.56, 53.54, 52.53, 41.00, 39.76, 37.08, 29.46, 24.94; HRMS (ESI) *m/z* calcd for $C_{30}H_{27}BrN_2O_3$ $[M+H]^+$ = 543.1278, found = 543.1282; The ee value was 98%, $t_R$ (major) = 11.8 min, $t_R$ (minor) = 23.4 min (Chiralcel IA, λ = 254 nm, 10% i-PrOH/hexane, flow rate = 1.0 mL/min).

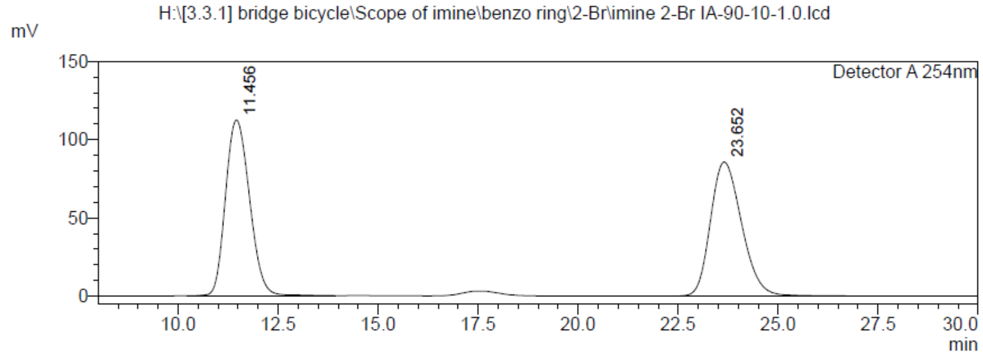


Detector A 254nm

| Peak# | Ret. Time | Height | Height% | Area | Area% |
|---|---|---|---|---|---|
| 1 | 11.456 | 112343 | 56.742 | 4701478 | 50.350 |
| 2 | 23.652 | 85647 | 43.258 | 4636181 | 49.650 |
| Total | | 197990 | 100.000 | 9337659 | 100.000 |

Racemic **5d**

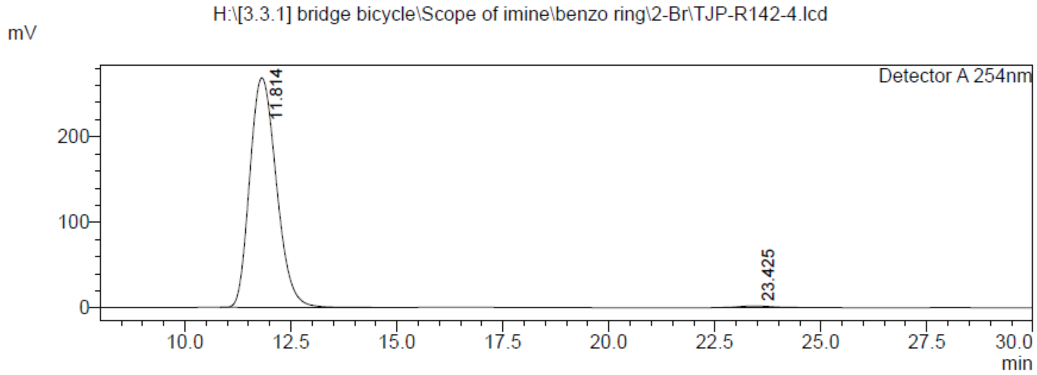


Detector A 254nm

| Peak# | Ret. Time | Height | Height% | Area | Area% |
|---|---|---|---|---|---|
| 1 | 11.814 | 268253 | 99.334 | 11739701 | 99.111 |
| 2 | 23.425 | 1800 | 0.666 | 105306 | 0.889 |
| Total | | 270052 | 100.000 | 11845007 | 100.000 |

Enantiomerically enriched **5d**

**Methyl(6S,7S,13R)-2-fluoro-8,13-dimethyl-7-(2-oxo-2-phenylethyl)-5,7,8,13-tetrahydro-6H-6,13-epiminobenzo[4,5]cycloocta[1,2-b]indole-6-carboxylate(5e)**

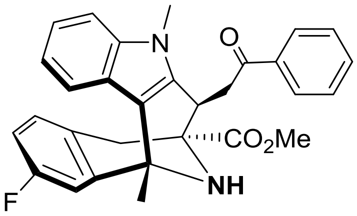


**5e**

A white solid; 44.8 mg, 93% yield; m.p. =200.2-202.2 °C, $[\alpha]^{25}_{D}$ = -51,26 (*c* 0.35, $CHCl_3$); *dr* >20:1; $^1$H NMR (400 MHz, $CDCl_3$) δ 7.99 (d, *J* = 7.2 Hz, 2H), 7.58 (dd, *J* = 18.0, 8.0 Hz, 2H), 7.46 (t, *J* = 8.0 Hz, 2H), 7.38 (dd, *J* = 9.6, 2.4 Hz, 1H), 7.22 (d, *J* = 8.4 Hz, 1H), 7.14 (t, *J* = 7.2 Hz, 1H), 7.06 (t, *J* = 7.6 Hz, 1H), 6.87 (dd, *J* = 8.0, 6.0 Hz, 1H), 6.75 (td, *J* = 8.4, 2.4 Hz, 1H), 4.21 (dd, *J* = 6.4, 3.2 Hz, 1H), 3.94 (dd, *J* = 18.0, 6.4 Hz, 1H), 3.68 (s, 3H), 3.49 (s, 3H), 3.24 (d, *J* = 17.6 Hz, 1H), 2.99 (dd, *J* = 18.0, 3.2 Hz, 1H), 2.63 (d, *J* = 17.6 Hz, 1H), 2.31 (s, 1H), 2.12 (s, 3H); $^{13}$C NMR (100 MHz, $CDCl_3$) δ 198.41, 174.81, 161.30 (d, *J* = 244.4 Hz), 147.72 (d, *J* = 5.6 Hz), 137.68, 136.49 (d, *J* = 31.6 Hz), 133.44, 130.03 (d, *J* = 8.2 Hz), 129.10 (d, *J* = 3.0 Hz), 128.72, 128.28, 123.86, 121.21, 119.36, 119.06, 112.69 (d, *J* = 21.4 Hz), 111.65, 109.26 (d, *J* = 3.2 Hz), 109.05, 61.55, 53.64 , 52.36, 40.91, 39.70, 36.52, 29.34, 24.87; $^{19}$F NMR (376 MHz, $CDCl_3$) δ -116.22; HRMS (ESI) *m/z* calcd for $C_{30}H_{27}FN_2O_3$ $[M+H]^+$ = 483.2078, found = 483.2081; The ee value was 98%, $t_R$ (major) = 15.3 min, $t_R$ (minor) = 20.7 min (Chiralcel IA, λ = 254 nm, 5% i-PrOH/hexane, flow rate = 1.0 mL/min).

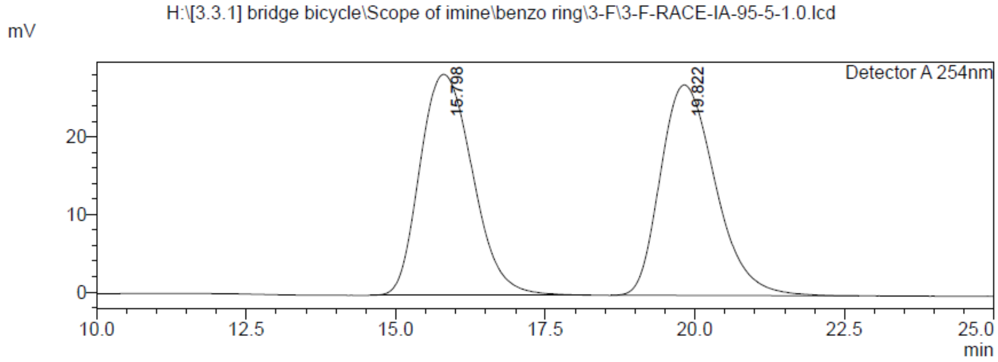


Detector A 254nm

| Peak# | Ret. Time | Height | Height% | Area | Area% |
|---|---|---|---|---|---|
| 1 | 15.798 | 28467 | 51.204 | 1743782 | 50.141 |
| 2 | 19.822 | 27128 | 48.796 | 1733962 | 49.859 |
| Total | | 55595 | 100.000 | 3477744 | 100.000 |

Racemic **5e**

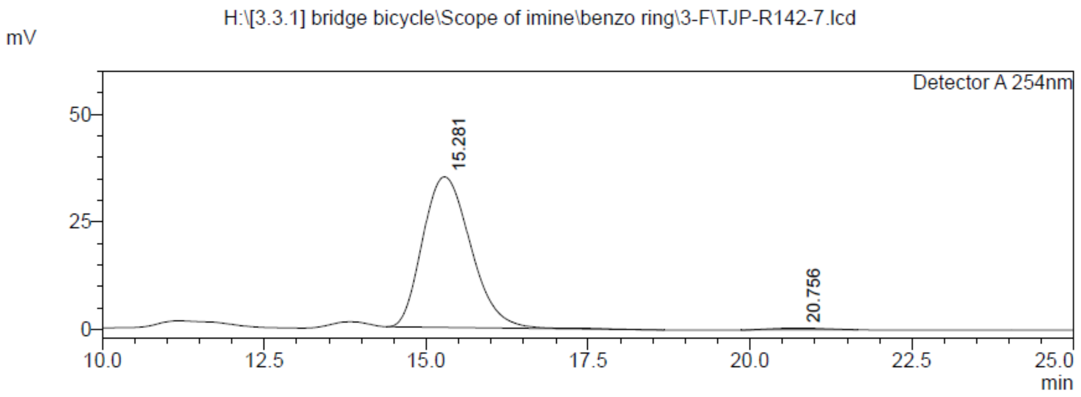


Detector A 254nm

| Peak# | Ret. Time | Height | Height% | Area | Area% |
|---|---|---|---|---|---|
| 1 | 15.281 | 34975 | 98.949 | 1815312 | 98.773 |
| 2 | 20.756 | 371 | 1.051 | 22560 | 1.227 |
| Total | | 35347 | 100.000 | 1837871 | 100.000 |

Enantiomerically enriched **5e**

<u>**Methyl(6S,7S,13R)-2-chloro-8,13-dimethyl-7-(2-oxo-2-phenylethyl)-5,7,8,13-tetrahydro-6H-6,13-epiminobenzo[4,5]cycloocta[1,2-b]indole-6-carboxylate(5f)**</u>

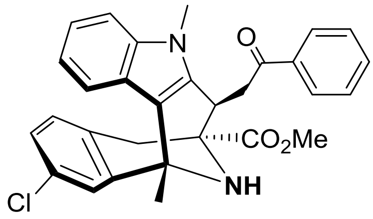


**5f**

A white solid; 45.8 mg, 92% yield; m.p. =208.4-209.0 °C, $[\alpha]^{25}_{D}$ = -56,25 (*c* 0.38 $CHCl_3$); *dr* >20:1; $^1$H NMR (400 MHz, $CDCl_3$) δ 8.00 (d, *J* = 7.2 Hz, 2H), 7.66 (d, *J* = 2.0 Hz, 1H), 7.61 (d, *J* = 7.6 Hz, 1H), 7.57 (t, *J* = 7.6 Hz, 1H), 7.46 (t, *J* = 8.0 Hz, 2H), 7.23 (d, *J* = 8.0 Hz, 1H), 7.16 (td, *J* = 7.2, 1.2 Hz, 1H), 7.09 (t, *J* = 7.4 Hz, 1H), 7.04 (dd, *J* = 8.0, 2.0 Hz, 1H), 6.86 (d, *J* = 8.0 Hz, 1H), 4.22 (dd, *J* = 6.4, 3.2 Hz, 1H), 3.95 (dd, *J* = 18.0, 6.4 Hz, 1H), 3.69 (s, 3H), 3.49 (s, 3H), 3.24 (d, *J* = 17.6 Hz, 1H), 2.99 (dd, *J* = 18.0, 3.2 Hz, 1H), 2.65 (d, *J* = 17.6 Hz, 1H), 2.13 (s, 3H); $^{13}$C NMR (100 MHz, $CDCl_3$) δ 198.50, 174.82, 147.57, 137.78, 136.72, 136.41, 133.58, 132.22, 131.88, 130.25, 128.85, 128.39, 126.26, 123.93, 122.16, 121.36, 119.53, 119.17, 111.74, 109.36, 61.58, 53.74, 52.51, 40.98, 39.77, 36.77, 29.46, 24.96; HRMS (ESI) *m/z* calcd for $C_{30}H_{27}ClN_2O_3$ $[M+H]^+$ = 499.1783, found = 499.1781; The ee value was 93%, $t_R$ (major) = 15.6 min, $t_R$ (minor) = 17.6 min (Chiralcel IA, λ = 254 nm, 5% i-PrOH/hexane, flow rate = 1.0 mL/min).

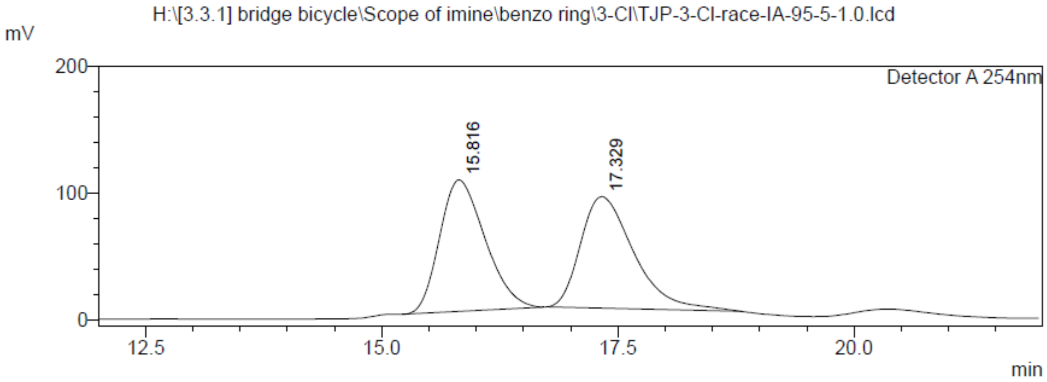


Detector A 254nm

| Peak# | Ret. Time | Height | Height% | Area | Area% |
|---|---|---|---|---|---|
| 1 | 15.816 | 103803 | 54.159 | 3476561 | 50.140 |
| 2 | 17.329 | 87860 | 45.841 | 3457109 | 49.860 |
| Total | | 191663 | 100.000 | 6933669 | 100.000 |

Racemic **5f**

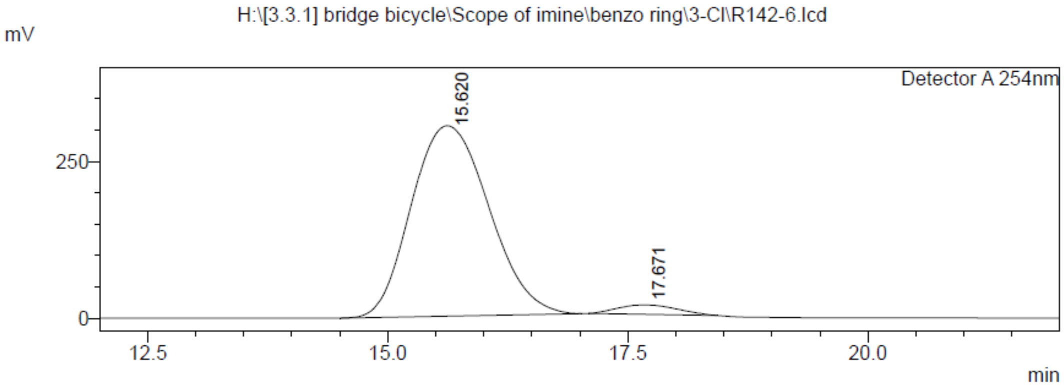


Detector A 254nm

| Peak# | Ret. Time | Height | Height% | Area | Area% |
|---|---|---|---|---|---|
| 1 | 15.620 | 303463 | 95.255 | 17101154 | 96.310 |
| 2 | 17.671 | 15116 | 4.745 | 655244 | 3.690 |
| Total | | 318579 | 100.000 | 17756398 | 100.000 |

Enantiomerically enriched **5f**

**Methyl(6S,7S,13R)-2,8,13-trimethyl-7-(2-oxo-2-phenylethyl)-5,7,8,13-tetrahydro-6H-6,13-epiminobenzo[4,5]cycloocta[1,2-b]indole-6-carboxylate (5g)**

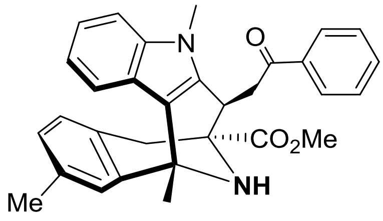


**5g**

A white solid; 43.4 mg, 91% yield; m.p. =75.6-76.8 °C, $[\alpha]^{25}_D$ = -58,21 (*c* 0.32 $CHCl_3$); *dr* >20:1; $^1H$ NMR (400 MHz, $CDCl_3$) δ 7.99 (d, *J* = 7.6 Hz, 2H), 7.63 (d, *J* = 7.6 Hz,

1H), 7.56 (t, $J$ = 7.2 Hz, 1H), 7.46 (dd, $J$ = 14.6, 6.8 Hz, 3H), 7.21 (d, $J$ = 8.4 Hz, 1H), 7.12 (t, $J$ = 7.6 Hz, 1H), 7.05 (t, $J$ = 7.6 Hz, 1H), 6.85 (dd, $J$ = 21.2, 7.6 Hz, 2H), 4.20 (s, 1H), 3.94 (dd, $J$ = 18.0, 5.6 Hz, 1H), 3.67 (s, 3H), 3.48 (s, 3H), 3.23 (d, $J$ = 17.6 Hz, 1H), 3.00 (d, $J$ = 17.6 Hz, 1H), 2.67 (d, $J$ = 18.0 Hz, 1H), 2.37 (s, 3H), 2.14 (s, 3H); $^{13}$C NMR (100 MHz, $CDCl_3$) δ 198.59, 175.01, 145.60, 137.71, 136.72, 136.15, 135.50, 133.36, 130.47, 128.76, 128.68, 128.29, 126.79, 124.05, 122.47, 121.00, 119.19, 119.13, 112.39, 109.14, 61.66, 53.64, 52.26, 41.01, 39.72, 37.01, 29.33, 24.98, 21.47; HRMS (ESI) *m/z* calcd for $C_{31}H_{30}N_2O_3$ $[M+H]^+$ = 479.2329, found = 479.2337; The ee value was 84%, $t_R$ (major) = 12.2 min, $t_R$ (minor) = 21.3 min (Chiralcel ID, λ = 254 nm, 5% i-PrOH/hexane, flow rate = 1.0 mL/min).

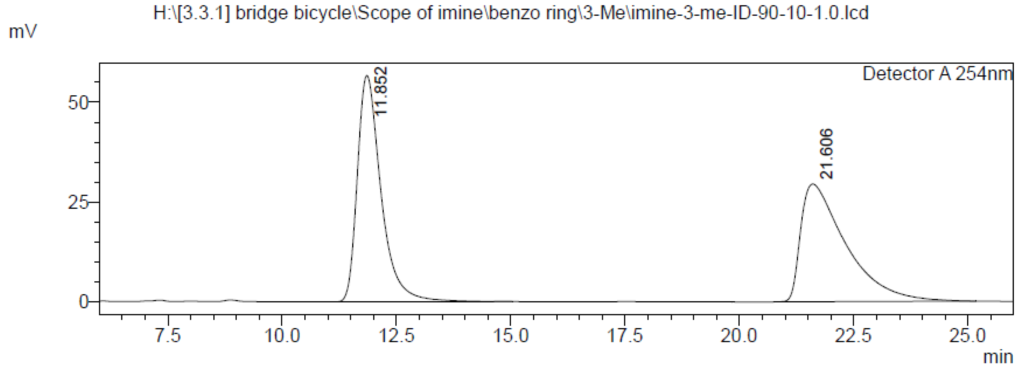


Detector A 254nm

| Peak# | Ret. Time | Height | Height% | Area | Area% |
|---|---|---|---|---|---|
| 1 | 11.852 | 56643 | 65.780 | 2055382 | 50.331 |
| 2 | 21.606 | 29467 | 34.220 | 2028340 | 49.669 |
| Total | | 86109 | 100.000 | 4083722 | 100.000 |

Racemic **5g**

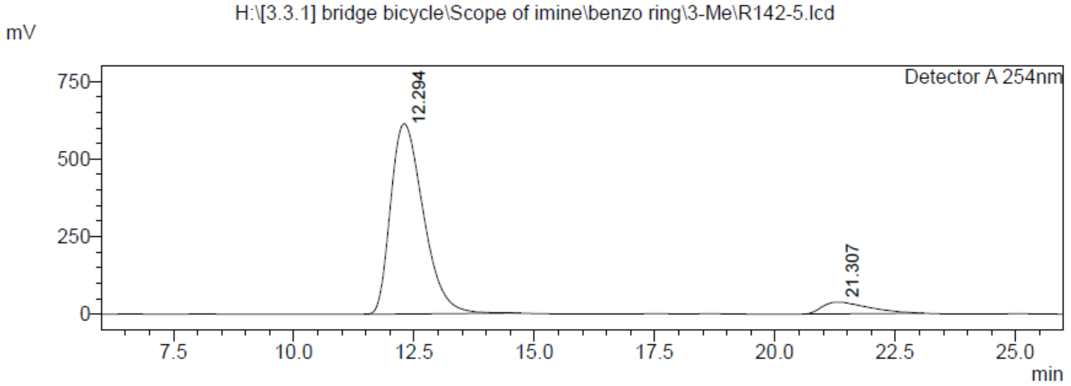


Detector A 254nm

| Peak# | Ret. Time | Height | Height% | Area | Area% |
|---|---|---|---|---|---|
| 1 | 12.294 | 611852 | 94.217 | 29018937 | 92.067 |
| 2 | 21.307 | 37555 | 5.783 | 2500346 | 7.933 |
| Total | | 649407 | 100.000 | 31519283 | 100.000 |

Enantiomerically enriched **5g**

**Methyl(6S,7S,13R)-2-((tert-butyldimethylsilyl)oxy)-8,13-dimethyl-7-(2-oxo-2-phenylethyl)-5,7,8,13-tetrahydro-6H-6,13-epiminobenzo[4,5]cycloocta[1,2-b]indole-6-carboxylate (5h)**

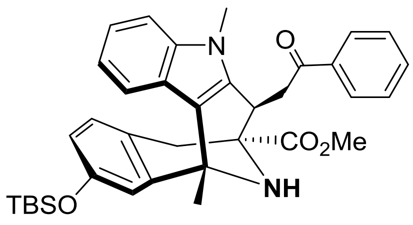

**5h**

A white solid; 54.1 mg, 91% yield; m.p. =197.6-198.3 °C, $[\alpha]^{25}_{D}$ = -21.35 (*c* 0.38 $CHCl_3$); *dr* >20:1; $^1H$ NMR (400 MHz, $CDCl_3$) δ 8.01 (d, *J* = 7.2 Hz, 2H), 7.62 (d, *J* = 8.0 Hz, 1H), 7.57 (t, *J* = 7.2 Hz, 1H), 7.46 (t, *J* = 8.0 Hz, 2H), 7.23 (d, *J* = 8.4 Hz, 1H), 7.19 (d, *J* = 2.4 Hz, 1H), 7.14 (td, *J* = 6.8, 0.8 Hz, 1H), 7.05 (t, *J* = 8.0 Hz, 1H), 6.78 (d, *J* = 8.0 Hz, 1H), 6.56 (dd, *J* = 8.4, 2.4 Hz, 1H), 4.21 (dd, *J* = 6.0, 2.8 Hz, 1H), 3.96 (dd, *J* = 18.0, 6.0 Hz, 1H), 3.69 (s, 3H), 3.50 (s, 3H), 3.21 (d, *J* = 17.2 Hz, 1H), 3.01 (dd, *J* = 18.0, 2.8 Hz, 1H), 2.63 (d, *J* = 17.2 Hz, 1H), 2.13 (s, 3H), 1.02 (s, 9H), 0.22 (d, *J* = 10.4 Hz, 6H); $^{13}C$ NMR (100 MHz, $CDCl_3$) δ 198.69, 175.17, 153.92, 146.81, 137.75, 136.81, 136.34, 133.50, 129.61, 128.81, 128.40, 126.30, 124.13, 121.13, 119.39, 119.23, 117.81, 114.21, 112.31, 109.27, 61.79, 53.74, 52.38, 41.08, 39.86, 36.68, 25.94, 25.06, 18.42, -4.26, -4.18; HRMS (ESI) *m/z* calcd for $C_{36}H_{42}N_2O_4Si$ $[M+H]^+$ = 595.2987, found = 595.2989; The ee value was 94%, $t_R$ (major) = 8.3 min, $t_R$ (minor) = 10.1 min (Chiralcel IB, λ = 254 nm, 5% i-PrOH/hexane, flow rate = 1.0 mL/min).

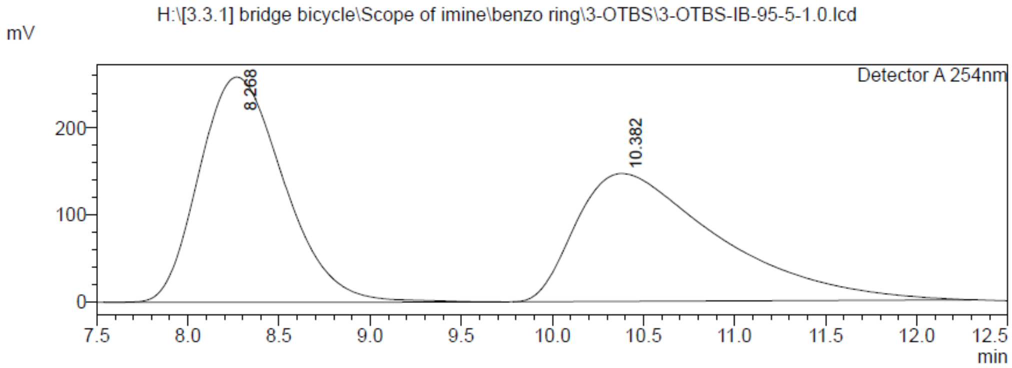


Detector A 254nm

| Peak# | Ret. Time | Height | Height% | Area | Area% |
|---|---|---|---|---|---|
| 1 | 8.268 | 259009 | 63.784 | 8370196 | 51.031 |
| 2 | 10.382 | 147064 | 36.216 | 8031942 | 48.969 |
| Total | | 406072 | 100.000 | 16402138 | 100.000 |

Racemic **5h**

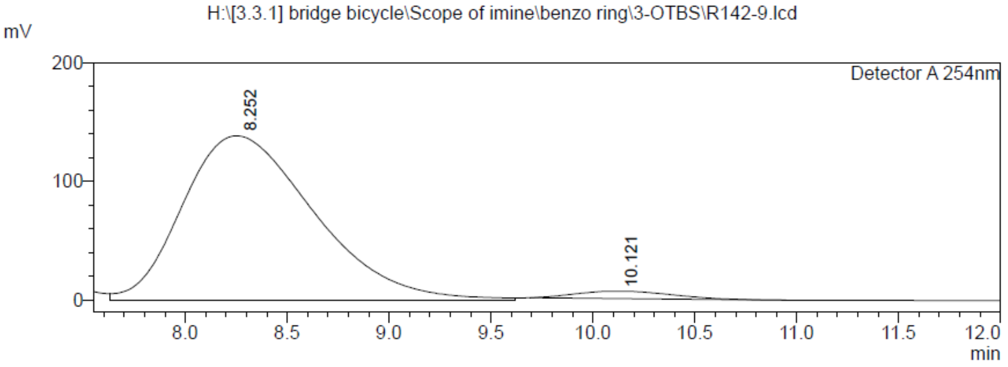


Detector A 254nm

| Peak# | Ret. Time | Height | Height% | Area | Area% |
|---|---|---|---|---|---|
| 1 | 8.252 | 138819 | 95.707 | 6243490 | 96.882 |
| 2 | 10.121 | 6227 | 4.293 | 200957 | 3.118 |
| Total | | 145046 | 100.000 | 6444447 | 100.000 |

Enantiomerically enriched **5h**

**Methyl(6S,7S,13R)-2-((tert-butyldiphenylsilyl)oxy)-8,13-dimethyl-7-(2-oxo-2-phenylethyl)-5,7,8,13-tetrahydro-6H-6,13-epiminobenzo[4,5]cycloocta[1,2-b]indole-6-carboxylate(5i)**

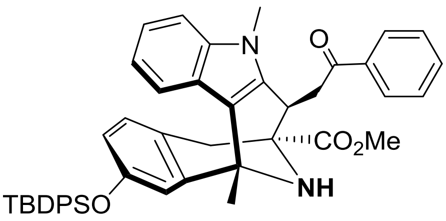


**5i**

A white solid; 64.6 mg, 90% yield; m.p. =75.4-76.6 °C, $[\alpha]^{25}_{D}$ = -18.30 (*c* 0.23 $CHCl_3$); *dr* >20:1; $^1$H NMR (400 MHz, $CDCl_3$) δ 7.96 (d, *J* = 7.2 Hz, 2H), 7.77 (td, *J* = 8.0, 1.2 Hz, 4H), 7.56 – 7.49 (m, 2H), 7.46 – 7.37 (m, 7H), 7.18 (d, *J* = 8.0 Hz, 1H), 7.11 (t, *J* = 6.8 Hz, 1H), 7.07 (d, *J* = 8.0 Hz, 1H), 6.98 (d, *J* = 2.4 Hz, 1H), 6.92 (t, *J* = 7.6 Hz, 1H), 6.69 (d, *J* = 8.0 Hz, 1H), 6.57 (dd, *J* = 8.4, 2.4 Hz, 1H), 4.14 (dd, *J* = 6.0, 2.8 Hz, 1H), 3.88 (dd, *J* = 18.0, 6.0 Hz, 1H), 3.64 (s, 3H), 3.46 (s, 3H), 3.13 (d, *J* = 17.6 Hz, 1H), 2.94 (dd, *J* = 18.0, 2.8 Hz, 1H), 2.55 (d, *J* = 17.6 Hz, 1H), 1.13 (s, 9H); $^{13}$C NMR (100 MHz, $CDCl_3$) δ 198.72, 175.21, 153.94, 146.41, 137.69, 136.81, 136.23, 135.72, 135.67, 133.48, 133.32, 133.27, 130.03, 129.59, 128.79, 128.39, 128.06, 127.97, 125.82, 124.05, 120.99, 119.39, 119.34, 117.31, 113.55, 112.17, 109.08, 61.78, 53.66, 52.36, 41.00, 39.85, 36.63, 29.41, 26.76, 24.76, 19.61; HRMS (ESI) *m/z* calcd for $C_{46}H_{46}N_2O_4Si$ $[M+H]^+$ = 719.3330, found = 719.3305; The ee value was 82%, $t_R$ (major) = 9.4 min, $t_R$ (minor) = 13.7 min (Chiralcel ID, λ = 254 nm, 10% i-PrOH/hexane, flow rate = 1.0 mL/min).

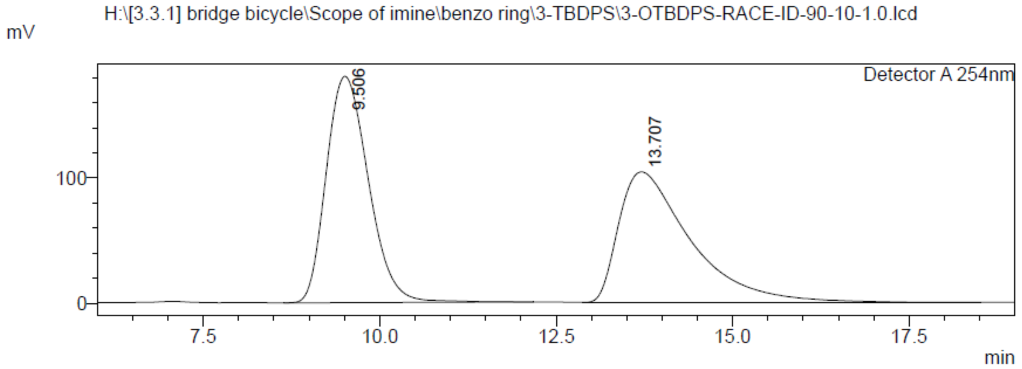


Detector A 254nm

| Peak# | Ret. Time | Height | Height% | Area | Area% |
|---|---|---|---|---|---|
| 1 | 9.506 | 180423 | 63.426 | 7514175 | 50.487 |
| 2 | 13.707 | 104038 | 36.574 | 7369314 | 49.513 |
| Total | | 284460 | 100.000 | 14883489 | 100.000 |

Racemic **5i**

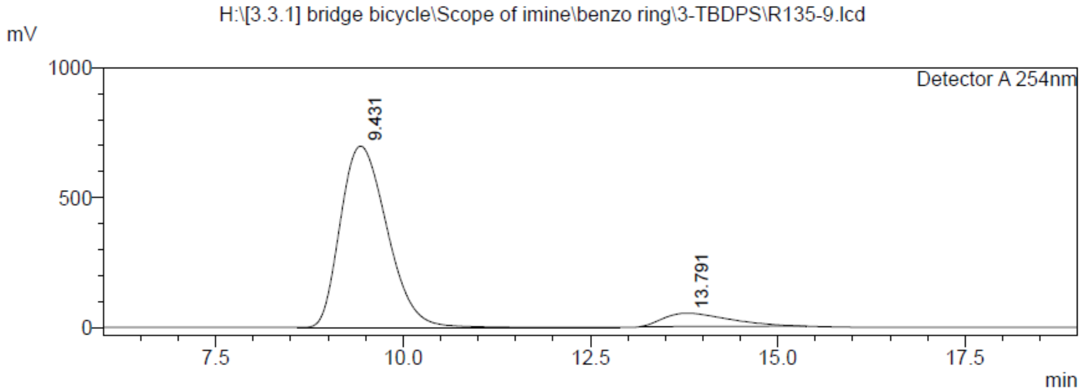


Detector A 254nm

| Peak# | Ret. Time | Height | Height% | Area | Area% |
|---|---|---|---|---|---|
| 1 | 9.431 | 698344 | 93.233 | 30395844 | 90.729 |
| 2 | 13.791 | 50688 | 6.767 | 3106069 | 9.271 |
| Total | | 749033 | 100.000 | 33501913 | 100.000 |

Enantiomerically enriched **5i**

**Methyl(6S,7S,13R)-8,13-dimethyl-7-(2-oxo-2-phenylethyl)-2-(tosyloxy)-5,7,8,13-tetrahydro-6H-6,13-epiminobenzo[4,5]cycloocta[1,2-b]indole-6-carboxylate(5j)**

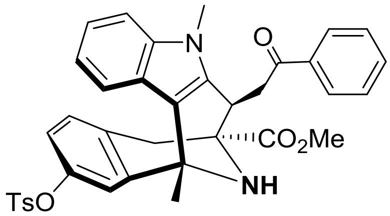


**5j**

A white solid; 55.1 mg, 87% yield; m.p. =85.6-86.7 °C, $[\alpha]^{25}_D$ = -17.21 (*c* 0.25 $CHCl_3$); *dr* >20:1; $^1H$ NMR (400 MHz, $CDCl_3$) δ 7.98 (d, *J* = 7.2 Hz, 2H), 7.71 (d, *J* = 8.4 Hz,

2H), 7.57 (t, $J$ = 7.6 Hz, 1H), 7.59 – 7.43 (m, 3H), 7.31 (t, $J$ = 5.6 Hz, 3H), 7.22 (d, $J$ = 8.0 Hz, 1H), 7.15 (t, $J$ = 7.6 Hz, 1H), 7.05 (t, $J$ = 7.2 Hz, 1H), 6.82 (d, $J$ = 8.4 Hz, 1H), 6.66 (dd, $J$ = 8.0, 2.0 Hz, 1H), 4.19 (dd, $J$ = 6.0, 2.8 Hz, 1H), 3.93 (dd, $J$ = 18.4, 6.0 Hz, 1H), 3.67 (s, 3H), 3.48 (s, 3H), 3.23 (d, $J$ = 18.0 Hz, 1H), 2.98 (dd, $J$ = 18.4, 2.8 Hz, 1H), 2.63 (d, $J$ = 18.0 Hz, 1H), 2.46 (s, 3H), 1.99 (s, 3H); $^{13}$C NMR (100 MHz, $CDCl_3$) δ 198.45, 174.82, 147.95, 147.38, 145.29, 137.74, 136.70, 136.35, 133.60, 132.78, 132.66, 129.88, 128.85, 128.72, 128.38, 123.89, 121.35, 119.90, 119.51, 119.15, 116.03, 111.66, 109.31, 61.57, 53.64, 52.52, 40.92, 39.78, 36.72, 29.44, 24.83, 21.87; HRMS (ESI) $m/z$ calcd for $C_{37}H_{34}N_2O_6S$ $[M+H]^+$ = 635.2210, found = 635.2212; The ee value was 96%, $t_R$ (major) = 16.9 min, $t_R$ (minor) = 25.3 min (Chiralcel IA, λ = 254 nm, 10% i-PrOH/hexane, flow rate = 1.0 mL/min).

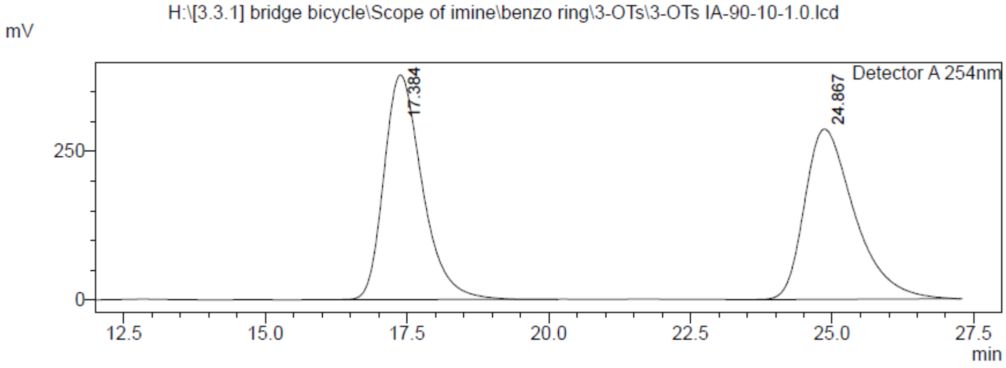


Detector A 254nm

| Peak# | Ret. Time | Height | Height% | Area | Area% |
|---|---|---|---|---|---|
| 1 | 17.384 | 377455 | 56.880 | 17754088 | 50.202 |
| 2 | 24.867 | 286148 | 43.120 | 17611275 | 49.798 |
| Total | | 663603 | 100.000 | 35365363 | 100.000 |

Racemic **5j**

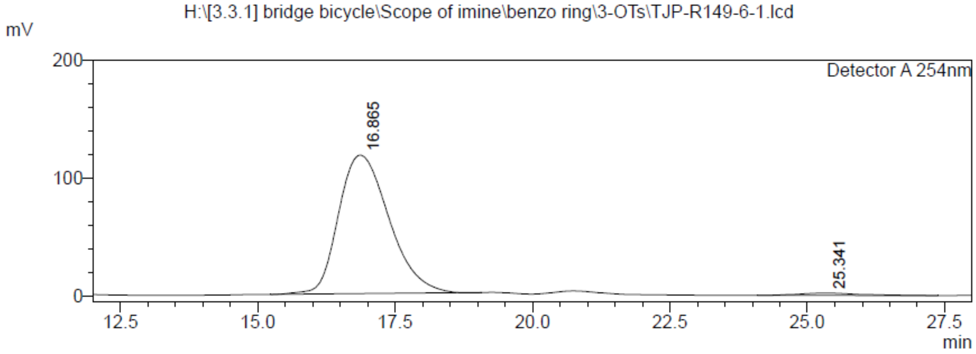


Detector A 254nm

| Peak# | Ret. Time | Height | Height% | Area | Area% |
|---|---|---|---|---|---|
| 1 | 16.865 | 117463 | 98.326 | 7523490 | 97.974 |
| 2 | 25.341 | 2000 | 1.674 | 155605 | 2.026 |
| Total | | 119463 | 100.000 | 7679096 | 100.000 |

Enantiomerically enriched **5j**

**Methyl(6S,7S,13R)-2-((tert-butoxycarbonyl)oxy)-8,13-dimethyl-7-(2-oxo-2-phenylethyl)-5,7,8,13-tetrahydro-6H-6,13-epiminobenzo[4,5]cycloocta[1,2-b]indole-6-carboxylate(5k)**

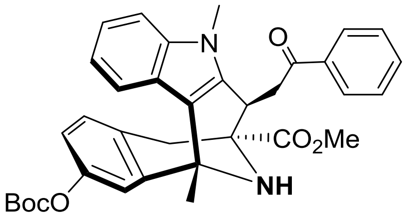


**5k**

A white solid; 51.6 mg, 89% yield; m.p. = 190.2-191.1 $^{o}$C, $[\alpha]^{25}_{D}$ = -41.67 (*c* 0.45 $CHCl_3$); *dr* >20:1; $^{1}$H NMR (400 MHz, $CDCl_3$) δ 8.00 (d, *J* = 7.2 Hz, 2H), 7.58 (dd, *J* = 18.4, 8.0 Hz, 2H), 7.47 – 7.44 (m, 3H), 7.22 (d, *J* = 8.0 Hz, 1H), 7.13 (t, *J* = 7.2 Hz, 1H), 7.05 (t, *J* = 7.6 Hz, 1H), 6.95 – 6.90 (m, 2H), 4.22 (dd, *J* = 6.0, 2.8 Hz, 1H), 3.95 (dd, *J* = 18.0, 6.0 Hz, 1H), 3.68 (s, 3H), 3.49 (s, 3H), 3.26 (d, *J* = 17.6 Hz, 1H), 3.00 (dd, *J* = 18.0, 2,8 Hz, 1H), 2.66 (d, *J* = 17.6 Hz, 1H), 2.14 (s, 3H), 1.59 (s, 9H); $^{13}$C NMR (100 MHz, $CDCl_3$) δ 198.62, 174.87, 152.01, 149.32, 147.09, 137.80, 136.79, 136.38, 133.52, 131.16, 129.66, 128.82, 128.40, 124.01, 121.28, 119.43, 119.31, 118.99, 114.95, 111.94, 109.29, 83.59, 61.73, 53.77, 52.45, 41.08, 39.85, 36.82, 29.46, 27.88, 25.01; HRMS (ESI) *m/z* calcd for $C_{35}H_{36}N_2O_6$ $[M+H]^+$ = 581.2646, found = 581.2649; The ee value was 85%, $t_R$ (major) = 21.5 min, $t_R$ (minor) = 13.8 min (Chiralcel IA, λ = 254 nm, 10% i-PrOH/hexane, flow rate = 1.0 mL/min).

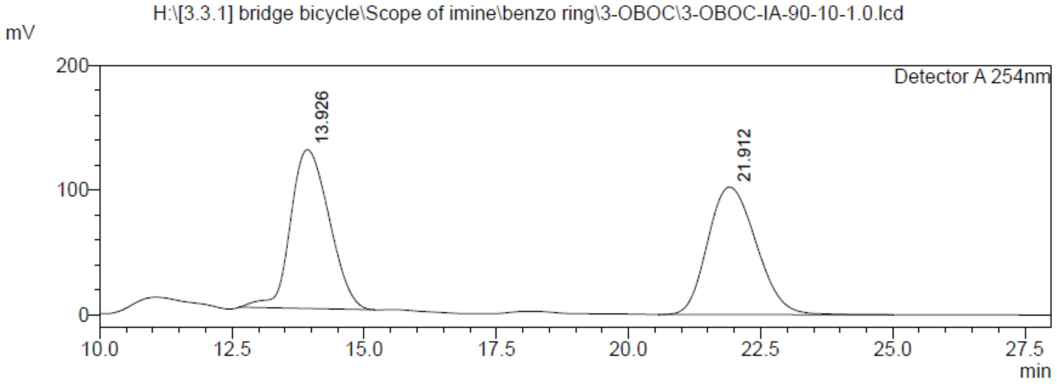


Detector A 254nm

| Peak# | Ret. Time | Height | Height% | Area | Area% |
|---|---|---|---|---|---|
| 1 | 13.926 | 127410 | 55.475 | 6508580 | 49.932 |
| 2 | 21.912 | 102261 | 44.525 | 6526304 | 50.068 |
| Total | | 229671 | 100.000 | 13034884 | 100.000 |

Racemic **5k**

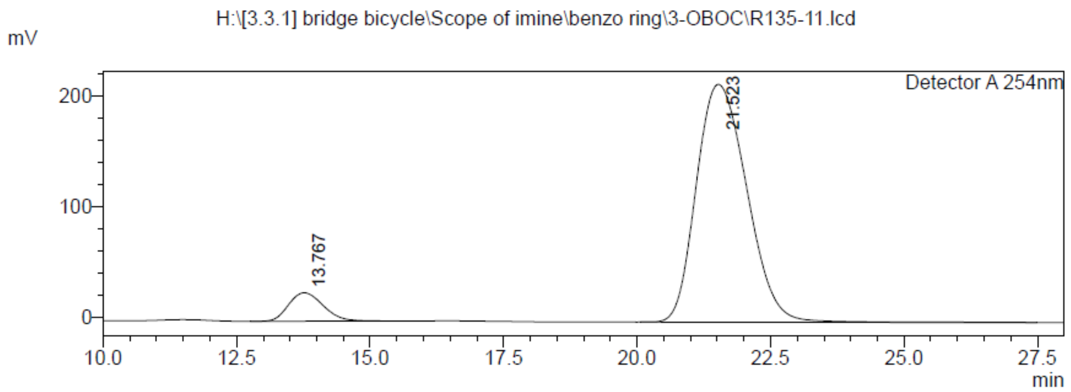


Detector A 254nm

| Peak# | Ret. Time | Height | Height% | Area | Area% |
|---|---|---|---|---|---|
| 1 | 13.767 | 25620 | 10.661 | 1160555 | 7.568 |
| 2 | 21.523 | 214686 | 89.339 | 14174165 | 92.432 |
| Total | | 240306 | 100.000 | 15334719 | 100.000 |

Enantiomerically enriched **5k**

**Methyl(6S,7S,13R)-8,13-dimethyl-2-((naphthalen-2-ylsulfonyl)oxy)-7-(2-oxo-2-phenylethyl)-5,7,8,13-tetrahydro-6H-6,13-epiminobenzo[4,5]cycloocta[1,2-b]indole-6-carboxylate (5l)**

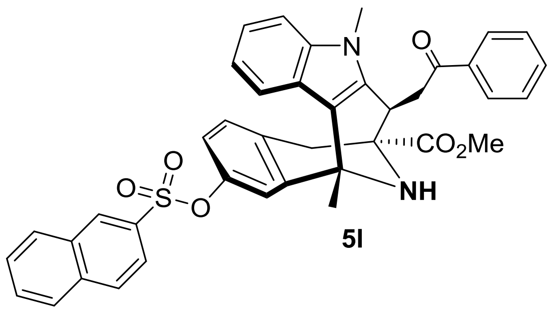


A white solid; 54.9 mg, 82% yield; m.p. = 73.4-74.5 °C, $[\alpha]^{25}_D$ = -65.98 (*c* 0.65 $CHCl_3$); *dr* >20:1; $^1$H NMR (400 MHz, $CDCl_3$) δ 8.33 (s, 1H), 7.99 – 7.93 (m, 4H), 7.86 (dd, *J* = 8.8, 2.0 Hz, 1H), 7.82 (d, *J* = 8.0 Hz, 1H), 7.71 – 7.67 (m, 1H), 7.61 (t, *J* = 7.6 Hz, 1H), 7.56 (t, *J* = 7.2 Hz, 1H), 7.45 (t, *J* = 8.0 Hz, 2H), 7.38 – 7.35 (m, 2H), 7.20 (d, *J* = 8.0 Hz, 1H), 7.11 (t, *J* = 8.0 Hz, 1H), 6.89 (t, *J* = 7.2 Hz, 1H), 6.77 (d, *J* = 8.4 Hz, 1H), 6.64 (dd, *J* = 8.4, 2.4 Hz, 1H), 4.16 (dd, *J* = 6.0, 3.2 Hz, 1H), 3.90 (dd, *J* = 18.0, 6.0 Hz, 1H), 3.65 (s, 3H), 3.47 (s, 3H), 3.19 (d, *J* = 18.0 Hz, 1H), 2.95 (dd, *J* = 18.0, 3.2 Hz, 1H), 2.59 (d, *J* = 18.0 Hz, 1H), 1.91 (s, 3H); $^{13}$C NMR (100 MHz, $CDCl_3$) δ 198.45, 174.80, 147.97, 147.45, 137.73, 136.72, 136.31, 135.52, 133.60, 132.88, 132.44, 131.96, 130.75, 129.94, 129.68, 129.61, 128.86, 128.38, 128.13, 127.93, 123.85, 123.11, 121.34, 119.85, 119.55, 119.10, 116.06, 111.68, 109.29, 61.55, 53.61, 52.52, 40.96, 39.76, 36.70, 29.44, 24.74; HRMS (ESI) *m/z* calcd for

$C_{40}H_{34}N_2O_6S[M+H]^+$ = 671.2210, found = 671.2210; The ee value was 90%, $t_R$ (major) = 36.7 min, $t_R$ (minor) = 22.5 min (Chiralcel IB, λ = 254 nm, 15% i-PrOH/hexane, flow rate = 1.0 mL/min).

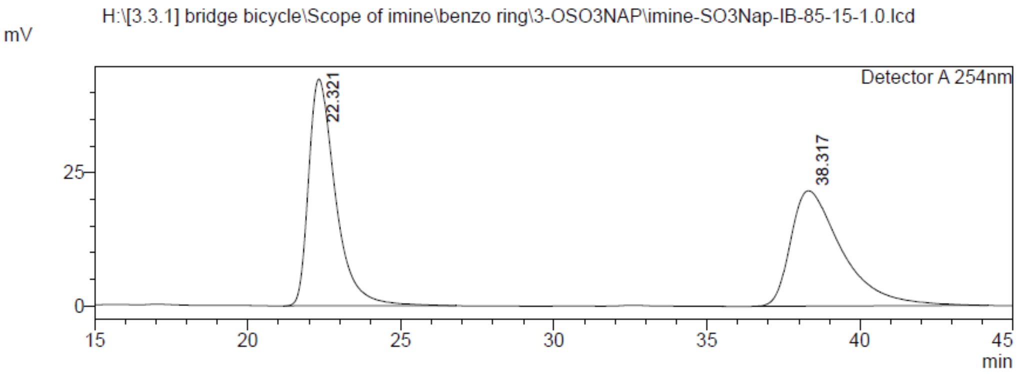


Detector A 254nm

| Peak# | Ret. Time | Height | Height% | Area | Area% |
|---|---|---|---|---|---|
| 1 | 22.321 | 42569 | 66.254 | 2604058 | 50.674 |
| 2 | 38.317 | 21683 | 33.746 | 2534795 | 49.326 |
| Total | | 64252 | 100.000 | 5138853 | 100.000 |

Racemic **5l**

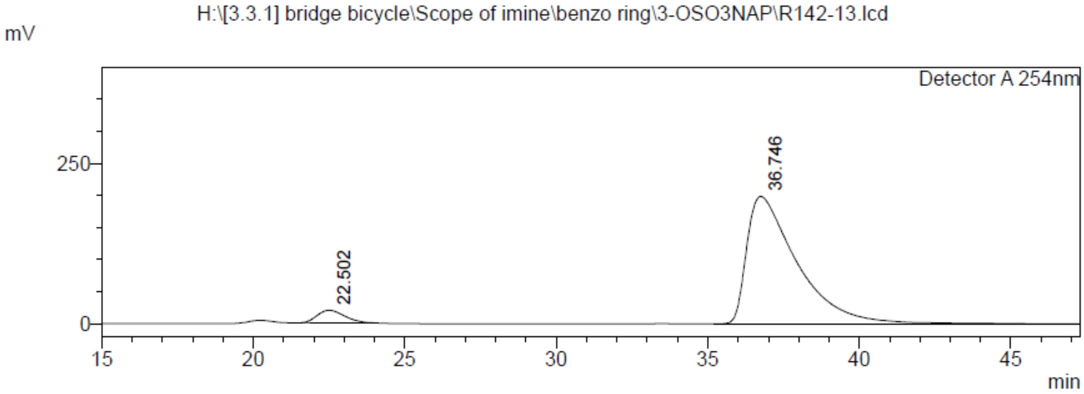


Detector A 254nm

| Peak# | Ret. Time | Height | Height% | Area | Area% |
|---|---|---|---|---|---|
| 1 | 22.502 | 19520 | 8.943 | 1232322 | 5.064 |
| 2 | 36.746 | 198762 | 91.057 | 23100749 | 94.936 |
| Total | | 218282 | 100.000 | 24333071 | 100.000 |

Enantiomerically enriched **5l**

**Methyl(6S,7S,13R)-8,13-dimethyl-7-(2-oxo-2-phenylethyl)-5,7,8,13-tetrahydro-6H-6,13-epiminonaphtho[2',1':4,5]cycloocta[1,2-b]indole-6-carboxylate(5m)**

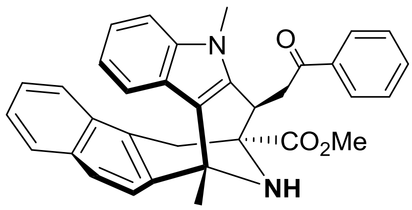


**5m**

A white solid; 43.6 mg, 85% yield; m.p. = 198.2-198.9 $^{o}$C, $[\alpha]^{25}_{D}$ = -18.97 (*c* 0.26 $CHCl_3$); *dr* >20:1; $^{1}$H NMR (400 MHz, $CDCl_3$) δ 8.03 (d, *J* = 7.2 Hz, 2H), 7.93 (d, *J* = 8.4 Hz, 1H), 7.81 (d, *J* = 8.4 Hz, 1H), 7.76 – 7.66 (m, 3H), 7.58 (t, *J* = 7.2 Hz, 1H), 7.48 (t, *J* = 7.6 Hz, 2H), 7.43 – 7.39 (m, 1H), 7.36 (t, *J* = 7.6 Hz, 1H), 7.18 (d, *J* = 8.0 Hz, 1H), 7.09 (td, *J* = 6.8, 0.8 Hz, 1H), 7.02 (t, *J* = 8.0 Hz, 1H), 4.36 (dd, *J* = 6.0, 2.8 Hz, 1H), 4.02 (dd, *J* = 18.0, 6.0 Hz, 1H), 3.99 (d, *J* = 18.4 Hz, 1H), 3.67 (s, 3H), 3.54 (s, 3H), 3.01 (dd, *J* = 18.0, 2.8 Hz, 1H), 2.86 (d, *J* = 18.4 Hz, 1H), 2.50 (s, 1H), 2.30 (s, 3H); $^{13}$C NMR (100 MHz, $CDCl_3$) δ 198.51, 175.33, 143.13, 137.76, 136.81, 135.44, 133.56, 132.12, 131.83, 128.85, 128.74, 128.61, 128.43, 126.38, 126.26, 125.05, 123.98, 122.18, 121.22, 120.84, 119.32, 119.20, 114.28, 109.30, 61.94, 53.98, 52.56, 41.47, 39.84, 33.50, 29.43, 25.27; HRMS (ESI) *m/z* calcd for $C_{34}H_{30}N_2O_3[M+H]^+$ = 515.2329, found = 515.2333; The ee value was 81%, $t_R$ (major) = 22.5 min, $t_R$ (minor) = 14.1 min (Chiralcel IB, λ = 254 nm, 10% i-PrOH/hexane, flow rate = 1.0 mL/min).

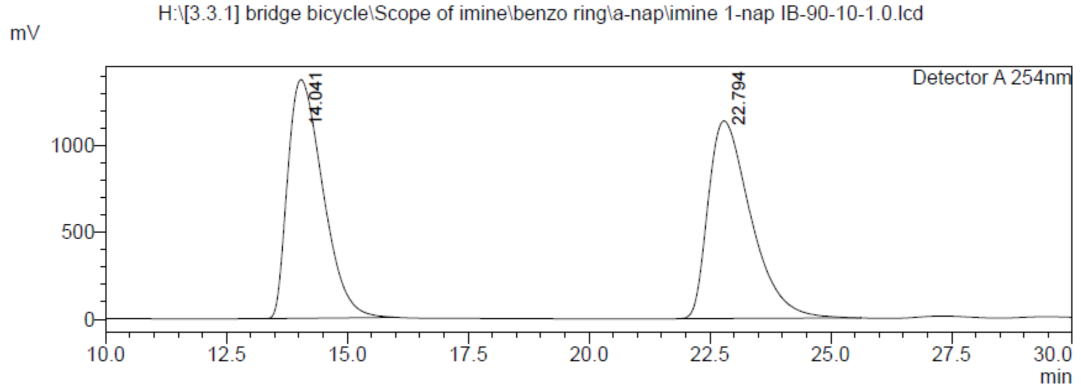


Detector A 254nm

| Peak# | Ret. Time | Height | Height% | Area | Area% |
|---|---|---|---|---|---|
| 1 | 14.041 | 1372397 | 54.715 | 69540639 | 49.845 |
| 2 | 22.794 | 1135871 | 45.285 | 69973870 | 50.155 |
| Total | | 2508268 | 100.000 | 139514509 | 100.000 |

Racemic **5m**

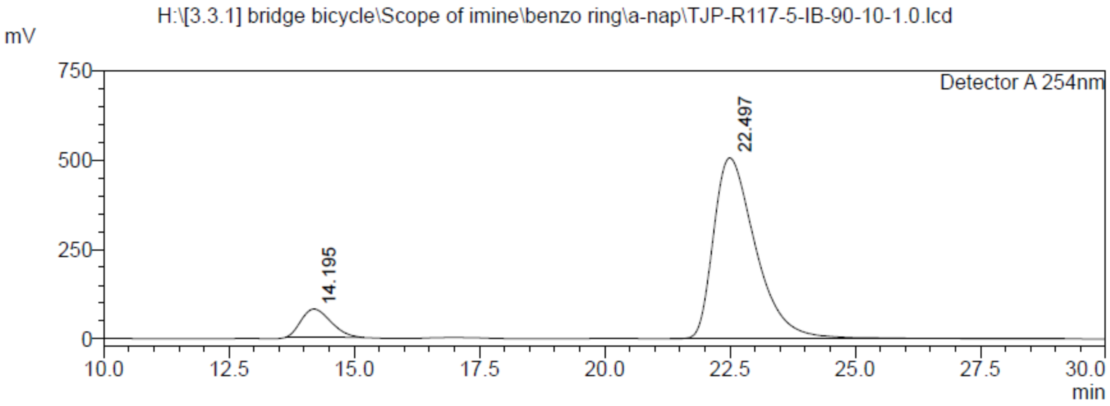


Detector A 254nm

| Peak# | Ret. Time | Height | Height% | Area | Area% |
|---|---|---|---|---|---|
| 1 | 14.195 | 78544 | 13.449 | 3247308 | 9.841 |
| 2 | 22.497 | 505456 | 86.551 | 29749096 | 90.159 |
| Total | | 584000 | 100.000 | 32996404 | 100.000 |

Enantiomerically enriched **5m**

**Ethyl(6S,7S,13R)-8,13-dimethyl-7-(2-oxo-2-phenylethyl)-5,7,8,13-tetrahydro-6H-6,13-epiminobenzo[4,5]cycloocta[1,2-b]indole-6-carboxylate(5n)**

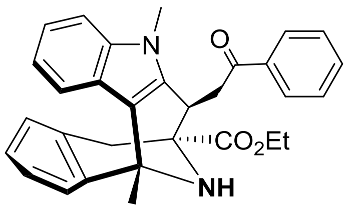


**5n**

A white solid; 43.5 mg, 91% yield; m.p. = 169.5–171.3 °C, $[\alpha]^{25}_{D}$ = -78.23 (*c* 0.31CHCl$_3$); *dr* >20:1; $^1$H NMR (400 MHz, CDCl$_3$) δ 8.00 (d, *J* = 7.2 Hz, 2H), 7.69 (d, *J* = 7.6 Hz, 1H), 7.63 (d, *J* = 8.0 Hz, 1H), 7.56 (t, *J* = 7.2 Hz, 1H), 7.45 (t, *J* = 8.0 Hz, 2H), 7.20 (t, *J* = 8.4 Hz, 2H), 7.12 (t, *J* = 7.2 Hz, 1H), 7.05 (q, *J* = 7.2 Hz, 2H), 6.94 (d, *J* = 7.2 Hz, 1H), 4.26 (dd, *J* = 5.6, 3.2 Hz, 1H), 4.04 (dq, *J* = 10.8, 7.2 Hz, 1H), 3.92 (dd, *J* = 18.0, 5.6 Hz, 1H), 3.84 (dq, *J* = 10.8, 7.2 Hz, 1H), 3.67 (s, 3H), 3.25 (d, *J* = 17.6 Hz, 1H), 3.05 (dd, *J* = 18.0, 3.2 Hz, 1H), 2.72 (d, *J* = 17.6 Hz, 1H), 2.16 (s, 3H), 1.17 (t, *J* = 7.2 Hz, 3H); $^{13}$C NMR (100 MHz, CDCl$_3$) δ 198.52, 174.71, 145.97, 137.83, 136.82, 136.54, 133.84, 133.50, 129.06, 128.80, 128.40, 126.27, 126.16, 124.18, 121.79, 121.13, 119.30, 112.51, 109.29, 61.77, 53.75, 41.38, 39.45, 37.47, 29.51, 25.11, 13.98; HRMS (ESI) *m/z* calcd for $C_{31}H_{30}N_2O_3[M+H]^+$ = 479.2329, found = 479.2331; The ee value was 81%, $t_R$ (major) = 9.3 min, $t_R$ (minor) = 15.6 min (Chiralcel IA, λ = 254 nm, 10% i-PrOH/hexane, flow rate = 1.0 mL/min).

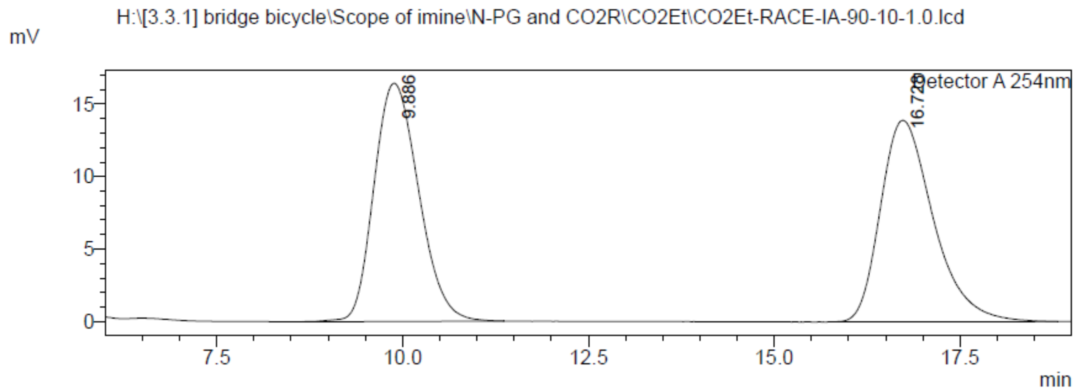


Detector A 254nm

| Peak# | Ret. Time | Height | Height% | Area | Area% |
|---|---|---|---|---|---|
| 1 | 9.886 | 16412 | 54.171 | 687180 | 49.981 |
| 2 | 16.728 | 13885 | 45.829 | 687702 | 50.019 |
| Total | | 30296 | 100.000 | 1374882 | 100.000 |

Racemic **5n**

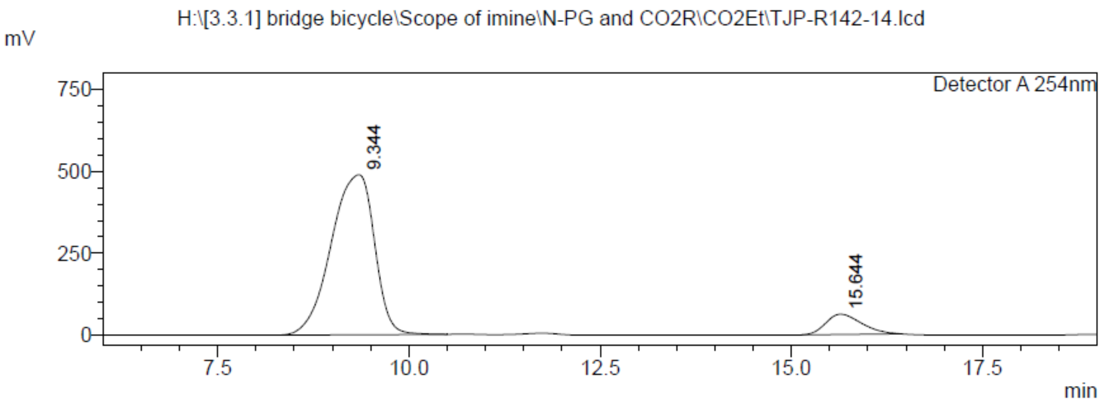


Detector A 254nm

| Peak# | Ret. Time | Height | Height% | Area | Area% |
|---|---|---|---|---|---|
| 1 | 9.344 | 487480 | 88.721 | 19181234 | 90.455 |
| 2 | 15.644 | 61975 | 11.279 | 2024095 | 9.545 |
| Total | | 549455 | 100.000 | 21205329 | 100.000 |

Enantiomerically enriched **5n**

**Isopropyl(6S,7S,13R)-8,13-dimethyl-7-(2-oxo-2-phenylethyl)-5,7,8,13-tetrahydro-6H-6,13-epiminobenzo[4,5]cycloocta[1,2-b]indole-6-carboxylate(5o)**

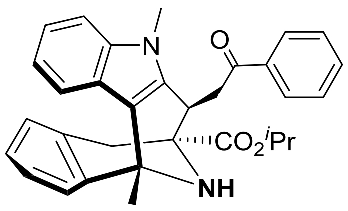


**5o**

A white solid; 39.8 mg, 81% yield; m.p. = 159.0–159.8 °C, $[\alpha]^{25}_{D}$ = -46.10 (*c* 0.33

$CHCl_3$); *dr* >20:1; $^1$H NMR (400 MHz, $CDCl_3$) δ 8.00 (d, *J* = 7.2 Hz, 2H), 7.69 (d, *J* = 6.8 Hz, 1H), 7.63 (d, *J* =8.0 Hz, 1H), 7.57 – 7.53 (m, 1H), 7.44 (t, *J* = 7.8 Hz, 2H), 7.22 – 7.18 (m, 2H), 7.11 (td, *J* = 6.8, 0.8 Hz, 1H), 7.06 – 7.01 (m, 2H), 6.94 (d, *J* = 7.6 Hz, 1H), 4.92 – 4.82 (m, 1H), 4.29 (t, *J* = 4.4 Hz, 1H), 3.81 (dd, *J* = 18.8, 4.8 Hz, 1H), 3.64 (s, 3H), 3.19 (d, *J* = 18.0 Hz, 1H), 3.18 (dd, *J* = 18.8, 4.0 Hz, 2H), 2.71 (d, *J* = 18.0 Hz, 1H), 2.16 (s, 3H), 1.25 (d, *J* = 6.2 Hz, 3H), 1.08 (d, *J* = 6.2 Hz, 3H); $^{13}$C NMR (100 MHz, $CDCl_3$) δ 198.25, 174.27, 146.05, 137.83, 136.91, 136.82, 133.91, 133.45, 129.08, 128.76, 128.40, 126.24, 126.15, 124.21, 121.77, 121.08, 119.29, 119.26, 112.35, 109.29, 69.67, 61.92, 53.74, 42.00, 38.69, 37.51, 29.60, 25.09, 21.67, 21.46; HRMS (ESI) *m/z* calcd for $C_{32}H_{32}N_2O_3[M+H]^+$ = 493.2486, found = 493.2484; The ee value was 68%, $t_R$ (major) =8.2 min, $t_R$ (minor) = 10.7 min (Chiralcel IA, λ = 254 nm, 10% i-PrOH/hexane, flow rate = 1.0 mL/min).

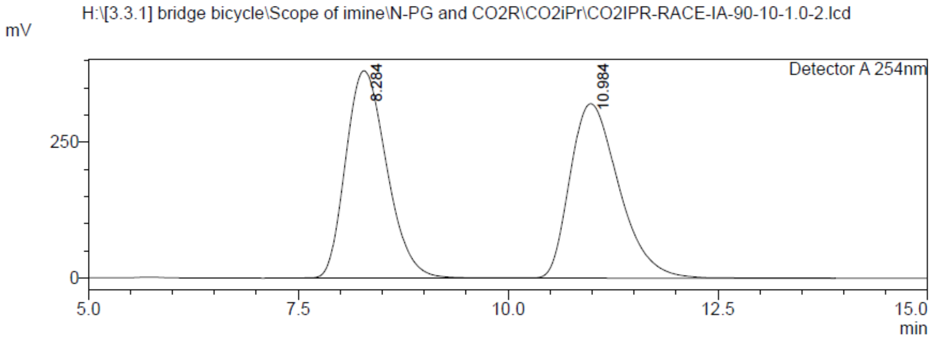


Detector A 254nm

| Peak# | Ret. Time | Height | Height% | Area | Area% |
|---|---|---|---|---|---|
| 1 | 8.284 | 382575 | 54.313 | 13054858 | 49.952 |
| 2 | 10.984 | 321810 | 45.687 | 13080200 | 50.048 |
| Total | | 704385 | 100.000 | 26135058 | 100.000 |

Racemic **5o**

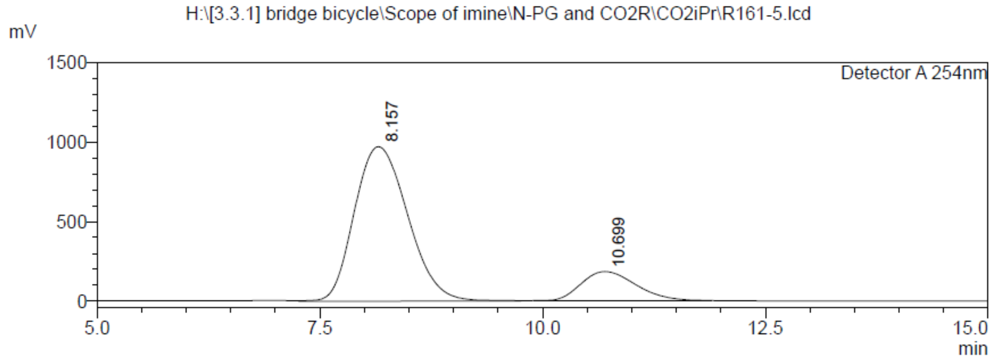


Detector A 254nm

| Peak# | Ret. Time | Height | Height% | Area | Area% |
|---|---|---|---|---|---|
| 1 | 8.157 | 970692 | 84.166 | 40536918 | 83.852 |
| 2 | 10.699 | 182615 | 15.834 | 7806310 | 16.148 |
| Total | | 1153307 | 100.000 | 48343228 | 100.000 |

Enantiomerically enriched **5o**

**Methyl(6S,7S,13R)-13-benzyl-8-methyl-7-(2-oxo-2-phenylethyl)-5,7,8,13-tetrahydro-6H-6,13-epiminobenzo[4,5]cycloocta[1,2-b]indole-6-carboxylate(5p)**

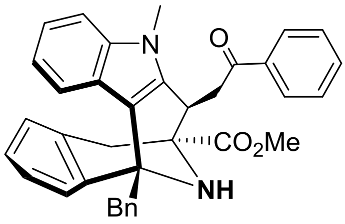

**5p**

A white solid; 48.1 mg, 89% yield; m.p. = 90.4–92.1 °C, $[\alpha]^{25}_{D}$ = -65.45 (*c* 0.68 $CHCl_3$); *dr* >20:1; $^1$H NMR (400 MHz, $CDCl_3$) δ 7.77 (dd, *J* = 14.8, 7.8 Hz, 2H), 7.61 (d, *J* = 7.2 Hz, 2H), 7.53 (t, *J* = 7.6 Hz, 1H), 7.39 (t, *J* = 8.0 Hz, 2H), 7.23 (t, *J* = 8.0 Hz, 1H), 7.20 – 7.15 (m, 4H), 7.14 –7.09 (m, 4H), 7.06 (t, *J* = 7.2 Hz, 1H), 6.95 (d, *J* = 7.6 Hz, 1H), 4.50 (d, *J* = 13.2 Hz, 1H), 3.97 (dd, *J* = 6.0, 4.0 Hz, 1H), 3.52 (s, 6H), 3.39 (d, *J* = 8.8 Hz, 1H), 3.36 (d, *J* = 18.0 Hz, 1H) 3.02 (dd, *J* = 18.0, 6.0 Hz, 1H), 2.61 (d, *J* = 18.0 Hz, 1H), 2.33 (dd, *J* = 18.0, 4.0 Hz, 1H); $^{13}$C NMR (100 MHz, $CDCl_3$) δ 198.12, 174.83, 145.92, 137.98, 137.86, 137.78, 136.47, 134.53, 133.38, 131.23, 129.24, 128.61, 128.37, 127.55, 126.17, 126.07, 126.01, 124.75, 121.12, 121.06, 119.58, 119.54, 109.60, 109.39, 61.09, 57.09 , 52.47, 41.69, 40.88, 38.68, 37.25, 29.37; HRMS (ESI) *m/z* calcd for $C_{36}H_{32}N_2O_3[M+H]^+$ = 541.2486, found = 541.2488; The ee value was 91%, $t_R$ (major) = 17.6 min, $t_R$ (minor) = 32.2 min (Chiralcel IA, λ = 254 nm, 5% i-PrOH/hexane, flow rate = 1.0 mL/min).

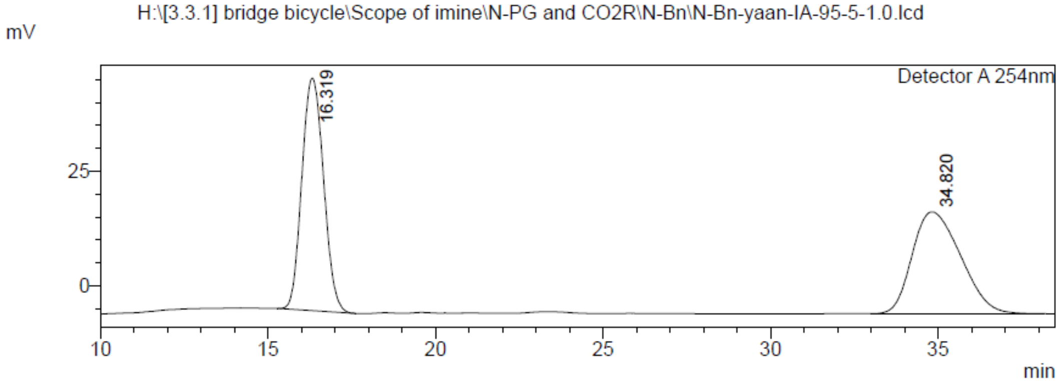


Detector A 254nm

| Peak# | Ret. Time | Height | Height% | Area | Area% |
|---|---|---|---|---|---|
| 1 | 16.319 | 50806 | 69.560 | 2295215 | 50.666 |
| 2 | 34.820 | 22233 | 30.440 | 2234898 | 49.334 |
| Total | | 73038 | 100.000 | 4530114 | 100.000 |

Racemic **5p**

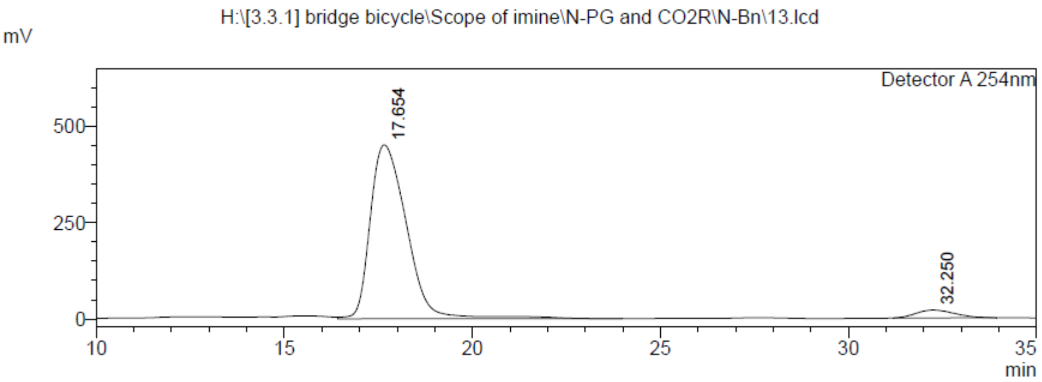


Detector A 254nm

| Peak# | Ret. Time | Height | Height% | Area | Area% |
|---|---|---|---|---|---|
| 1 | 17.654 | 451179 | 95.714 | 30495592 | 95.509 |
| 2 | 32.250 | 20205 | 4.286 | 1434000 | 4.491 |
| Total | | 471384 | 100.000 | 31929593 | 100.000 |

Enantiomerically enriched **5p**

**Methyl(6S,7S,13R)-13-ethyl-8-methyl-7-(2-oxo-2-phenylethyl)-5,7,8,13-tetrahydro-6H-6,13-epiminobenzo[4,5]cycloocta[1,2-b]indole-6-carboxylate(5q)**

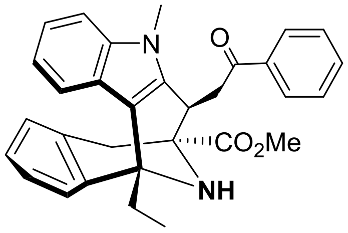


**5q**

A white solid; 43.9 mg, 92% yield; m.p. = 120.9–122.3 °C, $[\alpha]^{25}_{D}$ = -86.12 (*c* 0.89 $CHCl_3$); *dr* >20:1; $^1$H NMR (400 MHz, $CDCl_3$) δ 7.99 (d, *J* = 7.6 Hz, 2H), 7.65 (d, *J* = 7.6 Hz, 1H), 7.57 (t, *J* = 8.0 Hz, 2H), 7.47 (t, *J* = 7.6 Hz, 2H), 7.19 (t, *J* = 8.4 Hz, 2H), 7.12 (t, *J* = 7.2 Hz, 1H), 7.03 (q, *J* = 8.0 Hz, 2H), 6.94 (d, *J* = 7.6 Hz, 1H), 4.20 (dd, *J* = 6.0, 3.2 Hz, 1H), 3.97 (dd, *J* = 18.0, 6.0 Hz, 1H), 3.68 (s, 3H), 3.50 (s, 3H), 3.23 (d, *J* = 18.0 Hz, 1H), 2,99 (dd, *J* = 18.0, 3.2 Hz, 1H), 2,93 (dd, *J* = 9.6, 6.8 Hz, 1H), 2.69 (d, *J* = 18.0 Hz, 1H), 2.32 (dq, *J* = 14.2, 7.2 Hz, 1H), 1.06 (t, *J* = 7.2 Hz, 3H); $^{13}$C NMR (100 MHz, $CDCl_3$) δ 198.86, 175.23, 137.86, 137.40, 136.91, 134.34, 133.49, 131.42, 129.24, 128.84, 128.39, 127.26, 126.02, 124.19, 121.71, 121.09, 119.23, 119.19, 110.89, 109.21, 61.49, 56.73, 52.35, 41.18, 39.76, 37.35, 29.46, 28.73, 8.63; HRMS (ESI) *m/z* calcd for $C_{31}H_{30}N_2O_3[M+H]^+$ = 479.2329, found = 479.2326; The ee value was 90%, $t_R$ (major) = 10.3 min, $t_R$ (minor) = 18.4 min (Chiralcel IA, λ = 254 nm, 10% i-PrOH/hexane, flow rate = 1.0 mL/min).

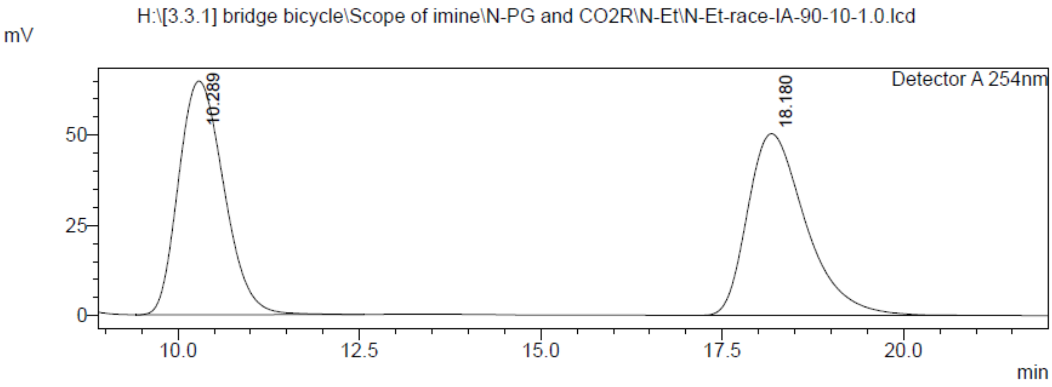


Detector A 254nm

| Peak# | Ret. Time | Height | Height% | Area | Area% |
|---|---|---|---|---|---|
| 1 | 10.289 | 64775 | 56.247 | 2819611 | 50.020 |
| 2 | 18.180 | 50386 | 43.753 | 2817359 | 49.980 |
| Total | | 115162 | 100.000 | 5636970 | 100.000 |

Racemic **5q**

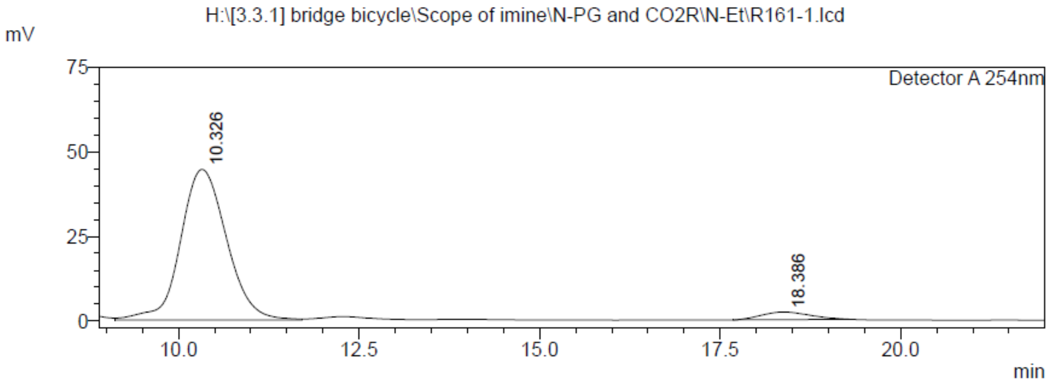


Detector A 254nm

| Peak# | Ret. Time | Height | Height% | Area | Area% |
|---|---|---|---|---|---|
| 1 | 10.326 | 44501 | 95.233 | 1981283 | 94.920 |
| 2 | 18.386 | 2228 | 4.767 | 106046 | 5.080 |
| Total | | 46728 | 100.000 | 2087329 | 100.000 |

Enantiomerically enriched **5q**

**Methyl(6S,7S,13R)-8-methyl-7-(2-oxo-2-phenylethyl)-13-pentyl-5,7,8,13-tetrahydro-6H-6,13-epiminobenzo[4,5]cycloocta[1,2-b]indole-6-carboxylate (5r)**

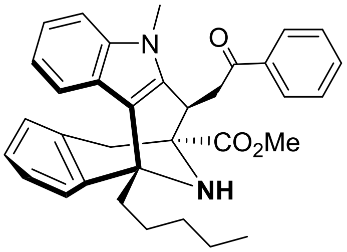


**5r**

A yellow liquid; 46.8 mg, 90% yield; $[\alpha]^{25}_{D}$ = -86.12 (*c* 0.89 $CHCl_3$); *dr* >20:1; $^1H$ NMR (400 MHz, $CDCl_3$) δ 7.99 (d, *J* = 8.0 Hz, 2H), 7.66 (d, *J* = 7.6 Hz, 1H), 7.58 (dd,

$J$ = 15.8, 7.6 Hz, 2H), 7.47 (t, $J$ = 7.2 Hz, 2H), 7.19 (t, $J$ = 7.2 Hz, 2H), 7.12 (t, $J$ = 7.6 Hz, 1H), 7.03 (q, $J$ = 6.8 Hz, 2H), 6.93 (d, $J$ = 7.2 Hz, 1H), 4.20 (s, 1H), 3.93 (dd, $J$ = 17.6, 5.6 Hz, 1H), 3.68 (s, 3H), 3.51 (s, 3H), 3.23 (d, $J$ = 17.6 Hz, 1H), 3.03 (dd, $J$ = 17.6, 2.0 Hz, 1H), 2.88 (td, $J$ = 13.2, 4.0 Hz, 1H), 2.68 (d, $J$ = 17.6 Hz, 1H), 2.28 (t, $J$ = 14.0 Hz, 1H), 1.79 – 1.76 (m, 1H), 1.46 – 1.28 (m, 5H), 0.91 (t, $J$ = 6.8 Hz, 3H); $^{13}$C NMR (100 MHz, $CDCl_3$) δ 198.93, 175.25, 145.52, 137.87, 137.05, 136.96, 134.25, 133.48, 129.24, 128.83, 128.41, 126.00, 124.24, 121.76, 121.07, 119.26, 119.23, 111.43, 109.20, 61.53, 56.52, 52.37, 41.24, 39.83, 37.30, 36.24, 32.74, 29.47, 23.69, 22.88, 14.37; HRMS (ESI) *m/z* calcd for $C_{34}H_{36}N_2O_3[M+H]^+$ = 521.2801, found = 521.2800; The ee value was 92%, $t_R$ (major) = 6.9 min, $t_R$ (minor) = 11.3 min (Chiralcel IA, λ = 254 nm, 10% i-PrOH/hexane, flow rate = 1.0 mL/min).

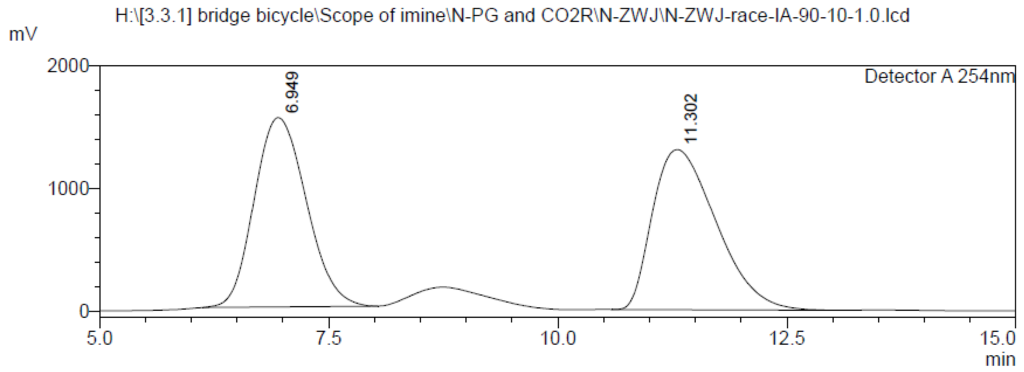


Detector A 254nm

| Peak# | Ret. Time | Height | Height% | Area | Area% |
|---|---|---|---|---|---|
| 1 | 6.949 | 1542267 | 54.188 | 61048649 | 49.184 |
| 2 | 11.302 | 1303898 | 45.812 | 63074217 | 50.816 |
| Total | | 2846165 | 100.000 | 124122865 | 100.000 |

Racemic **5r**

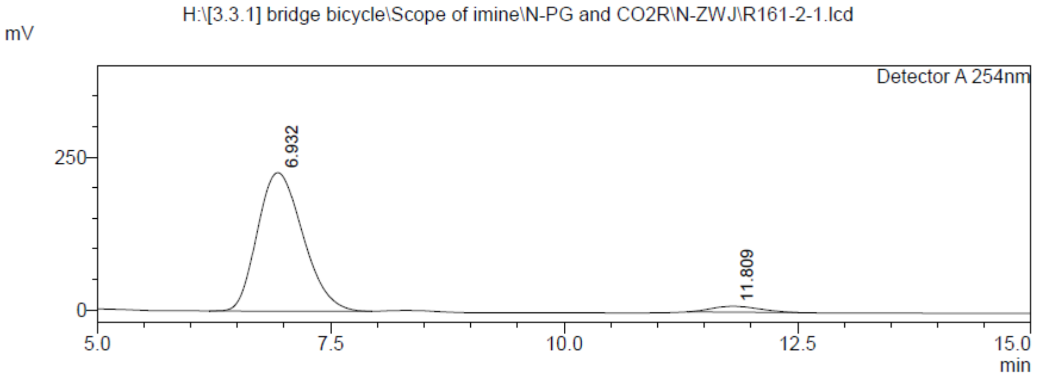


Detector A 254nm

| Peak# | Ret. Time | Height | Height% | Area | Area% |
|---|---|---|---|---|---|
| 1 | 6.932 | 226710 | 95.936 | 7714434 | 95.786 |
| 2 | 11.809 | 9603 | 4.064 | 339424 | 4.214 |
| Total | | 236313 | 100.000 | 8053858 | 100.000 |

Enantiomerically enriched **5r**

**Methyl(6S,7S,13R)-13-heptyl-8-methyl-7-(2-oxo-2-phenylethyl)-5,7,8,13-tetrahydro-6H-6,13-epiminobenzo[4,5]cycloocta[1,2-b]indole-6-carboxylate (5s)**

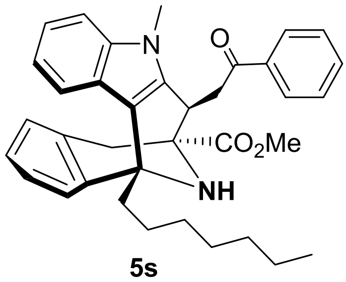


**5s**

A yellow liquid; 49.4 mg, 90% yield; $[\alpha]^{25}_{D}$ = -47.16 (*c* 0.51 $CHCl_3$); *dr* >20:1; $^1H$ NMR (400 MHz, $CDCl_3$) δ 7.98 (d, *J* = 8.0 Hz, 2H), 7.65 (d, *J* = 7.6 Hz, 1H), 7.57 (dd, *J* = 12.0, 8.0 Hz, 2H), 7.46 (t, *J* = 7.6 Hz, 2H), 7.18 (t, *J* = 7.2 Hz, 2H), 7.11 (t, *J* = 7.6 Hz, 1H), 7.02 (q, *J* = 7.2 Hz, 2H), 6.92 (d, *J* = 7.2 Hz, 1H), 4.18 (s, 1H), 3.91 (dd, *J* = 18.0, 5.6 Hz, 1H), 3.67 (s, 3H), 3.50 (s, 3H), 3.22 (d, *J* = 17.6 Hz, 1H), 3.01 (dd, *J* = 18.0, 2.0 Hz, 1H), 2.86 (td, *J* = 12.8, 3.6 Hz, 1H), 2.67 (d, *J* = 17.6 Hz, 1H), 2.26 (t, *J* = 11.2 Hz, 1H), 1.81 – 1.71 (m, 1H), 1.45 – 1.42 (m, 2H), 1.34 – 1.28 (m, 9H), 0.88 (t, *J* = 6.0 Hz, 3H); $^{13}C$ NMR (100 MHz, $CDCl_3$) δ 198.94, 175.27, 145.54, 137.87, 137.06, 136.97, 134.26, 133.49, 129.25, 128.84, 128.42, 126.01, 124.25, 121.77, 121.08, 119.28, 119.24, 111.44, 109.20, 61.54, 56.53, 52.38, 41.25, 39.83, 37.31, 36.27, 32.15, 30.49, 29.53, 29.46, 23.98, 22.87, 14.28; HRMS (ESI) *m/z* calcd for $C_{36}H_{40}N_2O_3[M+H]^+$ = 549.3114, found = 549.3112; The ee value was 90%, $t_R$ (major) = 6.9 min, $t_R$ (minor) = 11.9 min (Chiralcel IA, λ = 254 nm, 10% i-PrOH/hexane, flow rate = 1.0 mL/min).

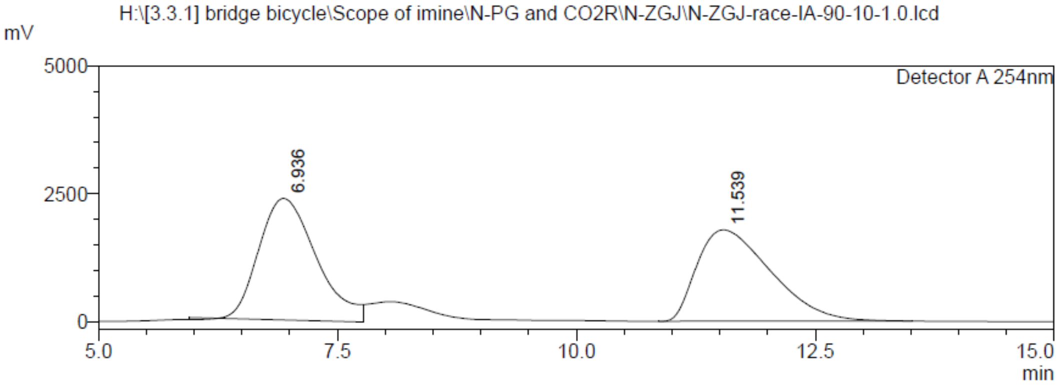


Detector A 254nm

| Peak# | Ret. Time | Height | Height% | Area | Area% |
|---|---|---|---|---|---|
| 1 | 6.936 | 2378787 | 57.123 | 99402769 | 50.855 |
| 2 | 11.539 | 1785570 | 42.877 | 96058525 | 49.145 |
| Total | | 4164357 | 100.000 | 195461294 | 100.000 |

Racemic **5s**

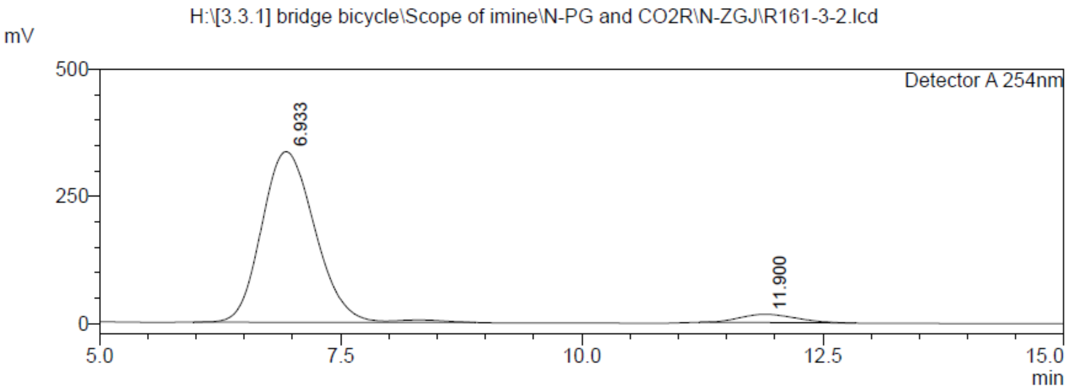


Detector A 254nm

| Peak# | Ret. Time | Height | Height% | Area | Area% |
|---|---|---|---|---|---|
| 1 | 6.933 | 335346 | 95.366 | 12990042 | 95.133 |
| 2 | 11.900 | 16293 | 4.634 | 664609 | 4.867 |
| Total | | 351640 | 100.000 | 13654651 | 100.000 |

Enantiomerically enriched **5s**

**Methyl(6S,7S,13R)-8-methyl-7-(2-oxo-2-phenylethyl)-13-undecyl-5,7,8,13-tetrahydro-6H-6,13-epiminobenzo[4,5]cycloocta[1,2-b]indole-6-carboxylate (5t)**

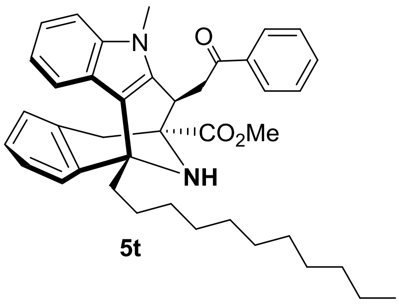


**5t**

A yellow liquid; 55.0 mg, 91% yield; $[\alpha]^{25}_{D}$ = -56.19 (*c* 0.62 $CHCl_3$); *dr* >20:1; $^1H$ NMR (400 MHz, $CDCl_3$) δ 7.98 (d, *J* = 7.6 Hz, 2H), 7.64 (d, *J* = 7.6 Hz, 1H), 7.57 (dd, *J* = 12.0, 8.0 Hz, 2H), 7.45 (t, *J* = 7.6 Hz, 2H), 7.18 (t, *J* = 7.6 Hz, 2H), 7.10 (t, *J* = 7.6 Hz, 1H), 7.02 (q, *J* = 7.2 Hz, 2H), 6.92 (d, *J* = 7.6 Hz, 1H), 4.18 (dd, *J* = 6.0, 3.2 Hz, 1H), 3.91 (dd, *J* = 17.6, 6.0 Hz, 1H), 3.67 (s, 3H), 3.50 (s, 3H), 3.21(d, *J* = 18.0 Hz, 1H), 3.01 (dd, *J* = 17.6, 3.2 Hz, 1H), 2.86 (td, *J* = 13.2, 4.4 Hz, 1H), 2.66 (d, *J* = 18.0 Hz, 1H), 2.23 – 2.22 (m, 1H), 1.77 – 1.69 (m, 2H), 1.42 (dd, *J* = 13.6, 7.6 Hz, 2H), 1.25 (s, 14H), 0.88 (t, *J* = 6.8 Hz, 3H); $^{13}C$ NMR (100 MHz, $CDCl_3$) δ 198.95, 175.26, 137.87, 137.05, 136.98, 134.26, 133.48, 129.25, 128.83, 128.42, 126.00, 125.74, 124.25, 121.77, 121.08, 119.28, 119.24, 111.45, 109.20, 61.55, 56.54 , 52.38, 41.25, 39.84, 37.32, 36.28, 32.08, 30.54, 29.95, 29.88, 29.81, 29.52, 29.49, 23.99, 22.84, 14.27, 1.17; HRMS (ESI) *m/z* calcd for $C_{40}H_{48}N_2O_3[M+H]^+$ = 605.3738, found =

605.3738; The ee value was 90%, $t_R$ (major) = 12.4 min, $t_R$ (minor) = 19.1 min (Chiralcel IA, λ = 254 nm, 10% i-PrOH/hexane, flow rate = 0.5 mL/min).

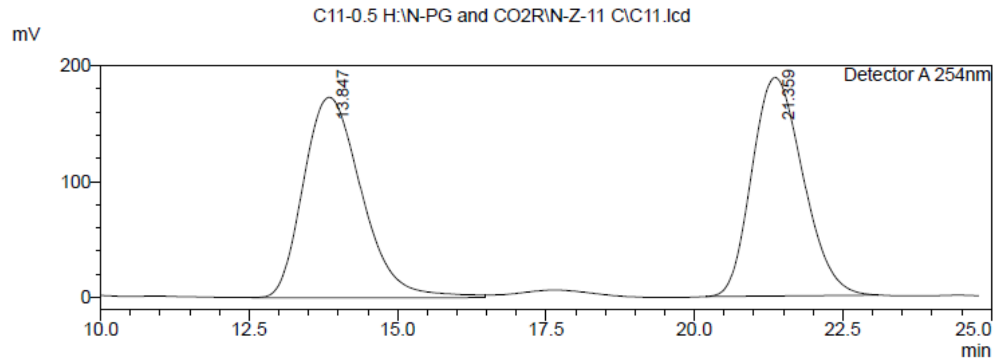


Detector A 254nm

| Peak# | Ret. Time | Height | Height% | Area | Area% |
|---|---|---|---|---|---|
| 1 | 13.847 | 172815 | 47.854 | 11811538 | 51.119 |
| 2 | 21.359 | 188312 | 52.146 | 11294316 | 48.881 |
| Total | | 361126 | 100.000 | 23105854 | 100.000 |

Racemic **5t**

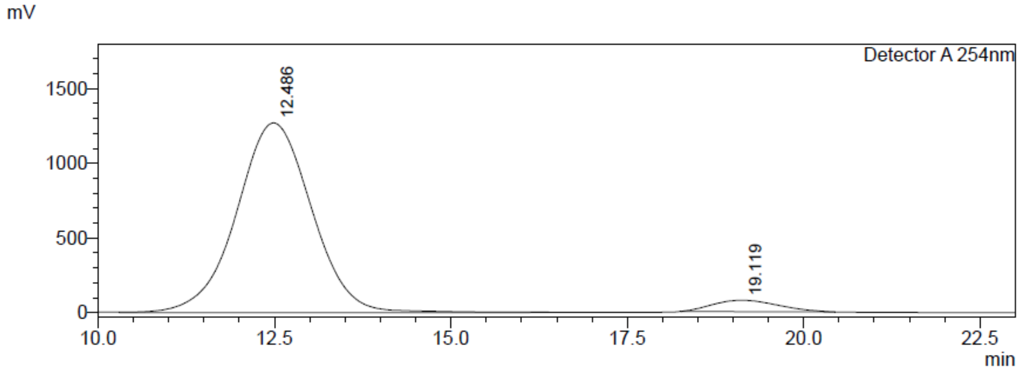


**<Peak Table>**

Detector A 254nm

| Peak# | Ret. Time | Height | Height% | Conc. | Area | Area% |
|---|---|---|---|---|---|---|
| 1 | 12.486 | 1270155 | 94.342 | 95.062 | 94517821 | 95.062 |
| 2 | 19.119 | 76171 | 5.658 | 4.938 | 4909404 | 4.938 |
| Total | | 1346326 | 100.000 | | 99427225 | 100.000 |

Enantiomerically enriched **5t**

# 7. Late-stage diversification

## A). Preparation of the substrates.

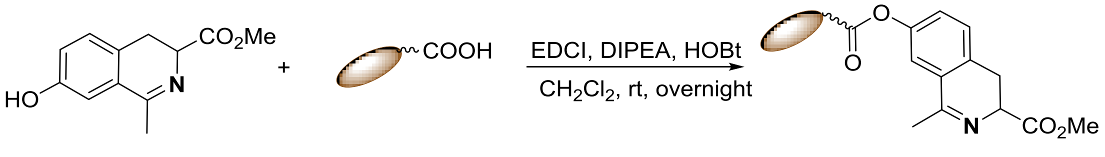

To the compound methyl 7-hydroxy-1-methyl-3,4-dihydroisoquinoline-3-carboxylate (110.0mg,0.5mmol), indicated acid (0.6 mmol) and EDCI (115.0 mg, 0.6 mmol), DIPEA (129.2 mg, 1.0 mmol), HOBT (91.3 mg, 0.6 mmol) in $CH_2Cl_2$ (5 mL), the mixture was stirred at room temperature overnight, TLC show the reaction was completed, then $H_2O$ (5 mL) was added and extracted with $CH_2Cl_2$ three times. The combined organic layers were dried over $Na_2SO_4$. After evaporation of solvent, the mixture was purified by flash chromato graphy on silica gel (petroleum ether/ethyl acetate = 2:1) to afford the product **2a-1~2a-4** .

## A. Characterization of the target substrates

**Methyl-7-((2-(4-(4-chlorobenzoyl)phenoxy)-2-methylpropanoyl)oxy)-1-methyl-3,4-dihydroisoquinoline-3-carboxylate (2a-1)**

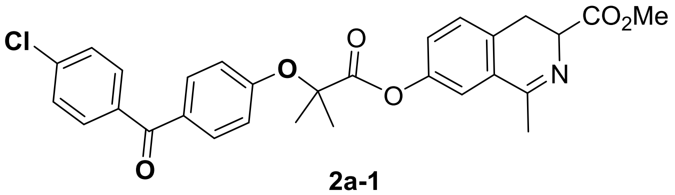

**2a-1**

A white solid; 78% yield, $^1$H NMR (400 MHz, $CDCl_3$) δ 7.80 – 7.76 (m, 2H), 7.71 – 7.68 (m, 2H), 7.45 – 7.42 (m, 2H), 7.21 (d, *J* = 8.0 Hz, 1H), 7.09 (d, *J* = 2.4 Hz, 1H), 7.01 – 6.98 (m,3H), 4.20 (ddd, *J* = 13.2, 6.0, 2.0 Hz, 1H), 3.80 (s, 3H), 3.00 – 2.87 (m, 2H), 2.38 (d, *J* = 2.0 Hz, 3H), 1.83 (s, 6H); $^{13}$C NMR (100 MHz, $CDCl_3$) δ 193.96, 172.93, 172.38, 164.69, 159.43, 149.43, 138.45, 136.18, 133.41, 132.09, 131.11, 130.80, 130.10, 128.70, 128.65, 123.79, 118.45, 117.36, 79.47, 59.81, 52.48, 27.72, 25.43 (d, *J* = 6.0 Hz), 23.23; HRMS (ESI) *m/z* calcd for $C_{29}H_{26}ClNO_6[M+H]^+$ = 520.1521, found = 520.1521.

**Methyl-7-((5-(2,5-dimethylphenoxy)-2,2-dimethylpentanoyl)oxy)-1-methyl-3,4-dihydroisoquinoline-3-carboxylate (2a-2)**

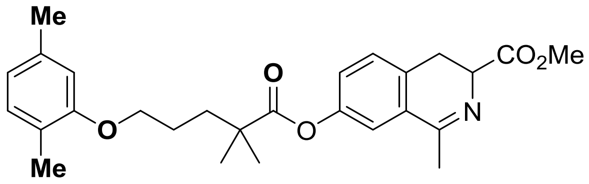

**2a-2**

A white solid; 84% yield, $^{1}H$ NMR (400 MHz, $CDCl_3$) δ 7.22 (d, *J* = 8.4 Hz, 1H), 7.19 (d, *J* = 2.0Hz, 1H), 7.05 (dd, *J* = 8.0, 2.4 Hz, 1H), 7.00 (d, *J* = 7.2 Hz, 1H), 6.66 (d, *J* = 7.6 Hz, 1H), 6.63 (s, 1H), 4.21 (ddd, *J* = 13.2, 6.0, 2.0 Hz, 1H), 3.99 (t, *J* = 4.8 Hz, 2H), 3.83 (s, 3H), 3.02 – 2.89 (m, 2H), 2.43 (d, *J* = 2.0 Hz, 3H), 2.30 (s, 3H), 2.17 (s, 3H), 1.94 – 1.85 (m, 4H), 1.39 (s, 6H); $^{13}C$ NMR (100 MHz, $CDCl_3$) δ 176.49, 173.29, 165.20, 156.95, 150.21, 136.61, 132.94, 130.48, 130.18, 128.64, 124.36, 123.67, 120.94, 119.01, 112.08, 67.84, 60.10, 52.64, 42.59, 37.21, 27.95, 25.40, 25.23 (d, *J* = 3.6 Hz), 23.47, 21.50, 15.89; HRMS (ESI) *m/z* calcd for $C_{27}H_{33}NO_5[M+H]^+$ = 452.2431, found = 452.2426.

**Methyl-1-methyl-7-((4-((3R,8R,9R,10S,13R,14R,17R)-3,9,14-trihydroxy-10,13-dimethylhexadecahydro-1H-cyclopenta[a]phenanthren-17-yl)pentanoyl)oxy)-3,4-dihydroisoquinoline-3-carboxylate (2a-3)**

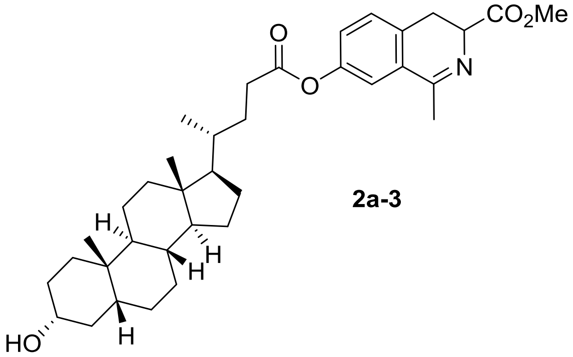

**2a-3**

A white solid; 75% yield, $^{1}H$ NMR (400 MHz, $CDCl_3$) δ 7.24 – 7.22 (m, 2H), 7.10 (dd, *J* = 8.4, 2.4 Hz, 1H), 4.23 – 4.18 (m, 1H), 3.82 (s, 3H), 3.66 – 3.58 (m, 1H), 3.01 – 2.88 (m, 2H), 2.62 (ddd, *J* = 14.8, 9.8, 4.8 Hz, 1H), 2.52 – 2.44 (m, 1H), 2.43 (d, *J* = 2.0 Hz, 3H), 1.99 – 1.83 (m, 4H), 1.82 – 1.74 (m, 2H), 1.61 – 1.56 (m, 2H), 1.53 – 1.46 (m, 3H), 1.45 – 1.30 (m, 8H), 1.19 – 1.03 (m, 6H), 0.98(d, *J* = 6.4 Hz, 3H), 0.91 (s, 3H), 0.87 – 0.82 (m, 1H), 0.66 (s, 3H); $^{13}C$ NMR (100 MHz, $CDCl_3$) δ 173.31, 172.90, 165.20, 149.97, 132.98, 130.19, 128.68, 124.47, 119.14, 71.93, 60.10, 56.65, 56.07, 52.66, 42.92, 42.21, 40.56, 40.32, 36.57, 35.98, 35.49, 34.70, 31.44, 31.04,

30.66, 28.39, 27.95, 27.31, 26.54, 24.33, 23.49,23.46, 20.95, 18.46, 12.20; HRMS (ESI) *m/z* calcd for $C_{36}H_{51}NO_5[M+H]^+$ = 578.3840, found = 578.3840.

**Methyl7-(((4R)-4-((10S,13R,17R)-10,13-dimethyl-3,7,12-trioxohexadecahydro-1H-cyclopenta[a]phenanthren-17-yl)pentanoyl)oxy)-1-methyl-3,4-dihydroisoquinoline-3-carboxylate (2a-4)**

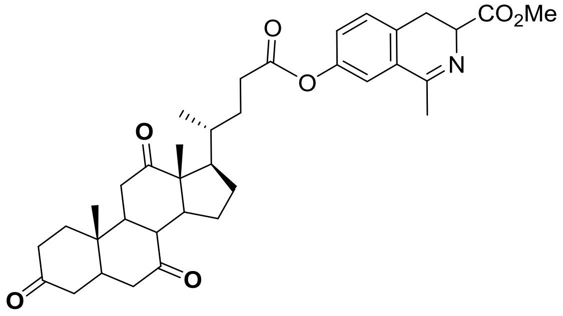


**2a-4**

A white solid; 77% yield, $^1$H NMR (400 MHz, $CDCl_3$) δ 7.21 – 7.19 (m, 2H), 7.07 (dd, *J* = 8.0, 2.0 Hz, 1H), 4.17 (ddd, *J* = 13.4, 6.0, 2.0 Hz, 1H), 3.78 (s, 3H), 2.98 – 2.79 (m, 5H), 2.63 (ddd, *J* = 14.8, 9.2, 5.2 Hz, 1H), 2.54 – 2.46 (m, 1H), 2.39 (d, *J* = 2.0 Hz, 3H), 2.36 – 2.25 (m, 4H), 2.17 (dd, *J* = 15.6, 4.4 Hz, 2H), 2.11 (dd, *J* = 12.8, 5.6 Hz, 2H), 2.06 – 1.90 (m, 6H), 1.82 (td, *J* = 11.6, 7.6 Hz, 1H), 1.60 (dd, *J* = 14.4, 4.8 Hz, 1H), 1.53 – 1.47 (m, 1H), 1.37 (s, 3H), 1.29 – 1.19 (m, 2H), 1.05 (s, 3H), 0.88 (d, *J* = 6.8 Hz, 3H); $^{13}$C NMR (100 MHz, $CDCl_3$) δ 211.92, 209.02, 208.68, 173.18, 172.59, 165.04, 149.82, 132.90, 130.07, 128.58, 124.36, 119.02, 60.00, 56.91, 52.55, 51.80, 48.98, 46.81, 45.63, 45.54, 44.99, 42.79, 38.66, 36.48, 36.03, 35.50, 35.27, 31.48, 30.33, 27.83, 27.69, 25.14, 23.38, 21.91, 18.71, 11.87; HRMS (ESI) *m/z* calcd for $C_{36}H_{45}NO_7[M+H]^+$ = 604.3269., found = 604.3268.

**B). Late-stage diversification**

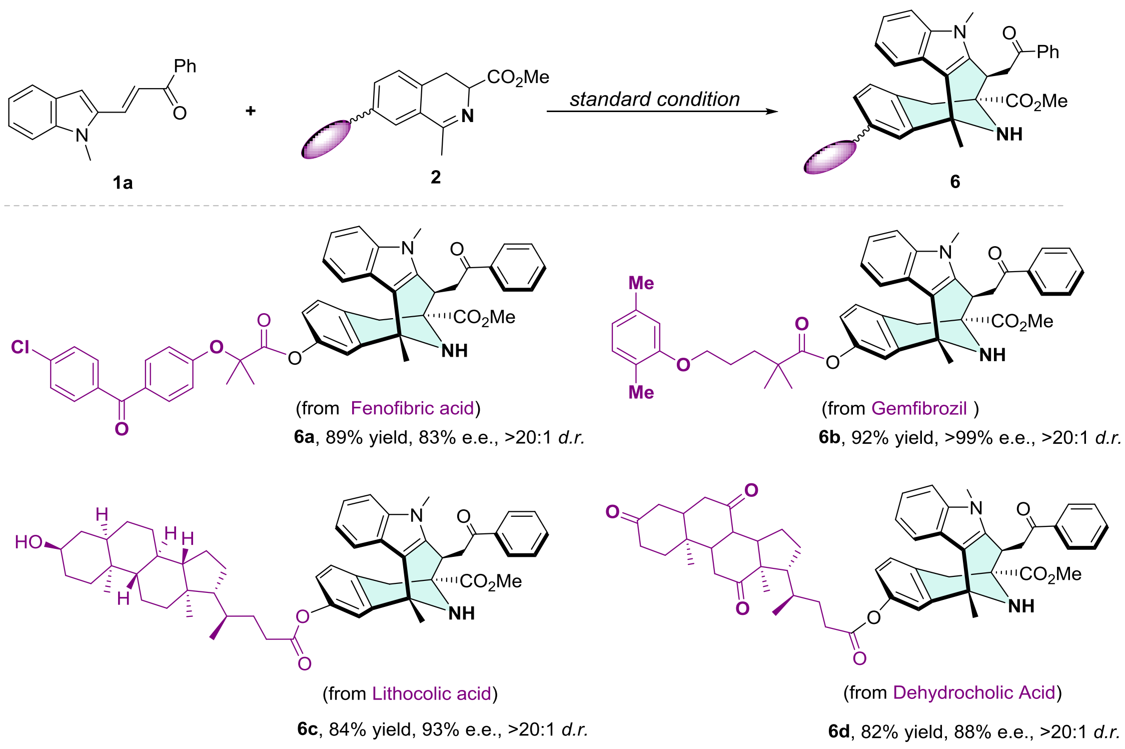


## A. Characterization of the products

**Methyl(6S,7S,13R)-2-((2-(4-(4-chlorobenzoyl)phenoxy)-2-methylpropanoyl)oxy)-8,13-dimethyl-7-(2-oxo-2-phenylethyl)-5,7,8,13-tetrahydro-6H-6,13-epiminobenzo[4,5]cycloocta[1,2-b]indole-6-carboxylate (6a)**

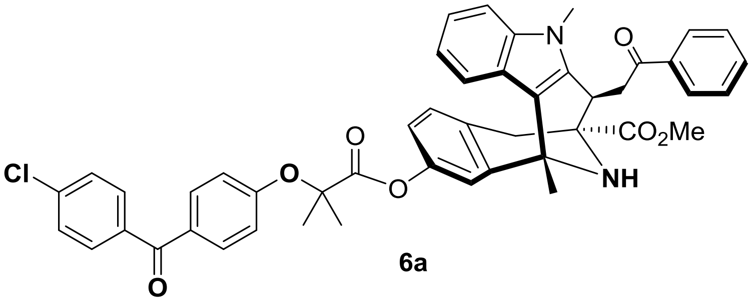


**6a**

A white solid; 69.4 mg, 89% yield; m.p. = 89.5-90.7 °C, $[\alpha]^{25}_D$ = -29.68 (*c* 0.29 $CHCl_3$); *dr* >20:1; $^1$H NMR (400 MHz, $CDCl_3$) δ 7.98 (d, *J* = 7.2 Hz, 2H), 7.83 (d, *J* = 8.8 Hz, 2H), 7.72 (d, *J* = 8.8 Hz, 2H), 7.56 (t, *J* = 7.2 Hz, 1H), 7.58 – 7.43 (m, 5H), 7.25 (d, *J* = 2.4 Hz, 1H), 7.21 (d, *J* = 8.0 Hz, 1H), 7.11 (t, *J* = 7.6 Hz, 1H), 7.03 (d, *J* = 8.8 Hz, 2H), 6.98 (t, *J* = 7.2 Hz, 1H), 6.92 (d, *J* = 8.4 Hz, 1H), 6.70 (dd, *J* = 8.0, 2.4 Hz, 1H), 4.20 (dd, *J* = 6.0, 2.8 Hz, 1H), 3.94 (dd, *J* = 18.0, 6.0 Hz, 1H), 3.67 (s, 3H), 3.48 (s, 3H), 3.25 (d, *J* = 17.6 Hz, 1H), 2.98 (dd, *J* = 18.0, 2.8 Hz, 1H), 2.65 (d, *J* =

17.6 Hz, 1H), 2.07 (s, 3H), 1.86 (d, $J$ = 5.2 Hz, 6H); $^{13}$C NMR (100 MHz, $CDCl_3$) δ 198.54, 194.28, 174.83, 172.54, 159.78, 148.67, 147.36, 138.57, 137.80, 136.77, 136.49, 136.43, 133.56, 132.28, 131.81, 131.31, 130.89, 129.84, 128.85, 128.73, 128.40, 123.94, 121.33, 119.47, 119.06, 118.79, 117.62, 114.68, 111.74, 109.34, 79.77, 61.66, 53.73, 52.49, 41.01, 39.83, 36.83, 29.47, 25.64(d, $J$ = 13.0 Hz), 24.97; HRMS (ESI) $m/z$ calcd for $C_{47}H_{41}ClN_2O_7[M+H]^+$ = 781.2675, found = 781.2664; The ee value was 83%, $t_R$ (major) = 19.4 min, $t_R$ (minor) = 14.1 min (Chiralcel IB, λ = 254 nm, 20% i-PrOH/hexane, flow rate = 1.0 mL/min).

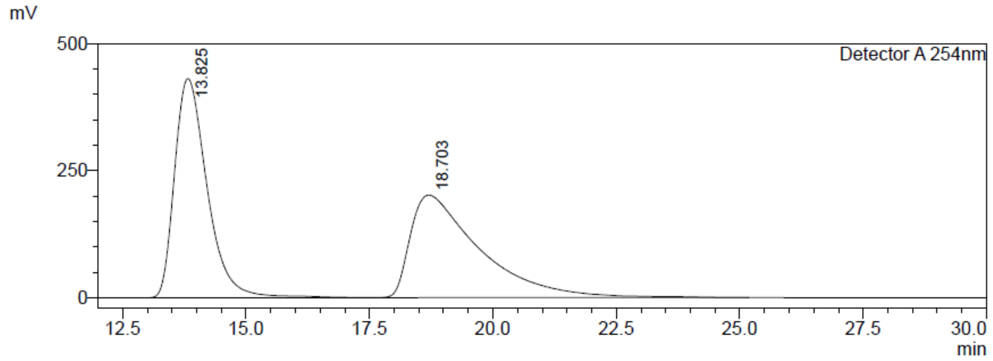


<Peak Table>

Detector A 254nm

| Peak# | Ret. Time | Height | Height% | Conc. | Area | Area% |
|---|---|---|---|---|---|---|
| 1 | 13.825 | 430839 | 68.125 | 50.129 | 19815765 | 50.129 |
| 2 | 18.703 | 201588 | 31.875 | 49.871 | 19713959 | 49.871 |
| Total | | 632427 | 100.000 | | 39529724 | 100.000 |

Racemic **6a**

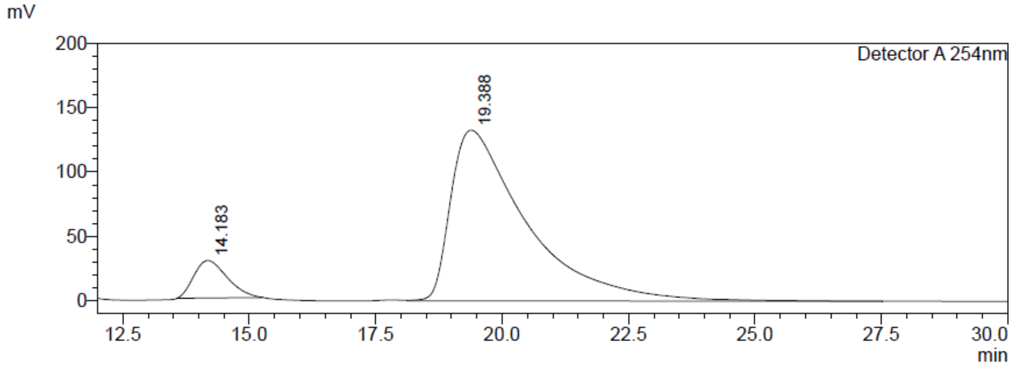


<Peak Table>

Detector A 254nm

| Peak# | Ret. Time | Height | Height% | Conc. | Area | Area% |
|---|---|---|---|---|---|---|
| 1 | 14.183 | 29007 | 17.956 | 8.757 | 1328900 | 8.757 |
| 2 | 19.388 | 132539 | 82.044 | 91.243 | 13845587 | 91.243 |
| Total | | 161546 | 100.000 | | 15174487 | 100.000 |

Enantiomerically enriched **6a**

**Methyl(6S,7S,13R)-2-((5-(2,5-dimethylphenoxy)-2,2-dimethylpentanoyl)oxy)-8,13**

**-dimethyl-7-(2-oxo-2-phenylethyl)-5,7,8,13-tetrahydro-6H-6,13-epiminobenzo[4,5]cycloocta[1,2-b]indole-6-carboxylate (6b)**

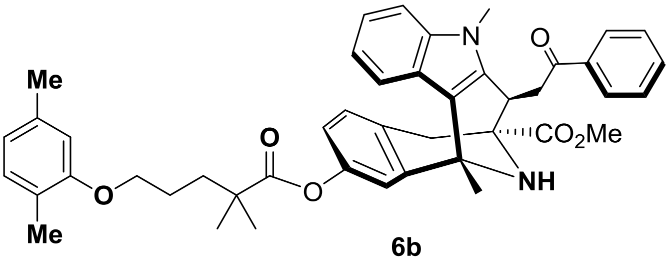


**6b**

A white solid; 65.5 mg, 92% yield; m.p. = 69.1-70.4 °C, $[\alpha]^{25}_{D}$ = -34.47 (*c* 0.35 $CHCl_3$); *dr* >20:1; $^1H$ NMR (400 MHz, $CDCl_3$) δ 7.99 (d, *J* = 7.2 Hz, 2H), 7.56 (dd, *J* = 10.4, 8.0 Hz, 2H), 7.45 (t, *J* = 7.6 Hz, 2H), 7.35 (d, *J* = 2.0 Hz, 1H), 7.21 (d, *J* = 8.0 Hz, 1H), 7.13 (t, *J* = 7.2 Hz, 1H), 7.03 (dd, *J* = 15.2, 7.2 Hz, 2H), 6.91 (d, *J* = 8.4 Hz, 1H), 6.74 (dd, *J* = 8.0, 2.0 Hz, 1H), 6.66 (d, *J* = 9.6 Hz, 2H), 4.21 (dd, *J* = 6.0, 2.8 Hz, 1H), 4.01 (d, *J* = 2.8 Hz, 2H), 3.95 (dd, *J* = 18.0, 6.0 Hz, 1H), 3.68 (s, 3H), 3.48 (s, 3H), 3.25 (d, *J* = 18.0 Hz, 1H), 2.99 (dd, *J* = 18.0, 2.8 Hz, 1H), 2.67 (d, *J* = 18.0 Hz, 1H), 2.31 (s, 3H), 2.20 (s, 3H), 2.11 (s, 3H), 1.91 (d, *J* = 2.8 Hz, 4H), 1.40 (d, *J* = 3.6 Hz, 6H); $^{13}C$ NMR (100 MHz, $CDCl_3$) δ 198.64, 176.54, 174.90, 157.10, 149.35, 147.13, 137.83, 136.64, 136.46, 133.53, 131.09, 130.50, 129.73, 128.84, 128.42, 124.07, 123.80, 121.28, 121.26, 120.92, 119.46, 119.27, 119.25, 115.12, 112.17, 111.94, 109.29, 68.08, 61.77, 53.81, 52.47, 42.61, 41.06, 39.91, 37.35, 36.92, 29.49, 25.44 (d, *J* = 8.4 Hz), 25.28, 25.08, 21.56, 15.98; HRMS (ESI) *m/z* calcd for $C_{45}H_{48}N_2O_6[M+H]^+$ = 713.3585, found = 713.3583; The ee value was >99%, $t_R$ (major) = 13.7 min, $t_R$ (minor) = 17.1 min (Chiralcel IA, λ = 254 nm, 30% i-PrOH/hexane, flow rate = 1.0 mL/min).

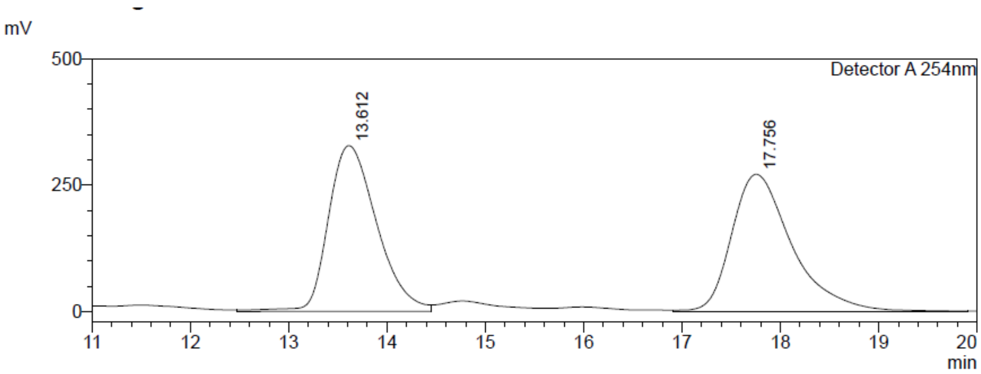


**<Peak Table>**

Detector A 254nm

| Peak# | Ret. Time | Height | Height% | Conc. | Area | Area% |
|---|---|---|---|---|---|---|
| 1 | 13.612 | 327461 | 54.751 | 49.364 | 11240922 | 49.364 |
| 2 | 17.756 | 270635 | 45.249 | 50.636 | 11530631 | 50.636 |
| Total | | 598097 | 100.000 | | 22771554 | 100.000 |

Racemic **6b**

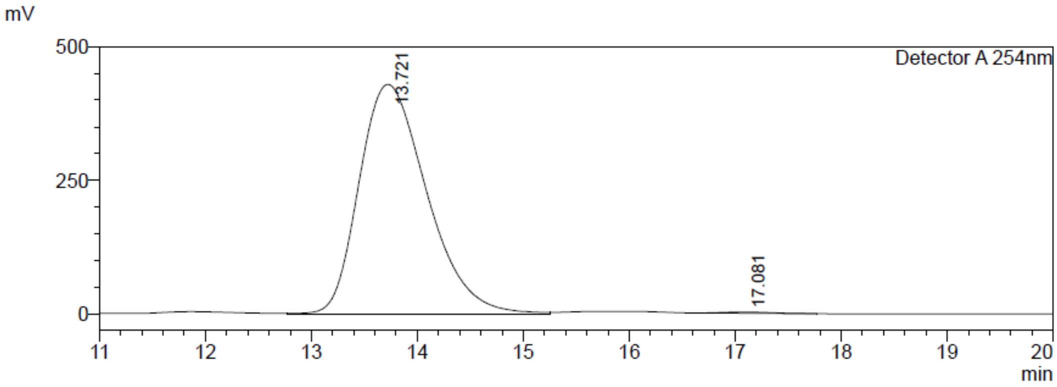


**<Peak Table>**

Detector A 254nm

| Peak# | Ret. Time | Height | Height% | Conc. | Area | Area% |
|---|---|---|---|---|---|---|
| 1 | 13.721 | 429010 | 99.528 | 99.651 | 19088681 | 99.651 |
| 2 | 17.081 | 2033 | 0.472 | 0.349 | 66759 | 0.349 |
| Total | | 431044 | 100.000 | | 19155440 | 100.000 |

Enantiomerically enriched **6b**

**Methyl(*6S,7S,13R*)-2-(((R)-4-((*3R,5R,8R,9S,10S,13R,14S,,17R*)-3-hydroxy-10,13-dimethylhexadecahydro-1H-cyclopenta[a]phenanthren-17-yl)pentanoyl)oxy)-8,13-dimethyl-7-(2-oxo-2-phenylethyl)-5,7,8,13-tetrahydro-6H-6,13-epiminobenzo[4,5]cycloocta[1,2-b]indole-6-carboxylate(6c)**

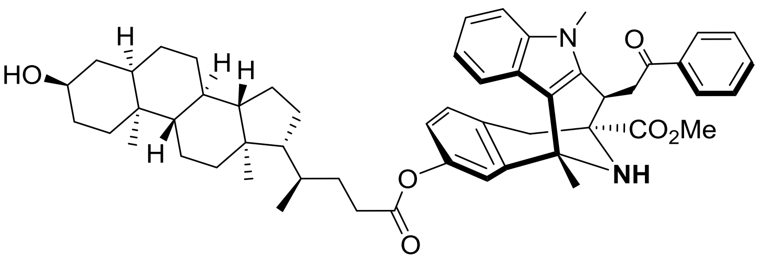

**6c**

A white solid; 70.4 mg, 84% yield; m.p. = 107.2-109.1$^{o}$C, $[\alpha]^{25}_{D}$ = -30.79 (*c* 0.25 $CHCl_3$); *dr* >20:1; $^{1}H$ NMR (400 MHz, $CDCl_3$) δ 7.99 (d, *J* = 7.2 Hz, 2H), 7.59 (d, *J* = 8.4 Hz, 1H), 7.55 (d, *J* = 7.2 Hz, 1H), 7.45 (t, *J* = 7.6 Hz, 1H), 7.39 (d, *J* = 1.2 Hz, 1H), 7.22 (d, *J* = 8.0 Hz, 1H), 7.13 (t, *J* = 7.6 Hz, 1H), 7.06 (t, *J* = 7.6 Hz, 1H), 6.93 (d, *J* = 7.4 Hz, 1H), 6.79 (dd, *J* = 8.0, 2.4 Hz, 1H), 4.21 (dd, *J* = 6.0, 2.8 Hz, 1H), 3.95 (dd, *J* = 18.0, 6.0 Hz, 1H), 3.68 (s, 3H), 3.67 – 3.57 (m, 1H), 3.48 ((q, *J* = 7.2, 1H), 3.49 (s, 3H), 3.47 (s, 1H), 3.25 (d, *J* = 18.0 Hz, 1H), 2.99 (dd, *J* = 18.0, 2.8 Hz, 1H), 2.67 (d, *J* = 18.0 Hz, 1H ), 2.54– 2.46 (m, 1H), 2.13 (s, 3H), 2.13 (s, 2H), 2.02 – 1.92 (m, 3H), 1.82 – 1.73 (m, 3H), 1.69 – 1.59 (m, 3H), 1.54 – 1.48 (m, 3H), 1.45 – 1.36 (m, 6H), 1.35 – 1.24 (m, 6H), 1.23 – 1.18 (m, 3H), 1.01 (d, *J* = 6.0 Hz, 3H), 0.93 (s, 3H), 0.89 (t, *J* = 6.8 Hz, 2H), 0.69 (d, *J* = 1.6 Hz, 3H); $^{13}C$ NMR (100 MHz, $CDCl_3$) δ 198.63, 174.89, 173.00, 149.05, 147.10, 137.82, 136.80, 136.42, 133.53, 131.15, 129.74, 128.83, 128.41, 124.05, 121.26, 119.45, 119.31, 115.26, 111.94, 109.31, 72.01, 65.99, 61.74, 56.69, 56.13, 53.79, 52.46, 42.95, 42.24, 40.59, 40.35, 36.88, 36.61, 36.01, 35.55, 34.73, 31.14, 30.70, 29.47, 28.43, 27.34, 26.57, 25.04, 24.38, 23.52, 22.79, 20.99, 18.52, 15.41, 14.26, 12.23; HRMS (ESI) *m/z* calcd for $C_{54}H_{66}N_2O_6[M+H]^+$ = 839.4994, found = 839.4995; The ee value was 93%, $t_R$ (major) = 11.5 min, $t_R$ (minor) = 13.8 min (Chiralcel IA, λ = 254 nm, 30% i-PrOH/hexane, flow rate = 1.0 mL/min).

**<Chromatogram>**

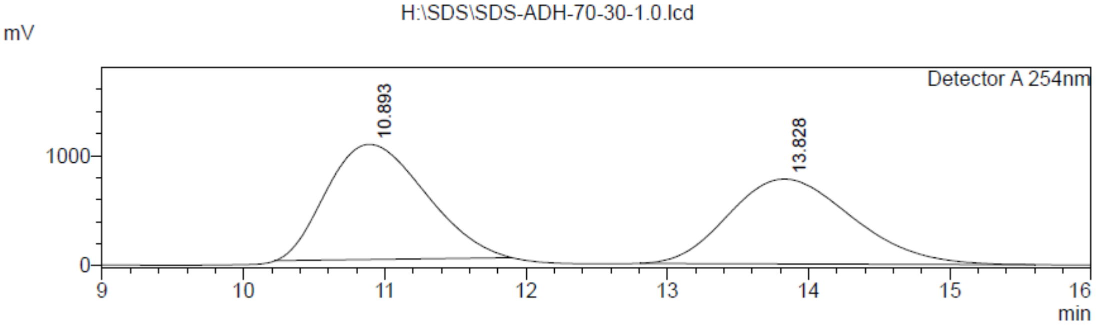


**<Peak Table>**

Detector A 254nm

| Peak# | Ret. Time | Height | Height% | Conc. | Area | Area% |
|---|---|---|---|---|---|---|
| 1 | 10.893 | 1045186 | 57.585 | 51.944 | 50881921 | 51.944 |
| 2 | 13.828 | 769855 | 42.415 | 48.056 | 47072907 | 48.056 |
| Total | | 1815041 | 100.000 | | 97954828 | 100.000 |

Racemic **6c**

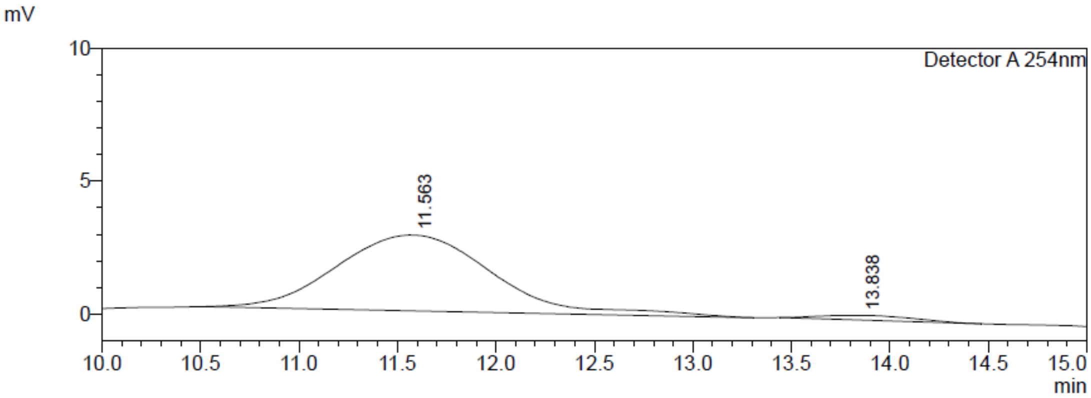


**<Peak Table>**

Detector A 254nm

| Peak# | Ret. Time | Height | Height% | Conc. | Area | Area% |
|---|---|---|---|---|---|---|
| 1 | 11.563 | 2840 | 94.166 | 96.380 | 156449 | 96.380 |
| 2 | 13.838 | 176 | 5.834 | 3.620 | 5877 | 3.620 |
| Total | | 3016 | 100.000 | | 162326 | 100.000 |

Enantiomerically enriched **6c**

**Methyl(6S,7S,13R)-2-(((4R)-4-((10S,13R,17R)-10,13-dimethyl-3,7,12-trioxohexadecahydro-1H-cyclopenta[a]phenanthren-17-yl)pentanoyl)oxy)-8,13-dimethyl-7-(2-oxo-2-phenylethyl)-5,7,8,13-tetrahydro-6H-6,13-epiminobenzo[4,5]cycloocta[1,2-b]indole-6-carboxylate(6d)**

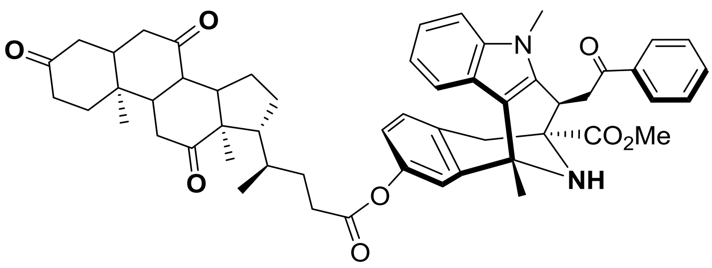

**6d**

A white solid; 70.8 mg, 82% yield; m.p. = 123.6-125.4 $^{o}$C, $[\alpha]^{25}_{D}$ = -25.81 (*c* 0.30 $CHCl_3$); *dr* >20:1; $^{1}$H NMR (400 MHz, $CDCl_3$) δ 7.99 (d, *J* = 7.2 Hz, 2H), 7.57 (dd, *J* = 15.6, 8.0 Hz, 2H), 7.45 (t, *J* = 8.0 Hz, 2H), 7.38 (d, *J* = 2.4 Hz, 1H), 7.22 (d, *J* = 7.6 Hz, 1H), 7.13 (t, *J* = 7.6 Hz, 1H), 6.93 (d, *J* = 8.4 Hz, 1H), 6.78 (dd, *J* = 8.0, 2.0 Hz, 1H), 4.21 (dd, *J* = 6.0, 3.2 Hz, 1H), 3.94 (dd, *J* = 18.0, 6.0 Hz, 1H), 3.68 (s, 3H), 3.48 (s, 3H), 3.25 (d, *J* = 17.6 Hz, 1H), 2.99 (dd, *J* = 18.0, 3.2 Hz, 1H), 2.95 – 2.84 (m, 1H), 2.66 (d, *J* = 17.6 Hz, 1H), 2.41 – 2.30 (m, 5H), 2.26 (t, *J* = 5.4 Hz, 2H), 2.17 (t, *J* = 6.0 Hz, 2H), 2.12 (s, 3H), 2.06 – 1.85 (m, 6H), 1.43 – 1.49 (m, 2H), 1.41 (s, 3H), 1.26 (s, 4H), 1.11 (s, 3H), 0.95 (d, *J* = 6.4 Hz, 3H); $^{13}$C NMR (100 MHz, $CDCl_3$) δ 212.06, 209.17, 208.80, 198.60, 174.88, 172.77, 148.99, 147.12, 137.81, 136.78, 136.43, 133.66, 133.53, 131.20, 129.74, 128.83, 128.40, 124.03, 121.26, 119.43, 119.24, 115.24, 111.91, 109.31, 61.73, 57.07, 53.78, 52.46, 51.90, 49.14, 46.98, 45.76 (d, *J* = 13.8 Hz), 45.11, 42.93, 41.04, 39.87, 38.79, 36.63, 36.15, 35.65 (d, *J* = 3.3 Hz), 35.42, 31.81, 30.56 (d, *J* = 3.1 Hz), 29.47, 27.80 (d, *J* = 5.3 Hz), 25.29, 25.03, 22.04, 18.88, 14.33, 12.02; HRMS (ESI) *m/z* calcd for $C_{54}H_{60}N_2O_8[M+H]^+$ = 865.4422, found =865.4406; The ee value was 88%, $t_R$ (major) = 36.1 min, $t_R$ (minor) = 31.4 min (Chiralcel ADH, λ = 254 nm, 40% i-PrOH/hexane, flow rate = 1.0 mL/min).

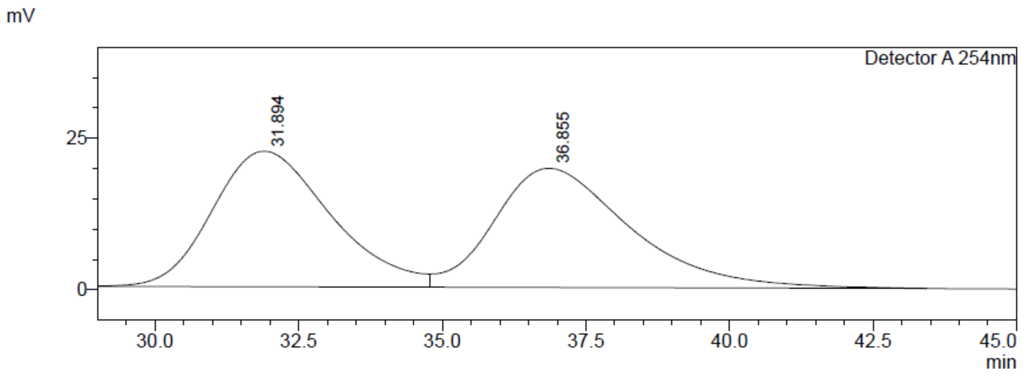


**<Peak Table>**

Detector A 254nm

| Peak# | Ret. Time | Height | Height% | Conc. | Area | Area% |
|---|---|---|---|---|---|---|
| 1 | 31.894 | 22358 | 53.221 | 49.797 | 3210008 | 49.797 |
| 2 | 36.855 | 19652 | 46.779 | 50.203 | 3236118 | 50.203 |
| Total | | 42010 | 100.000 | | 6446127 | 100.000 |

Racemic **6d**

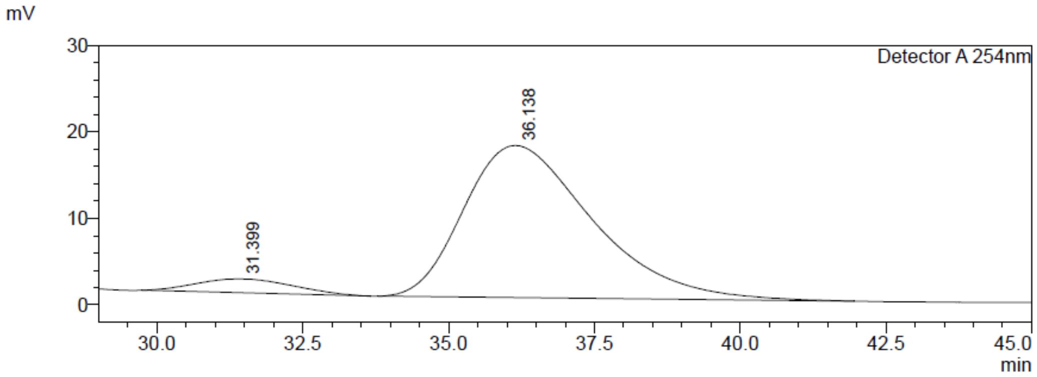


**<Peak Table>**

Detector A 254nm

| Peak# | Ret. Time | Height | Height% | Conc. | Area | Area% |
|---|---|---|---|---|---|---|
| 1 | 31.399 | 1600 | 8.343 | 6.393 | 184338 | 6.393 |
| 2 | 36.138 | 17576 | 91.657 | 93.607 | 2699051 | 93.607 |
| Total | | 19176 | 100.000 | | 2883389 | 100.000 |

Enantiomerically enriched **6d**

# 8. Gram-scale preparations and synthetic applications

## A). Gram-scale preparations

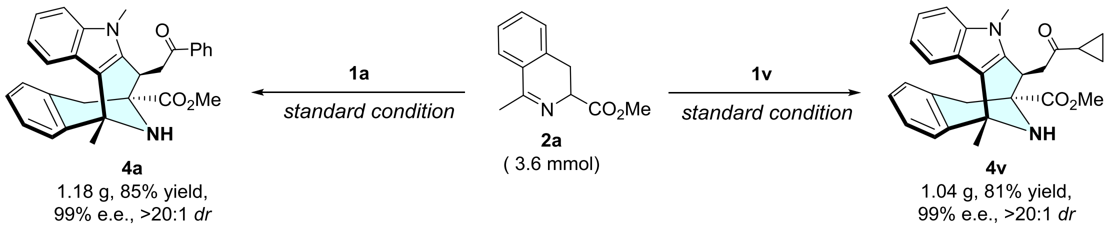


**General procedures:** To a dried round bottle flask with a magnetic stirring bar were

added methyl 1-methyl-3,4-dihydroisoquinoline-3-carboxylate **2a** (0.73 g, 3.6 mmol) and 2-vinyl indolyl ketone **1a** (0.78 g, 3 mmol), followed by the addition of $Cs_2CO_3$ (5.85 g, 18 mmol ) and catalyst **P10** (0.42 g, 10 mol%), followed by the addition of $Et_2O$ (20 mL). The reaction mixture was stirred at -20 $^oC$ for 3.5 days, and TLC show that the reaction was completed. Then, the $BF_3{\cdot}Et_2O$ (30 mmol) was added to the mixture, The reaction mixture was stirred at rt for 12 h to afford **4a** (1.18 g, 85% yield, 99% ee,>20:1 *dr*) as a white solid. The product **4v** was obtained in 1.04 g, 81% yield, 99% ee, >20:1 *dr*, with following the same procedures.

## B). Synthetic applications

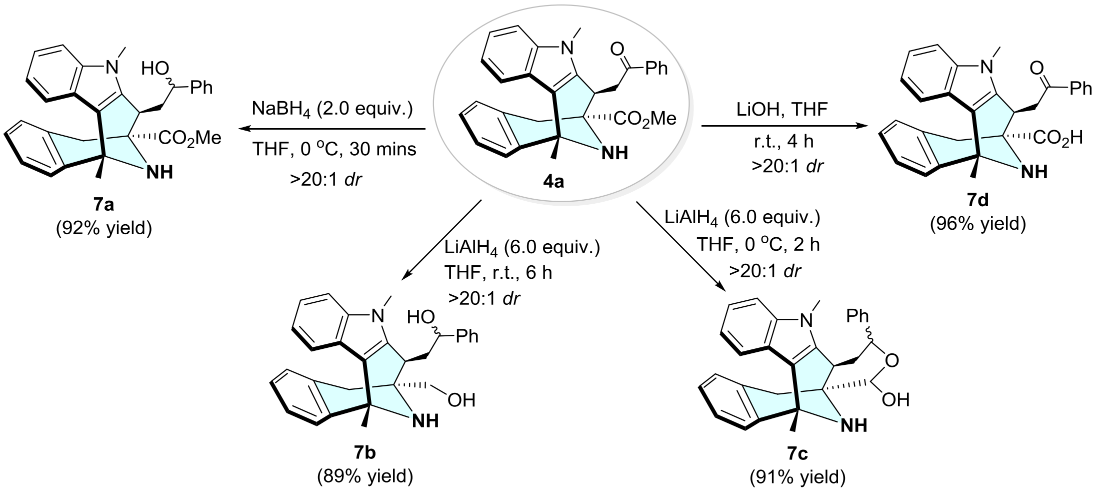


## A. Characterization of the products

**<u>Methyl(6S,7S,13R)-8,13-dimethyl-7-(2-oxo-2-phenylethyl)-5,7,8,13-tetrahydro-6H-6,13-epiminobenzo[4,5]cycloocta[1,2-b]indole-6-carboxylate(7a)</u>**

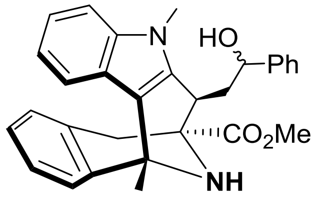

**7a**

To a dried round bottle flask with a magnetic stirring bar were added product **4a** (46.4 mg, 0.1 mmol) and $NaBH_4$ (0.2 mmol) in 5 mL THF. The reaction mixture was stirred at 0 $^oC$ for 30 min, and TLC show that the reaction was completed. Then, the resulting mixture was then quenched with $H_2O$ and extracted with $CH_2Cl_2$ three times. The combined organic layers were dried over $Na_2SO_4$. The solvent was removed under reduce pressure and the residue was purified by column chromatography on

silica gel to afford **7a**; A white solid; 42.9 mg, 92% yield; *dr* >20:1;$^1$H NMR (400 MHz, DMSO) δ 7.91 (s, 1H), 7.74 (d, *J* = 7.6 Hz, 1H), 7.65 (d, *J* = 7.6 Hz, 1H), 7.38 (d, *J* = 8.0 Hz, 1H), 7.28 – 7.25 (m, 4H), 7.20 – 7.12 (m, 2H), 7.05 (q, *J* = 7.2 Hz, 2H), 6.96 (dd, *J* = 16.2, 8.0 Hz, 2H), 5.08 (t, *J* = 4.8 Hz, 1H), 4.11 (d, *J* = 9.2 Hz, 1H), 3.82 (dd, *J* = 10.0, 5.2 Hz, 1H), 3.72 (s, 3H), 3.72 (d, *J* = 14.4 Hz, 1H), 3.33 (s, 3H), 3.29 (d, *J* = 10.4 Hz, 1H), 2.99 (s, 1H), 2.21 (d, *J* = 17.6 Hz, 1H), 2.02 (s, 3H); $^{13}$C NMR (100 MHz, DMSO) δ 167.54, 146.53, 145.54, 137.36, 135.89, 135.39, 128.53, 127.88, 126.35, 125.68, 125.45, 125.02, 123.74, 121.66, 120.31, 118.66, 118.59, 113.16, 109.42, 69.22, 65.81, 55.20, 53.16, 42.11, 40.55, 36.74 , 29.27, 24.08; HRMS (ESI) *m/z* calcd for $C_{30}H_{30}N_2O_3[M+H]^+$ = 467.2329, found = 467.2327.

**2-((6S,7S,13R)-6-(hydroxymethyl)-8,13-dimethyl-6,7,8,13-tetrahydro-5H-6,13-epiminobenzo[4,5]cycloocta[1,2-b]indol-7-yl)-1-phenylethan-1-ol(7b)**

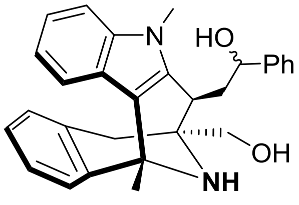


**7b**

To a dried round bottle flask with a magnetic stirring bar were added product **4a** (46.4 mg, 0.1 mmol) in 5 mL THF, $LiAlH_4$(0.6 mmol) was added solwly to the solution at 0$^o$C.The reaction mixture was stirred at this temperature for 6 h, and TLC show that the reaction was completed. Then, the resulting mixture was then quenched with $H_2O$ and extracted with $CH_2Cl_2$ three times. The combined organic layers were dried over $Na_2SO_4$. The solvent was removed under reduce pressure and the residue was purified by column chromatography on silica gel to afford **7b**; A white solid; 38.9 mg, 89% yield; *dr* >20:1;$^1$H NMR (400 MHz, $CDCl_3$) δ 7.60 (dd, *J* = 18.0, 7.2 Hz, 2H), 7.26 (s,1H), 7.20 (t, *J* = 7.6 Hz, 2H), 7.15 (d, *J* = 6.4 Hz, 1H), 7.12 (d, *J* = 7.6 Hz, 2H), 7.09 – 7.05 (m, 3H), 7.03 – 6.98 (m, 2H), 5.06 (dd, *J* = 7.6, 4.4 Hz, 1H), 3.97 (d, *J* = 10.8 Hz, 1H), 3.73 (d, *J* = 10.8 Hz, 1H), 3.43 (s, 3H), 3.31 (d, *J* = 17.2 Hz, 1H), 3.10 (dd, *J* = 5.2, 3.6 Hz, 1H), 2.52 (ddd, *J* = 15.6, 7.6, 5.6 Hz, 1H), 2.30 (d, *J* = 17.2 Hz, 1H), 2.22 (ddd, *J* = 15.2, 7.2, 3.2 Hz, 1H), 2.11 (s, 3H), 1.27 (s, 1H); $^{13}$C NMR (100 MHz, $CDCl_3$) δ 145.28, 144.71, 137.63, 137.31, 135.32, 129.02, 128.18, 127.20, 126.42, 125.80, 125.44, 124.37, 121.56, 120.93, 119.22, 119.11, 111.99, 109.05, 73.22,

67.41, 56.85, 54.28, 40.76, 39.57, 37.49, 29.56, 24.62; HRMS (ESI) *m/z* calcd for $C_{29}H_{30}N_2O_2[M+H]^+$ = 439.2380, found =439.2379.

**(4R,4aS,10R,15bS)-10,15-dimethyl-2-phenyl-1,2,5,10,15,15b-hexahydro-4H-4a,10-epiminobenzo[4,5]pyrano[3',4':7,8]cycloocta[1,2-b]indol-4-ol (7c)**

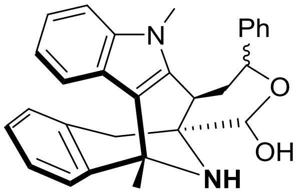


**7c**

To a dried round bottle flask with a magnetic stirring bar were added product **4a** (46.7 mg, 0.1 mmol) in 5 mL THF, $LiAlH_4$ (0.6 mmol) was added solwly to the solution at 0 °C. The reaction mixture was stirred at this temperature for 2 h.Then, the resulting mixture was then quenched with $H_2O$ and extracted with $CH_2Cl_2$ three times. The combined organic layers were dried over $Na_2SO_4$. The solvent was removed under reduce pressure and the residue was purified by column chromatography on silica gel to afford **7c**; A white solid; 39.7 mg, 91% yield; *dr* >20:1; $^1$H NMR (400 MHz, $CDCl_3$) δ 7.64 (dd, *J* = 17.8, 7.4 Hz, 2H), 7.45 (d, *J* = 7.2, 2H), 7.36 (t, *J* = 7.2 Hz, 2H), 7.30 (dt, *J* = 6.4, 1.6 Hz, 1H), 7.23 (d, *J* = 8.4 Hz, 1H), 7.19 – 7.12 (m, 2H), 7.06 (td, *J* = 7.6, 0.8 Hz, 2H), 7.01 (d, *J* = 8.0 Hz, 1H), 4.98 (s, 1H), 4.82 (dd, *J* = 11.6, 1.6 Hz, 1H), 3.66 (s, 3H), 3.32 (d, *J* = 17.4 Hz, 1H), 3.02 (dd, *J* = 12.0, 5.2 Hz, 1H), 2.51 (d, *J* = 17.2 Hz, 1H), 2.33 (ddd, *J* = 14.4, 4.8, 2.0 Hz, 1H), 2.11 (s, 3H), 1.91 (q, *J* = 12.0 Hz, 1H), 1.27 (s, 1H); $^{13}$C NMR (100 MHz, $CDCl_3$) δ 144.16, 140.62, 137.62, 135.72, 134.25, 128.63, 128.46, 128.10, 126.30, 126.17, 125.65, 124.37, 121.40, 121.16, 119.71, 119.47, 115.18, 109.09, 100.08, 75.90, 53.64, 53.31, 40.80, 36.77, 36.61, 29.29, 25.18; HRMS (ESI) *m/z* calcd for $C_{29}H_{28}N_2O_2[M+H]^+$ = 437.2224, found = 437.2225.

**(6S,7S,13R)-8,13-dimethyl-7-(2-oxo-2-phenylethyl)-5,7,8,13-tetrahydro-6H-6,13-epiminobenzo[4,5]cycloocta[1,2-b]indole-6-carboxylic acid(7d)**

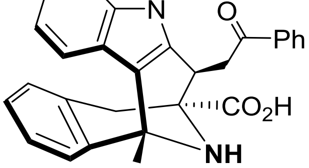


**7d**

To a dried round bottle flask with a magnetic stirring bar were added product **4a** (46.7 mg, 0.1 mmol) and LiOH (0.5 mmol) in 10 mL THF/$H_2O$ (V:V = 1:1). The reaction mixture was stirred at rt for 4 h, and TLC show that the reaction was completed. Then, 1 M HCl (aq) was added and adjusted PH to 3-4. After that, the solvent was removed under reduce pressure and extracted with $CH_2Cl_2$ three times. The combined organic layers were dried over $Na_2SO_4$ and after evaporation of solvent, the residue was purified by column chromatography on silicagel to afford **7d**. A white solid; 43.2 mg, 96% yield; *dr* >20:1; $^1$H NMR (400 MHz, $CDCl_3$) δ 7.89 (d, *J* = 7.0 Hz, 2H), 7.55 (d, *J* = 7.6 Hz, 2H), 7.41 (d, *J* = 6.4 Hz, 1H), 7.29 (d, *J* = 7.2 Hz, 2H), 7.18 – 7.10 (m,, 3H), 7.08 – 7.02 (m, 1H), 6.95 (t, *J* = 7.0 Hz, 1H), 6.83 (d, *J* = 7.0 Hz, 1H), 4.06 (s,2H), 3.61 (d, *J* = 17.8 Hz, 1H), 3.58 (s, 3H), 3.51 (d, *J* = 16.8 Hz, 1H), 2.74 (d, *J* = 17.8 Hz, 1H), 2.41 (s, 3H); $^{13}$C NMR (100 MHz, $CDCl_3$) δ 199.92, 173.55, 139.11, 138.24, 136.43, 133.66, 133.03, 128.87, 128.66, 127.48, 126.40, 123.31, 122.31, 121.90, 119.78, 119.09, 111.15, 109.50, 62.68, 57.64, 40.92 , 39.46, 37.83, 29.96, 22.27; HRMS (ESI) *m/z* calcd for $C_{29}H_{26}N_2O_3[M+H]^+$ = 451.2016, found =451.2015.

## 9. Determination of absolute configuration of products

### A). X-ray structure of 3a

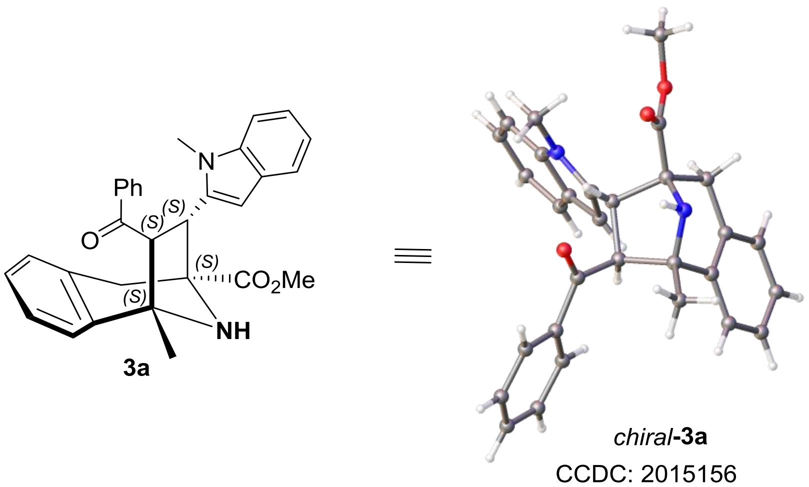


*chiral*-**3a**
CCDC: 2015156

**Supplementary Table S7.** Crystal data and structure refinement for **3a**

| | |
|---|---|
| Identification code | **3a** |
| Empirical formula | $C_{30}H_{28}N_2O_3$ |

| | |
|---|---|
| Formula weight | 464.54 |
| Temperature/K | 140(2) |
| Crystal system | orthorhombic |
| Space group | $P2_12_12_1$ |
| a/Å | 8.3361(3) |
| b/Å | 12.7644(4) |
| c/Å | 22.5526(7) |
| α/° | 90 |
| β/° | 90 |
| γ/° | 90 |
| Volume/Å$^3$ | 2399.72(14) |
| Z | 4 |
| $\rho_{calc}$g/cm$^3$ | 1.286 |
| μ/mm$^{-1}$ | 0.662 |
| F(000) | 984.0 |
| Crystal size/mm$^3$ | 0.188 × 0.163 × 0.105 |
| Radiation | CuKα (λ = 1.54178) |
| 2Θ range for data collection/° | 7.84 to 160.878 |
| Index ranges | $-10 \leq h \leq 10$, $-16 \leq k \leq 16$, $-28 \leq l \leq 26$ |
| Reflections collected | 24674 |
| Independent reflections | 5181 [$R_{int}$ = 0.0449, $R_{sigma}$ = 0.0331] |
| Data/restraints/parameters | 5181/1/323 |
| Goodness-of-fit on $F^2$ | 1.076 |
| Final R indexes [I>=2σ (I)] | $R_1$ = 0.0335, $wR_2$ = 0.0810 |
| Final R indexes [all data] | $R_1$ = 0.0367, $wR_2$ = 0.0847 |
| Largest diff. peak/hole / e Å$^{-3}$ | 0.29/-0.22 |
| Flack parameter | 0.02(8) |

**B).** X-ray structure of **4a**

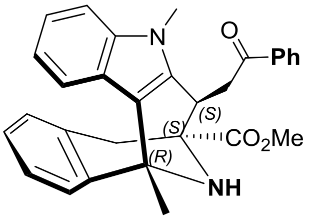


**4a**

≡

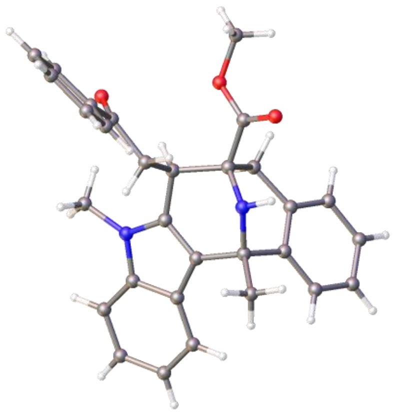

*chiral*-**4a**
CCDC: 2019560

**Supplementary Table S8.** Crystal data and structure refinement for **4a**

| | |
|---|---|
| Identification code | **4a** |
| Empirical formula | $C_{30}H_{28}N_2O_3$ |
| Formula weight | 464.54 |
| Temperature/K | 142.62 |
| Crystal system | orthorhombic |
| Space group | $P2_12_12_1$ |
| a/Å | 8.1575(2) |
| b/Å | 14.4080(4) |
| c/Å | 20.4914(5) |
| α/° | 90 |
| β/° | 90 |
| γ/° | 90 |
| Volume/Å$^3$ | 2408.42(11) |
| Z | 4 |
| $\rho_{calc}$g/cm$^3$ | 1.281 |
| μ/mm$^{-1}$ | 0.660 |
| F(000) | 984.0 |
| Crystal size/mm$^3$ | 0.188 × 0.179 × 0.127 |
| Radiation | CuKα (λ = 1.54178) |
| 2Θ range for data collection/° | 7.5 to 161.15 |

| | |
|---|---|
| Index ranges | $-9 \le h \le 10$, $-16 \le k \le 18$, $-22 \le l \le 25$ |
| Reflections collected | 13542 |
| Independent reflections | 5004 [$R_{int}$ = 0.2143, $R_{sigma}$ = 0.1144] |
| Data/restraints/parameters | 5004/0/323 |
| Goodness-of-fit on $F^2$ | 1.043 |
| Final R indexes [I>=2σ (I)] | $R_1$ = 0.0532, $wR_2$ = 0.1198 |
| Final R indexes [all data] | $R_1$ = 0.0717, $wR_2$ = 0.1299 |
| Largest diff. peak/hole / e Å$^{-3}$ | 0.28/-0.28 |
| Flack parameter | 0.0(2) |

## **C).** X-ray structure of *rac*-**3a**

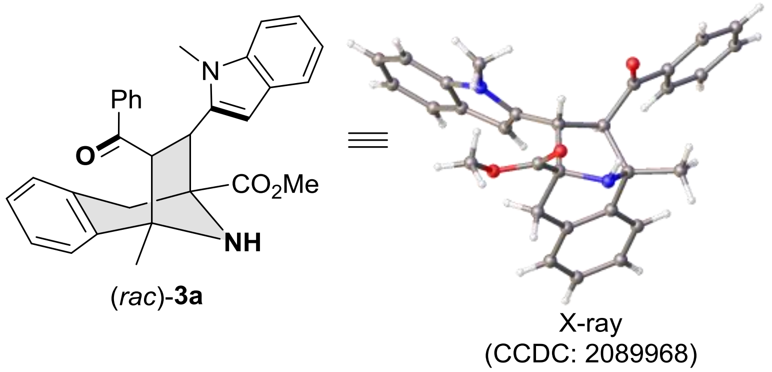


(*rac*)-**3a**

X-ray
(CCDC: 2089968)

**Supplementary Table S9.** Crystal data and structure refinement for *rac*-**3a**

| | |
|---|---|
| Identification code | *rac*-**3a** |
| Empirical formula | $C_{30}H_{28}N_2O_3$ |
| Formula weight | 464.54 |
| Temperature/K | 304.0 |
| Crystal system | monoclinic |
| Space group | $P2_1/n$ |
| a/Å | 12.3986(4) |
| b/Å | 8.1607(3) |
| c/Å | 25.0636(8) |

| | |
|---|---|
| α/° | 90 |
| β/° | 103.092(2) |
| γ/° | 90 |
| Volume/Å$^3$ | 2470.05(15) |
| Z | 4 |
| $\rho_{calc}$g/cm$^3$ | 1.249 |
| μ/mm$^{-1}$ | 0.643 |
| F(000) | 984.0 |
| Crystal size/mm$^3$ | 0.39 × 0.29 × 0.13 |
| Radiation | CuKα (λ = 1.54178) |
| 2Θ range for data collection/° | 7.242 to 144.436 |
| Index ranges | $-15 \leq h \leq 15$, $-10 \leq k \leq 9$, $-30 \leq l \leq 30$ |
| Reflections collected | 32160 |
| Independent reflections | 4857 [$R_{int}$ = 0.0381, $R_{sigma}$ = 0.0231] |
| Data/restraints/parameters | 4857/0/322 |
| Goodness-of-fit on $F^2$ | 1.052 |
| Final R indexes [I>=2σ (I)] | $R_1$ = 0.0422, $wR_2$ = 0.1142 |
| Final R indexes [all data] | $R_1$ = 0.0522, $wR_2$ = 0.1213 |
| Largest diff. peak/hole / e Å$^{-3}$ | 0.13/-0.20 |

## **D).** X-ray structure of *rac*-**4a**

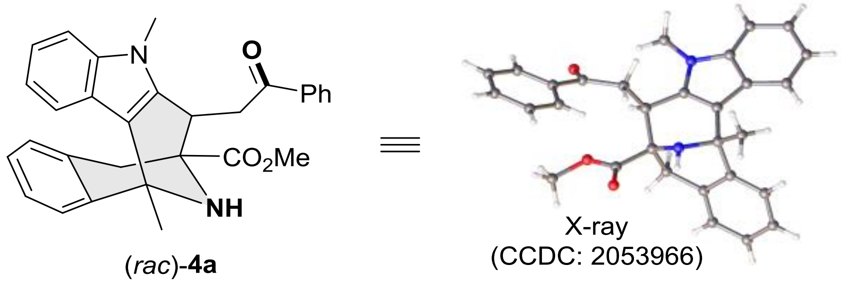


(*rac*)-**4a**

**Supplementary Table S10.** Crystal data and structure refinement for *rac*-**4a**

| | |
|---|---|
| Identification code | *rac*-**4a** |

| | |
|---|---|
| Empirical formula | $C_{30}H_{28}N_2O_3$ |
| Formula weight | 464.54 |
| Temperature/K | 298.91(10) |
| Crystal system | orthorhombic |
| Space group | Pccn |
| a/Å | 32.1838(6) |
| b/Å | 18.6455(4) |
| c/Å | 8.08254(14) |
| α/° | 90 |
| β/° | 90 |
| γ/° | 90 |
| Volume/Å$^3$ | 4850.20(16) |
| Z | 8 |
| $\rho_{calc}$g/cm$^3$ | 1.272 |
| μ/mm$^{-1}$ | 0.655 |
| F(000) | 1968.0 |
| Crystal size/mm$^3$ | 0.7 × 0.4 × 0.15 |
| Radiation | CuKα (λ = 1.54184) |
| 2Θ range for data collection/° | 9.512 to 142.598 |
| Index ranges | $-39 \leq h \leq 38$, $-22 \leq k \leq 14$, $-9 \leq l \leq 6$ |
| Reflections collected | 13491 |
| Independent reflections | 4554 [$R_{int}$ = 0.0547, $R_{sigma}$ = 0.0396] |
| Data/restraints/parameters | 4554/0/322 |
| Goodness-of-fit on F$^2$ | 1.039 |
| Final R indexes [I>=2σ (I)] | $R_1$ = 0.0614, $wR_2$ = 0.1600 |
| Final R indexes [all data] | $R_1$ = 0.0723, $wR_2$ = 0.1759 |
| Largest diff. peak/hole / e Å$^{-3}$ | 0.23/-0.30 |

## **E).** X-ray structure of *ent*-**4a**

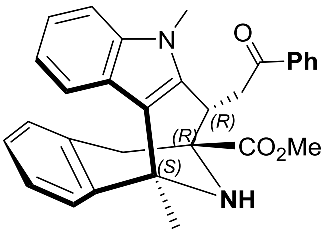


***ent*-4a**

≡

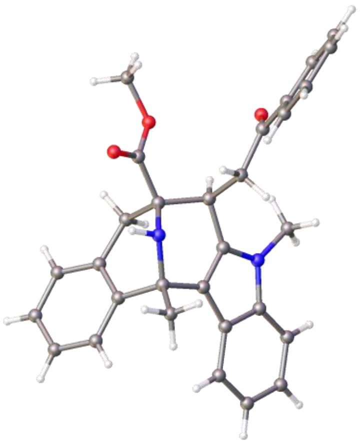

*chiral*- ***ent*-4a**
CCDC: 2047395

**Supplementary Table S11.** Crystal data and structure refinement for *ent*-**4a**

| | |
|---|---|
| Identification code | *ent*-**4a** |
| Empirical formula | $C_{30}H_{28}N_2O_3$ |
| Formula weight | 464.54 |
| Temperature/K | 299.0 |
| Crystal system | orthorhombic |
| Space group | $P2_12_12_1$ |
| a/Å | 8.2733(2) |
| b/Å | 14.4045(4) |
| c/Å | 20.7550(5) |
| α/° | 90 |
| β/° | 90 |
| γ/° | 90 |
| Volume/Å$^3$ | 2473.43(11) |
| Z | 4 |
| $\rho_{calc}$g/cm$^3$ | 1.247 |
| μ/mm$^{-1}$ | 0.643 |
| F(000) | 984.0 |
| Crystal size/mm$^3$ | 0.5 × 0.3 × 0.2 |
| Radiation | CuKα (λ = 1.54178) |
| 2Θ range for data collection/° | 7.47 to 144.536 |

| | |
|---|---|
| Index ranges | $-7 \le h \le 10$, $-17 \le k \le 17$, $-25 \le l \le 25$ |
| Reflections collected | 30379 |
| Independent reflections | 4863 [$R_{int}$ = 0.0645, $R_{sigma}$ = 0.0350] |
| Data/restraints/parameters | 4863/0/322 |
| Goodness-of-fit on $F^2$ | 1.094 |
| Final R indexes [I>=2σ (I)] | $R_1$ = 0.0417, $wR_2$ = 0.1032 |
| Final R indexes [all data] | $R_1$ = 0.0470, $wR_2$ = 0.1081 |
| Largest diff. peak/hole / e Å$^{-3}$ | 0.17/-0.18 |
| Flack parameter | 0.04(10) |

## **F).** X-ray structure of **3a-1**

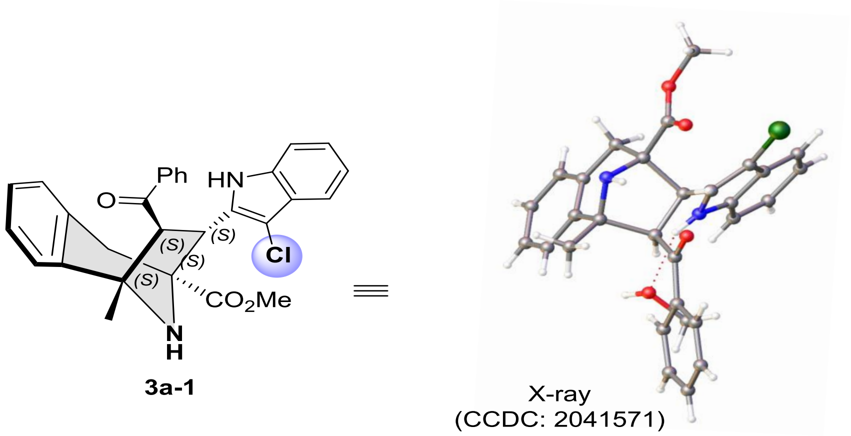


**Supplementary Table S12.** Crystal data and structure refinement for **3a-1**

| | |
|---|---|
| Identification code | **3a-1** |
| Empirical formula | $C_{30}H_{29}ClN_2O_4$ |
| Formula weight | 517.00 |
| Temperature/K | 294.3(4) |
| Crystal system | triclinic |
| Space group | P1 |
| a/Å | 8.0236(6) |
| b/Å | 8.8217(7) |
| c/Å | 10.5470(9) |
| α/° | 76.380(7) |

| | |
|---|---|
| β/° | 72.627(7) |
| γ/° | 67.059(7) |
| Volume/$Å^3$ | 650.08(10) |
| Z | 1 |
| $\rho_{calc}g/cm^3$ | 1.321 |
| $\mu/mm^{-1}$ | 1.618 |
| F(000) | 272.0 |
| Crystal size/$mm^3$ | 0.5 × 0.4 × 0.3 |
| Radiation | CuKα (λ = 1.54184) |
| 2Θ range for data collection/° | 8.866 to 142.848 |
| Index ranges | -6 ≤ h ≤ 9, -10 ≤ k ≤ 10, -12 ≤ l ≤ 12 |
| Reflections collected | 6517 |
| Independent reflections | 3514 [$R_{int}$ = 0.0322, $R_{sigma}$ = 0.0375] |
| Data/restraints/parameters | 3514/3/342 |
| Goodness-of-fit on $F^2$ | 1.030 |
| Final R indexes [I>=2σ (I)] | $R_1$ = 0.0470, $wR_2$ = 0.1229 |
| Final R indexes [all data] | $R_1$ = 0.0478, $wR_2$ = 0.1245 |
| Largest diff. peak/hole / e $Å^{-3}$ | 0.18/-0.26 |
| Flack parameter | -0.001(18) |

## 10. Bioactive investigations

The cytotoxic effects of these newly synthesized compounds were evaluated by Cell Counting Kit-8 (CCK-8, Dojindo, Japan) assay. Cell lines include HCT116, A375, HELA, NCIN87, and HUVEC were obtained from the Cell Bank of the Chinese Academy of Sciences (Shanghai, China), and BB4 cell line (gefitinib-resistant non-small cell lung cancer PC-9 cells) was a gift from professor Qinghua Zhou’s laboratory at West China Hospital, Sichuan University. Firstly, we screened the anti-cancer activity of these compounds on three different cancer cells at the concentration of 20 μM, including HCT116 human colon cancer cells, BB4 human lung cancer Cells, and A375 human melanoma cells. According to the results, compounds with strong activity were screened out, and then the half maximal

inhibitory concentration ($IC_{50}$) value of these compounds was determined in a panel of cancer cells and normal cells. These cells include HCT116 human colon cancer cells, BB4 human lung cancer cells, A375 human melanoma cells, HELA human cervical cancer cells, NCIN87 human gastric cancer cells, and HUVEC normal human umbilical vein endothelial cells. In addition, two classic anti-cancer chemotherapeutic drugs (Cisplatin and Irinotecan) were used as positive controls, respectively.

Briefly, the cells at the logarithmic growth phase were inoculated into the 96-well culture plate at an appropriate density and were adhered to the bottom overnight. Then, the cells were treated with solvent control or tested compounds at the indicated concentrations. After 72 hours of incubation, the culture medium was removed, and 100 μL of fresh medium containing 10% volume of CCK-8 reagent was added to each well. After incubation at 37 ℃ for 2 hours, the absorbance values at 450 nm and 600 nm were read using a microplate reader (Bio Tek Instrument, EPOCH), and the OD values at 600 nm were subtracted in the calculation as the reference. The cell viability (%) was calculated as: (ODcmpd. - ODblank)/(ODcontrol - ODblank) × 100%. According to the cell viability data, the half maximal inhibitory concentration ($IC_{50}$) value of the compounds was calculated by dose-response-inhibition method in Graphpad prism 8.4.3 software.

The preliminary screening results demonstrated that the compounds **4o** and **4s** showed high cytotoxicity in different cancer cells (Supplementary Figure S4 and Supplementary Table S13). In addition, the representative compounds exhibited selective cytotoxicity to cancer cells. Their $IC_{50}$ values ranged from 2.24 μM to 109.9 μM in these cancer cells, with the highest activity in HCT116 cancer cells (3.29 μM and 2.29 μM), which is similar to the positive control drugs (4.12 μM and 1.61 μM). However, the cytoxicity of these compounds in normal HUVEC cells was about ten-fold lower than that in cancer cells, indicating they have certain cancer cell specificity (Supplementary Table S14).

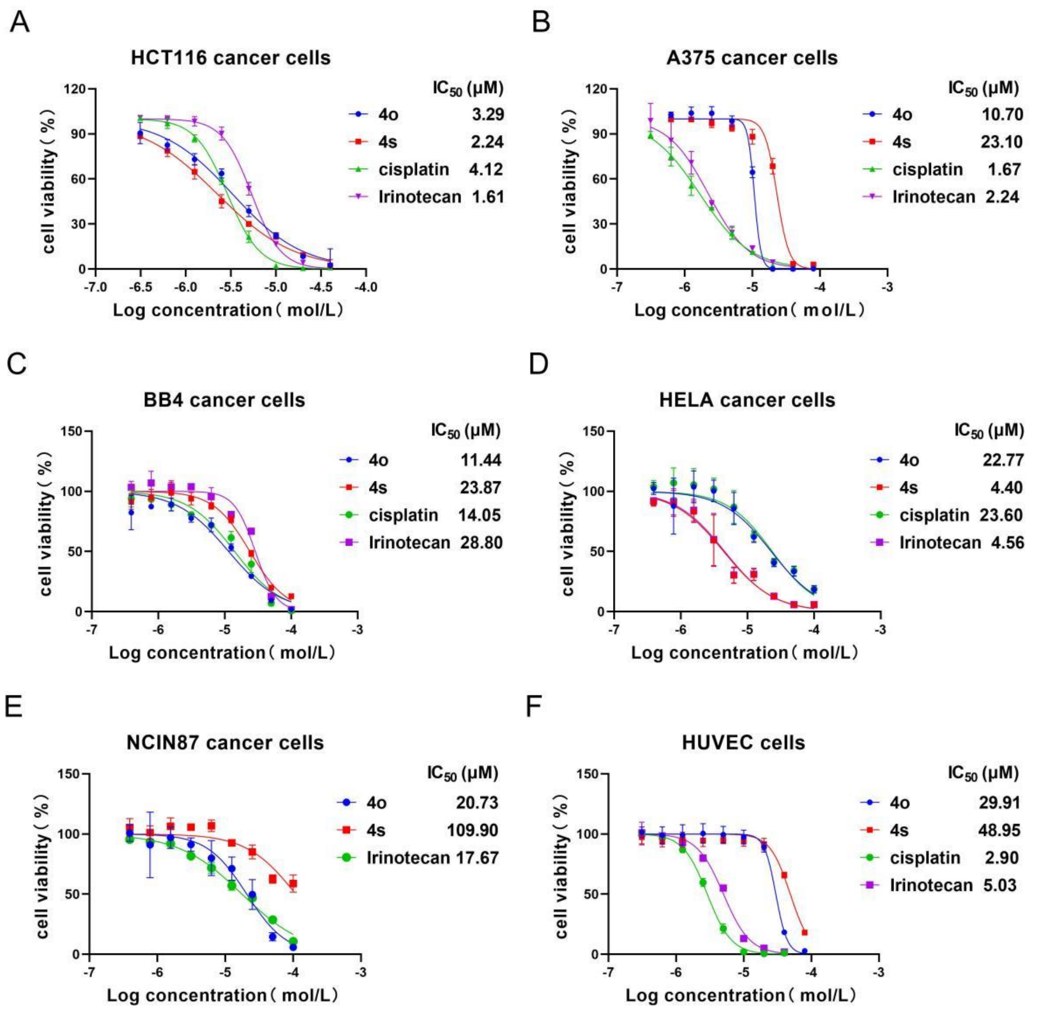


**Supplementary Figure S4.** The cytotoxicity of representative products against different cancer cells and normal cells (mean ± sd., n= 3 biologically independent samples)

**Supplementary Table S13.** The cytotoxicity screen of these newly synthesized compounds against different cancer cells

| No. | Cmpd ID | Cell viability at 20 μM (%) | | | | | |
|---|---|---|---|---|---|---|---|
| | | HCT116 | SD(%) | BB4 | SD(%) | A375 | SD(%) |
| 1 | 4a | 62.1 | 6.6 | 58.4 | 7.5 | 93.8 | 10.9 |
| 2 | 4j | 80.7 | 4.7 | 92.5 | 15.0 | 77.3 | 5.6 |
| 3 | 4h | 70.7 | 4.3 | 53.2 | 6.3 | 71.7 | 0.3 |
| 4 | 4g | 73.1 | 0.5 | 42.7 | 3.7 | 75.6 | 5.0 |
| 5 | 4b | 73.2 | 2.3 | 75.8 | 7.2 | 56.1 | 10.1 |
| 6 | 4c | 76.7 | 11.6 | 86.9 | 13.0 | 76.6 | 8.5 |
| 7 | 4d | 73.3 | 2.0 | 88.3 | 9.2 | 71.7 | 11.5 |

| | | | | | | | |
|---|---|---|---|---|---|---|---|
| 8 | 4e | 65.3 | 2.1 | 76.4 | 13.6 | 70.5 | 20.0 |
| 9 | 4k | 87 | 16.2 | 81.5 | 3.3 | 97.9 | 14.3 |
| 10 | 4l | 76.8 | 10.1 | 49.8 | 2.8 | 77.9 | 2.5 |
| 11 | 4m | 93.5 | 5.3 | 94.4 | 7.6 | 43.7 | 1.7 |
| 12 | 4n | 86.4 | 3.0 | 96.3 | 2.4 | 75.9 | 1.1 |
| 13 | 4q | 91.5 | 1.4 | 81.2 | 16.4 | 75.1 | 7.8 |
| 14 | 4o | 8.44 | 1.0 | 31.8 | 0.2 | 66.9 | 16.1 |
| 15 | 4r | 99.1 | 7.6 | 68 | 3.0 | 81.3 | 5.7 |
| 16 | 4p | 78.2 | 4.2 | 66 | 4.5 | 71.8 | 2.0 |
| 17 | 4s | 3.9 | 1.5 | 42.1 | 6.2 | 66.9 | 15.9 |
| 18 | 4v | 85.7 | 3.7 | 73.1 | 6.5 | 82.7 | 12.5 |
| 19 | 4w | 78.5 | 4.9 | 82.9 | 1.9 | 80.1 | 8.6 |
| 20 | 4ad | 67.2 | 9.6 | 41.8 | 6.6 | 93.3 | 11.7 |
| 21 | 4ag | 70.5 | 3.7 | 60.6 | 12.2 | 88.2 | 7.9 |
| 22 | 4ah | 56.1 | 9.4 | 51.8 | 8.1 | 78 | 2.8 |
| 23 | 4ai | 56.2 | 1.8 | 80.4 | 7.7 | 63.8 | 8.0 |
| 24 | 4af | 49.5 | 3.4 | 57.8 | 12.5 | 66.8 | 10.5 |
| 25 | 4ab | 83.1 | 0.7 | 103.6 | 7.4 | 83.2 | 10.4 |
| 26 | 5a | 73.3 | 6.8 | 83.4 | 3.5 | 69.5 | 7.8 |
| 27 | 5c | 70.2 | 7.6 | 80 | 11.2 | 63.7 | 7.7 |
| 28 | 5b | 89 | 3.2 | 94.3 | 4.7 | 82.1 | 8.2 |
| 29 | 5e | 56.2 | 0.9 | 61 | 2.5 | 74.5 | 0.1 |
| 30 | 5f | 58.5 | 1.8 | 67.8 | 6.3 | 95.1 | 5.6 |
| 31 | 5g | 63.6 | 6.7 | 79.8 | 11.1 | 91 | 8.6 |
| 32 | 5n | 64.7 | 3.9 | 97.4 | 4.3 | 75.4 | 2.3 |
| 33 | 5p | 71.9 | 8.5 | 87.3 | 8.2 | 85.8 | 1.5 |
| 34 | 5q | 75.6 | 4.7 | 102.7 | 9.1 | 70.6 | 2.2 |
| 35 | 5d | 64.8 | 4.2 | 84.1 | 10.1 | 61.1 | 0.6 |
| Irinotecan | | 4.5 | 0.7 | 49.6 | 5.0 | 4.4 | 0.5 |

The value highlighted in red indicates that the cell viability is less than 50%.

**Supplementary Table S14.** The median $IC_{50}$ values (μM) of selected compounds against cancer cells and normal cells

| No. | Cmpd ID | Cancer cells | | | | | Normal cell |
|---|---|---|---|---|---|---|---|
| | | **HCT116** | **BB4** | **A375** | **HELA** | **NCIN87** | **HUVEC** |
| 14 | **4o** | 3.30 | 11.44 | 10.70 | 22.77 | 20.73 | 29.91 |
| 17 | **4s** | 2.24 | 23.87 | 23.10 | 4.40 | 109.90 | 48.95 |
| Cisplatin | | 4.11 | 14.05 | 1.67 | 23.60 | - | 2.90 |
| Irinotecan | | 1.61 | 28.80 | 2.24 | 4.56 | 17.76 | 5.03 |

# 11. Mechanism studies

## A). Investigation of the reaction pathway

In order to understand the reaction mechanism, we carried out stepwise experiments. Firstly, the model reaction between 2-indole-vinyl ketone **1a** and cyclic azomethine ylide **2a** was performed in the presence of catalyst **P10** under standard PTC conditions, and chiral key intermediate **3a** (CCDC 2015156) was obtained and fully characterized (94% yield, >99% ee and >20:1 *d.r.*) and indeed, this seven-membered product **3a** could be quantitatively transformed into chiral eight-membered product **4a** (CCDC 2019560) via a Lewis acid $BF_3 \cdot Et_2O$ promoted ring-opening/Friedel-Crafts cascade process without any loss of the stereoselectivities. Furthermore, while the phosphonium *ent*-**P10** was utilized as catalyst, the model reaction proceeded efficiently to form the corresponding product *ent*-**4a** (CCDC 2047395) with excellent enantioselectivity under the standard "one-pot" conditions. These preliminary results clearly demonstrated that the desired [3.3.1] cyclic product **4a** was generated from the key [3.2.1] cyclic intermediate **3a**, but not directly constructed via formal [3 + 3] cyclization between **1a** and 1, 3-dipole **2a**.

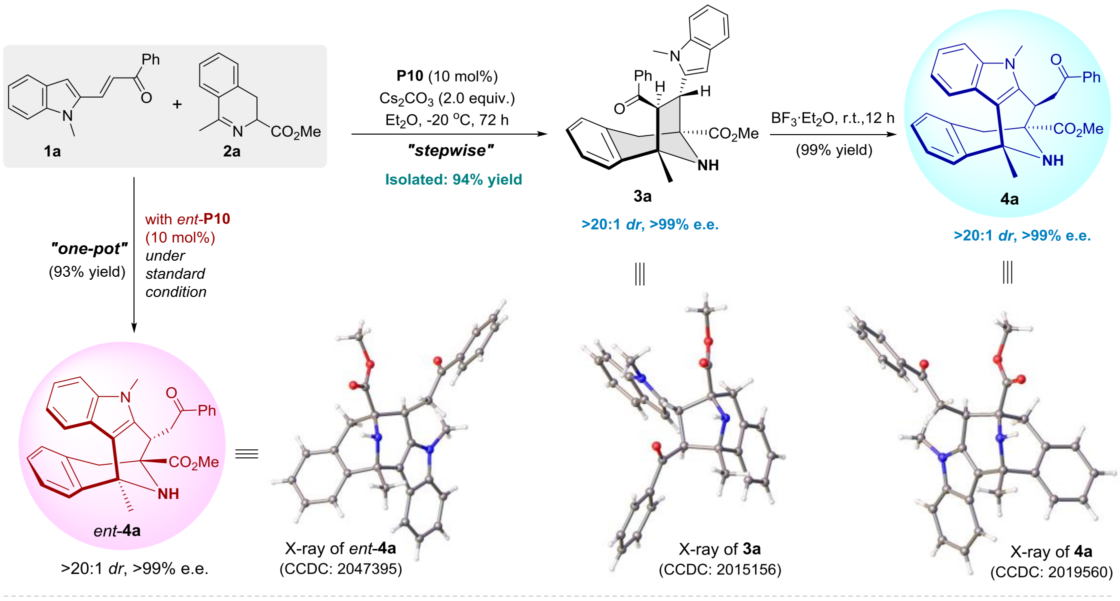


## B). Control experiments

To gain further insight into this reaction mechanism and reaction pathway, various

control experiments were further carried out. In particular, while the NH-unit of **3a** was protected to thus give *N*-acetyl [3.2.1] cyclic intermediate **3a-0**, this compound could not be converted into the ring-expanding [3.3.1] cyclic system under the Lewis acid catalytic conditions, which was probably due to the failure of the acid-promoted ring-opening (*eq. a*). When the C3-position of indole moiety of substrate was substituted by a group such as Cl atom, the corresponding [3 + 2] cyclization product **3a-1** (CCDC 2041571) was generated; however, the above ring-opening/ring expanding process of this seven-membered intermediate could not occur, suggesting that the C3-nucleophilicity of indole moiety was crucial for this cascade (*eq. b*). Alternatively, the related 2-indole-alkenyl ester **1a-2** was used as starting reactant under the standard PTC conditions, the Michael addition product **3a-2** was obtained, and moreover this compound could be quantitatively transformed into eight-membered [3.3.1] ring system **4a-2** via intramolecular Friedel-Crafts reaction by Lewis acid, suggesting that Friedel-Crafts process was involved in this cascade (*eq. c*). When the nitro-group ($NO_2$) substituted 2-indole-alkenyl **1a-3** was employed as substrate for the model reaction in the presence of catalyst **P0** under standard PTC condition, the [3 + 2] cyclization product **3a-3** was obtained with 84% yield, and notably this intermediate **3a-3** could not be converted into the corresponding eight-membered [3.3.1] ring under the above acid conditions, suggesting that the importance of ketone moiety for ring-opening step (*eq. d*). Besides, the monosubstituted 2-indole-alkenyl ester **1a-4** was used as a model substrate under the standard PTC conditions, the [3 + 2] cyclization reaction could not occur, which might be due to the low reactivity of **1a-4** itself *(eq. e).*

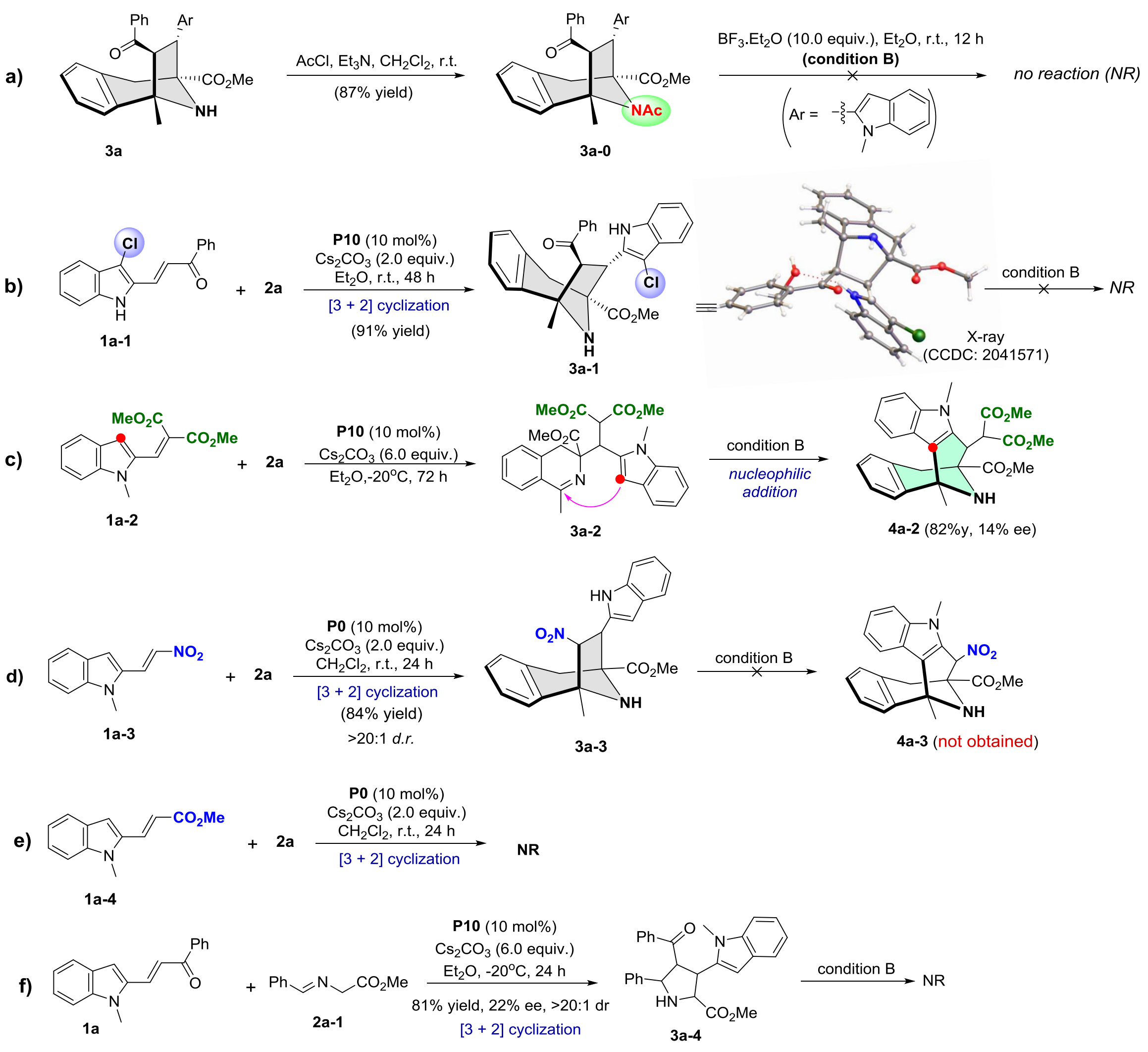


## Methyl(*5S,6S,7S,8S*)-6-benzoyl-5-methyl-7-(1-methyl-1H-indol-2-yl)-5,6,7,9-tetrahydro-8H-5,8-epiminobenzo[7]annulene-8-carboxylate (3a)

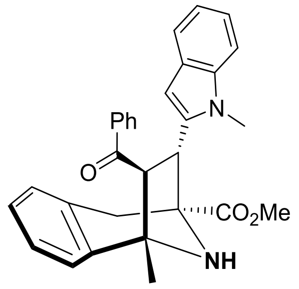

**3a**

A white solid; 43.6 mg, 94% yield; m.p. = 173.6-174.4 °C, $[\alpha]^{25}_D$ = -29.62 (*c* 0.22 $CHCl_3$); *dr* >20:1; $^1$H NMR (400 MHz, Acetone) δ 7.98 (dd, *J* = 8.2, 1.2 Hz, 2H),

7.70 – 7.66 (m, 1H), 7.59 – 7.56 (m, 2H), 7.47 (dd, *J* = 6.2, 2.8 Hz, 1H), 7.48 – 7.32 (m, 2H), 7.33 (d, *J* = 8.8 Hz, 2H), 7.21 (d, *J* = 6.4 Hz, 1H), 7.15 – 7.09 (m, 1H), 6.99–6.96 (m, 1H), 5.76 (s, 1H), 4.75 (d, *J* = 6.0 Hz, 1H), 4.64 (d, *J* = 6.0 Hz, 1H), 3.93 (s, 3H), 3.64 (s, 3H), 3.57 (s, 1H), 3.16 (d, *J* = 17.0 Hz, 1H), 3.05 (d, *J* = 17.0 Hz, 1H), 1.49 (s, 3H); $^{13}$C NMR (100 MHz, Acetone) δ 201.25, 174.41, 145.76, 139.29, 138.60, 138.32, 134.45, 133.94, 130.03, 129.90, 129.17, 128.40, 128.09, 127.45, 122.54, 121.90, 120.58, 120.15, 110.33, 100.97, 69.89, 68.61, 67.35, 53.01, 52.03, 35.14, 21.11; HRMS (ESI) *m/z* calcd for $C_{30}H_{28}N_2O_3[M+H]^+$ = 465.2178, found = 465.2182; The ee value was >99%, $t_R$ (major) = 20.6 min, $t_R$ (minor) = 24.5 min (Chiralcel IF, λ = 254 nm, 15% i-PrOH/hexane, flow rate = 1.0 mL/min).

**<Chromatogram>**

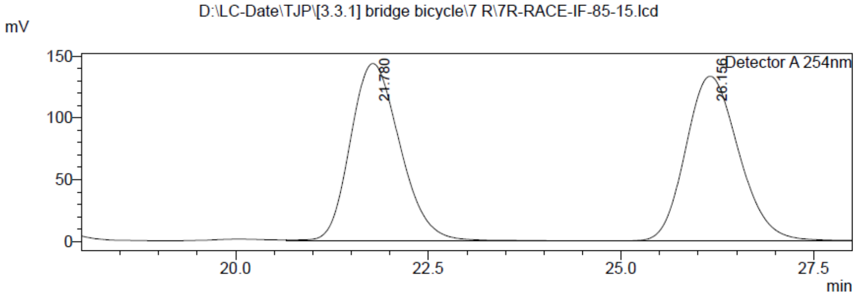


**<Peak Table>**

Detector A 254nm

| Peak# | Ret. Time | Height | Height% | Conc. | Area | Area% |
|---|---|---|---|---|---|---|
| 1 | 21.780 | 143751 | 51.867 | 50.145 | 6450275 | 50.145 |
| 2 | 26.156 | 133400 | 48.133 | 49.855 | 6412847 | 49.855 |
| Total | | 277151 | 100.000 | | 12863122 | 100.000 |

Racemic **3a**

**<Chromatogram>**

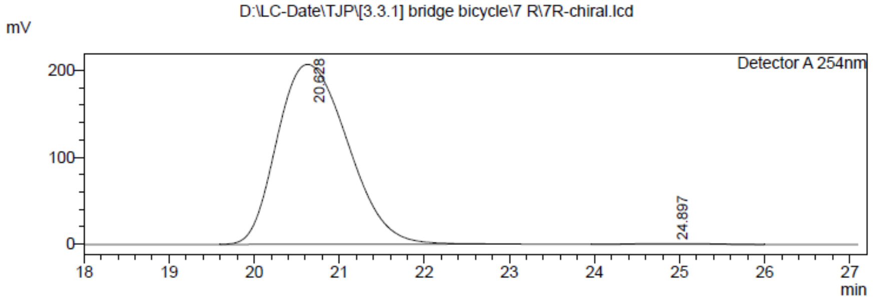


**<Peak Table>**

Detector A 254nm

| Peak# | Ret. Time | Height | Height% | Conc. | Area | Area% |
|---|---|---|---|---|---|---|
| 1 | 20.628 | 207055 | 99.565 | 99.563 | 11922764 | 99.563 |
| 2 | 24.897 | 905 | 0.435 | 0.437 | 52288 | 0.437 |
| Total | | 207959 | 100.000 | | 11975052 | 100.000 |

Enantiomerically enriched **3a**

**Methyl(5S,6S,7S,8S)-10-acetyl-7-argio-6-benzoyl-5-methyl-5,6,7,9-tetrahydro-8H-5,8-epiminobenzo[7]annulene-8-carboxylate(3a-0)**

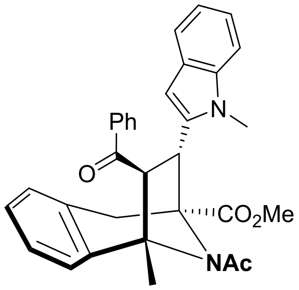


**3a-0**

To a dried round bottle flask with a magnetic stirring bar were added **3a** (92.8 mg, 0.2 mmol) in 5 mL $CH_2Cl_2$ at 0 $^oC$, Then triethylamine (0.5 mmol) and AcCl (0.3 mmol) were added successively, the reaction mixture was stirred at 0 $^oC$ for 5 h, and TLC show that the reaction was completed. The resulting mixture was then quenched with saturated $NaHCO_3$ solution and extracted with $CH_2Cl_2$ three times. The combined organic layers were dried over $Na_2SO_4$. The solvent was removed under reduce pressure. The residue was purified by column chromatography on silica gel to afford **3a-0**. A white solid; 88.1 mg, 87% yield; *dr* >20:1. $^1H$ NMR (400 MHz, $CDCl_3$) δ 7.83 (d, *J* = 7.2 Hz, 2H), 7.56 (t, *J* = 7.2 Hz, 1H), 7.43 (dd, *J* = 14.0, 8.0 Hz, 3H), 7.34 (td, *J* = 13.4, 6.8 Hz, 2H), 7.27 (d, *J* = 4.4 Hz, 1H), 7.23 (d, *J* = 7.6 Hz, 2H), 7.16 (t, *J* = 7.2 Hz, 1H), 7.03 (t, *J* = 7.2 Hz, 1H), 5.93 (s, 1H), 4.99 (d, *J* = 7.4 Hz, 1H), 4.61 (d, *J* = 7.4 Hz, 1H), 3.91 (s, 3H), 3.80 (d, *J* = 18.0 Hz, 1H), 3.59 (s, 3H), 3.08 (d, *J* = 18.0 Hz, 1H), 2.01 (s, 3H), 1.92 (s, 3H); $^{13}C$ NMR (100 MHz, $CDCl_3$) δ 199.09, 172.99, 171.57, 144.81, 137.88, 137.82, 135.96, 134.19, 133.94, 130.75, 129.19, 128.68, 128.36, 127.33, 126.66, 121.84, 121.06, 120.24, 119.79, 109.68, 101.81, 70.06, 65.70， 65.61, 52.67, 44.49, 30.64, 29.51, 24.97, 21.82; HRMS (ESI) *m/z* calcd for $C_{32}H_{30}N_2O_4$ $[M + H]^+$ = 507.2278, found = 507.2276.

**Methyl(5S,6S,7S,8S)-6-benzoyl-7-(3-chloro-1H-indol-2-yl)-5-methyl-5,6,7,9-tetrahydro-8H-5,8-epiminobenzo[7]annulene-8-carboxylate(3a-1)**

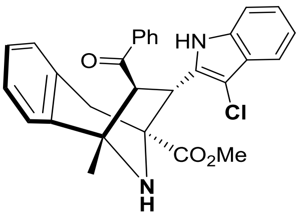

**3a-1**

To a dried round bottle flask with a magnetic stirring bar were added **2a** (24.2 mg, 0.12 mmol) and **1a-1** (28.2 mg, 0.1 mmol), followed by the addition of $Cs_2CO_3$ (65 mg, 0.2 mmol ) and catalyst **P10** (13.9 mg, 10 mol%), followed by the addition of $Et_2O$ (2 mL). The reaction mixture was stirred at rt for 48 h to afford **3a-1**; A white solid; 44.0 mg, 91% yield; *dr* >20:1;The Structure was confirmed by single crystal (see Supplementary Table S10).

**Dimethyl-2-((3-(methoxycarbonyl)-1-methyl-3,4-dihydroisoquinolin-3-yl)(1-methyl-1H-indol-2-yl)methyl)malonate(3a-2)**

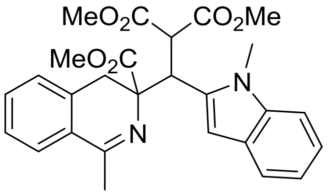

**3a-2**

To a dried round bottle flask with a magnetic stirring bar were added **2a** (24.2 mg, 0.12 mmol) and **1a-2** (27.3 mg, 0.1 mmol), followed by the addition of $Cs_2CO_3$ (65.2 mg, 0.2 mmol ) and catalyst $Ph_2Me_2P^+I^-$ (**P0**) (3.42 mg, 10 mol%), followed by the addition of $CH_2Cl_2$ (2 mL).The reaction mixture was stirred at rt for 24 h to afford **3a-2**; A white solid; 43.3 mg, 91% yield; *dr* >20:1; $^1$H NMR (400 MHz, $CDCl_3$) δ 7.51 (d, $J$ = 7.6 Hz, 1H), 7.40 (d, $J$ = 7.2 Hz, 1H), 7.30 (d, $J$ = 8.4 Hz, 1H), 7.25 – 7.14 (m, 3H), 7.04 (d, $J$ = 7.6 Hz, 1H), 6.99 (d, $J$ = 7.2 Hz, 1H), 6.41 (s, 1H), 4.93 (d, $J$ = 10.4 Hz, 1H), 4.65 (d, $J$ = 10.4 Hz, 1H), 3.91 (s, 3H), 3.73 (s, 3H), 3.45 (s, 3H), 3.33 (s, 3H), 3.01 (d, $J$ = 15.2 Hz, 1H), 2.52 (d, $J$ = 15.2 Hz, 1H), 2.50 (s, 3H); $^{13}$C NMR (100 MHz, $CDCl_3$) δ 172.97, 168.98, 168.62, 166.65, 137.83, 136.87, 134.75, 131.08, 129.36, 127.81, 127.72 , 127.30, 125.43, 121.30, 120.48, 119.46, 109.47, 102.21, 67.55, 54.60, 52.86, 52.60, 52.56, 43.33, 33.89, 29.99, 23.68; HRMS (ESI) *m/z* calcd for $C_{27}H_{28}N_2O_6$: $[M + H]^+$ = 477.2020, found = 477.2018.

**Dimethyl-2-(6-(methoxycarbonyl)-8,13-dimethyl-6,7,8,13-tetrahydro-5H-6,13-epiminobenzo[4,5]cycloocta[1,2-b]indol-7-yl)malonate(4a-2)**

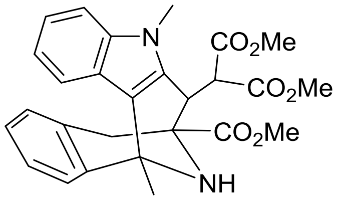


**4a-2**

To a dried round bottle flask with a magnetic stirring bar were added **3a-2** (47.7 mg, 0.1 mmol) with 2 ml $CH_2Cl_2$. Then $BF_3 \cdot Et_2O$ (1.0 mmol, 10 equiv.) was added to the mixture, The reaction mixture was stirred at rt for 12 h to afford **4a-2**; A white solid; 46.6 mg, 98% yield, >20:1 *dr;* $^1$H NMR (400 MHz, $CDCl_3$) δ 7.67 (d, $J$ = 7.2 Hz, 1H), 7.57 (d, $J$ = 8.0 Hz, 1H), 7.20 (t, $J$ = 8.4 Hz, 2H), 7.11 (td, $J$ = 7.2, 0.8 Hz, 1H), 7.05 (td, $J$ = 7.6, 1.2 Hz, 1H), 7.02 – 6.98 (m, 1H), 6.91 (d, $J$ = 7.6 Hz, 1H), 4.33 (d, $J$ = 3.6 Hz, 1H), 4.02 (d, $J$ = 3.6 Hz, 1H), 3.84 (s, 3H), 3.77 (s, 3H), 3.71 (s, 3H), 3.16 (s, 3H), 3.12 (d, $J$ = 17.6 Hz, 1H), 2.56 (d, $J$ = 17.6 Hz, 1H), 2.42 (s, 1H), 2.10 (s, 3H); $^{13}$C NMR (100 MHz, $CDCl_3$) δ 174.41, 169.98, 168.77, 145.86, 138.11, 133.48, 131.89 , 129.11, 126.35, 126.32, 123.92, 121.65, 121.39, 119.34, 119,19, 113.43, 109.41, 62.67, 54.61, 53.94, 52.96, 52.48, 43.02, 36.95, 29.99, 24.29, 22.77; HRMS (ESI) *m/z* calcd for $C_{27}H_{28}N_2O_6$: $[M + H]^+$ = 477.2020, found = 477.2019. The ee value was 14%, $t_R$ (major) = 12.9 min, $t_R$ (minor) = 13.9 min (Chiralcel IA, λ = 254 nm, 10% i-PrOH/hexane, flow rate = 1.0 mL/min).

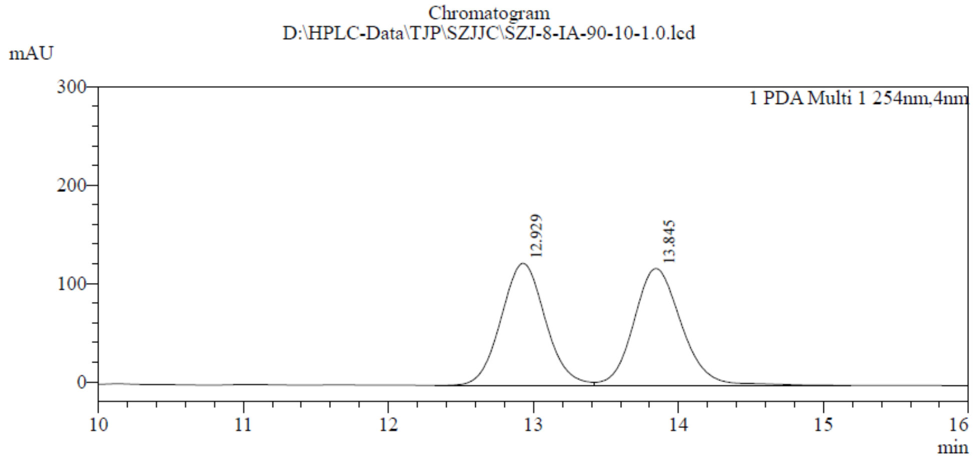


Peak Table

PDA Ch1 254nm

| Peak# | Ret. Time | Height | Height% | Area | Area% |
|---|---|---|---|---|---|
| 1 | 12.929 | 123992 | 51.068 | 2634552 | 49.258 |
| 2 | 13.845 | 118806 | 48.932 | 2713920 | 50.742 |
| Total | | 242798 | 100.000 | 5348472 | 100.000 |

Racemic **4a-2**

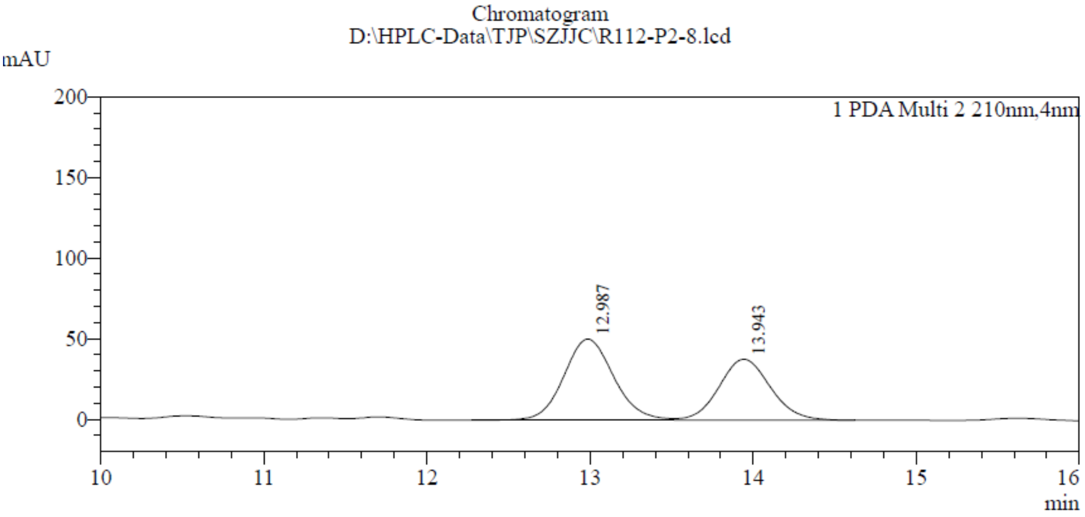


Peak Table

PDA Ch2 210nm

| Peak# | Ret. Time | Height | Height% | Area | Area% |
|---|---|---|---|---|---|
| 1 | 12.987 | 50291 | 57.091 | 1074051 | 56.504 |
| 2 | 13.943 | 37799 | 42.909 | 826793 | 43.496 |
| Total | | 88090 | 100.000 | 1900844 | 100.000 |

Enantiomerically enriched **4a-2**

**<u>5-methyl-7-(1-methyl-1H-indol-2-yl)-6-nitro-5,6,7,9-tetrahydro-8H-5,8-epiminobenzo[7]annulene-8-carboxylate(3a-3)</u>**

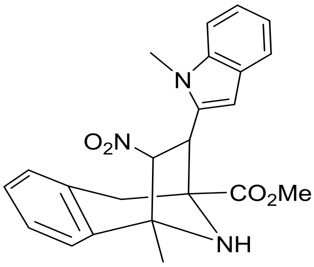


**3a-3**

To a dried round bottle flask with a magnetic stirring bar were added **2a** (24.2 mg, 0.12 mmol) and **1a-3** (20.2 mg, 0.1 mmol), followed by the addition of $Cs_2CO_3$ (65.2 mg,0.2 mmol ) and $Ph_2Me_2P^+I^-$ (**P0**) (3.42 mg, 10 mol%), followed by the addition of $CH_2Cl_2$ (2 mL).The reaction mixture was stirred at rt for 24 h to afford **3a-3**; A yellow solid; 34.1 mg, 84% yield; *dr* >20:1; $^1H$ NMR (400 MHz, $CDCl_3$) δ 7.42 (d, *J* = 7.2 Hz, 1H), 7.39 – 7.32 (m, 3H), 7.26 (d, *J* = 8.0 Hz, 1H), 7.20 (td, *J* = 7.4, 0.8 Hz, 1H),

7.05 (t, $J$ = 7.4 Hz, 2H), 5.33 (d, $J$ = 2.4 Hz, 2H), 4.77 (d, $J$ = 4.0 Hz, 1H), 3.91 (s, 3H), 3.58 (s, 3H), 3.18 (d, $J$ = 17.2 Hz, 1H), 2.88 (d, $J$ = 17.2 Hz, 1H), 1.73 (s,3H); $^{13}$C NMR (100 MHz, $CDCl_3$) δ 172.85, 139.22, 137.49, 135.12, 133.26, 129.48, 128.75, 127.43, 123.22, 122.14, 120.52, 120.05, 109.60, 104.60, 101.38, 77.36, 70.00, 67.69, 53.19, 52.55, 34.35, 29.74, 19.12; HRMS (ESI) *m/z* calcd for $C_{23}H_{23}N_3O_4$: [M + H]$^+$ = 406.1761, found = 406.1762.

**methyl4-benzoyl-3-(1-methyl-1H-indol-2-yl)-5-phenylpyrrolidine-2-carboxylate(3a-4)**

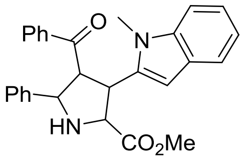


**3a-4**

To a dried round bottle flask with a magnetic stirring bar were added **1a** (26.1 mg, 0.1 mmol) and **1a-4** (21.2 mg, 0.12 mmol), followed by the addition of $Cs_2CO_3$ (65.2 mg, 0.2 mmol ) and **P10** (13.9 mg, 10 mol%), followed by the addition of $Et_2O$ (2 mL).The reaction mixture was stirred at -20$^o$C for 24 h to afford **3a-4**; 81% yield; 22% ee, *dr* >20:1; $^1$H NMR (400 MHz, $CDCl_3$) δ 7.60 (d, $J$ = 7.8 Hz, 1H), 7.53 (d, $J$ = 8.6 Hz, 2H), 7.39 (t, $J$ = 7.4 Hz, 1H), 7.30 (d, $J$ = 8.2 Hz, 1H), 7.25 – 7.18 (m, 3H), 7.14 – 7.07 (m, 4H), 7.07 – 7.03 (m, 2H), 6.59 (s, 1H), 5.00 (d, $J$ = 8.2 Hz, 1H), 4.50 (dd, $J$ = 8.2, 6.1 Hz, 1H), 4.43 (t, $J$ = 7.0 Hz, 1H), 4.36 (d, $J$ = 7.8 Hz, 1H), 3.81 (s, 3H), 3.72 (s, 3H), 3.15 (s, 1H). $^{13}$C NMR (100 MHz, $CDCl_3$) δ 199.48, 173.15, 141.16, 138.19, 137.48, 137.23, 133.06, 128.36, 128.70, 128.22, 127.86, 127.80, 127.39, 121.47, 120.27, 119.80, 109.42, 98.36, 67.24, 66.48, 60.61, 52.68, 44.02, 29.95. HRMS (ESI) *m/z* calcd for $C_{28}H_{26}N_2O_3$ [M + H]$^+$ = 439.2022, found = 439.2030. The ee value was 22%, $t_R$ (major) = 30.3 min, $t_R$ (minor) = 17.8 min (Chiralcel ODH, λ = 254 nm, 20% i-PrOH/hexane, flow rate = 1.0 mL/min).

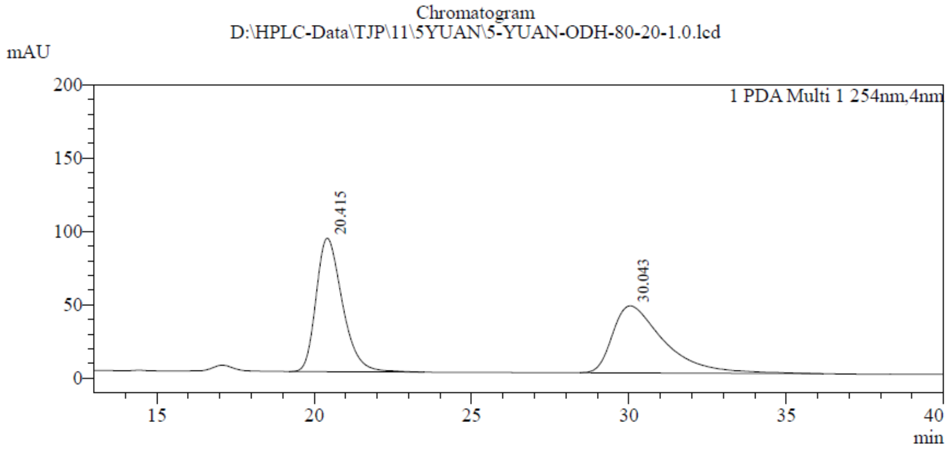


Peak Table

PDA Ch1 254nm

| Peak# | Ret. Time | Height | Height% | Area | Area% |
|---|---|---|---|---|---|
| 1 | 20.415 | 90901 | 66.564 | 5280386 | 50.318 |
| 2 | 30.043 | 45661 | 33.436 | 5213665 | 49.682 |
| Total | | 136562 | 100.000 | 10494051 | 100.000 |

Racemic 3**a-4**

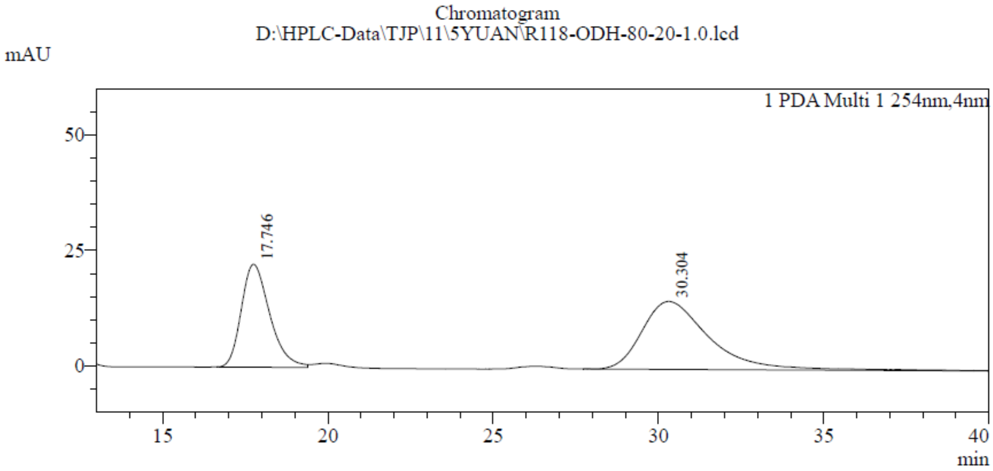


Peak Table

PDA Ch1 254nm

| Peak# | Ret. Time | Height | Height% | Area | Area% |
|---|---|---|---|---|---|
| 1 | 17.746 | 22283 | 60.214 | 1335537 | 38.860 |
| 2 | 30.304 | 14723 | 39.786 | 2101221 | 61.140 |
| Total | | 37006 | 100.000 | 3436759 | 100.000 |

Enantiomerically enriched **3a-4**

**Methyl(6R,7R,13S)-8,13-dimethyl-7-(2-oxo-2-phenylethyl)-5,7,8,13-tetrahydro-6H-6,13-epiminobenzo[4,5]cycloocta[1,2-b]indole-6-carboxylate (*ent*-4a)**

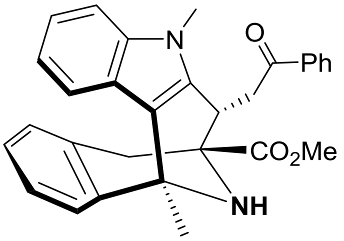

*ent*-**4a**

A white solid; 43.0 mg, 93% yield; m.p. =170.5-172.1 $^{o}$C, $[\alpha]^{25}_{D}$ = +75.21 (*c* 0.30, $CHCl_3$); *dr* >20:1; HRMS (ESI) *m/z* calcd for $C_{30}H_{28}N_2O_3$ $[M+H]^+$ = 465.2173, found = 465.2176; The ee value was >99%, $t_R$ (major) = 16.6 min, $t_R$ (minor) = 10.9 min (Chiralcel IA, λ = 254 nm, 10% i-PrOH/hexane, flow rate = 1.0 mL/min).

**<Chromatogram>**

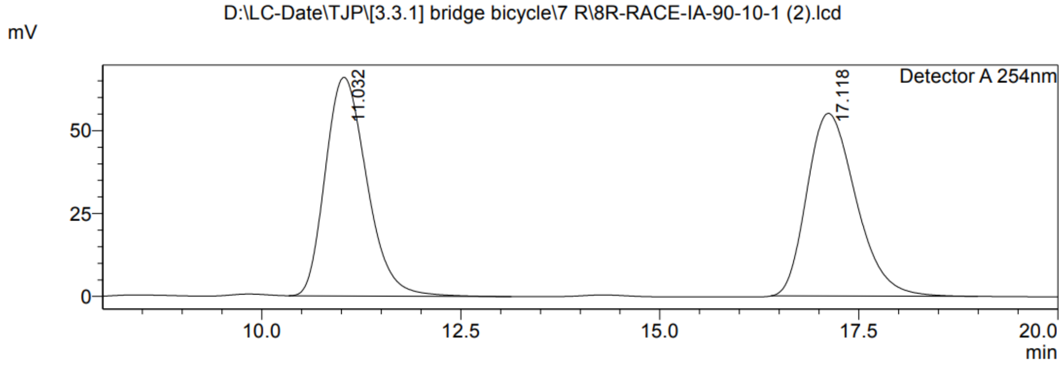


**<Peak Table>**

Detector A 254nm

| Peak# | Ret. Time | Height | Height% | Conc. | Area | Area% |
|---|---|---|---|---|---|---|
| 1 | 11.032 | 65910 | 54.469 | 50.287 | 2393672 | 50.287 |
| 2 | 17.118 | 55095 | 45.531 | 49.713 | 2366386 | 49.713 |
| Total | | 121006 | 100.000 | | 4760058 | 100.000 |

Racemic ***ent*-4a**

**<Chromatogram>**

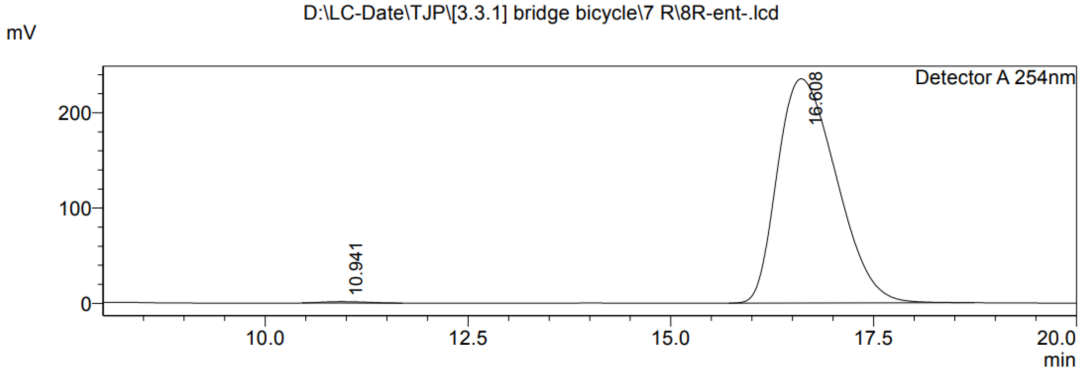


**<Peak Table>**

Detector A 254nm

| Peak# | Ret. Time | Height | Height% | Conc. | Area | Area% |
|---|---|---|---|---|---|---|
| 1 | 10.941 | 1429 | 0.603 | 0.456 | 54539 | 0.456 |
| 2 | 16.608 | 235421 | 99.397 | 99.544 | 11900208 | 99.544 |
| Total | | 236850 | 100.000 | | 11954747 | 100.000 |

Enantiomerically enriched ***ent*-4a**

## **C).** $^{1}$H NMR titration experiments

In order to understand the stereoselectivity control for the bifunctional phosphonium salt catalysted [3 + 2] cyclization reaction, $^{1}$H NMR titration experiments were carried out. Generally, titration of 2-indole-vinyl ketone substrate **1a** to the optimal catalyst **P10** led to obvious pronounced change in the position of thiourea-$NH_1$ signal of catalyst (Supplementary Figure S5), while titration of cyclic azomethine ylide **2a** to catalyst **P10** led to a less change (Supplementary Figure S6). These results clearly suggested that there have strong H-boing interactions between the catalyst **P10** and **1a** (Supplementary Figure S7).

**General procedures**: $^{1}$H NMR titration experiments were conducted on a Bruker spectrometer by recording the changes of $^{1}$H NMR spectra of the catalyst **P10** after the corresponding targets addition. In a typical experiment: the catalyst **P10** (41.9 mg, 0.03 mmol) was dissolved with DMSO-*d6* (0.5 mL) in nuclear magnetic tube. Then, the $^{1}$H NMR spectrum of catalyst **P10** was recorded. After that, the corresponding equivalent of substrate (**1a** or **2a**) was stepwise added to the NMR-tube and chemical shifts of the protons on the catalyst **P10** were recorded after each addition.

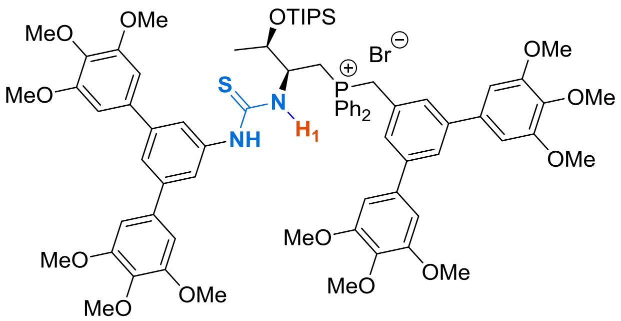


**P10**

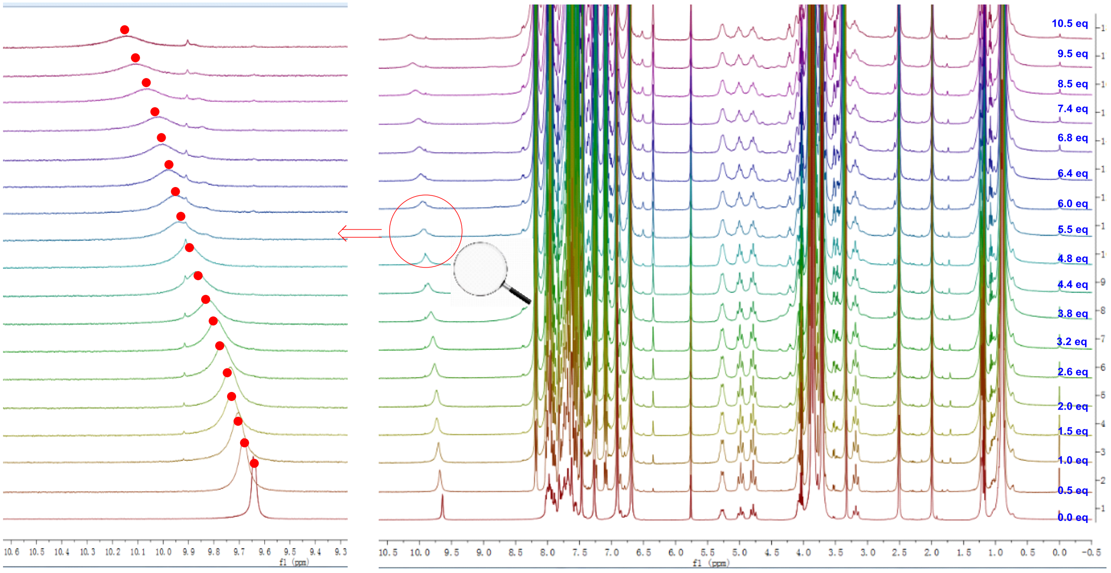


**Supplementary Figure S5.** $^{1}$H NMR titration of catalyst **P10** with **1a**

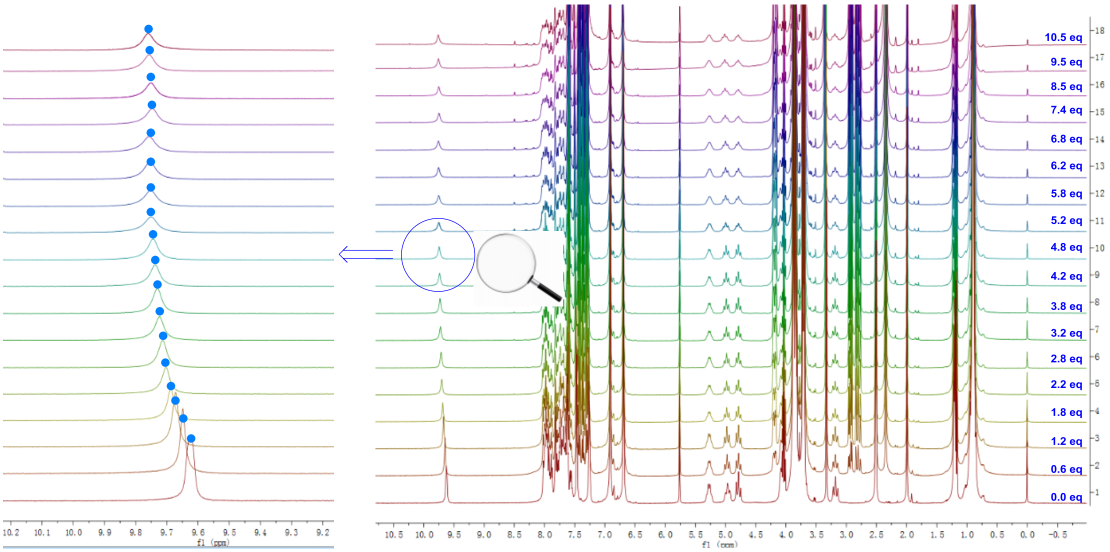


**Supplementary Figure S6.** $^{1}$H NMR titration of catalyst **P10** with **2a**

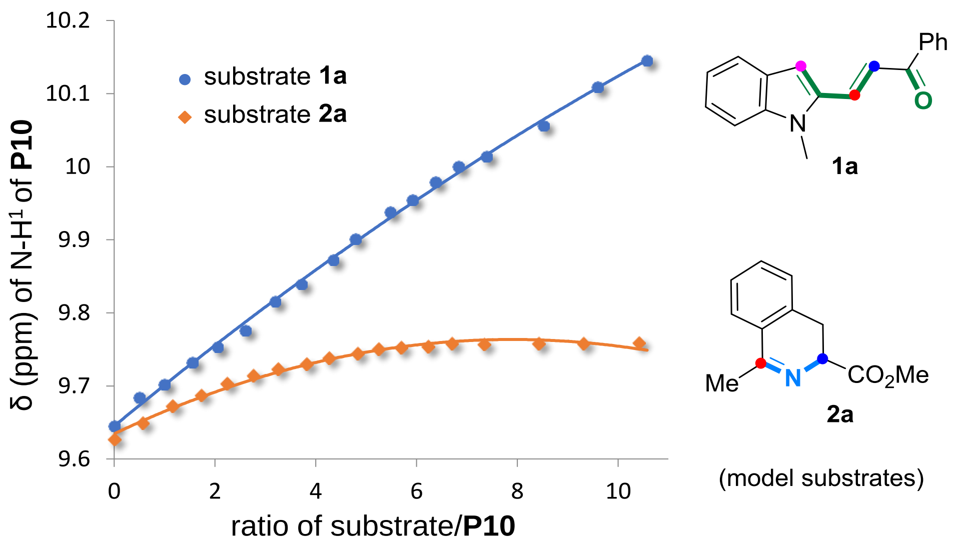


**Supplementary Figure S7.** The $^{1}$H NMR titration curve (Sample size, n = 18)

## D). Model reaction catalyzed by different phosphonium salts

We also prepared the methylated of thiourea-NH catalyst **P10'** and tested for the model reaction. When methylated phosphonium salts **P10'** was used, the model reaction became much slower and its enantioselectivity decreased dramatically, the racemic product was obtained with 85% yield. Of note, when the model reaction was performed in methanol, the enantioselectivity was also dropped down to 0% ee. These preliminary results indicated the importance of both hydrogen-bonding and ion-pair interactions in this phase-transfer system (Supplementary Figure S8).

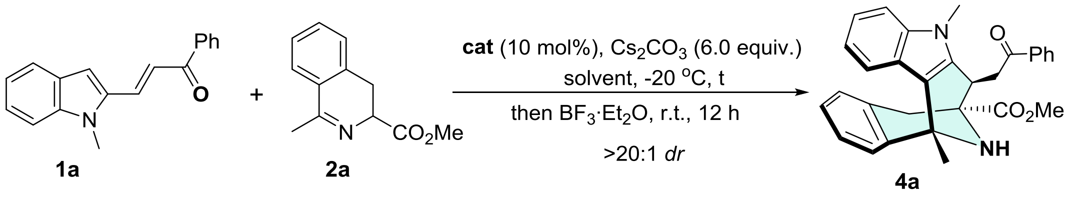


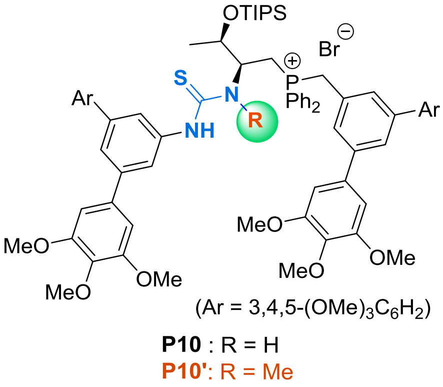


| cat. | sol. | t (h) | results (4a) |
|---|---|---|---|
| **P10** | $Et_2O$ | 72 h | 94% y, >99% ee |
| **P10'** | $Et_2O$ | 72 h | 85% y, 0% ee |
| **P10** | MeOH | 64 h | 93% y, 0% ee |

**Supplementary Figure S8.** Model reaction catalyzed by different phosphonium salts

## E). Proposed transition state models for the [3 + 2] cycloaddition

According to these results and our previous studies,[3] the probable transition state models for the first step of [3 + 2] cycloaddition reaction was proposed in Supplementary Figure S9, in which the thiourea moiety of the catalyst interacts with 2-indole-vinyl ketone **1a** through hydrogen-bonding interactions that contribute significantly to the formation of the observed major stereoisomer. In addition, the phosphonium cation of catalyst **P10** particularly possessed an attractive semi-enclosed cavity, and may have strong ion-pair interaction with the anionic substrate. Besides. the bifunctional phosphonium salt catalyst **P10** also has fascinating electrostatic potential (ESP) with an electropositive region (Supplementary Figure S10), which would be favourable for controlling the stereoselectivities of this cascade reaction.

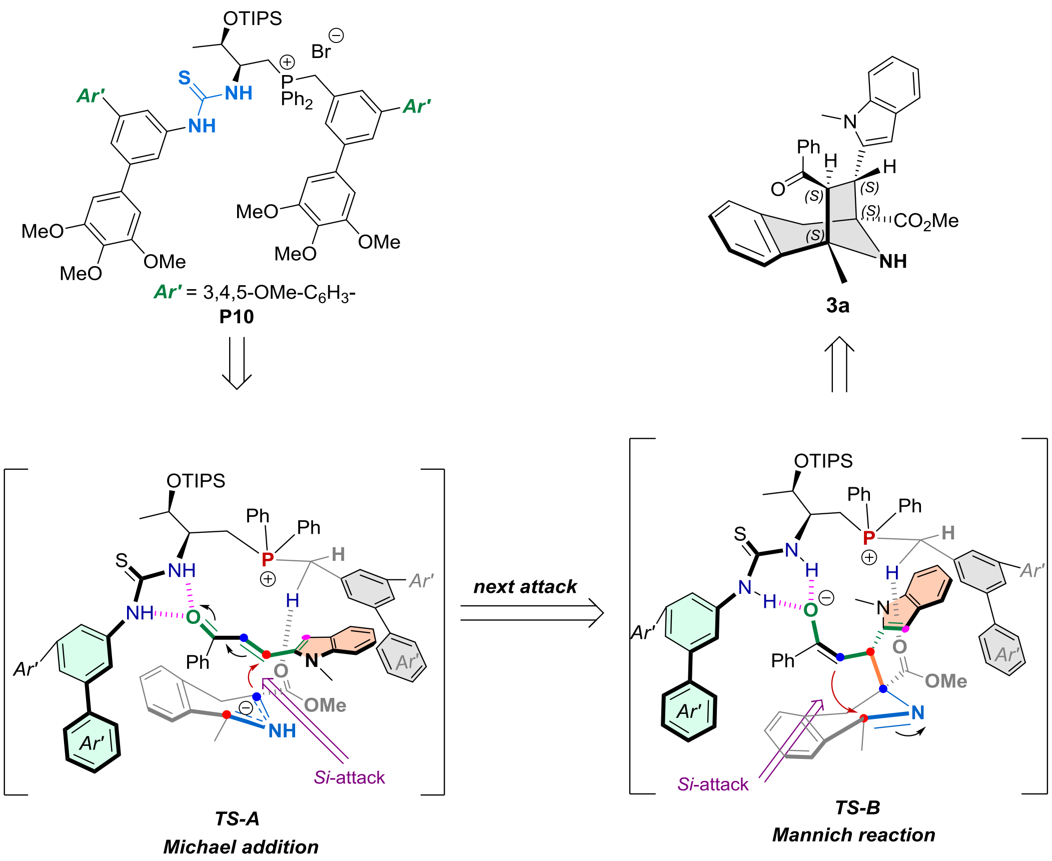


**Supplementary Figure S9.** Proposed transition state models for the [3 + 2] cycloaddition by bifunctional phosphonium salt catalyst **P10**.

## 12. Computational investigations

### A). Computational Methods

All of the DFT calculations were carried out with the Gaussian 16 series of programs[4] in the solution phase (solvent = diethylether) with the SMD[5] (solvation-model density) solvent model. The B3LYP functional[6] with the standard def2-SVP basis set was used for geometry optimizations. Harmonic vibrational frequency calculations were performed for all stationary points to determine whether they are local minima or transition structures and to derive thermochemical corrections for the enthalpies and free energies. The M06-2X[7] functional with the def2-TZVP basis set was used to calculate the single-point energies as well as high-level DLPNO-CCSD(T)/def2-TZVP method implemented by SMD implicit solvent model in ORCA[8]. The energies reported in this paper are the M06-2X

calculated Gibbs free energies in diethylether solvent that are based on the B3LYP calculated geometries with thermodynamic corrections calculated at the same level. The electrostatic potential (ESP) surface was calculated using GaussView. The 3D diagrams of molecules were generated using CYLView [9] .

**B). Electrostatic potential surface of the catalyst P10**

To further demonstrate electrical character of the catalyst **P10**, the electrostatic potential (ESP) surface was calculated at the B3LYP/def2-SVP level of theory (Supplementary Figure S10). The electrostatic potential (ESP) surface shows that hydrogen atoms on thiocarbamide moiety and the phosphonium cation can form an electropositive region (blue color in the ESP map), which enables strong interactions between the phosphonium cation and the sulfourea moiety on the catalyst (blue color in the center of the ESP map). The results indicate that the geometry of the catalyst **P10** shows strong electropositive character.

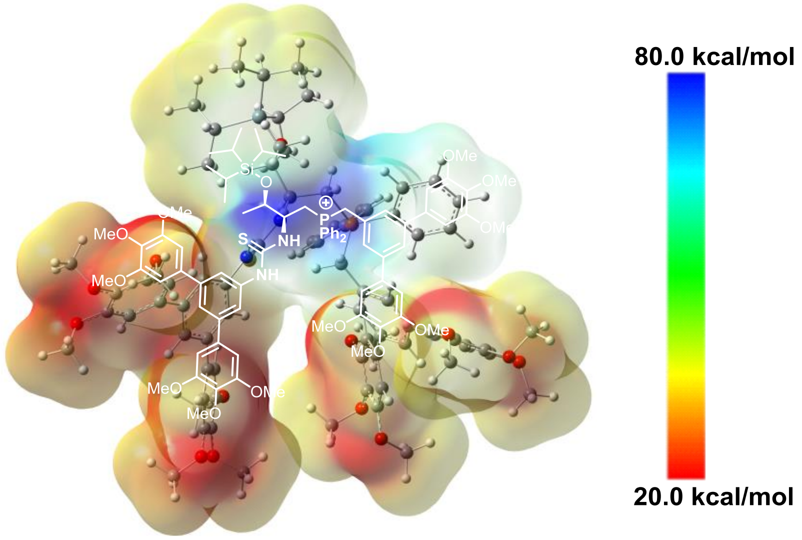


**Supplementary Figure S10.** Electrostatic potential surface of the catalyst **P10**. Energy values are given in kcal/mol

**C). Free-energy profiles for Lewis acid catalyzed ring-opening/Friedel-Crafts cascade process**

In our theoretical calculations, seven-membered ring complex **3a** was chosen as reactants in model reaction, which can deliver azabicyclo[3.3.1]nonane derivatives **4a**

in experimental observations, where seven-membered ring complex **3a** was set as the relative zero point. As depicted in Supplementary Figure S11, ligand exchange between boron trifluoride ethyl ether complex and substrate **3a** gives carbonyl-coordinated complex **D** with an endothermic energy of 3.6 kca/mol, which can attribute to the weak coordination ability of carbonyl. In complex **D**, the coordination with the Lewis acidic boron trifluoride catalyst facilitates electron density flow from the N-H moiety to the carbonyl moiety in substrate **3a**, which significantly activate the C-C bond. The cleavage of C-C bond then can smoothly occur via transition state **TS1** with an energy barrier of 7.5 kca/mol, in which the iminium species **E** is generated. In the geometry of the transition state **TS1** (Supplementary Figure S12), the lengths of the breaking C-C bond is 2.13 Å. Subsequently, the intramolecular Friedel–Crafts-type reaction take place via transition state **TS2** with an energy barrier of 9.3 kca/mol to generated cationic species **F**. In the geometry of the **TS2** (Supplementary Figure S12), the length of the forming new C-C bond is 2.04 Å. Aromatization of the latter through loss of a proton then affords the eight-membered ring complex **G**. Finally, the ligand exchange with the diethyl ether yields azabicyclo[3.3.1]nonane product **4a** with the regeneration of active catalytic species boron trifluoride ethyl ether, which is exergonic by 5.1 kcal/mol, thus completing the catalytic cycle. It should also be noted that the calculated results shows the free energy difference between **3a** and **4a** is 5.5 kcal/mol, which indicate that the ring-opening/Friedel-Crafts is thermodynamically favored, since the seven-membered ring complex **3a** is less stable than the eight-membered ring complex **4a**. The calculations indicate that the C-C bond breaking step is the rate-determining step in the catalytic cycle, and the overall activation free energy for this reaction is 11.1 kcal/mol.

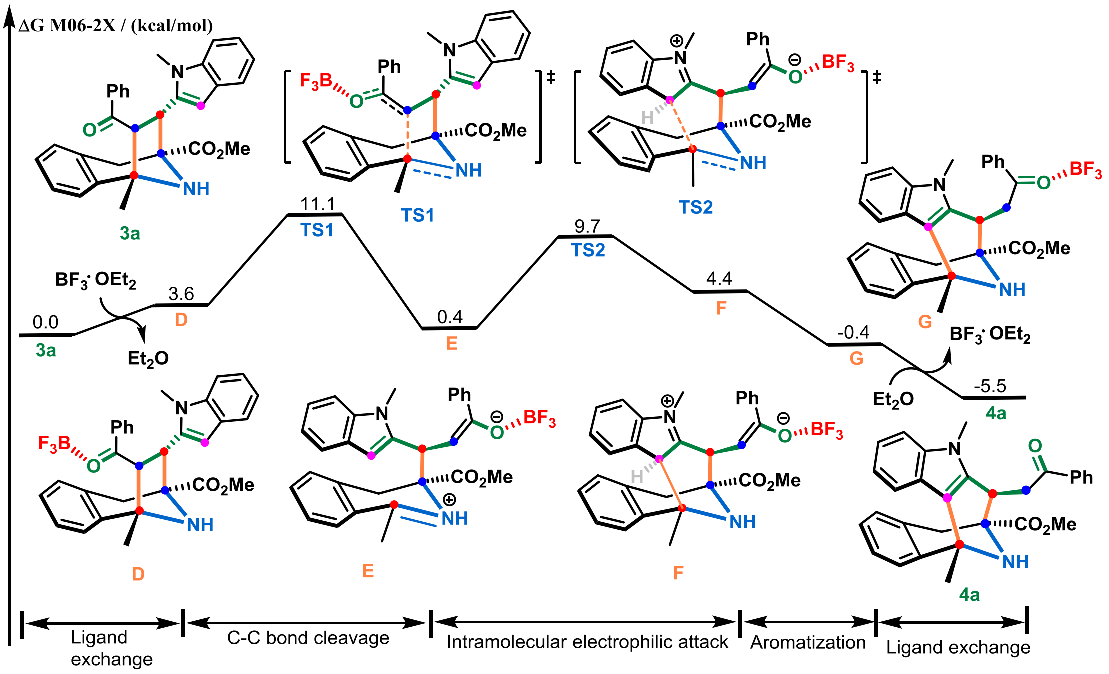


**Supplementary Figure S11.** Free-energy profiles for Lewis acid catalyzed ring-opening/Friedel-Crafts cascaded process.

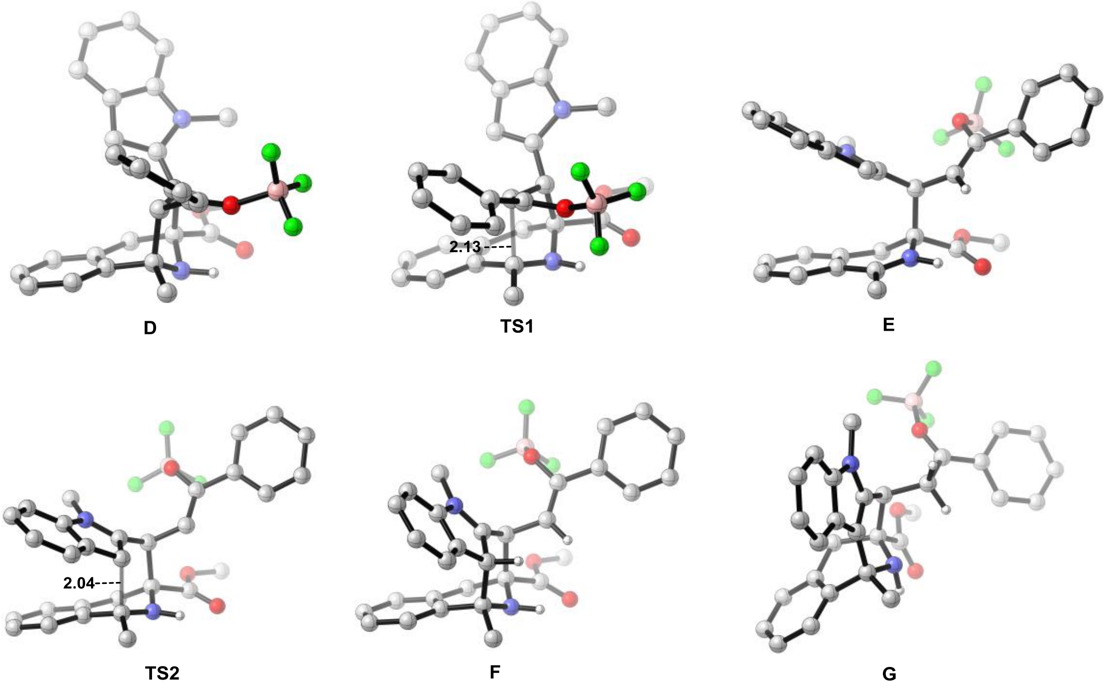


**Supplementary Figure S12.** Optimized structures for Lewis acid catalyzed ring-opening/Friedel-Crafts cascade process.

### D). Free-energy profiles for Brønsted acid catalyzed ring-opening/Friedel-Crafts cascade process

We also used the same method to explore the mechanism of Brønsted acid catalyzed ring-opening/ Friedel-Crafts cascade process of seven-membered ring substrate **3a** generate azabicyclo[3.3.1]nonane derivatives **4a**. As shown in Supplementary Figure S13, the direct protonation carbonyl moiety of substrate **3a** by **$Et_2O$-$H^+$** gives oxinum ionic intermediate **D-1** with an exothermic energy 5.8 kcal/mol. In the geometry of the oxinum ionic intermediate **D-1** (Supplementary Figure S13) , the lengths of the **O1-H···O2** bond are 1.65 Å, as well as in the **TS3** and **E-1** (1.86 Å and 1.83 Å, respectively) suggests there are exit hydrogen bond interaction between keto oxygen and carbonyl oxygen moiety of ester group. Afterwards, C-C bond cleavage takes place via transition state **TS3** to generate iminium species **E-1** reversibly with a barrier of 3.3 kcal/mol. Subsequently, the keto-enol tautomerization of **E-1** occurs to form more stable keto intermediate **F-1** irreversibly, which is 28.8 kcal/mol lower in energy. The intramolecular Friedel–Crafts-type reaction take place via transition state **TS4** with an energy barrier of 8.9 kca/mol to generate cationic species **G-1**. Finally, aromatization of species **G-1** through loss of a proton then affords the eight-membered ring product **4a** with the regeneration of active catalytic species **$Et_2O$-$H^+$**.

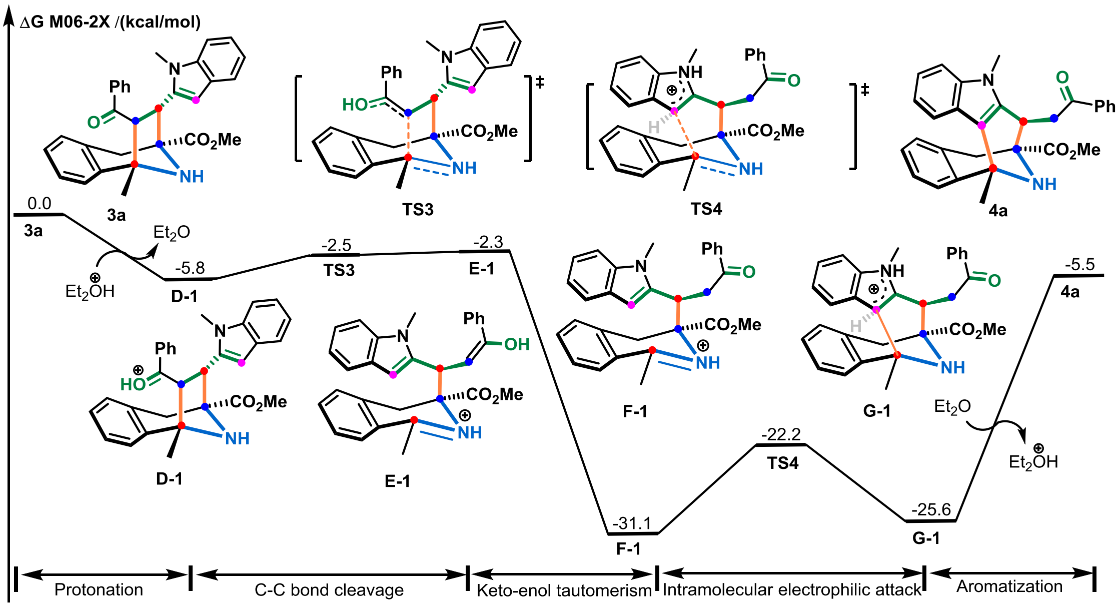


**Supplementary Figure S13.** Free-energy profiles for Brønsted acid catalyzed ring-opening/Friedel-Crafts cascade process

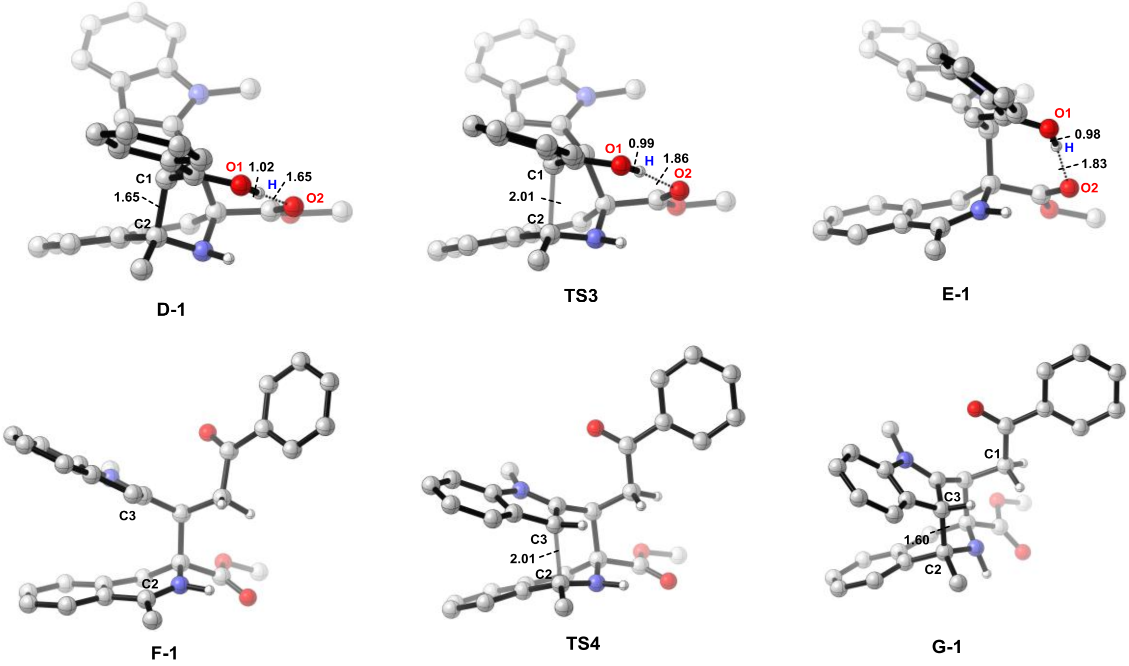


**Supplementary Figure S14.** Optimized structures for Brønsted acid catalyzed ring-opening/Friedel-Crafts cascade process

**Supplementary Table S15.**

SMD$_{(diethylether)}$/M06-2X/def2-TZVP//SMD$_{(diethylether)}$/B3LYP/def2-SVP calculated absolute energies of all structures. Values are given in Hartree.

| Geometry | $\Delta E_{(elec\text{-}B3LYP)}$[1] | $\Delta G_{(corr)}$[2] | $\Delta H_{(corr)}$[3] | $\Delta E_{(solv\text{-}M06\text{-}2X)}$[4] | IF[5] |
|---|---|---|---|---|---|
| **3a** | -1494.204008 | 0.454931 | 0.547467 | -1495.230607 | |
| **$BF_3 \cdot OEt_2$** | -557.840343 | 0.114665 | 0.161857 | -558.281799 | |
| **$Et_2O$** | -233.494702 | 0.105086 | 0.143313 | -233.637527 | |
| **D** | -1818.546747 | 0.464347 | 0.565928 | -1819.868918 | |
| **TS1** | -1818.538014 | 0.462846 | 0.564151 | -1819.855598 | -282.45 |
| **E** | -1818.550562 | 0.460508 | 0.565075 | -1819.870297 | |
| **TS2** | -1818.534229 | 0.463702 | 0.563983 | -1819.858637 | -322.70 |
| **F** | -1818.541426 | 0.466752 | 0.564452 | -1819.870097 | |
| **G** | -1818.553085 | 0.465889 | 0.565951 | -1819.876863 | |
| **4a** | -1494.213243 | 0.455294 | 0.547249 | -1495.239703 | |
| **$Et_2O^+H$** | -233.894939 | 0.118686 | 0.156853 | -234.033117 | |
| **D-1** | -1494.619439 | 0.472016 | 0.559806 | -1495.638911 | |
| **TS3** | -1494.614324 | 0.469117 | 0.558381 | -1495.630696 | -328.37 |
| **E-1** | -1494.636506 | 0.469373 | 0.560804 | -1495.647715 | |
| **F-1** | -1494.661955 | 0.467624 | 0.560287 | -1495.674806 | |

| | | | | | |
|---|---|---|---|---|---|
| **TS4** | -1494.642979 | 0.469143 | 0.558829 | -1495.662148 | -306.59 |
| **G-1** | -1494.646821 | 0.471897 | 0.559268 | -1495.670016 | |

[1]The electronic energy calculated by B3LYP in solution phase.[2]The thermal correction to Gibbs free energy calculated by B3LYP in solution phase. [3]The thermal correction to enthalpy calculated by B3LYP in solution phase. [4]The electronic energy calculated by M06-2X in diethylether solvent. [5]The B3LYP calculated imaginary frequencies for the transition states.

**Supplementary Table S16.** $SMD_{(diethylether)}$/M06-2X/def2-TZVP//B3LYP/def2-SVP calculated absolute energies of the enantio-determining transition structures. Values are given in Hartree.

| Geometry | $\Delta E_{(elec\text{-}B3LYP)}$[1] | $\Delta G_{(corr\text{-}B3LYP)}$[2] | $\Delta H_{(corr\text{-}B3LYP)}$[3] | $\Delta E_{(solv\text{-}M06\text{-}2X)}$[4] | IF[5] |
|---|---|---|---|---|---|
| **TS-(*1S,2S,3S,4S*)** | -3642.41688 | 0.96016 | 1.13750 | -3644.633050 | -277.07 |
| **TS-(*1S,2S,3S,4S*)-conformation-2** | -3642.415465 | 0.96062 | 1.13744 | -3644.631912 | -272.65 |
| **TS-(*1S,2S,3S,4S*)-conformation-3** | -3642.415381 | 0.95976 | 1.13736 | -3644.630416 | -272.68 |
| **TS-(*1R,2R,3S,4S*)** | -3642.41230 | 0.95998 | 1.13714 | -3644.63053 | -259.80 |
| **TS-(*1S,2S,3R,4R*)** | -3642.41221 | 0.96127 | 1.13760 | -3644.62832 | -266.49 |
| **TS-(*1R,2R,3R,4R*)** | -3642.41172 | 0.96124 | 1.13748 | -3644.632637 | -268.31 |

[1]The electronic energy calculated by B3LYP in gas phase.[2]The thermal correction to Gibbs free energy calculated by B3LYP in gas phase. [3]The thermal correction to enthalpy calculated by B3LYP in gas phase. [4]The electronic energy calculated by M06-2X in diethylether solvent. [5]The B3LYP calculated imaginary frequencies for the transition states.

**Supplementary Table S17.** The calculated relative free energies for intermediate 3a and transition states TS1 by using various density functionals as well as high-level DLPNO-CCSD(T)/def2-TZVP method implemented by SMD implicit solvent model in ORCA 4.2. Values are given in kcal/mol.

| Methods / Structure | M06-2X | B3LYP | B3LYP-D3 | ωB97XD | DLPNO-CCSD(T) |
|---|---|---|---|---|---|
| **3a** | 0.0 | 0.0 | 0.0 | 0.0 | 0.0 |
| **TS1** | 11.1 | 4.8 | 4.2 | 10.8 | 13.7 |

## E). Free-energy profiles for possible TS models of the first [3 + 2] cycloaddition reaction

To gain insights into the asymmetric induction of this cascade, we carried out additional DFT studies on the stereoselectivity-determining transition states, particularly towards the first [3 + 2] annulation step. Because the large size and conformational flexibility of the optimal catalyst **P10** would result in an insurmountable computational cost, we chose a smaller catalyst **P4** as a model, which retains important hydrogen bonding and ion paring capabilities as the optimal catalyst **P10** does. Three conformations (with respect to *i*-Pr group of the catalyst) of the Michael addition TS that leads to (*S*, *S*, *S*, *S*)-**3a** (following subsequent Mannich reaction) are shown below, and mportant hydrogen bonding interactions are indicated (Supplementary Figure S15).

To compare with the experimental outcome, four stereoisomeric TSs are optimized and shown in Figure S16. Particularly, the second lowest energy transition state TS-(*1R,2R,3R,4R*) is 0.9 kcal/mol (~64% ee at r.t.) higher in energy than that of TS-(*1S,2S,3S,4S*), in good agreement with the experimentally observed stereoselectivity for catalyst **P4** (42% ee).

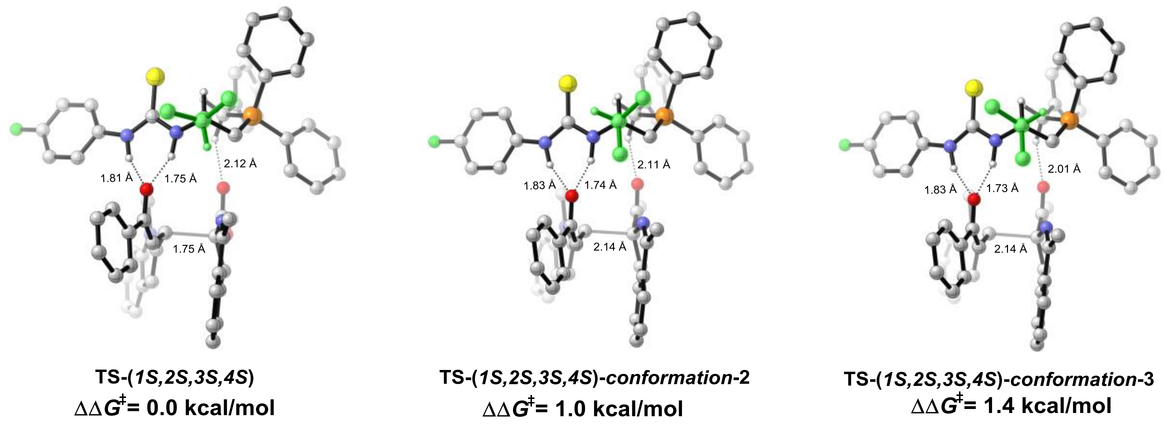


**Supplementary Figure S15**. Three conformations (with respect to the *i*-Pr group of the catalyst) of the Michael addition TS.

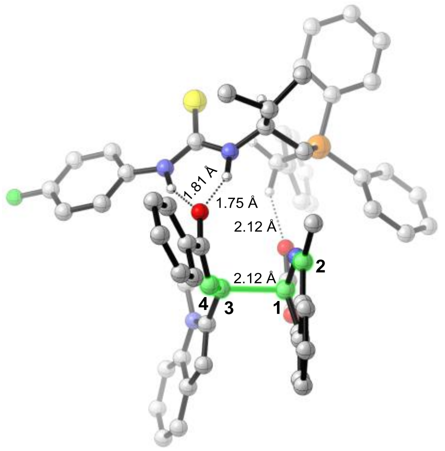


**TS-(*1S,2S,3S,4S*)**
**$\Delta\Delta G^{\ddagger}$= 0.0 kcal/mol**

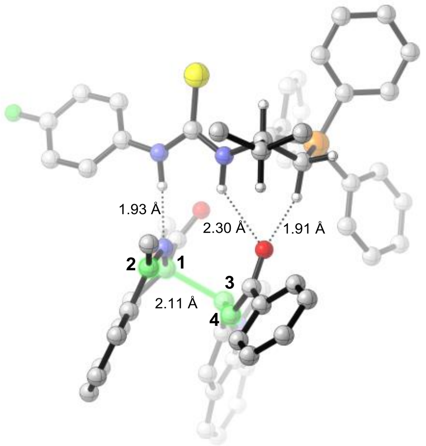


**TS-(*1R,2R,3R,4R*)**
**$\Delta\Delta G^{\ddagger}$= 0.9 kcal/mol**

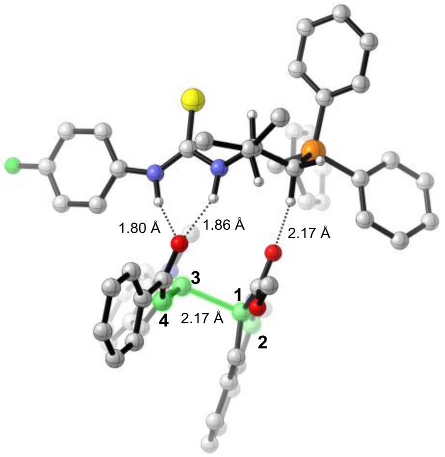


**TS-(*1R,2R,3S,4S*)**
**$\Delta\Delta G^{\ddagger}$= 1.5 kcal/mol**

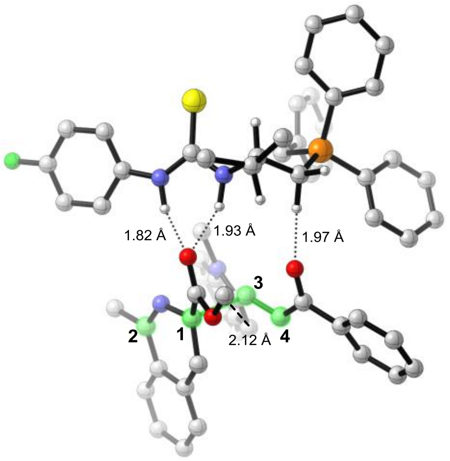


**TS-(1*S,2S,3R,4R*)**
**$\Delta\Delta G^{\ddagger}$= 3.7 kcal/mol**

Supplementary **Figure S16.** The relative energies of the enantio-determining transition structures were evaluated. The values are relative energies compared to the lowest TS-(1S,2S,3S,4S). Distances are in Å.

**F). Cartesian coordinates for DFT-optimized structures for all the optimized compounds and transition states**

# 3a

| | | | |
|---|---|---|---|
| C | 1.74253900 | -1.47493000 | -0.49666200 |
| C | 1.08166800 | -0.02790400 | -0.41101700 |
| C | -0.40242800 | -0.26612900 | -0.82412200 |
| C | -0.52668500 | -1.83668900 | -0.87737200 |
| C | -0.79326600 | -2.52404200 | 0.48517600 |
| H | 1.13413500 | 0.31922400 | 0.62450200 |
| H | -0.46348900 | 0.04896500 | -1.87272000 |
| C | 2.79453100 | -2.15698200 | 1.76949000 |
| C | 1.68078900 | -2.07993100 | 0.91794000 |
| C | 0.43333600 | -2.55599700 | 1.37796500 |
| C | 0.33695500 | -3.09721600 | 2.66899000 |
| C | 1.45059500 | -3.16652900 | 3.50881000 |
| C | 2.68554800 | -2.69539500 | 3.05509200 |
| H | 3.76692400 | -1.79516100 | 1.43059900 |
| H | -0.63091500 | -3.47020800 | 3.01802200 |
| H | 1.35433300 | -3.58926300 | 4.51247100 |
| H | 3.56736100 | -2.74805200 | 3.69940900 |
| C | 3.13893500 | -1.53322800 | -1.11722900 |
| H | 3.87754300 | -0.93221900 | -0.57043400 |
| H | 3.49896300 | -2.57441600 | -1.13701700 |
| H | 3.09711600 | -1.17226400 | -2.15449500 |
| C | 1.75106600 | 1.01815000 | -1.30959800 |
| O | 1.37820100 | 1.18333800 | -2.45981600 |
| C | 2.87344400 | 1.85487900 | -0.76102800 |
| C | 3.49459900 | 2.76884600 | -1.63458200 |
| C | 3.32356600 | 1.77909600 | 0.56985900 |
| C | 4.53545000 | 3.58269900 | -1.19274800 |
| H | 3.13964500 | 2.82372500 | -2.66527200 |
| C | 4.36769300 | 2.59605200 | 1.01263500 |
| H | 2.86521100 | 1.08549600 | 1.27585600 |
| C | 4.97501900 | 3.49825200 | 0.13452200 |
| H | 5.00747700 | 4.28729600 | -1.88245600 |
| H | 4.70559000 | 2.52831300 | 2.04984200 |
| H | 5.79040300 | 4.13743900 | 0.48375800 |
| C | -2.62818900 | 1.50098000 | 1.62135700 |
| C | -3.13633500 | 1.91117300 | 0.35141300 |
| C | -4.22592400 | 2.79220100 | 0.24772900 |
| C | -4.80466700 | 3.25668300 | 1.42707600 |

| | | | |
|---|---|---|---|
| C | -4.31535800 | 2.85875200 | 2.69178500 |
| C | -3.23523300 | 1.98802300 | 2.79712400 |
| C | -1.52884600 | 0.62333700 | 1.35458800 |
| C | -1.40115100 | 0.51459400 | -0.01841300 |
| H | -4.61582600 | 3.10929600 | -0.72184000 |
| H | -5.65463200 | 3.94230200 | 1.37110000 |
| H | -4.79426900 | 3.24281600 | 3.59666300 |
| H | -2.85920700 | 1.68342800 | 3.77805500 |
| H | -0.89962600 | 0.13046300 | 2.09307800 |
| N | -2.37425000 | 1.30243100 | -0.62803800 |
| C | -2.53853300 | 1.49908800 | -2.05616100 |
| H | -2.79280200 | 0.55722800 | -2.56457300 |
| H | -3.35362700 | 2.21157900 | -2.23164100 |
| H | -1.62416700 | 1.91082500 | -2.51074000 |
| N | 0.79782300 | -2.15769600 | -1.38708800 |
| H | 0.96746400 | -3.16198800 | -1.44375500 |
| C | -1.58811000 | -2.30871900 | -1.86896400 |
| O | -2.81147200 | -1.88228800 | -1.51468500 |
| C | -3.90925200 | -2.31926100 | -2.32392100 |
| H | -4.80894100 | -1.86773900 | -1.88740500 |
| H | -3.78719500 | -1.98866100 | -3.36637500 |
| H | -3.99253500 | -3.41671700 | -2.30760800 |
| O | -1.37982300 | -3.01399900 | -2.82604100 |
| H | -1.10815400 | -3.56627300 | 0.29336500 |
| H | -1.63757900 | -2.04410200 | 1.00141100 |

# $BF_3^{\cdot}$ $Et_2O$

| | | | |
|---|---|---|---|
| B | -1.25355300 | -0.09966300 | 0.10318600 |
| F | -1.20801600 | -0.65514700 | 1.35642900 |
| F | -1.89961100 | -0.89761900 | -0.80783900 |
| F | -1.67589000 | 1.20313200 | 0.08722800 |
| C | 2.05922400 | -1.52405800 | 0.46973200 |
| H | 1.61658200 | -1.51810900 | 1.47712100 |
| H | 2.51252400 | -2.51375200 | 0.29754500 |
| H | 2.86533000 | -0.77555500 | 0.42715800 |
| C | 1.00019700 | -1.28863300 | -0.58451400 |
| H | 1.42099100 | -1.26285700 | -1.60163900 |
| H | 0.22390800 | -2.06311300 | -0.56084200 |
| C | 0.92925900 | 1.22163400 | -0.76309200 |
| H | 0.20408900 | 1.76978000 | -1.37846200 |
| H | 1.78335800 | 0.94549800 | -1.39759800 |
| C | 1.35510100 | 2.02573300 | 0.44600800 |

| | | | |
|---|---|---|---|
| H | 2.07681800 | 1.47852500 | 1.07045600 |
| H | 1.83538900 | 2.95571000 | 0.10011900 |
| H | 0.48638800 | 2.30013500 | 1.06153600 |
| O | 0.27891900 | -0.03491200 | -0.39281100 |

## $Et_2O$

| | | | |
|---|---|---|---|
| C | -2.10095500 | 0.59968700 | -0.16122300 |
| H | -2.11019900 | 0.76712200 | -1.25087100 |
| H | -3.14060100 | 0.44227300 | 0.17092000 |
| H | -1.73411400 | 1.51824300 | 0.32438800 |
| C | -1.25136200 | -0.61839100 | 0.18622900 |
| H | -1.24151700 | -0.77320700 | 1.28566300 |
| H | -1.70251400 | -1.52041500 | -0.26059100 |
| C | 0.95107600 | 0.28762500 | 0.39296700 |
| H | 0.94974700 | 0.02891800 | 1.47349400 |
| H | 0.62946200 | 1.34634100 | 0.32150500 |
| C | 2.34633200 | 0.13441500 | -0.18217900 |
| H | 2.35959000 | 0.40769400 | -1.24973600 |
| H | 3.05744900 | 0.78614000 | 0.35043800 |
| H | 2.69358700 | -0.90755500 | -0.09175100 |
| O | 0.07107100 | -0.56444700 | -0.31102900 |

## D

| | | | |
|---|---|---|---|
| C | 1.16402300 | 1.78224800 | 0.93295400 |
| C | 0.81380300 | 0.48905200 | -0.02886100 |
| C | -0.49566900 | -0.06429700 | 0.60034200 |
| C | -0.88150200 | 1.02755400 | 1.71164100 |
| C | -1.70067700 | 2.21577900 | 1.18365900 |
| H | 0.66087000 | 0.86006400 | -1.04356700 |
| H | -0.19876400 | -0.94167000 | 1.18699900 |
| C | 1.26946100 | 3.87227100 | -0.56777800 |
| C | 0.52605300 | 2.97874300 | 0.21720700 |
| C | -0.86893400 | 3.15187400 | 0.33019100 |
| C | -1.48173000 | 4.22280800 | -0.33696100 |
| C | -0.73500000 | 5.11038300 | -1.11453300 |
| C | 0.64656300 | 4.93366300 | -1.23012600 |
| H | 2.34902800 | 3.74701600 | -0.66816600 |
| H | -2.56327000 | 4.35917600 | -0.24291600 |
| H | -1.23095000 | 5.93810300 | -1.62842300 |
| H | 1.24312400 | 5.62277900 | -1.83366500 |
| C | 2.64044200 | 1.97710700 | 1.26114000 |

| | | | |
|---|---|---|---|
| H | 3.26203100 | 2.06502300 | 0.36050500 |
| H | 2.76665800 | 2.89086000 | 1.86049100 |
| H | 3.02089900 | 1.13124200 | 1.85321200 |
| C | 1.93208400 | -0.49951300 | -0.02634300 |
| O | 2.02284600 | -1.21902800 | 1.00504200 |
| C | 2.90728500 | -0.54760300 | -1.12334900 |
| C | 4.24618700 | -0.92244900 | -0.86744600 |
| C | 2.53124300 | -0.19510100 | -2.43918200 |
| C | 5.17842500 | -0.93413900 | -1.90050200 |
| H | 4.55654800 | -1.17474200 | 0.14592000 |
| C | 3.46494400 | -0.23934300 | -3.47226200 |
| H | 1.49937600 | 0.07648900 | -2.66456700 |
| C | 4.78958900 | -0.60146400 | -3.20391300 |
| H | 6.21484600 | -1.20777000 | -1.68980500 |
| H | 3.15866300 | 0.01065200 | -4.49057600 |
| H | 5.52321700 | -0.62372200 | -4.01405500 |
| C | -3.00977600 | -0.59434100 | -2.12417400 |
| C | -3.16891000 | -1.74807000 | -1.30149200 |
| C | -4.11120800 | -2.74645900 | -1.59680700 |
| C | -4.89701000 | -2.57762400 | -2.73526200 |
| C | -4.75294300 | -1.44196400 | -3.56410000 |
| C | -3.81942800 | -0.45234400 | -3.26961400 |
| C | -1.97597900 | 0.19112600 | -1.51998800 |
| C | -1.54698400 | -0.47645600 | -0.38585800 |
| H | -4.22972800 | -3.62592500 | -0.96046100 |
| H | -5.63945100 | -3.33834600 | -2.99128400 |
| H | -5.38703900 | -1.34278100 | -4.44932700 |
| H | -3.71488800 | 0.42383500 | -3.91576200 |
| H | -1.59452400 | 1.14630800 | -1.87554900 |
| N | -2.27026700 | -1.66263400 | -0.25682700 |
| C | -2.16637200 | -2.68148000 | 0.77071400 |
| H | -3.07719500 | -2.71296800 | 1.39081600 |
| H | -2.02519900 | -3.67206100 | 0.31032000 |
| H | -1.30631400 | -2.49642600 | 1.42403300 |
| N | 0.40995700 | 1.53004100 | 2.14447600 |
| C | -1.56667200 | 0.32709500 | 2.88250400 |
| O | -2.87702600 | 0.16952300 | 2.67750600 |
| C | -3.61984300 | -0.50435200 | 3.70340700 |
| H | -4.64641700 | -0.59221700 | 3.32700100 |
| H | -3.19885100 | -1.50049500 | 3.90311800 |
| H | -3.60868500 | 0.08137400 | 4.63495200 |
| O | -0.98095500 | -0.07193100 | 3.86181800 |
| H | -2.08429400 | 2.77916500 | 2.05147900 |

| | | | |
|---|---|---|---|
| H | -2.57919000 | 1.86294700 | 0.62594000 |
| B | 2.74112300 | -2.58049100 | 1.41759600 |
| F | 2.93559600 | -3.31104000 | 0.27274200 |
| F | 1.82900500 | -3.15207500 | 2.26700300 |
| F | 3.91161600 | -2.21809200 | 2.04627200 |
| H | 0.87239800 | 0.85081500 | 2.75148500 |

# TS1

| | | | |
|---|---|---|---|
| C | -1.37078000 | 1.53045800 | -1.35837900 |
| C | -0.72892900 | 0.24836200 | 0.21929900 |
| C | 0.53670800 | -0.21414900 | -0.48334900 |
| C | 0.76971500 | 0.68449300 | -1.81531200 |
| C | 1.46879800 | 2.03050700 | -1.57858100 |
| H | -0.61495200 | 0.93410100 | 1.05545500 |
| H | 0.30250300 | -1.20482700 | -0.89015700 |
| C | -1.63035900 | 3.71536900 | -0.10007500 |
| C | -0.81230500 | 2.77846000 | -0.75445300 |
| C | 0.57514800 | 3.02028500 | -0.86368500 |
| C | 1.10673800 | 4.19792400 | -0.32517000 |
| C | 0.28720200 | 5.12668600 | 0.32212700 |
| C | -1.08488500 | 4.88323300 | 0.43499100 |
| H | -2.70281200 | 3.53944100 | -0.00613000 |
| H | 2.18043300 | 4.38676800 | -0.41291200 |
| H | 0.72086900 | 6.03901400 | 0.73960800 |
| H | -1.73389800 | 5.60409000 | 0.93824800 |
| C | -2.84755700 | 1.43619000 | -1.62488600 |
| H | -3.44452200 | 1.64290900 | -0.72901400 |
| H | -3.12242900 | 2.17663400 | -2.39501400 |
| H | -3.11191400 | 0.43641500 | -1.99676200 |
| C | -1.83635900 | -0.63171700 | 0.28151100 |
| O | -1.92855400 | -1.51569400 | -0.65540000 |
| C | -2.88749400 | -0.46045900 | 1.31317200 |
| C | -4.24018000 | -0.73359800 | 1.02284900 |
| C | -2.54833000 | 0.00369900 | 2.60111500 |
| C | -5.22280200 | -0.53035700 | 1.99099000 |
| H | -4.52060100 | -1.09769200 | 0.03504000 |
| C | -3.53418700 | 0.18733500 | 3.57095000 |
| H | -1.50377700 | 0.18936100 | 2.85766700 |
| C | -4.87442000 | -0.07269700 | 3.26661500 |
| H | -6.26839100 | -0.73711800 | 1.74938000 |
| H | -3.25351600 | 0.52982000 | 4.57009200 |
| H | -5.64720500 | 0.07533500 | 4.02558300 |

| | | | |
|---|---|---|---|
| C | 3.32937300 | 0.01460100 | 2.00563800 |
| C | 3.50475100 | -1.28016600 | 1.43450400 |
| C | 4.54374600 | -2.13490700 | 1.83670900 |
| C | 5.41239300 | -1.67621600 | 2.82531500 |
| C | 5.25441500 | -0.39661900 | 3.40418100 |
| C | 4.22391500 | 0.44960100 | 3.00478900 |
| C | 2.18769900 | 0.58810000 | 1.35915000 |
| C | 1.71520200 | -0.33443200 | 0.44209400 |
| H | 4.67228900 | -3.12491900 | 1.39406100 |
| H | 6.23140300 | -2.31902000 | 3.15923400 |
| H | 5.95459700 | -0.06992300 | 4.17784500 |
| H | 4.10839300 | 1.43813700 | 3.45824600 |
| H | 1.75844400 | 1.56986800 | 1.54855100 |
| N | 2.51404400 | -1.47633300 | 0.49273800 |
| C | 2.39229500 | -2.70546300 | -0.26824900 |
| H | 3.25614900 | -2.84210500 | -0.93937200 |
| H | 2.34052800 | -3.57067700 | 0.41141600 |
| H | 1.47810700 | -2.70582400 | -0.87276500 |
| N | -0.57244400 | 0.93076600 | -2.28979300 |
| C | 1.49038500 | -0.16938100 | -2.85818300 |
| O | 2.80786800 | -0.18225800 | -2.66823700 |
| C | 3.58991400 | -0.98258800 | -3.56931700 |
| H | 4.62246500 | -0.92769600 | -3.20409100 |
| H | 3.23787700 | -2.02397500 | -3.57210200 |
| H | 3.52768900 | -0.57973000 | -4.59131500 |
| O | 0.91635700 | -0.78704900 | -3.72445100 |
| H | 1.75590800 | 2.45165300 | -2.55848100 |
| H | 2.40072400 | 1.87621700 | -1.01942100 |
| B | -2.71500400 | -2.81717300 | -0.85947200 |
| F | -3.00457600 | -3.38061800 | 0.36889900 |
| F | -1.86646000 | -3.61653000 | -1.60337100 |
| F | -3.86742500 | -2.50113600 | -1.57516000 |
| H | -0.97862700 | 0.14780100 | -2.80289500 |

# E

| | | | |
|---|---|---|---|
| C | 2.21813700 | -1.85421000 | -1.76714100 |
| C | -1.36714100 | -0.02164900 | -0.76751700 |
| C | -0.13742200 | -0.27772500 | 0.07334000 |
| C | 0.39014800 | -1.76691800 | -0.09948900 |
| C | 1.42517200 | -2.18866600 | 0.95956800 |
| H | -1.36111500 | -0.29594300 | -1.82335300 |
| H | -0.47094500 | -0.24507200 | 1.11677600 |

| | | | |
|---|---|---|---|
| C | 4.56196700 | -1.34392000 | -1.06151800 |
| C | 3.22400000 | -1.64175400 | -0.73247300 |
| C | 2.83781100 | -1.77899800 | 0.62282300 |
| C | 3.80337900 | -1.61793300 | 1.61837800 |
| C | 5.12776100 | -1.31569300 | 1.28426100 |
| C | 5.50884500 | -1.17741100 | -0.05524400 |
| H | 4.86083600 | -1.22665300 | -2.10411700 |
| H | 3.51865700 | -1.73417300 | 2.66707600 |
| H | 5.86814000 | -1.18608800 | 2.07770900 |
| H | 6.54241200 | -0.93655100 | -0.31238400 |
| C | 2.57430700 | -1.99407900 | -3.20971800 |
| H | 3.03870100 | -1.06178700 | -3.56824700 |
| H | 3.31285100 | -2.79931000 | -3.34383200 |
| H | 1.68775100 | -2.20585600 | -3.82298300 |
| C | -2.45778200 | 0.57954900 | -0.22659400 |
| O | -2.38903900 | 1.08474500 | 1.01251300 |
| C | -3.72411100 | 0.80787000 | -0.97954300 |
| C | -4.63925400 | 1.76917700 | -0.51227600 |
| C | -4.04436500 | 0.09631900 | -2.15272100 |
| C | -5.82340800 | 2.02703800 | -1.20768100 |
| H | -4.41350800 | 2.30468100 | 0.41062300 |
| C | -5.22653700 | 0.35599000 | -2.84724200 |
| H | -3.37507900 | -0.68813900 | -2.51261400 |
| C | -6.12093600 | 1.32712900 | -2.38072500 |
| H | -6.51966500 | 2.77960000 | -0.82716900 |
| H | -5.45836300 | -0.21224700 | -3.75254400 |
| H | -7.04905100 | 1.52709300 | -2.92320600 |
| C | 2.42491700 | 2.25067300 | -0.97741800 |
| C | 2.48219700 | 2.30009300 | 0.44771400 |
| C | 3.36223100 | 3.16025500 | 1.12321800 |
| C | 4.19071300 | 3.97567300 | 0.35413400 |
| C | 4.14768000 | 3.94163200 | -1.05777200 |
| C | 3.27427900 | 3.08963200 | -1.72767300 |
| C | 1.42703900 | 1.27812600 | -1.30639000 |
| C | 0.92547400 | 0.77392200 | -0.11859700 |
| H | 3.40361700 | 3.19183900 | 2.21395500 |
| H | 4.88657800 | 4.65502900 | 0.85384000 |
| H | 4.80981300 | 4.59799300 | -1.62908000 |
| H | 3.24283600 | 3.07365200 | -2.82094400 |
| H | 1.09003900 | 1.00061600 | -2.30315700 |
| N | 1.55776900 | 1.40247600 | 0.94920500 |
| C | 1.26421800 | 1.25434400 | 2.36440100 |
| H | 0.23532100 | 0.90483600 | 2.51700300 |

| | | | |
|---|---|---|---|
| H | 1.96314600 | 0.55791300 | 2.85759300 |
| H | 1.34951600 | 2.23382000 | 2.85628000 |
| N | 0.96087600 | -1.96622700 | -1.43516600 |
| C | -0.85003600 | -2.68201500 | -0.05204400 |
| O | -1.33486200 | -2.76142200 | 1.17162800 |
| C | -2.57085300 | -3.47748500 | 1.35733900 |
| H | -2.79792500 | -3.40531400 | 2.42631800 |
| H | -3.36955900 | -3.00340400 | 0.77300200 |
| H | -2.44981300 | -4.52785300 | 1.05462800 |
| O | -1.29860500 | -3.23989200 | -1.02911600 |
| H | 1.41026900 | -3.28918100 | 1.05747300 |
| H | 1.12323000 | -1.79141800 | 1.93710300 |
| H | 0.27720300 | -2.20888700 | -2.15710300 |
| B | -3.03428000 | 0.52667100 | 2.21382600 |
| F | -3.85266400 | 1.49952500 | 2.79989200 |
| F | -3.78703700 | -0.61345300 | 1.89074800 |
| F | -2.01424900 | 0.17420200 | 3.12951300 |

# TS2

| | | | |
|---|---|---|---|
| C | 2.24768800 | -0.71921200 | -1.78077700 |
| C | -1.43547400 | 0.17296200 | -0.69170000 |
| C | -0.19768900 | -0.36653200 | -0.00705400 |
| C | 0.33671500 | -1.72741000 | -0.68432100 |
| C | 1.22989500 | -2.52833200 | 0.28083000 |
| H | -1.46309200 | 0.19116900 | -1.78145700 |
| H | -0.46759300 | -0.62139600 | 1.02164600 |
| C | 4.47774800 | -0.69396400 | -0.57929600 |
| C | 3.14507100 | -1.13398600 | -0.65226700 |
| C | 2.64212900 | -1.98994400 | 0.35252700 |
| C | 3.48645400 | -2.38415800 | 1.39924500 |
| C | 4.81052400 | -1.94388600 | 1.46190100 |
| C | 5.30802400 | -1.09715100 | 0.46741000 |
| H | 4.87656800 | -0.02840300 | -1.34528000 |
| H | 3.09625100 | -3.05127900 | 2.17312900 |
| H | 5.45271800 | -2.26272000 | 2.28697200 |
| H | 6.34211900 | -0.74645500 | 0.50589800 |
| C | 2.88685100 | -0.40333700 | -3.11413700 |
| H | 3.61765300 | 0.41090900 | -3.03881400 |
| H | 3.41087000 | -1.29999100 | -3.48461000 |
| H | 2.12318100 | -0.11694700 | -3.85226100 |
| C | -2.48010800 | 0.64679100 | 0.03382100 |
| O | -2.37768200 | 0.78012800 | 1.36440400 |

| | | | |
|---|---|---|---|
| C | -3.74971400 | 1.13151400 | -0.57988700 |
| C | -4.63771400 | 1.89439200 | 0.20071600 |
| C | -4.10304300 | 0.85648600 | -1.91613700 |
| C | -5.82905600 | 2.38358500 | -0.34121700 |
| H | -4.38225800 | 2.09079100 | 1.24278500 |
| C | -5.29260000 | 1.34637500 | -2.45623400 |
| H | -3.45533000 | 0.23272500 | -2.53581900 |
| C | -6.16069000 | 2.11676800 | -1.67258100 |
| H | -6.50386800 | 2.97494000 | 0.28391500 |
| H | -5.55035800 | 1.11590500 | -3.49373500 |
| H | -7.09436500 | 2.49654300 | -2.09632100 |
| C | 2.55302900 | 2.06369500 | -0.75500000 |
| C | 2.51703200 | 2.04651600 | 0.65701500 |
| C | 3.37662600 | 2.81686400 | 1.44175600 |
| C | 4.28494000 | 3.63999500 | 0.77020200 |
| C | 4.32351600 | 3.68979600 | -0.63500600 |
| C | 3.46200200 | 2.90637900 | -1.40792800 |
| C | 1.55013500 | 1.10746300 | -1.21172000 |
| C | 0.88621300 | 0.65320700 | -0.01931000 |
| H | 3.34475900 | 2.78504600 | 2.53193800 |
| H | 4.97269600 | 4.26079900 | 1.34976000 |
| H | 5.03675700 | 4.35512900 | -1.12795000 |
| H | 3.49331600 | 2.95775500 | -2.49902200 |
| H | 0.99810000 | 1.24861800 | -2.14008600 |
| N | 1.49140100 | 1.17001300 | 1.06390300 |
| C | 1.18818700 | 0.91287200 | 2.46671200 |
| H | 0.22740600 | 0.39584500 | 2.56628200 |
| H | 1.98660300 | 0.30751000 | 2.92296900 |
| H | 1.12129800 | 1.87372300 | 2.99648600 |
| N | 1.08883900 | -1.44503000 | -1.88061700 |
| C | -0.90809600 | -2.52778500 | -1.09255900 |
| O | -1.52694800 | -3.01780000 | -0.03056900 |
| C | -2.80073800 | -3.65097600 | -0.23006800 |
| H | -3.15356500 | -3.93556000 | 0.76716700 |
| H | -3.50643100 | -2.94542400 | -0.68960100 |
| H | -2.69247700 | -4.53867300 | -0.87081500 |
| O | -1.26968200 | -2.66113800 | -2.23966200 |
| H | 1.27487200 | -3.57308500 | -0.07417100 |
| H | 0.77089300 | -2.55891300 | 1.27842400 |
| H | 0.53488100 | -1.35463700 | -2.73014500 |
| B | -2.83118300 | -0.20358000 | 2.36454200 |
| F | -1.68414400 | -0.80524600 | 2.95214500 |
| F | -3.54560400 | 0.46465000 | 3.36115500 |

| | | | |
|---|---|---|---|
| F | -3.61803200 | -1.19766700 | 1.76936700 |

**F**

| | | | |
|---|---|---|---|
| C | 2.28617100 | -0.58633600 | -1.64778100 |
| C | -1.37153100 | 0.03708700 | -0.66567000 |
| C | -0.16356900 | -0.49709200 | 0.08397500 |
| C | 0.44334400 | -1.81215700 | -0.58920400 |
| C | 1.37393400 | -2.55849100 | 0.38469100 |
| H | -1.35904000 | -0.01799100 | -1.75409800 |
| H | -0.45827800 | -0.72748100 | 1.11232900 |
| C | 4.57107600 | -0.63662300 | -0.48819800 |
| C | 3.24091000 | -1.08394300 | -0.55277100 |
| C | 2.77055200 | -1.97515500 | 0.43460700 |
| C | 3.63997700 | -2.38250700 | 1.46044100 |
| C | 4.95722600 | -1.92876300 | 1.51659600 |
| C | 5.42675600 | -1.05400200 | 0.53245000 |
| H | 4.95155600 | 0.04932300 | -1.24575900 |
| H | 3.27029500 | -3.07589700 | 2.22162100 |
| H | 5.61670500 | -2.26018200 | 2.32301800 |
| H | 6.45856400 | -0.69439300 | 0.55791300 |
| C | 2.95762200 | -0.43337000 | -3.01427900 |
| H | 3.75742600 | 0.31773900 | -3.00858800 |
| H | 3.38596600 | -1.39799600 | -3.32415600 |
| H | 2.21359600 | -0.12640200 | -3.76676400 |
| C | -2.43194200 | 0.57318700 | -0.00681500 |
| O | -2.36517900 | 0.78777600 | 1.31325000 |
| C | -3.67755600 | 1.02179900 | -0.69141900 |
| C | -4.58131500 | 1.84376300 | 0.00701300 |
| C | -3.99243800 | 0.65509600 | -2.01534600 |
| C | -5.75024400 | 2.30320800 | -0.60492800 |
| H | -4.35827600 | 2.10948300 | 1.04115800 |
| C | -5.15972900 | 1.11571200 | -2.62491300 |
| H | -3.33525900 | -0.01852400 | -2.56895400 |
| C | -6.04268900 | 1.94681600 | -1.92440800 |
| H | -6.43828500 | 2.94165100 | -0.04396800 |
| H | -5.38885600 | 0.81415400 | -3.65074500 |
| H | -6.95884100 | 2.30350600 | -2.40283800 |
| C | 2.54583700 | 1.97443300 | -0.76221700 |
| C | 2.25992200 | 2.25342000 | 0.58297500 |
| C | 2.88770600 | 3.26359300 | 1.30488200 |
| C | 3.84072800 | 4.02885200 | 0.62158700 |
| C | 4.12906700 | 3.78493000 | -0.72794200 |
| C | 3.48155000 | 2.76044600 | -1.43280100 |

| | | | |
|---|---|---|---|
| C | 1.65985600 | 0.83735100 | -1.19021600 |
| C | 0.86087800 | 0.57164800 | 0.04702700 |
| H | 2.65074800 | 3.46007600 | 2.35126700 |
| H | 4.35879800 | 4.83323200 | 1.14909900 |
| H | 4.86729200 | 4.40621300 | -1.24075400 |
| H | 3.70741500 | 2.59171800 | -2.48766900 |
| H | 0.99561400 | 1.13113000 | -2.01869800 |
| N | 1.23881600 | 1.36127800 | 1.02918600 |
| C | 0.74641600 | 1.37986600 | 2.40492000 |
| H | -0.09445700 | 0.68962800 | 2.52386800 |
| H | 1.56881400 | 1.10531800 | 3.08174600 |
| H | 0.40649100 | 2.39761700 | 2.64259200 |
| N | 1.19714600 | -1.52980200 | -1.78789000 |
| C | -0.76608400 | -2.67921100 | -0.96657000 |
| O | -1.36410600 | -3.15583300 | 0.12022300 |
| C | -2.61369300 | -3.83963700 | -0.05170400 |
| H | -2.95204200 | -4.10668200 | 0.95598700 |
| H | -3.34862200 | -3.17628700 | -0.52896900 |
| H | -2.47936700 | -4.74361600 | -0.66436000 |
| O | -1.13814300 | -2.86692900 | -2.10131500 |
| H | 1.45443500 | -3.60429100 | 0.04107700 |
| H | 0.93209100 | -2.59538300 | 1.39050100 |
| H | 0.60360700 | -1.36246100 | -2.59766900 |
| B | -2.91721200 | -0.12149200 | 2.34395200 |
| F | -1.82632500 | -0.65661700 | 3.07580700 |
| F | -3.73014600 | 0.61078800 | 3.21063000 |
| F | -3.63539200 | -1.16302600 | 1.74709900 |

# G

| | | | |
|---|---|---|---|
| C | 2.43318700 | -0.89706900 | -1.12369100 |
| C | -1.14851600 | 0.59316900 | -0.86308600 |
| C | -0.04765400 | 0.29525300 | 0.19210600 |
| C | 0.38733400 | -1.20297000 | 0.18676700 |
| C | 1.29910600 | -1.49569100 | 1.44813200 |
| H | -0.99533500 | 1.60626400 | -1.28047100 |
| C | 4.57988700 | -2.16300500 | -0.38484500 |
| C | 3.28223900 | -1.70243600 | -0.12179600 |
| C | 2.67089300 | -2.03295500 | 1.10797300 |
| C | 3.36526600 | -2.82846400 | 2.02975600 |
| C | 4.66217300 | -3.27550200 | 1.76249500 |
| C | 5.27192500 | -2.93777500 | 0.55201800 |
| H | 5.06466100 | -1.92193600 | -1.33266700 |

| | | | |
|---|---|---|---|
| H | 2.88243200 | -3.09525800 | 2.97487300 |
| H | 5.19150000 | -3.88916000 | 2.49636800 |
| H | 6.28482800 | -3.28406200 | 0.32901300 |
| C | 2.92076600 | -1.02548800 | -2.57272600 |
| H | 3.92418200 | -0.60397900 | -2.71717500 |
| H | 2.94785400 | -2.08352600 | -2.88046100 |
| H | 2.22658400 | -0.48979800 | -3.23639000 |
| C | -2.55536100 | 0.65105500 | -0.33629400 |
| O | -2.68973700 | 1.26315600 | 0.75253300 |
| C | -3.68466100 | 0.13835900 | -1.11920800 |
| C | -4.97181400 | 0.70358500 | -0.96674100 |
| C | -3.49758500 | -0.90012900 | -2.06075800 |
| C | -6.03551900 | 0.23824100 | -1.73372100 |
| H | -5.12606500 | 1.52701300 | -0.27104600 |
| C | -4.57432700 | -1.37699700 | -2.80444500 |
| H | -2.51795700 | -1.36062700 | -2.19268400 |
| C | -5.84230400 | -0.80630800 | -2.64574200 |
| H | -7.02258500 | 0.69217200 | -1.61986500 |
| H | -4.42324400 | -2.19397900 | -3.51354000 |
| H | -6.68334100 | -1.17444300 | -3.23912200 |
| C | 3.31539700 | 1.62095900 | -0.71587100 |
| C | 2.68049900 | 2.76126600 | -0.13184200 |
| C | 3.33333200 | 3.99617500 | -0.00197500 |
| C | 4.64763800 | 4.08758800 | -0.45684500 |
| C | 5.29943000 | 2.97260500 | -1.02536400 |
| C | 4.64904800 | 1.74764200 | -1.15687600 |
| C | 2.34230200 | 0.55661000 | -0.68329400 |
| C | 1.21078800 | 1.07038800 | -0.08076500 |
| H | 2.83274600 | 4.85920300 | 0.44247800 |
| H | 5.18219300 | 5.03720600 | -0.36812300 |
| H | 6.33347700 | 3.07262700 | -1.36613200 |
| H | 5.17673600 | 0.89580700 | -1.59032300 |
| N | 1.40281400 | 2.40291000 | 0.25137000 |
| C | 0.46995800 | 3.29079700 | 0.91792400 |
| H | 0.92710200 | 3.72419600 | 1.82198200 |
| H | 0.16152300 | 4.11671400 | 0.25509400 |
| H | -0.42735700 | 2.73982700 | 1.22388300 |
| N | 1.05788700 | -1.43962900 | -1.08339400 |
| H | 1.07162700 | -2.43980500 | -1.28292400 |
| C | -0.79026000 | -2.17765300 | 0.27174900 |
| O | -1.72408400 | -1.76168600 | 1.13428400 |
| C | -2.81287500 | -2.65799200 | 1.41436800 |
| H | -3.48776400 | -2.11053100 | 2.08059500 |

| | | | |
|---|---|---|---|
| H | -3.33596500 | -2.93754800 | 0.48880900 |
| H | -2.43651300 | -3.56836900 | 1.90461200 |
| O | -0.84448600 | -3.22787500 | -0.32499800 |
| H | 0.79849300 | -2.18726800 | 2.14295200 |
| H | 1.41979800 | -0.55429600 | 2.00913600 |
| H | -1.04199700 | -0.08547300 | -1.71718400 |
| H | -0.43617000 | 0.55058800 | 1.18793600 |
| B | -3.76545400 | 1.37232100 | 1.92638900 |
| F | -4.44745300 | 0.17908400 | 1.96346900 |
| F | -4.56780700 | 2.45011200 | 1.62530600 |
| F | -2.98300500 | 1.58253300 | 3.02940700 |

# 4a

| | | | |
|---|---|---|---|
| C | -1.86175800 | -0.65441000 | 1.21412800 |
| C | 1.68940900 | 0.74895500 | 0.29707900 |
| C | 0.46941100 | 0.26730800 | -0.53035200 |
| C | 0.06554100 | -1.20177500 | -0.19217000 |
| C | -0.94553400 | -1.74828800 | -1.28022400 |
| H | 1.49229000 | 1.74987500 | 0.72185000 |
| C | -4.04067200 | -2.04388600 | 0.93310600 |
| C | -2.77958600 | -1.64308100 | 0.47004700 |
| C | -2.27157400 | -2.21056400 | -0.71955600 |
| C | -3.02870800 | -3.17442500 | -1.39971300 |
| C | -4.28888400 | -3.56134200 | -0.93512800 |
| C | -4.79730600 | -2.99019200 | 0.23379100 |
| H | -4.44512700 | -1.61959100 | 1.85388200 |
| H | -2.62514900 | -3.62355100 | -2.31254400 |
| H | -4.86812800 | -4.30974600 | -1.48270800 |
| H | -5.78025800 | -3.28648700 | 0.61030900 |
| C | -2.21860900 | -0.49931900 | 2.69836100 |
| H | -3.21213600 | -0.05609900 | 2.84707800 |
| H | -2.20147600 | -1.47733900 | 3.20655800 |
| H | -1.47686600 | 0.15384000 | 3.18033800 |
| C | 2.96826100 | 0.92108000 | -0.51210300 |
| O | 2.91399000 | 1.25677400 | -1.68518800 |
| C | 4.30224500 | 0.76091100 | 0.16123700 |
| C | 5.45718300 | 1.04371800 | -0.59406900 |
| C | 4.45050800 | 0.35292400 | 1.49940000 |
| C | 6.72478800 | 0.92129000 | -0.02909700 |
| H | 5.33343200 | 1.36192000 | -1.63088100 |
| C | 5.72256500 | 0.23097100 | 2.06565000 |
| H | 3.57648300 | 0.12461100 | 2.11077700 |

| | | | |
|---|---|---|---|
| C | 6.86030200 | 0.51391500 | 1.30444100 |
| H | 7.61277400 | 1.14448800 | -0.62637500 |
| H | 5.82362800 | -0.08775400 | 3.10620200 |
| H | 7.85432000 | 0.41713000 | 1.74937000 |
| C | -2.82116800 | 1.74160400 | 0.42492000 |
| C | -2.25388200 | 2.75870100 | -0.40530100 |
| C | -2.93355900 | 3.94988600 | -0.69904400 |
| C | -4.20666500 | 4.12343200 | -0.15758600 |
| C | -4.79132100 | 3.13003100 | 0.65603800 |
| C | -4.11400100 | 1.94784600 | 0.94916600 |
| C | -1.83891200 | 0.68794800 | 0.49847300 |
| C | -0.76911400 | 1.08282400 | -0.28122000 |
| H | -2.48509400 | 4.71698300 | -1.33435100 |
| H | -4.76102600 | 5.04151100 | -0.37060200 |
| H | -5.79433400 | 3.29046700 | 1.06073400 |
| H | -4.58988900 | 1.18872400 | 1.57327200 |
| N | -1.00714600 | 2.33694400 | -0.82301300 |
| C | -0.14087900 | 3.09388300 | -1.70548700 |
| H | -0.62759400 | 3.26812400 | -2.67979300 |
| H | 0.10833000 | 4.07297600 | -1.26371700 |
| H | 0.79415500 | 2.54696800 | -1.87882200 |
| N | -0.48505700 | -1.18709900 | 1.15545300 |
| H | -0.45322600 | -2.12546700 | 1.55335000 |
| C | 1.26678300 | -2.15086300 | -0.18158300 |
| O | 2.06787100 | -1.95382100 | -1.23422000 |
| C | 3.18722100 | -2.83782700 | -1.37942700 |
| H | 3.73779900 | -2.48717900 | -2.26094500 |
| H | 3.83317600 | -2.80176100 | -0.49013400 |
| H | 2.84538500 | -3.87269000 | -1.53302800 |
| O | 1.44947800 | -3.01103700 | 0.64844500 |
| H | -0.49269700 | -2.56420000 | -1.86412700 |
| H | -1.13589300 | -0.94046700 | -2.00599100 |
| H | 1.82004100 | 0.10029300 | 1.17316500 |
| H | 0.73927100 | 0.32265400 | -1.59489900 |

## $Et_2O^+H$

| | | | |
|---|---|---|---|
| C | -2.12270900 | 0.63279600 | -0.18557700 |
| H | -2.15128900 | 0.72734900 | -1.28299600 |
| H | -3.16213700 | 0.52833900 | 0.16517200 |
| H | -1.71340000 | 1.55416300 | 0.25462000 |
| C | -1.37303900 | -0.59731300 | 0.24859400 |
| H | -1.28085400 | -0.70546000 | 1.33625500 |

| | | | |
|---|---|---|---|
| H | -1.77030000 | -1.52730700 | -0.17743900 |
| C | 1.04115500 | 0.33782400 | 0.43782100 |
| H | 1.00055200 | 0.04041200 | 1.49320000 |
| H | 0.65327800 | 1.35821100 | 0.31350800 |
| C | 2.39047600 | 0.12617100 | -0.18926800 |
| H | 2.39403700 | 0.39474900 | -1.25829400 |
| H | 3.10554800 | 0.79208500 | 0.31912000 |
| H | 2.73793900 | -0.91056200 | -0.06694200 |
| O | 0.05760400 | -0.59080900 | -0.22081500 |
| H | 0.11050100 | -0.52238600 | -1.19910800 |

# D-1

| | | | |
|---|---|---|---|
| C | -1.34266000 | 0.34948100 | 1.63114400 |
| C | -1.22534400 | -0.15373100 | 0.06930100 |
| C | 0.02195400 | 0.61678900 | -0.43908700 |
| C | 0.18998400 | 1.80203100 | 0.67842000 |
| C | 1.36496300 | 1.53593100 | 1.61680000 |
| H | -1.08856800 | -1.23472300 | 0.03850900 |
| H | -0.23077700 | 1.10773000 | -1.38720500 |
| C | -0.41609000 | -1.49965600 | 3.14890000 |
| C | -0.20746100 | -0.31762900 | 2.42324700 |
| C | 1.06734600 | 0.29389200 | 2.44390200 |
| C | 2.08188500 | -0.27629900 | 3.22447500 |
| C | 1.86136000 | -1.44443900 | 3.95570700 |
| C | 0.60966000 | -2.06457900 | 3.91037400 |
| H | -1.39263100 | -1.98724300 | 3.13486900 |
| H | 3.06641300 | 0.19944700 | 3.24534300 |
| H | 2.66829700 | -1.87396200 | 4.55480400 |
| H | 0.42617000 | -2.98242600 | 4.47449600 |
| C | -2.71810900 | 0.15437900 | 2.25765700 |
| H | -3.04715100 | -0.89217500 | 2.21137800 |
| H | -2.69414600 | 0.46567100 | 3.31182300 |
| H | -3.47618800 | 0.76442200 | 1.74161900 |
| C | -2.45181200 | 0.22080700 | -0.67813500 |
| O | -2.65207500 | 1.44870200 | -1.03642300 |
| C | -3.50956600 | -0.71181900 | -1.01091100 |
| C | -4.66904900 | -0.23910500 | -1.67945300 |
| C | -3.41895600 | -2.08814300 | -0.68353200 |
| C | -5.69837400 | -1.11334800 | -2.00073600 |
| H | -4.74816800 | 0.81648800 | -1.93981900 |
| C | -4.45717500 | -2.95436200 | -1.00780600 |
| H | -2.53849100 | -2.48970900 | -0.18180300 |

| | | | |
|---|---|---|---|
| C | -5.59544600 | -2.47087900 | -1.66499300 |
| H | -6.58696200 | -0.74248000 | -2.51607700 |
| H | -4.37920400 | -4.01345400 | -0.75352500 |
| H | -6.40680100 | -3.15687700 | -1.92095200 |
| C | 2.78831000 | -1.88534700 | -0.83971300 |
| C | 3.16467700 | -0.83016400 | -1.71988500 |
| C | 4.35109100 | -0.88400000 | -2.47137300 |
| C | 5.15009000 | -2.01677500 | -2.33737100 |
| C | 4.78854400 | -3.07600500 | -1.47360500 |
| C | 3.61850700 | -3.01945500 | -0.72449800 |
| C | 1.55487900 | -1.48866900 | -0.23276500 |
| C | 1.21618500 | -0.24750300 | -0.73695200 |
| H | 4.65017100 | -0.06819000 | -3.13282000 |
| H | 6.07958000 | -2.08488800 | -2.90884800 |
| H | 5.44371100 | -3.94738900 | -1.39338600 |
| H | 3.34560500 | -3.83791600 | -0.05283000 |
| H | 0.99487000 | -2.05148300 | 0.50937300 |
| N | 2.19035500 | 0.14961300 | -1.65845000 |
| C | 2.23599200 | 1.37176100 | -2.43842800 |
| H | 2.76247300 | 1.18059100 | -3.38319300 |
| H | 1.22339500 | 1.70762100 | -2.70096600 |
| H | 2.75798100 | 2.18662000 | -1.90865500 |
| N | -1.00946900 | 1.74502600 | 1.51040100 |
| H | -1.78369100 | 2.31768000 | 1.18191900 |
| C | 0.13466700 | 3.12523400 | -0.06601400 |
| O | 1.18841400 | 3.89253000 | 0.01676000 |
| C | 1.17336400 | 5.15364900 | -0.69275300 |
| H | 0.33266100 | 5.77019800 | -0.34641700 |
| H | 2.12865800 | 5.63580700 | -0.45762600 |
| H | 1.08515300 | 4.97482800 | -1.77344400 |
| O | -0.87613100 | 3.40510400 | -0.71097100 |
| H | 1.49780400 | 2.40791700 | 2.27714200 |
| H | 2.30583600 | 1.40007500 | 1.06414000 |
| H | -1.92436300 | 2.14239000 | -0.87795600 |

# TS3

| | | | |
|---|---|---|---|
| C | -1.49987700 | 0.45384100 | 1.64081900 |
| C | -1.17497900 | -0.17086700 | -0.23888300 |
| C | 0.10452300 | 0.62248700 | -0.48128300 |
| C | 0.23002200 | 1.73605300 | 0.73292200 |
| C | 1.28111100 | 1.34702800 | 1.76992600 |
| H | -1.07322300 | -1.24879500 | -0.14295000 |

| | | | |
|---|---|---|---|
| H | -0.04125500 | 1.21450800 | -1.39406100 |
| C | -0.86129600 | -1.58288100 | 3.00896700 |
| C | -0.49138800 | -0.38229000 | 2.37791000 |
| C | 0.83945900 | 0.08540600 | 2.48795600 |
| C | 1.75064000 | -0.63987700 | 3.26452700 |
| C | 1.37174000 | -1.82364200 | 3.90023000 |
| C | 0.06414600 | -2.30238300 | 3.76420100 |
| H | -1.88257100 | -1.95705500 | 2.92028100 |
| H | 2.77777900 | -0.27684300 | 3.35685200 |
| H | 2.10083900 | -2.37843400 | 4.49607600 |
| H | -0.23750600 | -3.23103500 | 4.25439900 |
| C | -2.94598000 | 0.33196900 | 2.04791600 |
| H | -3.32927200 | -0.68558200 | 1.90067700 |
| H | -3.04168300 | 0.57835400 | 3.11769900 |
| H | -3.58149700 | 1.02425500 | 1.47714400 |
| C | -2.34464700 | 0.26170200 | -0.91398900 |
| O | -2.50230000 | 1.52419200 | -1.25796600 |
| C | -3.49904600 | -0.59061800 | -1.20217800 |
| C | -4.70965800 | -0.00226500 | -1.63573400 |
| C | -3.43081700 | -1.99643700 | -1.07169300 |
| C | -5.81810100 | -0.79611600 | -1.91525600 |
| H | -4.77418000 | 1.08054500 | -1.74520700 |
| C | -4.54431400 | -2.78334400 | -1.35538300 |
| H | -2.50312100 | -2.48540500 | -0.77162600 |
| C | -5.73947400 | -2.18691600 | -1.77486200 |
| H | -6.74907100 | -0.32976800 | -2.24571900 |
| H | -4.47807000 | -3.86927300 | -1.25855100 |
| H | -6.61001600 | -2.80841800 | -1.99935200 |
| C | 2.85493600 | -1.90715200 | -0.80359700 |
| C | 3.32245700 | -0.81067700 | -1.58370200 |
| C | 4.56096900 | -0.84824100 | -2.24616300 |
| C | 5.32286500 | -2.00774800 | -2.12413600 |
| C | 4.87276300 | -3.10761200 | -1.35847300 |
| C | 3.64936100 | -3.06739500 | -0.69822100 |
| C | 1.58220100 | -1.51761100 | -0.27711100 |
| C | 1.31059400 | -0.24173700 | -0.73250400 |
| H | 4.92258500 | -0.00216900 | -2.83418200 |
| H | 6.29051800 | -2.06685100 | -2.62939000 |
| H | 5.49959600 | -4.00027800 | -1.28624400 |
| H | 3.30724500 | -3.92006000 | -0.10534000 |
| H | 0.94778200 | -2.11215400 | 0.37538100 |
| N | 2.36762100 | 0.18843800 | -1.53965600 |
| C | 2.51278500 | 1.45271400 | -2.23567900 |

| | | | |
|---|---|---|---|
| H | 3.14476500 | 2.16221400 | -1.67485800 |
| H | 2.97379100 | 1.28288700 | -3.21939000 |
| H | 1.53479700 | 1.91513600 | -2.41992700 |
| N | -1.05814200 | 1.73651500 | 1.40593300 |
| C | 0.35328300 | 3.10495200 | 0.07245600 |
| O | 1.44793100 | 3.77382000 | 0.33966300 |
| C | 1.61780600 | 5.07242600 | -0.27265300 |
| H | 2.60399800 | 5.42436800 | 0.04997800 |
| H | 1.57619100 | 4.98440900 | -1.36712500 |
| H | 0.83201400 | 5.75722700 | 0.07581700 |
| O | -0.54459600 | 3.49765600 | -0.66192700 |
| H | 1.39936800 | 2.17226500 | 2.49170600 |
| H | 2.26095000 | 1.18981900 | 1.29833700 |
| H | -1.74118700 | 2.13621100 | -1.08488300 |
| H | -1.75669700 | 2.39328300 | 1.06435900 |

# E-1

| | | | |
|---|---|---|---|
| C | 1.18384800 | -1.99362600 | 1.59538400 |
| C | 1.27527700 | 0.69312800 | -0.38924300 |
| C | 0.04434300 | 0.01950200 | -0.93925800 |
| C | -0.09246500 | -1.57137500 | -0.49777300 |
| C | -1.35216500 | -1.94016700 | 0.29313900 |
| H | 1.16932000 | 1.27965100 | 0.52146300 |
| H | 0.16429700 | -0.01650900 | -2.03031700 |
| C | 0.11539900 | -1.52887500 | 3.80115200 |
| C | 0.01097200 | -1.71507600 | 2.40542100 |
| C | -1.25008300 | -1.63872600 | 1.76575600 |
| C | -2.38257100 | -1.37609300 | 2.53825100 |
| C | -2.26961300 | -1.18271600 | 3.91856100 |
| C | -1.02200200 | -1.25745400 | 4.55265500 |
| H | 1.08792400 | -1.58245200 | 4.29227900 |
| H | -3.36147900 | -1.31560300 | 2.05759200 |
| H | -3.16573800 | -0.96852100 | 4.50642900 |
| H | -0.94182000 | -1.10236600 | 5.63053500 |
| C | 2.51351300 | -2.30569600 | 2.19421400 |
| H | 2.87937800 | -1.42514100 | 2.74726000 |
| H | 2.42436600 | -3.13186000 | 2.91553000 |
| H | 3.25340400 | -2.56843200 | 1.42650900 |
| C | 2.49879600 | 0.64968900 | -0.97443800 |
| O | 2.80314600 | -0.11602100 | -2.04944500 |
| C | 3.66803100 | 1.42983000 | -0.48922000 |
| C | 4.97161500 | 1.00770200 | -0.81273500 |

| | | | |
|---|---|---|---|
| C | 3.51225600 | 2.59947000 | 0.27996100 |
| C | 6.08489900 | 1.72102500 | -0.36233800 |
| H | 5.10606600 | 0.11292000 | -1.42200700 |
| C | 4.62640300 | 3.30908600 | 0.72978300 |
| H | 2.51213500 | 2.97533600 | 0.50588800 |
| C | 5.91790300 | 2.87214500 | 0.41302400 |
| H | 7.08935300 | 1.37632700 | -0.62237100 |
| H | 4.48507900 | 4.21935500 | 1.31835000 |
| H | 6.78921700 | 3.43360000 | 0.76015000 |
| C | -2.80159200 | 2.14577800 | 0.25692100 |
| C | -3.26441800 | 1.66415400 | -1.00186900 |
| C | -4.52754600 | 2.01063000 | -1.50937400 |
| C | -5.32443900 | 2.85240600 | -0.73690100 |
| C | -4.88164700 | 3.34180100 | 0.51330900 |
| C | -3.63097700 | 2.99749600 | 1.01539200 |
| C | -1.49356000 | 1.59550900 | 0.44503000 |
| C | -1.19992100 | 0.81983700 | -0.65974200 |
| H | -4.88002300 | 1.63522900 | -2.47219600 |
| H | -6.31290200 | 3.13982700 | -1.10502100 |
| H | -5.53503300 | 4.00117900 | 1.09069700 |
| H | -3.29312600 | 3.38103200 | 1.98206400 |
| H | -0.84396500 | 1.75654600 | 1.30207300 |
| N | -2.27773700 | 0.86423500 | -1.54836700 |
| C | -2.40308300 | 0.23758000 | -2.85154400 |
| H | -3.03985200 | -0.66250800 | -2.81549800 |
| H | -2.84865100 | 0.94819000 | -3.56332700 |
| H | -1.41930700 | -0.04180600 | -3.24818400 |
| N | 1.09234600 | -1.96096000 | 0.28980200 |
| C | 0.06177300 | -2.36522800 | -1.80731900 |
| O | -0.98042400 | -3.08640100 | -2.14724800 |
| C | -0.90954900 | -3.82806900 | -3.38762500 |
| H | -1.87700100 | -4.33282200 | -3.48573000 |
| H | -0.74252500 | -3.14175900 | -4.22916100 |
| H | -0.09328700 | -4.56232400 | -3.33972200 |
| O | 1.08927200 | -2.28732100 | -2.45996500 |
| H | -1.52640100 | -3.02400900 | 0.18551600 |
| H | -2.22822900 | -1.44552400 | -0.14283200 |
| H | 2.12790500 | -0.79540500 | -2.24907900 |
| H | 1.94631100 | -2.12914500 | -0.24512400 |

# F-1

| | | | |
|---|---|---|---|
| C | 1.70641800 | 1.56517400 | 1.91589400 |

| | | | |
|---|---|---|---|
| C | -1.88058400 | 0.56088700 | 0.14998900 |
| C | -0.49553100 | 0.65691500 | -0.51611900 |
| C | 0.30067200 | 1.95935200 | -0.09061200 |
| C | 1.61503100 | 2.16255800 | -0.87501500 |
| H | -1.80056800 | 0.27476000 | 1.20987900 |
| H | -0.66676900 | 0.80161400 | -1.58990200 |
| C | 3.97688900 | 0.55541200 | 1.66854200 |
| C | 2.84041500 | 1.16362900 | 1.09686000 |
| C | 2.79783500 | 1.43066200 | -0.29240100 |
| C | 3.89934900 | 1.09072900 | -1.07868500 |
| C | 5.02116400 | 0.48184100 | -0.50514700 |
| C | 5.06114600 | 0.21156700 | 0.86758900 |
| H | 4.00585500 | 0.33777800 | 2.73705900 |
| H | 3.88551100 | 1.30514200 | -2.15013800 |
| H | 5.87194800 | 0.21525100 | -1.13717500 |
| H | 5.93638800 | -0.26876800 | 1.30971600 |
| C | 1.77191900 | 1.58511200 | 3.40568000 |
| H | 1.93284100 | 0.56233900 | 3.78317600 |
| H | 2.62646700 | 2.19298300 | 3.73996100 |
| H | 0.84663700 | 1.98277900 | 3.84372600 |
| C | -2.79359400 | -0.42197600 | -0.58436100 |
| O | -2.46930600 | -0.87000900 | -1.67063000 |
| C | -4.10198200 | -0.79277700 | 0.04156900 |
| C | -4.90001300 | -1.74989900 | -0.61337100 |
| C | -4.56714600 | -0.22037700 | 1.23976600 |
| C | -6.13033800 | -2.13024500 | -0.08109900 |
| H | -4.53198100 | -2.18723000 | -1.54336800 |
| C | -5.80346600 | -0.59814400 | 1.76870900 |
| H | -3.97503500 | 0.53035000 | 1.76649700 |
| C | -6.58489600 | -1.55393900 | 1.11163900 |
| H | -6.73966500 | -2.87801300 | -0.59506400 |
| H | -6.15871800 | -0.14499300 | 2.69758400 |
| H | -7.55060800 | -1.85073400 | 1.52920700 |
| C | 1.29949400 | -2.46108300 | 0.53542100 |
| C | 1.58236000 | -2.38002200 | -0.85984000 |
| C | 2.38028900 | -3.33781600 | -1.50736400 |
| C | 2.89246900 | -4.38076200 | -0.73915300 |
| C | 2.62174300 | -4.47728100 | 0.64459300 |
| C | 1.83151600 | -3.52929000 | 1.28632900 |
| C | 0.46983100 | -1.33554500 | 0.84569300 |
| C | 0.28110900 | -0.62050500 | -0.32305400 |
| H | 2.60174100 | -3.27290000 | -2.57480400 |
| H | 3.51772900 | -5.13943200 | -1.21732200 |

| | | | |
|---|---|---|---|
| H | 3.04024800 | -5.31109900 | 1.21433800 |
| H | 1.62257700 | -3.61130700 | 2.35670700 |
| H | 0.04895500 | -1.09181200 | 1.81894300 |
| N | 0.94718200 | -1.25892100 | -1.36393100 |
| C | 0.89613200 | -0.91340700 | -2.77253300 |
| H | 1.03595300 | -1.82209500 | -3.37245600 |
| H | -0.09020300 | -0.50505500 | -3.02747100 |
| H | 1.67868200 | -0.18895100 | -3.05391600 |
| N | 0.59504000 | 1.95769900 | 1.34890400 |
| H | -0.14880400 | 2.33083900 | 1.94416900 |
| C | -0.61415000 | 3.18011000 | -0.31502700 |
| O | -0.85323600 | 3.36994000 | -1.60259200 |
| C | -1.70185900 | 4.47950100 | -1.96194900 |
| H | -1.75161800 | 4.47559000 | -3.05683400 |
| H | -2.70545100 | 4.34664000 | -1.53283800 |
| H | -1.26880600 | 5.42294400 | -1.60043700 |
| O | -1.05312600 | 3.85854800 | 0.58475400 |
| H | 1.86101400 | 3.23938000 | -0.88076100 |
| H | 1.45751100 | 1.88026300 | -1.92373200 |
| H | -2.40537700 | 1.53216000 | 0.14293100 |

# TS4

| | | | |
|---|---|---|---|
| C | 1.60777900 | 0.71242600 | 1.61947300 |
| C | -1.97522100 | 0.46972900 | 0.03573500 |
| C | -0.55984600 | 0.70719100 | -0.52637700 |
| C | 0.16159900 | 1.98921000 | 0.14871800 |
| C | 1.35558800 | 2.47409400 | -0.69995800 |
| H | -1.95653600 | 0.28930000 | 1.12260800 |
| H | -0.64848000 | 0.93572100 | -1.59432500 |
| C | 3.98949700 | 0.21170100 | 0.91149700 |
| C | 2.76818300 | 0.86724800 | 0.68125500 |
| C | 2.62904400 | 1.69449400 | -0.45275000 |
| C | 3.71441900 | 1.84653200 | -1.32612000 |
| C | 4.92442900 | 1.19000600 | -1.09042200 |
| C | 5.06289500 | 0.37231900 | 0.03463200 |
| H | 4.10982900 | -0.43053000 | 1.78406600 |
| H | 3.60898500 | 2.49752400 | -2.19870400 |
| H | 5.75927200 | 1.32036500 | -1.78366200 |
| H | 6.00617500 | -0.14273900 | 0.23113900 |
| C | 1.91043400 | 0.38627800 | 3.06502900 |
| H | 2.46129300 | -0.55612200 | 3.16793400 |
| H | 2.52770700 | 1.19239100 | 3.49427100 |

| | | | |
|---|---|---|---|
| H | 0.98084900 | 0.31234600 | 3.64835800 |
| C | -2.69417200 | -0.69157900 | -0.64427000 |
| O | -2.14332000 | -1.32711200 | -1.52861400 |
| C | -4.08537200 | -1.01770400 | -0.20399400 |
| C | -4.74053300 | -2.10188300 | -0.81813000 |
| C | -4.75835600 | -0.28489200 | 0.79155300 |
| C | -6.03764800 | -2.44748400 | -0.44438600 |
| H | -4.21142200 | -2.66330400 | -1.59049100 |
| C | -6.05855900 | -0.63273100 | 1.16385200 |
| H | -4.27632400 | 0.56286700 | 1.28190900 |
| C | -6.69898300 | -1.71299100 | 0.54797800 |
| H | -6.53827900 | -3.29098000 | -0.92642900 |
| H | -6.57423800 | -0.05852000 | 1.93755000 |
| H | -7.71680600 | -1.98335100 | 0.84116500 |
| C | 1.58698800 | -2.08660800 | 0.74314500 |
| C | 1.80629700 | -2.13072500 | -0.65017300 |
| C | 2.65835900 | -3.06082100 | -1.24891000 |
| C | 3.28567600 | -3.98131300 | -0.40565200 |
| C | 3.06283800 | -3.97002400 | 0.98320700 |
| C | 2.21558300 | -3.02626900 | 1.56951900 |
| C | 0.69101700 | -0.95996300 | 0.99183400 |
| C | 0.31510600 | -0.47980700 | -0.31308500 |
| H | 2.83226700 | -3.07678100 | -2.32588300 |
| H | 3.95817000 | -4.72728500 | -0.83607300 |
| H | 3.55879700 | -4.71414600 | 1.61117200 |
| H | 2.04198100 | -3.02933100 | 2.64817300 |
| H | -0.02717300 | -0.96722900 | 1.81184100 |
| N | 1.01854800 | -1.12840200 | -1.25372100 |
| C | 0.98458400 | -0.91603100 | -2.69294600 |
| H | 0.94579000 | -1.89245300 | -3.19482300 |
| H | 0.08581300 | -0.35608100 | -2.96882900 |
| H | 1.88461400 | -0.37678900 | -3.02624700 |
| N | 0.62923900 | 1.66919400 | 1.47046400 |
| C | -0.88312000 | 3.10789500 | 0.28695500 |
| O | -1.18428900 | 3.63856600 | -0.89337300 |
| C | -2.15852100 | 4.69897200 | -0.90830000 |
| H | -2.25645000 | 5.00270600 | -1.95711700 |
| H | -3.12429800 | 4.34104300 | -0.52284800 |
| H | -1.81343500 | 5.54467700 | -0.29616600 |
| O | -1.37356100 | 3.43897800 | 1.33991700 |
| H | 1.54950000 | 3.53059500 | -0.44694800 |
| H | 1.09090400 | 2.46164600 | -1.76668500 |
| H | -2.60095800 | 1.36377400 | -0.11149900 |

| | | | |
|---|---|---|---|
| H | -0.06257000 | 1.75806700 | 2.21245100 |

## G-1

| | | | |
|---|---|---|---|
| C | 1.50095000 | 0.46757100 | 1.53256200 |
| C | -1.95012000 | 0.56757100 | -0.06796200 |
| C | -0.53849600 | 0.75846400 | -0.66973900 |
| C | 0.20565800 | 1.95317900 | 0.05389200 |
| C | 1.47283900 | 2.40710700 | -0.72243100 |
| H | -1.88680100 | 0.55156500 | 1.03199700 |
| H | -0.62853100 | 0.99099900 | -1.73695800 |
| C | 4.01515900 | 0.16985300 | 1.07267500 |
| C | 2.78745000 | 0.77258000 | 0.74536500 |
| C | 2.73496200 | 1.65467300 | -0.35191800 |
| C | 3.90340500 | 1.90604400 | -1.09173900 |
| C | 5.11333900 | 1.29969500 | -0.75933900 |
| C | 5.16972500 | 0.42929000 | 0.33372200 |
| H | 4.07591300 | -0.51712900 | 1.91752700 |
| H | 3.85687000 | 2.60061000 | -1.93590900 |
| H | 6.01123100 | 1.51084700 | -1.34585100 |
| H | 6.11236100 | -0.04893700 | 0.61156100 |
| C | 1.74816000 | 0.25168200 | 3.02736000 |
| H | 2.38918700 | -0.61594500 | 3.22578300 |
| H | 2.24043200 | 1.13875500 | 3.45633200 |
| H | 0.78844300 | 0.10051900 | 3.54313900 |
| C | -2.67984700 | -0.67373100 | -0.55333600 |
| O | -2.12732900 | -1.45734900 | -1.31152400 |
| C | -4.07606900 | -0.91369700 | -0.07755900 |
| C | -4.74189800 | -2.06940400 | -0.52865400 |
| C | -4.74007200 | -0.04012600 | 0.80397200 |
| C | -6.04137500 | -2.34568900 | -0.10861300 |
| H | -4.21888800 | -2.74167400 | -1.21140900 |
| C | -6.04207200 | -0.31955600 | 1.22399500 |
| H | -4.24736000 | 0.86255100 | 1.16898400 |
| C | -6.69361300 | -1.47077200 | 0.76939300 |
| H | -6.55068800 | -3.24510600 | -0.46374500 |
| H | -6.54995800 | 0.36336800 | 1.90938000 |
| H | -7.71281800 | -1.68742000 | 1.09996900 |
| C | 1.61773700 | -2.05418400 | 0.63546800 |
| C | 1.62285600 | -2.26079700 | -0.75032400 |
| C | 2.27848800 | -3.32246800 | -1.36538300 |
| C | 2.95488400 | -4.21423200 | -0.52455000 |
| C | 2.95211300 | -4.03904700 | 0.86594200 |
| C | 2.28016900 | -2.96125200 | 1.45967000 |

| | | | |
|---|---|---|---|
| C | 0.79427300 | -0.82657800 | 0.90942800 |
| C | 0.31031600 | -0.45071300 | -0.46036400 |
| H | 2.26550700 | -3.46730400 | -2.44619100 |
| H | 3.48384900 | -5.06400400 | -0.96218700 |
| H | 3.47754000 | -4.75813500 | 1.49874600 |
| H | 2.27632700 | -2.84558800 | 2.54499200 |
| H | -0.06605500 | -1.03946500 | 1.56460900 |
| N | 0.83357500 | -1.24057800 | -1.36600600 |
| C | 0.62974400 | -1.21386900 | -2.80906300 |
| H | 1.61022100 | -1.18587900 | -3.30461500 |
| H | 0.09178000 | -2.12468500 | -3.10658300 |
| H | 0.04337700 | -0.33762600 | -3.09755700 |
| N | 0.50187600 | 1.52428600 | 1.40551800 |
| C | -0.70762200 | 3.18655400 | 0.13932900 |
| O | -1.22022600 | 3.49823100 | -1.05319800 |
| C | -2.01411100 | 4.69522200 | -1.13107800 |
| H | -2.36288800 | 4.76012100 | -2.16870300 |
| H | -2.87087400 | 4.63766700 | -0.44409700 |
| H | -1.40610300 | 5.57636800 | -0.87831800 |
| O | -0.88105300 | 3.82762400 | 1.14657000 |
| H | 1.65196700 | 3.47350600 | -0.49814700 |
| H | 1.29485900 | 2.36017200 | -1.80785800 |
| H | -2.56403200 | 1.44423100 | -0.32624100 |
| H | 0.67424800 | 2.31340400 | 2.02567100 |

# TS-(1S,2S,3S,4S)

| | | | |
|---|---|---|---|
| C | -1.79255400 | 1.72842900 | -3.47762900 |
| C | -2.11509100 | 1.37610600 | -1.99230900 |
| C | -2.28269100 | -0.15720700 | -1.80373300 |
| H | -1.31450200 | -0.60646500 | -1.51432200 |
| H | -2.61736800 | -0.64504700 | -2.73185600 |
| P | -3.51690600 | -0.67064400 | -0.55592400 |
| C | -5.15473900 | -0.09317200 | -1.11650400 |
| C | -5.96418700 | -0.91268900 | -1.92453800 |
| C | -5.56799900 | 1.22078900 | -0.82925100 |
| C | -7.17788600 | -0.43051000 | -2.41911000 |
| H | -5.65314700 | -1.93019100 | -2.16818500 |
| C | -6.78385100 | 1.69436000 | -1.33007200 |
| H | -4.93313800 | 1.89485900 | -0.24728300 |
| C | -7.59138100 | 0.87106900 | -2.11915000 |
| H | -7.80142300 | -1.07586900 | -3.04241300 |

| | | | |
|---|---|---|---|
| H | -7.09380500 | 2.71727800 | -1.10406300 |
| H | -8.54212900 | 1.24556500 | -2.50663600 |
| C | -3.54153100 | -2.48662500 | -0.48626500 |
| C | -4.63168400 | -3.14682600 | 0.11186300 |
| C | -2.45571000 | -3.23479800 | -0.97451000 |
| C | -4.64319700 | -4.53973200 | 0.19942600 |
| H | -5.47370000 | -2.57724200 | 0.50975400 |
| C | -2.48014300 | -4.62942000 | -0.88547500 |
| H | -1.57768900 | -2.74507100 | -1.39951000 |
| C | -3.56985500 | -5.28235200 | -0.30324000 |
| H | -5.49305200 | -5.04479500 | 0.66442400 |
| H | -1.63470200 | -5.20490900 | -1.26955400 |
| H | -3.58178000 | -6.37321500 | -0.23666900 |
| N | -1.08967500 | 1.84101900 | -1.08344200 |
| H | -0.12280900 | 1.52609800 | -1.28885400 |
| H | -3.03678200 | 1.91549600 | -1.72652400 |
| C | -1.19669600 | 2.87644400 | -0.20492800 |
| C | -3.09616300 | 0.03684800 | 1.08628100 |
| C | -3.92761000 | -0.46464100 | 2.24363100 |
| C | -5.10241900 | 0.19408800 | 2.64258600 |
| C | -3.51201800 | -1.59818600 | 2.96333400 |
| C | -5.84904200 | -0.27540800 | 3.72696200 |
| C | -4.25737700 | -2.06553100 | 4.04863000 |
| C | -5.43069000 | -1.40823100 | 4.43148700 |
| H | -3.14214800 | 1.13581800 | 0.97602100 |
| H | -2.04016600 | -0.25084500 | 1.20121700 |
| H | -2.58813700 | -2.09951100 | 2.66724800 |
| H | -3.91667900 | -2.94439700 | 4.60226500 |
| H | -6.01215300 | -1.77139500 | 5.28272100 |
| H | -6.75677200 | 0.25354400 | 4.02837100 |
| H | -5.42804000 | 1.09169000 | 2.11239900 |
| C | -3.01085400 | 1.49286300 | -4.38123900 |
| H | -2.75532700 | 1.69497500 | -5.43319700 |
| H | -3.39305200 | 0.46043500 | -4.33537600 |
| H | -3.83983100 | 2.16636600 | -4.10534000 |
| N | 0.02203700 | 3.24632800 | 0.27948600 |
| H | 0.80392900 | 2.76143000 | -0.19590700 |
| C | 0.43963800 | 4.14733100 | 1.27549000 |
| C | 1.83638100 | 4.30153100 | 1.41380800 |
| C | -0.40473600 | 4.87309400 | 2.13552600 |
| C | 2.37767100 | 5.15150300 | 2.37591500 |
| H | 2.50395900 | 3.74472700 | 0.75113300 |

| | | | |
|---|---|---|---|
| C | 0.13897000 | 5.72605300 | 3.09966400 |
| H | -1.48293000 | 4.76942700 | 2.03987400 |
| C | 1.51859500 | 5.86002600 | 3.21302100 |
| H | 3.45764400 | 5.27123300 | 2.48147100 |
| H | -0.50978200 | 6.29410800 | 3.76985800 |
| F | 2.03237200 | 6.68422400 | 4.14322900 |
| C | 1.63854800 | -1.84496100 | -2.58659600 |
| C | 3.27373200 | 0.12040000 | -0.78365100 |
| C | 2.54659100 | -0.38871100 | 0.33501300 |
| C | 1.55463200 | -2.16290800 | -0.27614800 |
| C | 2.60426000 | -3.26532100 | -0.32758900 |
| H | 4.29603800 | -0.21695200 | -0.94491200 |
| H | 1.61669100 | 0.15846200 | 0.50206300 |
| C | 3.73585600 | -2.46499600 | -3.86854900 |
| C | 2.96704900 | -2.49230700 | -2.69008200 |
| C | 3.46161400 | -3.18606700 | -1.56437400 |
| C | 4.70584000 | -3.81844600 | -1.63490300 |
| C | 5.46685400 | -3.78013300 | -2.80915400 |
| C | 4.97731300 | -3.10275100 | -3.92870400 |
| H | 3.37234800 | -1.92756100 | -4.74559600 |
| H | 5.08375800 | -4.35142300 | -0.75731700 |
| H | 6.43856800 | -4.27932600 | -2.84779000 |
| H | 5.56307900 | -3.06698500 | -4.85084100 |
| C | 0.99531700 | -1.32321200 | -3.84628500 |
| H | 1.55189300 | -0.46054900 | -4.25040300 |
| H | 0.96472800 | -2.09044400 | -4.63811300 |
| H | -0.02852500 | -0.99661500 | -3.63067600 |
| C | 2.74974200 | 1.13131900 | -1.60512700 |
| O | 1.61368500 | 1.69131200 | -1.41213000 |
| C | 3.58082900 | 1.66295700 | -2.74483000 |
| C | 3.20342000 | 2.88366100 | -3.33494500 |
| C | 4.70949300 | 0.99914300 | -3.26346100 |
| C | 3.93222200 | 3.43119800 | -4.39181200 |
| H | 2.32594100 | 3.39404200 | -2.93598500 |
| C | 5.43619100 | 1.54288500 | -4.32523400 |
| H | 5.01889200 | 0.03914100 | -2.84904000 |
| C | 5.05400000 | 2.76275100 | -4.89301300 |
| H | 3.62447000 | 4.38603000 | -4.82729600 |
| H | 6.30658600 | 1.00687000 | -4.71360200 |
| H | 5.62600200 | 3.18810300 | -5.72207300 |
| C | 4.78836600 | -1.48940200 | 3.09225700 |
| C | 3.67583100 | -0.91660900 | 3.77873600 |

| | | | |
|---|---|---|---|
| C | 3.63840300 | -0.83837600 | 5.17950000 |
| C | 4.71530700 | -1.36358000 | 5.89155400 |
| C | 5.81639300 | -1.94843300 | 5.22887900 |
| C | 5.86165700 | -2.01337400 | 3.84012600 |
| C | 4.50994000 | -1.36845100 | 1.69573100 |
| C | 3.27345400 | -0.75619200 | 1.55802600 |
| H | 2.79946700 | -0.37877500 | 5.70638400 |
| H | 4.70822800 | -1.31643400 | 6.98363700 |
| H | 6.64518600 | -2.34858000 | 5.81868100 |
| H | 6.72087500 | -2.45909500 | 3.33163200 |
| H | 5.14688300 | -1.69041200 | 0.87496700 |
| N | 2.75878400 | -0.49489800 | 2.83286300 |
| C | 1.60059900 | 0.32324200 | 3.13695000 |
| H | 0.77310200 | 0.10019000 | 2.45216700 |
| H | 1.25747200 | 0.10114900 | 4.15642600 |
| H | 1.82924400 | 1.40162900 | 3.07574500 |
| N | 0.97440900 | -1.75458500 | -1.47245900 |
| C | 0.59423800 | -2.26009000 | 0.83922600 |
| O | 1.03644900 | -3.09612300 | 1.80658500 |
| C | 0.24516900 | -3.24069900 | 2.97644300 |
| H | 0.83359400 | -3.85373600 | 3.67065400 |
| H | 0.02273800 | -2.26648300 | 3.43572900 |
| H | -0.70607100 | -3.74785100 | 2.74694100 |
| O | -0.46394400 | -1.65215700 | 0.95595800 |
| H | 2.09848900 | -4.25254900 | -0.30553900 |
| H | 3.22613900 | -3.24682800 | 0.57909000 |
| C | -1.29690100 | 3.17368200 | -3.61223700 |
| H | -1.13177800 | 3.42236900 | -4.67251800 |
| H | -2.03051500 | 3.88761800 | -3.20327100 |
| H | -0.34595700 | 3.31931200 | -3.08203200 |
| H | -0.97594800 | 1.06188700 | -3.80889300 |
| S | -2.69155600 | 3.58209400 | 0.20684700 |

# TS-(1S,2S,3S,4S)-conformation-2

| | | | |
|---|---|---|---|
| C | 1.14849000 | -2.40586600 | 3.73358200 |
| H | 1.07121100 | -2.82787000 | 4.74838600 |
| H | 0.18926100 | -1.92032200 | 3.49810300 |
| H | 1.27983800 | -3.25496600 | 3.04251500 |
| C | 2.32334800 | -1.42632700 | 3.65346900 |
| C | 2.37016900 | -0.59705200 | 2.33623000 |
| C | 2.46802000 | -1.46199900 | 1.04954800 |

| | | | |
|---|---|---|---|
| H | 1.47237400 | -1.59074000 | 0.59106000 |
| H | 2.88046600 | -2.46233700 | 1.25349800 |
| P | 3.57635300 | -0.75319000 | -0.22616100 |
| C | 5.26125800 | -0.74473100 | 0.47239900 |
| C | 6.09071000 | -1.87418600 | 0.34375700 |
| C | 5.69598000 | 0.34812800 | 1.24430900 |
| C | 7.34435100 | -1.89794600 | 0.95804800 |
| H | 5.76317100 | -2.73800900 | -0.23777300 |
| C | 6.95200400 | 0.31468200 | 1.85651700 |
| H | 5.05075300 | 1.21518300 | 1.40524500 |
| C | 7.77864600 | -0.80226300 | 1.71057900 |
| H | 7.98266300 | -2.77791200 | 0.84881000 |
| H | 7.27861600 | 1.16746900 | 2.45606400 |
| H | 8.76069800 | -0.82281100 | 2.18952100 |
| C | 3.54893400 | -1.82514500 | -1.69188300 |
| C | 4.62787600 | -1.81253800 | -2.59625600 |
| C | 2.41699100 | -2.61457000 | -1.96415800 |
| C | 4.58688300 | -2.60589300 | -3.74366000 |
| H | 5.50080400 | -1.18449700 | -2.40916100 |
| C | 2.38998200 | -3.40749600 | -3.11489900 |
| H | 1.53826400 | -2.59663700 | -1.31475900 |
| C | 3.47081500 | -3.40901600 | -4.00042100 |
| H | 5.42859100 | -2.59281500 | -4.44005000 |
| H | 1.50891600 | -4.02026800 | -3.31904400 |
| H | 3.44173800 | -4.03225400 | -4.89784600 |
| N | 1.23472900 | 0.28963500 | 2.21119000 |
| H | 0.30328500 | -0.14735200 | 2.09426200 |
| H | 3.25109200 | 0.05376700 | 2.43207000 |
| H | 2.18016600 | -0.65906100 | 4.43434500 |
| C | 1.23632200 | 1.62644100 | 2.47820100 |
| C | 3.03069400 | 0.94716200 | -0.65840500 |
| C | 3.78269800 | 1.59444100 | -1.79926800 |
| C | 4.87574800 | 2.44538600 | -1.56652200 |
| C | 3.36975700 | 1.37354900 | -3.12478000 |
| C | 5.54808400 | 3.04999000 | -2.63237600 |
| C | 4.04236700 | 1.97856000 | -4.18944600 |
| C | 5.13645700 | 2.81503700 | -3.94745300 |
| H | 3.07468400 | 1.54453000 | 0.27014300 |
| H | 1.97146300 | 0.79562400 | -0.91977100 |
| H | 2.50096700 | 0.73731400 | -3.30548200 |
| H | 3.70502900 | 1.80151500 | -5.21411100 |
| H | 5.65999400 | 3.29137400 | -4.78030300 |

| | | | |
|---|---|---|---|
| H | 6.39161000 | 3.71553800 | -2.43212600 |
| H | 5.19277700 | 2.65486100 | -0.54273600 |
| C | 3.66127900 | -2.11955300 | 3.93821800 |
| H | 3.64253600 | -2.59662900 | 4.93094800 |
| H | 3.88103400 | -2.91639200 | 3.20652200 |
| H | 4.50275700 | -1.40829100 | 3.92433500 |
| N | -0.02995200 | 2.12907700 | 2.50993500 |
| H | -0.75251800 | 1.39466000 | 2.40967700 |
| C | -0.55926200 | 3.42664100 | 2.63089100 |
| C | -1.96751500 | 3.51969800 | 2.58079000 |
| C | 0.18682300 | 4.60873500 | 2.78988900 |
| C | -2.61448000 | 4.74921900 | 2.68467900 |
| H | -2.56073700 | 2.60953700 | 2.45956200 |
| C | -0.46304600 | 5.84162800 | 2.89299100 |
| H | 1.27201000 | 4.55416400 | 2.83400100 |
| C | -1.85131000 | 5.90434000 | 2.84042200 |
| H | -3.70319900 | 4.82009700 | 2.64645700 |
| H | 0.10903700 | 6.76353400 | 3.01765200 |
| F | -2.46744400 | 7.09531800 | 2.94148100 |
| C | -1.55818300 | -3.26885100 | -0.14680300 |
| C | -3.17226100 | -0.68044200 | 0.65345100 |
| C | -2.52354900 | -0.00830700 | -0.42617500 |
| C | -1.57447400 | -1.48365100 | -1.64995900 |
| C | -2.66873100 | -2.12465100 | -2.49277200 |
| H | -4.19738600 | -1.02341000 | 0.52316100 |
| H | -1.57792100 | 0.44356400 | -0.12034400 |
| C | -3.63085900 | -4.70179800 | 0.13177400 |
| C | -2.90924700 | -3.71218900 | -0.56174200 |
| C | -3.47719100 | -3.13153200 | -1.71630400 |
| C | -4.74684600 | -3.53518100 | -2.13888800 |
| C | -5.46099300 | -4.51364900 | -1.43774800 |
| C | -4.89812500 | -5.09949000 | -0.30094500 |
| H | -3.20879800 | -5.15783200 | 1.02853000 |
| H | -5.18177500 | -3.07806500 | -3.03267000 |
| H | -6.45339900 | -4.81761800 | -1.78076000 |
| H | -5.44650700 | -5.86595500 | 0.25275500 |
| C | -0.84021400 | -4.05895900 | 0.91674200 |
| H | -1.34907200 | -3.97464800 | 1.89171000 |
| H | -0.79526400 | -5.13236000 | 0.66567900 |
| H | 0.18080200 | -3.67783300 | 1.03327200 |
| C | -2.57146700 | -0.79636700 | 1.91712100 |
| O | -1.43055200 | -0.29383100 | 2.21398100 |

| | | | |
|---|---|---|---|
| C | -3.32366100 | -1.47133800 | 3.03454200 |
| C | -2.91083000 | -1.23231000 | 4.35874500 |
| C | -4.41538500 | -2.33605800 | 2.82679200 |
| C | -3.57062200 | -1.82210100 | 5.43788900 |
| H | -2.06259100 | -0.56446100 | 4.51627800 |
| C | -5.07160900 | -2.93306800 | 3.90587300 |
| H | -4.74804100 | -2.56288700 | 1.81318400 |
| C | -4.65546700 | -2.67700800 | 5.21652500 |
| H | -3.23829300 | -1.61313000 | 6.45864700 |
| H | -5.91337800 | -3.60590600 | 3.72023100 |
| H | -5.17280500 | -3.14248500 | 6.05980700 |
| C | -4.94864600 | 1.64594300 | -2.70923000 |
| C | -3.84226900 | 2.54751300 | -2.69343400 |
| C | -3.86927300 | 3.75621500 | -3.40627900 |
| C | -5.00616700 | 4.04418400 | -4.15927200 |
| C | -6.10304800 | 3.15584800 | -4.19917700 |
| C | -6.08376800 | 1.96463700 | -3.48126000 |
| C | -4.59605700 | 0.55613400 | -1.85518200 |
| C | -3.32352800 | 0.79641700 | -1.35812500 |
| H | -3.03399000 | 4.45901600 | -3.37570300 |
| H | -5.05031200 | 4.97845800 | -4.72504800 |
| H | -6.97982900 | 3.41472000 | -4.79845000 |
| H | -6.93964200 | 1.28475100 | -3.50820000 |
| H | -5.20734300 | -0.31304200 | -1.62319400 |
| N | -2.85912800 | 2.00697000 | -1.88506700 |
| C | -1.67417800 | 2.72911500 | -1.46403400 |
| H | -0.83688000 | 2.04113300 | -1.29521100 |
| H | -1.37440800 | 3.42842000 | -2.25648400 |
| H | -1.84958800 | 3.30685500 | -0.53979800 |
| N | -0.93634800 | -2.26792300 | -0.69587400 |
| C | -0.66029800 | -0.58637000 | -2.37947900 |
| O | -1.16773400 | -0.22830700 | -3.58199700 |
| C | -0.42328600 | 0.68197000 | -4.37764800 |
| H | -1.07726300 | 0.96704800 | -5.21145500 |
| H | -0.13257400 | 1.57475000 | -3.80558000 |
| H | 0.48957800 | 0.20409400 | -4.76950000 |
| O | 0.41467900 | -0.14862800 | -1.98488900 |
| H | -2.20803400 | -2.63249400 | -3.36507700 |
| H | -3.32164400 | -1.35411900 | -2.92780400 |
| S | 2.67558800 | 2.50518600 | 2.71207000 |

## TS-(1S,2S,3S,4S)-conformation-3

| | | | |
|---|---|---|---|
| C | 2.40535800 | -0.54707200 | 4.91766600 |
| H | 2.53445000 | -1.16130600 | 5.82299100 |
| H | 3.22842800 | 0.18203200 | 4.87216300 |
| H | 1.46847200 | 0.02079000 | 5.03129600 |
| C | 2.35880300 | -1.43760600 | 3.67004300 |
| C | 2.36111800 | -0.56991800 | 2.37649200 |
| C | 2.44686500 | -1.43935200 | 1.08807400 |
| H | 1.45477100 | -1.53855300 | 0.61431100 |
| H | 2.81886700 | -2.45245500 | 1.30870800 |
| P | 3.60329500 | -0.79989800 | -0.18039400 |
| C | 5.26639600 | -0.79487600 | 0.56840700 |
| C | 6.07848900 | -1.94268700 | 0.50985400 |
| C | 5.69709800 | 0.32011900 | 1.31051500 |
| C | 7.31245500 | -1.96304900 | 1.16297400 |
| H | 5.75296900 | -2.82330300 | -0.04712900 |
| C | 6.93297900 | 0.28944900 | 1.96241600 |
| H | 5.06174700 | 1.20280500 | 1.41734500 |
| C | 7.74348500 | -0.84594000 | 1.88521900 |
| H | 7.93784000 | -2.85724900 | 1.10785100 |
| H | 7.25634800 | 1.15921500 | 2.53887800 |
| H | 8.71003500 | -0.86415800 | 2.39481500 |
| C | 3.59215500 | -1.93482600 | -1.59870300 |
| C | 4.69574300 | -1.98766800 | -2.47119400 |
| C | 2.44773800 | -2.70665600 | -1.87031400 |
| C | 4.66593600 | -2.82789100 | -3.58517800 |
| H | 5.57919400 | -1.37402200 | -2.28563800 |
| C | 2.43196700 | -3.54646400 | -2.98757200 |
| H | 1.55222700 | -2.64035300 | -1.24726800 |
| C | 3.53664900 | -3.61268000 | -3.84064000 |
| H | 5.52683700 | -2.86558400 | -4.25677600 |
| H | 1.54112500 | -4.14509700 | -3.19125500 |
| H | 3.51615900 | -4.27235700 | -4.71186900 |
| N | 1.21801200 | 0.30912100 | 2.28382300 |
| H | 0.28520900 | -0.13338900 | 2.19876300 |
| H | 3.23954300 | 0.08596400 | 2.45641200 |
| H | 3.30320600 | -2.01398300 | 3.63333000 |
| C | 1.22588900 | 1.66565200 | 2.40710900 |
| C | 3.10275700 | 0.88611000 | -0.70759400 |
| C | 3.90103800 | 1.46857500 | -1.85145900 |
| C | 5.00236000 | 2.30979300 | -1.62226800 |
| C | 3.52503200 | 1.19659200 | -3.17847400 |

| | | | |
|---|---|---|---|
| C | 5.71771600 | 2.85523900 | -2.69199800 |
| C | 4.24044400 | 1.74227000 | -4.24724100 |
| C | 5.34180000 | 2.56989400 | -4.00781500 |
| H | 3.12369200 | 1.52476700 | 0.19448600 |
| H | 2.05016700 | 0.73853500 | -0.99713500 |
| H | 2.65174800 | 0.56652200 | -3.35975000 |
| H | 3.93132800 | 1.52561600 | -5.27317300 |
| H | 5.89904500 | 3.00005400 | -4.84390000 |
| H | 6.56714400 | 3.51414900 | -2.49477800 |
| H | 5.29227900 | 2.55786000 | -0.59906400 |
| C | 1.19337700 | -2.43079300 | 3.74665400 |
| H | 1.22562200 | -2.98544300 | 4.69781000 |
| H | 0.22060900 | -1.91667600 | 3.68990700 |
| H | 1.22317600 | -3.17541200 | 2.93698800 |
| N | -0.03993000 | 2.17096600 | 2.40513400 |
| H | -0.76361500 | 1.43248800 | 2.34728900 |
| C | -0.56978800 | 3.47334400 | 2.45038700 |
| C | -1.97818900 | 3.56167300 | 2.39388100 |
| C | 0.17420100 | 4.66371400 | 2.54455900 |
| C | -2.62711200 | 4.79404500 | 2.42840100 |
| H | -2.57011800 | 2.64543100 | 2.32254600 |
| C | -0.47760800 | 5.89947600 | 2.57851900 |
| H | 1.25941100 | 4.61379200 | 2.59141600 |
| C | -1.86585700 | 5.95724100 | 2.52075300 |
| H | -3.71588400 | 4.86088200 | 2.38490600 |
| H | 0.09309300 | 6.82767100 | 2.65258200 |
| F | -2.48371800 | 7.15105400 | 2.55472600 |
| C | -1.58314000 | -3.28785900 | -0.13057600 |
| C | -3.16415200 | -0.67358700 | 0.65336600 |
| C | -2.50516700 | -0.01404500 | -0.42851500 |
| C | -1.56002400 | -1.50427600 | -1.63567100 |
| C | -2.65266700 | -2.13280900 | -2.49003600 |
| H | -4.18718500 | -1.02039800 | 0.51714800 |
| H | -1.55955800 | 0.43536600 | -0.11899500 |
| C | -3.67868800 | -4.69071900 | 0.12933200 |
| C | -2.93602300 | -3.71281700 | -0.55855400 |
| C | -3.48359700 | -3.12634000 | -1.71998300 |
| C | -4.75455900 | -3.51264200 | -2.15483400 |
| C | -5.48977800 | -4.47926900 | -1.45916700 |
| C | -4.94709500 | -5.07093800 | -0.31553100 |
| H | -3.27211000 | -5.15110500 | 1.03104300 |
| H | -5.17372300 | -3.05103000 | -3.05379200 |

| | | | |
|---|---|---|---|
| H | -6.48291500 | -4.76961400 | -1.81172600 |
| H | -5.51211500 | -5.82841900 | 0.23378800 |
| C | -0.88587600 | -4.08722200 | 0.93981800 |
| H | -1.39607300 | -3.98712000 | 1.91270500 |
| H | -0.86171400 | -5.16252200 | 0.69453700 |
| H | 0.14247100 | -3.72602500 | 1.05627200 |
| C | -2.57634900 | -0.77276800 | 1.92458600 |
| O | -1.44348700 | -0.25706500 | 2.22922000 |
| C | -3.33477400 | -1.44427300 | 3.03996400 |
| C | -2.93467100 | -1.19423800 | 4.36599200 |
| C | -4.41969800 | -2.31665400 | 2.82883000 |
| C | -3.59965900 | -1.78126600 | 5.44348900 |
| H | -2.09239600 | -0.51946000 | 4.52590200 |
| C | -5.08149400 | -2.91050000 | 3.90626200 |
| H | -4.74262600 | -2.55178300 | 1.81398600 |
| C | -4.67760700 | -2.64393400 | 5.21867100 |
| H | -3.27694300 | -1.56399200 | 6.46559900 |
| H | -5.91797500 | -3.58918300 | 3.71796600 |
| H | -5.19913200 | -3.10718400 | 6.06059700 |
| C | -4.91339800 | 1.63468200 | -2.73329700 |
| C | -3.79773100 | 2.52470600 | -2.73094500 |
| C | -3.81207500 | 3.72409300 | -3.45966400 |
| C | -4.94656400 | 4.01396600 | -4.21559100 |
| C | -6.05283100 | 3.13678600 | -4.24251100 |
| C | -6.04581100 | 1.95516200 | -3.50858800 |
| C | -4.57093100 | 0.55350300 | -1.86447800 |
| C | -3.29550700 | 0.78771400 | -1.37135100 |
| H | -2.96917300 | 4.41806000 | -3.43827500 |
| H | -4.98154600 | 4.94097800 | -4.79382000 |
| H | -6.92740100 | 3.39693700 | -4.84446900 |
| H | -6.90911800 | 1.28441100 | -3.52564900 |
| H | -5.19045100 | -0.30641300 | -1.62023700 |
| N | -2.81963400 | 1.98609600 | -1.91594000 |
| C | -1.62490400 | 2.70334500 | -1.51578200 |
| H | -0.81069900 | 2.00617300 | -1.28516600 |
| H | -1.28953800 | 3.34344900 | -2.34375900 |
| H | -1.80339200 | 3.34214100 | -0.63345000 |
| N | -0.94264700 | -2.29601500 | -0.67395100 |
| C | -0.62182900 | -0.62537300 | -2.35717900 |
| O | -1.11137100 | -0.25510500 | -3.56330400 |
| C | -0.33653700 | 0.63225900 | -4.35552300 |
| H | -0.98153100 | 0.94597000 | -5.18606800 |

| | | | |
|---|---|---|---|
| H | -0.01071900 | 1.50965700 | -3.77879100 |
| H | 0.55692500 | 0.12318700 | -4.75297300 |
| O | 0.45937600 | -0.21252900 | -1.95342000 |
| H | -2.18845700 | -2.64978900 | -3.35508900 |
| H | -3.29048800 | -1.35519200 | -2.93468400 |
| S | 2.66580100 | 2.56854600 | 2.52976600 |

# TS-(1R,2R,3S,4S)

| | | | |
|---|---|---|---|
| **C** | **-3.20446900** | **3.36324400** | **-1.71845800** |
| C | -3.06015600 | 2.16932500 | -0.72172300 |
| C | -3.31644200 | 0.82913000 | -1.45632800 |
| H | -2.39063700 | 0.50588400 | -1.95875900 |
| H | -4.11288000 | 0.93147400 | -2.20869800 |
| P | -3.83687300 | -0.58435600 | -0.41503500 |
| C | -5.44624200 | -0.16946200 | 0.33193100 |
| C | -6.60457800 | -0.21772200 | -0.46857700 |
| C | -5.53848100 | 0.29375400 | 1.65434700 |
| C | -7.83735600 | 0.17007000 | 0.05739600 |
| H | -6.55030600 | -0.56450700 | -1.50295600 |
| C | -6.77861100 | 0.67740700 | 2.17418000 |
| H | -4.64497200 | 0.40556300 | 2.26952100 |
| C | -7.92650100 | 0.61249000 | 1.38191100 |
| H | -8.73085800 | 0.12677300 | -0.56985000 |
| H | -6.83871500 | 1.04051200 | 3.20261400 |
| H | -8.89301600 | 0.91465400 | 1.79277400 |
| C | -4.06386100 | -2.03951700 | -1.48647700 |
| C | -5.04667000 | -3.00038500 | -1.18646700 |
| C | -3.18844600 | -2.25318900 | -2.56642200 |
| C | -5.17239900 | -4.14276200 | -1.97883300 |
| H | -5.71333400 | -2.86442800 | -0.33346000 |
| C | -3.32503200 | -3.39904400 | -3.35427000 |
| H | -2.37355400 | -1.55563600 | -2.76657400 |
| C | -4.31771000 | -4.33978500 | -3.06760900 |
| H | -5.93890200 | -4.88358000 | -1.73954800 |
| H | -2.64058900 | -3.55941000 | -4.19050200 |
| H | -4.41901200 | -5.23449500 | -3.68698500 |
| N | -1.75631200 | 2.13592900 | -0.08632300 |
| H | -0.93547800 | 2.12766000 | -0.70834300 |
| H | -3.79273500 | 2.31752700 | 0.08725800 |
| C | -1.49687800 | 2.47634400 | 1.21453500 |
| C | -2.53473100 | -0.96036100 | 0.82830400 |

| | | | |
|---|---|---|---|
| C | -2.63149900 | -2.33593100 | 1.44711200 |
| C | -3.41389500 | -2.58027200 | 2.58750400 |
| C | -1.91380400 | -3.40106500 | 0.87579100 |
| C | -3.48807000 | -3.86285600 | 3.13835500 |
| C | -1.99026800 | -4.68263300 | 1.42757300 |
| C | -2.78006700 | -4.91871200 | 2.55703700 |
| H | -2.55218900 | -0.14214700 | 1.56897100 |
| H | -1.61108200 | -0.85928800 | 0.23343300 |
| H | -1.27349700 | -3.21268000 | 0.00912600 |
| H | -1.42135600 | -5.49919600 | 0.97595900 |
| H | -2.83555500 | -5.92090200 | 2.98961000 |
| H | -4.09560900 | -4.03453900 | 4.03058600 |
| H | -3.96044600 | -1.76097100 | 3.05990800 |
| C | -4.67490700 | 3.59842200 | -2.09242900 |
| H | -4.76037300 | 4.40292200 | -2.83955700 |
| H | -5.16157600 | 2.70769600 | -2.52122200 |
| H | -5.25848900 | 3.90285700 | -1.20733600 |
| N | -0.18328600 | 2.75847700 | 1.41342600 |
| H | 0.37430500 | 2.72486700 | 0.53805200 |
| C | 0.57696200 | 3.02942200 | 2.57178400 |
| C | 1.97533900 | 2.89767500 | 2.44053000 |
| C | 0.05054700 | 3.44957200 | 3.80631300 |
| C | 2.82628500 | 3.16475900 | 3.51117100 |
| H | 2.40214400 | 2.57828100 | 1.48622700 |
| C | 0.90393200 | 3.71197300 | 4.88103500 |
| H | -1.02444000 | 3.56628900 | 3.91988700 |
| C | 2.27876900 | 3.56703400 | 4.72700100 |
| H | 3.90778700 | 3.05608700 | 3.41178300 |
| H | 0.50506300 | 4.03807900 | 5.84392400 |
| F | 3.09199400 | 3.82278900 | 5.76623900 |
| C | 2.37639800 | -3.30596100 | -0.78178500 |
| C | 2.85203600 | 1.39165500 | -1.13977500 |
| C | 2.34566700 | 0.27421100 | -0.40770500 |
| C | 1.79721800 | -1.27987800 | -1.81333500 |
| C | 2.96204200 | -1.35539000 | -2.79094400 |
| H | 3.91956200 | 1.44075600 | -1.35152400 |
| H | 1.31814500 | 0.39790100 | -0.06315500 |
| C | 4.68928500 | -4.29066100 | -1.08089700 |
| C | 3.71338100 | -3.33297200 | -1.41605200 |
| C | 4.01493100 | -2.35799500 | -2.39068800 |
| C | 5.27215000 | -2.36422000 | -3.00320800 |
| C | 6.23508500 | -3.31870200 | -2.66262300 |

| | | | |
|---|---|---|---|
| C | 5.94108700 | -4.28558300 | -1.69696200 |
| H | 4.47600800 | -5.04281800 | -0.31996000 |
| H | 5.50079700 | -1.60571700 | -3.75816200 |
| H | 7.21420900 | -3.30550900 | -3.14881800 |
| H | 6.68863600 | -5.03286700 | -1.41899600 |
| C | 2.00477500 | -4.41010500 | 0.17560400 |
| H | 2.09009600 | -5.40525200 | -0.29347700 |
| H | 2.66934900 | -4.41277900 | 1.05719200 |
| H | 0.97392900 | -4.26661800 | 0.52169200 |
| C | 2.03685700 | 2.46192900 | -1.54060400 |
| O | 0.80236600 | 2.58272100 | -1.20838100 |
| C | 2.63074100 | 3.57882200 | -2.35816400 |
| C | 1.97024600 | 4.82040200 | -2.38518200 |
| C | 3.80588100 | 3.43752900 | -3.12022000 |
| C | 2.47492700 | 5.88950700 | -3.12749300 |
| H | 1.05346900 | 4.92775200 | -1.80392700 |
| C | 4.30913300 | 4.50346700 | -3.86844200 |
| H | 4.32264500 | 2.47656400 | -3.14732200 |
| C | 3.64825400 | 5.73658800 | -3.87299300 |
| H | 1.95037400 | 6.84912900 | -3.12461700 |
| H | 5.22040000 | 4.36884200 | -4.45762700 |
| H | 4.04364500 | 6.57123000 | -4.45812100 |
| C | 4.98085800 | -1.24115600 | 1.72452600 |
| C | 3.76808900 | -1.58595800 | 2.39760200 |
| C | 3.77748100 | -2.30186500 | 3.60642500 |
| C | 5.01080800 | -2.68431200 | 4.12811900 |
| C | 6.21912700 | -2.36207400 | 3.46920900 |
| C | 6.21339800 | -1.64688500 | 2.27765900 |
| C | 4.60600400 | -0.50320100 | 0.56410800 |
| C | 3.22085800 | -0.42061700 | 0.54024500 |
| H | 2.85319500 | -2.54925700 | 4.13270800 |
| H | 5.04399000 | -3.24111600 | 5.06828400 |
| H | 7.16866400 | -2.67862400 | 3.90863800 |
| H | 7.15041900 | -1.39692100 | 1.77299400 |
| H | 5.27690600 | -0.08703600 | -0.18301900 |
| N | 2.71459100 | -1.09356500 | 1.65578900 |
| C | 1.32034500 | -1.15289000 | 2.04555100 |
| H | 0.70337600 | -1.40647500 | 1.17347700 |
| H | 1.18715400 | -1.94701300 | 2.79127800 |
| H | 0.97730300 | -0.20100300 | 2.48591700 |
| N | 1.50689200 | -2.36959700 | -0.99953800 |
| C | 0.60150900 | -0.58284500 | -2.31152800 |

| | | | |
|---|---|---|---|
| O | 0.88646700 | 0.14819200 | -3.40871100 |
| C | -0.11018100 | 1.01475100 | -3.93096900 |
| H | 0.29953700 | 1.42191000 | -4.86434700 |
| H | -1.04398800 | 0.47084500 | -4.14518900 |
| H | -0.30162000 | 1.83705100 | -3.22403300 |
| O | -0.52919900 | -0.60090900 | -1.82340000 |
| H | 3.40409700 | -0.35116200 | -2.91621000 |
| H | 2.58815100 | -1.61717700 | -3.79971200 |
| C | -2.58450100 | 4.64714400 | -1.15243900 |
| H | -2.75358100 | 5.48839700 | -1.84305500 |
| H | -3.03251200 | 4.90936500 | -0.17995700 |
| H | -1.50026600 | 4.54035300 | -1.01119600 |
| H | -2.64996100 | 3.09728600 | -2.63878100 |
| S | -2.71155400 | 2.48238600 | 2.40396800 |

# TS-(1S,2S,3R,4R)

| | | | |
|---|---|---|---|
| C | -3.08500900 | 0.78779600 | -3.65414000 |
| C | -2.90101300 | 0.96504600 | -2.11373400 |
| C | -3.05102900 | -0.39766000 | -1.39346100 |
| H | -2.09679600 | -0.95953600 | -1.44266800 |
| H | -3.82198100 | -1.01770700 | -1.87503100 |
| P | -3.55352500 | -0.34486400 | 0.36244200 |
| C | -5.24820000 | 0.31944200 | 0.47496000 |
| C | -6.34962000 | -0.55588900 | 0.43266900 |
| C | -5.46692600 | 1.70851600 | 0.49758900 |
| C | -7.64959900 | -0.04638800 | 0.43462200 |
| H | -6.19746900 | -1.63630800 | 0.40274800 |
| C | -6.77229500 | 2.20808800 | 0.49962200 |
| H | -4.62866500 | 2.41008800 | 0.47252700 |
| C | -7.86287900 | 1.33505100 | 0.47342600 |
| H | -8.49847100 | -0.73364200 | 0.40578000 |
| H | -6.93136800 | 3.28882100 | 0.51412000 |
| H | -8.88150500 | 1.73092500 | 0.47691200 |
| C | -3.54728800 | -2.03232500 | 1.02918300 |
| C | -4.21614600 | -2.30431800 | 2.23964300 |
| C | -2.82606500 | -3.05041200 | 0.38109700 |
| C | -4.17881200 | -3.58937800 | 2.77992700 |
| H | -4.76449800 | -1.51767700 | 2.76154400 |
| C | -2.79686800 | -4.33525900 | 0.93402200 |
| H | -2.25639500 | -2.85095700 | -0.53109600 |
| C | -3.47294300 | -4.60584900 | 2.12562700 |

| | | | |
|---|---|---|---|
| H | -4.69882700 | -3.79575800 | 3.71835200 |
| H | -2.22989600 | -5.11766000 | 0.42494200 |
| H | -3.44532100 | -5.61154500 | 2.55273800 |
| N | -1.61214100 | 1.55021300 | -1.77716100 |
| H | -0.78662500 | 1.04312900 | -2.11806900 |
| H | -3.66131800 | 1.68446100 | -1.77353100 |
| C | -1.40286800 | 2.84821700 | -1.38894500 |
| C | -2.36476500 | 0.69929800 | 1.30947000 |
| C | -2.46635700 | 0.61545900 | 2.81441200 |
| C | -3.33821400 | 1.45338600 | 3.52973600 |
| C | -1.66578000 | -0.29299100 | 3.52730600 |
| C | -3.41432100 | 1.37688300 | 4.92297000 |
| C | -1.73740900 | -0.36442800 | 4.92082400 |
| C | -2.61524500 | 0.46708200 | 5.62222400 |
| H | -2.48646600 | 1.73177000 | 0.93759000 |
| H | -1.38281300 | 0.34714400 | 0.95796200 |
| H | -0.97077700 | -0.94271300 | 2.98872400 |
| H | -1.09762700 | -1.06823800 | 5.45856600 |
| H | -2.66874100 | 0.41421200 | 6.71248700 |
| H | -4.09284900 | 2.04056400 | 5.46483700 |
| H | -3.95204200 | 2.18196300 | 2.99516300 |
| C | -4.54562400 | 0.47216200 | -4.00777300 |
| H | -4.65288500 | 0.30920700 | -5.09153200 |
| H | -4.92793700 | -0.43172100 | -3.50817200 |
| H | -5.20538100 | 1.31193700 | -3.73139300 |
| N | -0.09849600 | 3.21584300 | -1.48997800 |
| H | 0.52284000 | 2.47125400 | -1.83633500 |
| C | 0.60110800 | 4.40611400 | -1.19043100 |
| C | 2.00798300 | 4.30034200 | -1.21018600 |
| C | 0.01594700 | 5.65612500 | -0.92543300 |
| C | 2.81082600 | 5.41305300 | -0.96812500 |
| H | 2.47088000 | 3.32835800 | -1.40912300 |
| C | 0.82407400 | 6.76902100 | -0.67625900 |
| H | -1.06670100 | 5.75339200 | -0.91360400 |
| C | 2.20841500 | 6.64033000 | -0.69924600 |
| H | 3.89988500 | 5.33785600 | -0.98273700 |
| H | 0.38058300 | 7.74509600 | -0.46871000 |
| F | 2.97886000 | 7.71579500 | -0.46064500 |
| C | -2.61318600 | 2.02712600 | -4.42665000 |
| H | -2.80461500 | 1.89959600 | -5.50384700 |
| H | -3.14627600 | 2.93141800 | -4.09092100 |
| H | -1.53646000 | 2.20747000 | -4.29919100 |

| | | | |
|---|---|---|---|
| H | -2.45884600 | -0.06956800 | -3.96455200 |
| C | 4.72278000 | 0.87665200 | -0.54300200 |
| C | 1.54926400 | -2.55288200 | -0.52189600 |
| C | 1.83537200 | -1.19961600 | -0.15876500 |
| C | 3.03441600 | -0.30407300 | -1.66611100 |
| C | 3.99825000 | -1.38913200 | -2.11958200 |
| H | 2.23849000 | -3.33618100 | -0.20991000 |
| H | 0.99298300 | -0.52580400 | -0.32781700 |
| C | 6.85577700 | -0.29919000 | 0.14890300 |
| C | 5.63997700 | -0.28549100 | -0.56033700 |
| C | 5.27100900 | -1.42054500 | -1.31361300 |
| C | 6.11617100 | -2.53477600 | -1.33756000 |
| C | 7.31986200 | -2.54014300 | -0.62673200 |
| C | 7.69043000 | -1.41674200 | 0.11845400 |
| H | 7.14962000 | 0.56664800 | 0.74410400 |
| H | 5.82423100 | -3.41219800 | -1.92250200 |
| H | 7.96720200 | -3.42078300 | -0.65412800 |
| H | 8.62816500 | -1.41217200 | 0.67993800 |
| C | 5.17207000 | 2.14565600 | 0.13609200 |
| H | 6.12681500 | 2.51332100 | -0.27631500 |
| H | 5.32902700 | 1.98397500 | 1.21673700 |
| H | 4.40897000 | 2.92363000 | 0.01293100 |
| C | 0.39180800 | -2.90553400 | -1.23675200 |
| O | -0.48466500 | -2.07320600 | -1.64341700 |
| C | 0.13647200 | -4.36758900 | -1.53547600 |
| C | -0.77600400 | -4.69139300 | -2.55655100 |
| C | 0.73763100 | -5.42812000 | -0.83045500 |
| C | -1.06414500 | -6.01981500 | -2.87738000 |
| H | -1.25184800 | -3.86905500 | -3.09366100 |
| C | 0.44481900 | -6.75789700 | -1.14284900 |
| H | 1.42762500 | -5.21447500 | -0.01228900 |
| C | -0.45478600 | -7.06177700 | -2.17102900 |
| H | -1.76805700 | -6.24493000 | -3.68377900 |
| H | 0.92035500 | -7.56361700 | -0.57660300 |
| H | -0.68005000 | -8.10294200 | -2.41699400 |
| C | 3.85584200 | -1.08921500 | 2.96080200 |
| C | 3.20381900 | 0.18098000 | 2.94574700 |
| C | 3.37606900 | 1.11435900 | 3.98022300 |
| C | 4.22187500 | 0.76814600 | 5.03250700 |
| C | 4.88259100 | -0.48049900 | 5.06087900 |
| C | 4.70745500 | -1.40740900 | 4.03852600 |
| C | 3.43714400 | -1.77174100 | 1.78016900 |

| | | | |
|---|---|---|---|
| C | 2.57209200 | -0.93903200 | 1.08563500 |
| H | 2.86574300 | 2.07978100 | 3.96925400 |
| H | 4.37631600 | 1.47631000 | 5.85086600 |
| H | 5.54044300 | -0.71771600 | 5.90116800 |
| H | 5.22193800 | -2.37167800 | 4.06712000 |
| H | 3.74371600 | -2.76513600 | 1.46344100 |
| N | 2.44182200 | 0.25816900 | 1.79920500 |
| C | 1.60658100 | 1.39328100 | 1.47237200 |
| H | 0.69365600 | 1.41803200 | 2.09394800 |
| H | 1.33522800 | 1.36982000 | 0.41296300 |
| H | 2.15996200 | 2.32888300 | 1.64062700 |
| N | 3.53947400 | 0.85216400 | -1.07087000 |
| C | 1.91222300 | -0.04264700 | -2.58254300 |
| O | 1.81124400 | -0.97865400 | -3.53437600 |
| C | 0.66956900 | -0.95640100 | -4.38484700 |
| H | 0.87278600 | -1.68072200 | -5.18410900 |
| H | 0.51336200 | 0.04564100 | -4.81108700 |
| H | -0.21221300 | -1.27059000 | -3.80448000 |
| O | 1.10146900 | 0.88646300 | -2.50933500 |
| H | 3.48736000 | -2.36765700 | -2.08879000 |
| H | 4.25437200 | -1.23914700 | -3.18621900 |
| S | -2.66216700 | 3.82787900 | -0.80435800 |

## TS-(1R,2R,3R,4R)

| | | | |
|---|---|---|---|
| C | 2.28628300 | -3.03218200 | -2.44828400 |
| C | 2.59591600 | -1.97202700 | -1.34794800 |
| C | 2.61623800 | -0.54791600 | -1.94191500 |
| H | 1.57472400 | -0.28146400 | -2.19182300 |
| H | 3.21388700 | -0.50527500 | -2.86398800 |
| P | 3.34040100 | 0.77932600 | -0.91337100 |
| C | 5.15134600 | 0.61233800 | -1.06547800 |
| C | 5.86621100 | 1.29582400 | -2.06510500 |
| C | 5.81698300 | -0.32914700 | -0.25813700 |
| C | 7.23368000 | 1.06400300 | -2.23194400 |
| H | 5.36129400 | 2.00921200 | -2.71879300 |
| C | 7.18439200 | -0.55625800 | -0.43547600 |
| H | 5.27378100 | -0.91498200 | 0.48937700 |
| C | 7.89533000 | 0.14288300 | -1.41477000 |
| H | 7.78180000 | 1.60454400 | -3.00731000 |
| H | 7.69122100 | -1.29113000 | 0.19429700 |
| H | 8.96507200 | -0.03659600 | -1.54771200 |

| | | | |
|---|---|---|---|
| C | 2.78653000 | 2.37307200 | -1.59341200 |
| C | 3.54725600 | 3.54072600 | -1.39256200 |
| C | 1.53391700 | 2.45576800 | -2.23158300 |
| C | 3.07781500 | 4.76955300 | -1.85913000 |
| H | 4.50305900 | 3.49717300 | -0.86752500 |
| C | 1.07448400 | 3.69408600 | -2.69170300 |
| H | 0.89518600 | 1.57479200 | -2.35265600 |
| C | 1.84468000 | 4.84662000 | -2.51483900 |
| H | 3.67621600 | 5.67032000 | -1.70362300 |
| H | 0.10333500 | 3.74991700 | -3.18897900 |
| H | 1.48033800 | 5.80964100 | -2.88149400 |
| N | 1.61855700 | -1.99925400 | -0.27022300 |
| H | 0.68663000 | -1.63740600 | -0.50709800 |
| H | 3.56396300 | -2.23500200 | -0.89475600 |
| C | 1.84318200 | -2.46779600 | 0.99363800 |
| C | 2.85624300 | 0.69910500 | 0.86288400 |
| C | 3.31407200 | 1.88226100 | 1.68583100 |
| C | 4.56701800 | 1.88883300 | 2.32151500 |
| C | 2.47167900 | 2.99421800 | 1.85370300 |
| C | 2.87395700 | 4.08619700 | 2.62654800 |
| C | 4.12693300 | 4.08505200 | 3.24692300 |
| H | 3.25790200 | -0.25110700 | 1.25306600 |
| H | 1.75800500 | 0.60645100 | 0.87542000 |
| H | 1.48624200 | 2.99227000 | 1.38386800 |
| H | 2.20244900 | 4.93978300 | 2.74985100 |
| H | 4.44069900 | 4.93722300 | 3.85508800 |
| H | 5.22855500 | 1.02525800 | 2.22485700 |
| C | 3.34391200 | -3.03616000 | -3.56217400 |
| H | 3.13668000 | -3.84582500 | -4.27909600 |
| H | 3.36976200 | -2.10028100 | -4.14155800 |
| H | 4.35371300 | -3.21080400 | -3.15181400 |
| N | 0.71955100 | -2.49512600 | 1.75198000 |
| H | -0.14138400 | -2.16508000 | 1.26904900 |
| C | 0.50983600 | -2.83399600 | 3.10575400 |
| C | -0.79709800 | -3.22493300 | 3.45998600 |
| C | 1.48635700 | -2.76285000 | 4.11553400 |
| C | -1.12512800 | -3.54069000 | 4.77797500 |
| H | -1.56479300 | -3.28136800 | 2.68620500 |
| C | 1.16220800 | -3.08688600 | 5.43481600 |
| H | 2.50033100 | -2.46439000 | 3.86172400 |
| C | -0.13574700 | -3.47286900 | 5.75508900 |
| H | -2.13665900 | -3.84594900 | 5.05272000 |

| | | | |
|---|---|---|---|
| H | 1.91541400 | -3.03735200 | 6.22400800 |
| F | -0.44043900 | -3.78316200 | 7.02747200 |
| C | -2.53113600 | -2.45270700 | -0.17844900 |
| C | -2.62754200 | 0.10373400 | -1.82284400 |
| C | -2.41014200 | 0.86256200 | -0.63463400 |
| C | -2.39543900 | -0.44932600 | 1.02034600 |
| C | -3.82756600 | -0.57986500 | 1.51416000 |
| H | -3.62918900 | 0.03907900 | -2.24392700 |
| H | -1.35322100 | 1.09805200 | -0.49639700 |
| C | -4.77922600 | -3.20844100 | -1.06878400 |
| C | -4.00724800 | -2.36654600 | -0.24571800 |
| C | -4.66016000 | -1.43401900 | 0.58977300 |
| C | -6.05571700 | -1.35542500 | 0.57444700 |
| C | -6.81461600 | -2.18841200 | -0.25494200 |
| C | -6.17251400 | -3.11799300 | -1.07792500 |
| H | -4.28850700 | -3.93605900 | -1.71716700 |
| H | -6.55518400 | -0.62867100 | 1.22153300 |
| H | -7.90508500 | -2.11241800 | -0.25584300 |
| H | -6.75688400 | -3.77571200 | -1.72652400 |
| C | -1.84606500 | -3.64000700 | -0.80461100 |
| H | -1.98253100 | -3.64480600 | -1.89890400 |
| H | -2.25329600 | -4.59146600 | -0.42287500 |
| H | -0.76940700 | -3.61142800 | -0.60115100 |
| C | -1.54124500 | -0.46189700 | -2.51804000 |
| O | -0.33207900 | -0.35817400 | -2.13018400 |
| C | -1.80267100 | -1.17420900 | -3.82307100 |
| C | -0.74463900 | -1.31694900 | -4.73924100 |
| C | -3.05423800 | -1.71707200 | -4.17123300 |
| C | -0.92910300 | -1.96109300 | -5.96396000 |
| H | 0.22672700 | -0.89977900 | -4.46919400 |
| C | -3.23862100 | -2.37194300 | -5.39175700 |
| H | -3.89116900 | -1.64477100 | -3.47475100 |
| C | -2.17888700 | -2.49412400 | -6.29630600 |
| H | -0.09389700 | -2.04913200 | -6.66467400 |
| H | -4.21799600 | -2.79210300 | -5.63682100 |
| H | -2.32574600 | -3.00369700 | -7.25232300 |
| C | -5.19431100 | 3.25740100 | -0.02753900 |
| C | -4.05300800 | 3.95315200 | 0.47021600 |
| C | -4.14728400 | 5.25136200 | 0.99305100 |
| C | -5.40786500 | 5.84581600 | 1.03365100 |
| C | -6.55124700 | 5.16788500 | 0.55806800 |
| C | -6.45498000 | 3.88431200 | 0.02891500 |

| | | | |
|---|---|---|---|
| C | -4.72275100 | 1.99493900 | -0.50331100 |
| C | -3.35333600 | 1.93926800 | -0.29520200 |
| H | -3.26830700 | 5.78726500 | 1.35805800 |
| H | -5.51166800 | 6.85552500 | 1.43946300 |
| H | -7.52439600 | 5.66365000 | 0.60434300 |
| H | -7.34402900 | 3.36853000 | -0.34390300 |
| H | -5.32147500 | 1.20070300 | -0.94221000 |
| N | -2.95076200 | 3.13463300 | 0.31591800 |
| C | -1.60111500 | 3.57742900 | 0.58091100 |
| H | -0.91889600 | 2.72168500 | 0.62783800 |
| H | -1.56097900 | 4.09550700 | 1.55156900 |
| H | -1.23801700 | 4.27470000 | -0.19567400 |
| N | -1.80651500 | -1.58922600 | 0.47649900 |
| C | -1.46548500 | 0.31052400 | 1.88673600 |
| O | -2.10144100 | 0.93078500 | 2.90698900 |
| C | -1.29208700 | 1.65411200 | 3.82784800 |
| H | -1.97717600 | 2.06045800 | 4.58271500 |
| H | -0.75113400 | 2.47325700 | 3.33073500 |
| H | -0.55238200 | 0.99179800 | 4.30281900 |
| O | -0.26319300 | 0.44092700 | 1.71065300 |
| H | -3.82752700 | -1.03234900 | 2.52702100 |
| H | -4.28794900 | 0.41037700 | 1.63850300 |
| C | 2.16861700 | -4.43764200 | -1.84333100 |
| H | 1.91387900 | -5.16811400 | -2.62750700 |
| H | 3.11514900 | -4.75180600 | -1.37514400 |
| H | 1.38785300 | -4.49218100 | -1.07222100 |
| H | 1.31202200 | -2.75991400 | -2.89171700 |
| S | 3.39164100 | -2.95420500 | 1.51045000 |
| C | 4.97083800 | 2.98124000 | 3.09428800 |
| H | 5.94640200 | 2.96422200 | 3.58679100 |

# 13. NMR spectra

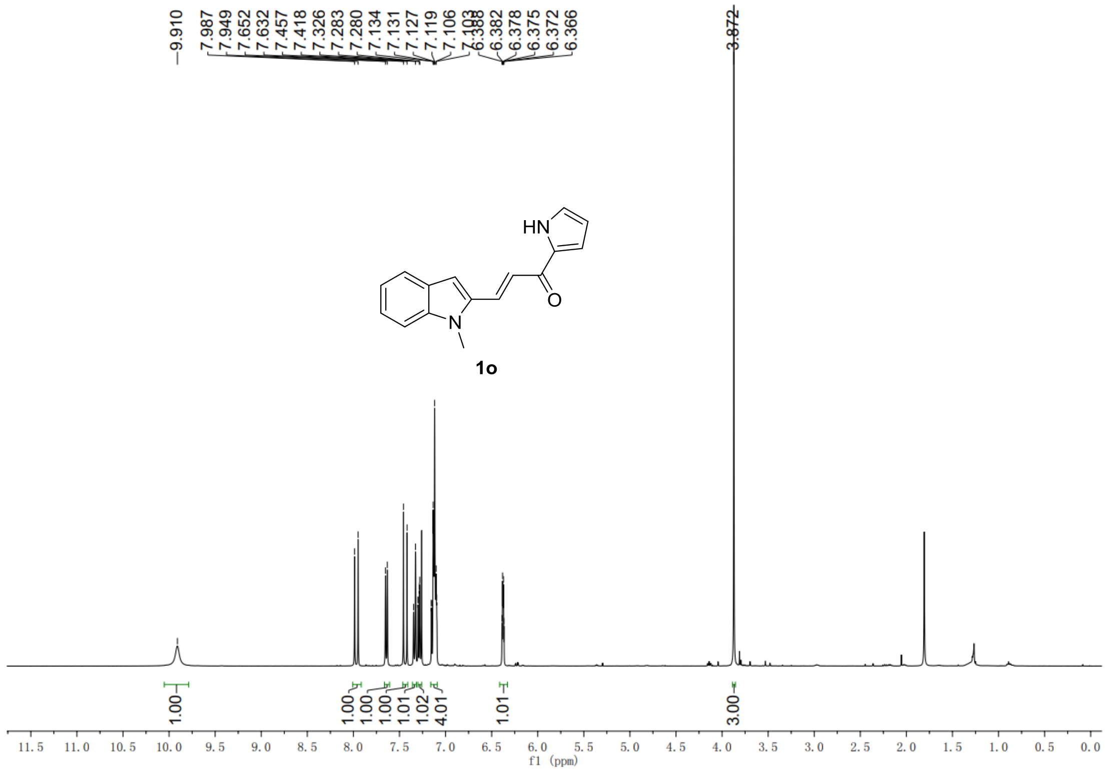

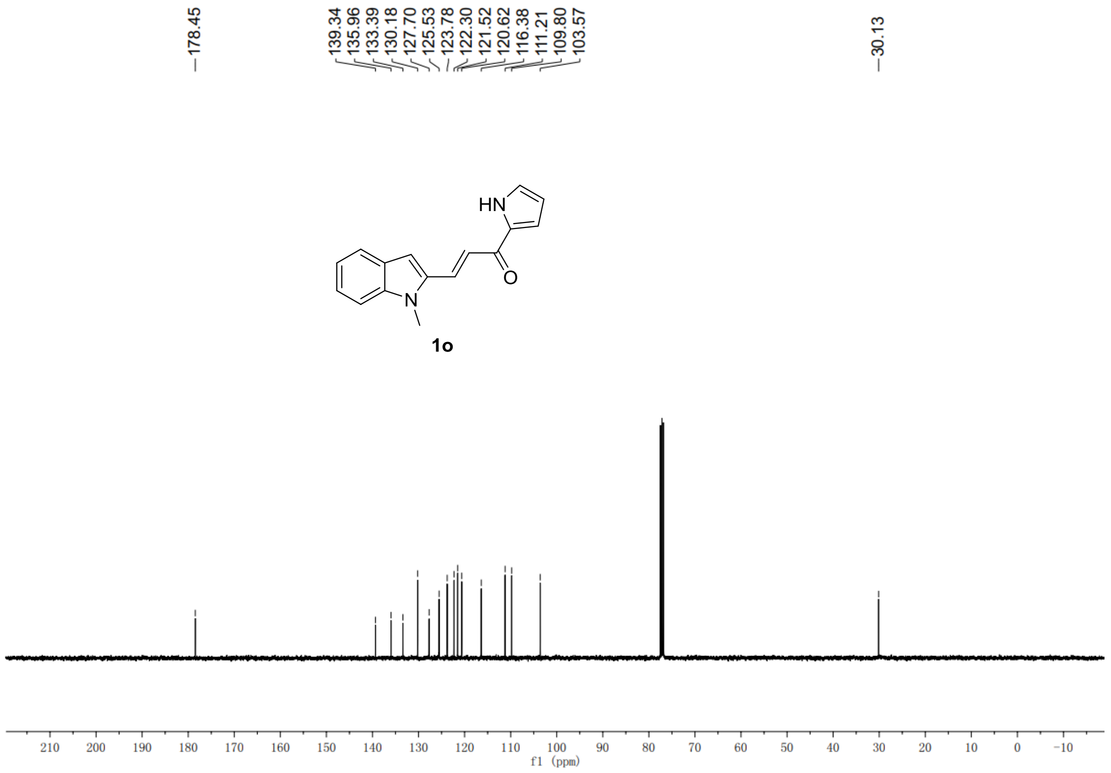

$^1$H-NMR and $^{13}$C-NMR of **1o**

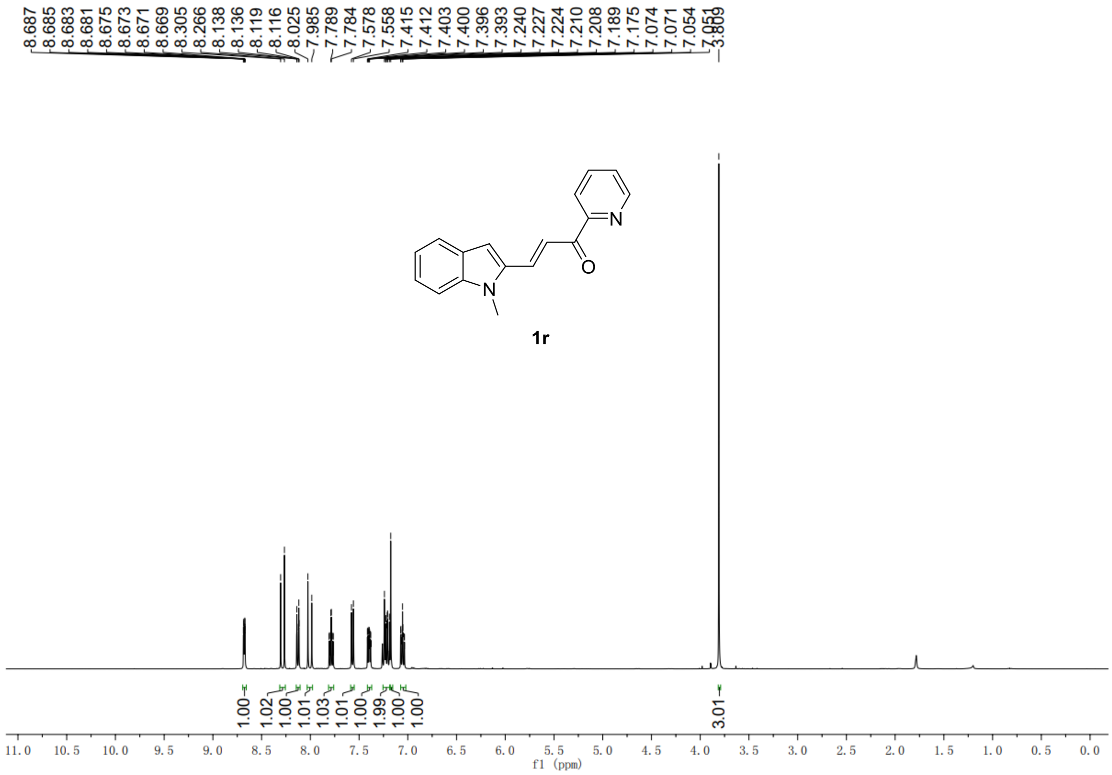


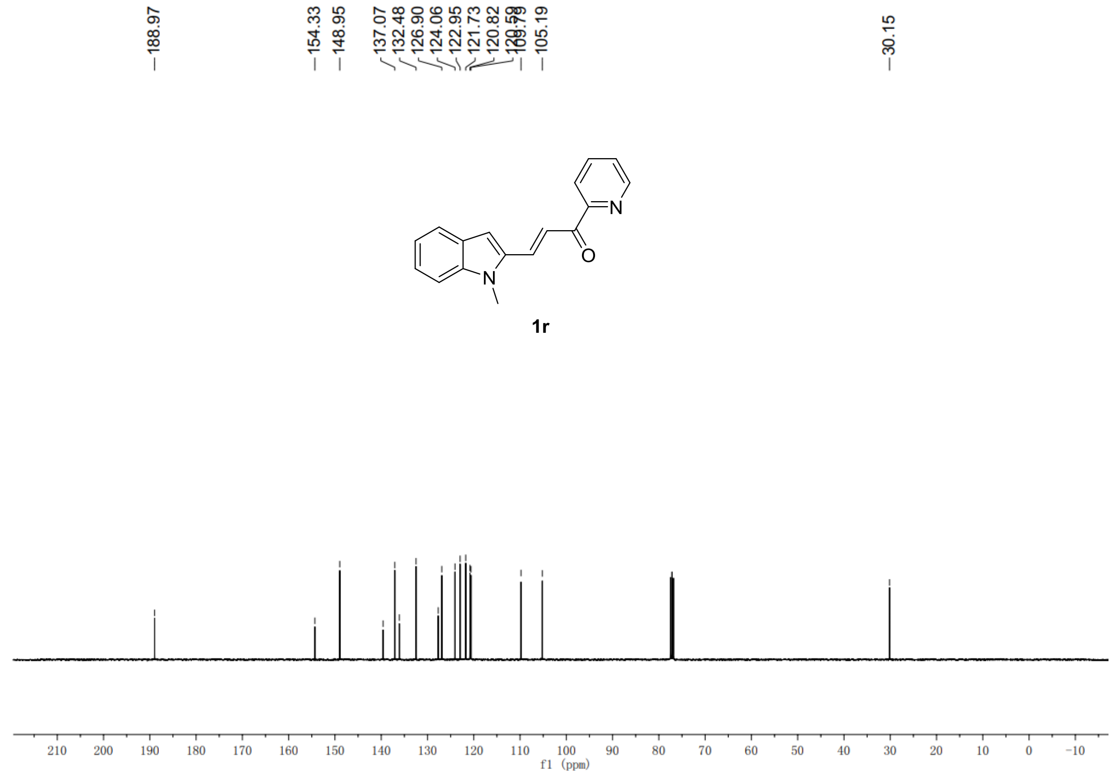


$^{1}$H-NMR and $^{13}$C-NMR of **1r**

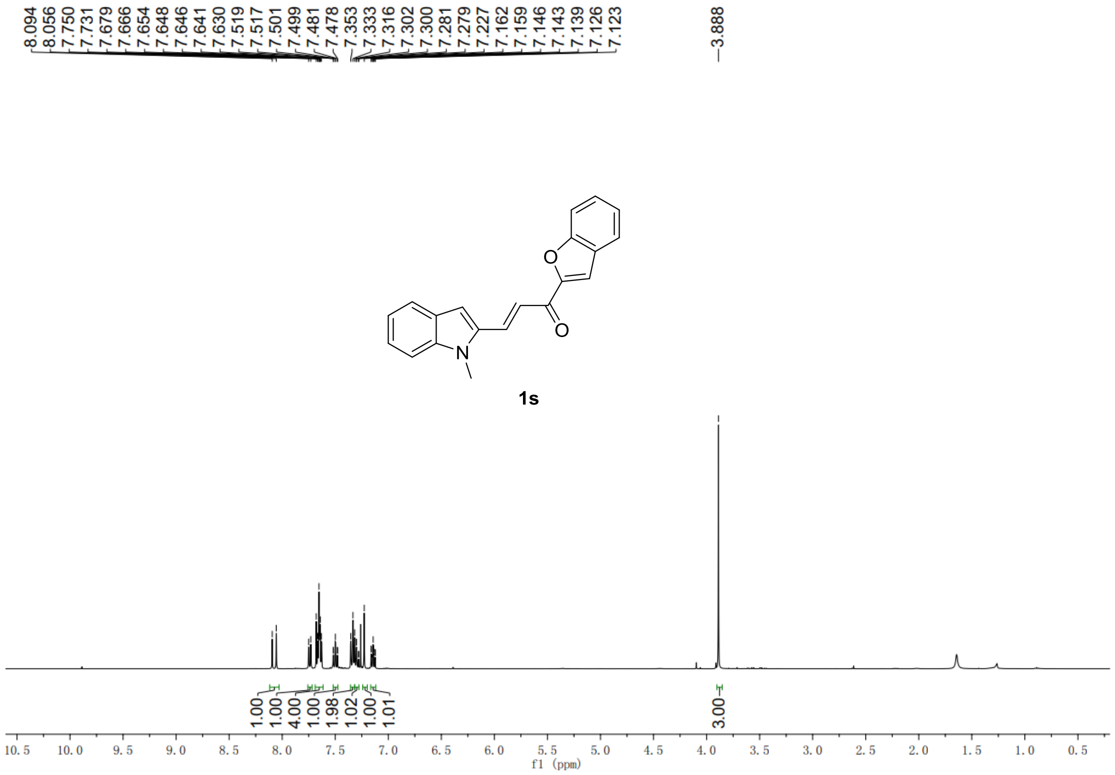


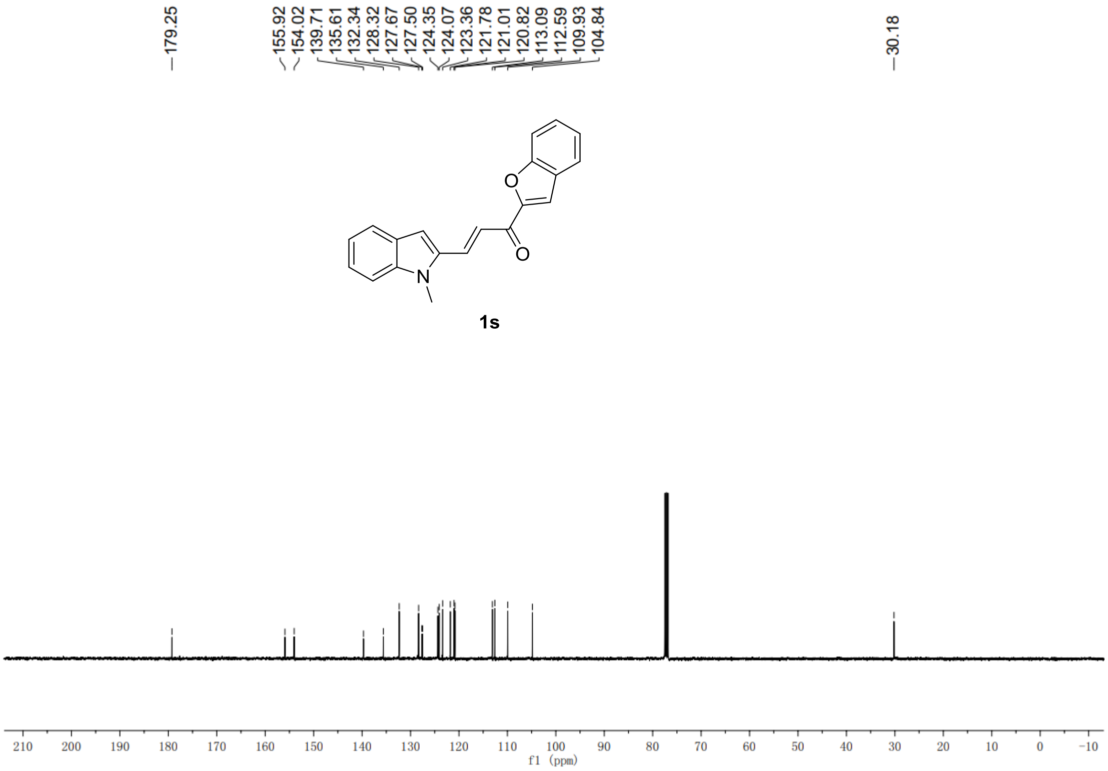


$^{1}$H-NMR and $^{13}$C-NMR of **1s**

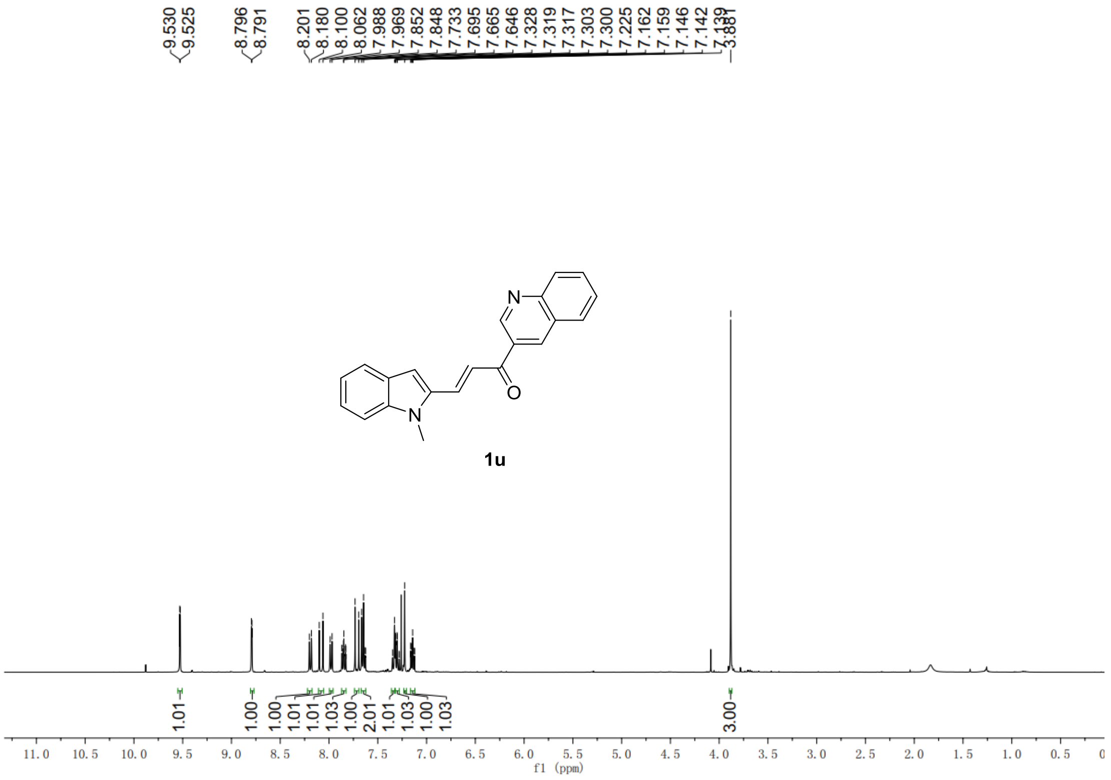


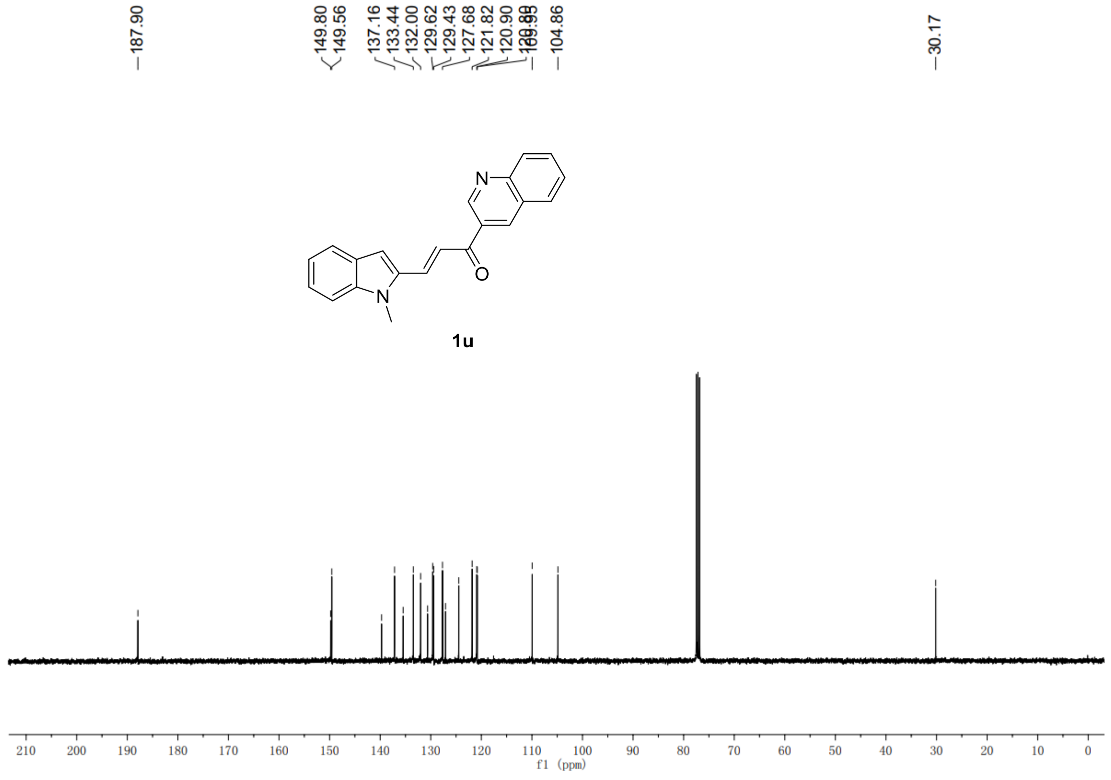


$^{1}$H-NMR and $^{13}$C-NMR of **1u**

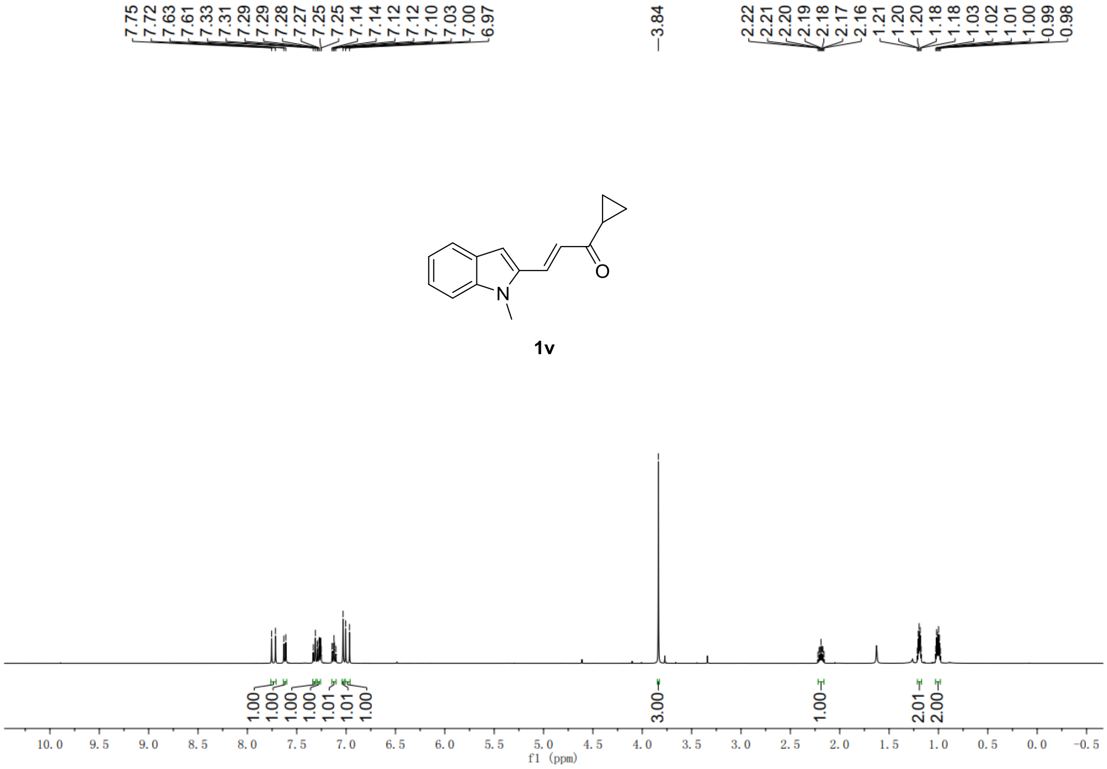


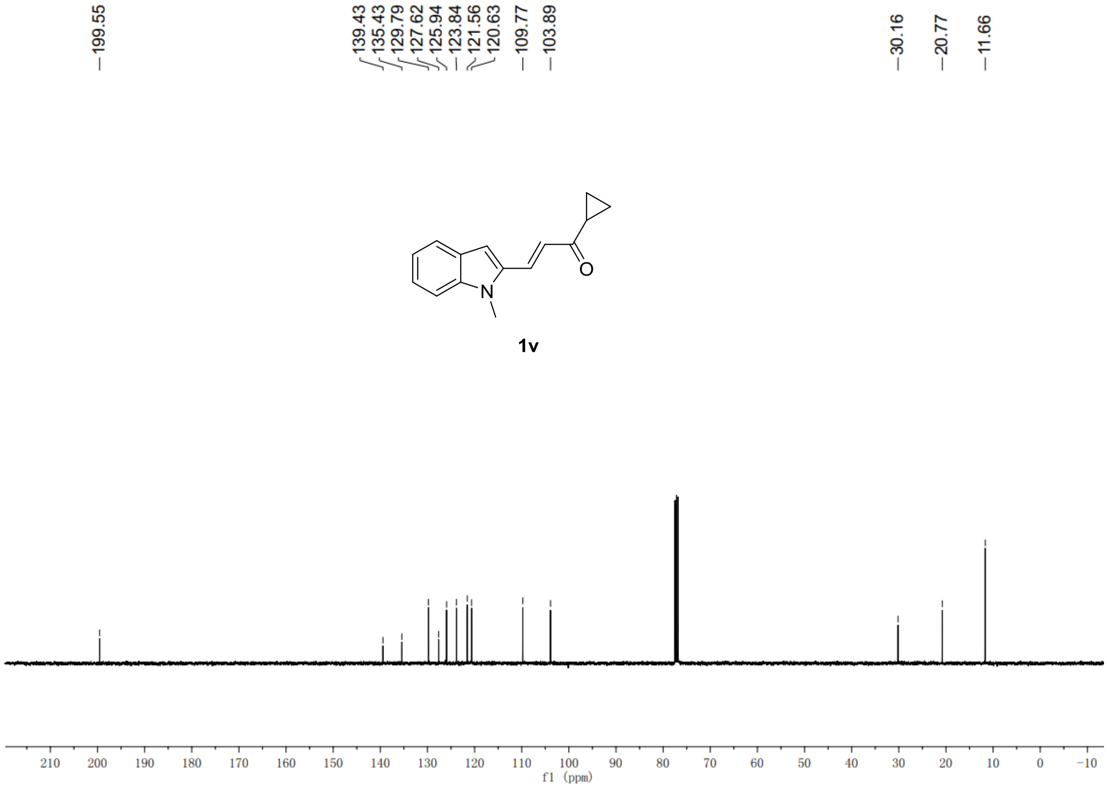


$^1$H-NMR and $^{13}$C-NMR of **1v**

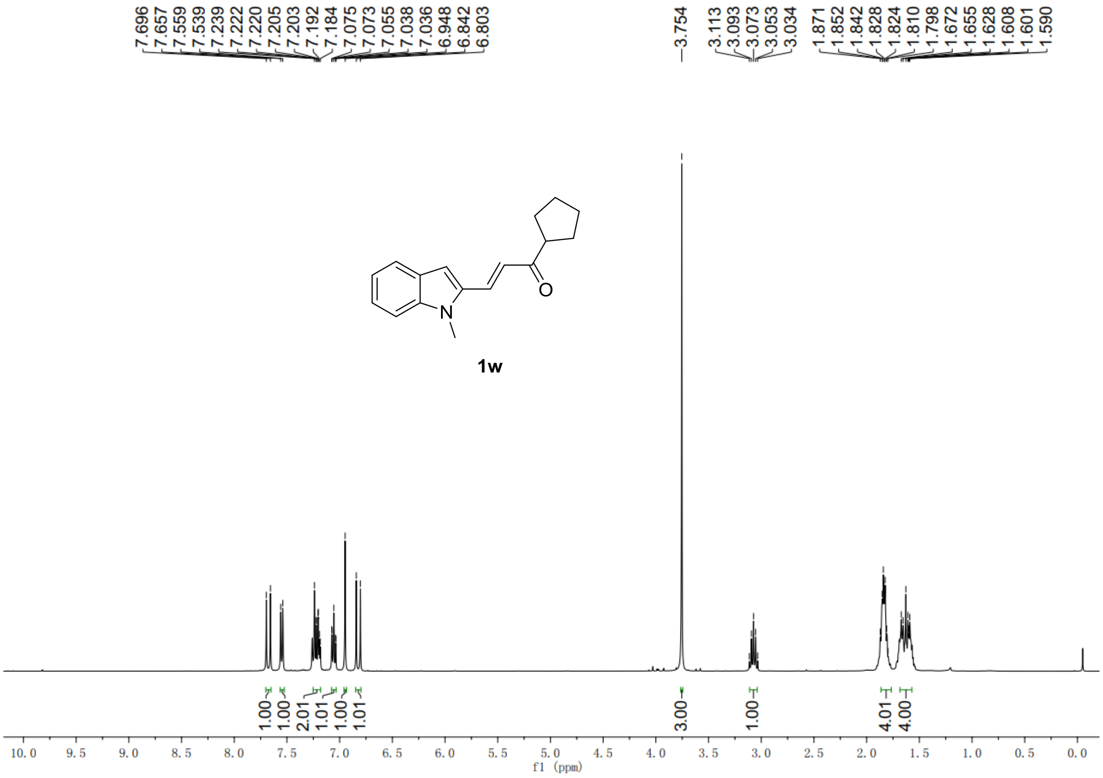

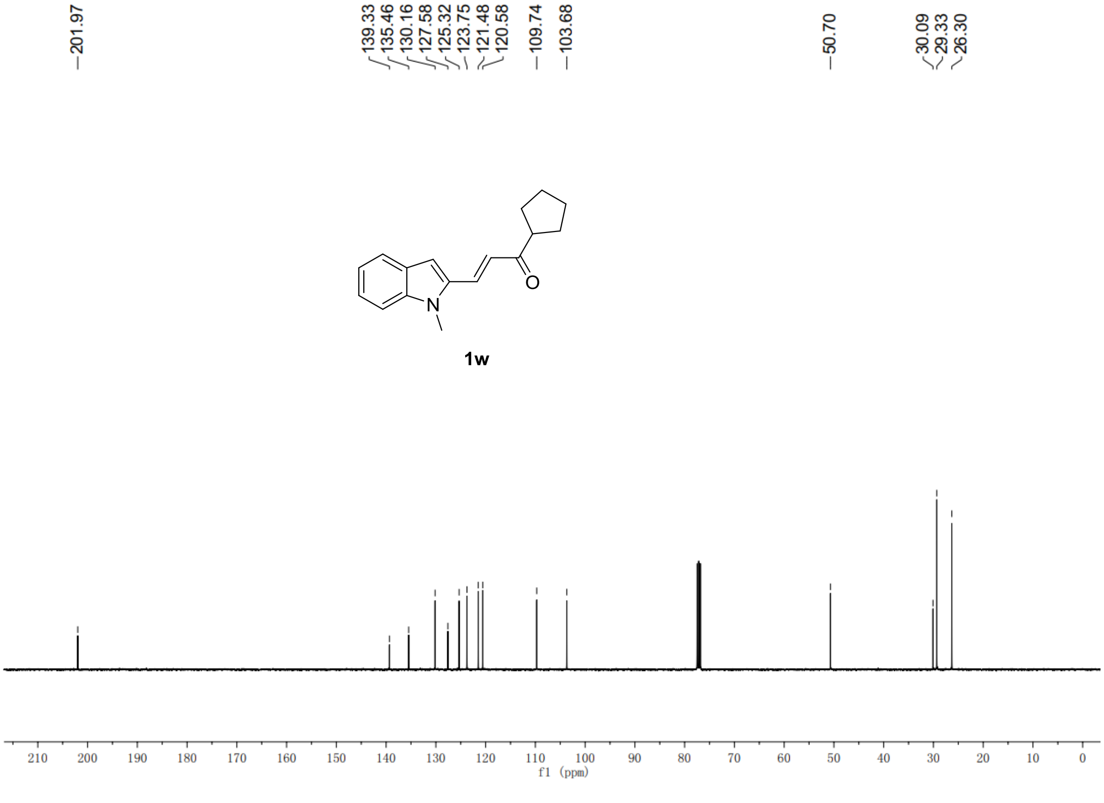

$^{1}$H-NMR and $^{13}$C-NMR of **1w**

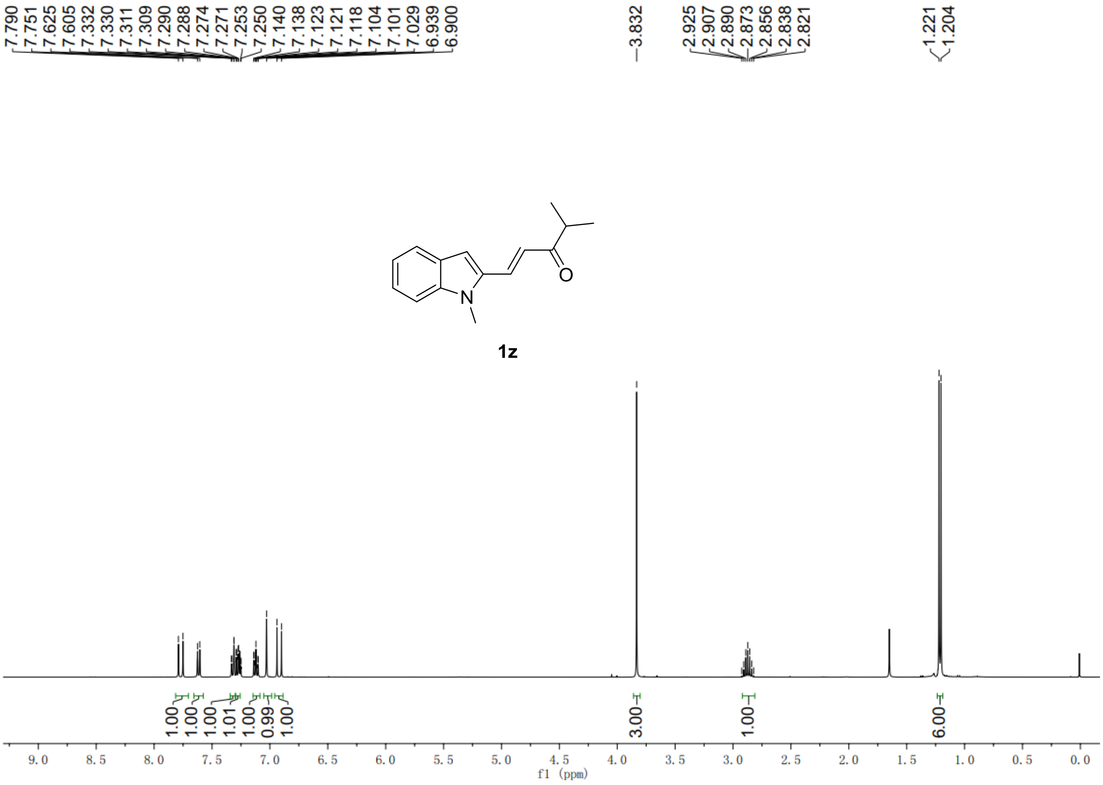

1z

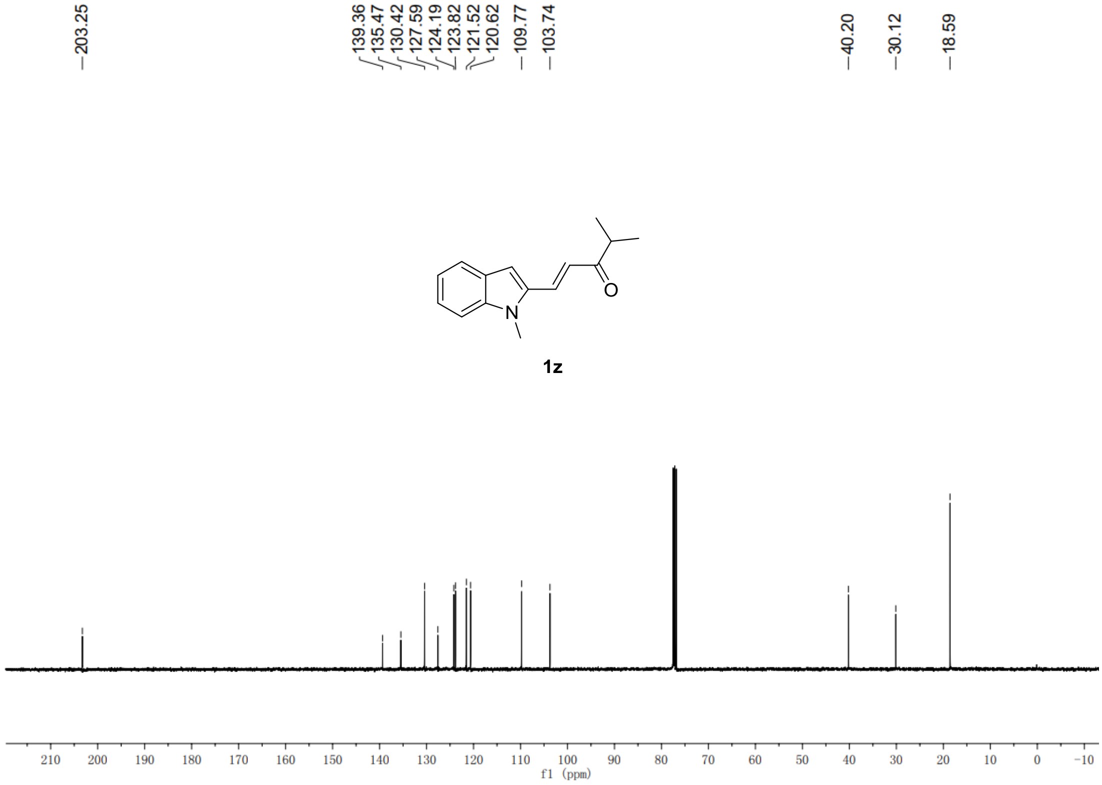

1z

$^{1}H$-NMR and $^{13}C$-NMR of **1z**

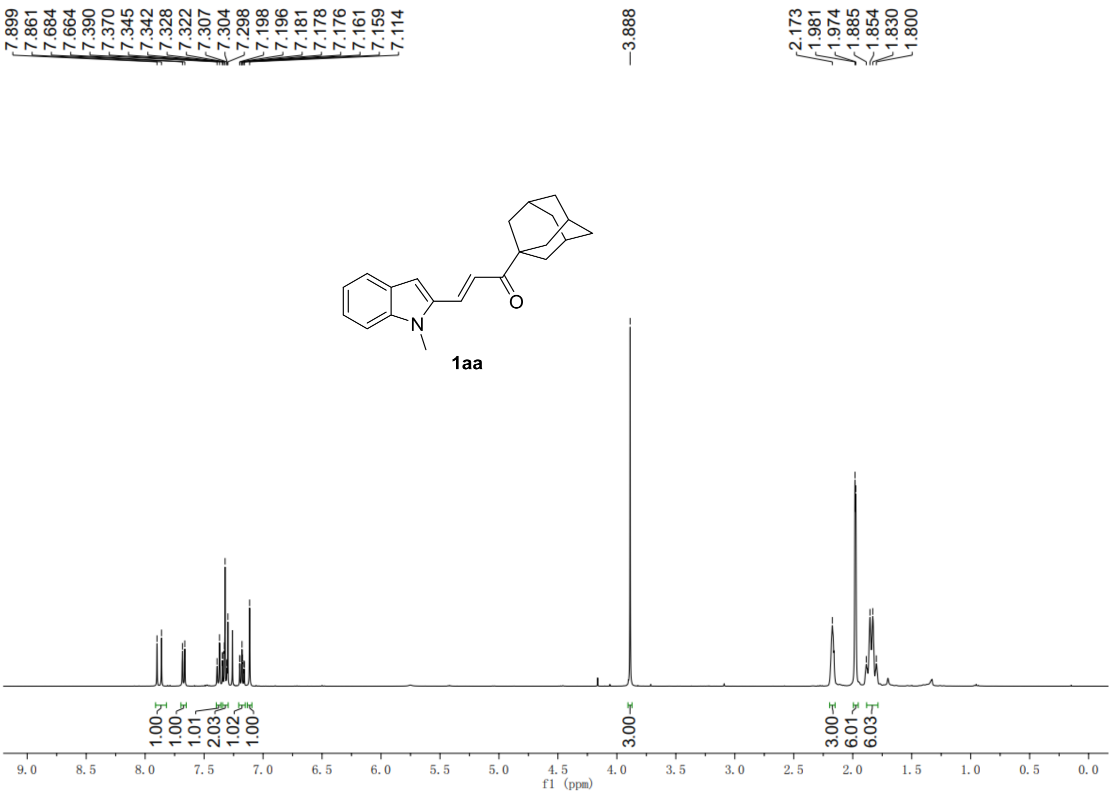


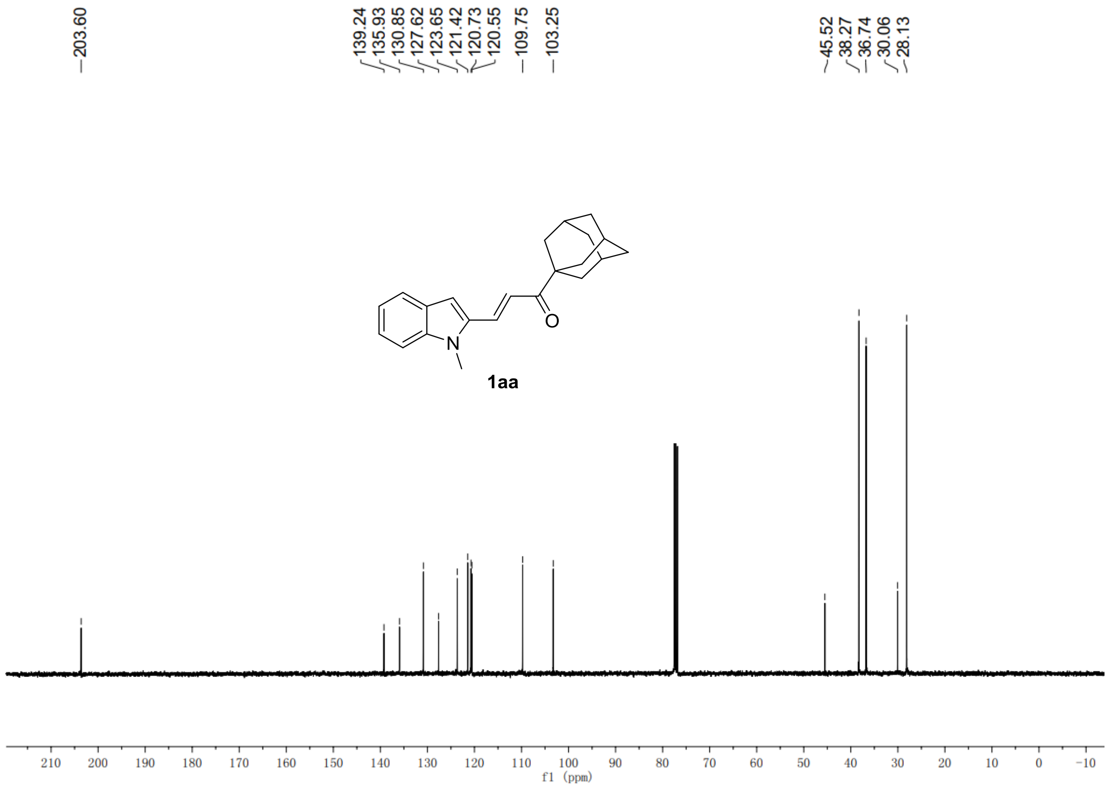


$^{1}$H-NMR and $^{13}$C-NMR of **1aa**

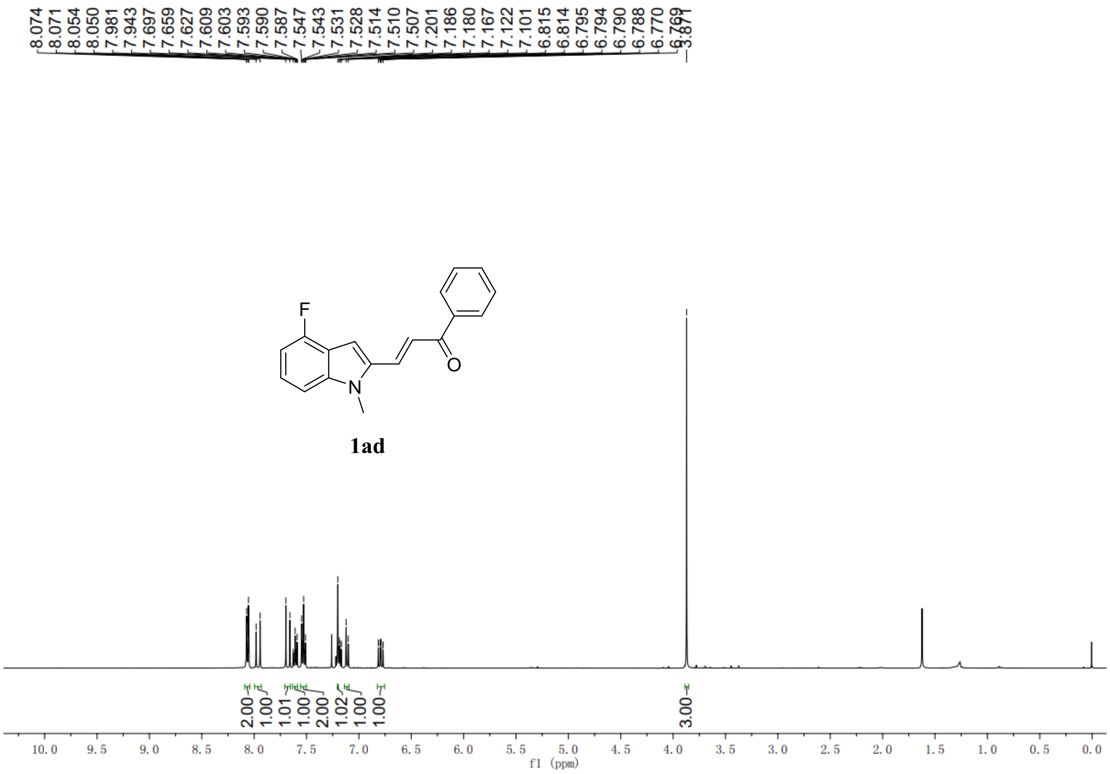


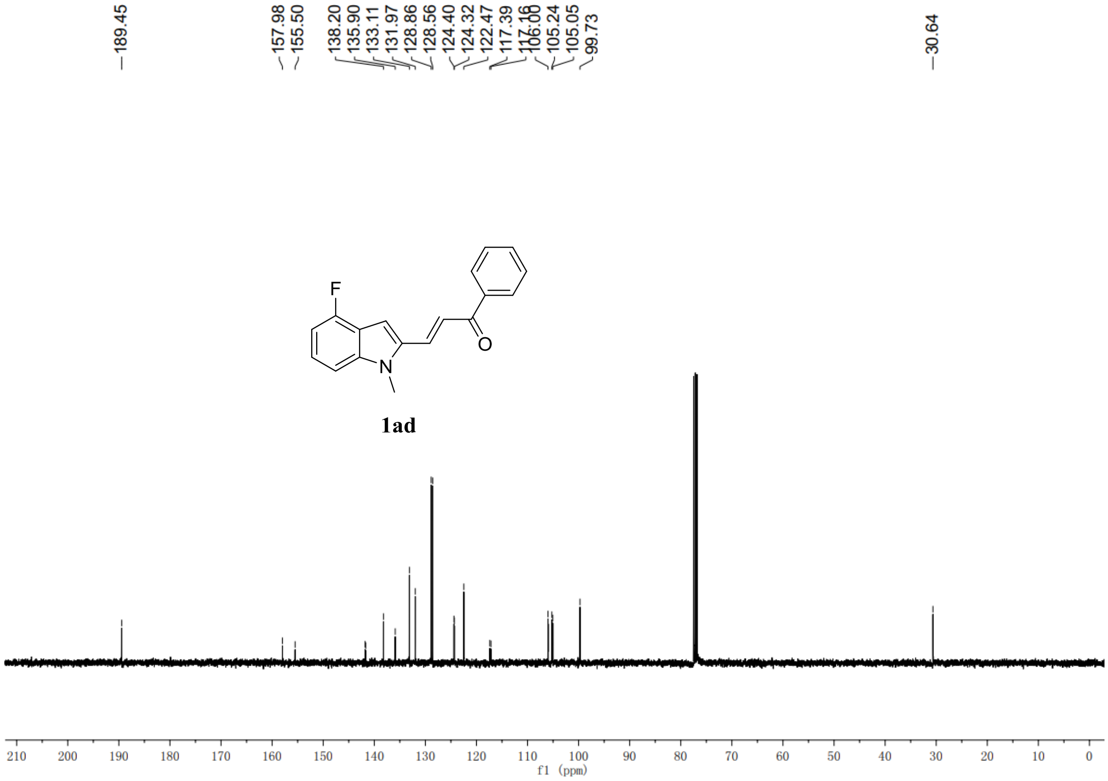


$^{1}$H-NMR and $^{13}$C-NMR of **1ad**

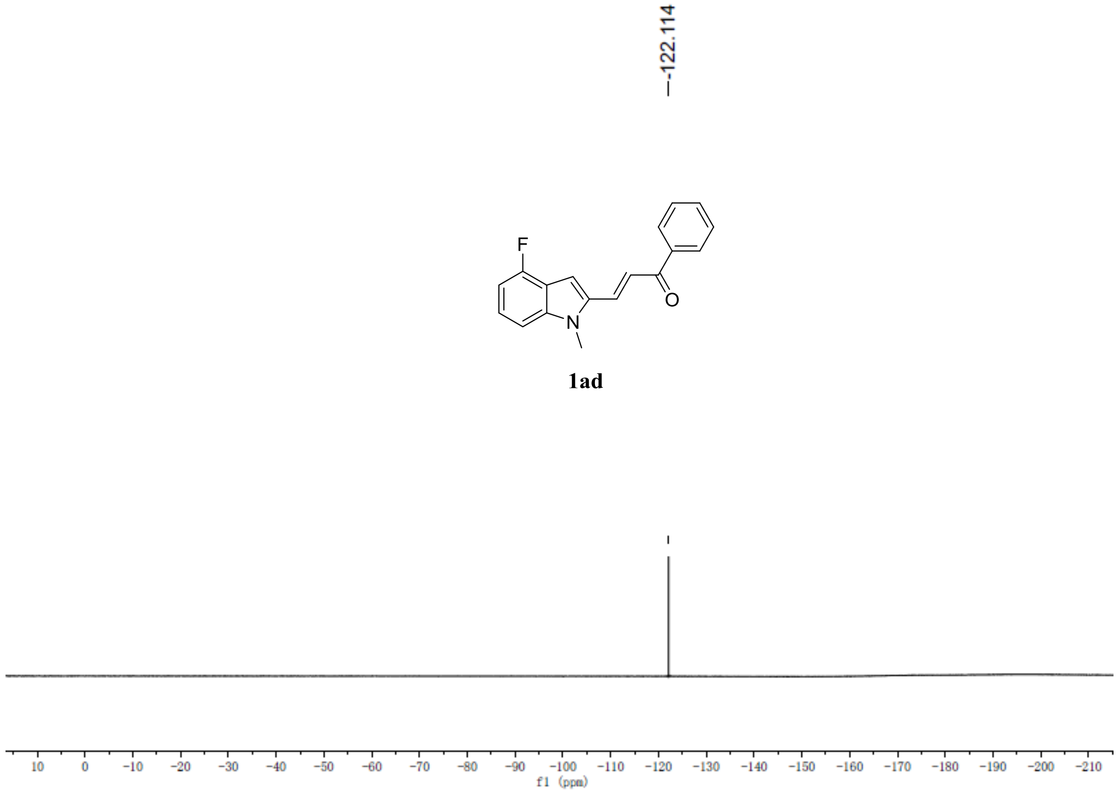


$^{19}$F-NMR of **1ad**

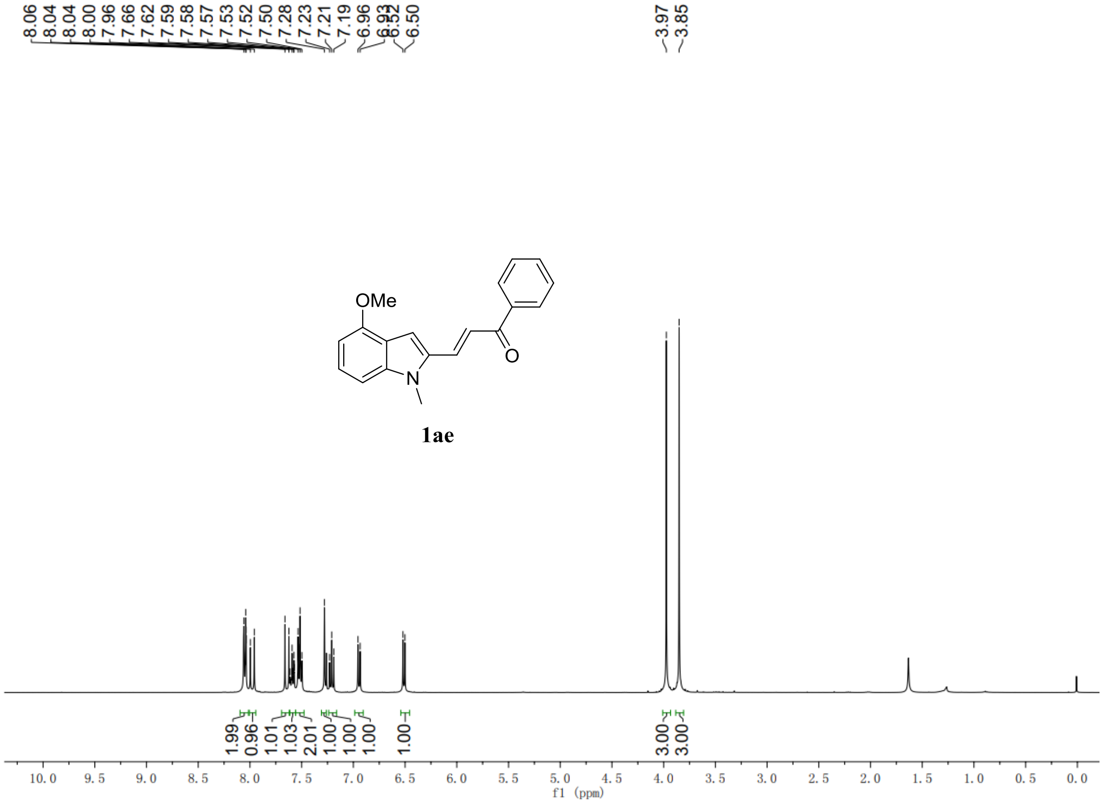

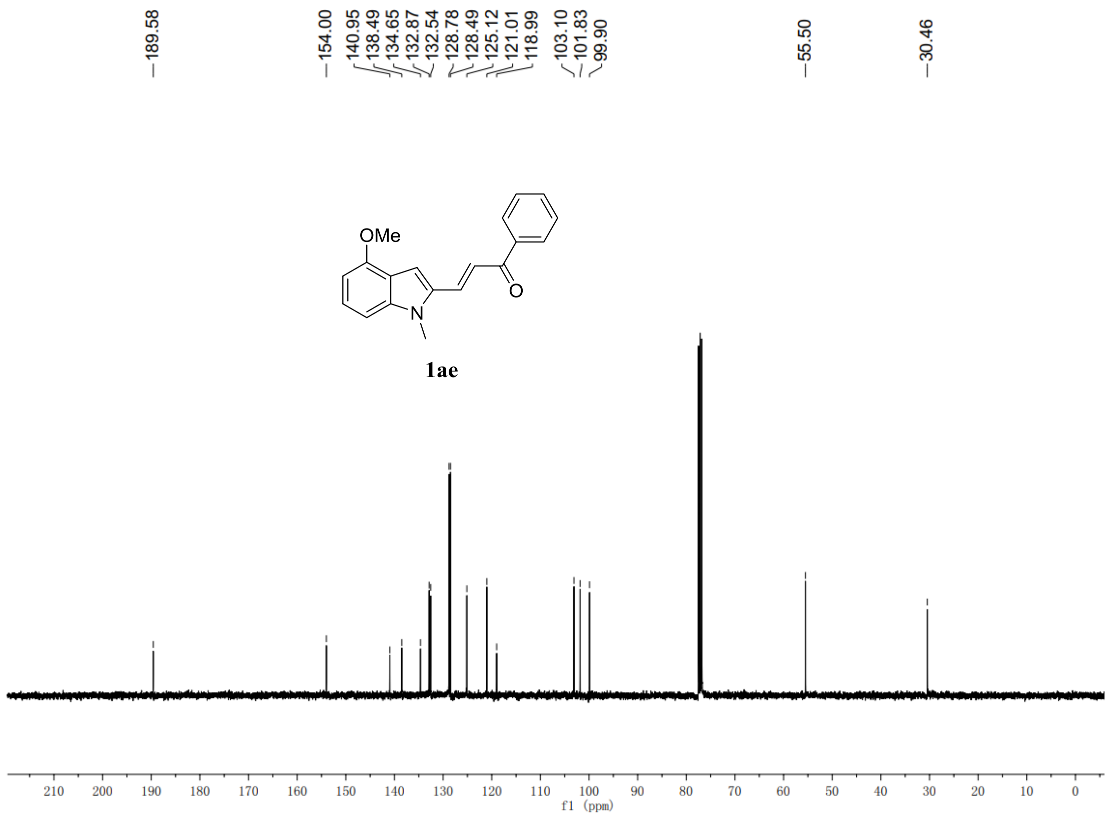


$^{1}$H-NMR and $^{13}$C-NMR of **1ae**

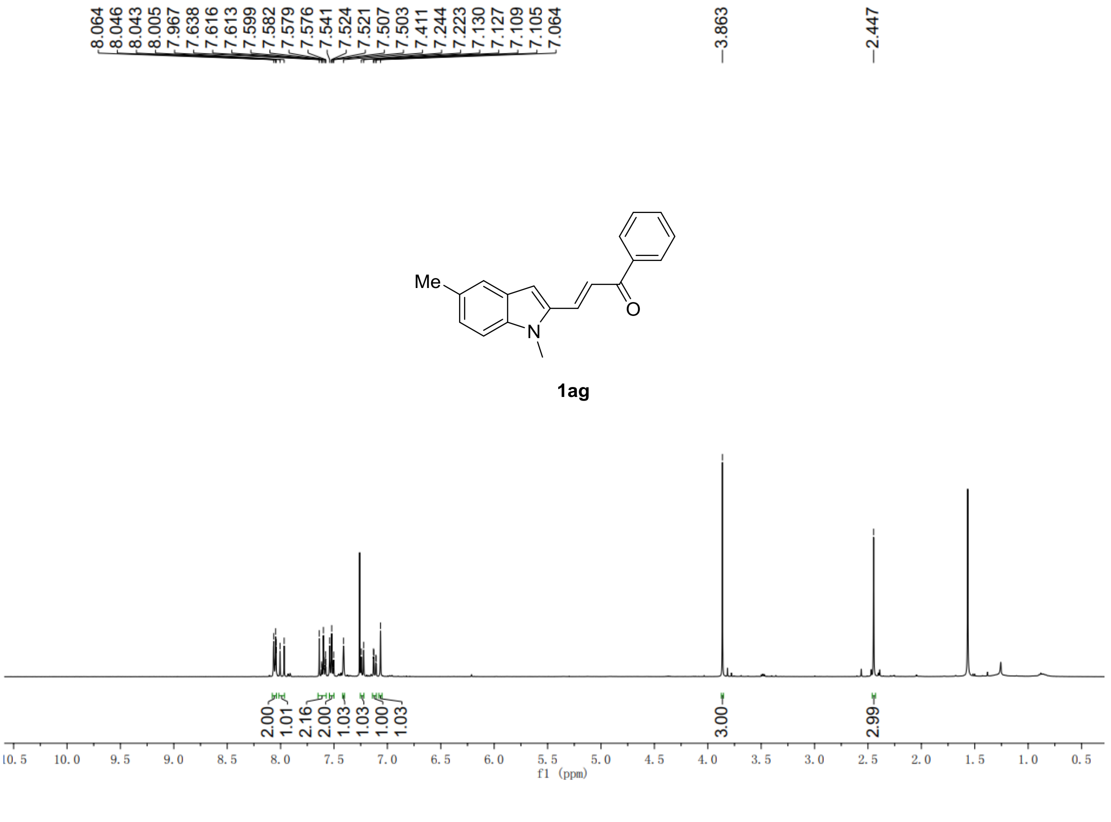

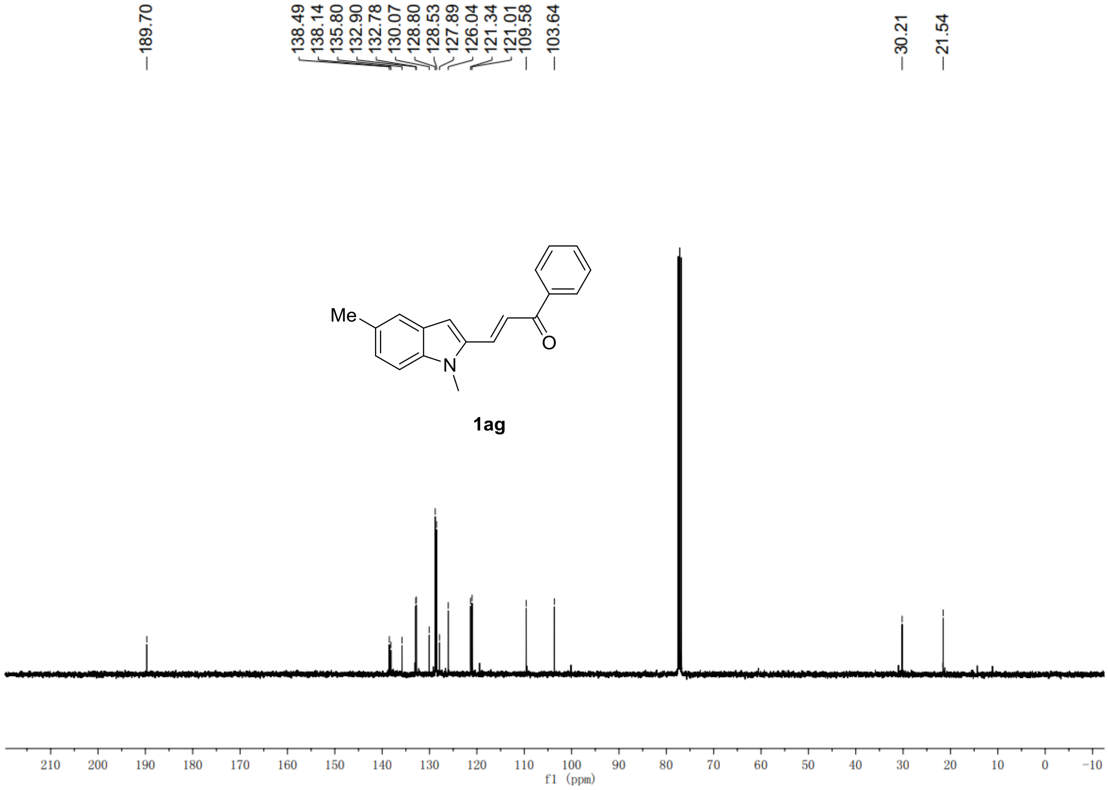


$^{1}$H-NMR and $^{13}$C-NMR of **1ag**

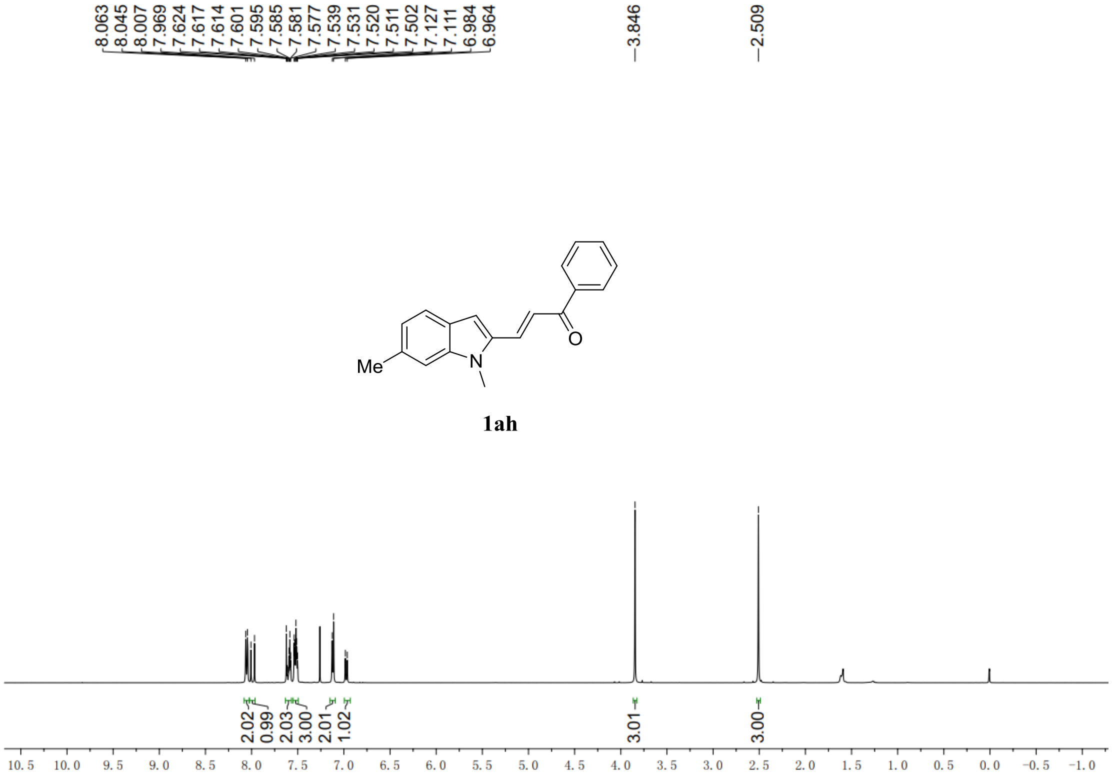

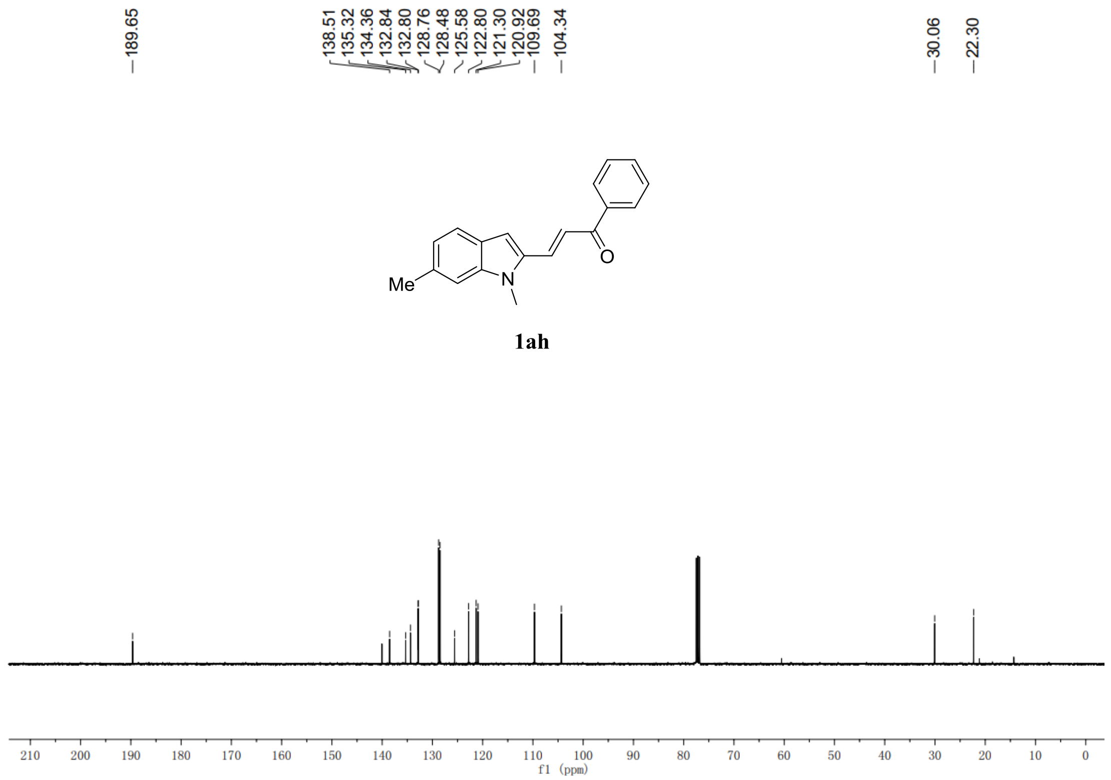


$^{1}$H-NMR and $^{13}$C-NMR of **1ah**

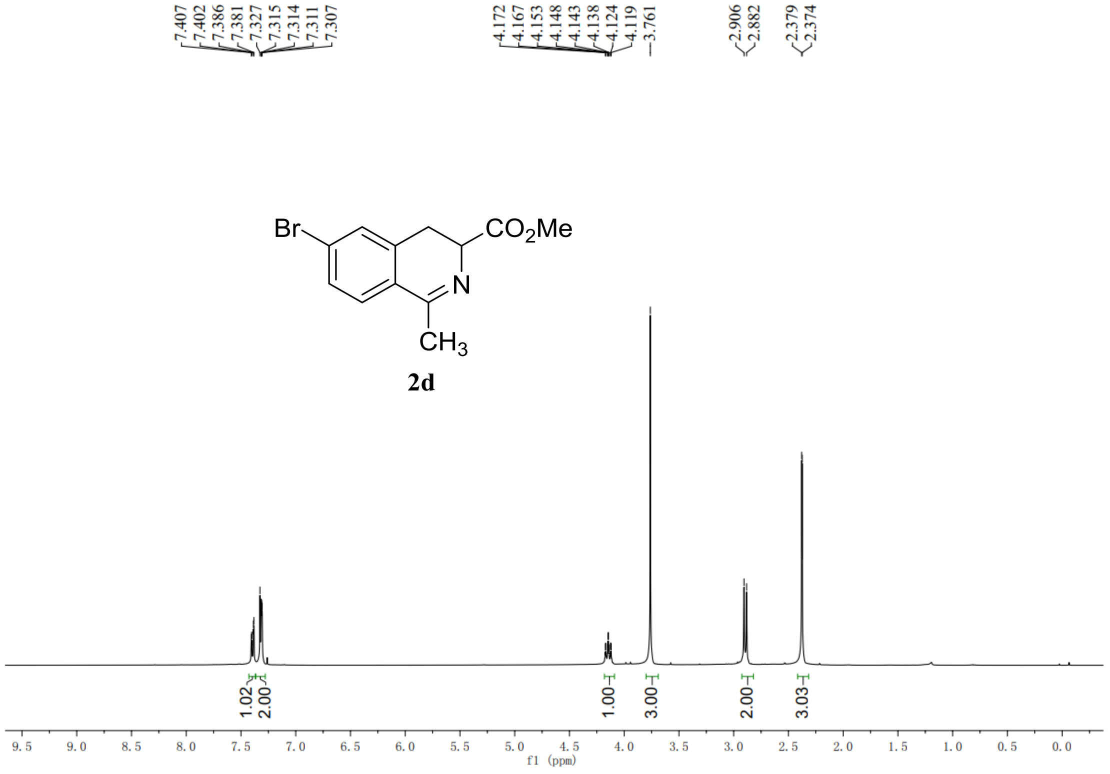




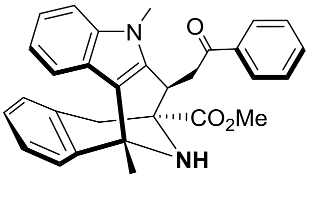

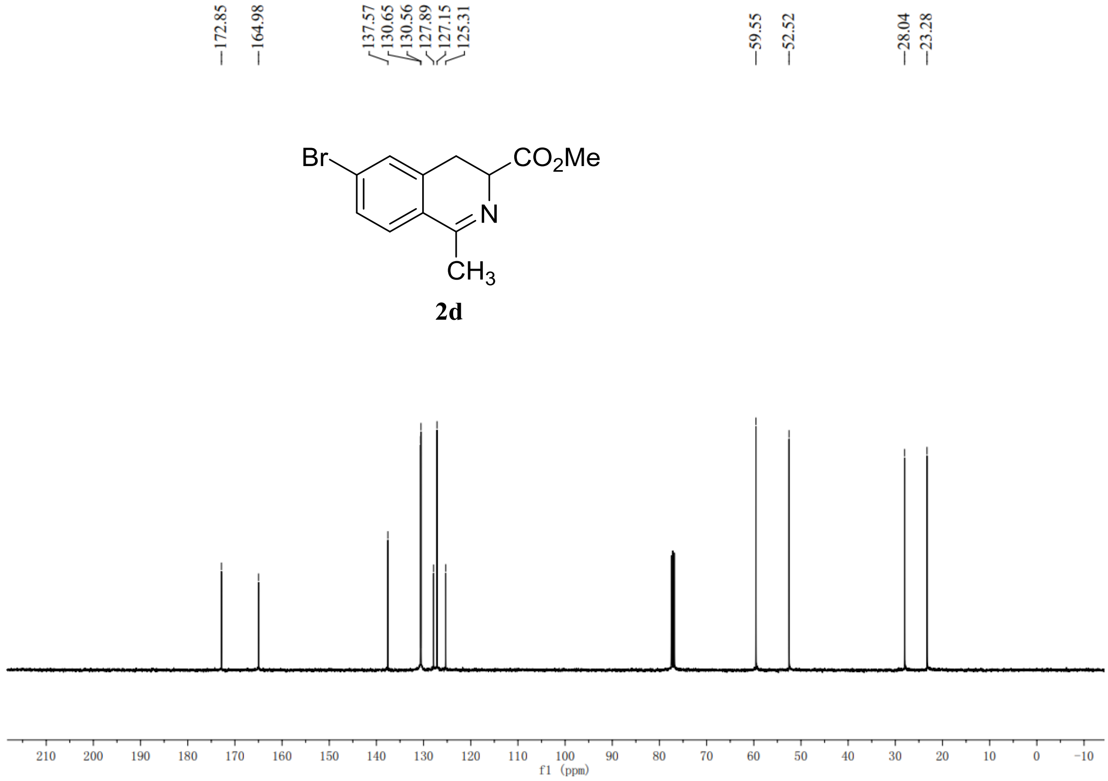


$^1$H-NMR and $^{13}$C-NMR of **2d**

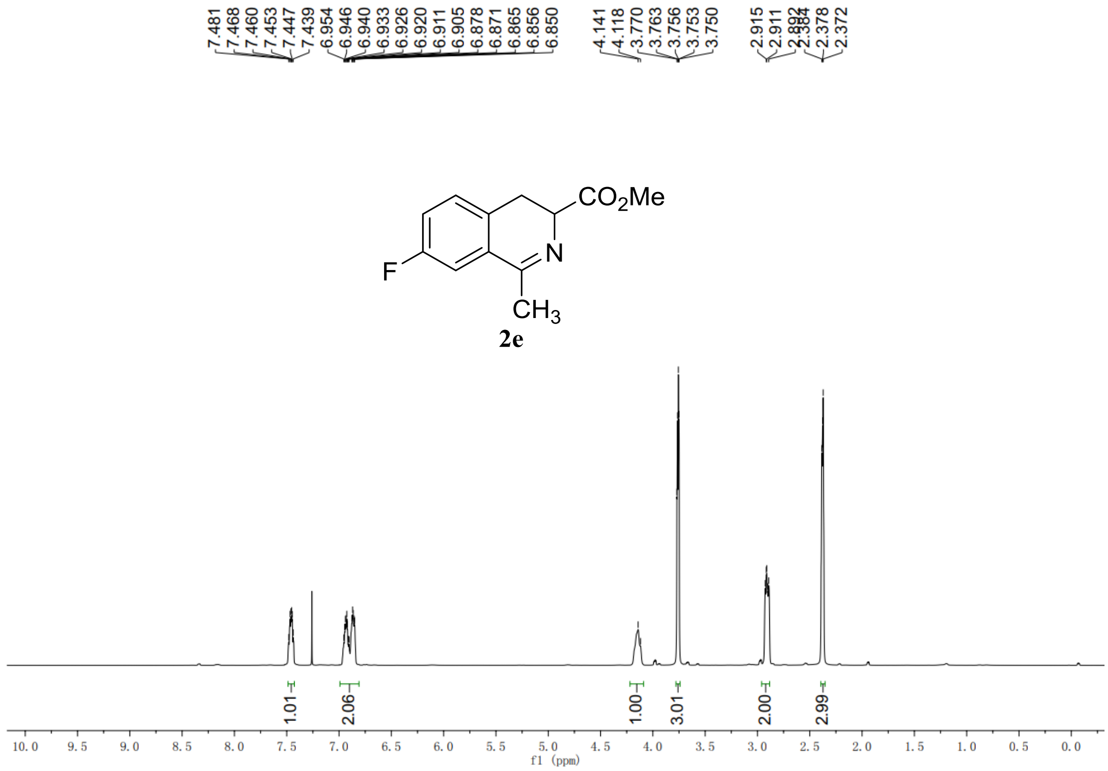

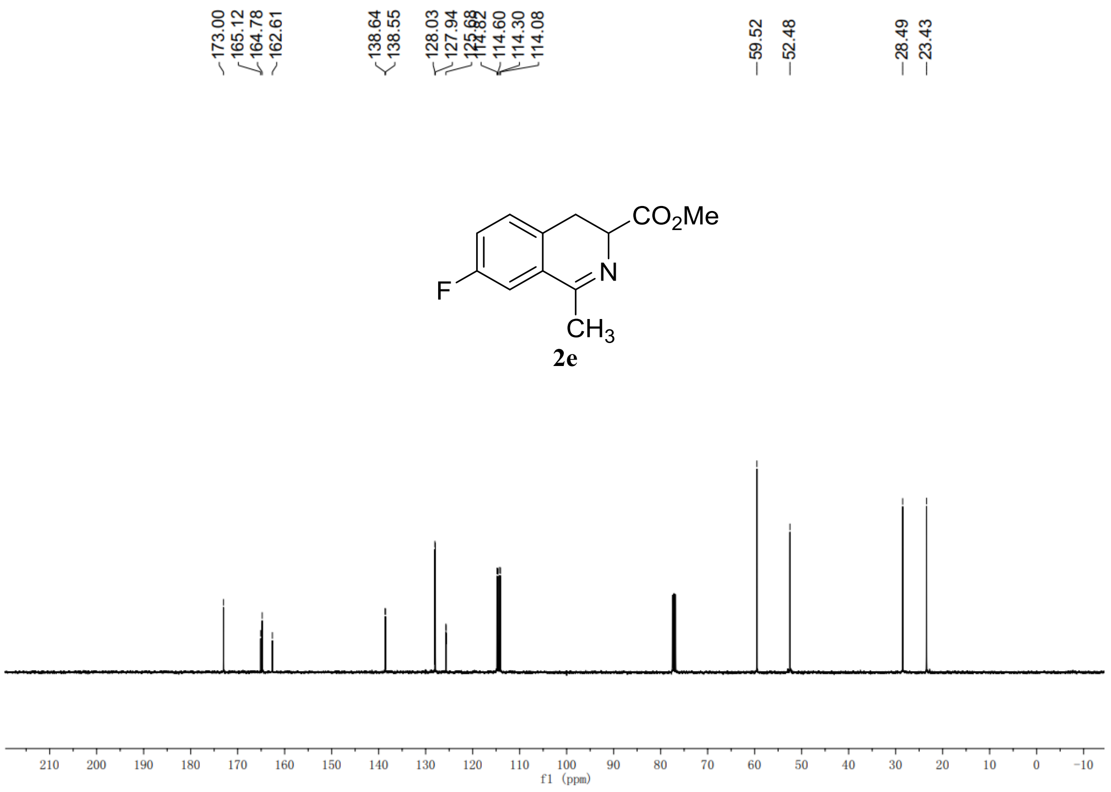

$^1$H-NMR and $^{13}$C-NMR of **2e**

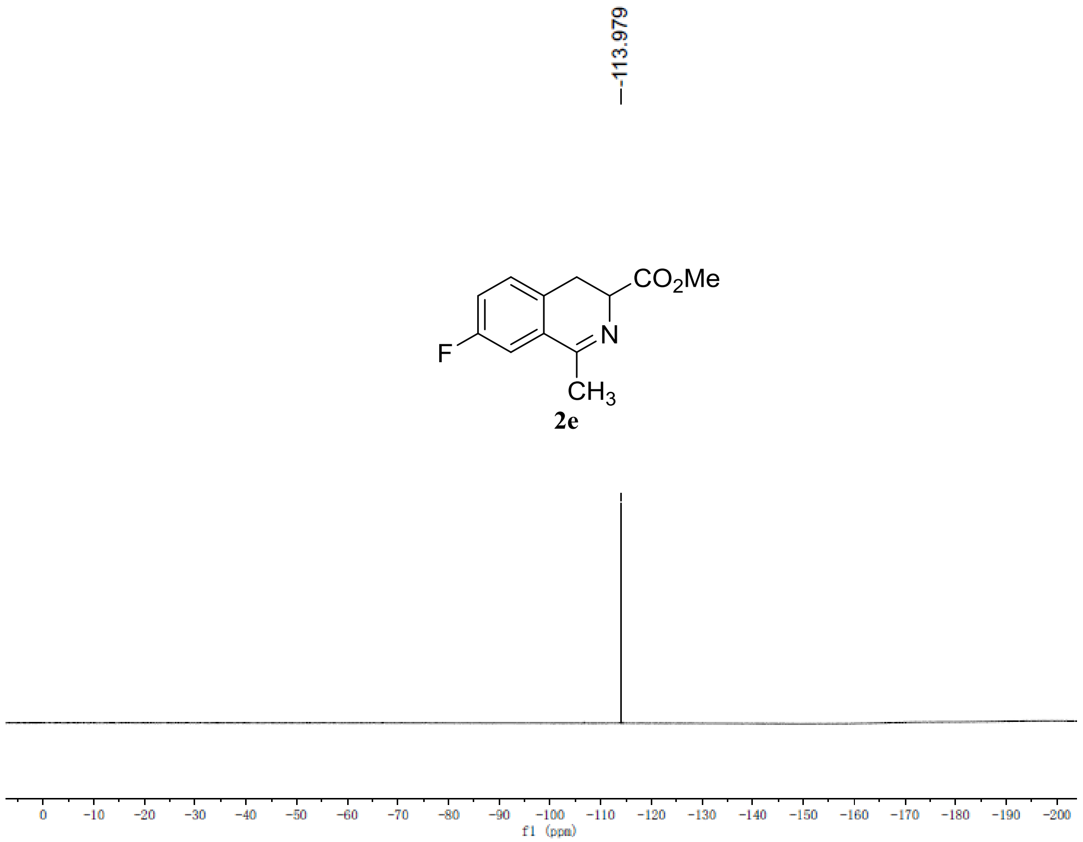


$^{19}$F-NMR of **2e**

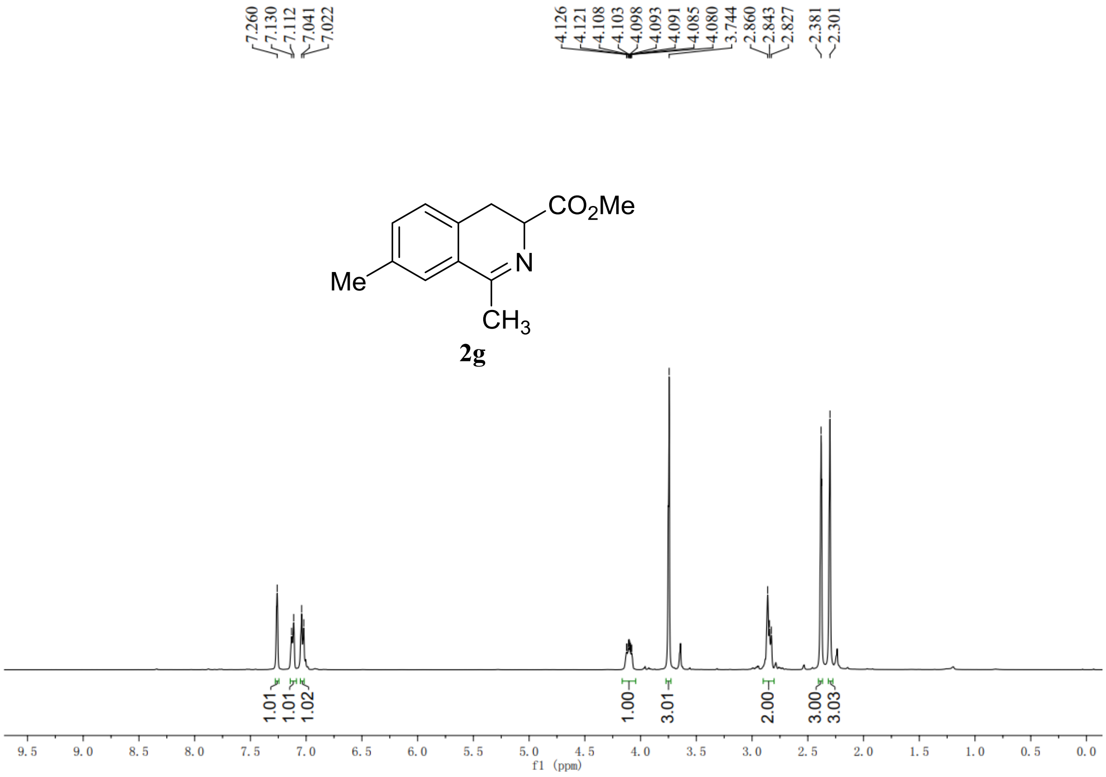

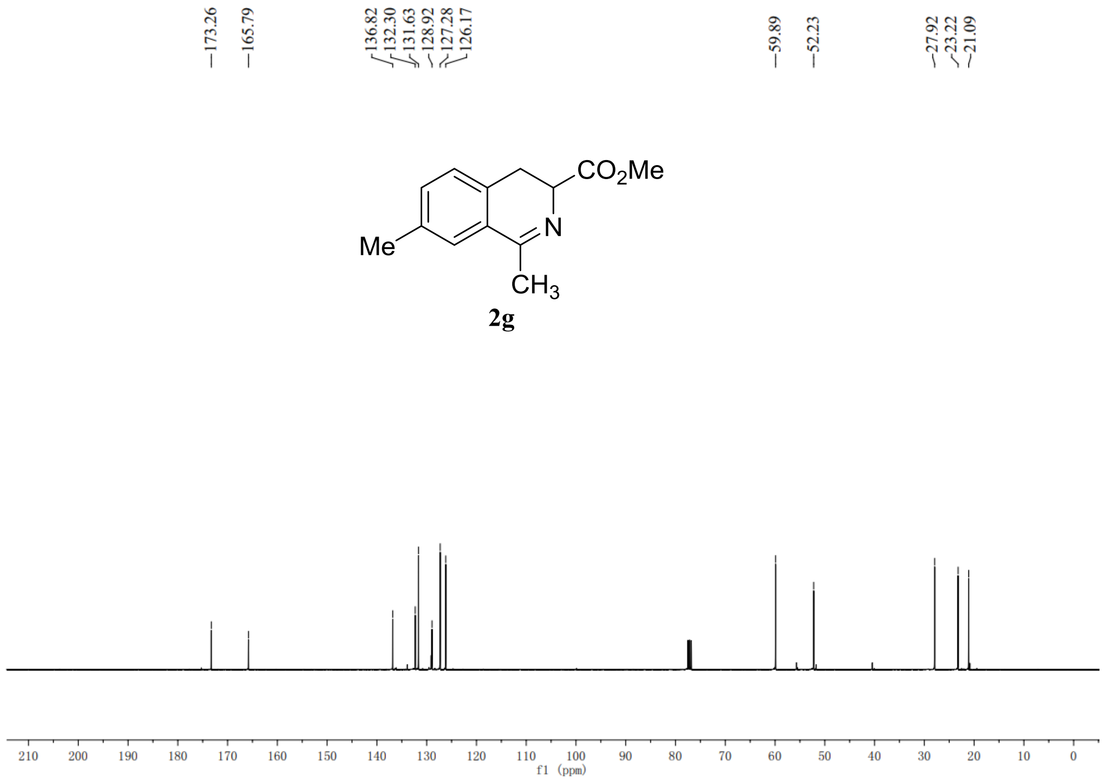


$^{1}$H-NMR and $^{13}$C-NMR of **2g**

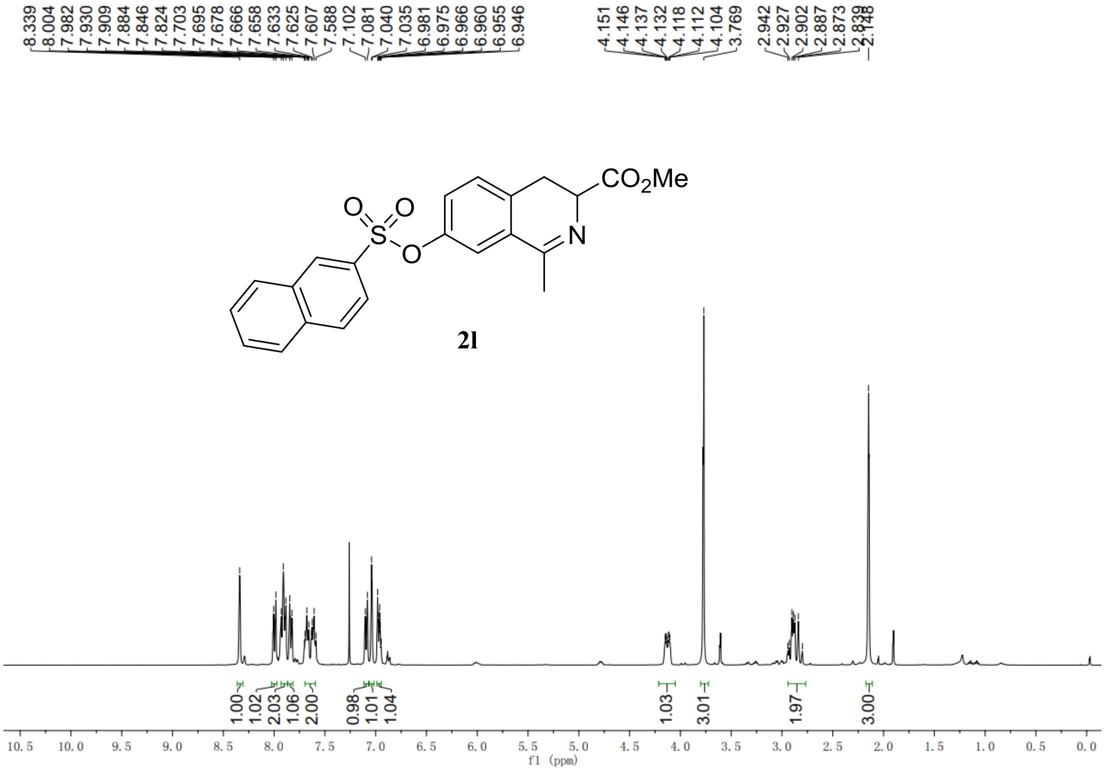

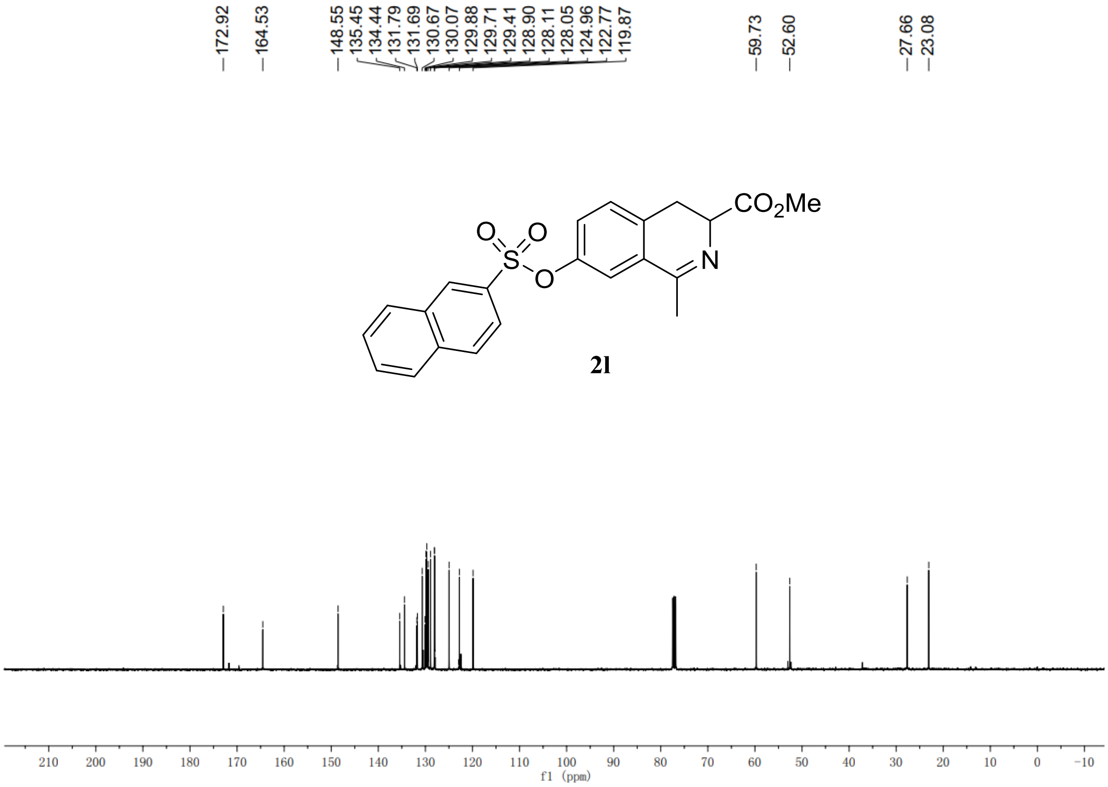


$^{1}$H-NMR and $^{13}$C-NMR of **2l**

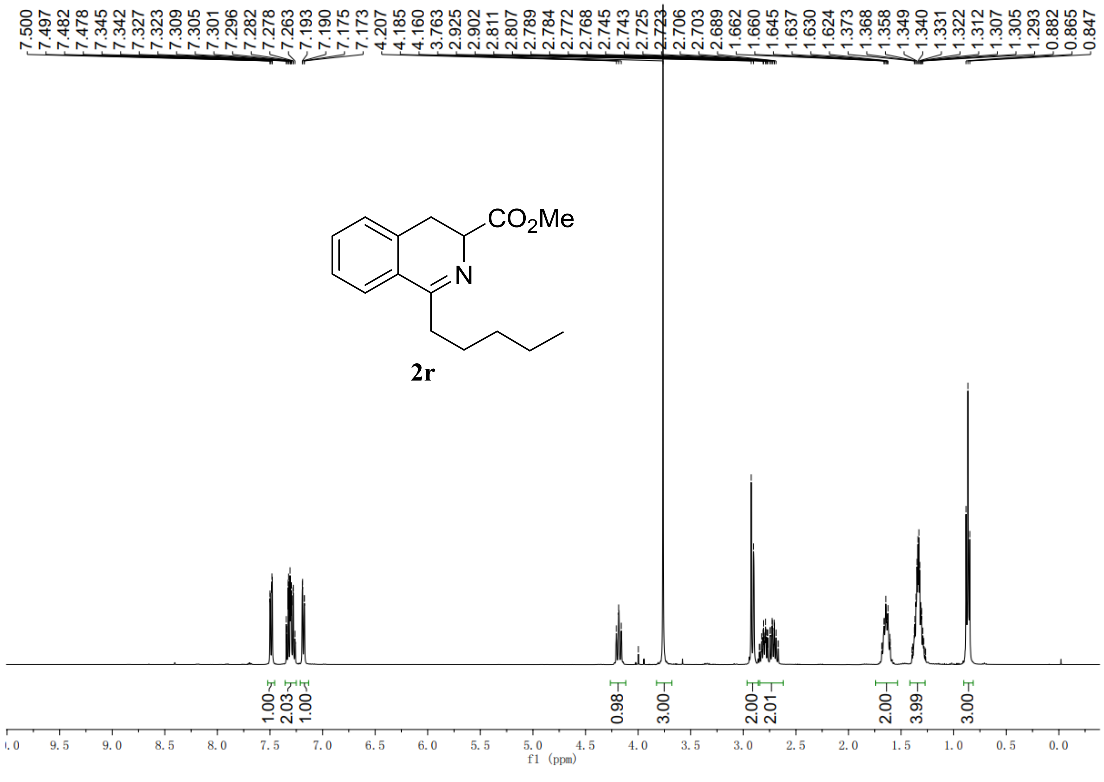

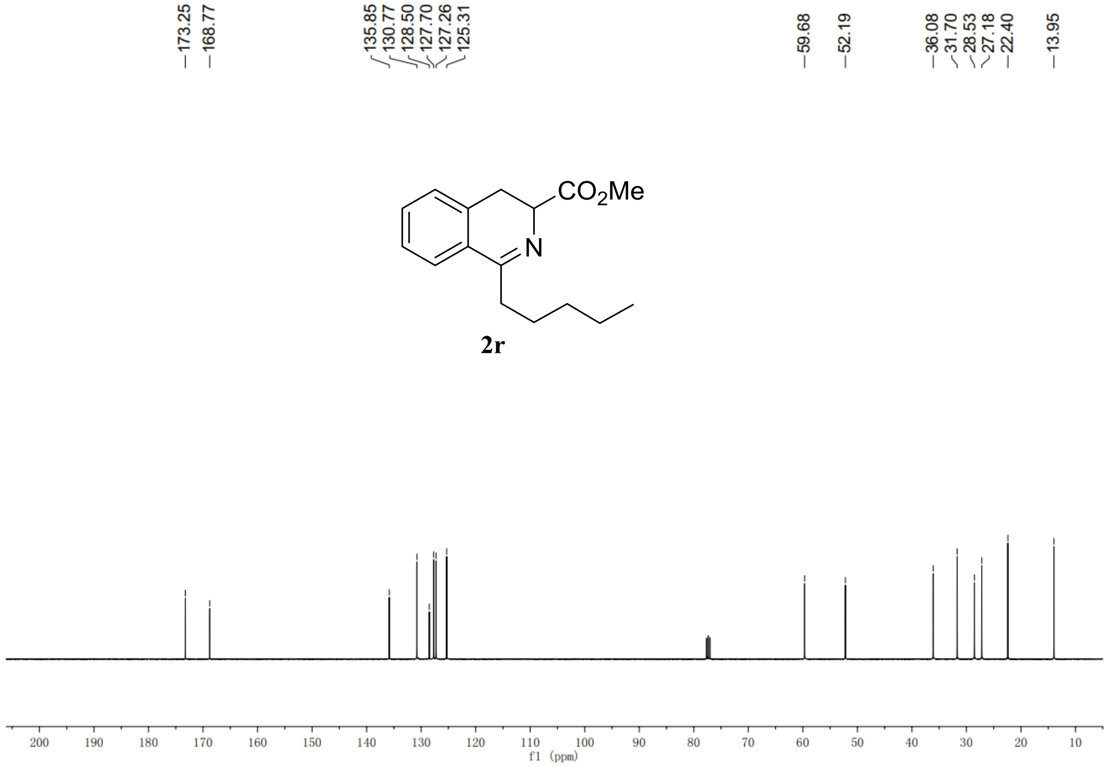


$^1$H-NMR and $^{13}$C-NMR of **2r**

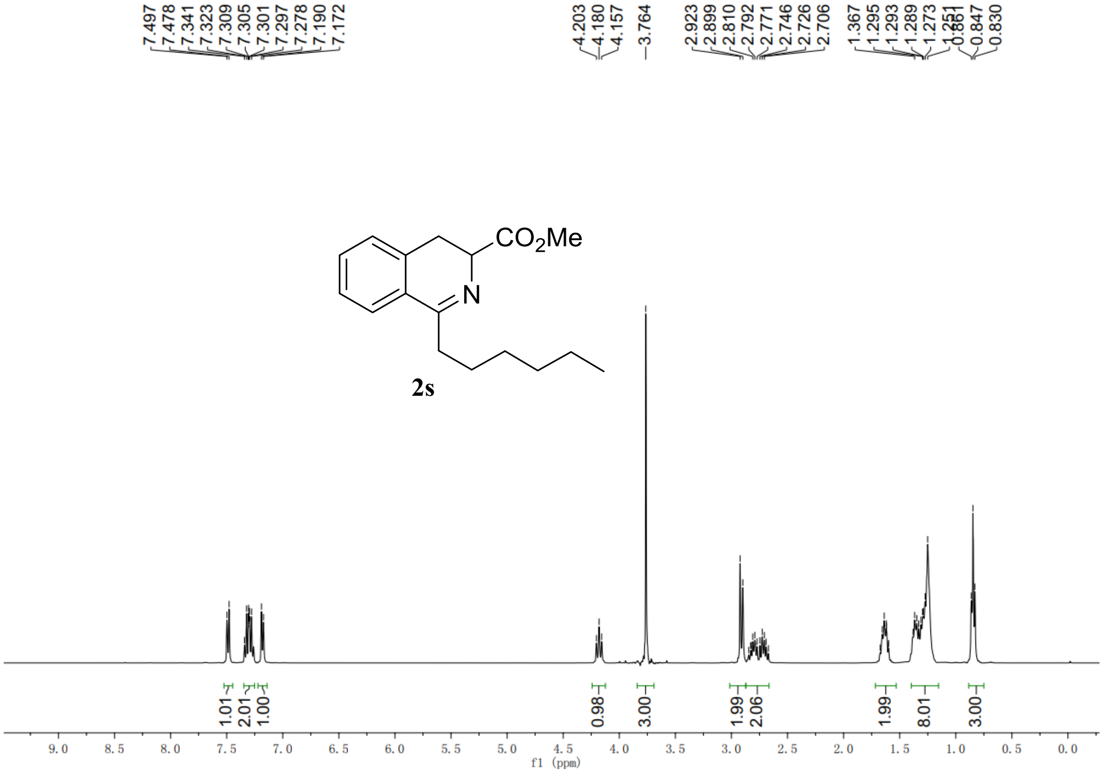

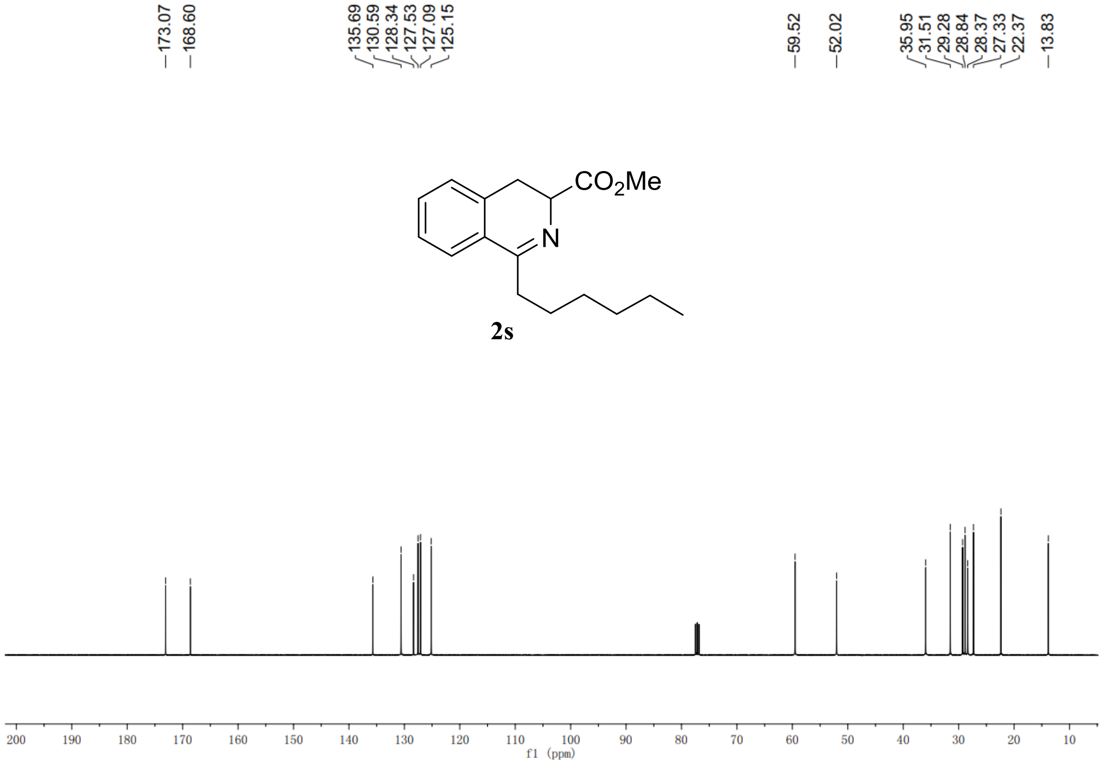


$^1$H-NMR and $^{13}$C-NMR of **2s**

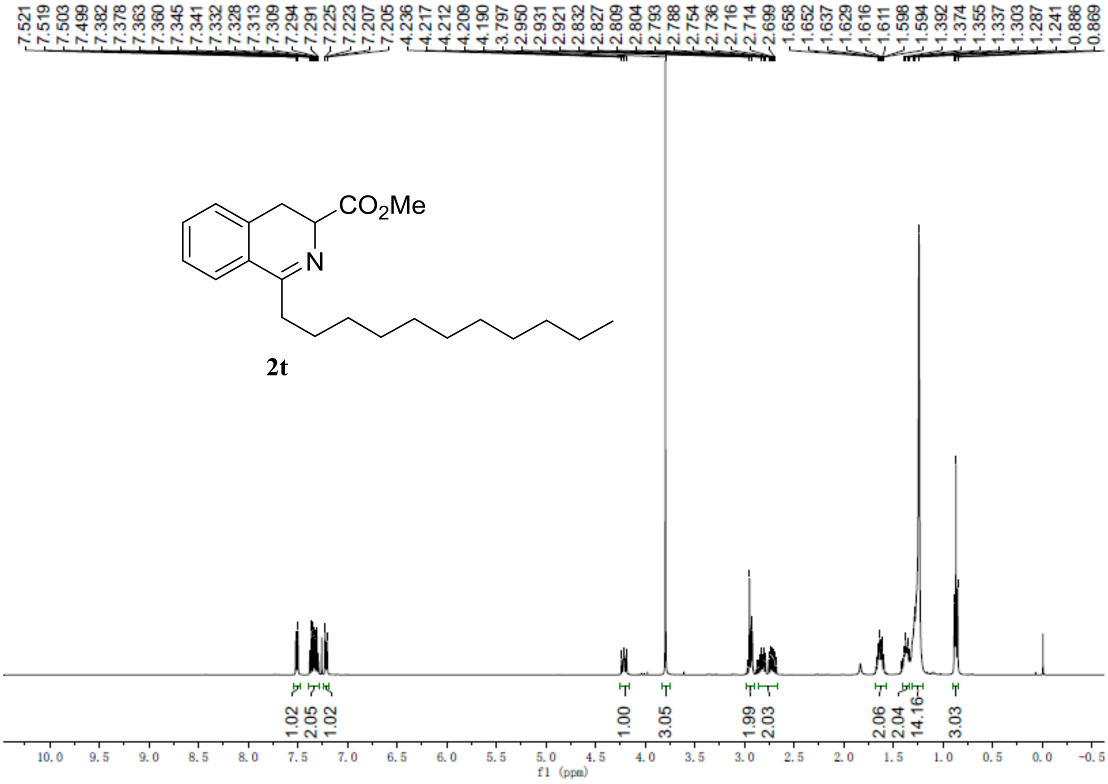

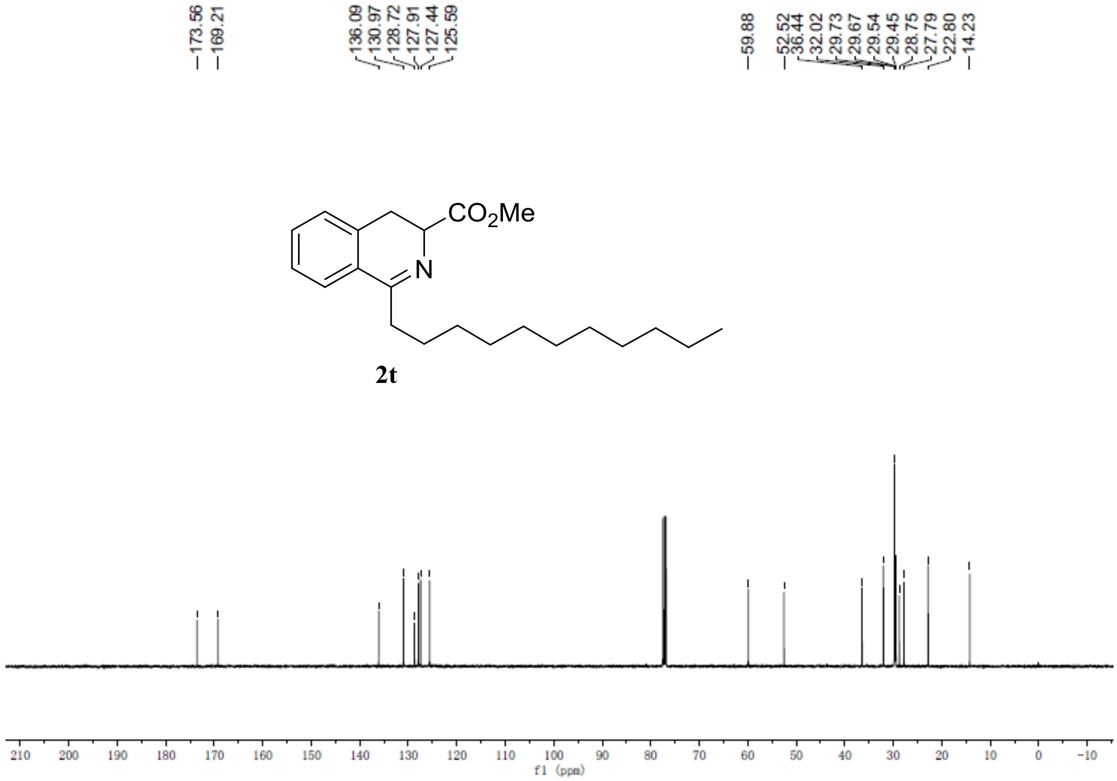


$^{1}$H-NMR and $^{13}$C-NMR of **2t**

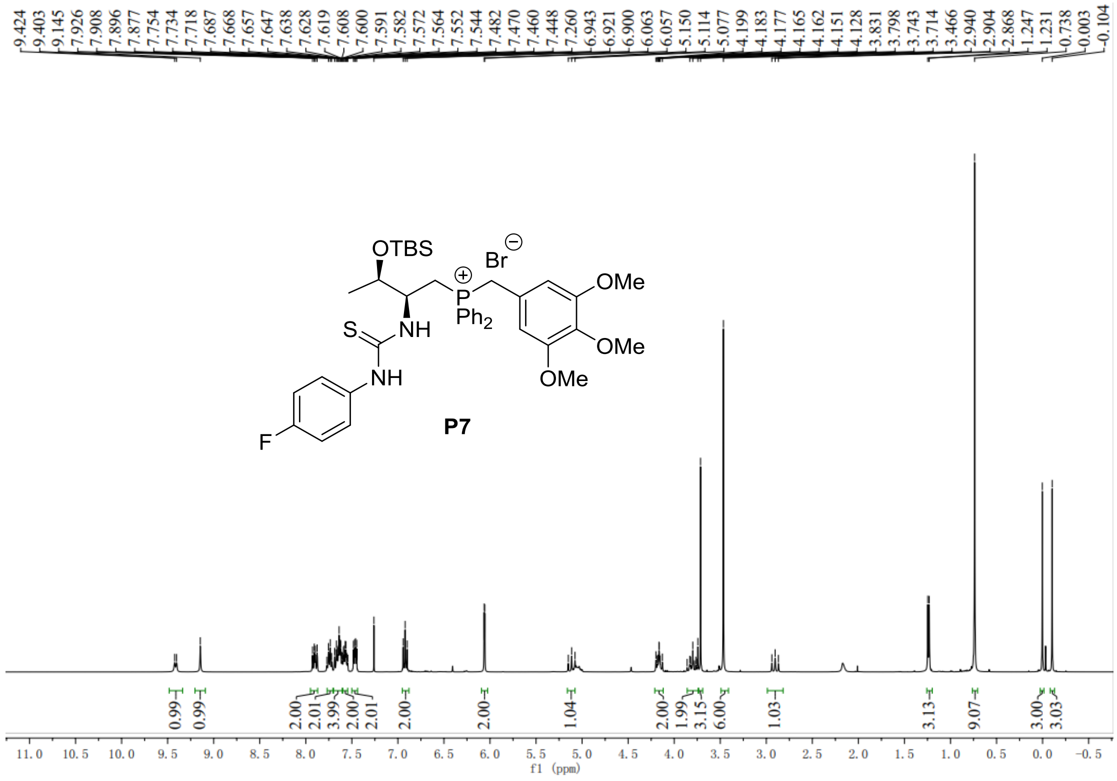

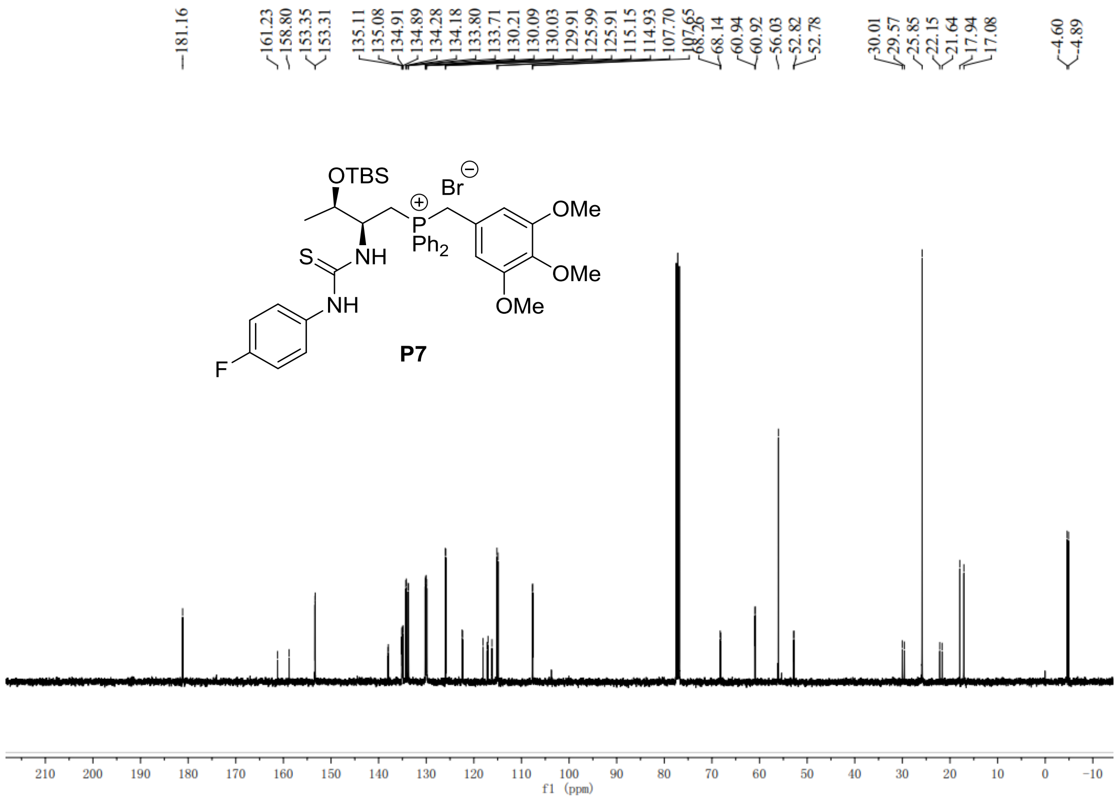


$^{1}$H-NMR and $^{13}$C-NMR of **P7**

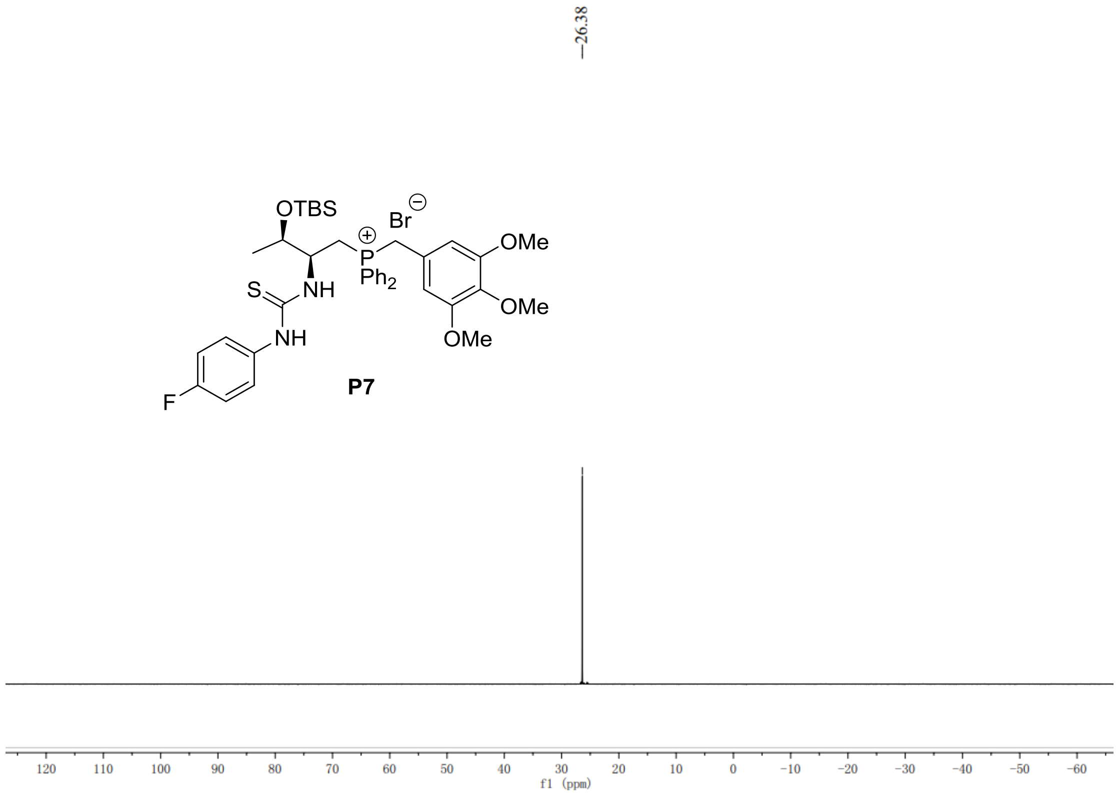

$^{31}$P-NMR of **P7**

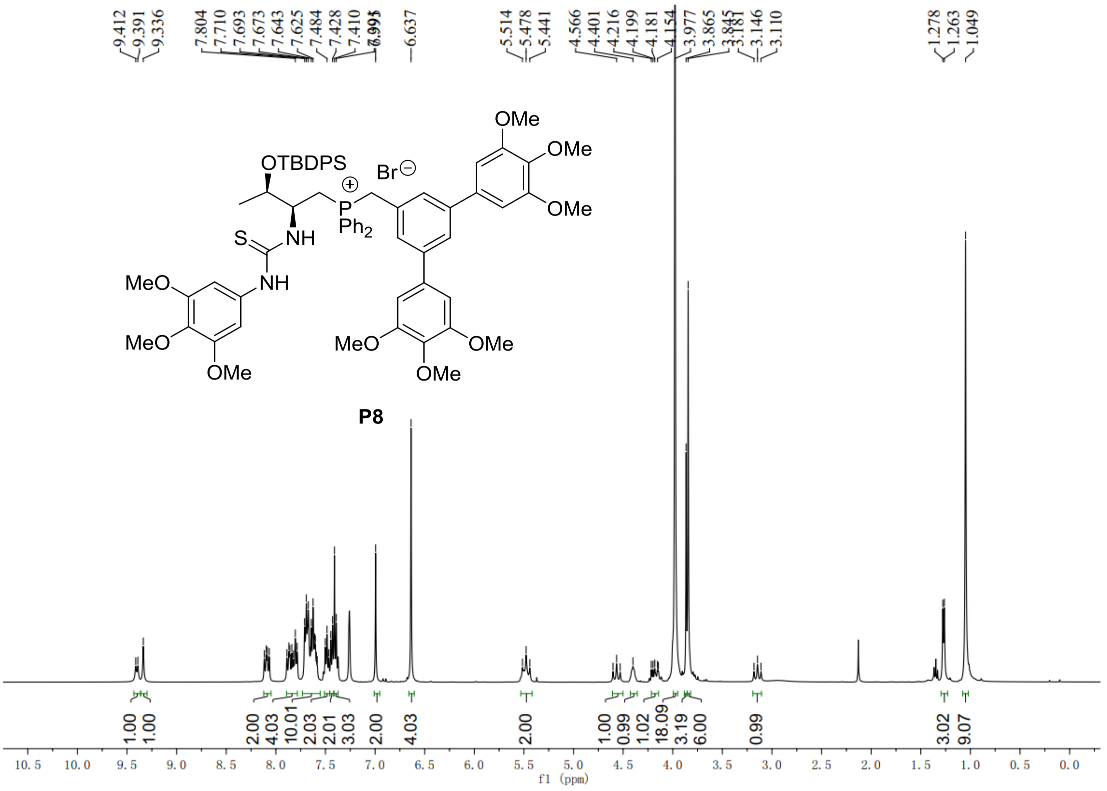


$^{1}$H-NMR of **P8**

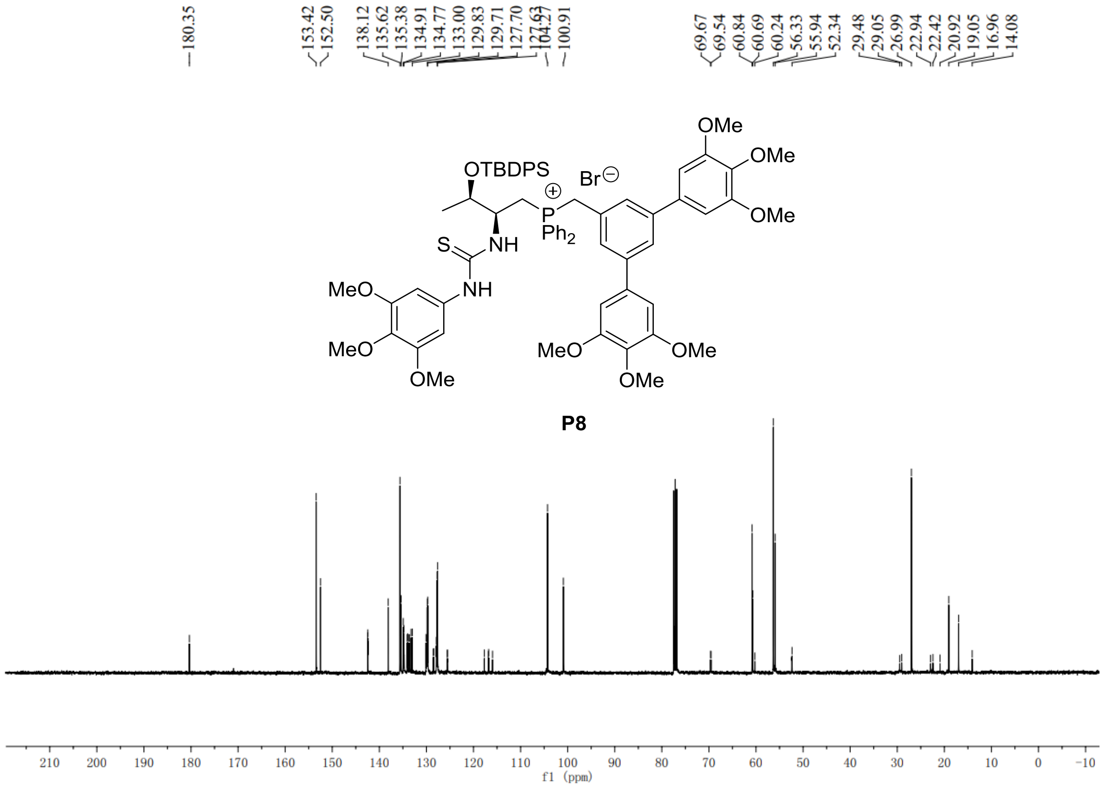


$^{13}$C-NMR of **P8**

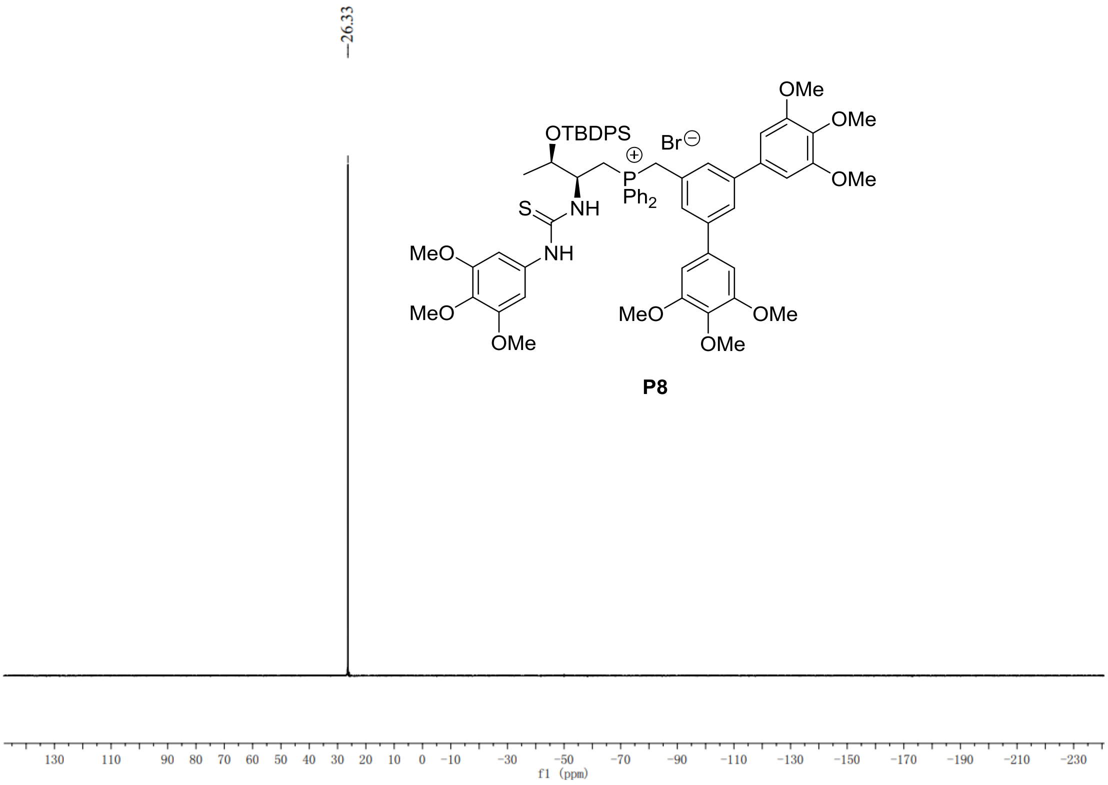

$^{31}$P-NMR of **P8**

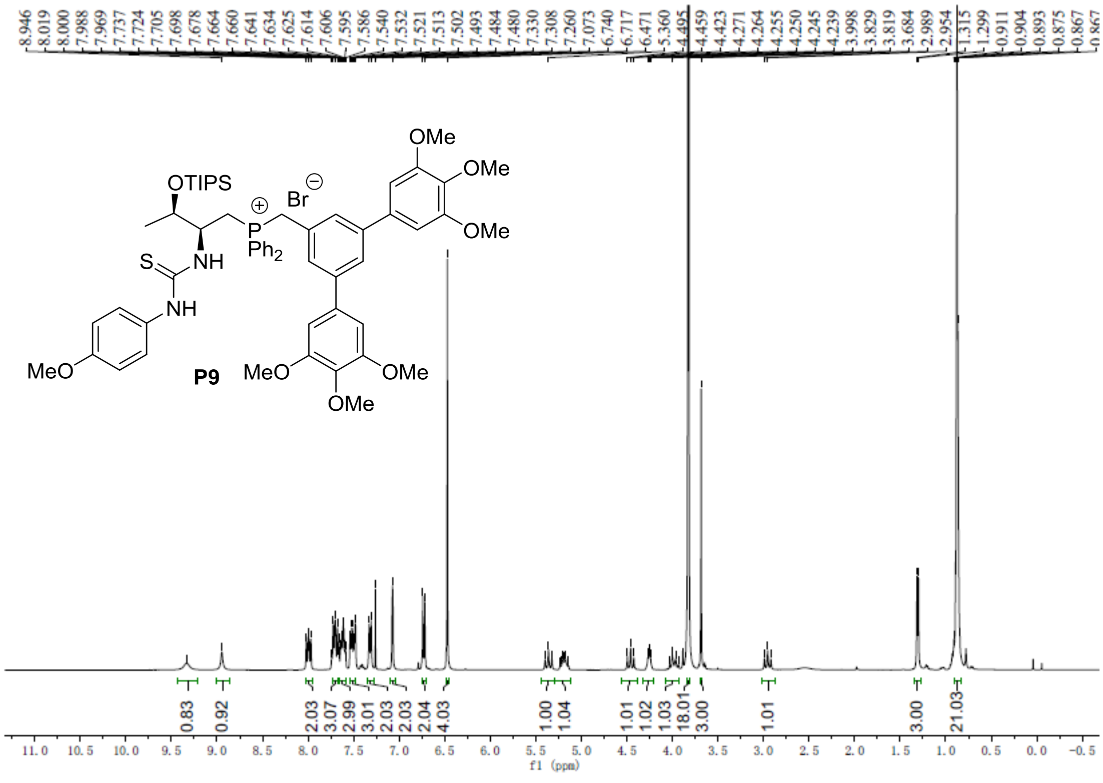

$^{1}$H-NMR of **P9**

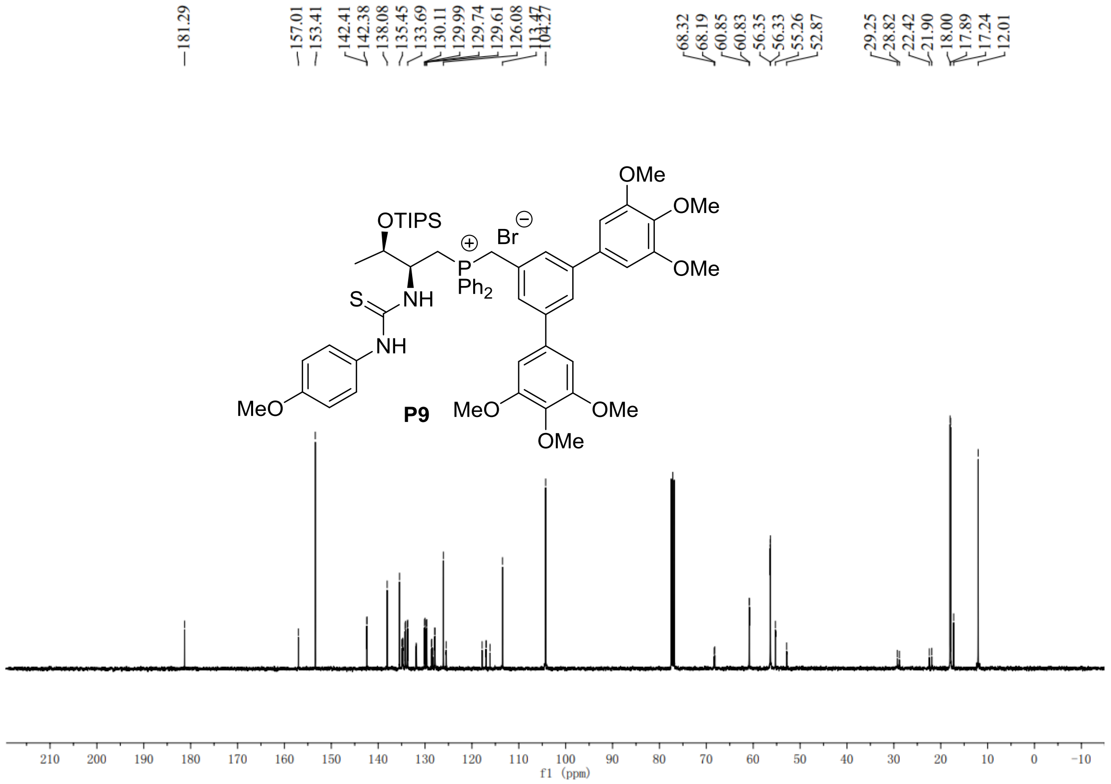


$^{13}$C-NMR of **P9**

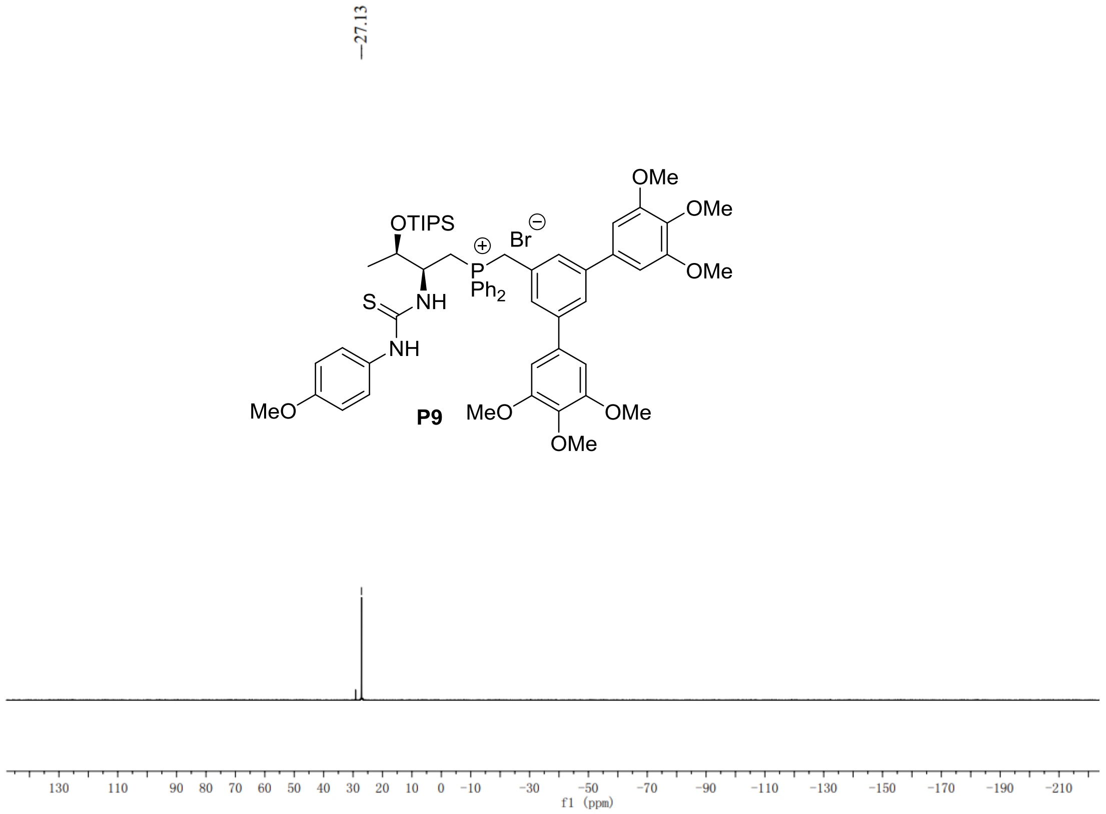


$^{31}$P-NMR of **P9**

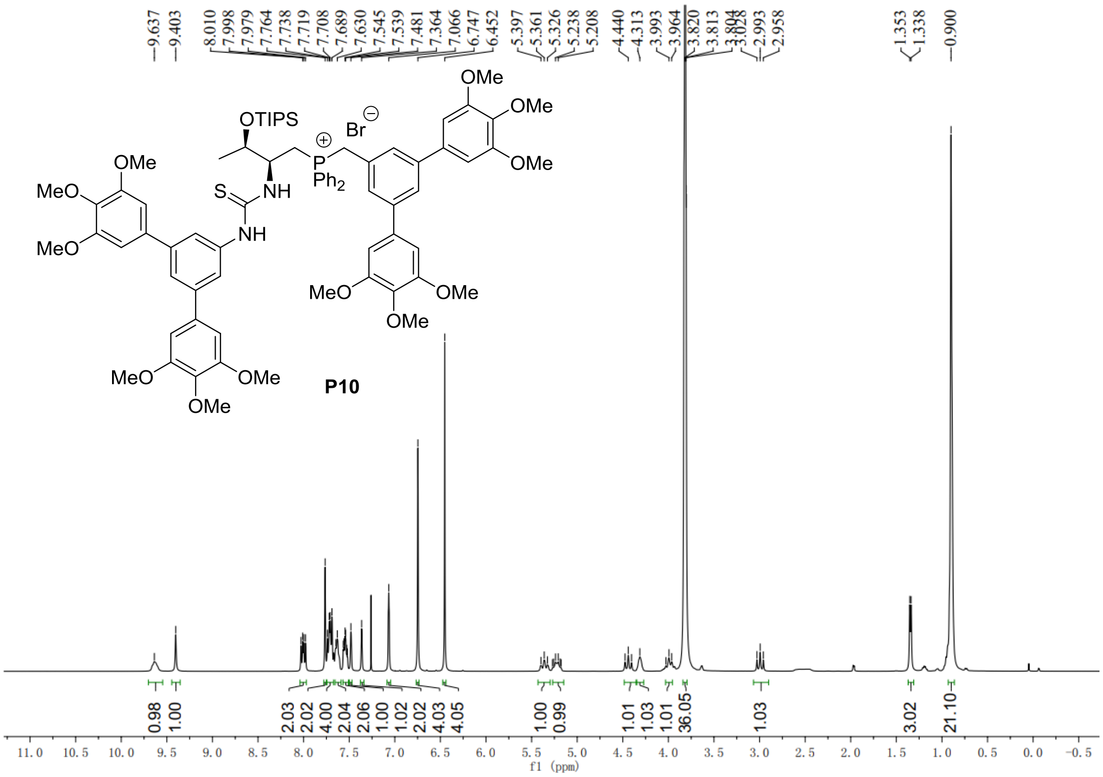

$^1$H-NMR of **P10**

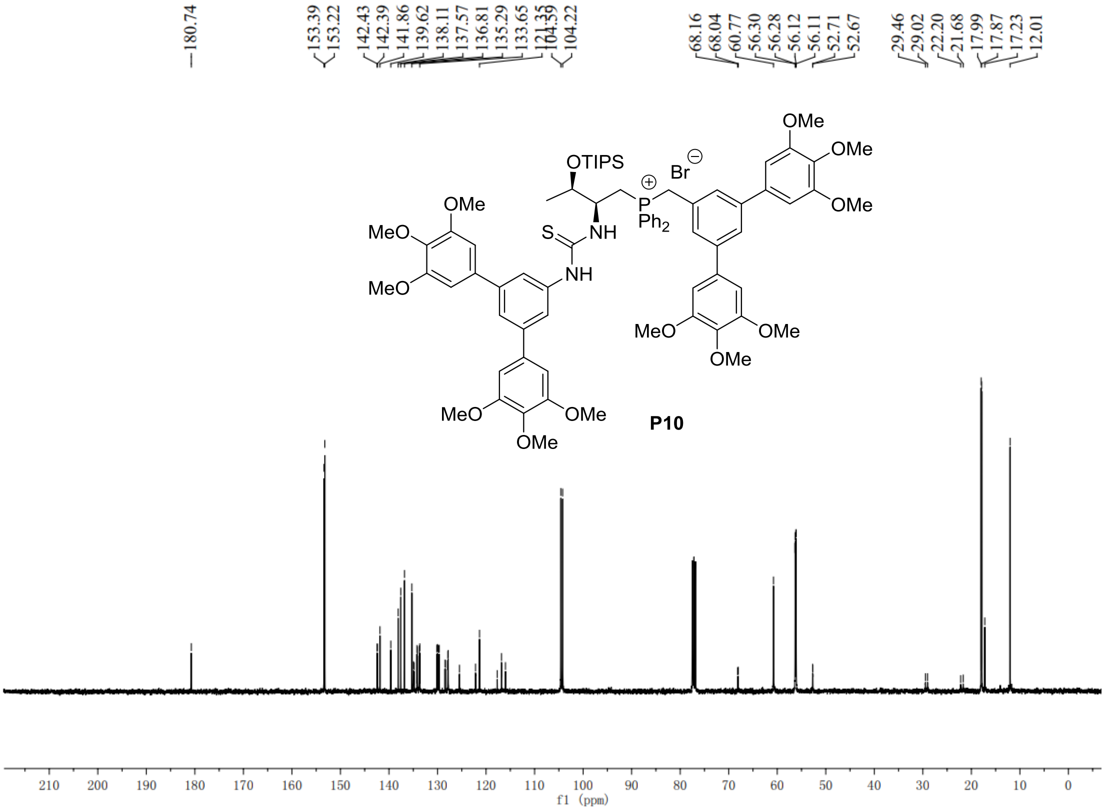

$^{13}$C-NMR of **P10**

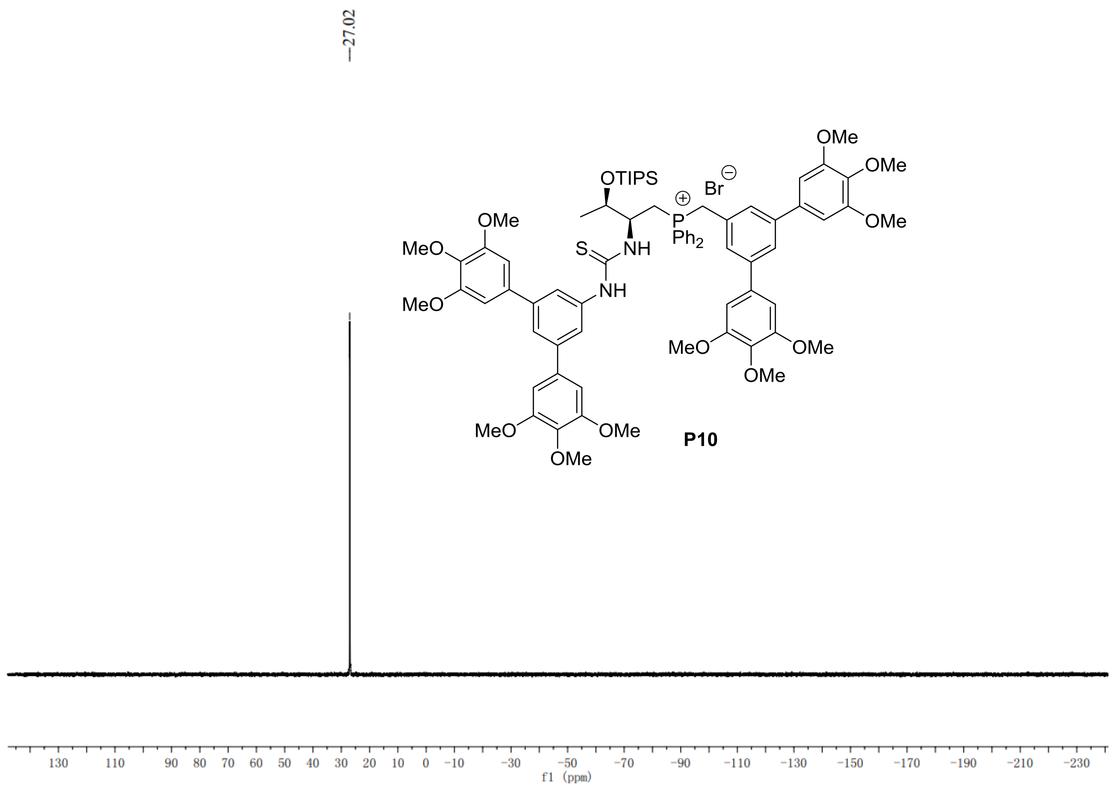

$^{31}$P-NMR of **P10**

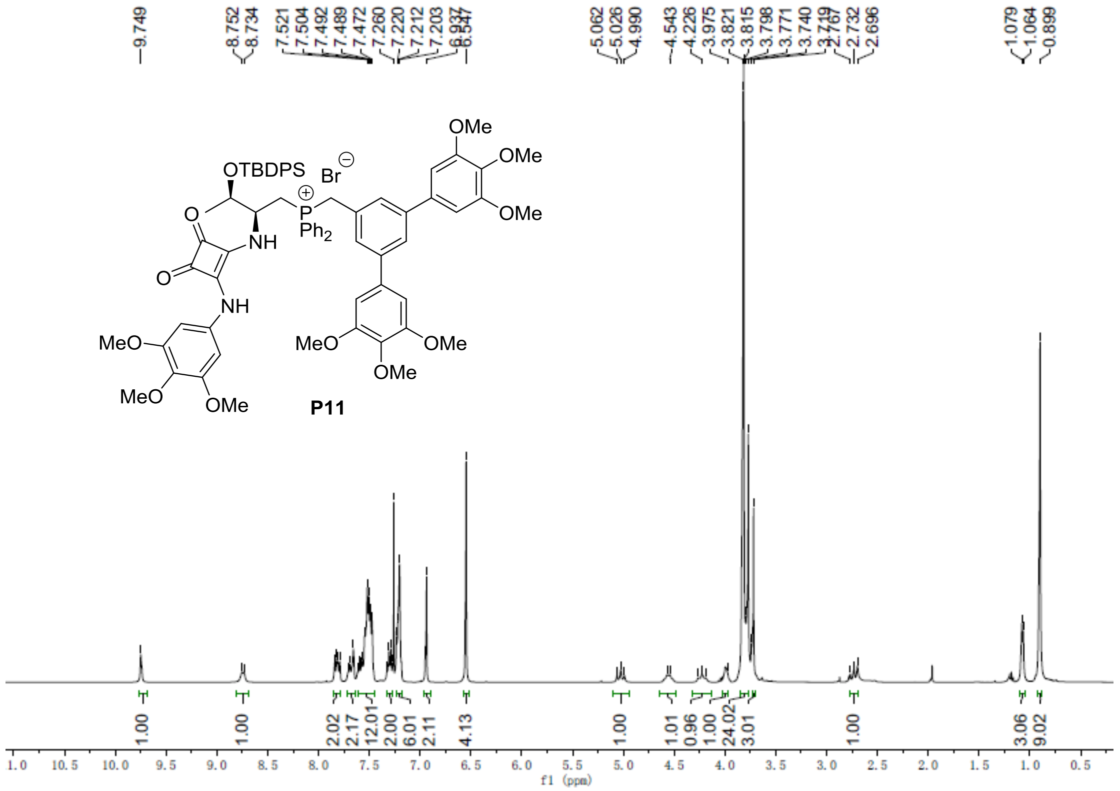

$^{1}$H-NMR of **P11**

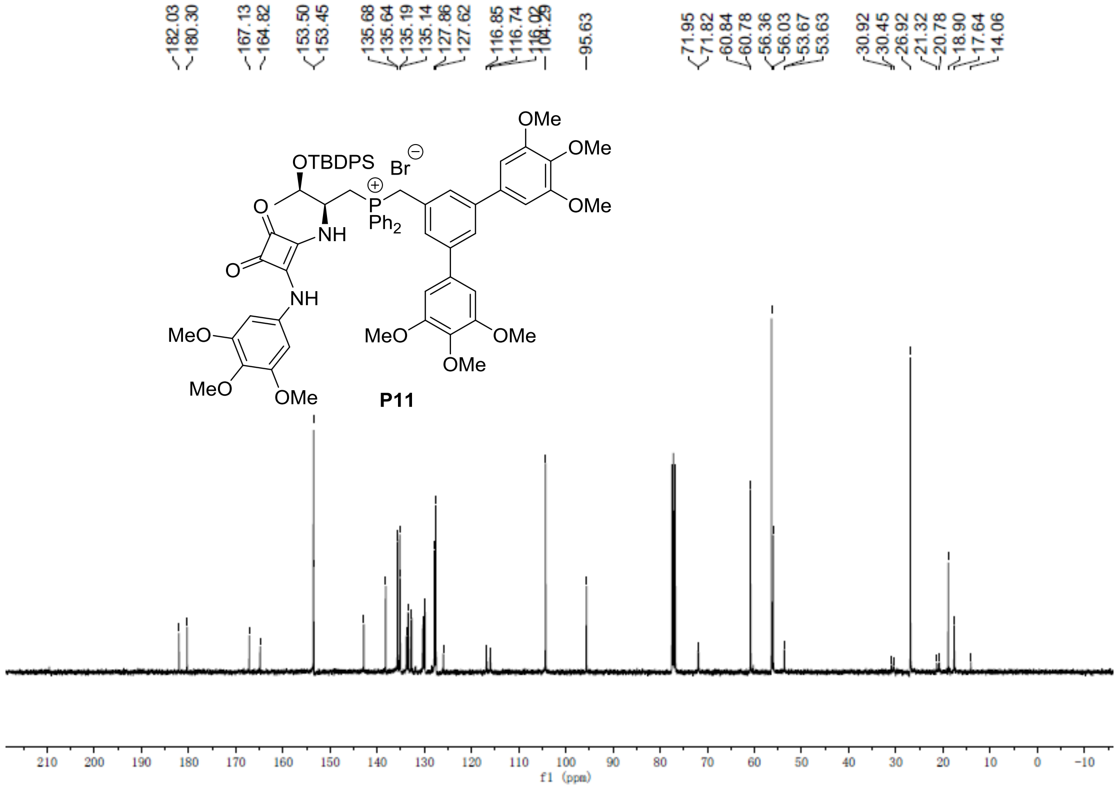

$^{13}$C-NMR of **P11**

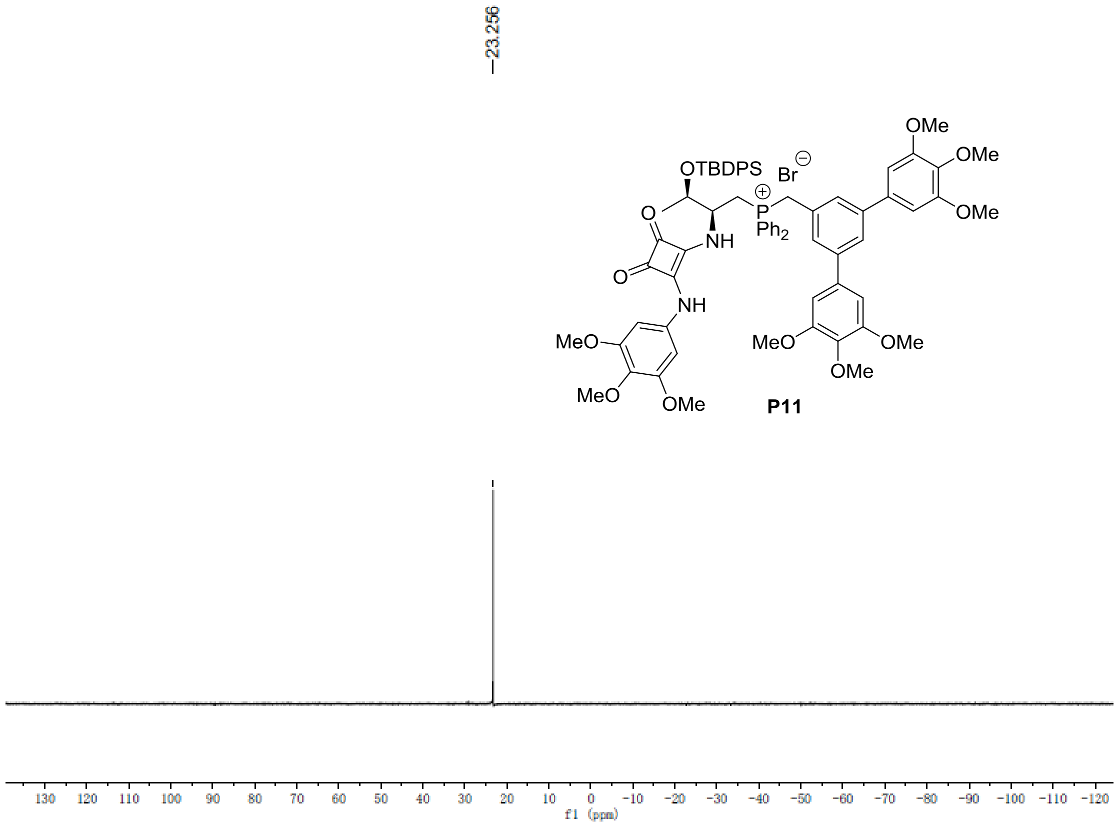

$^{31}$P-NMR of **P11**

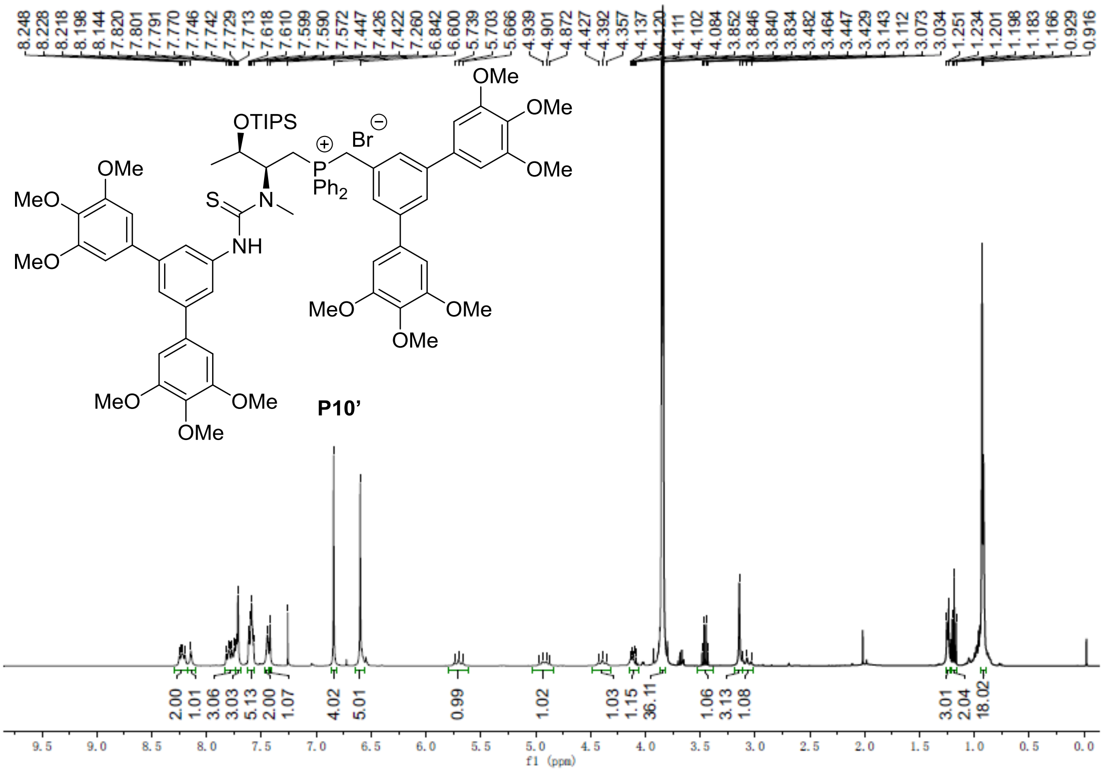

$^1$H-NMR of **P10’**

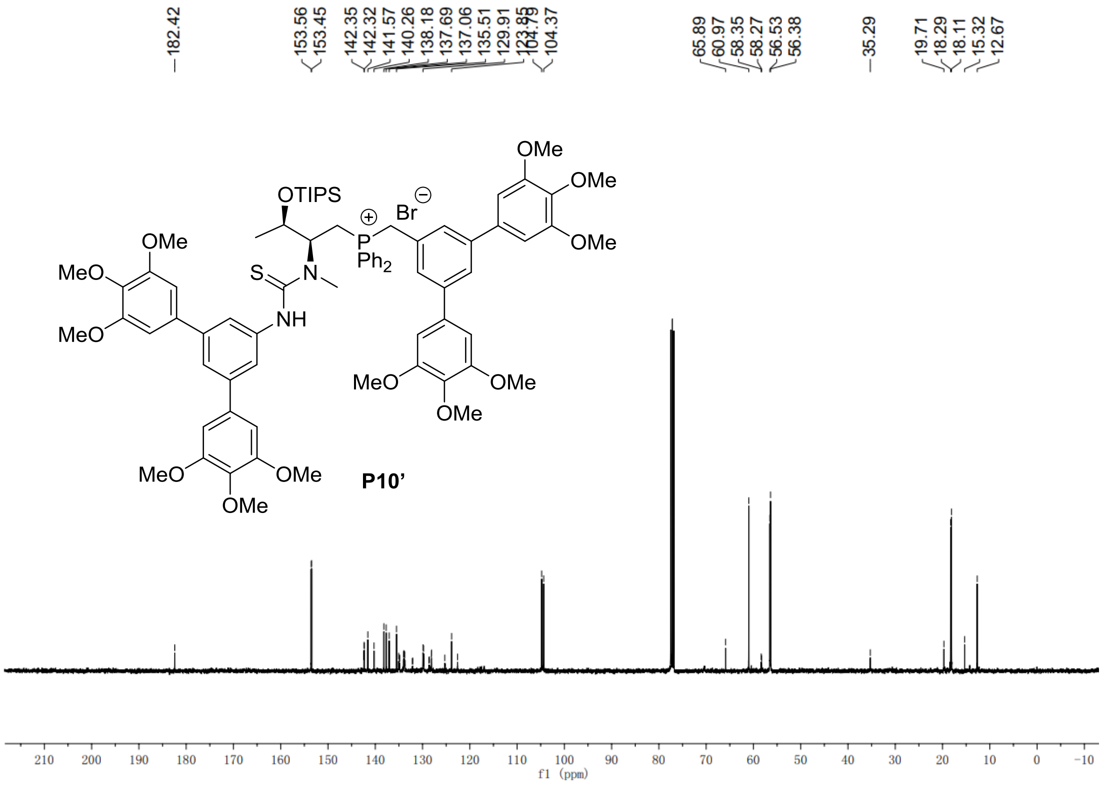

$^{13}$C-NMR of **P10’**

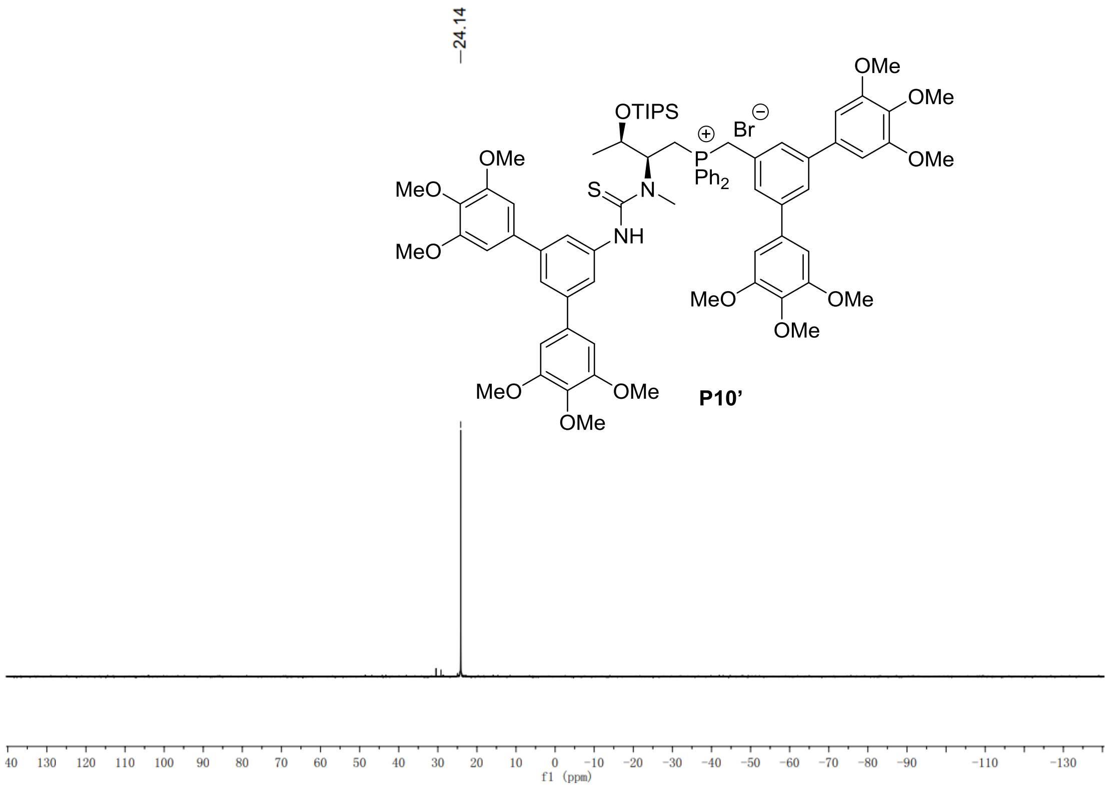

$^{31}$P-NMR of **P10'**

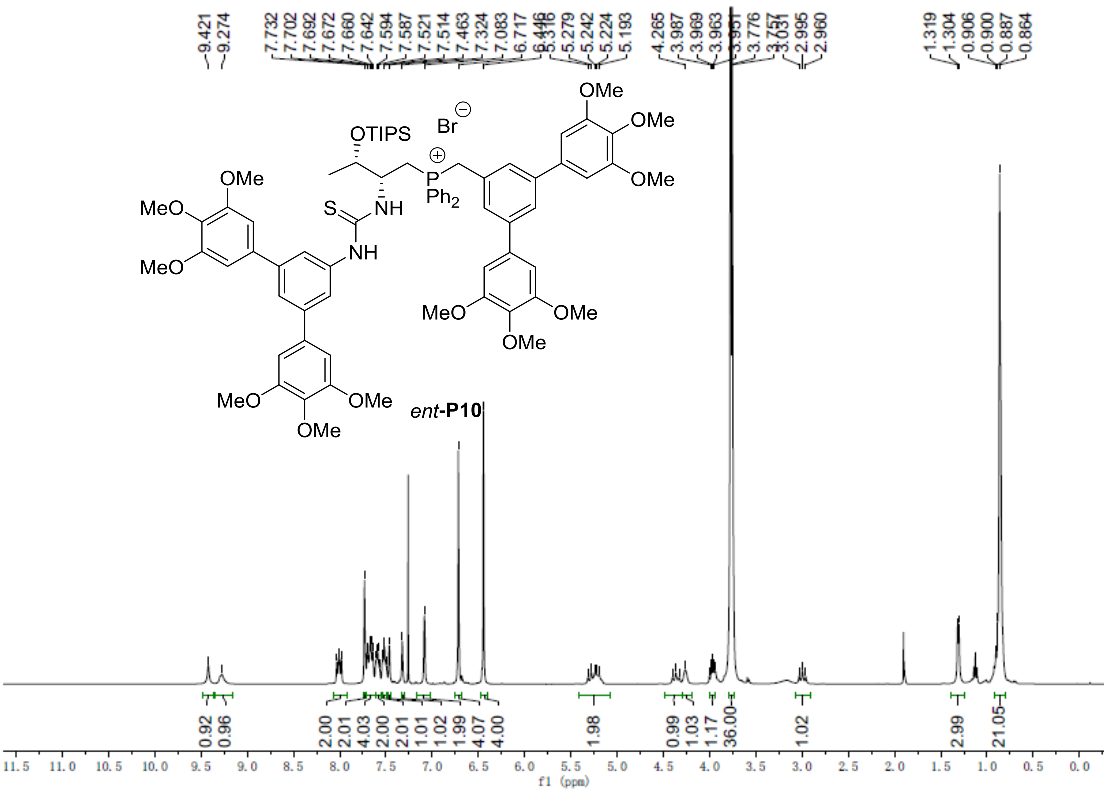

$^{1}$H-NMR of *ent*-**P10**

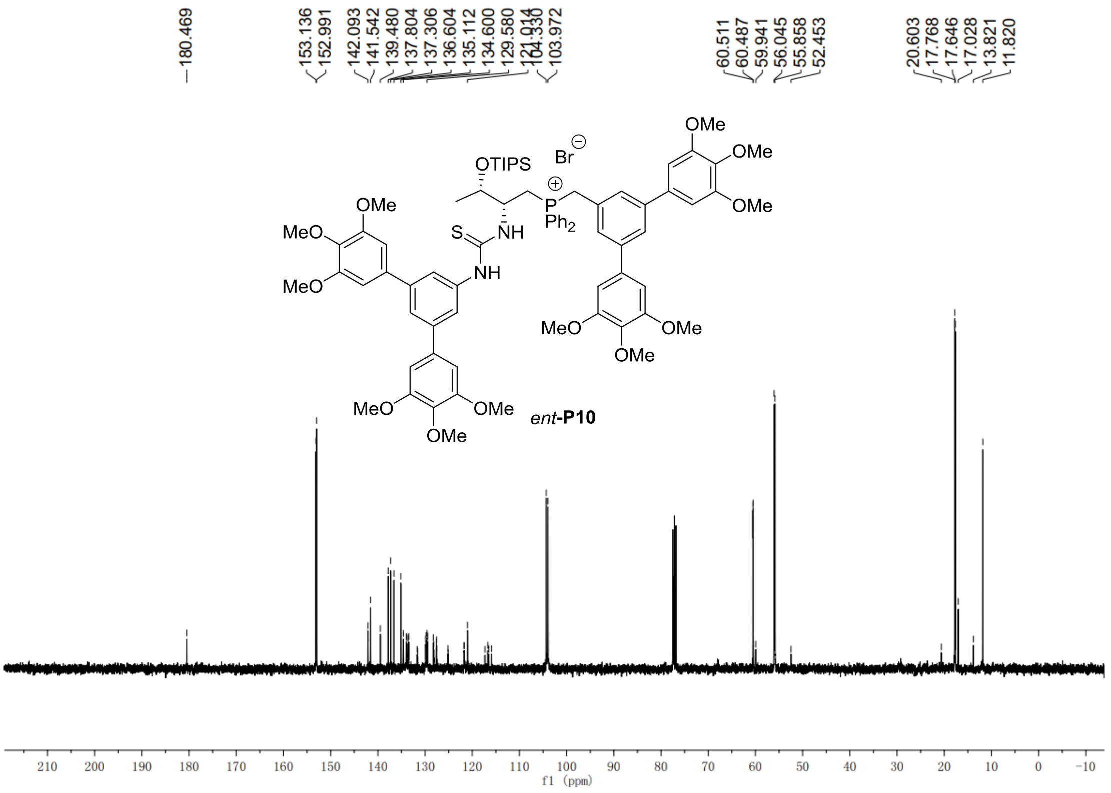


$^{13}$C-NMR of *ent*-**P10**

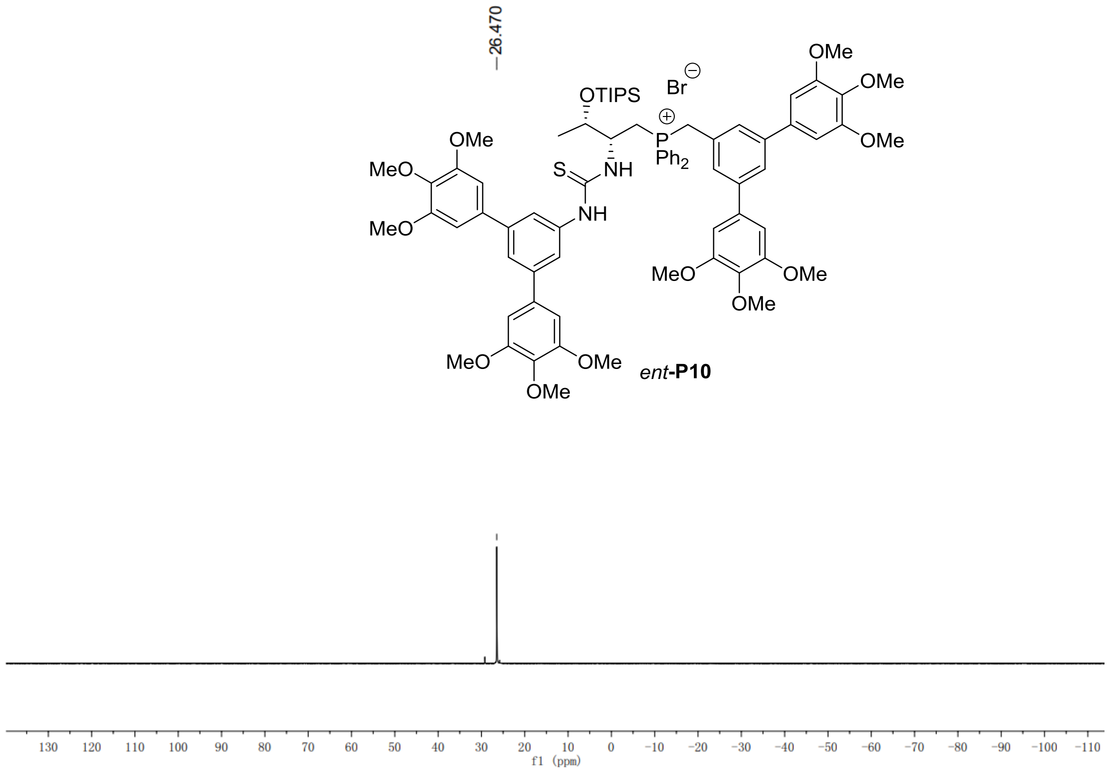


$^{31}$P-NMR of *ent*-**P10**

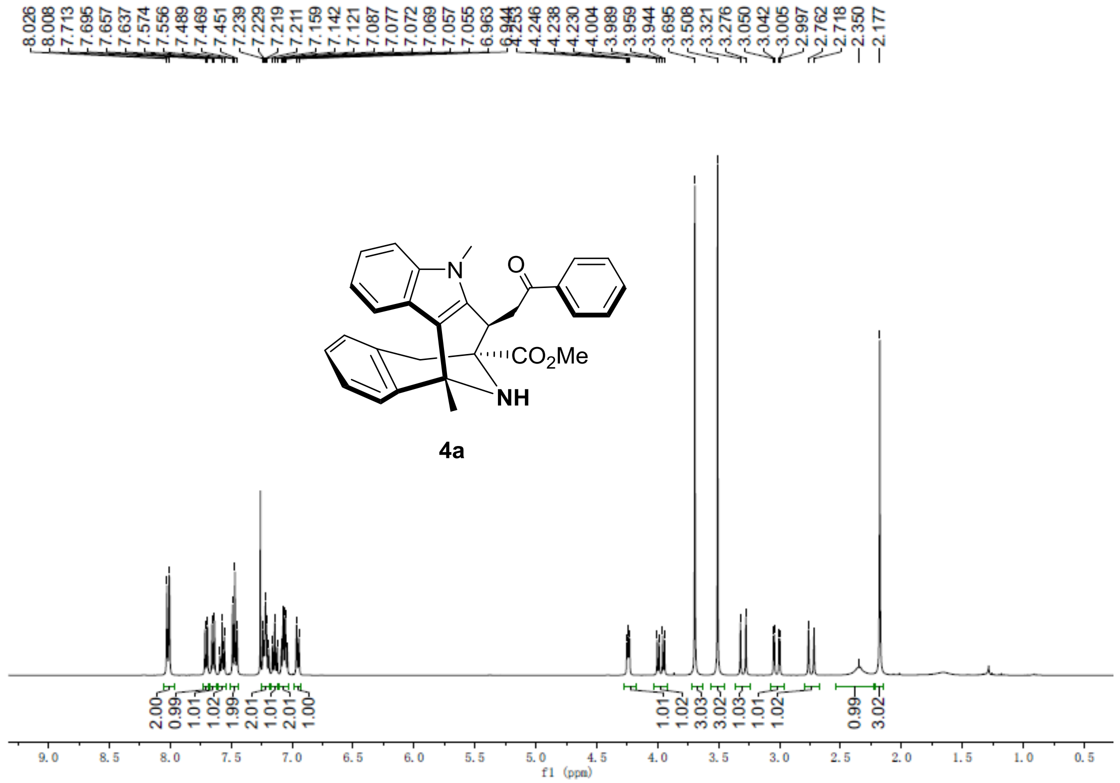

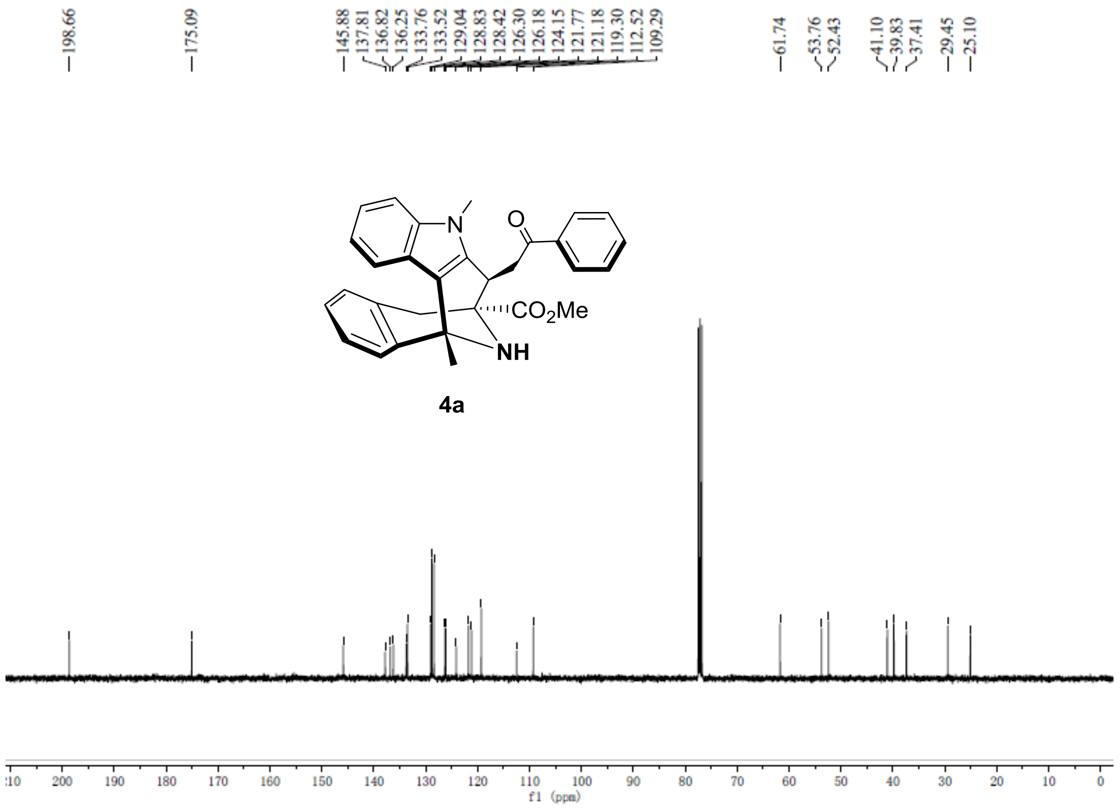

$^{1}$H-NMR and $^{13}$C-NMR of **4a**

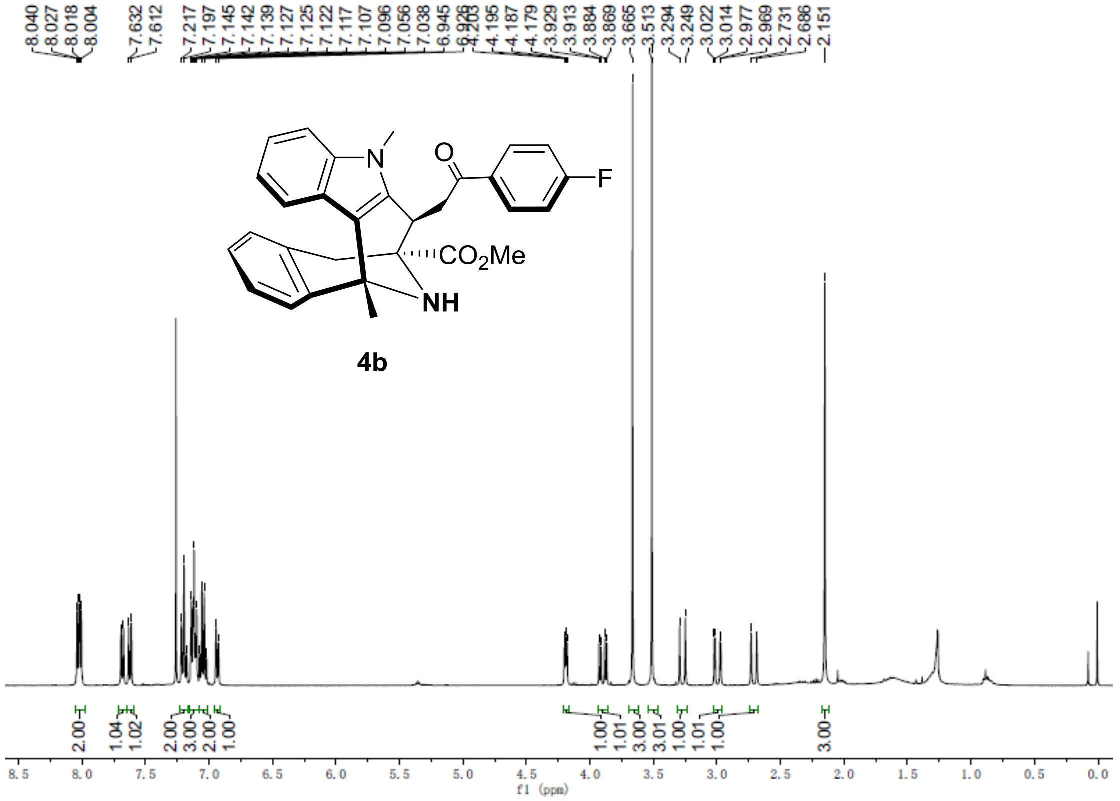

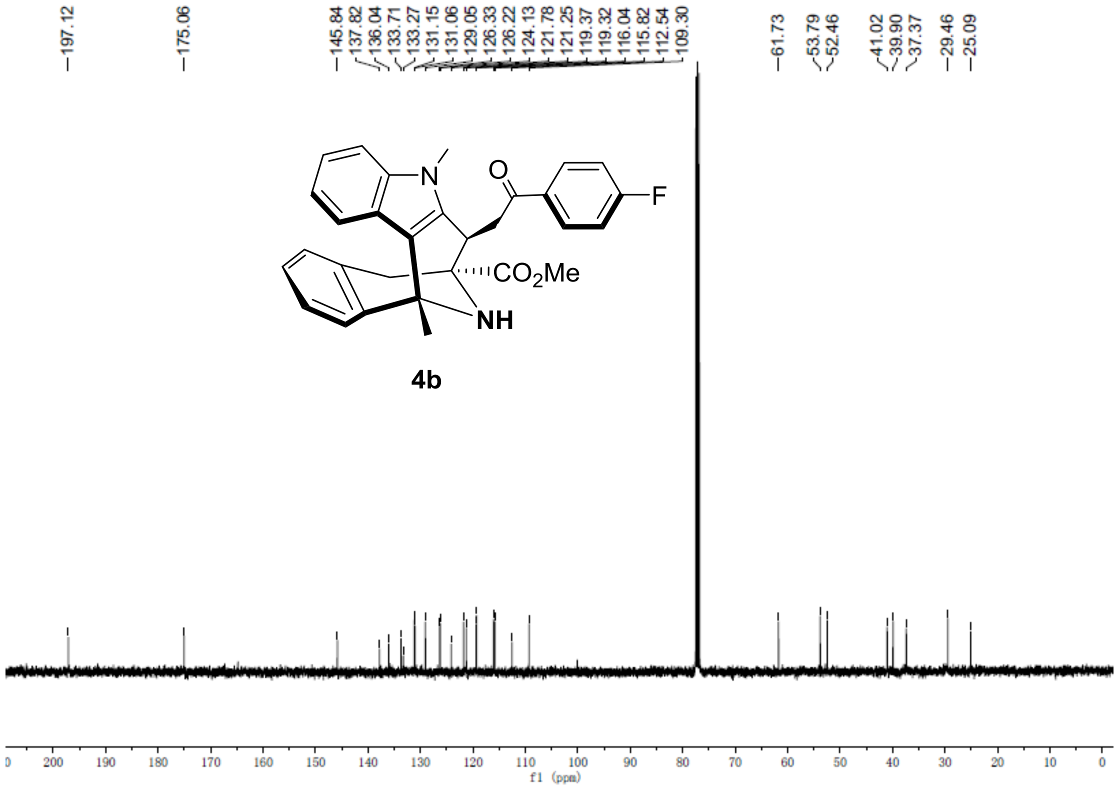

$^{1}$H-NMR and $^{13}$C-NMR of **4b**

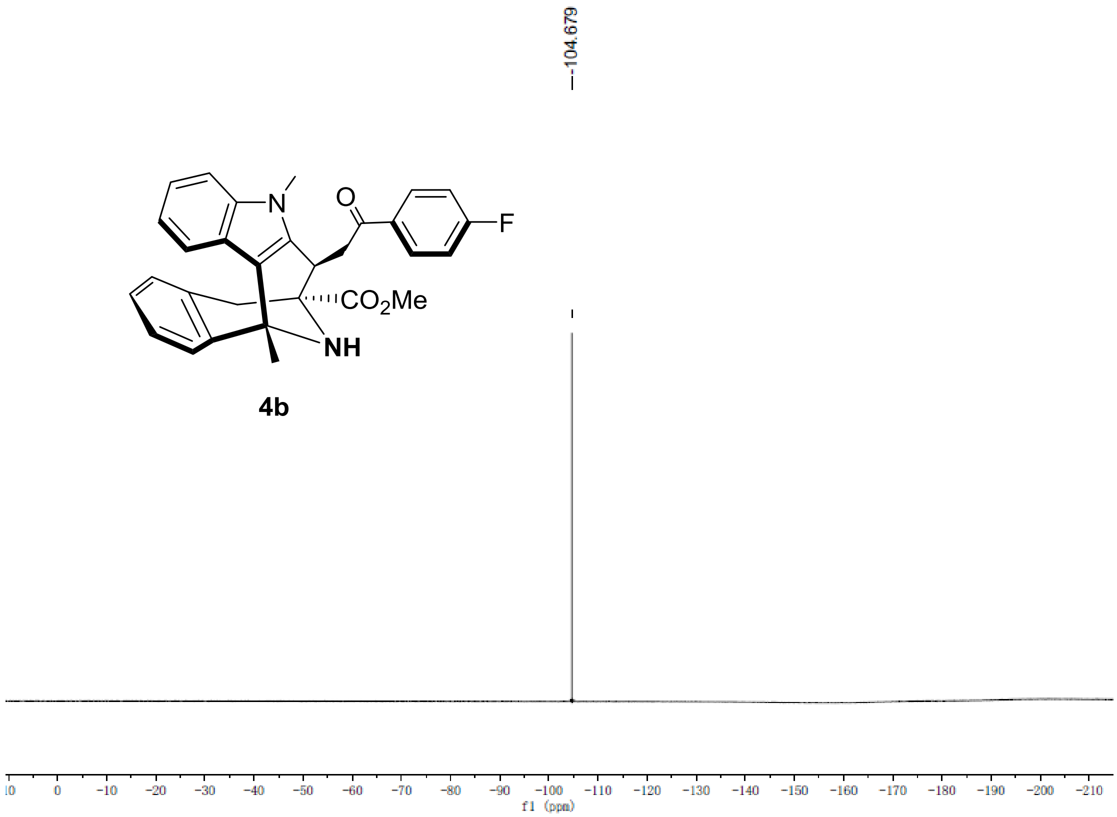

$^{19}$F-NMR of **4b**

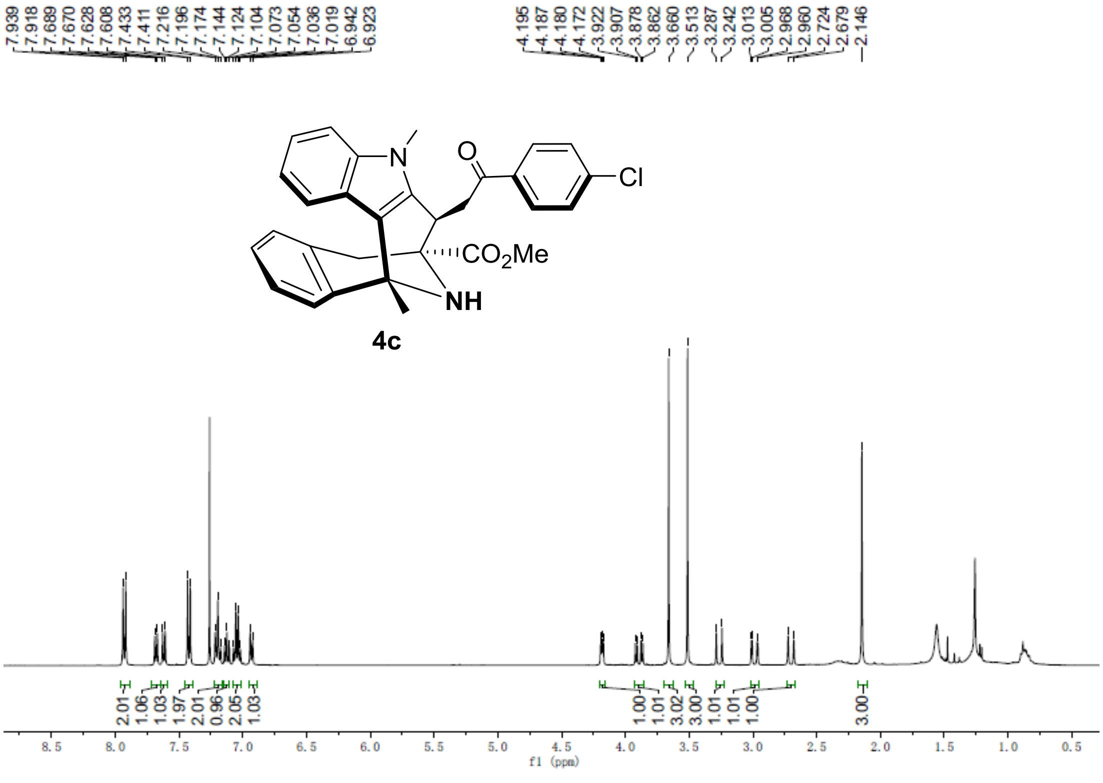

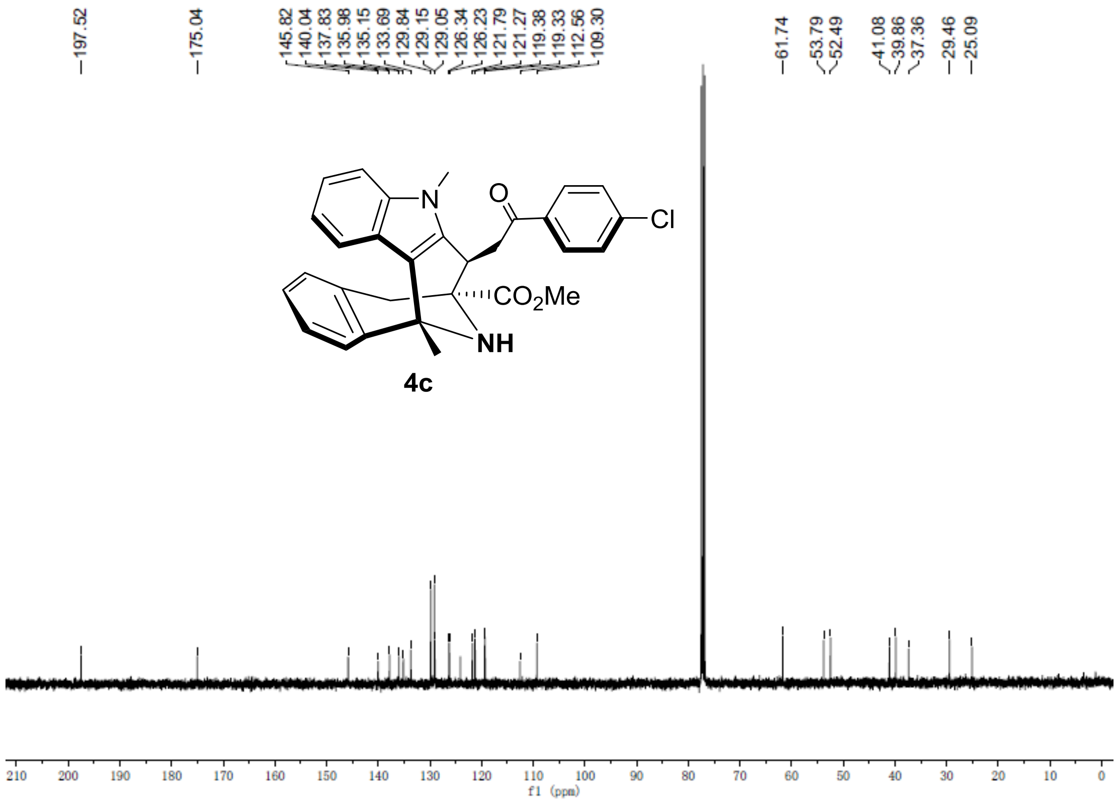

$^1$H-NMR and $^{13}$C-NMR of **4c**

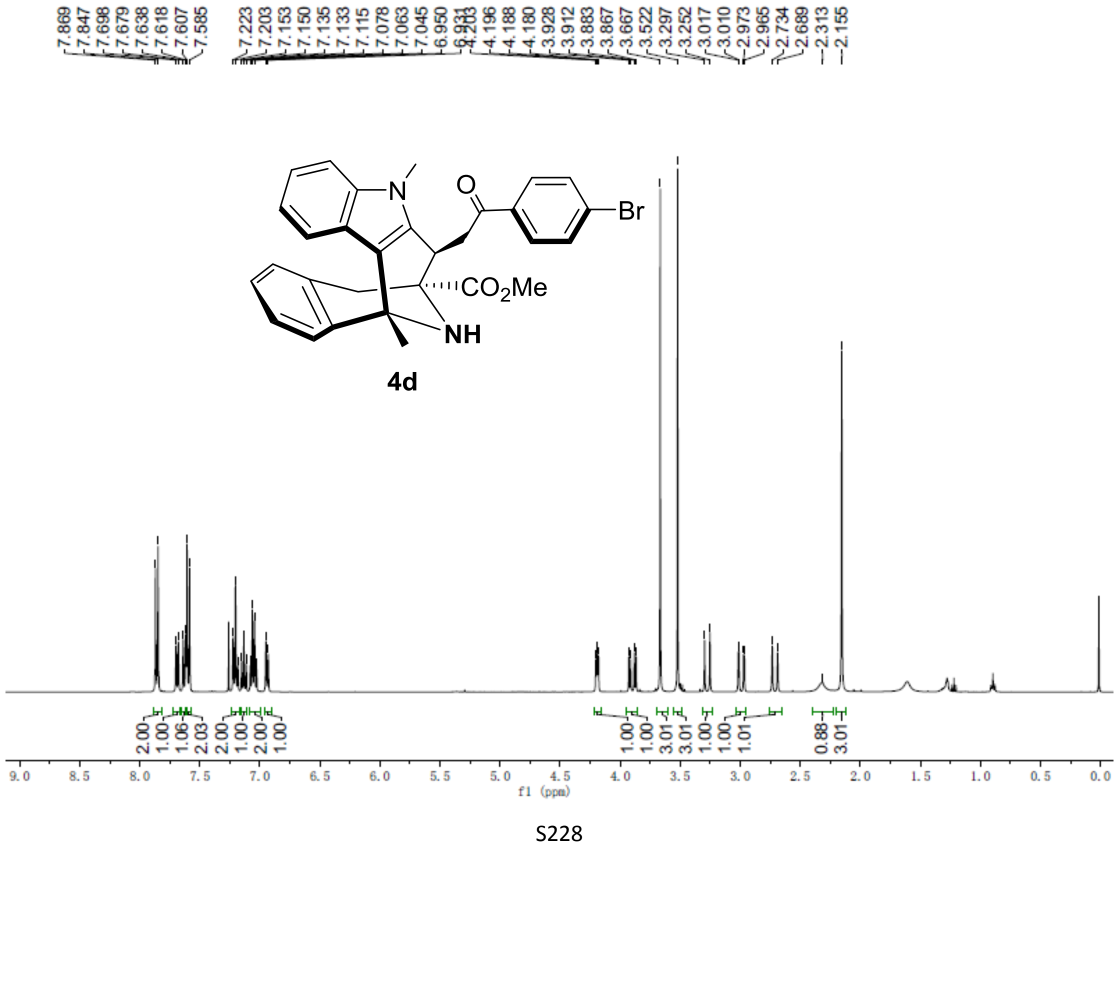

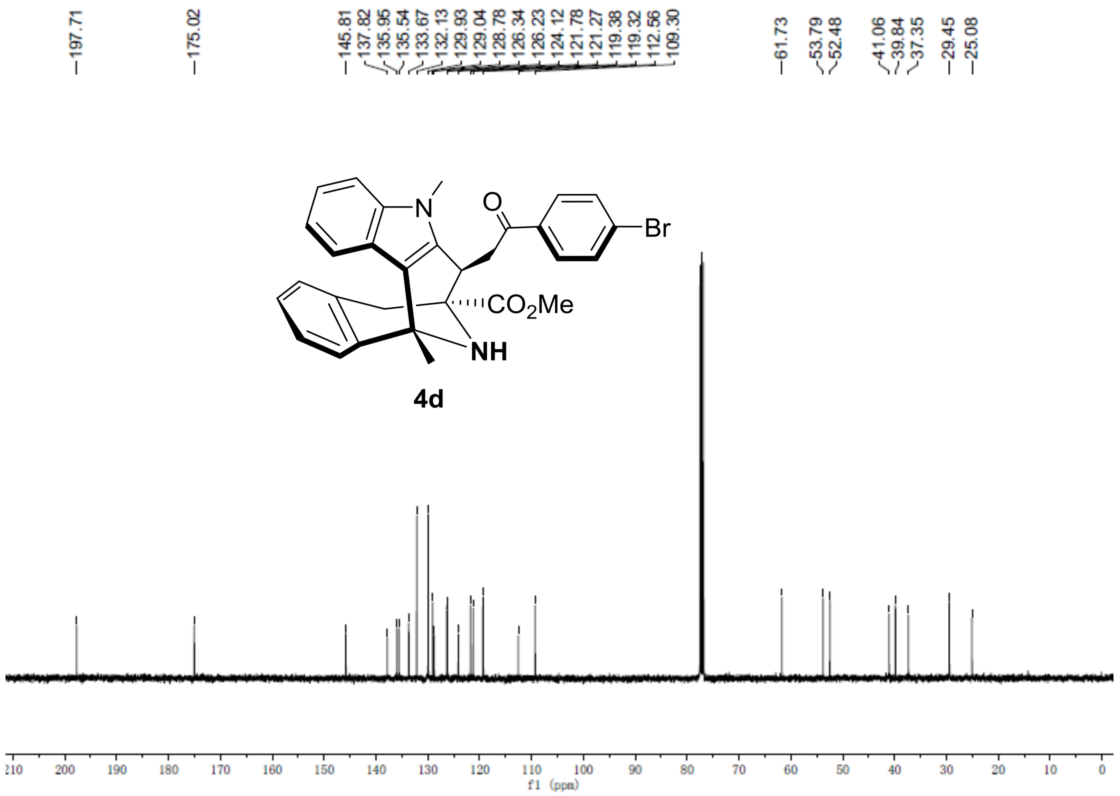


$^1$H-NMR and $^{13}$C-NMR of **4d**

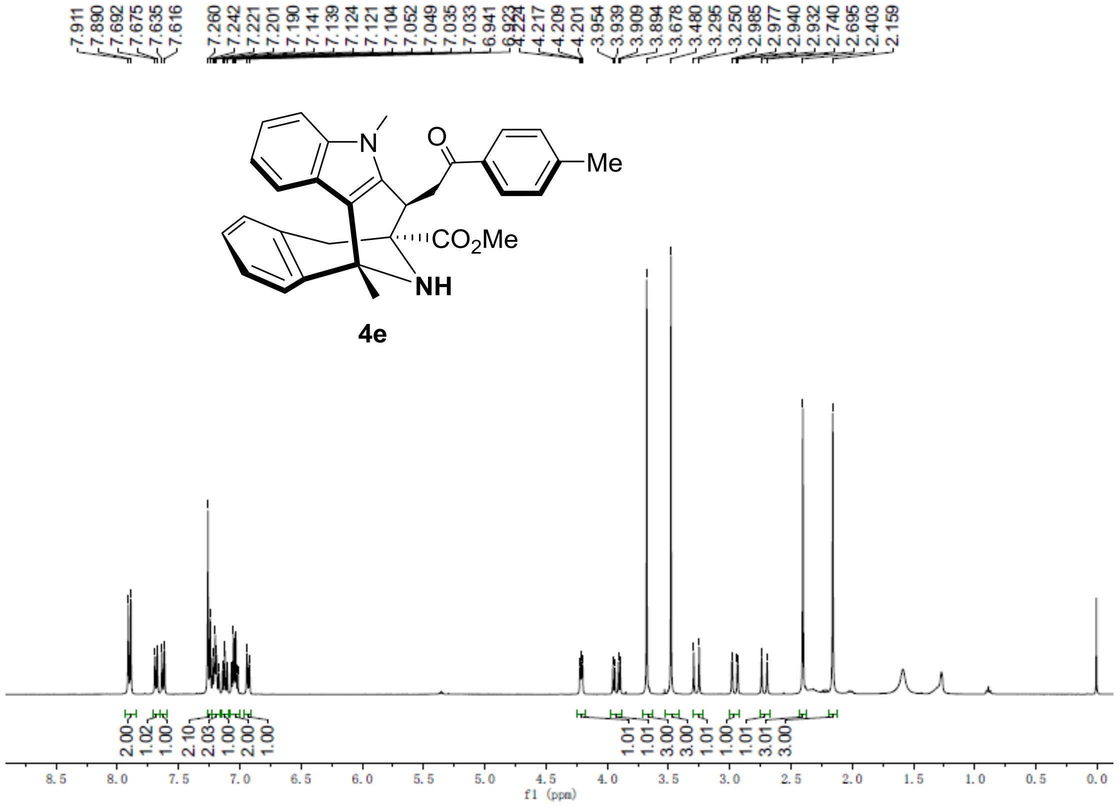

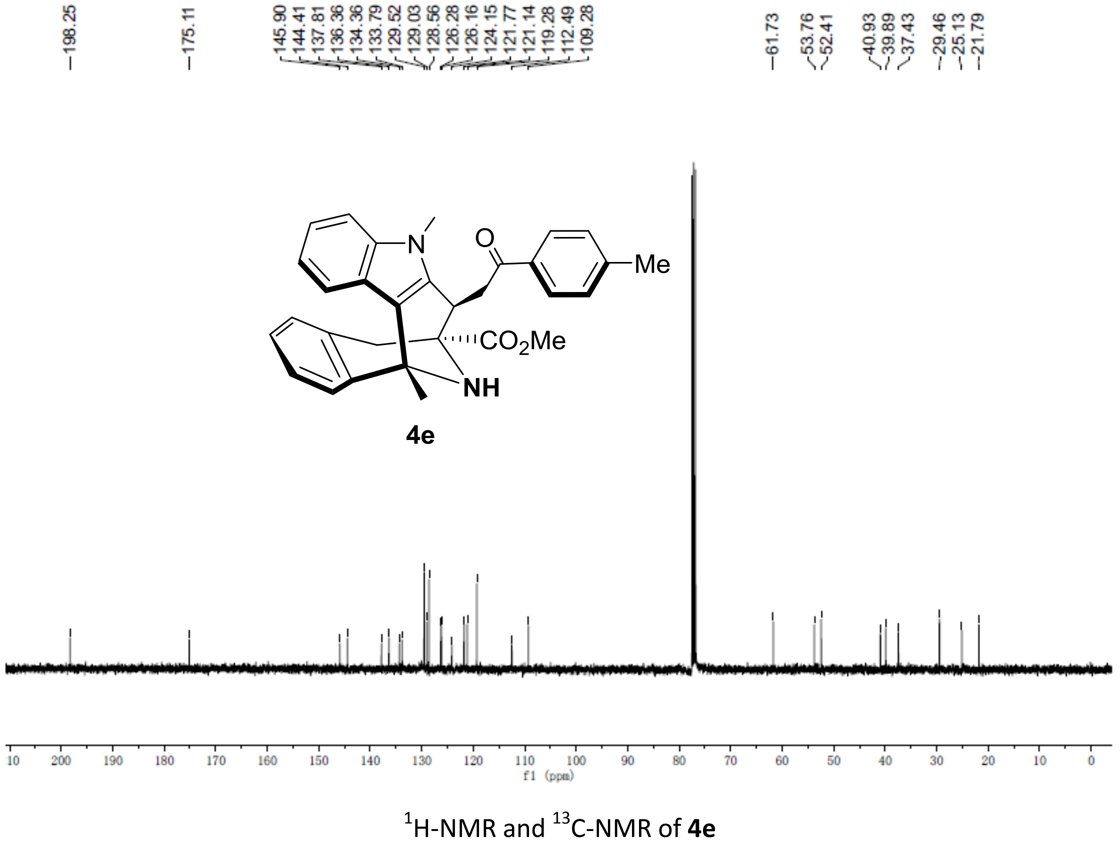

$^{1}$H-NMR and $^{13}$C-NMR of **4e**

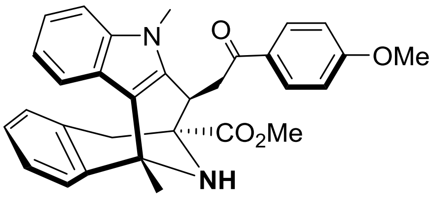

**4f**

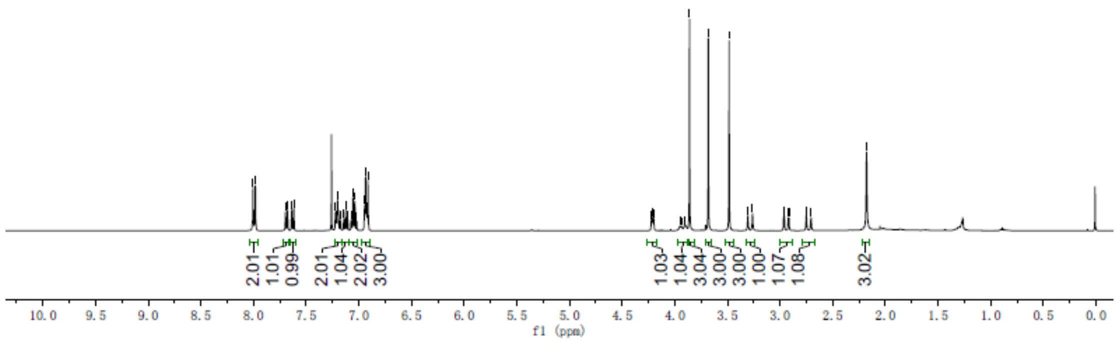


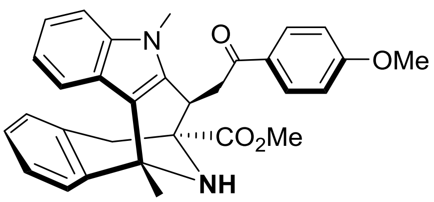

**4f**

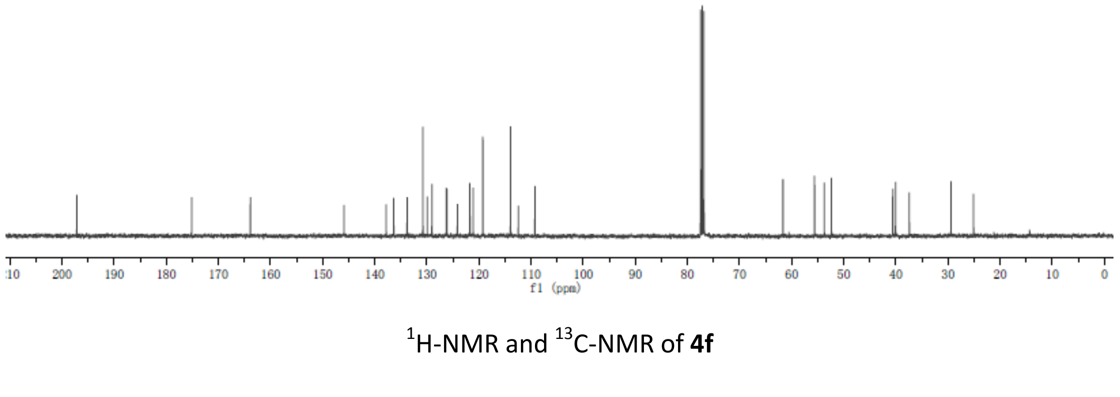


$^1$H-NMR and $^{13}$C-NMR of **4f**

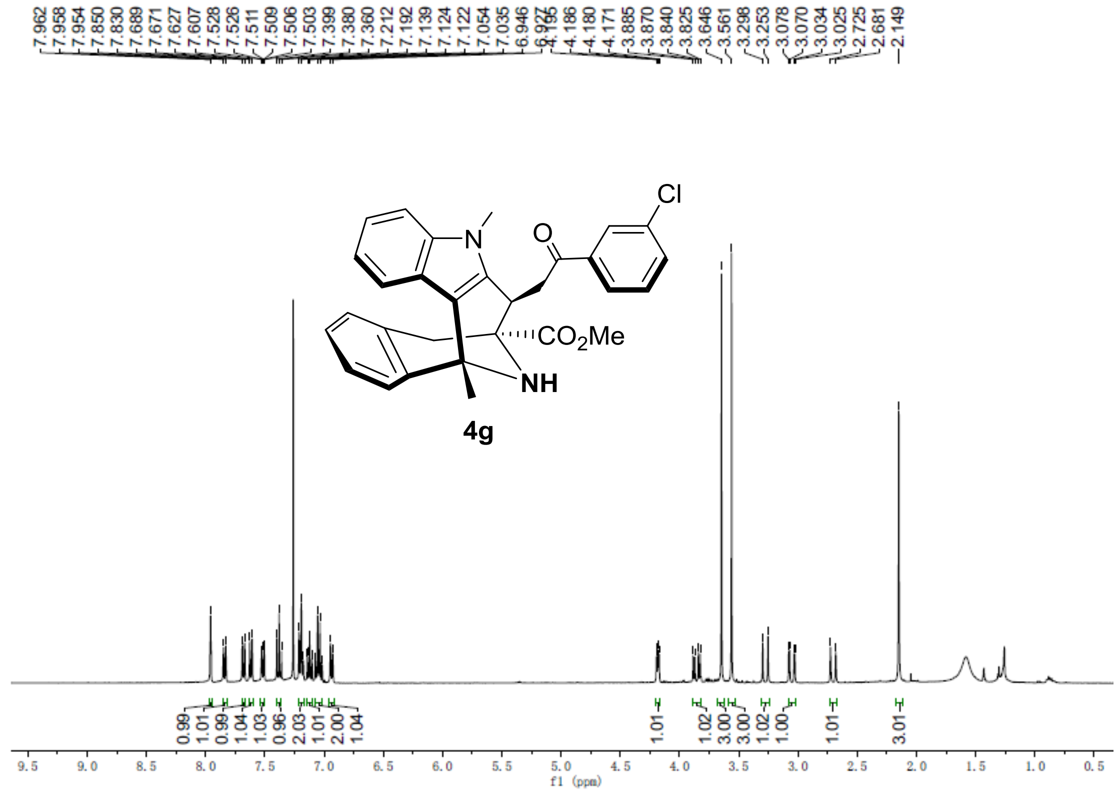

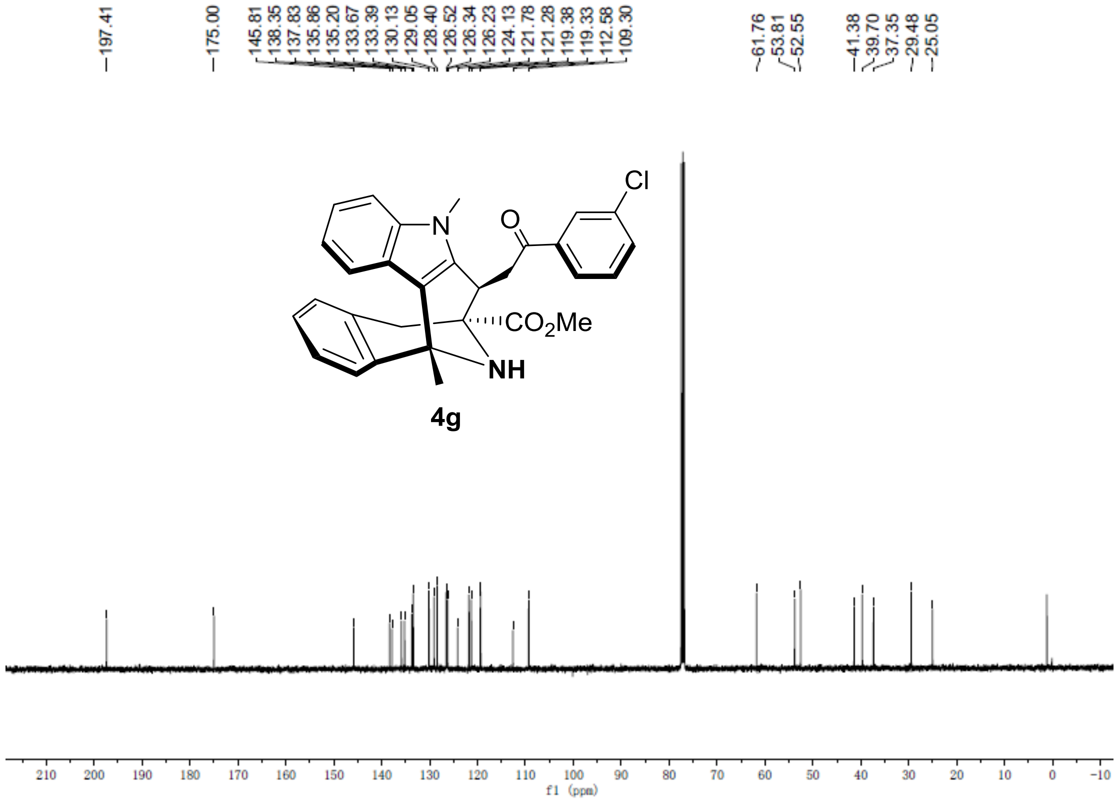

$^{1}$H-NMR and $^{13}$C-NMR of **4g**

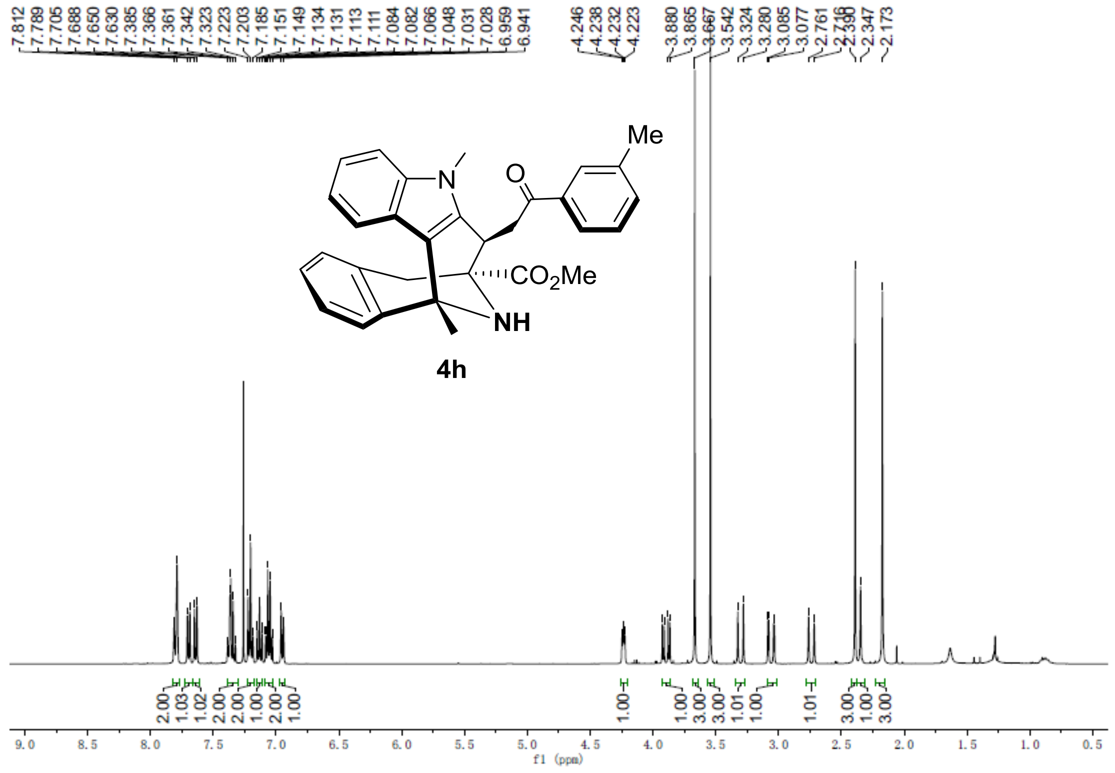

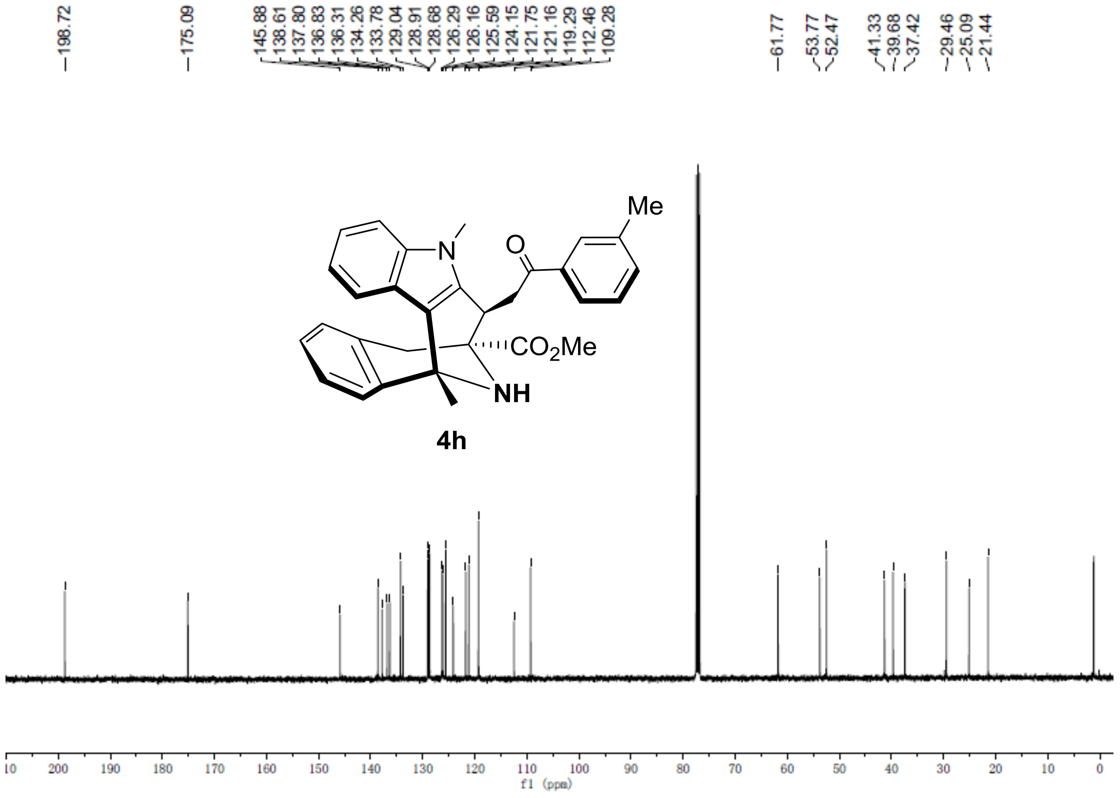

$^{1}$H-NMR and $^{13}$C-NMR of **4h**

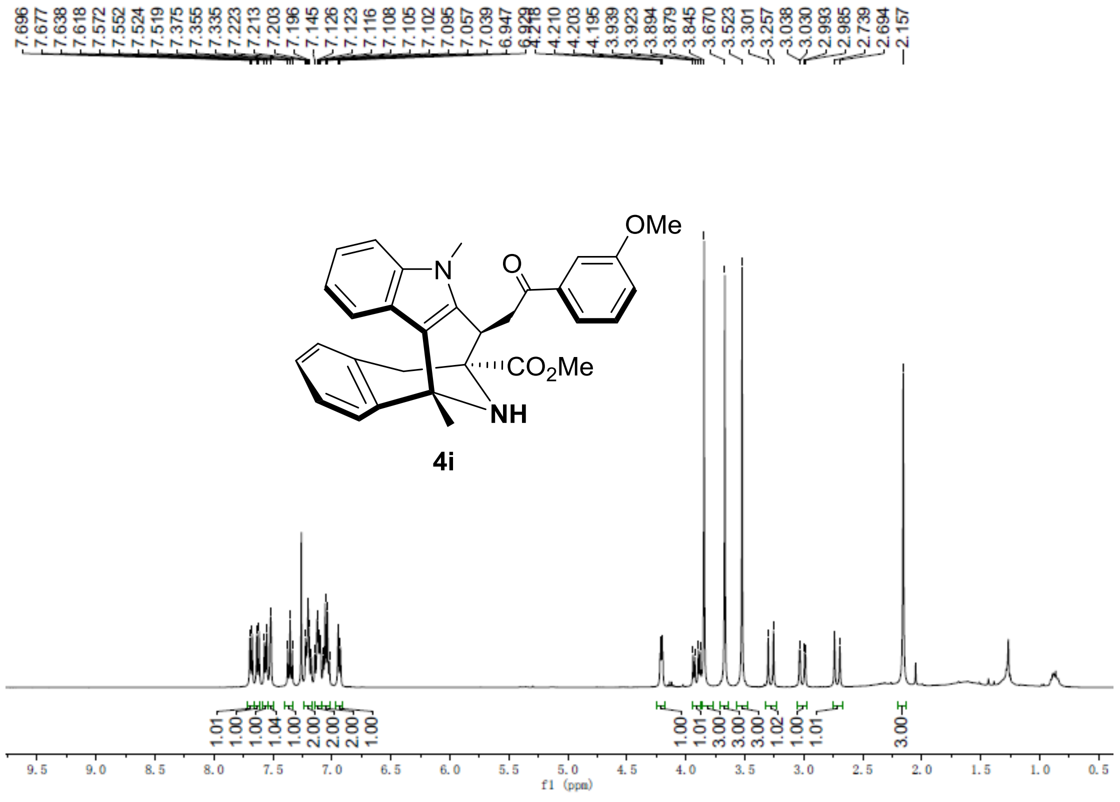

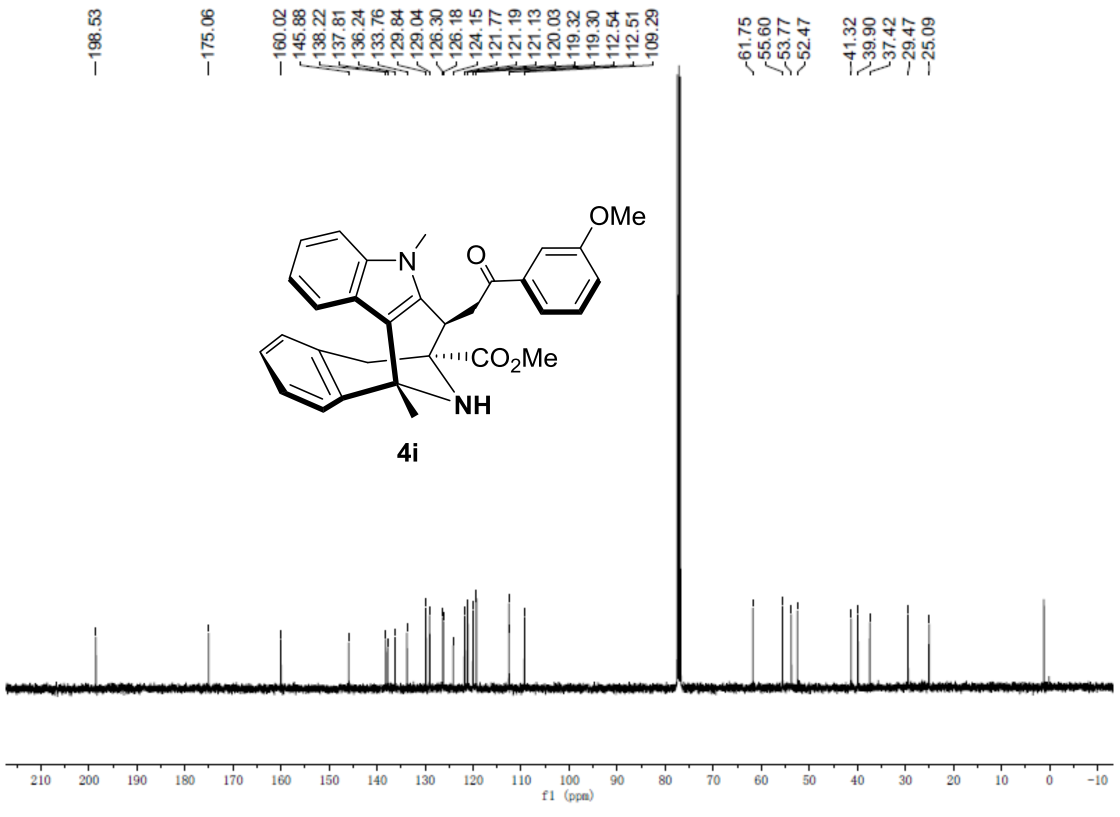

$^{1}$H-NMR and $^{13}$C-NMR of **4i**

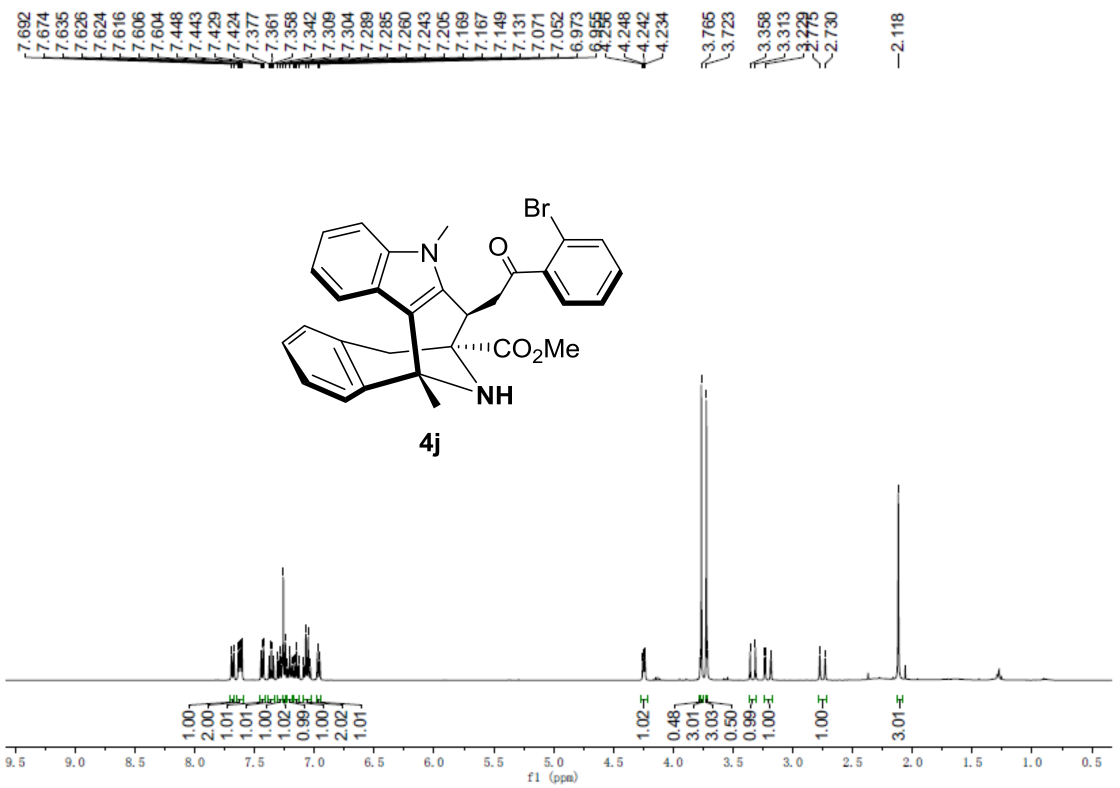


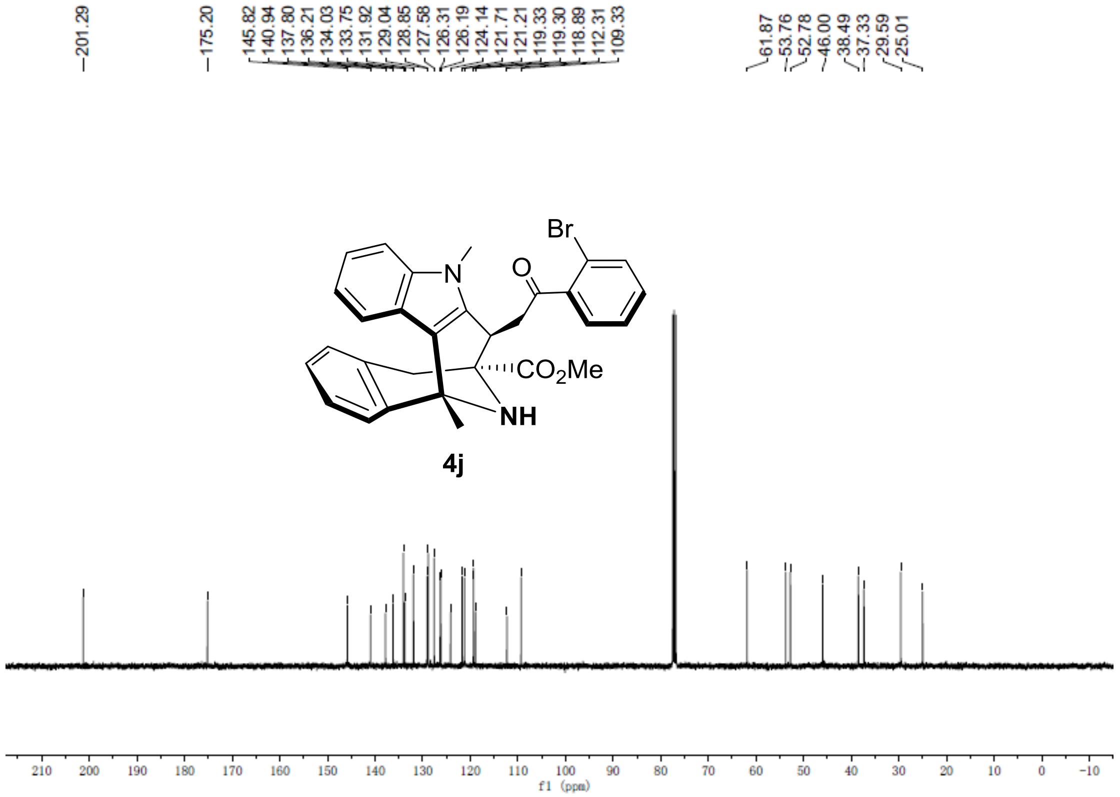


$^{1}$H-NMR and $^{13}$C-NMR of **4j**

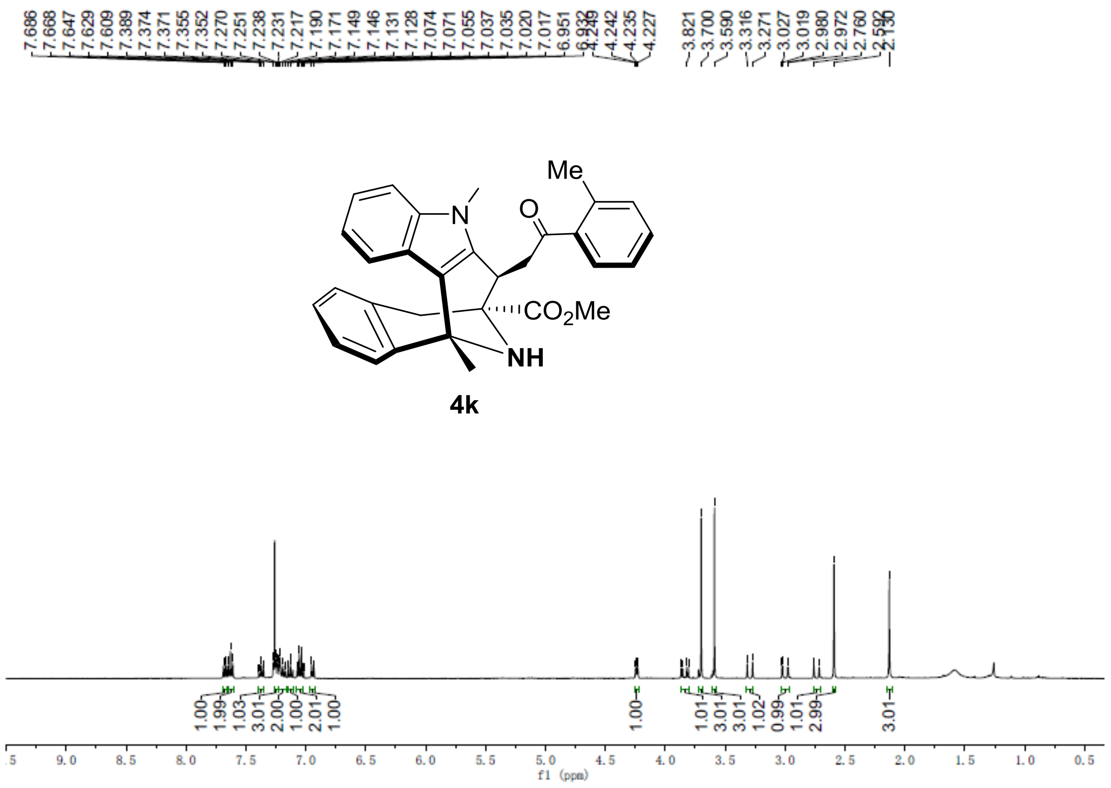

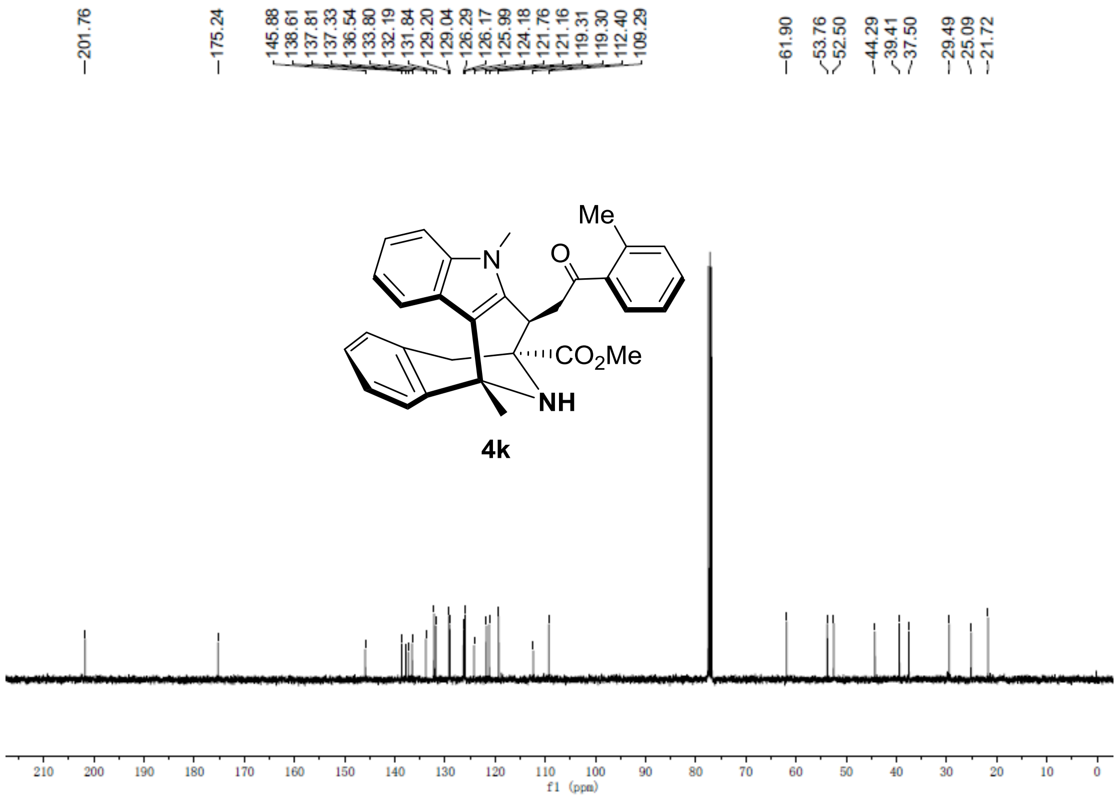

$^{1}$H-NMR and $^{13}$C-NMR of **4k**

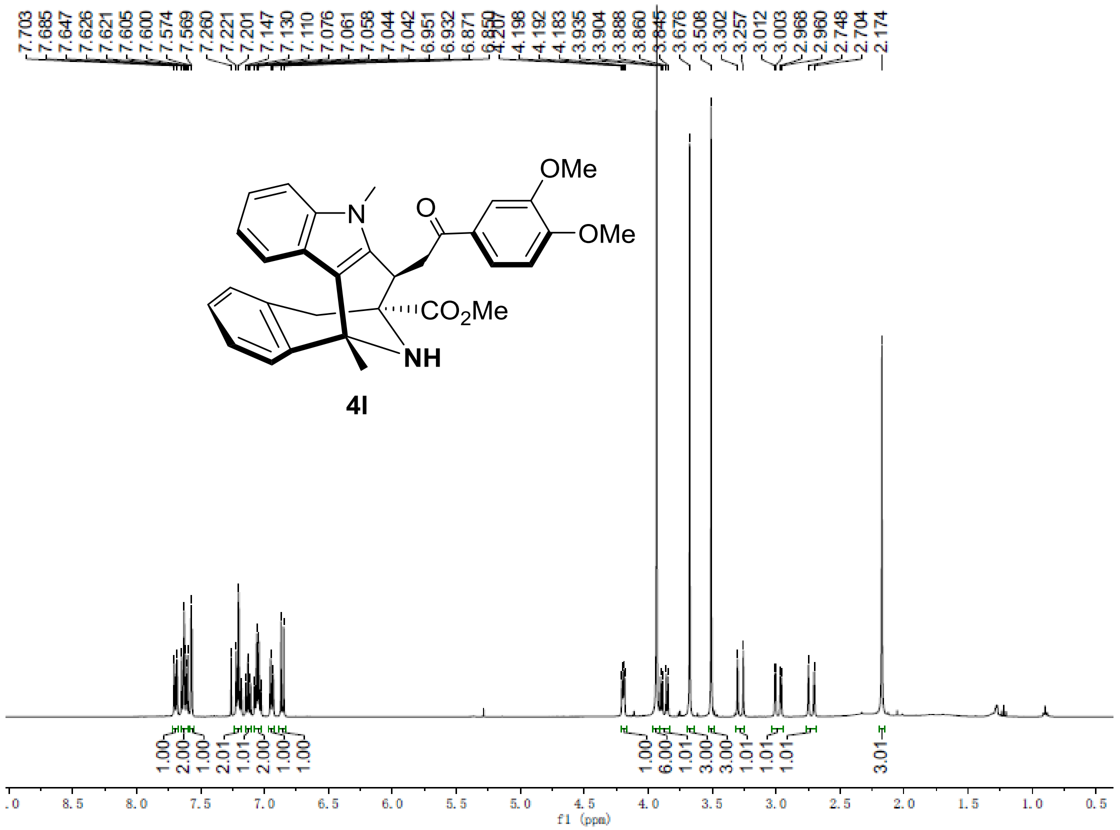


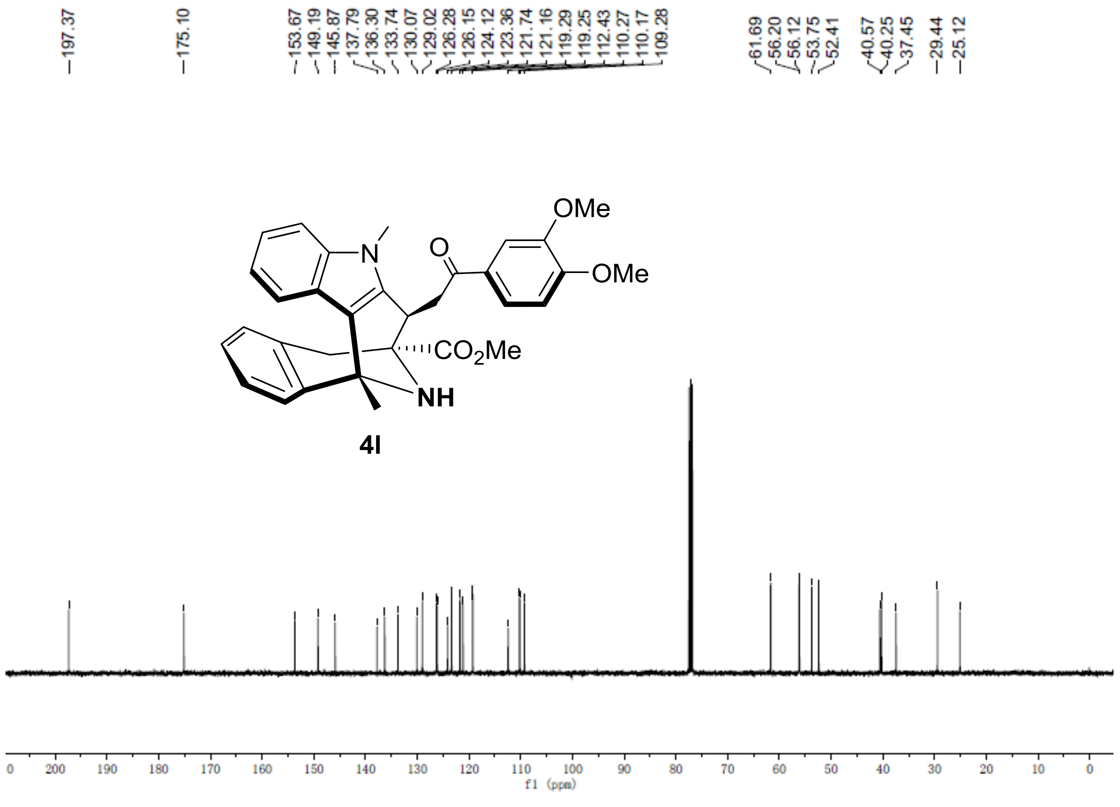


$^{1}$H-NMR and $^{13}$C-NMR of **4l**

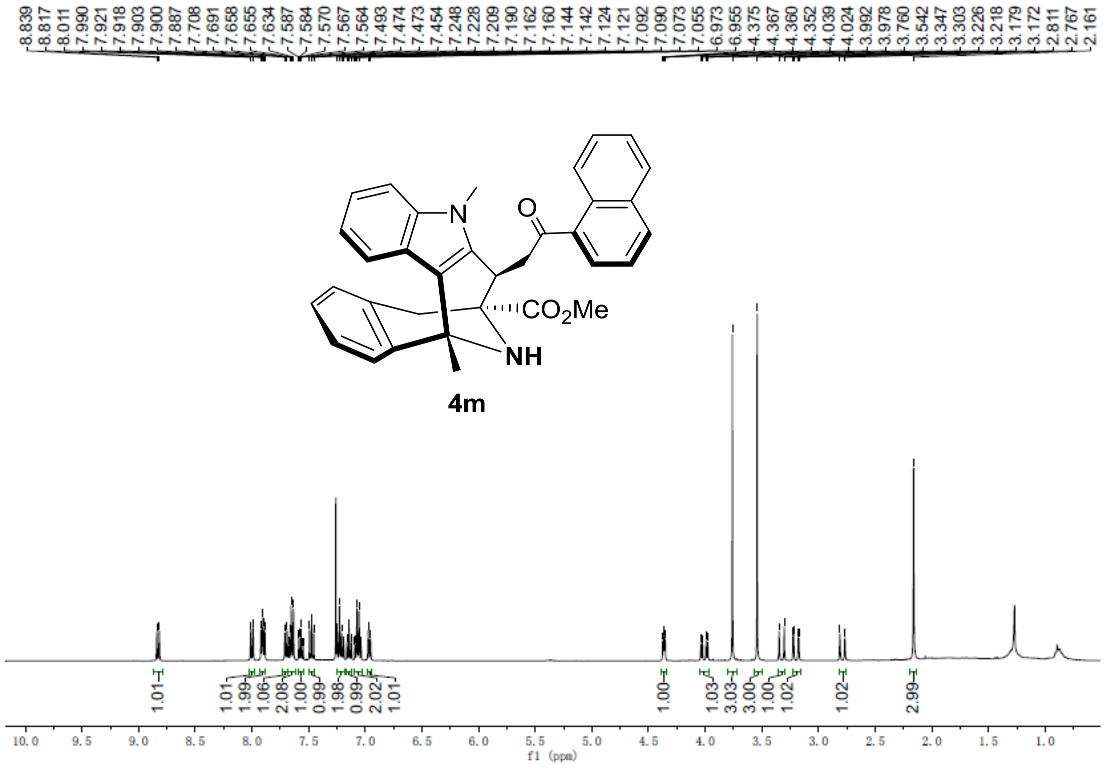


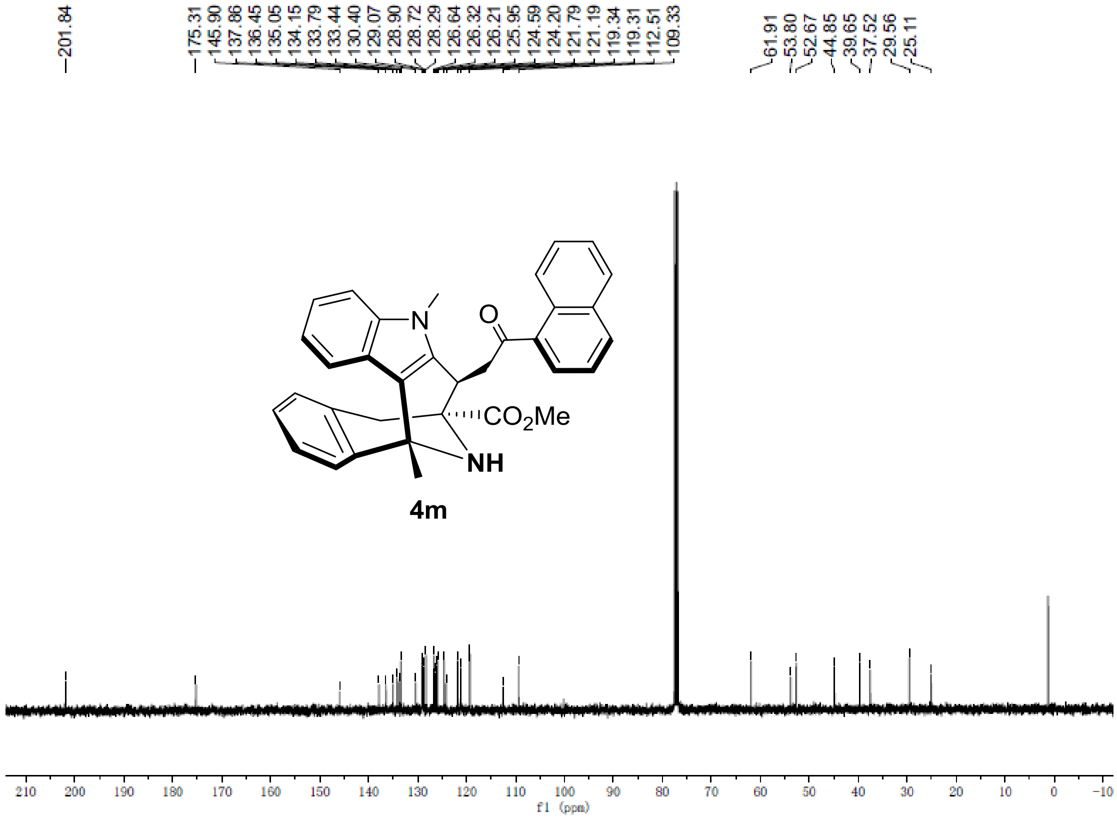


$^{1}$H-NMR and $^{13}$C-NMR of **4m**

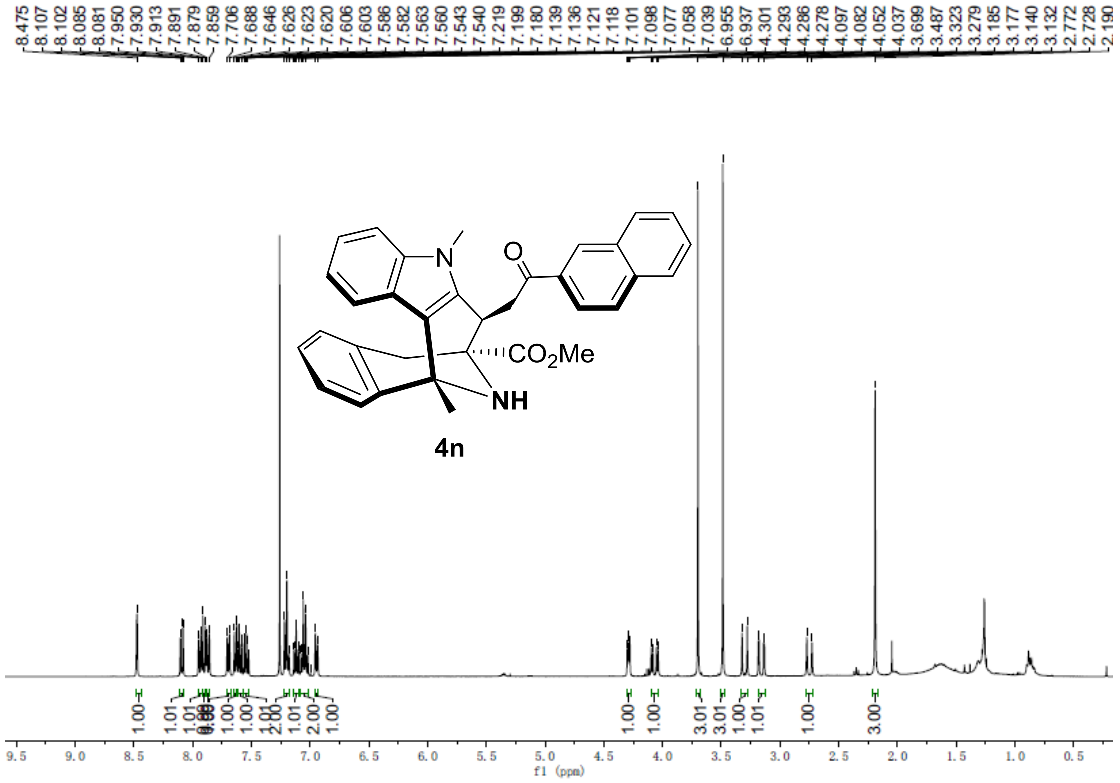

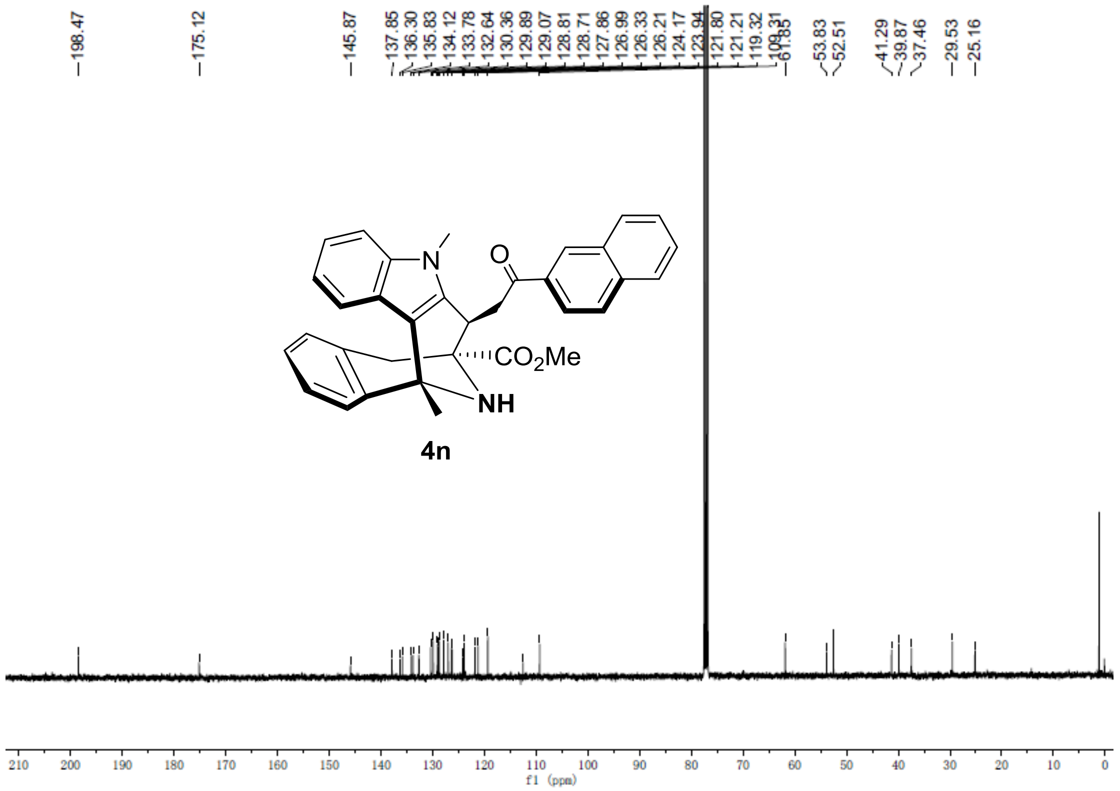

$^{1}$H-NMR and $^{13}$C-NMR of **4n**

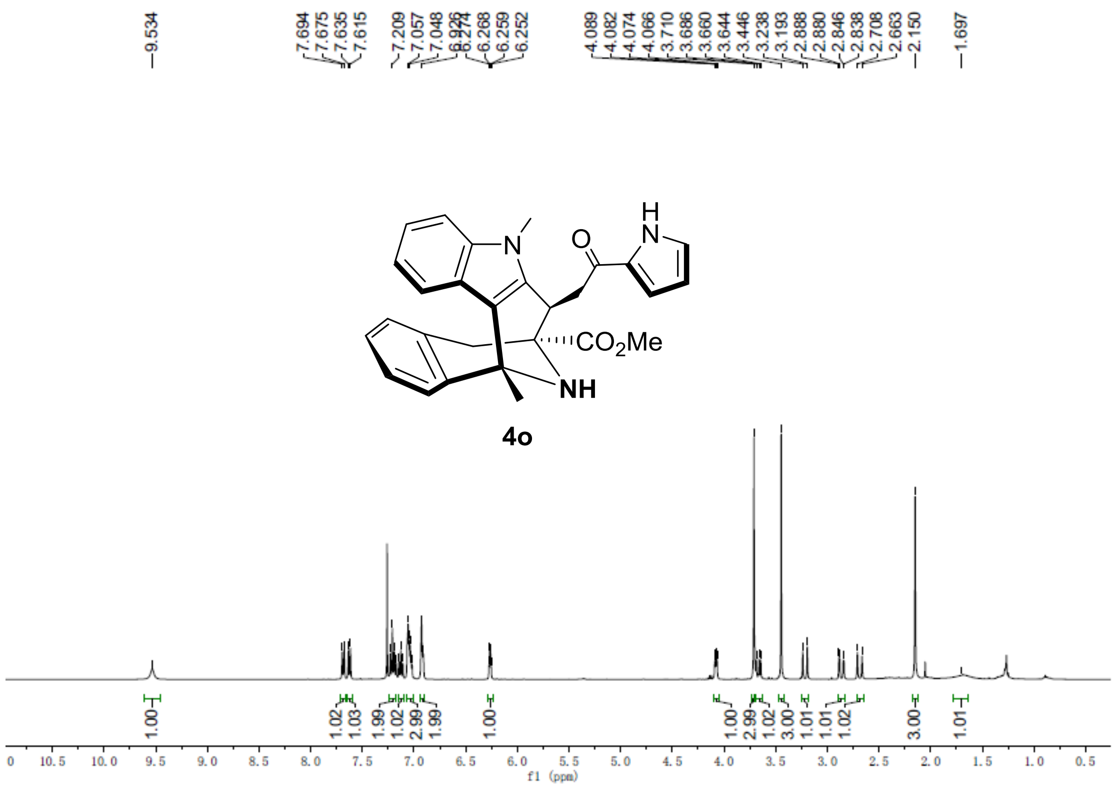

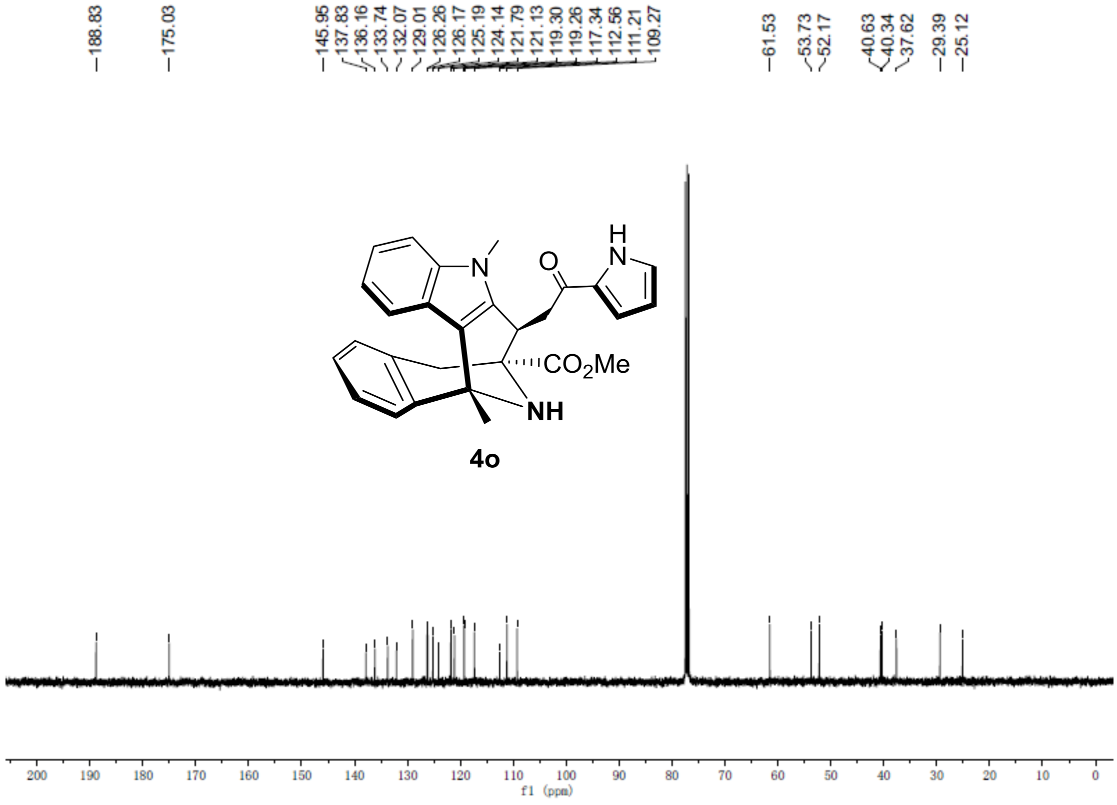

$^{1}$H-NMR and $^{13}$C-NMR of **4o**

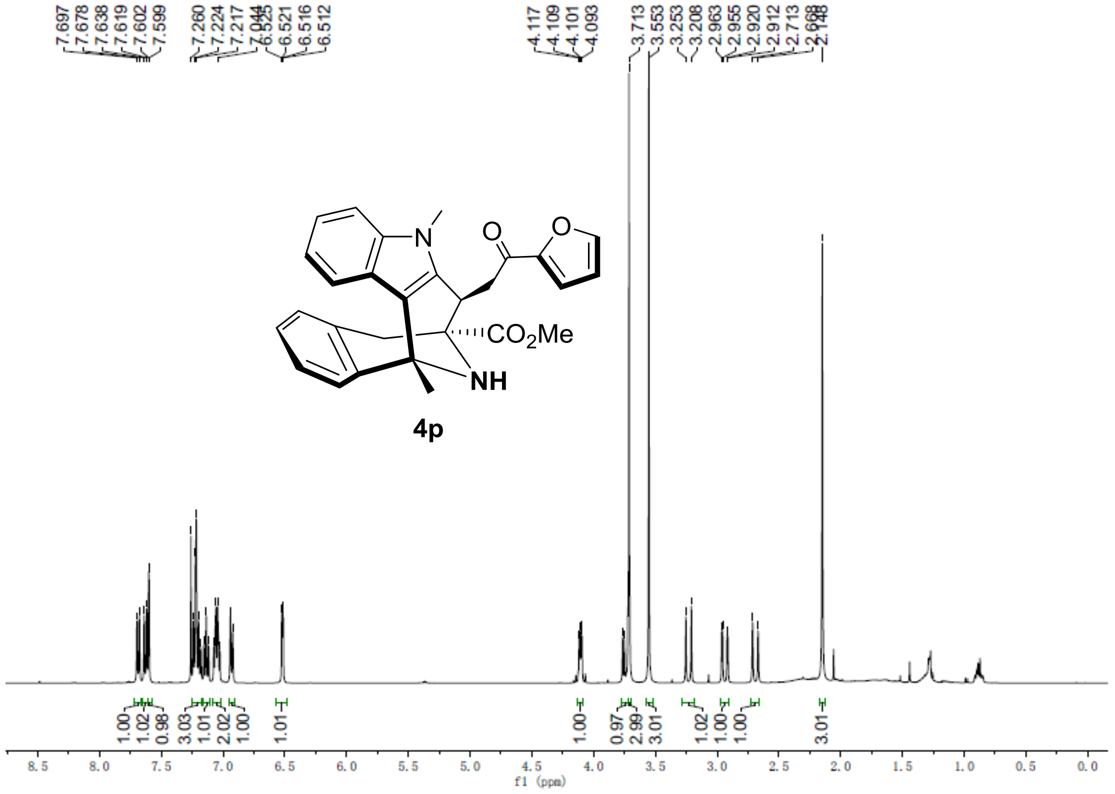


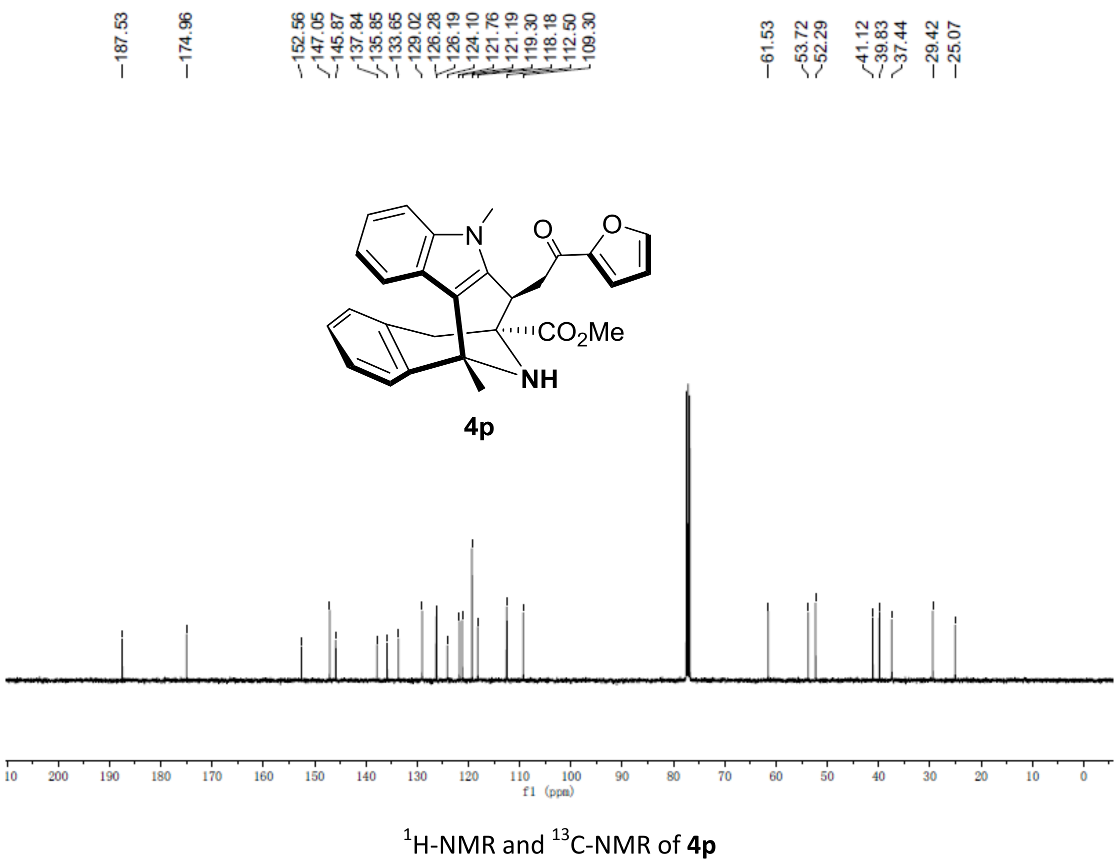


$^{1}$H-NMR and $^{13}$C-NMR of **4p**

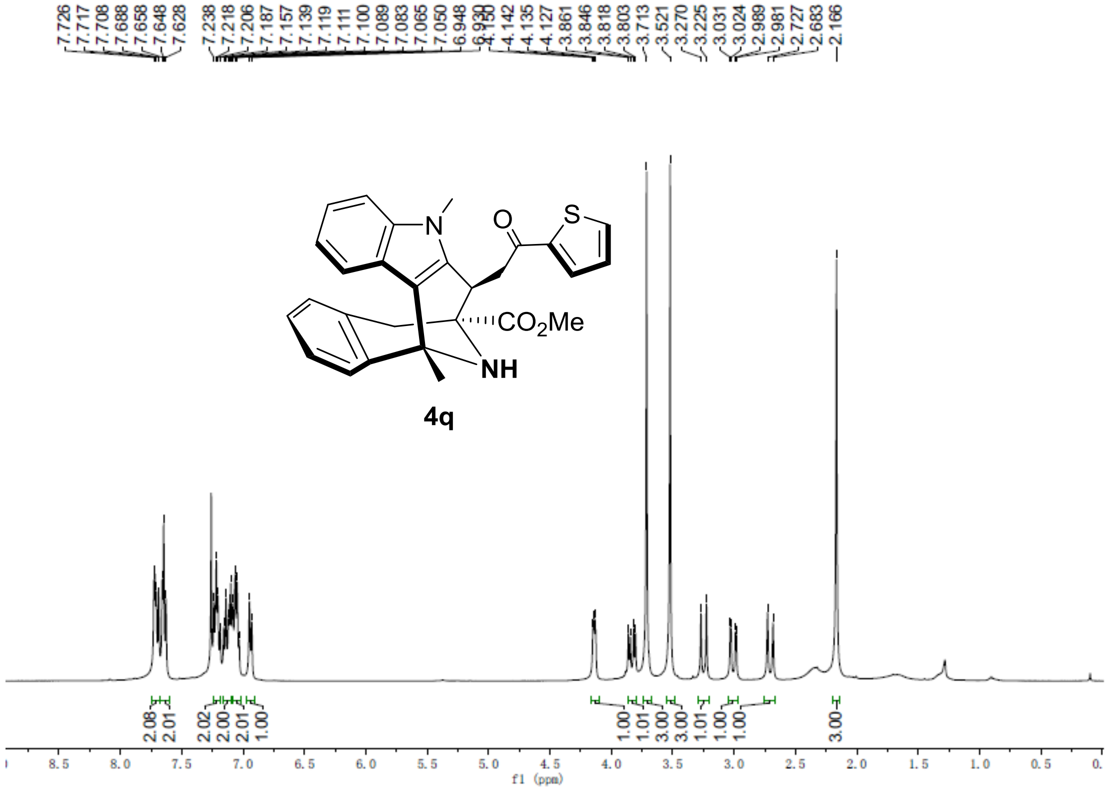


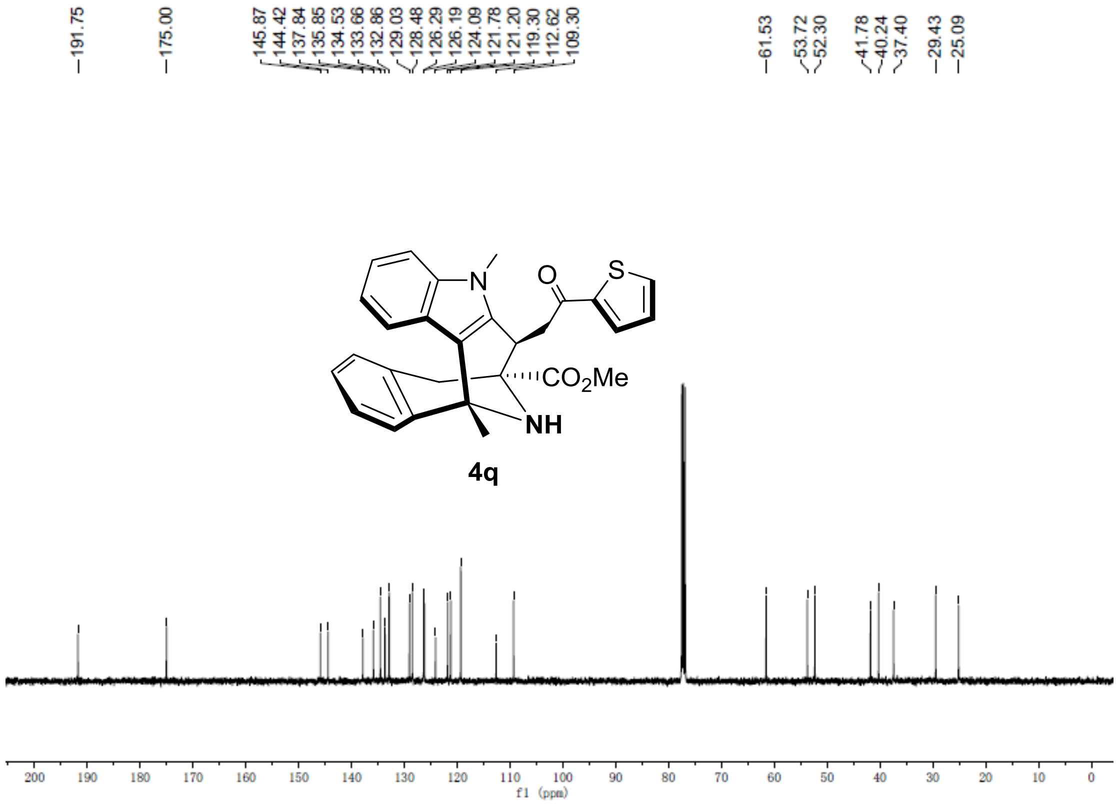


$^{1}$H-NMR and $^{13}$C-NMR of **4q**

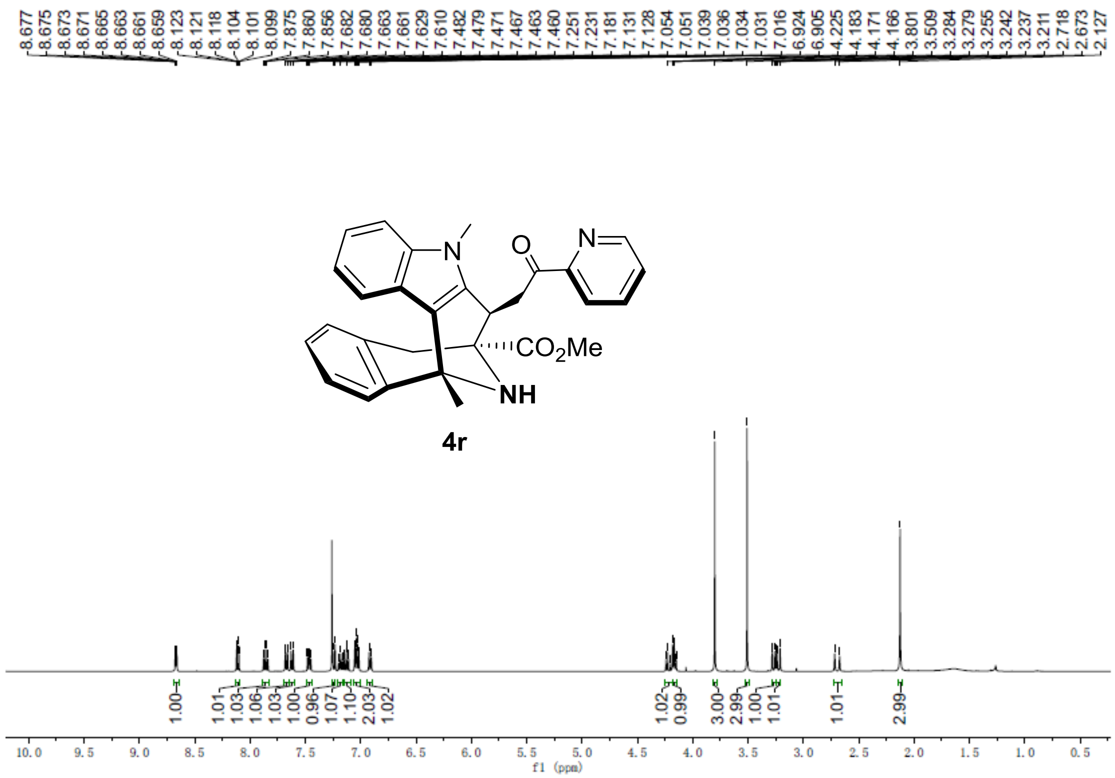

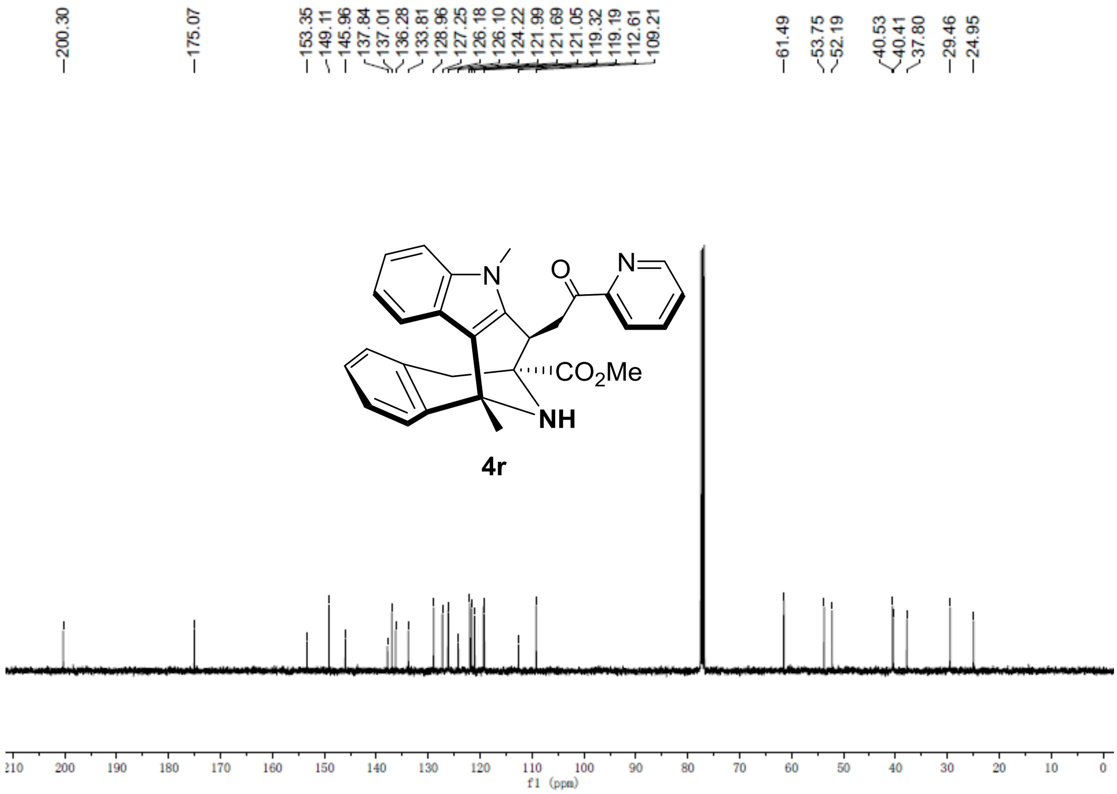

$^{1}$H-NMR and $^{13}$C-NMR of **4r**

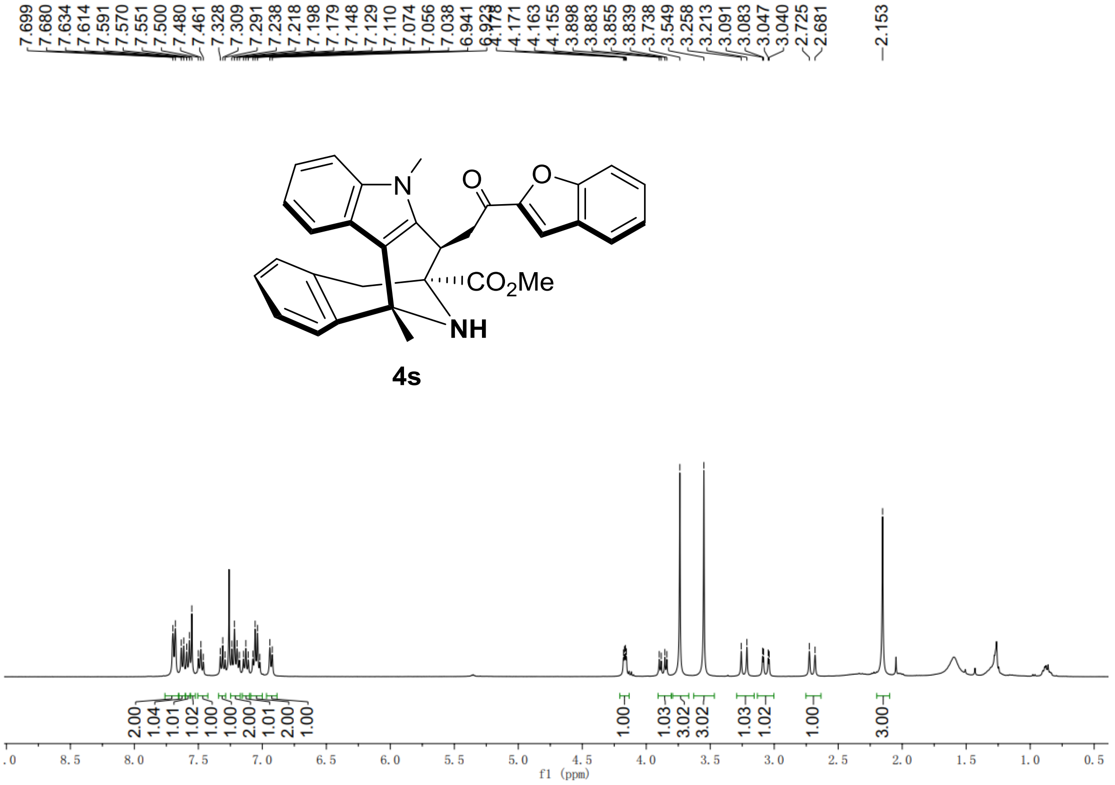


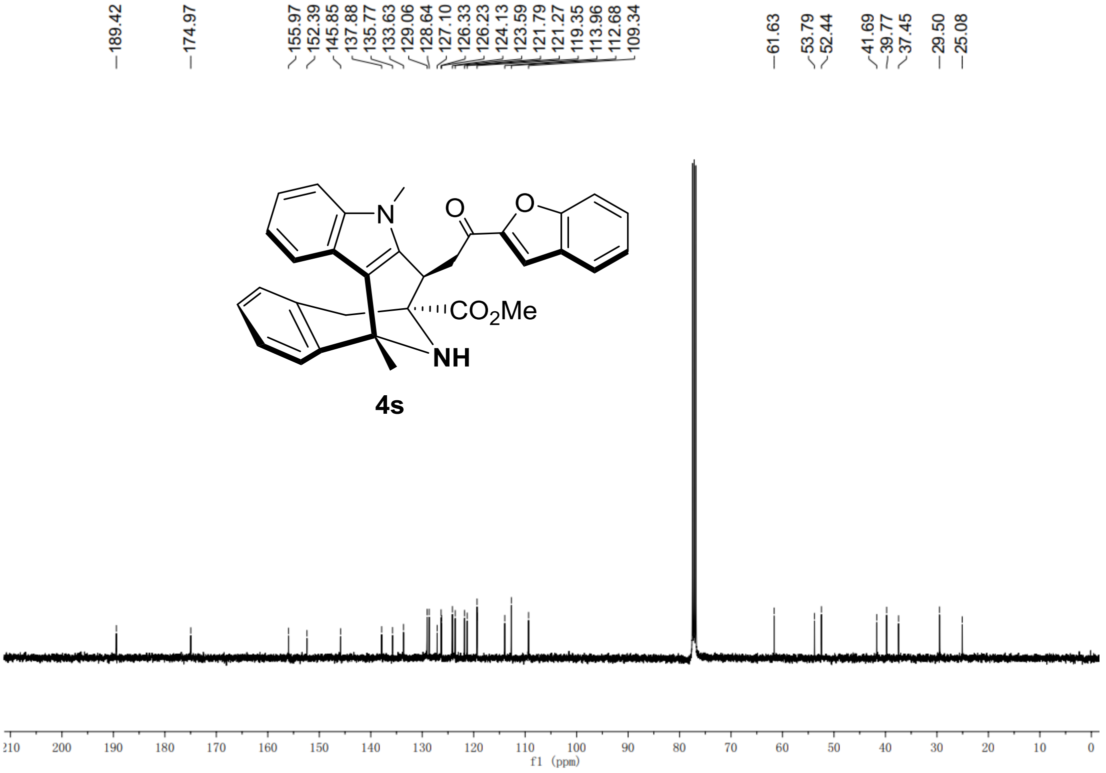


$^{1}$H-NMR and $^{13}$C-NMR of **4s**

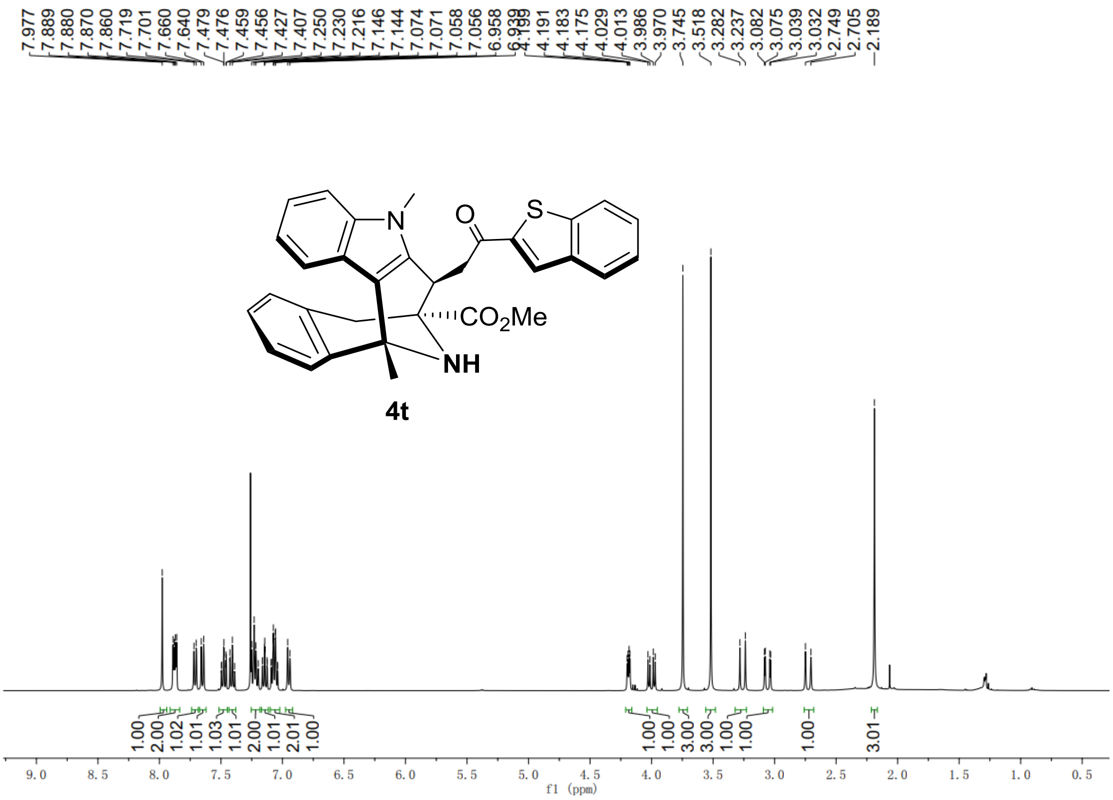


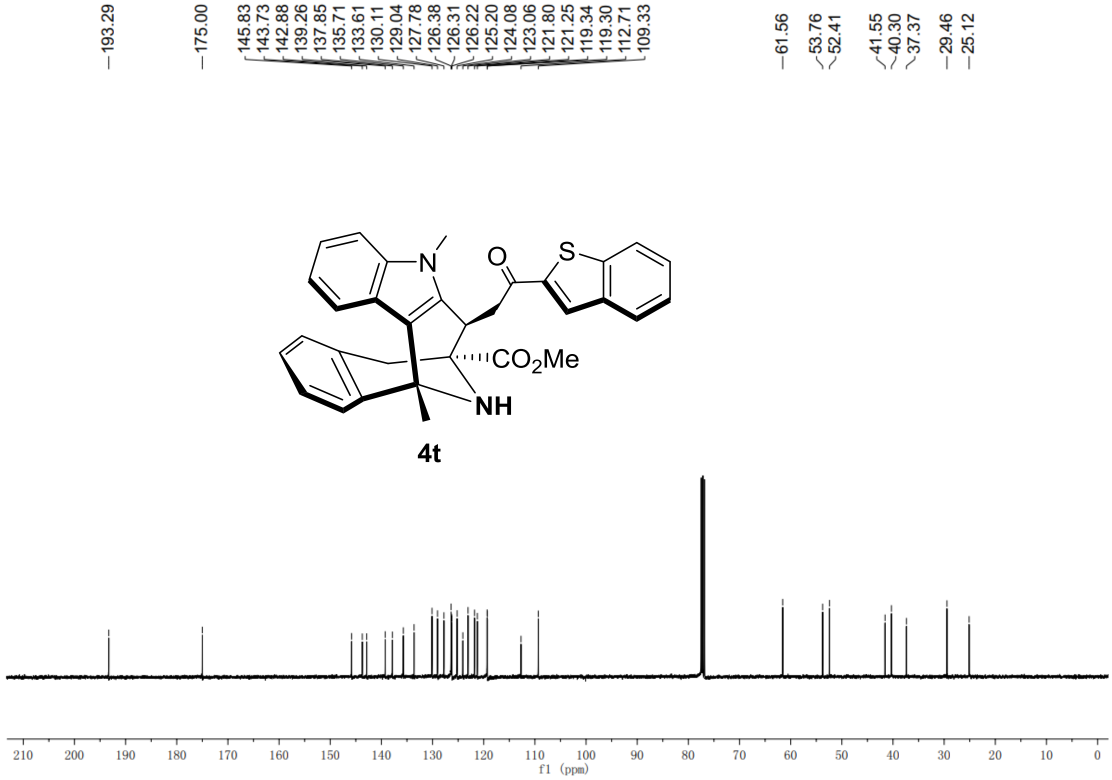


$^{1}$H-NMR and $^{13}$C-NMR of **4t**

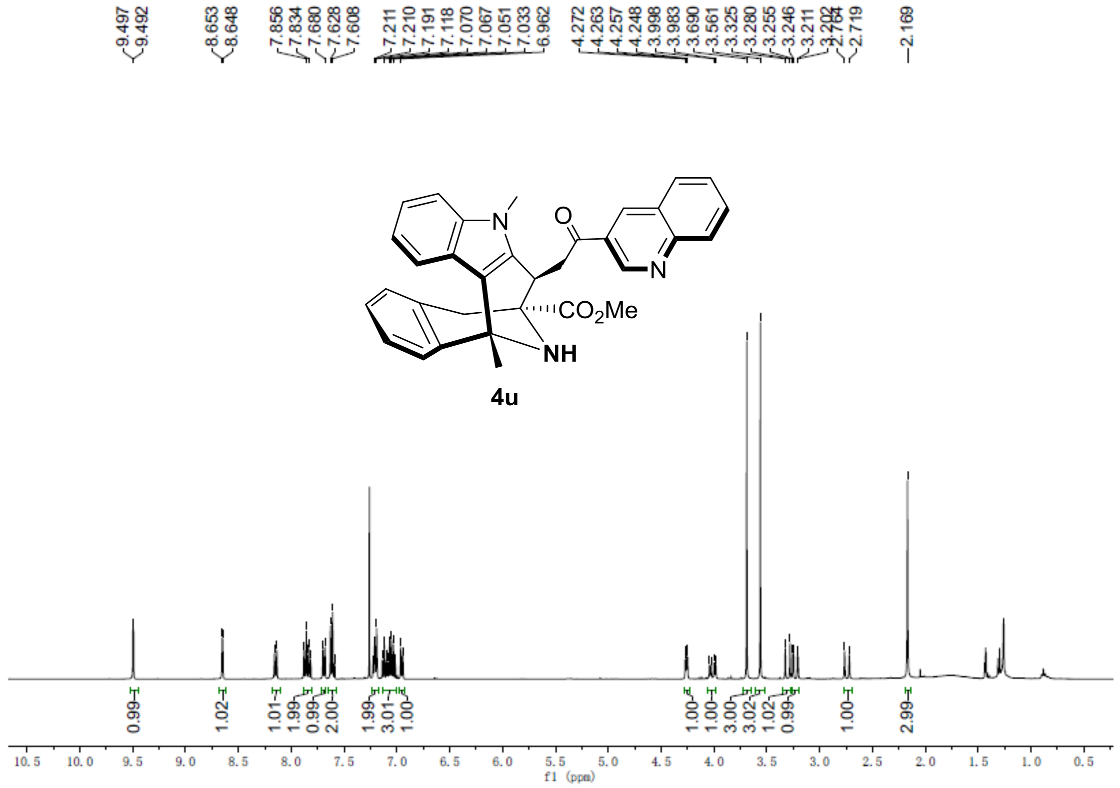


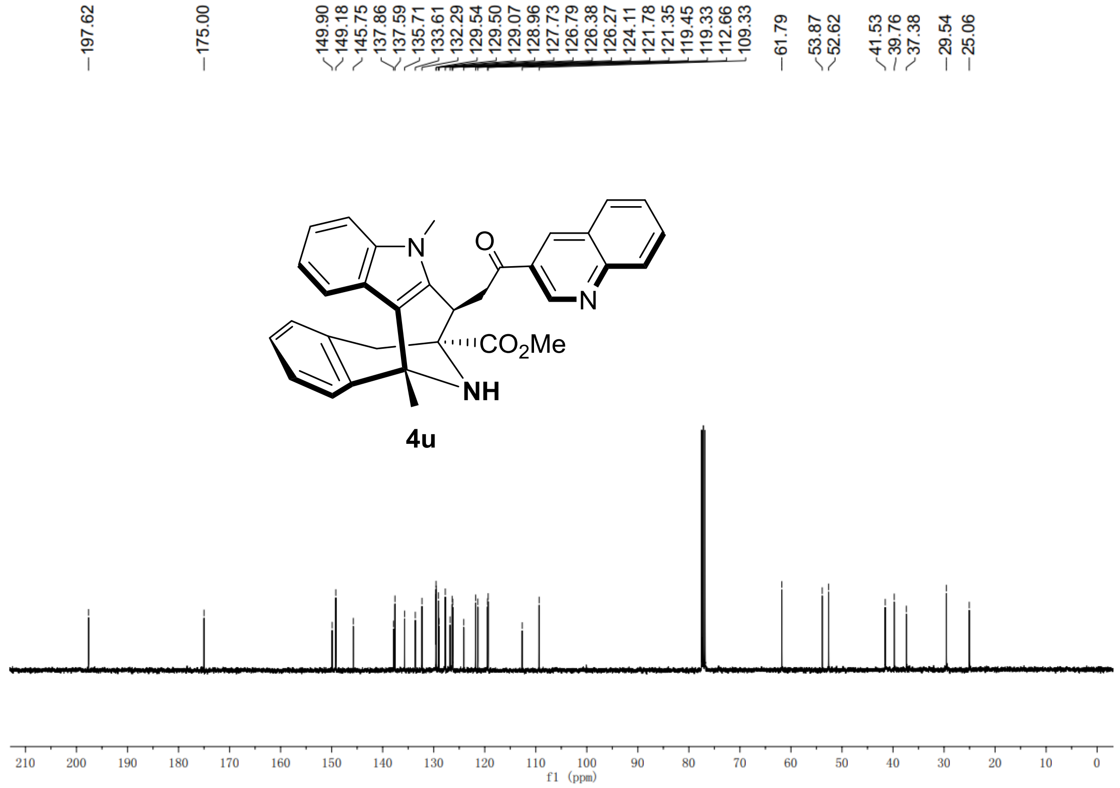


$^{1}$H-NMR and $^{13}$C-NMR of **4u**

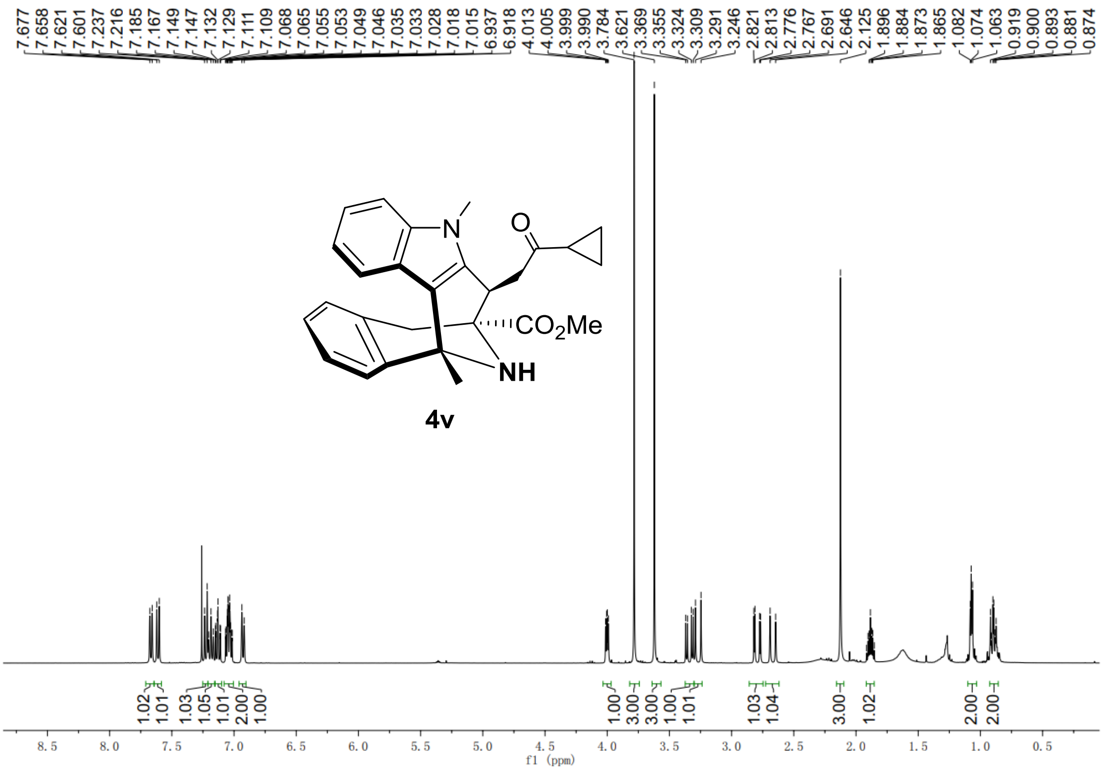


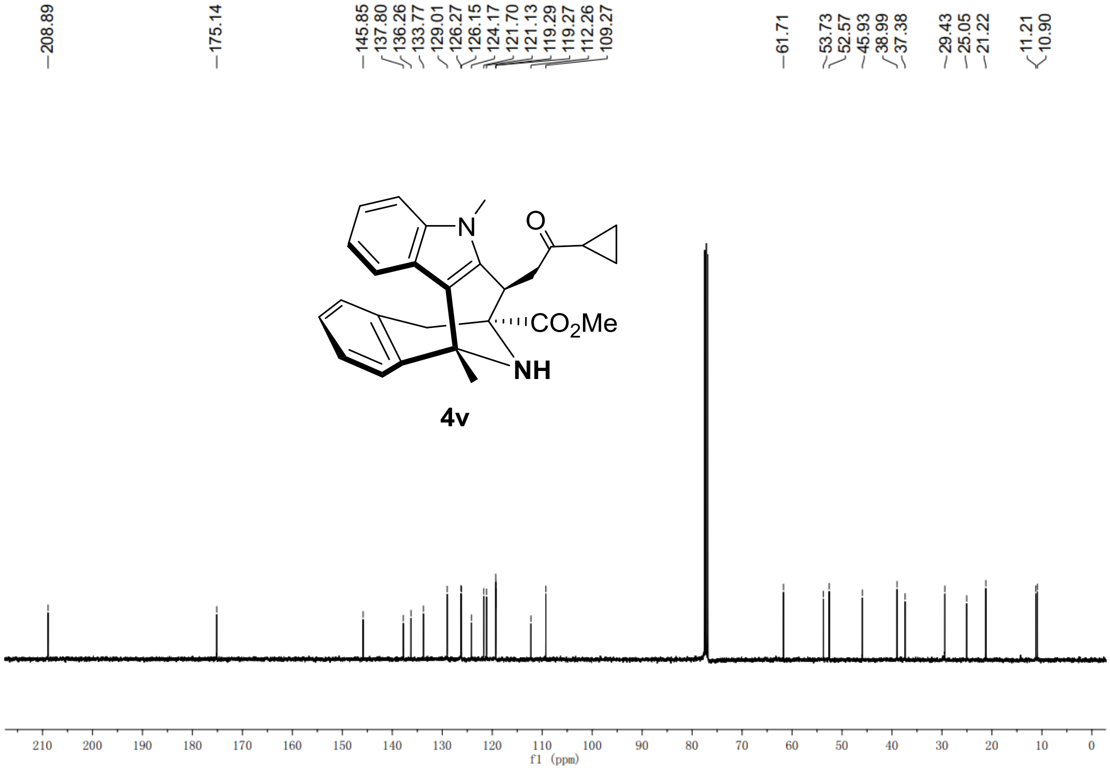


$^{1}$H-NMR and $^{13}$C-NMR of **4v**

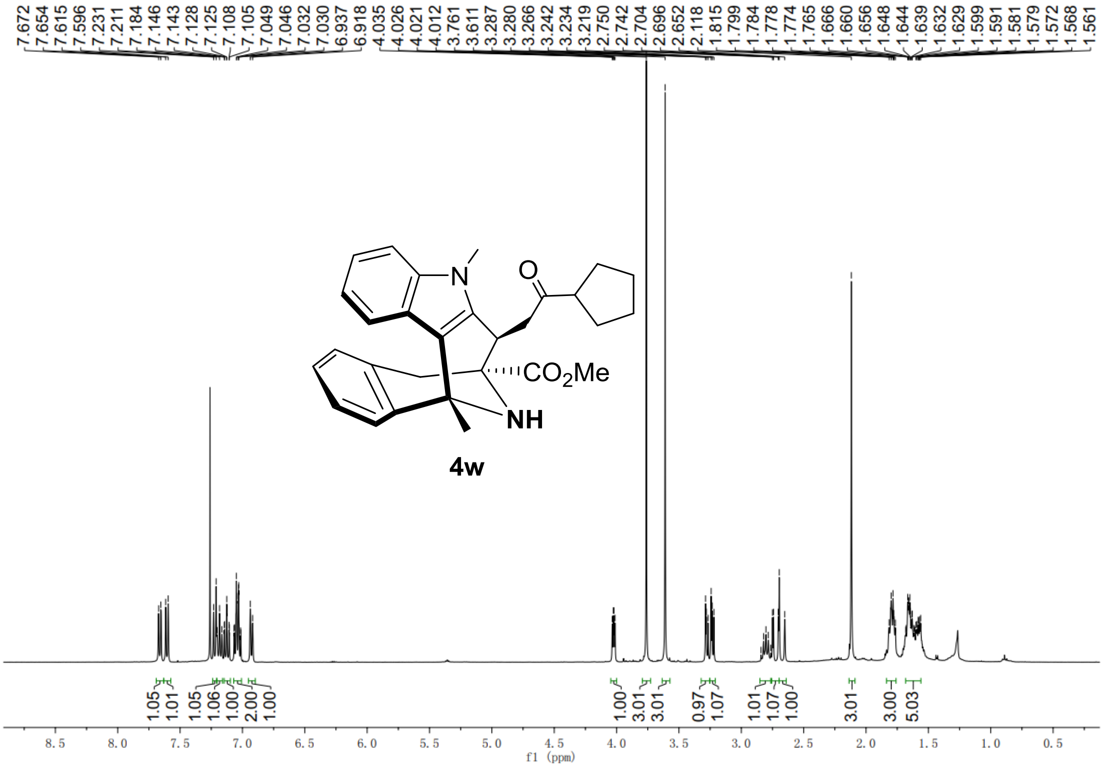


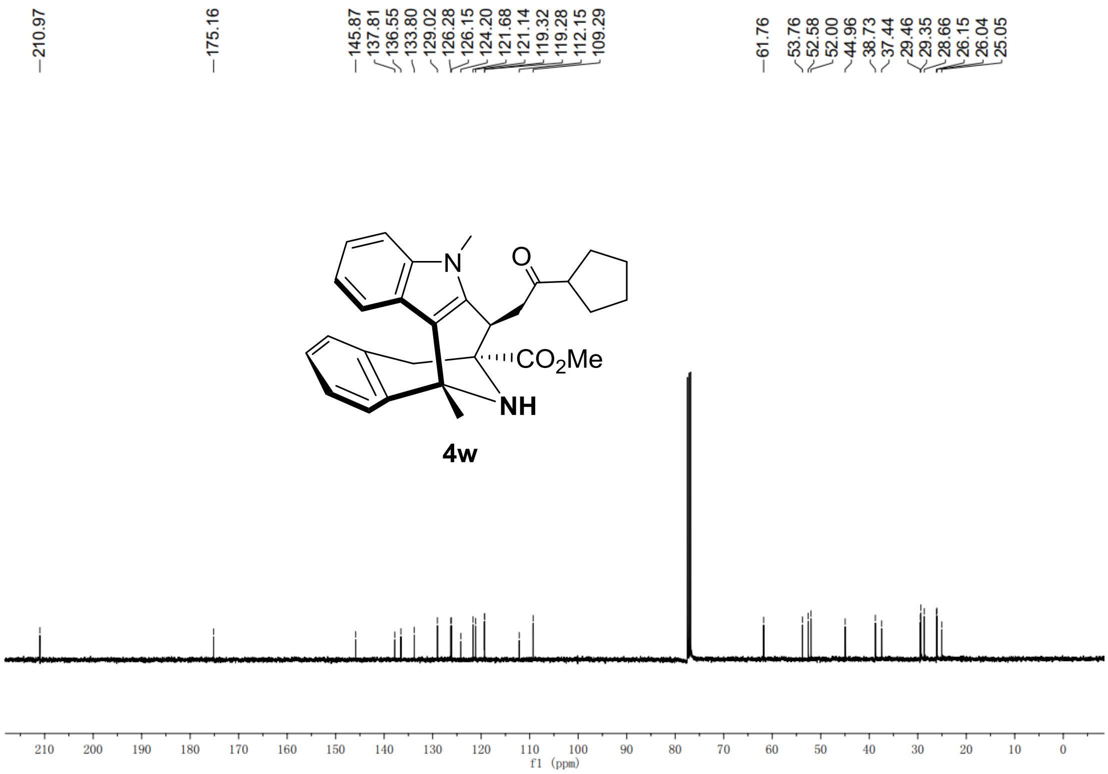


$^1$H-NMR and $^{13}$C-NMR of **4w**

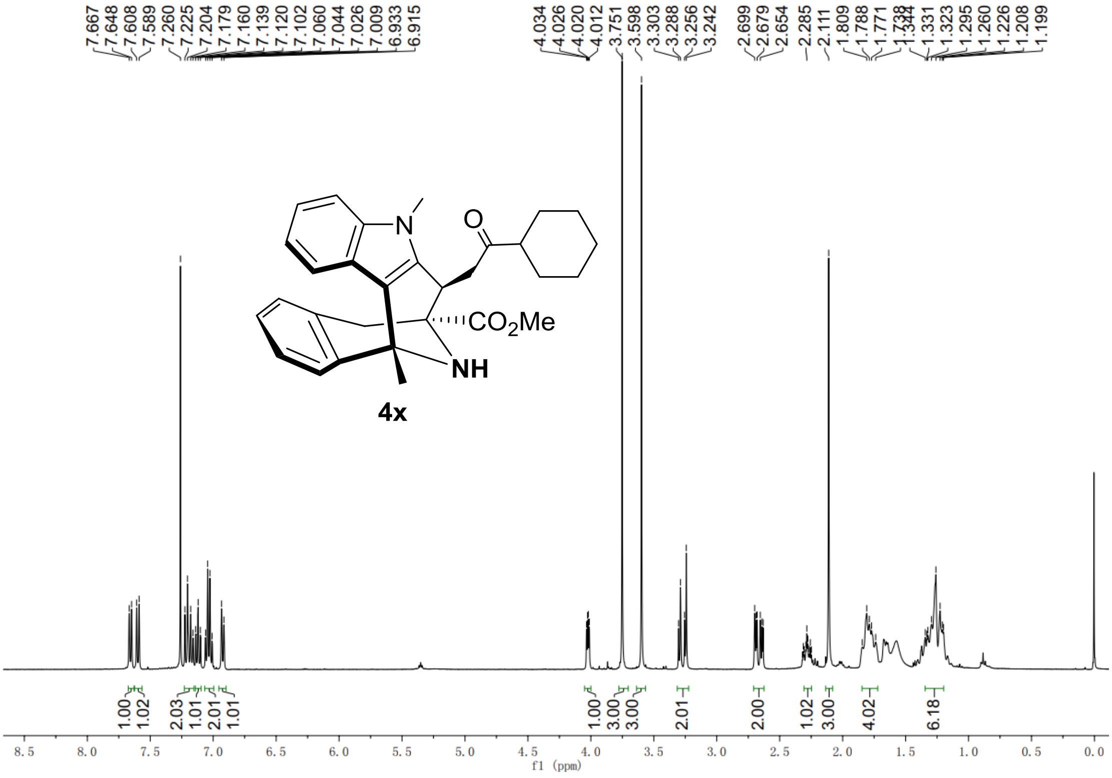

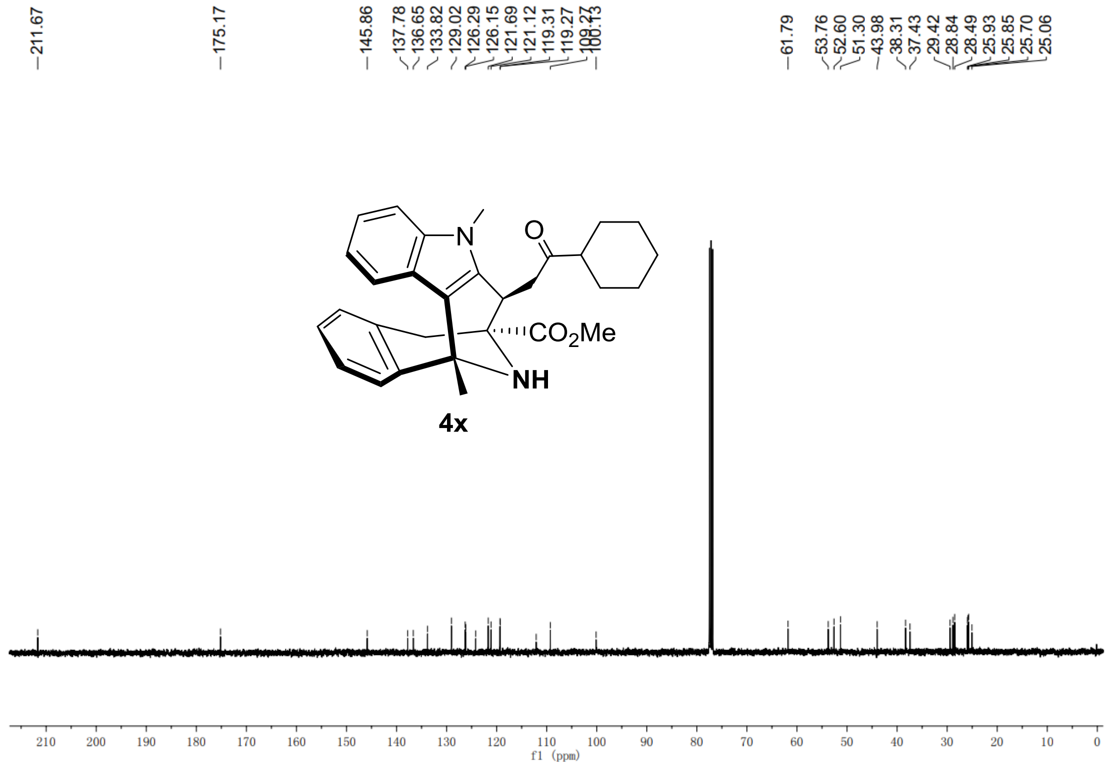

$^{1}$H-NMR and $^{13}$C-NMR of **4x**

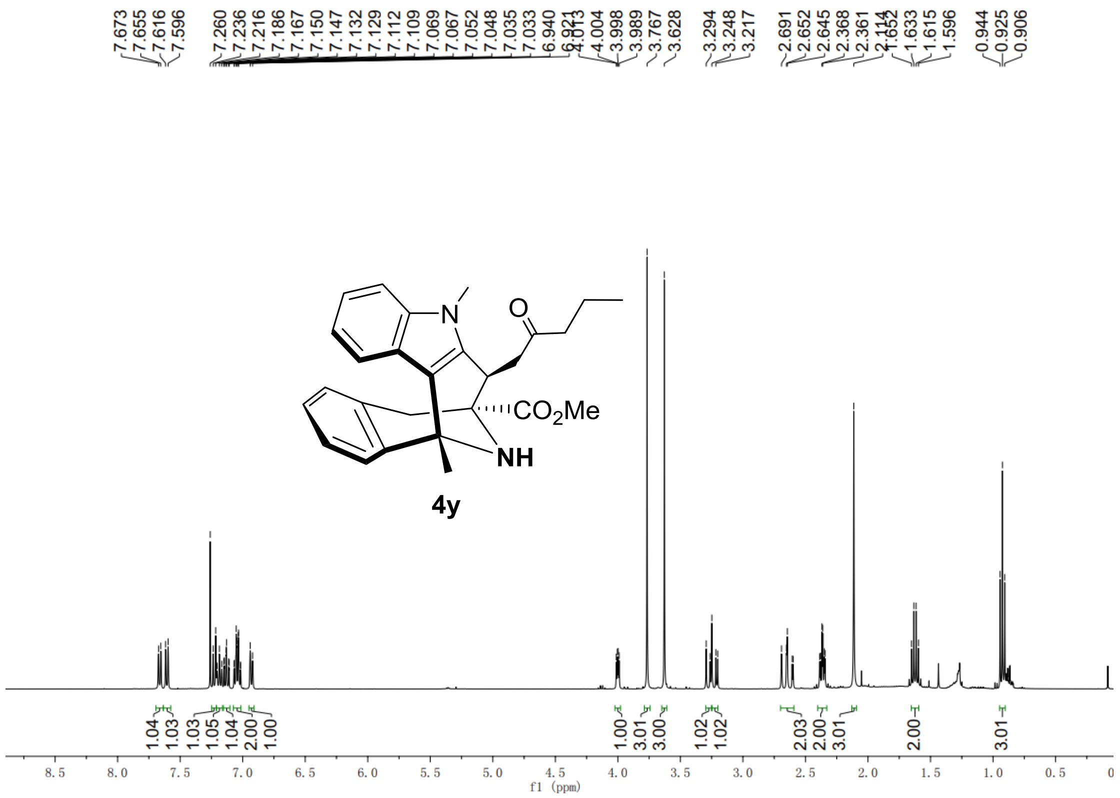

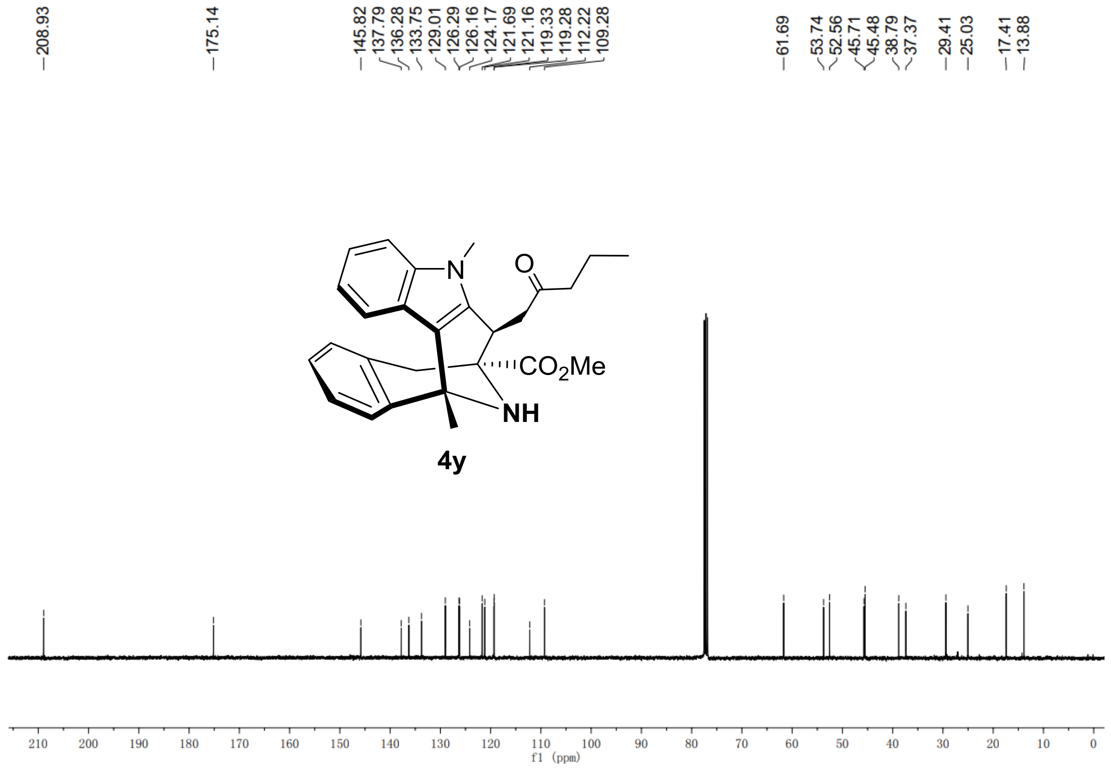

$^{1}$H-NMR and $^{13}$C-NMR of **4y**

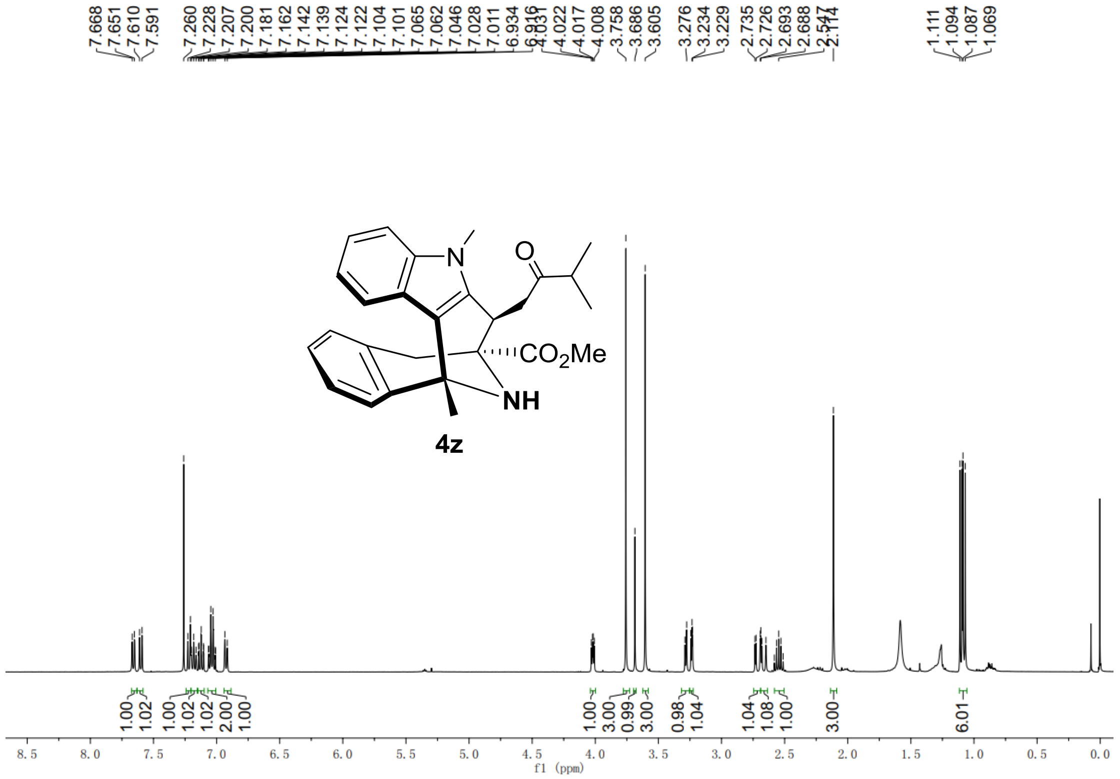

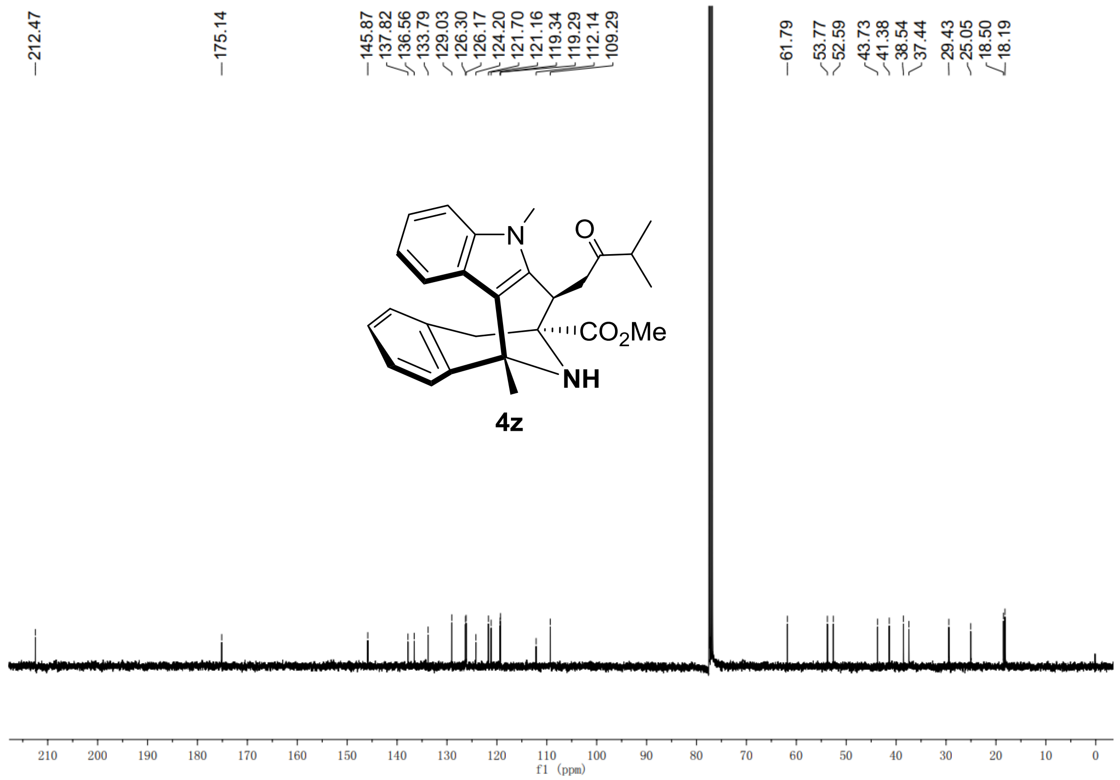

$^1$H-NMR and $^{13}$C-NMR of **4z**

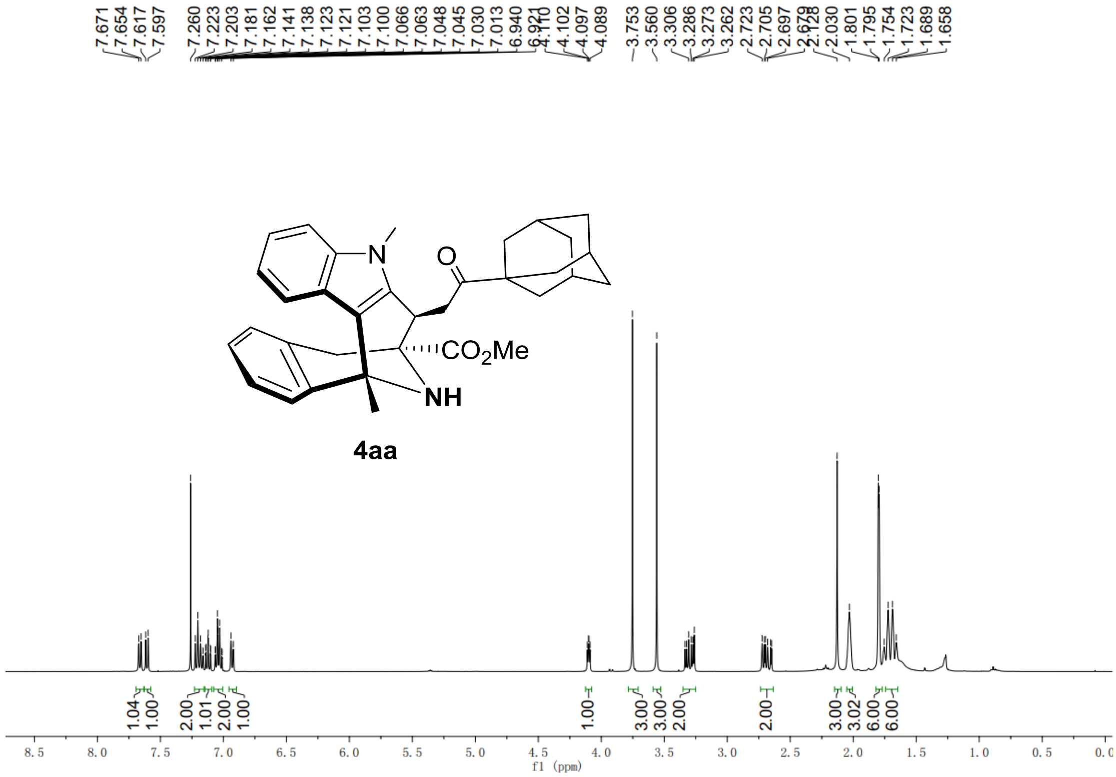


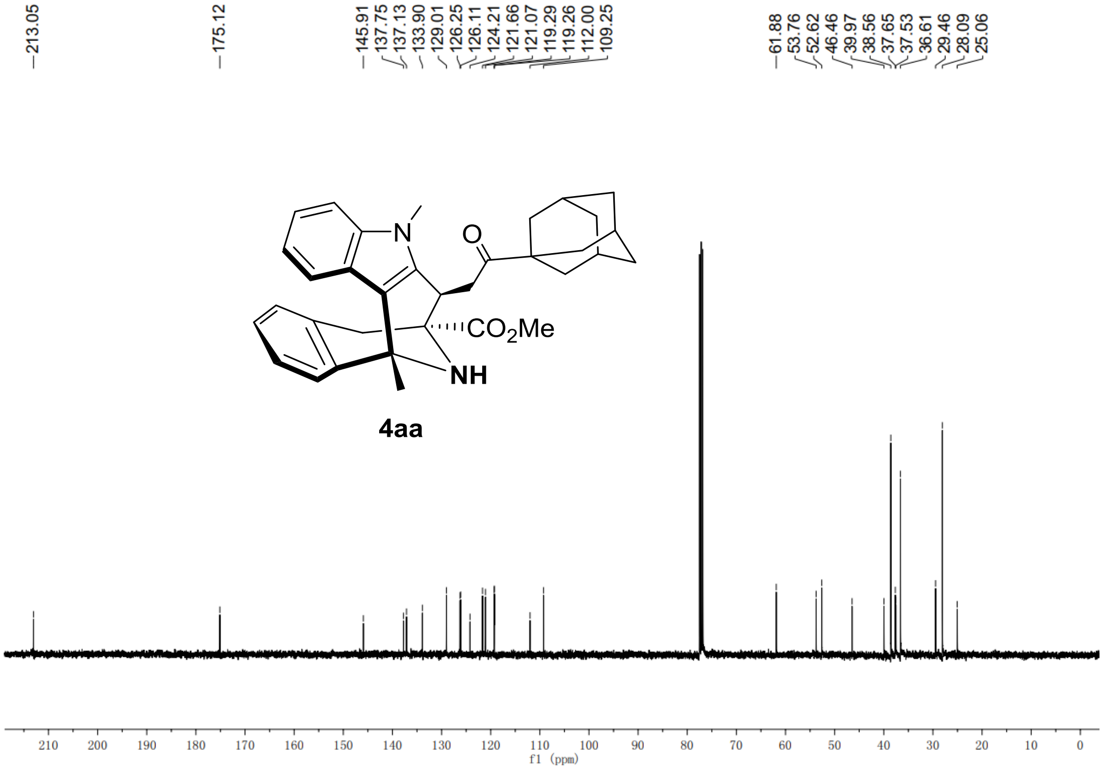


$^{1}$H-NMR and $^{13}$C-NMR of **4aa**

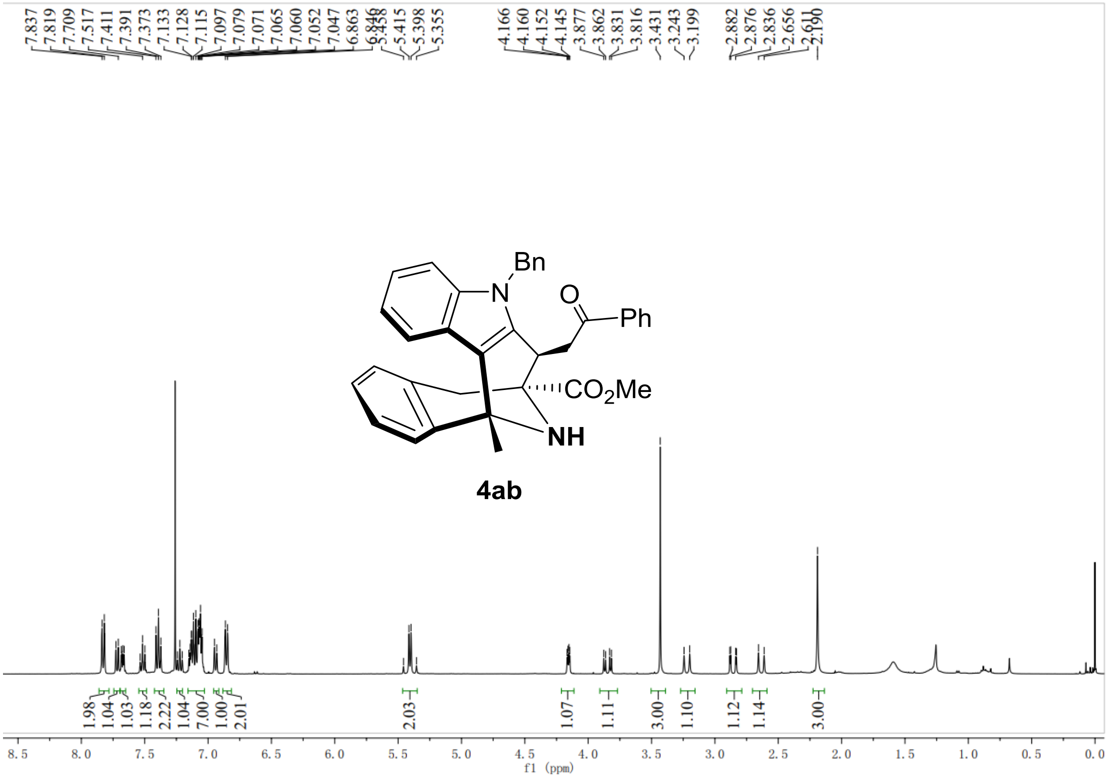


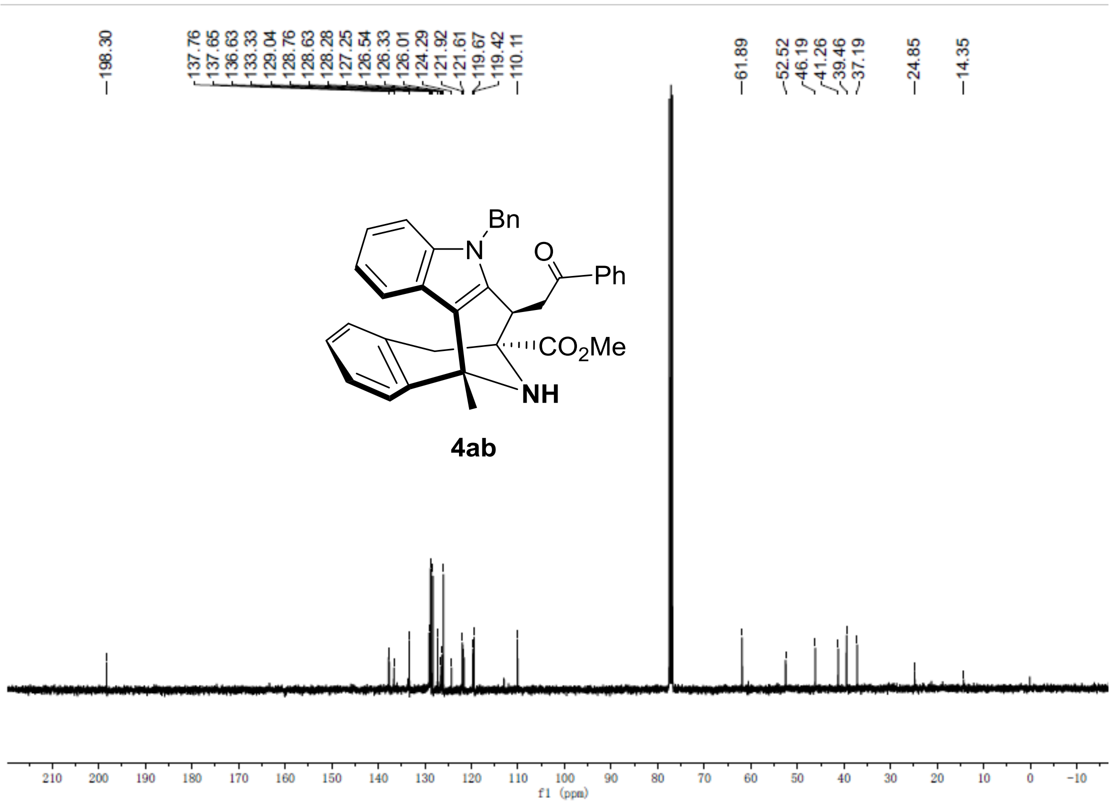


$^1$H-NMR and $^{13}$C-NMR of **4ab**

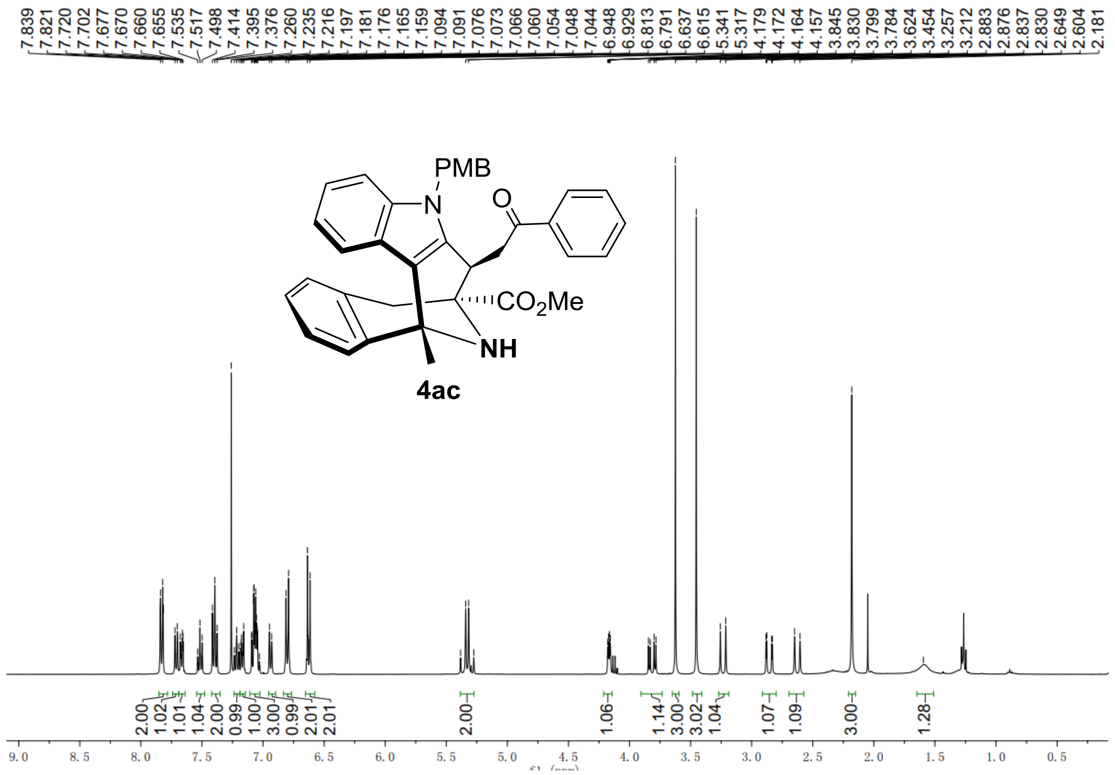

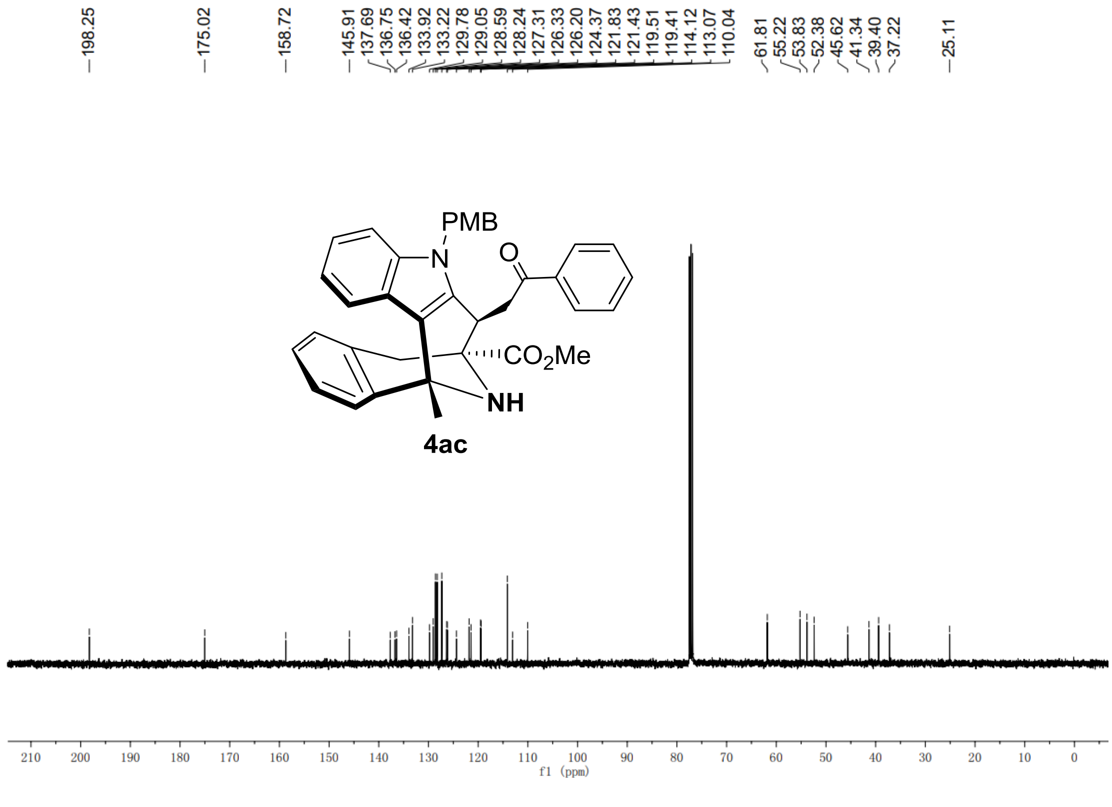

$^{1}$H-NMR and $^{13}$C-NMR of **4ac**

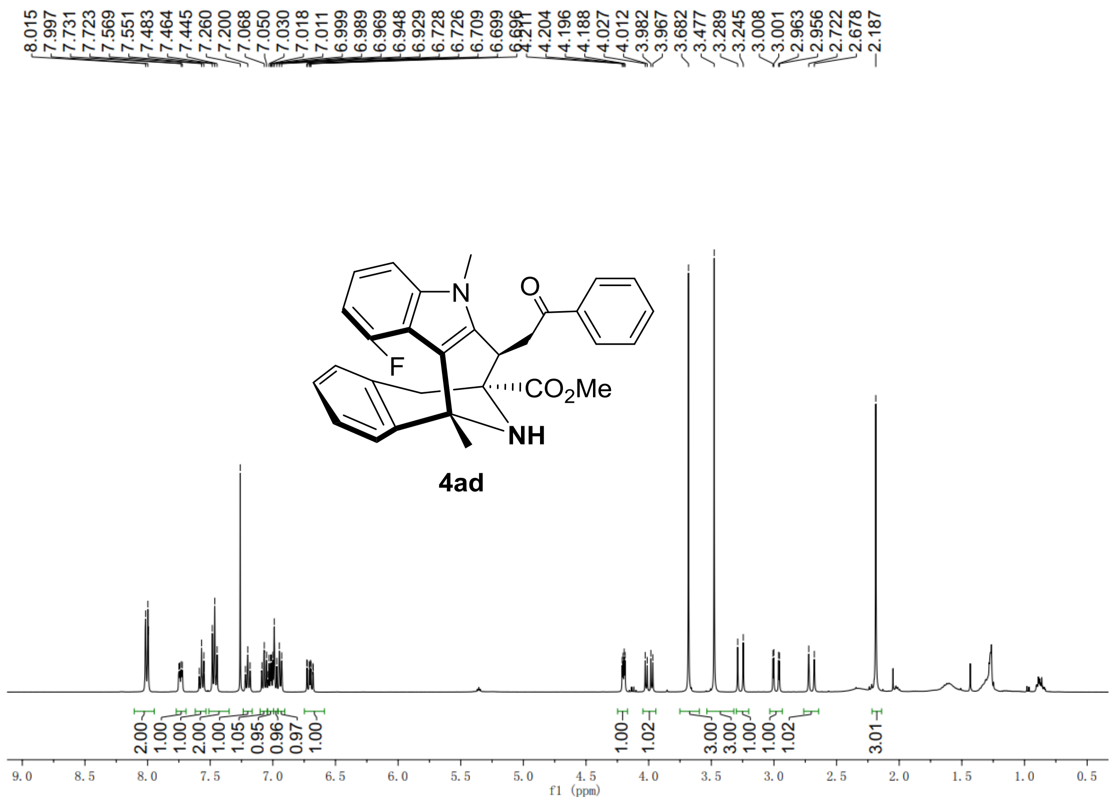

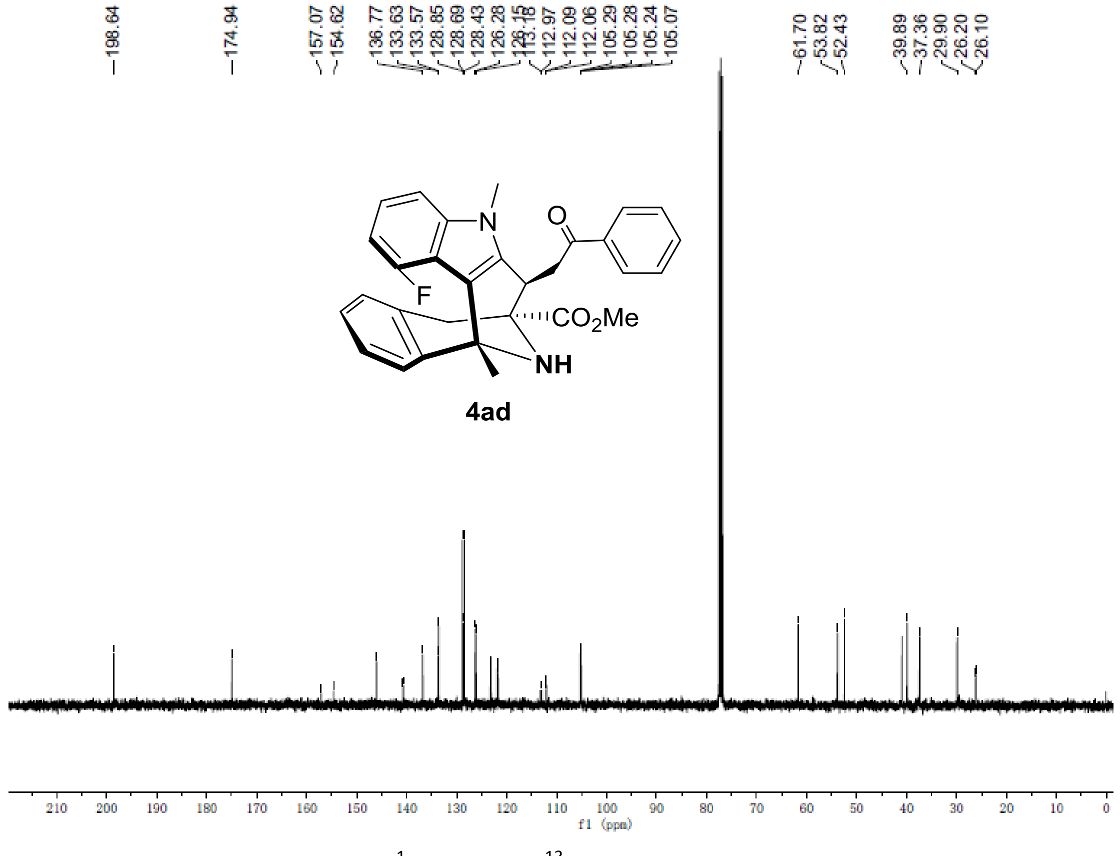

$^{1}$H-NMR and $^{13}$C-NMR of **4ad**

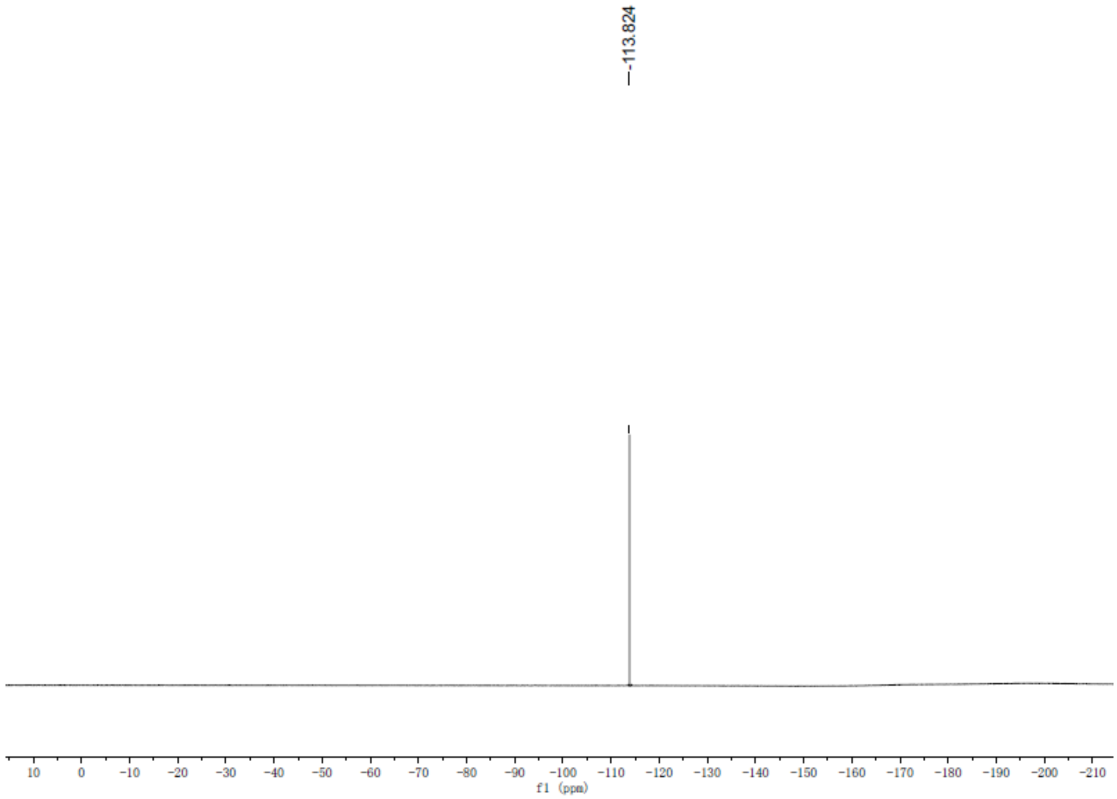


$^{19}$F-NMR of **4ad**

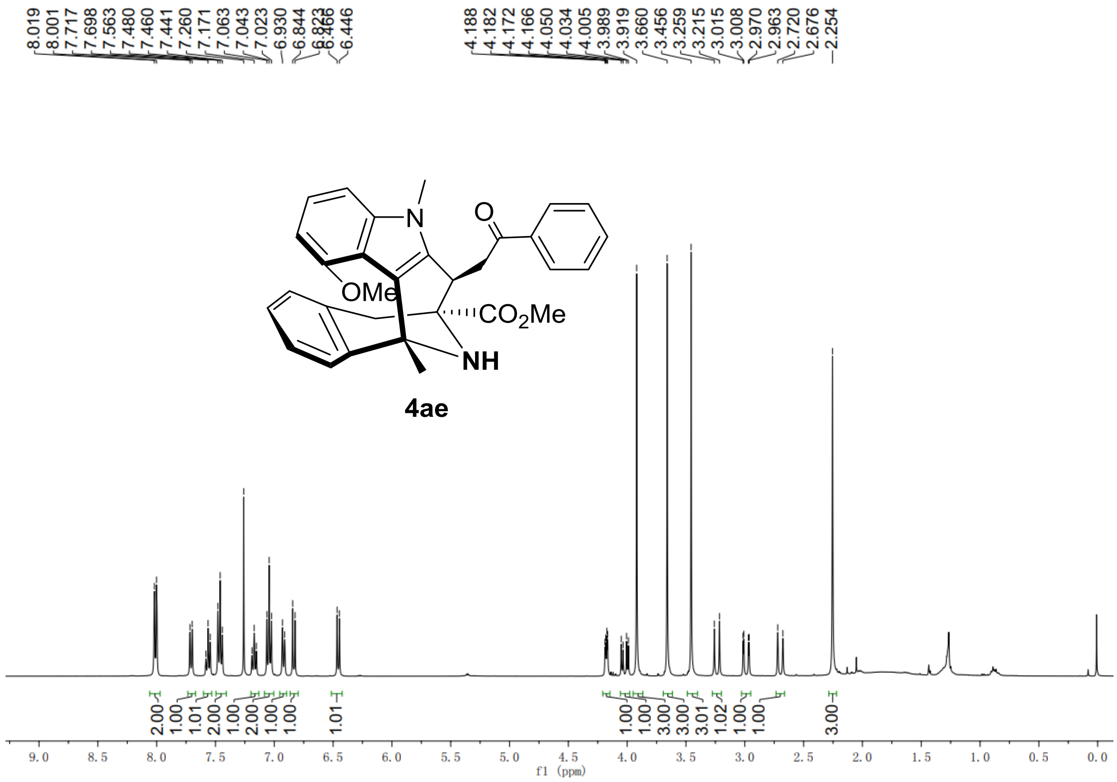

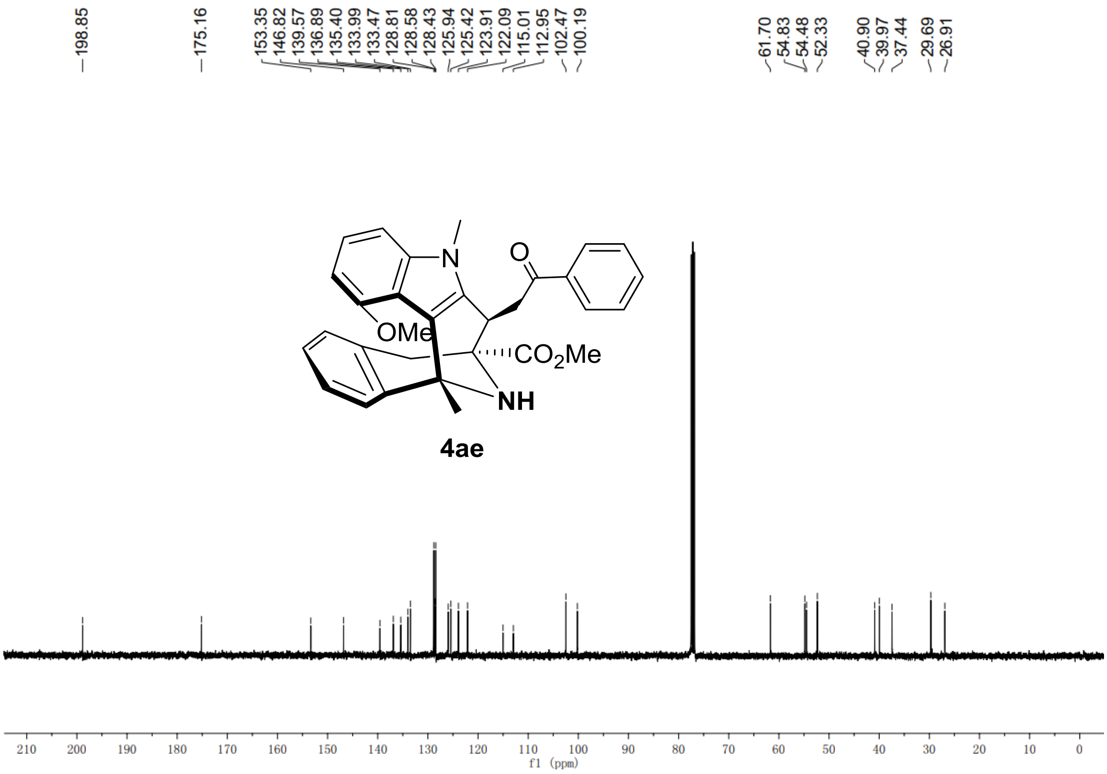

4ae

$^{1}$H-NMR and $^{13}$C-NMR of **4ae**

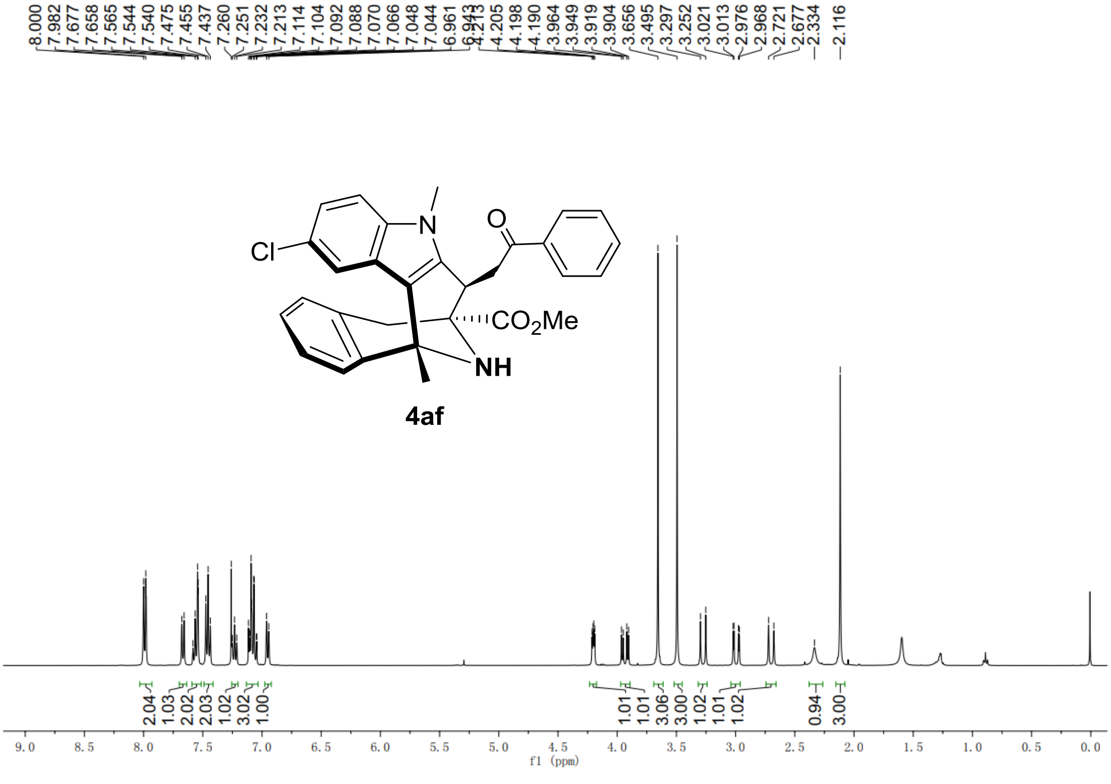

4af

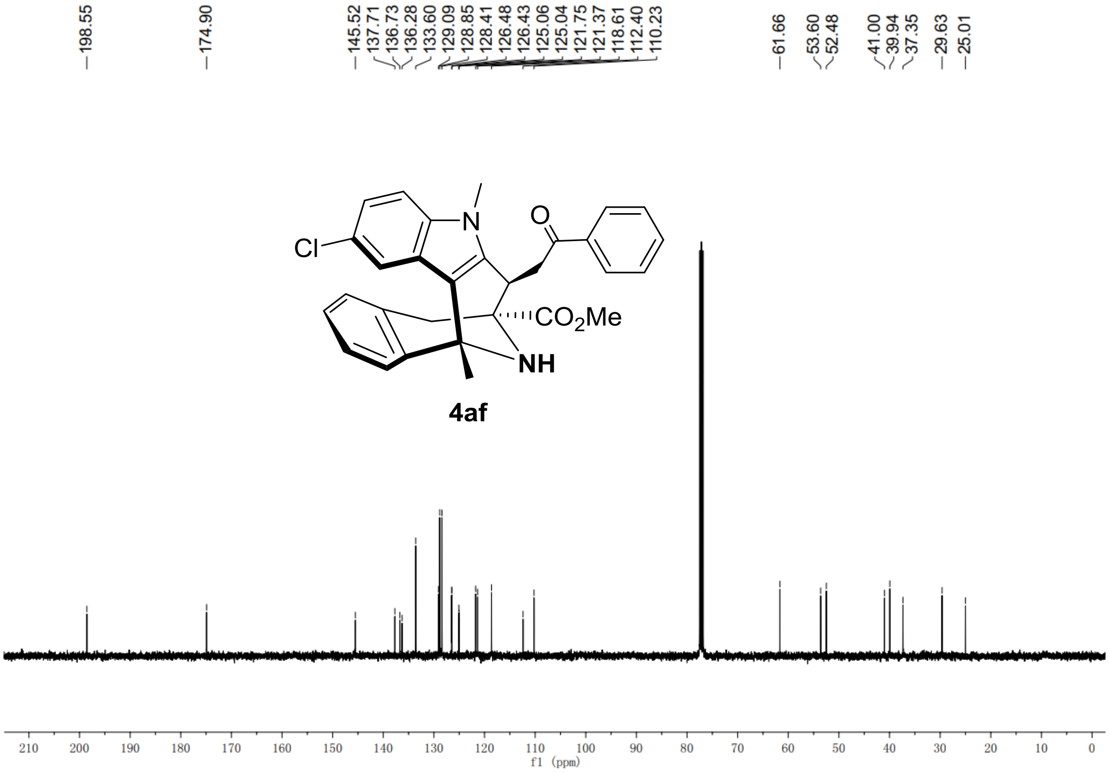


$^1$H-NMR and $^{13}$C-NMR of **4af**

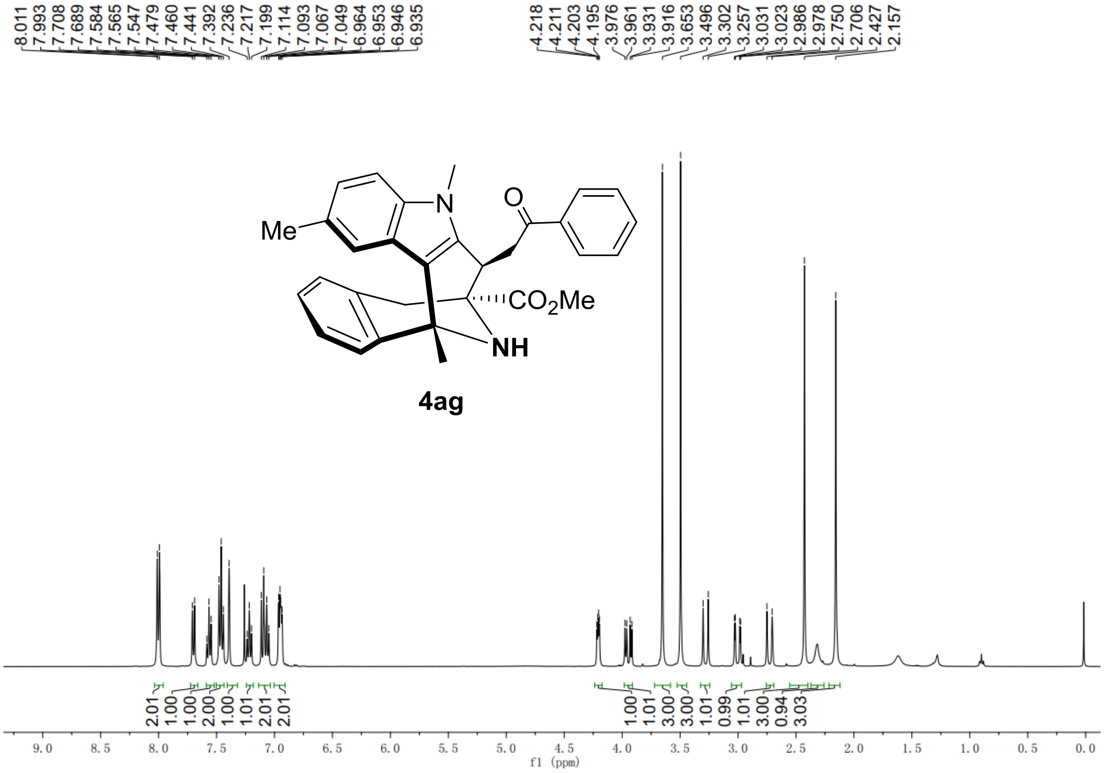

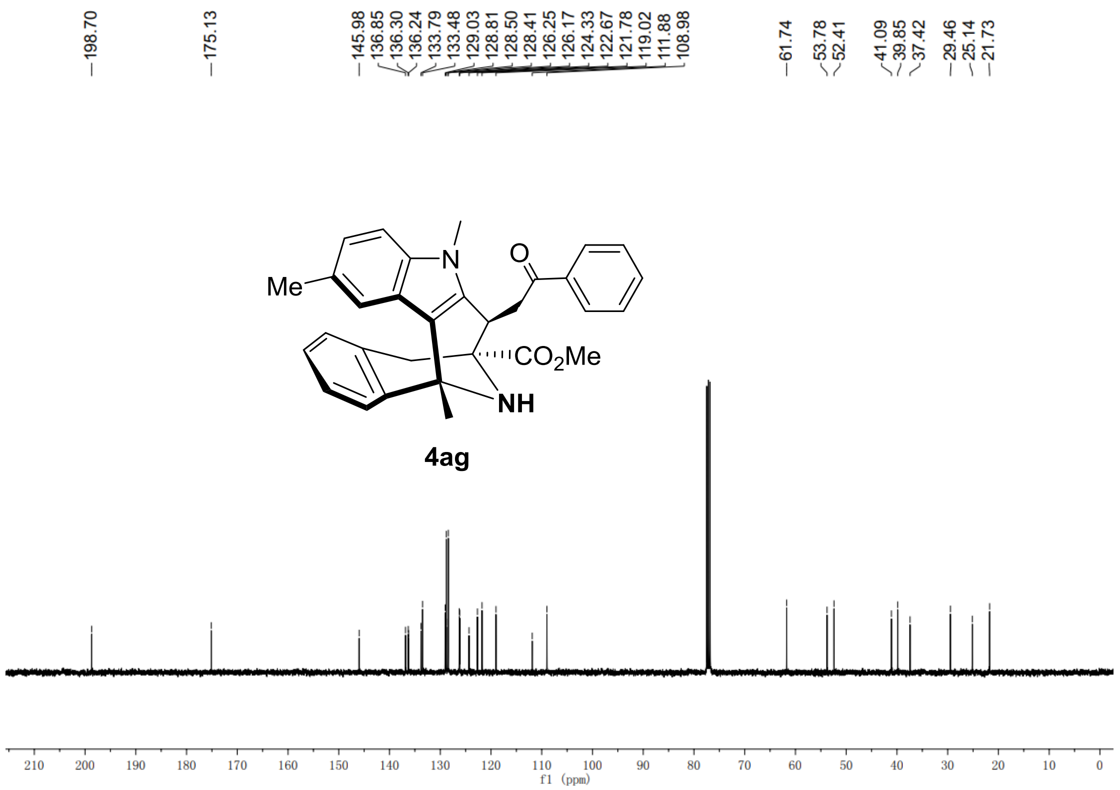


$^{1}$H-NMR and $^{13}$C-NMR of **4ag**

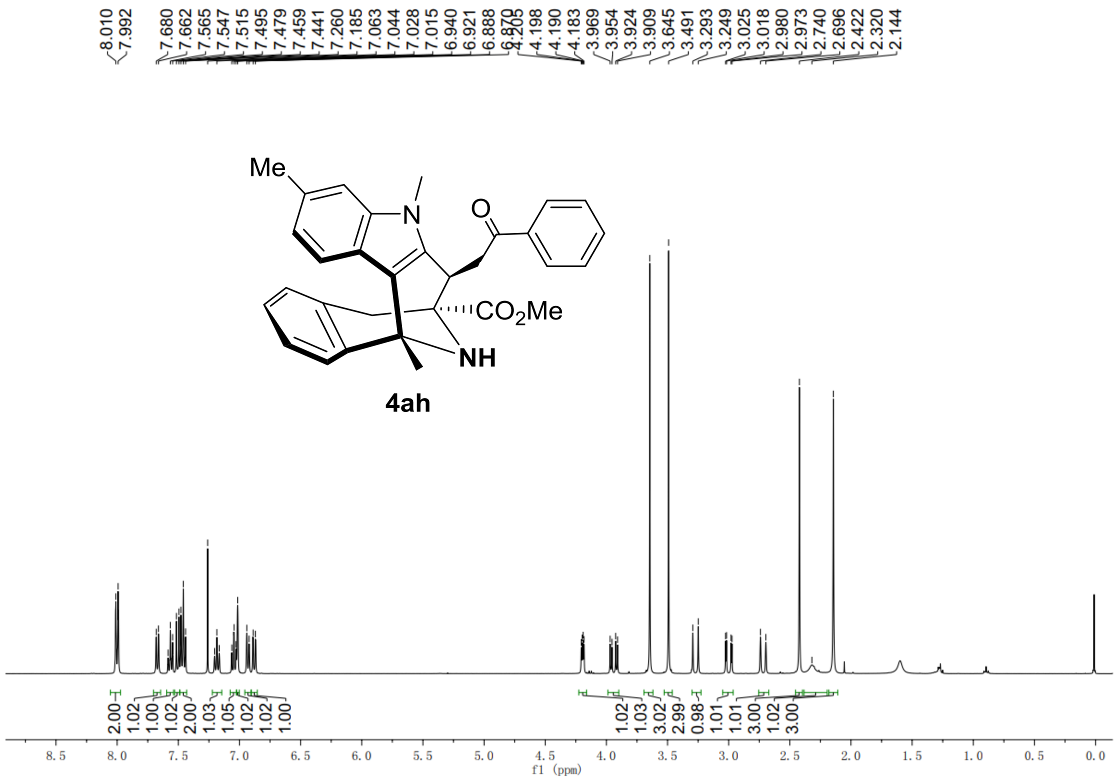

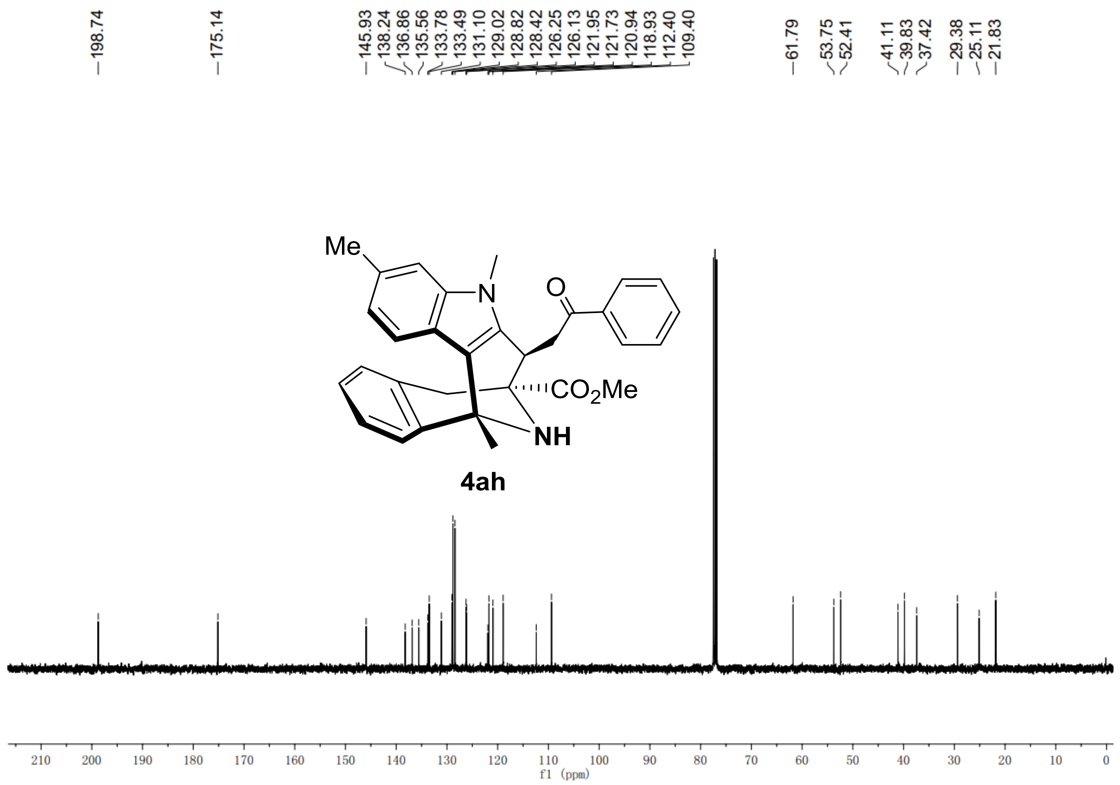


$^1$H-NMR and $^{13}$C-NMR of **4ah**

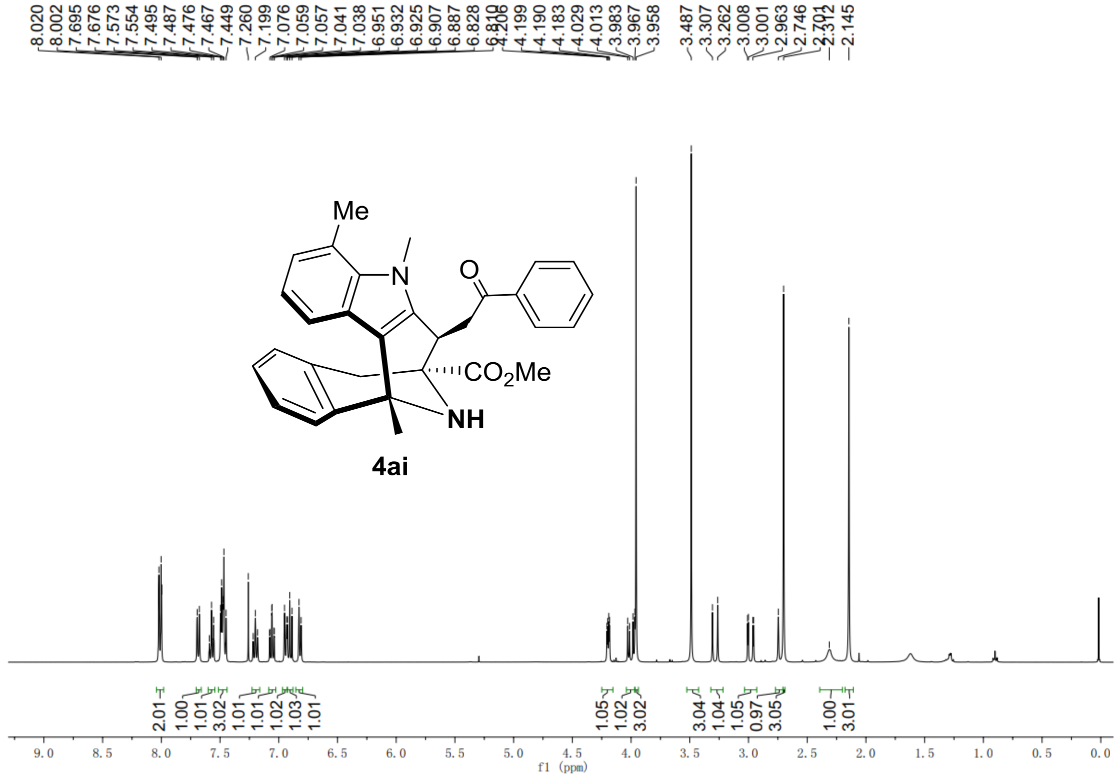

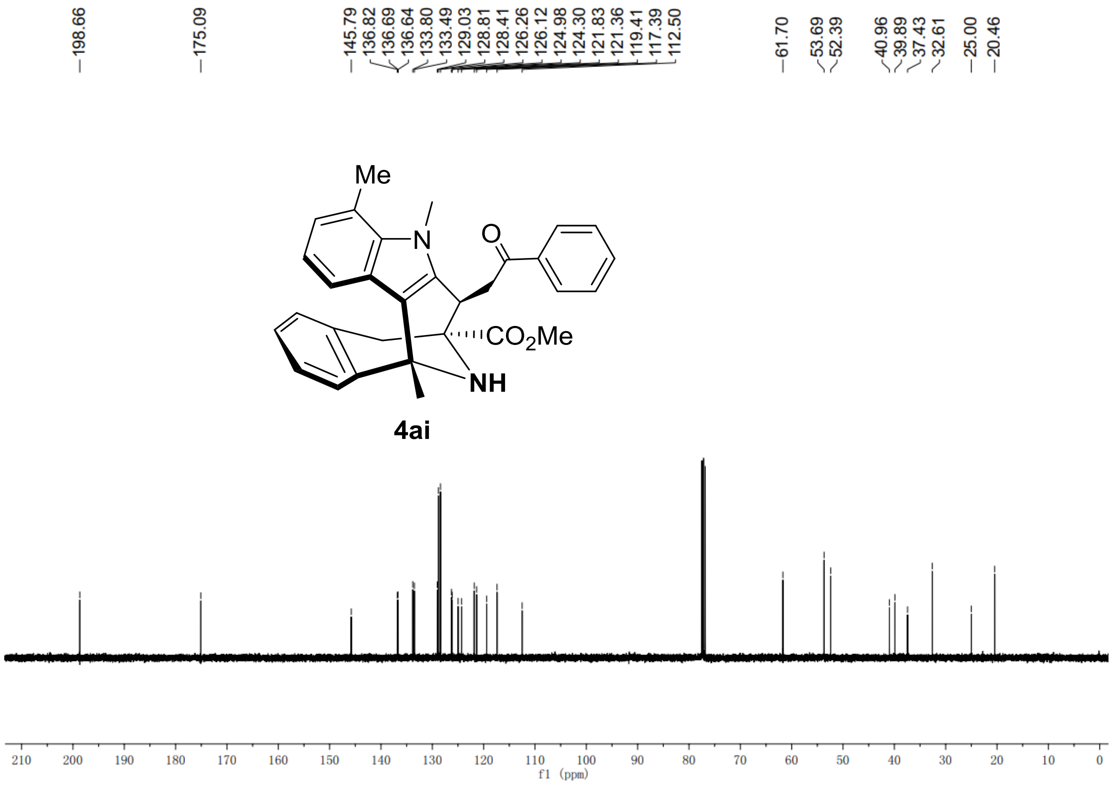


$^{1}$H-NMR and $^{13}$C-NMR of **4ai**

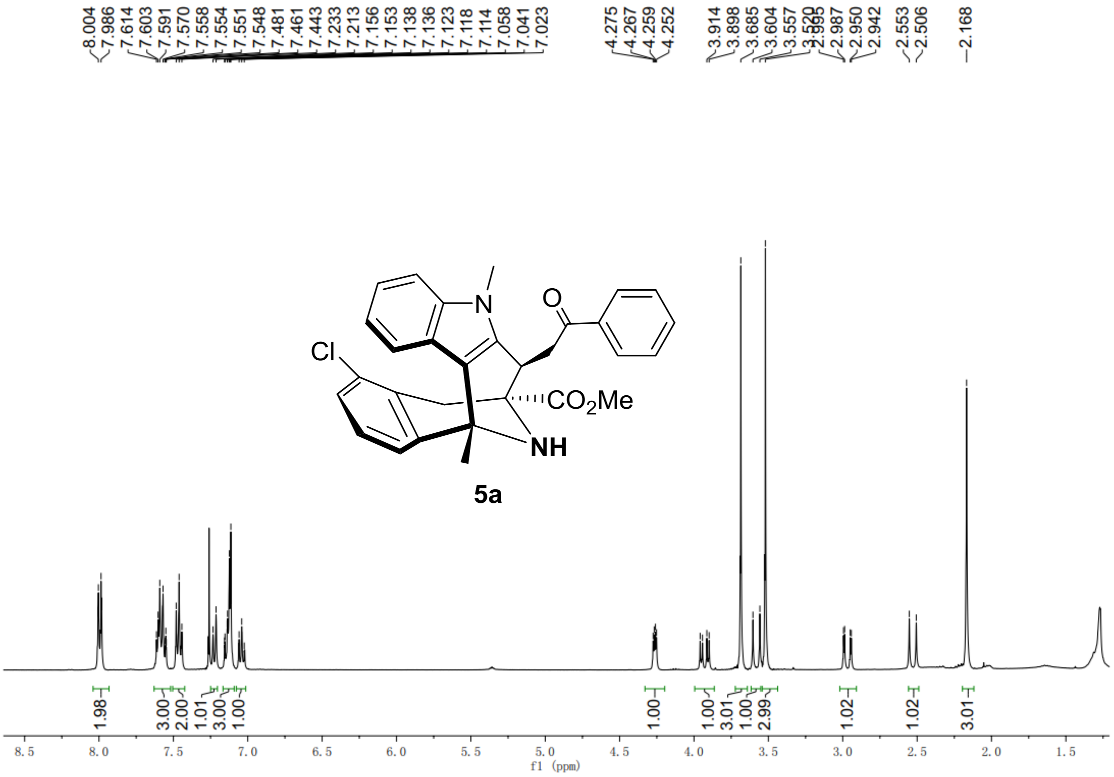

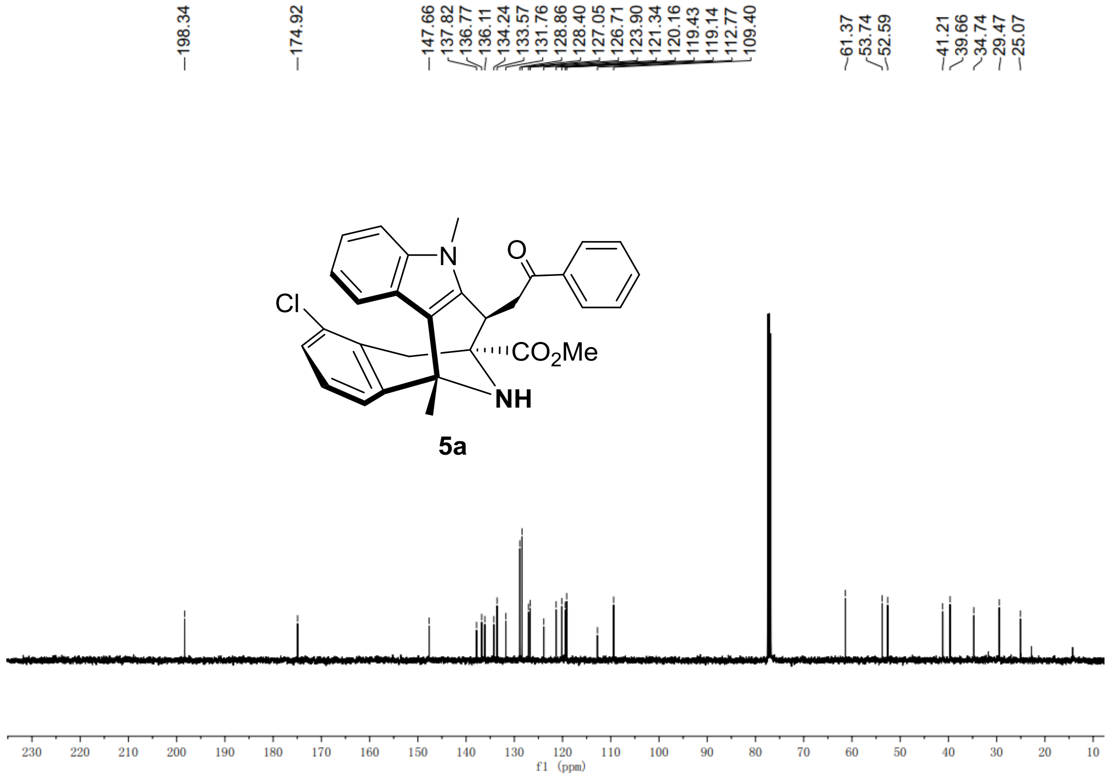


$^{1}$H-NMR and $^{13}$C-NMR of **5a**

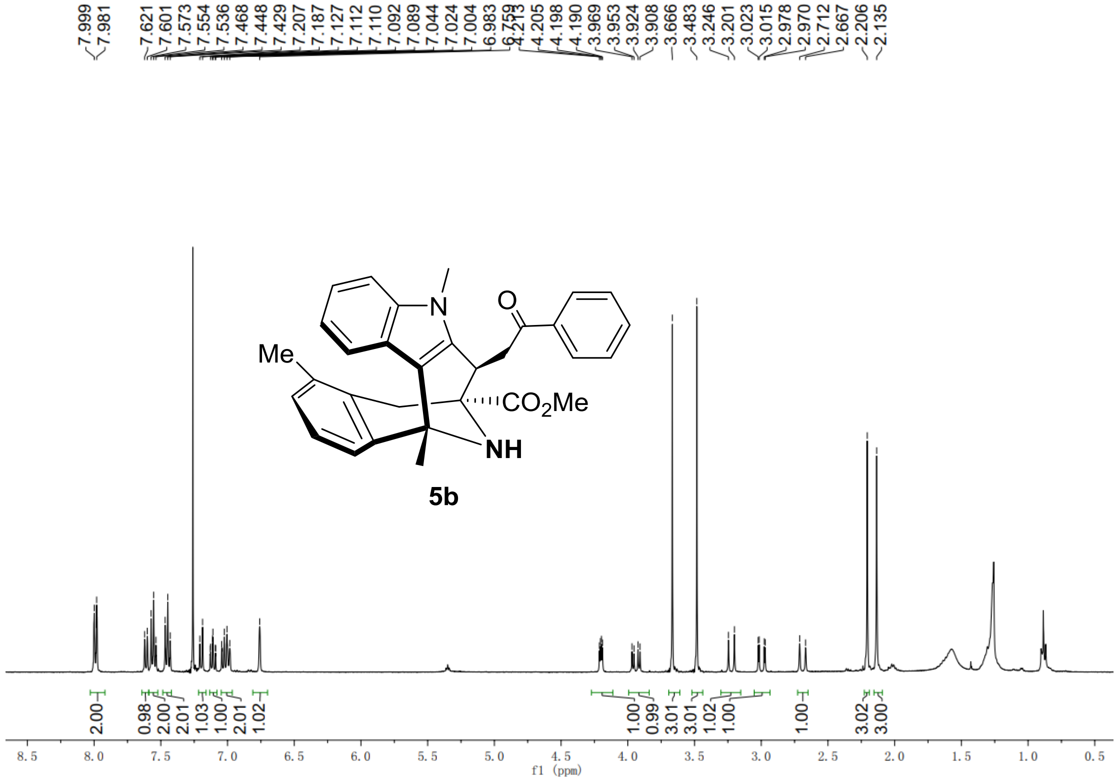

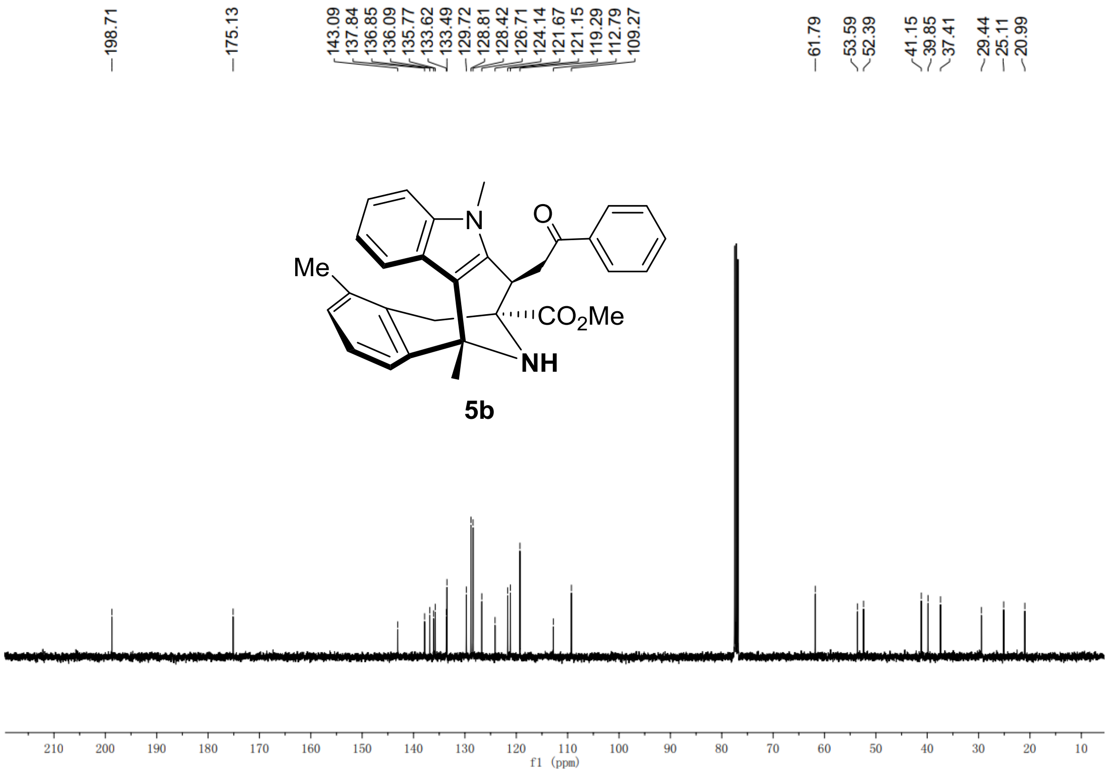


$^{1}$H-NMR and $^{13}$C-NMR of **5b**

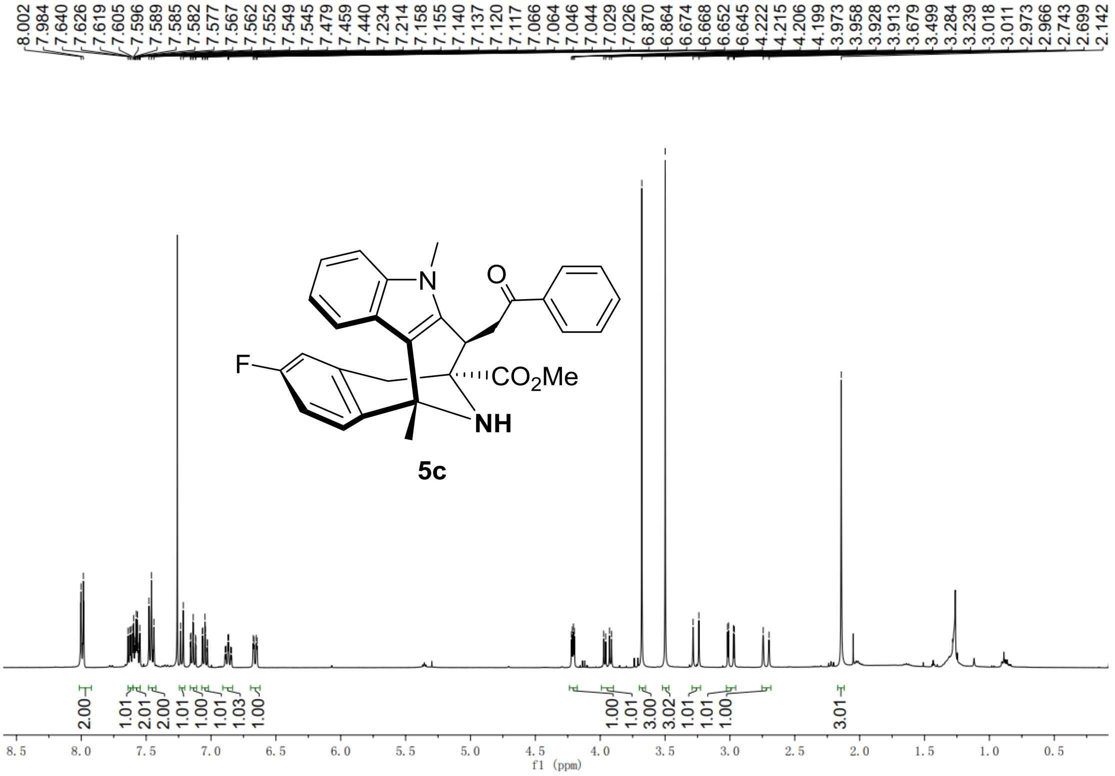

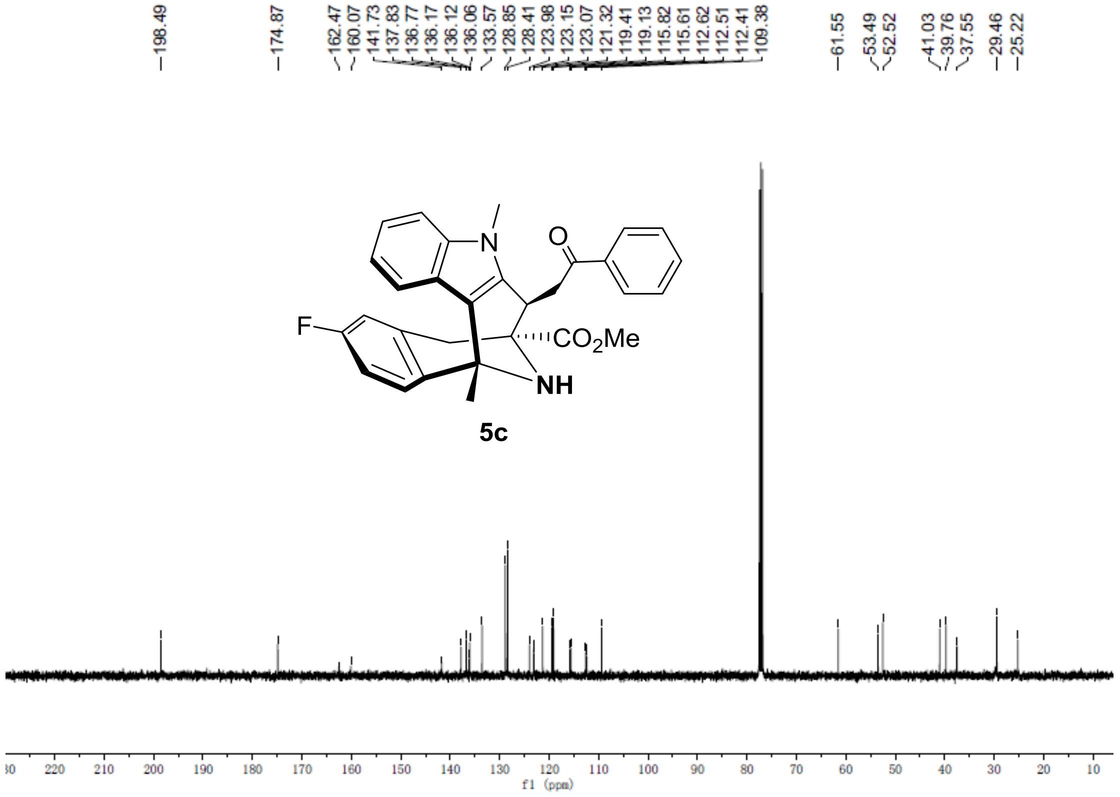


$^{1}$H-NMR and $^{13}$C-NMR of **5c**

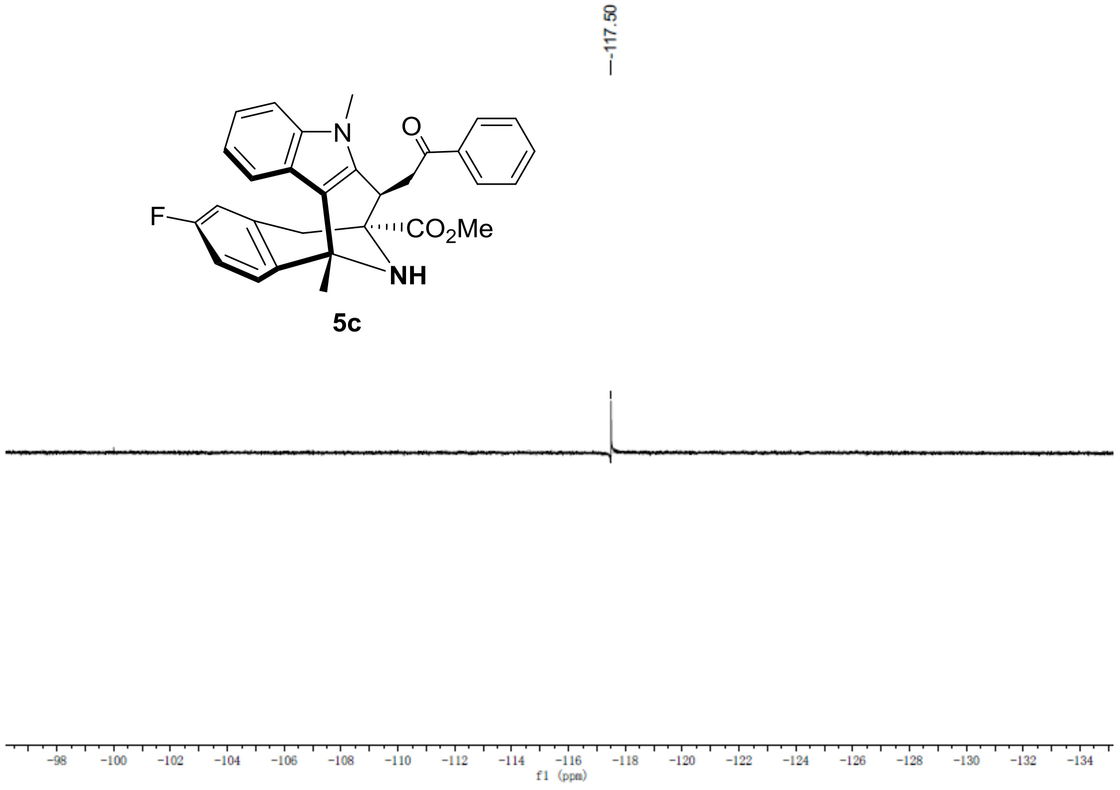


$^{19}$F-NMR of **5c**

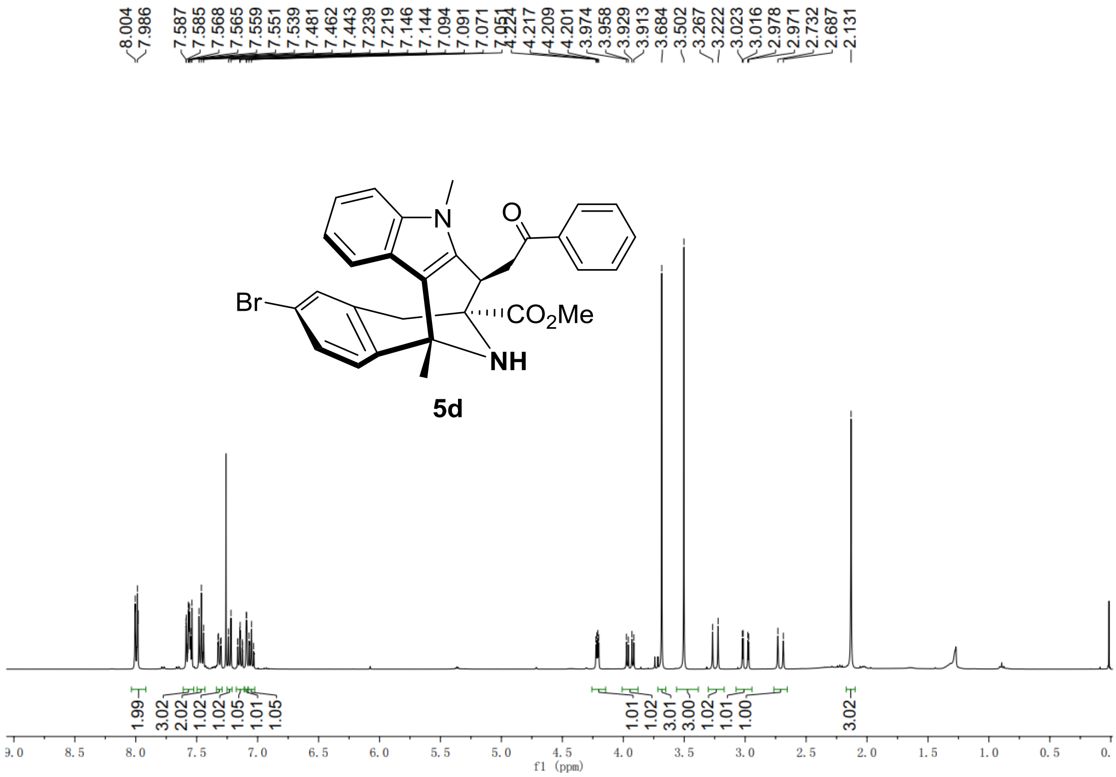


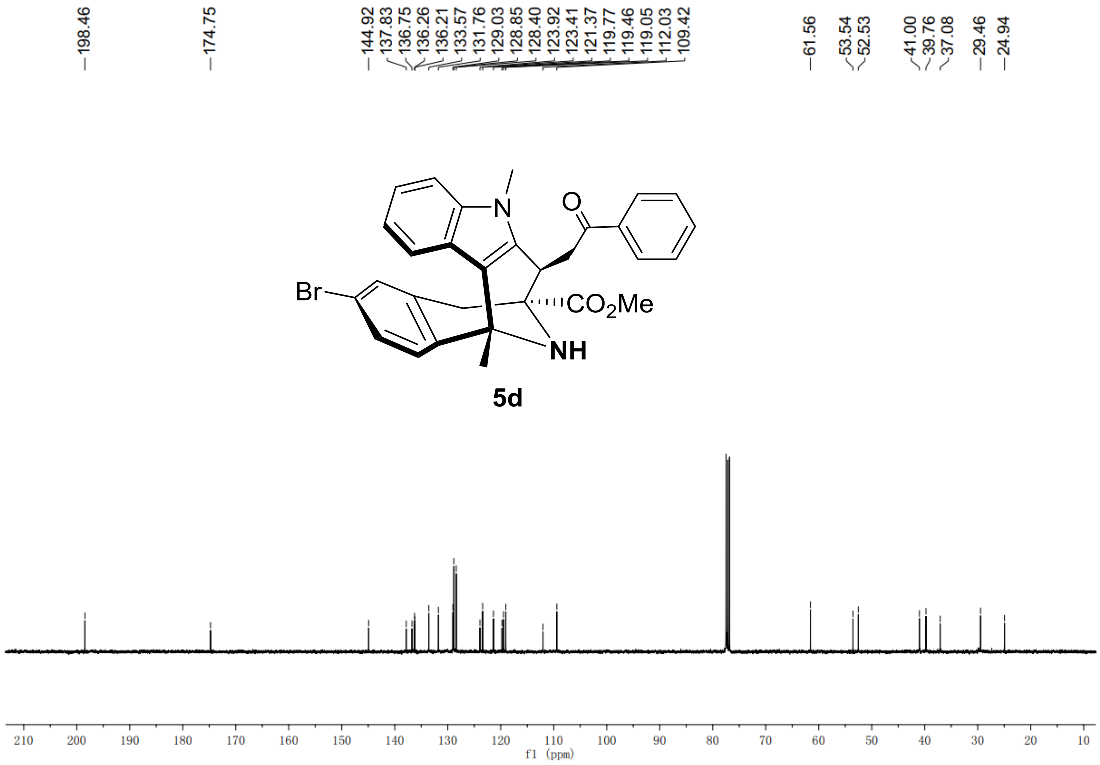


$^{1}$H-NMR and $^{13}$C-NMR of **5d**

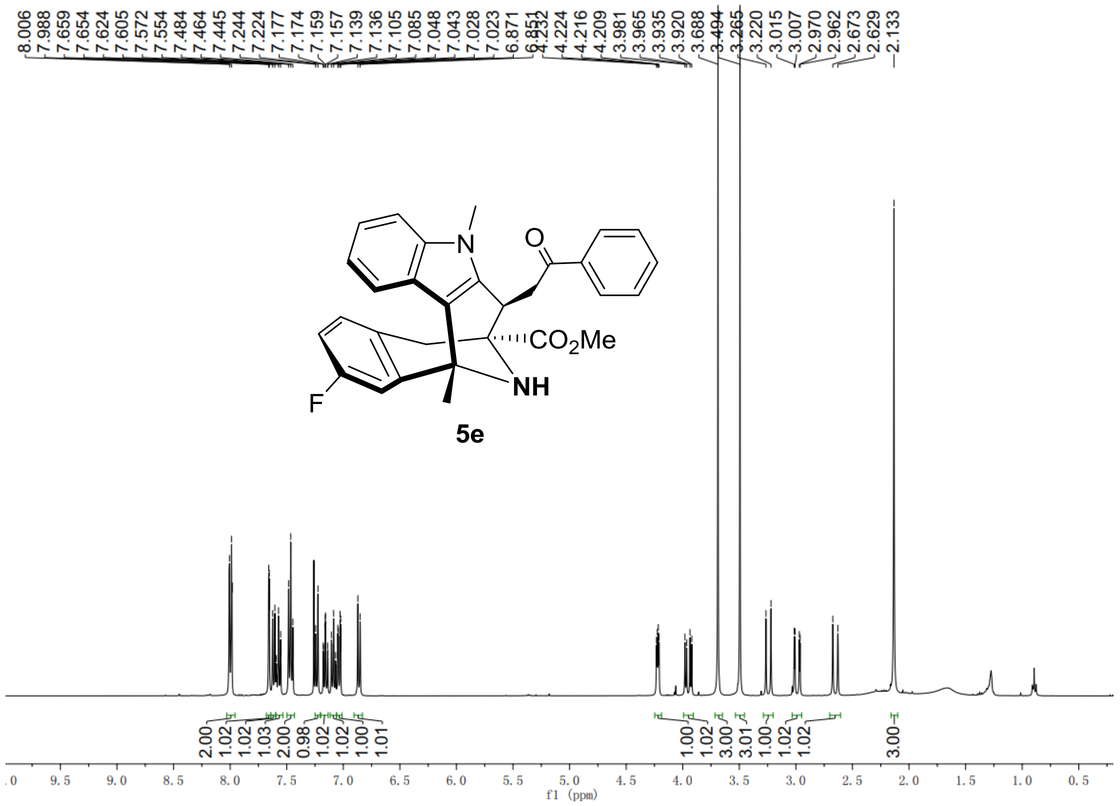


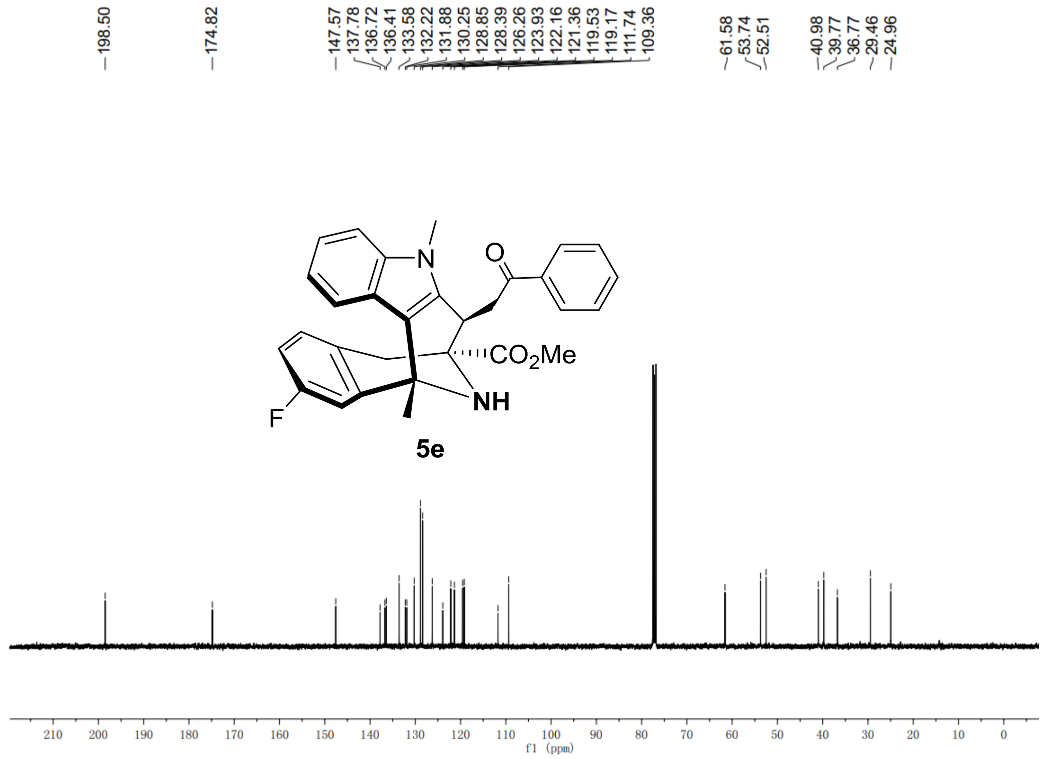


$^1$H-NMR and $^{13}$C-NMR of **5e**

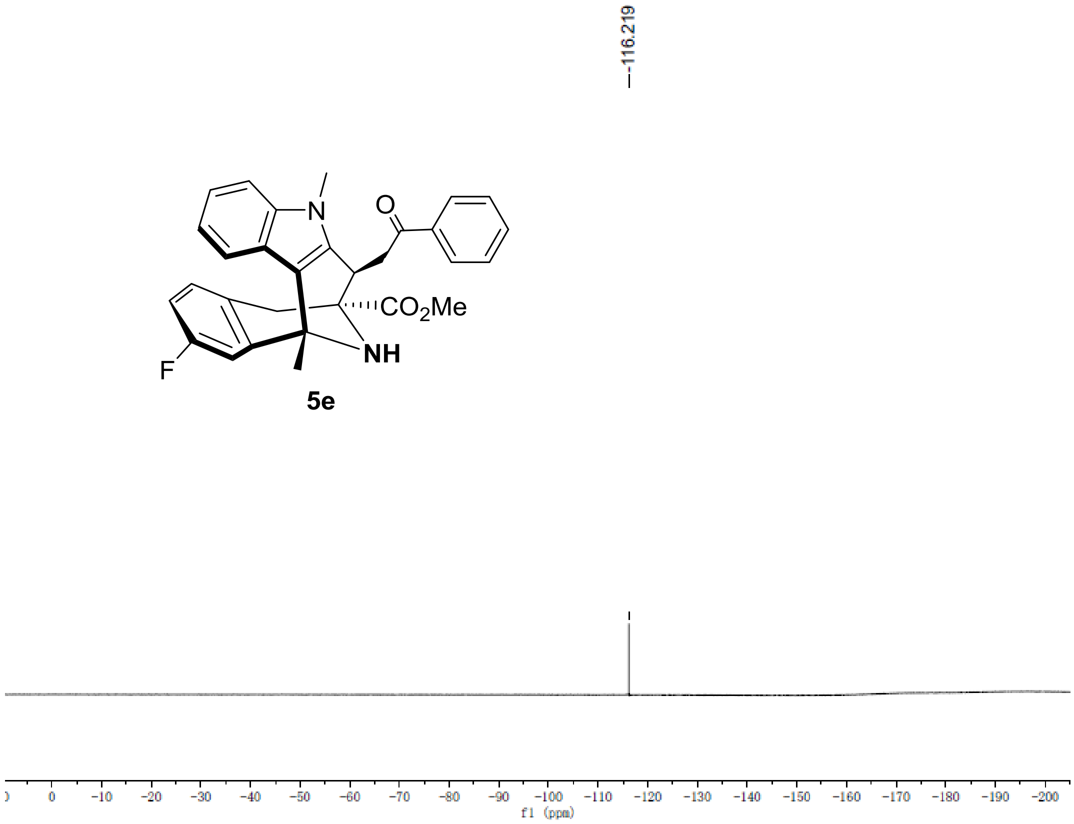

-116.219
F
N
O
CO2Me
NH
5e
f1 (ppm)


$^{19}$F-NMR of **5e**

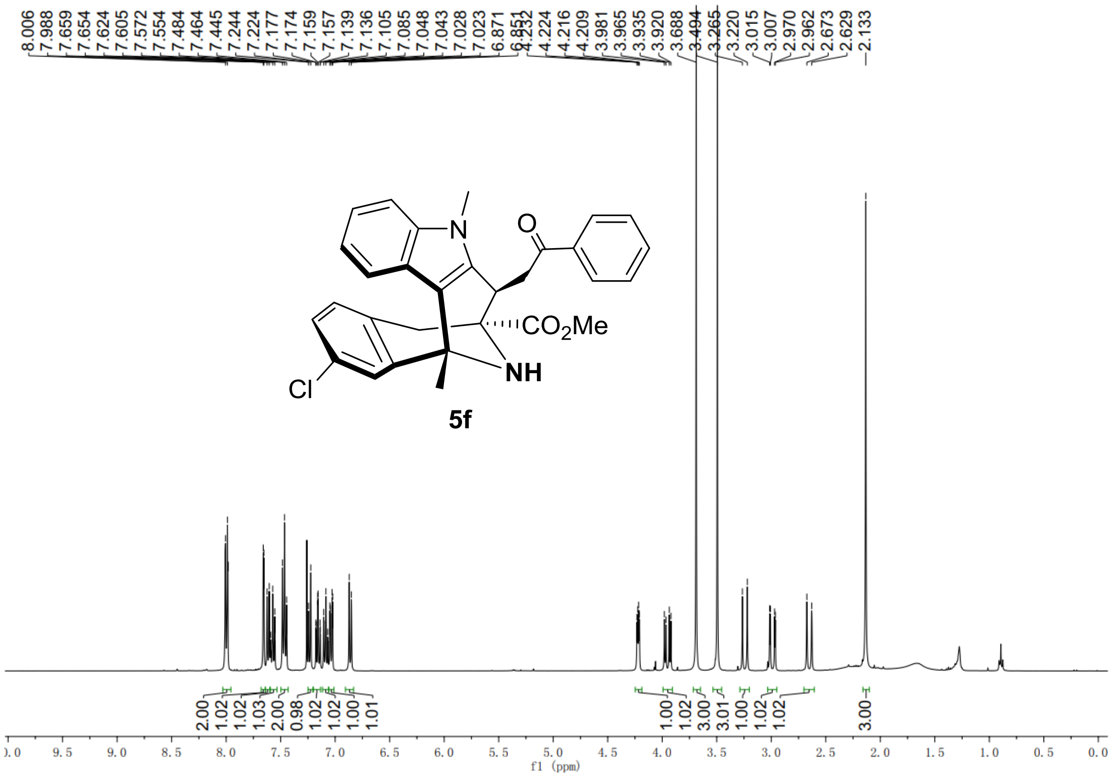

8.006
7.988
7.659
7.654
7.624
7.605
7.572
7.554
7.484
7.464
7.445
7.244
7.224
7.177
7.174
7.159
7.157
7.139
7.136
7.105
7.085
7.048
7.043
7.028
7.023
6.871
6.851
4.232
4.224
4.216
4.209
3.981
3.965
3.935
3.920
3.688
3.494
3.265
3.220
3.015
3.007
2.970
2.962
2.673
2.629
2.133
Cl
N
O
CO2Me
NH
5f
2.00
1.02
1.02
1.03
2.00
0.98
1.02
1.02
1.00
1.01
1.00
1.02
3.00
3.01
1.00
1.02
1.02
3.00
f1 (ppm)

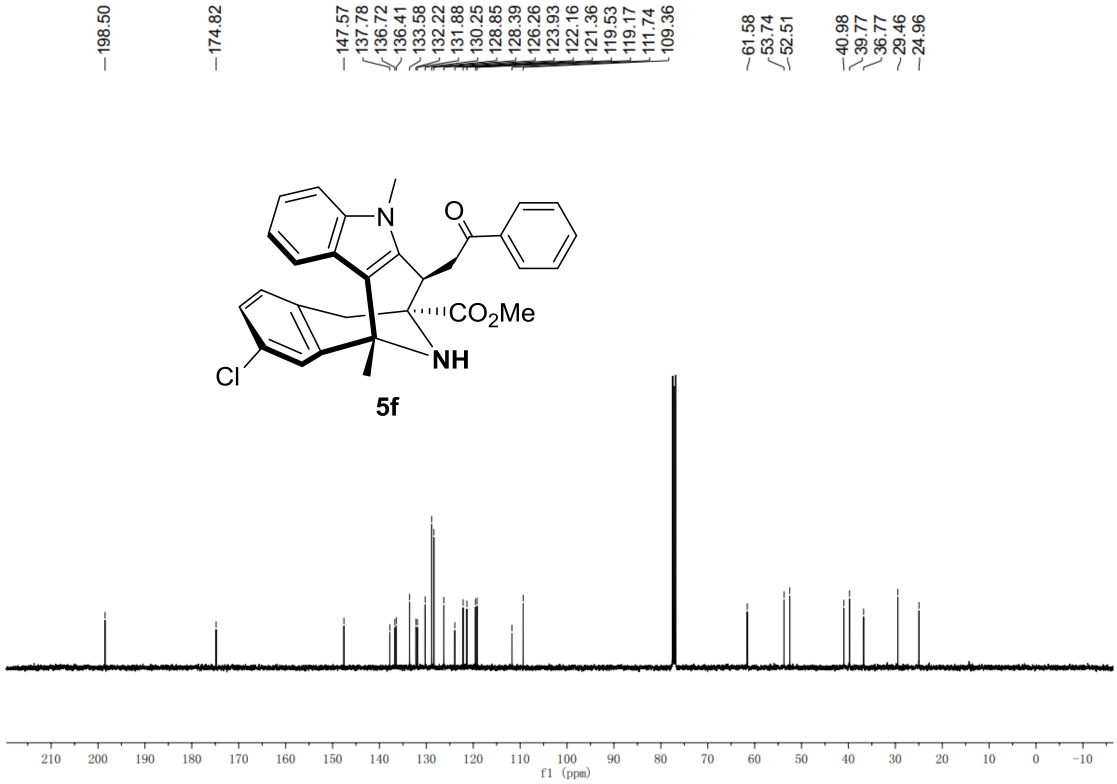


$^1$H-NMR and $^{13}$C-NMR of **5f**

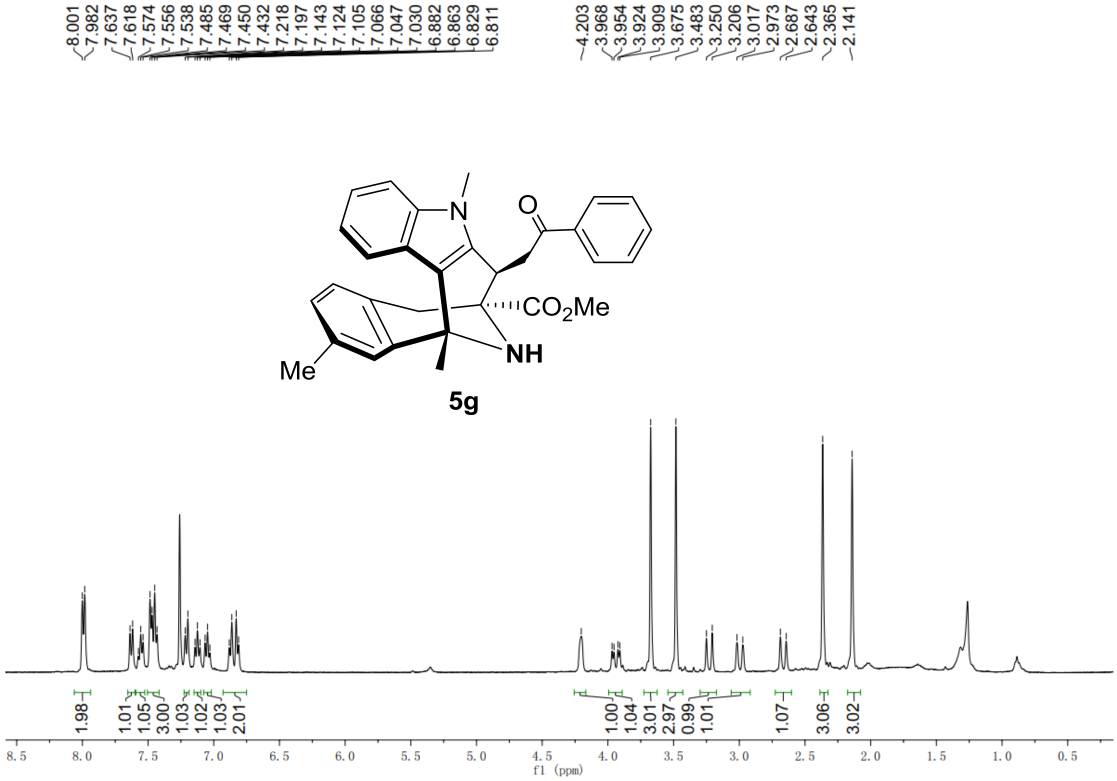

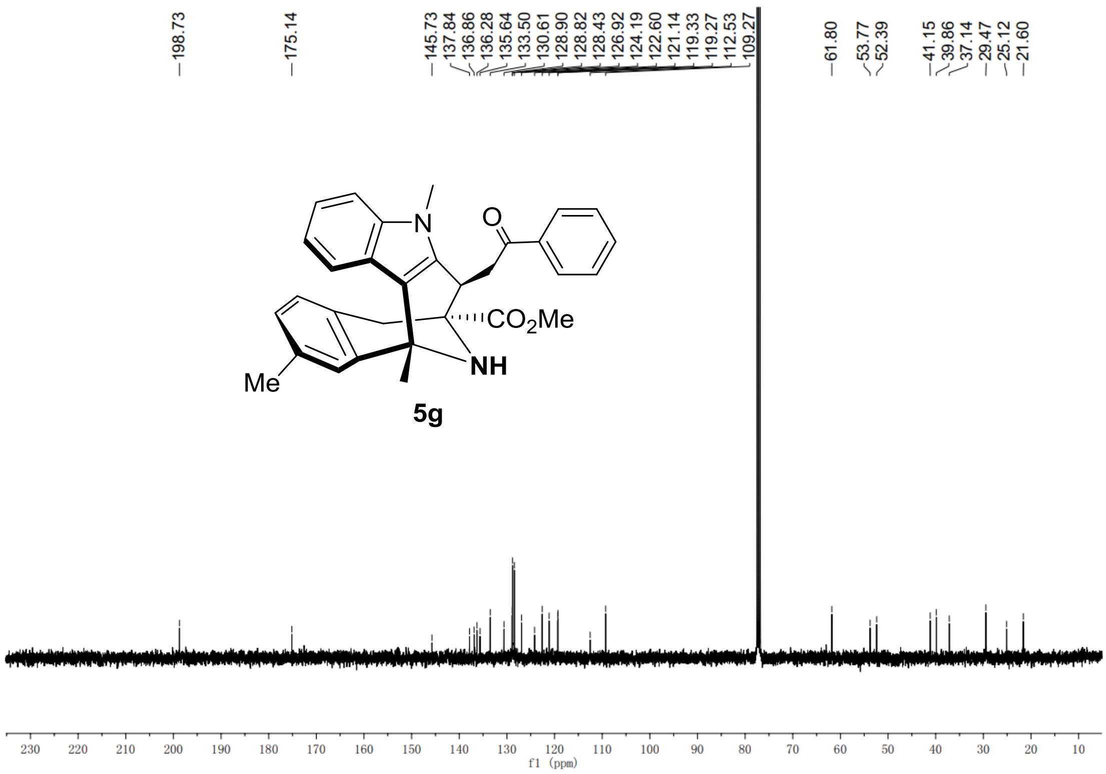


$^1$H-NMR and $^{13}$C-NMR of **5g**

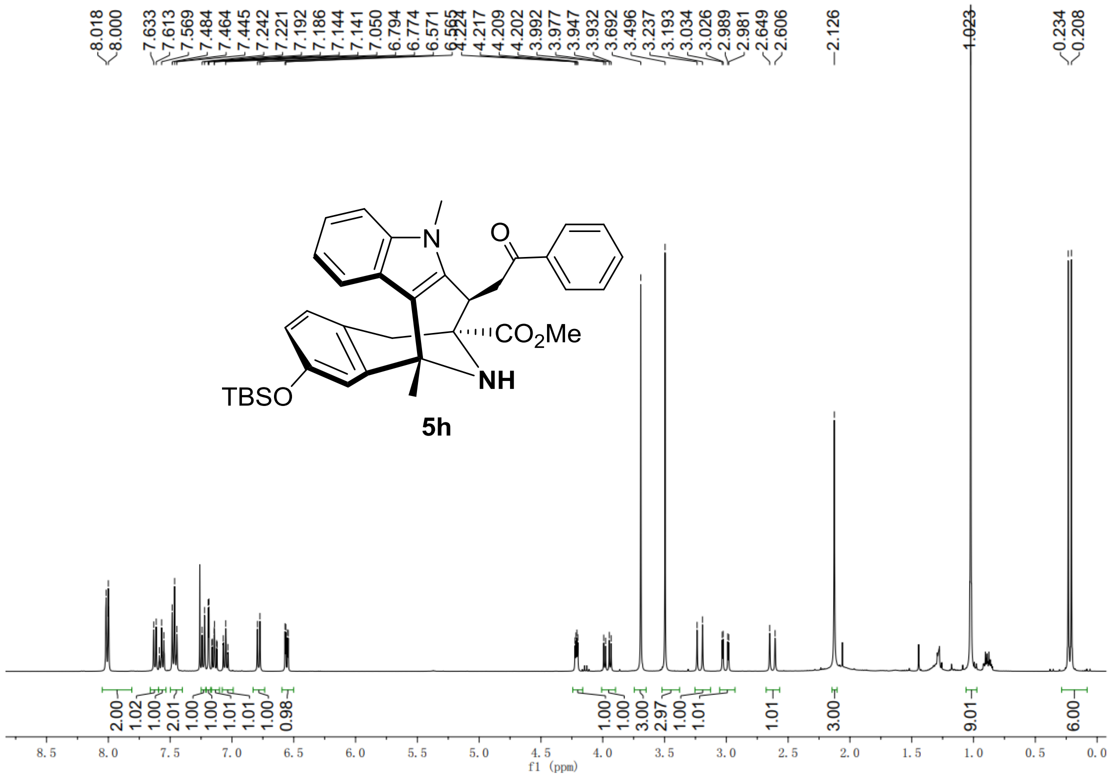

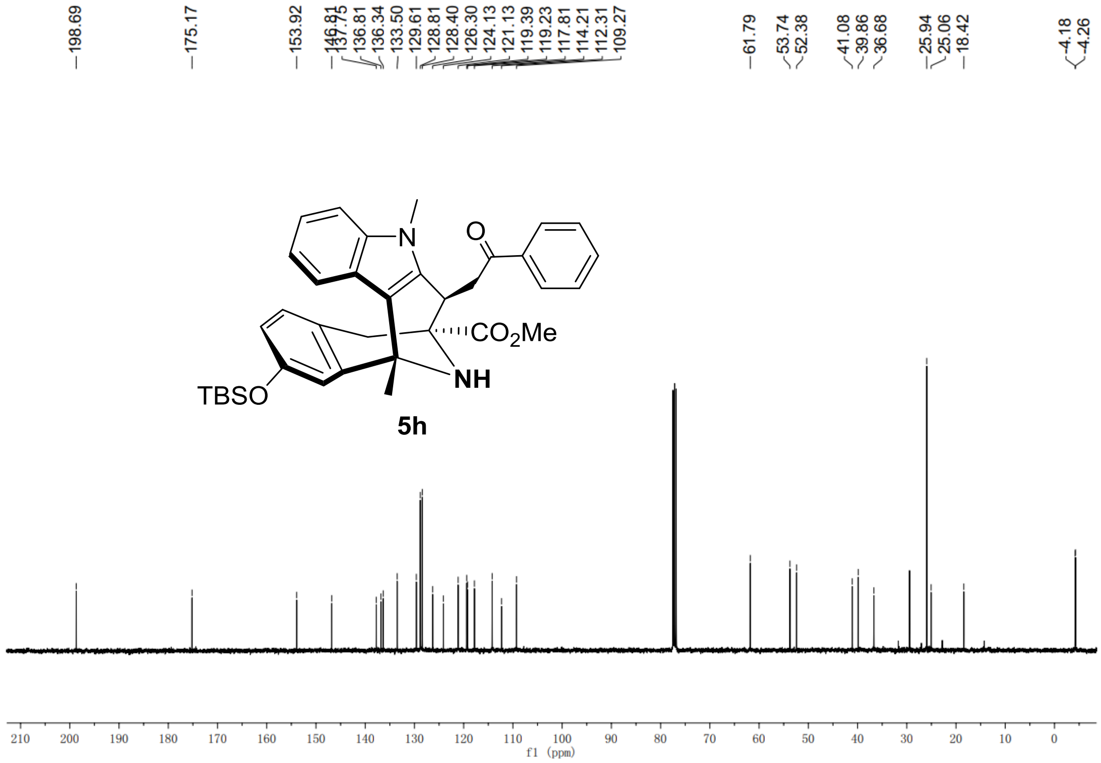


$^{1}$H-NMR and $^{13}$C-NMR of **5h**

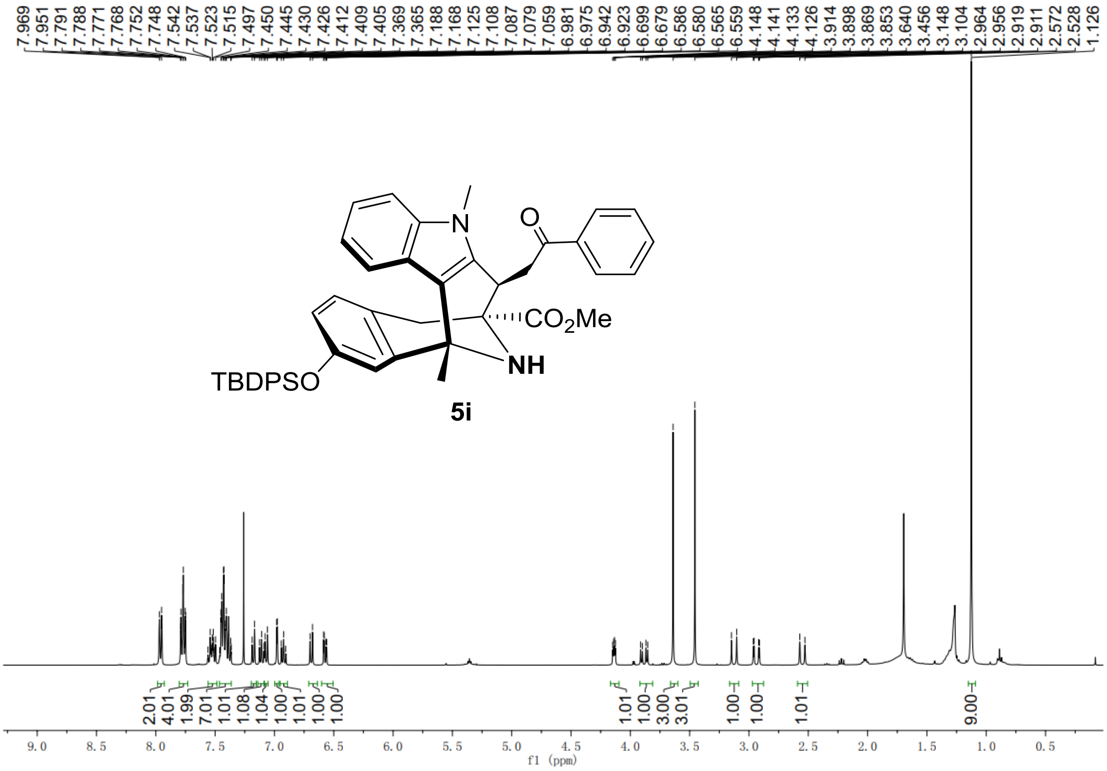

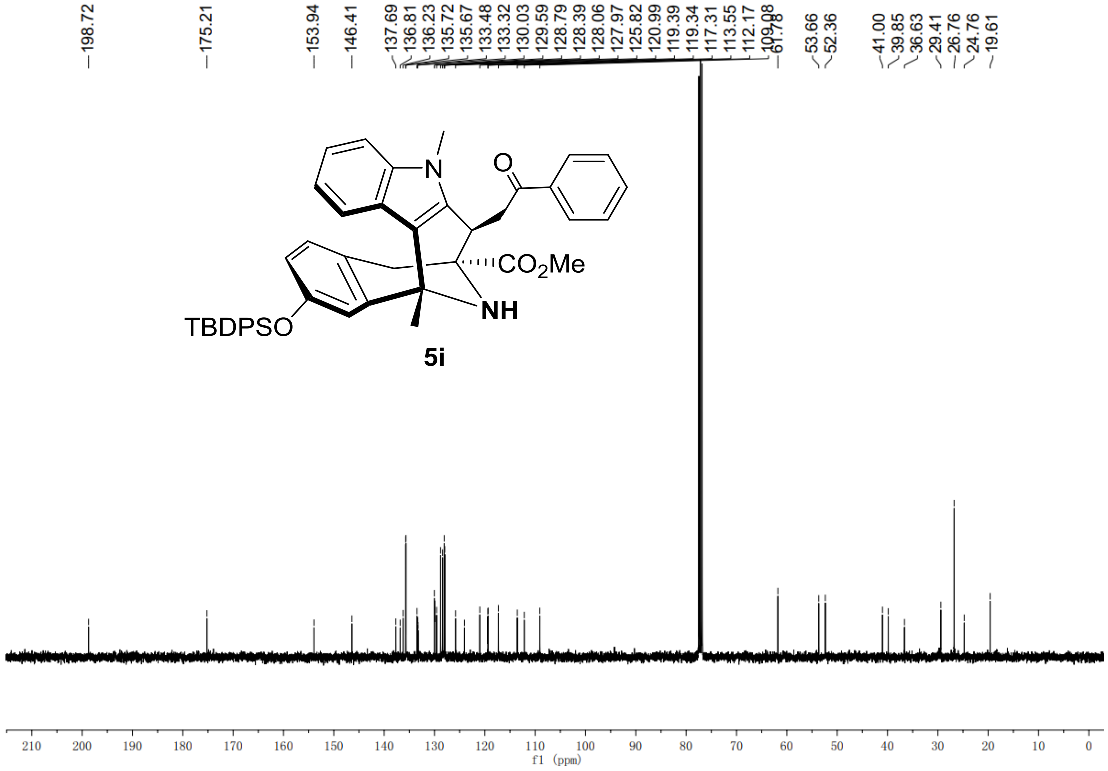


$^{1}$H-NMR and $^{13}$C-NMR of **5i**

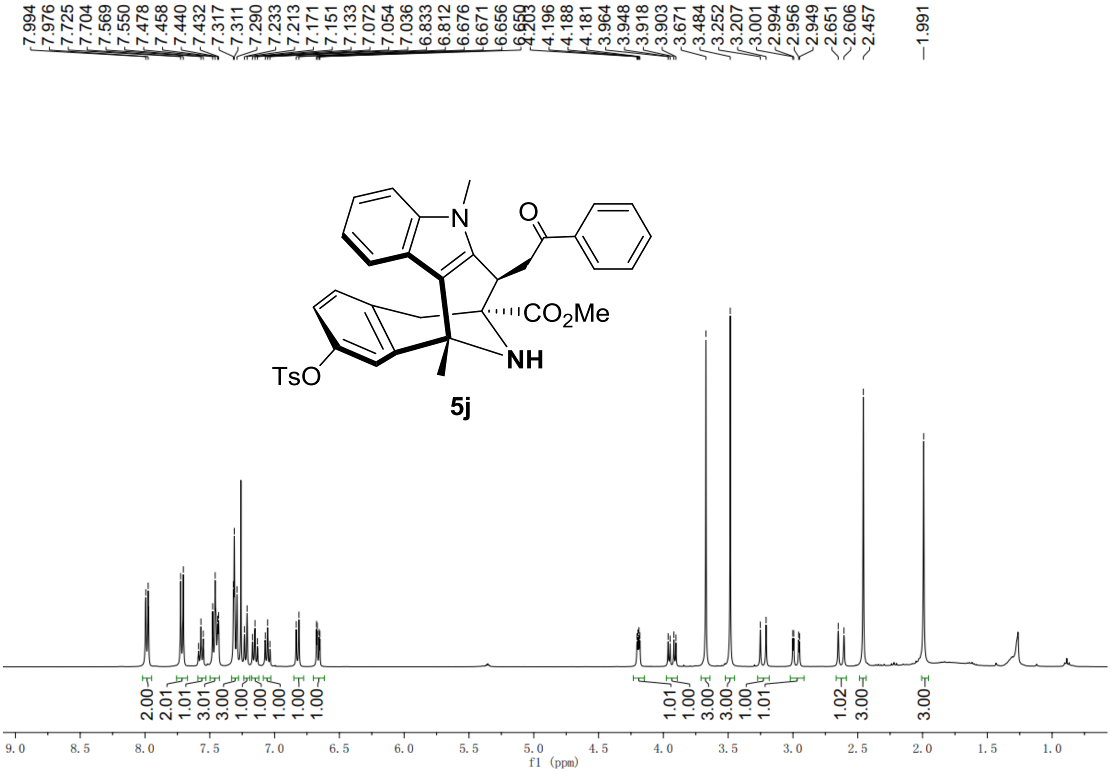

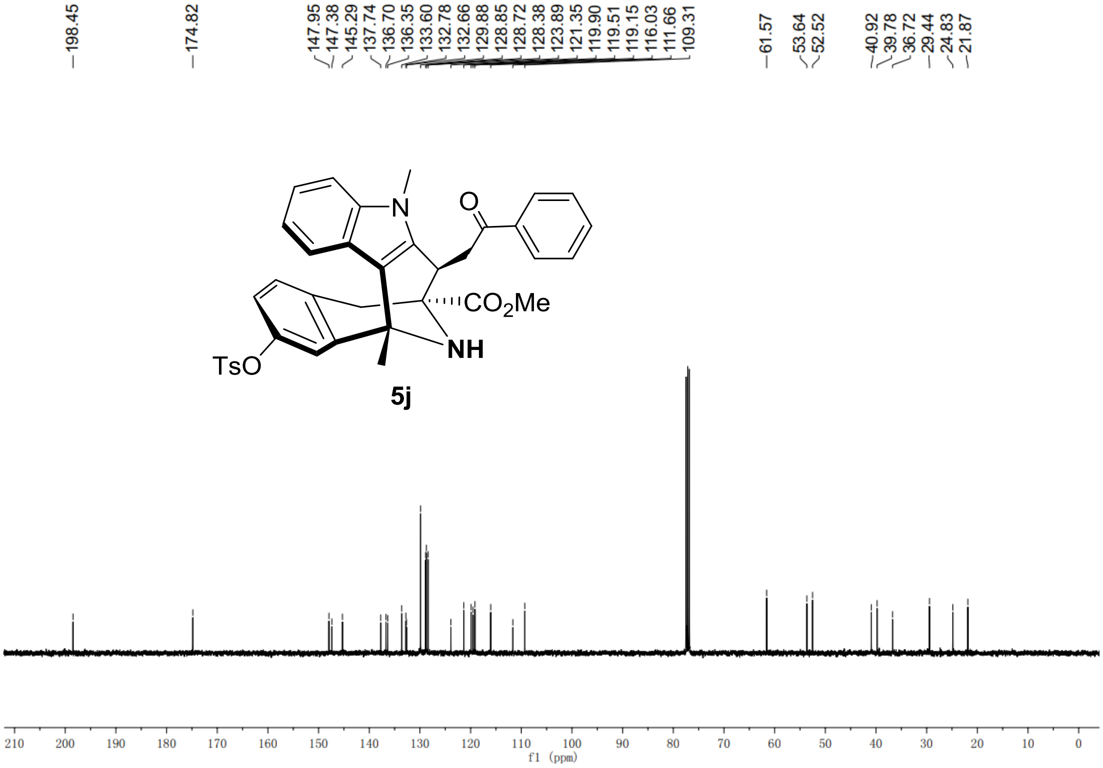


$^{1}$H-NMR and $^{13}$C-NMR of **5j**

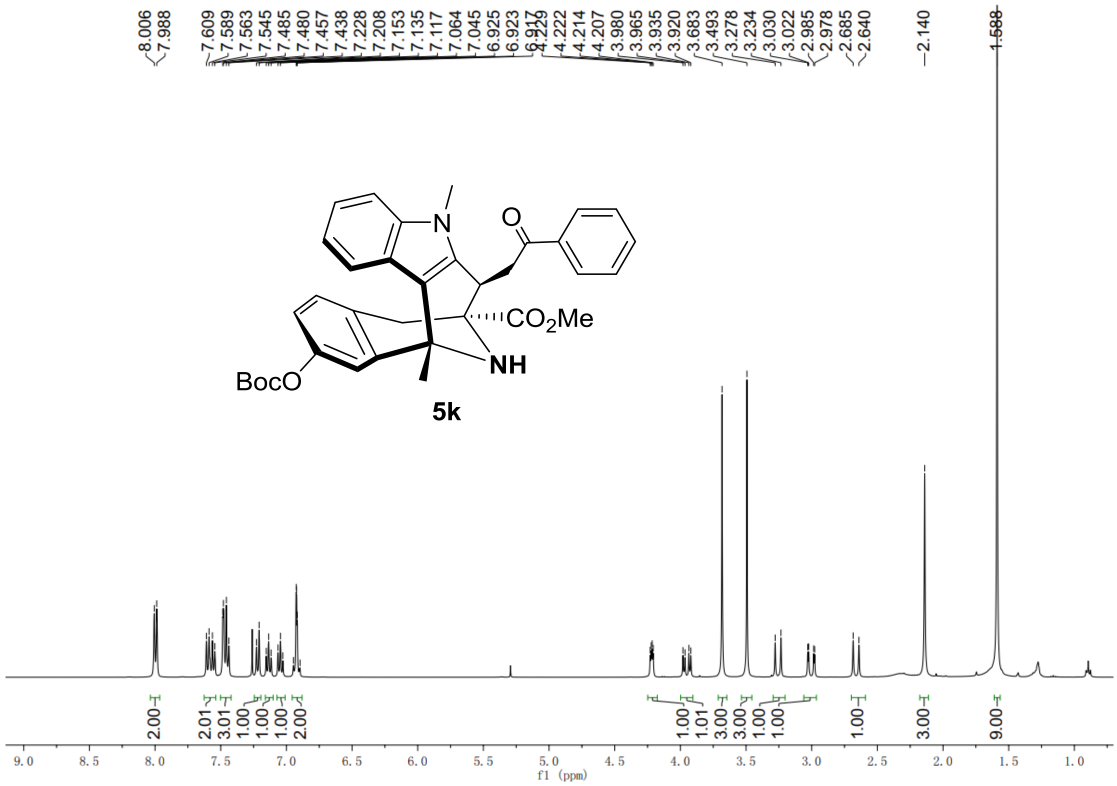

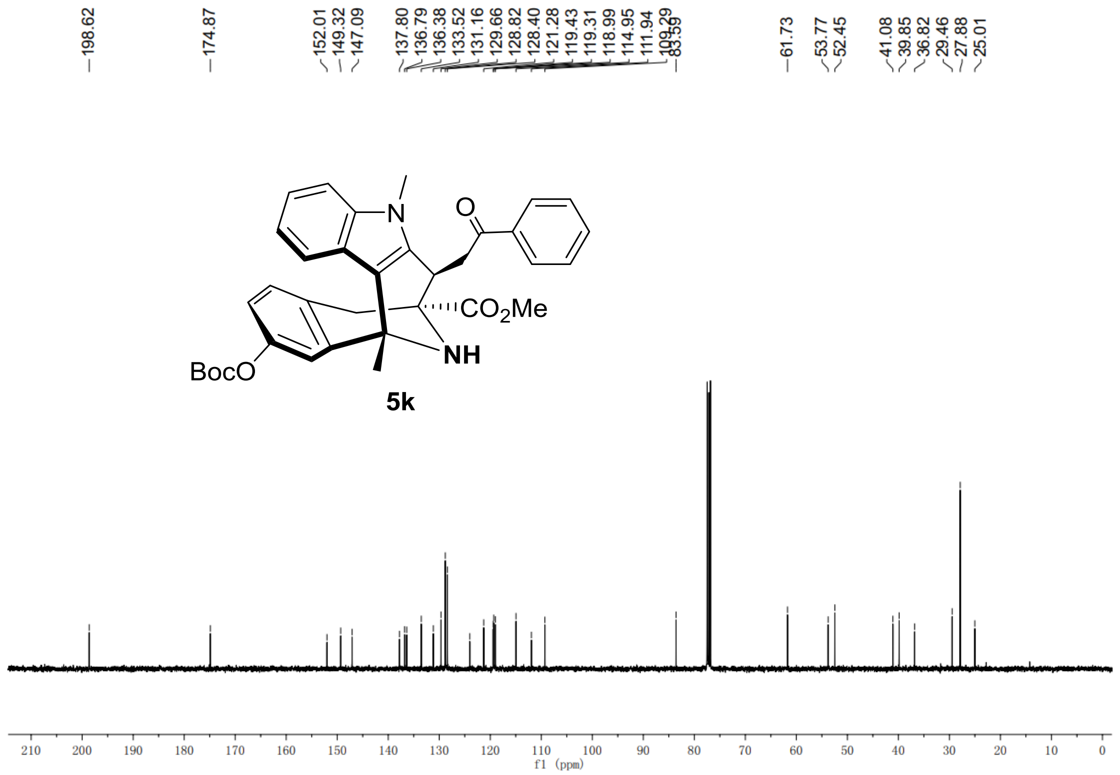


$^{1}$H-NMR and $^{13}$C-NMR of **5k**

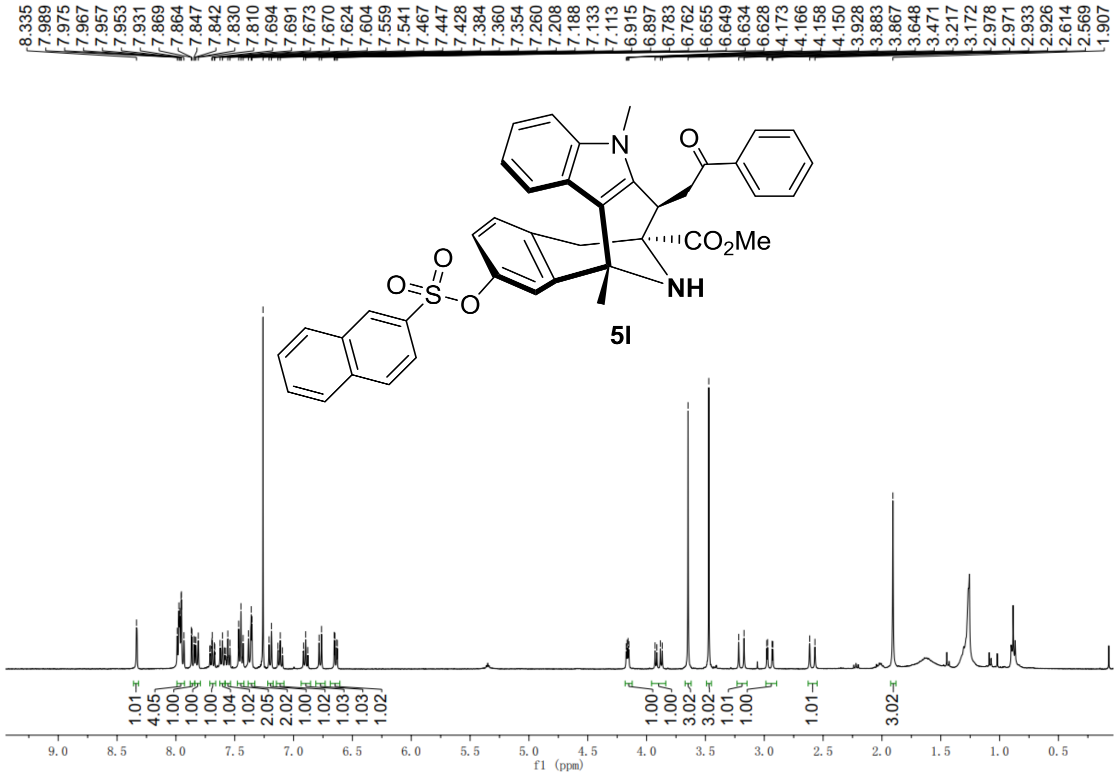

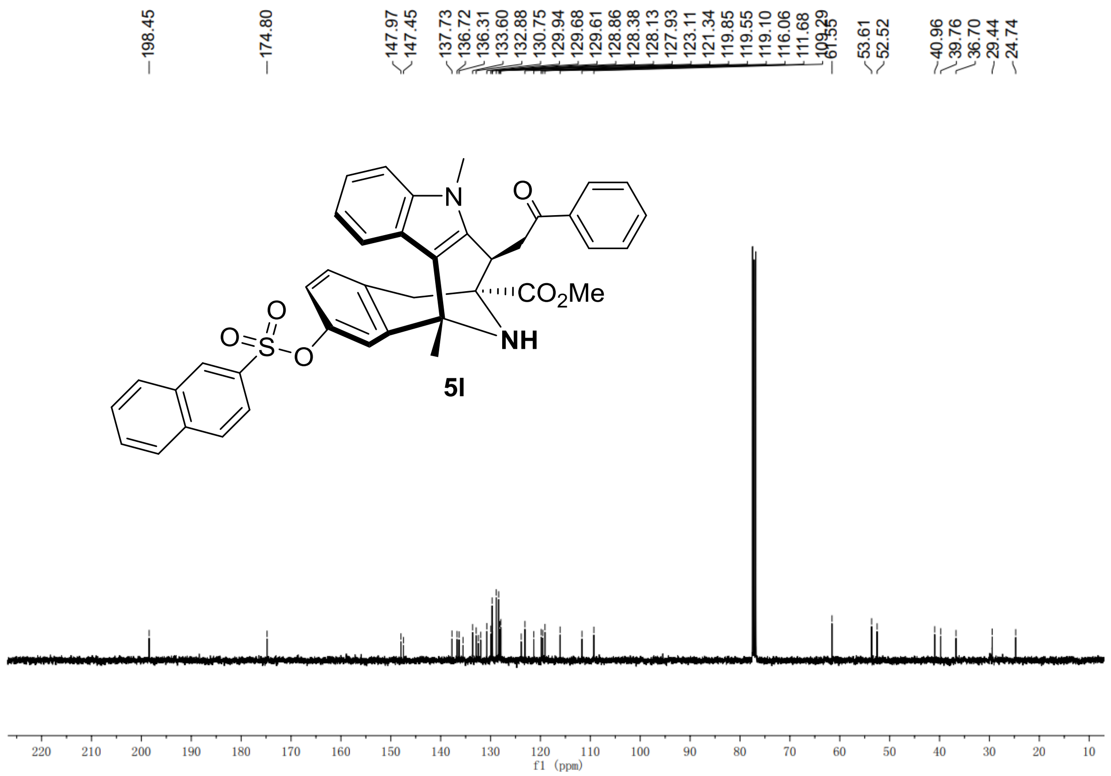


$^1$H-NMR and $^{13}$C-NMR of **5l**

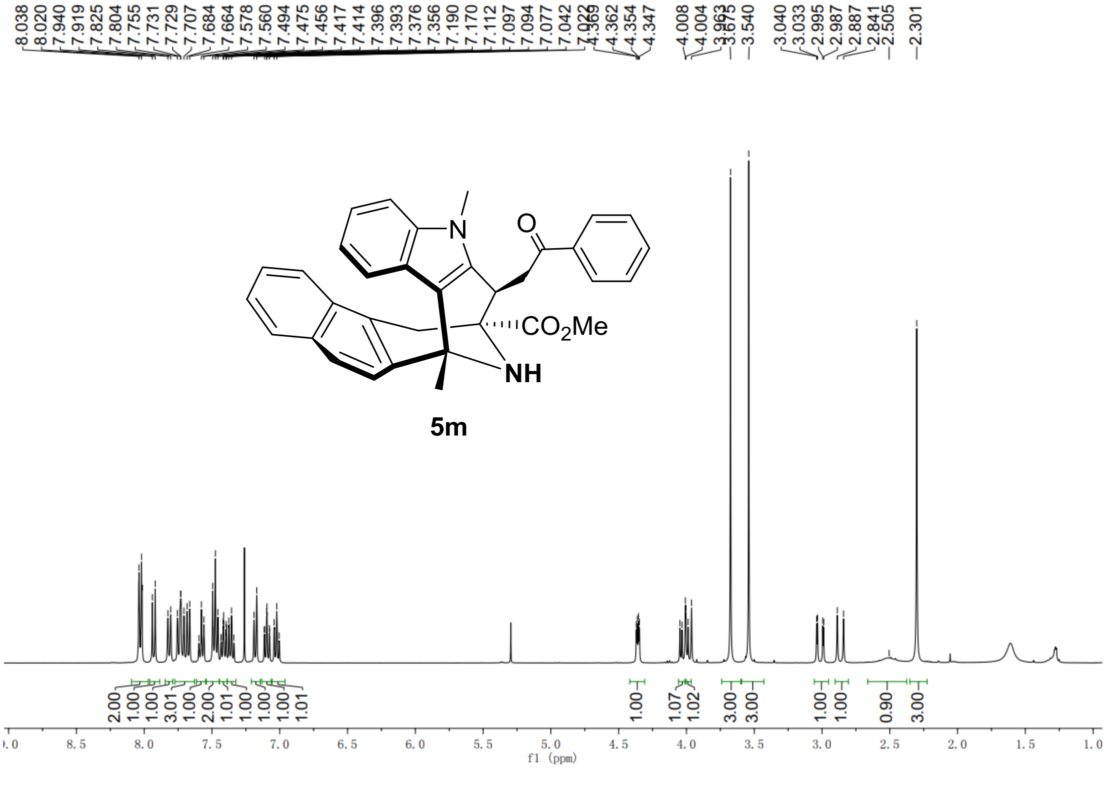

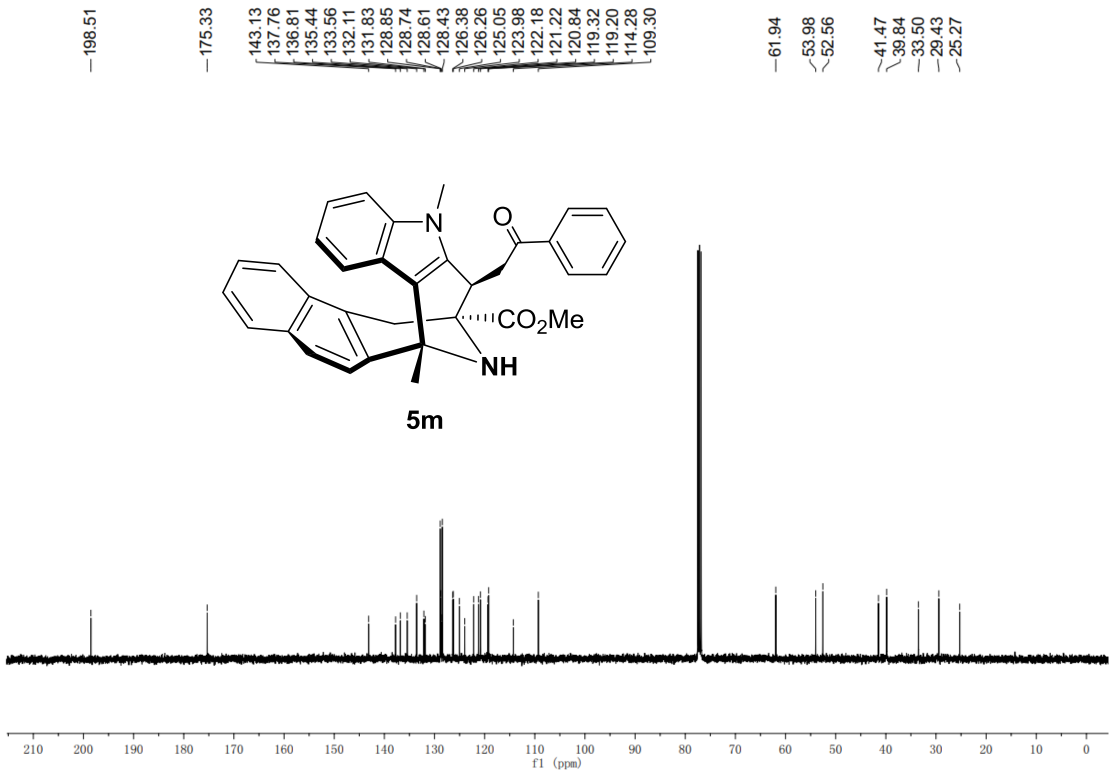


$^{1}$H-NMR and $^{13}$C-NMR of **5m**

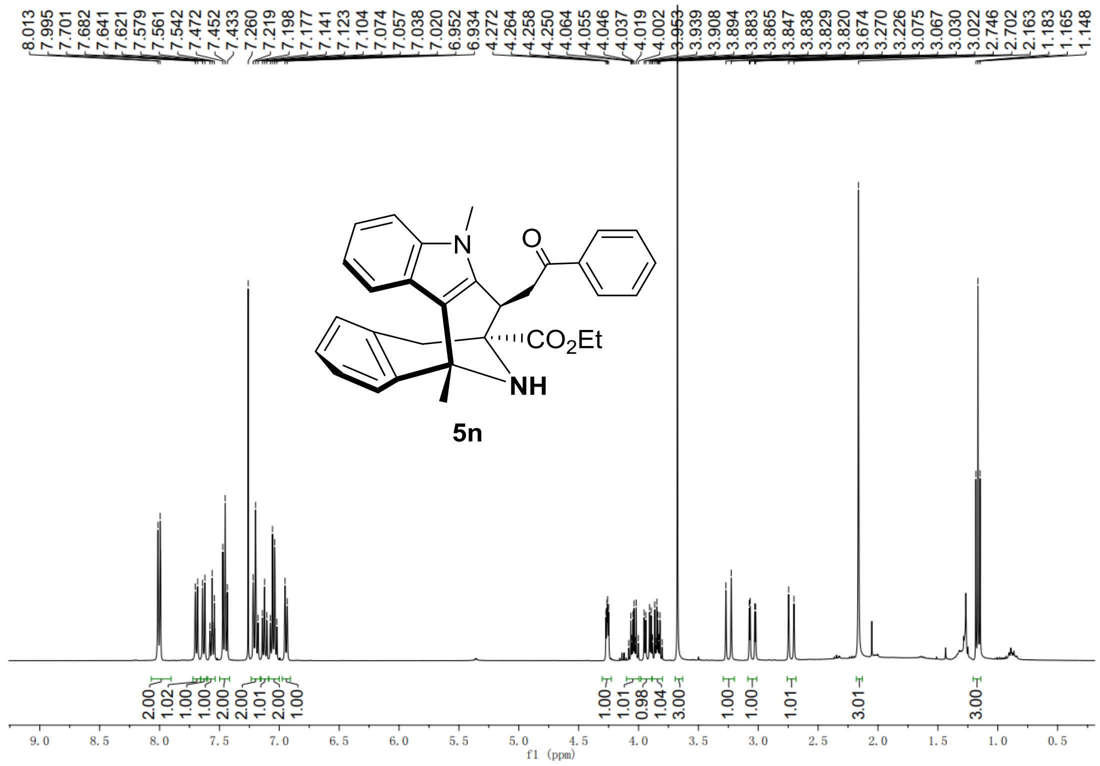

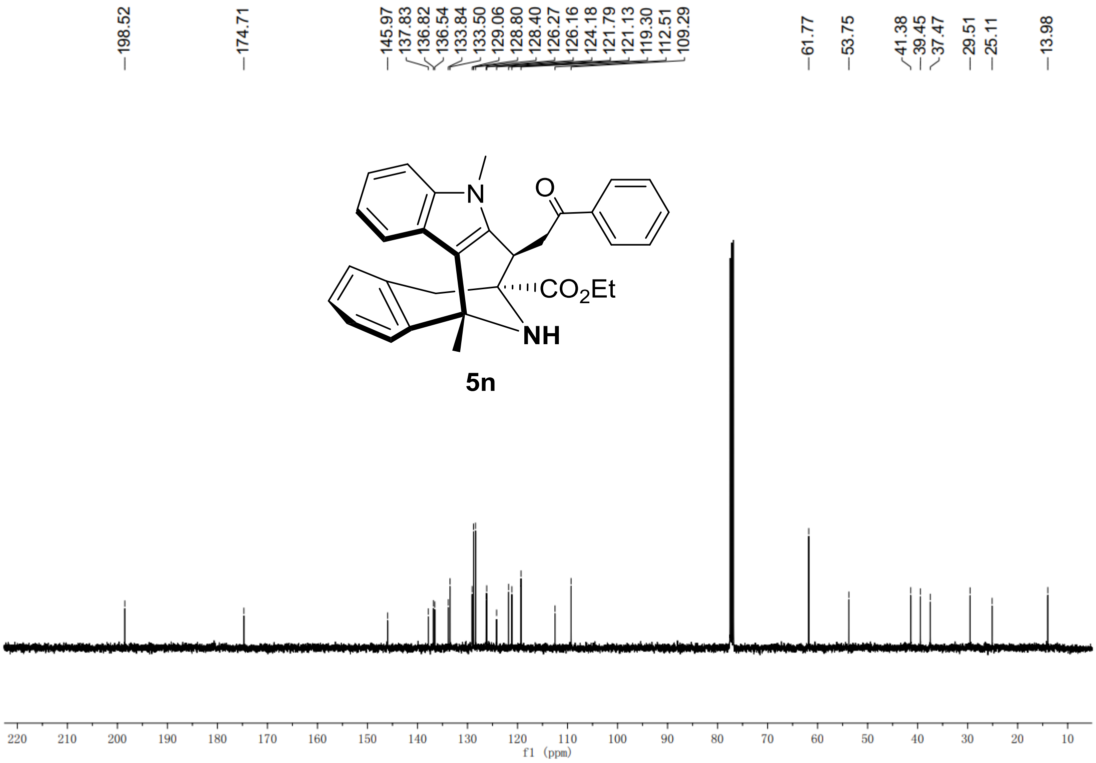


$^1$H-NMR and $^{13}$C-NMR of **5n**

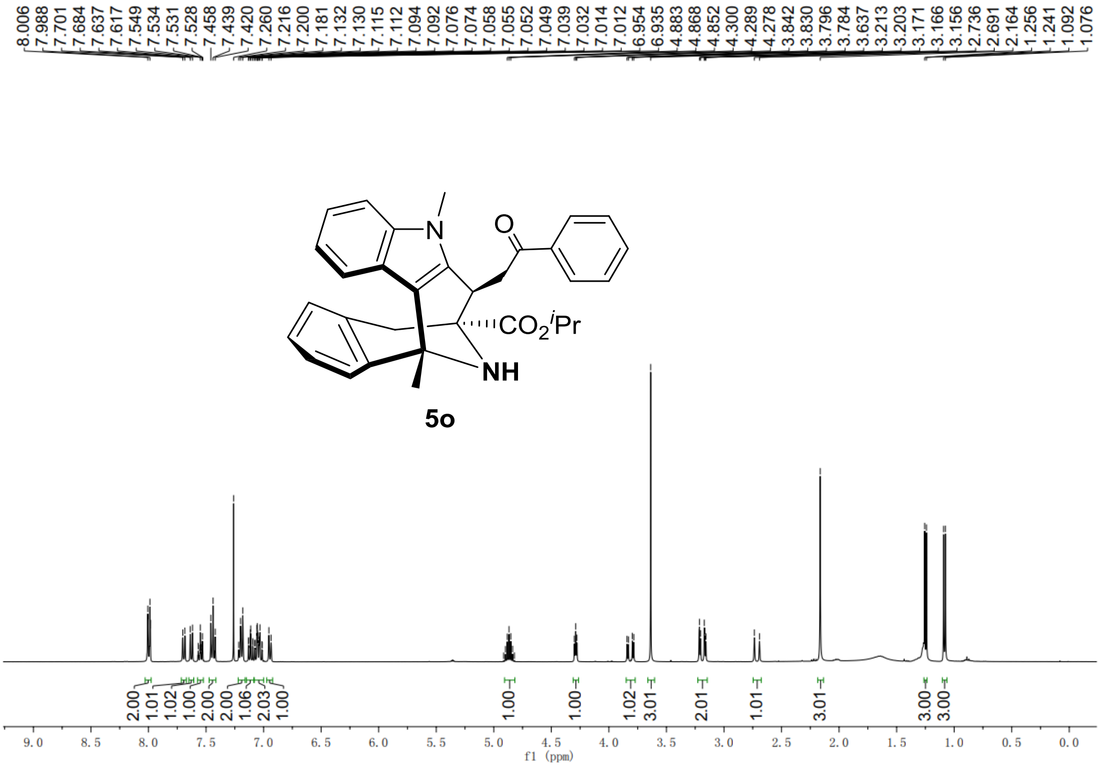

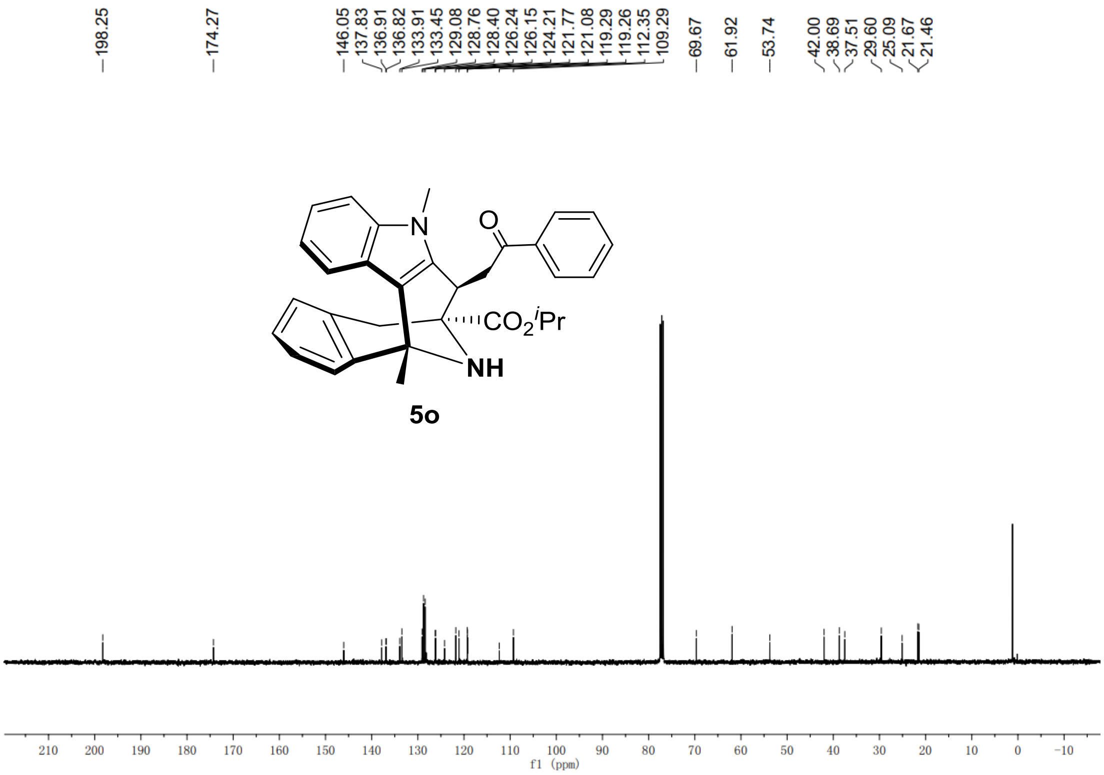


$^{1}$H-NMR and $^{13}$C-NMR of **5o**

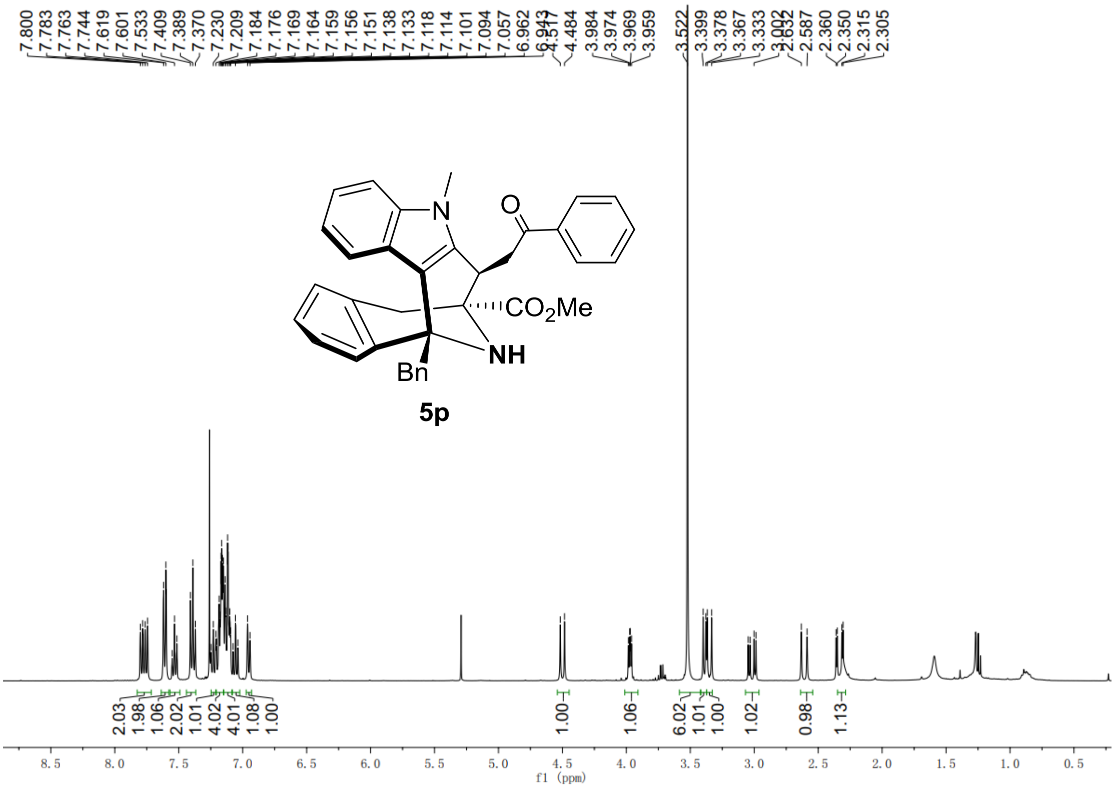

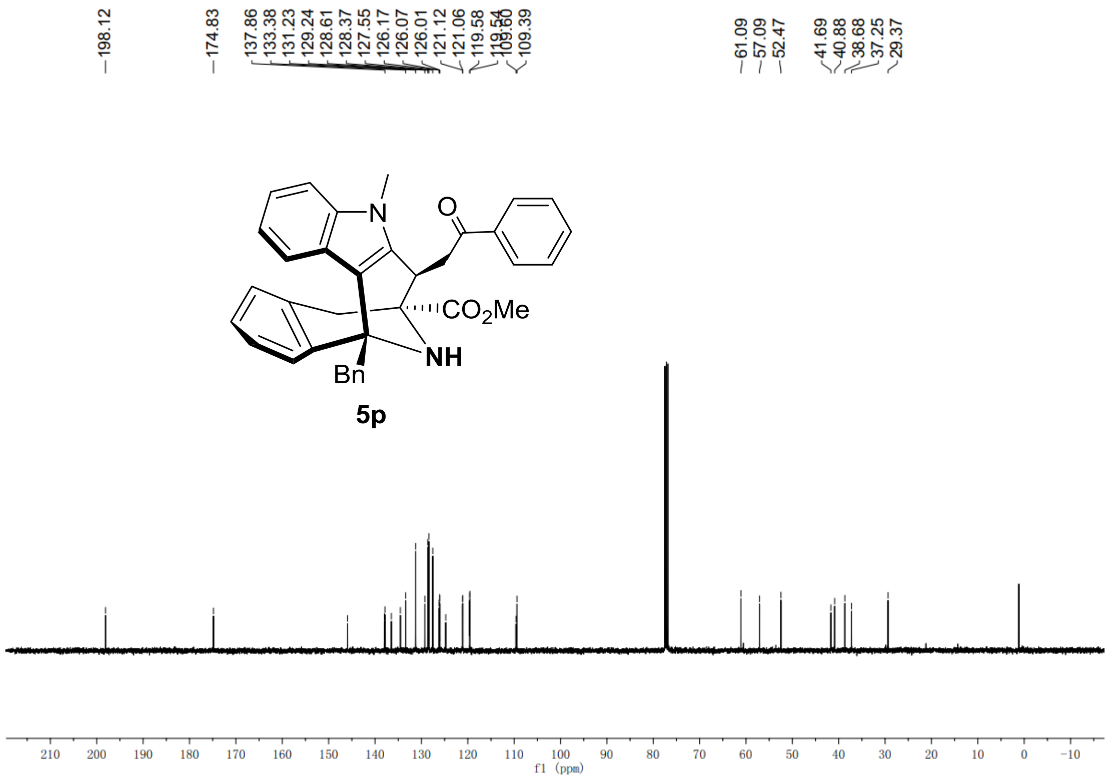


$^1$H-NMR and $^{13}$C-NMR of **5p**

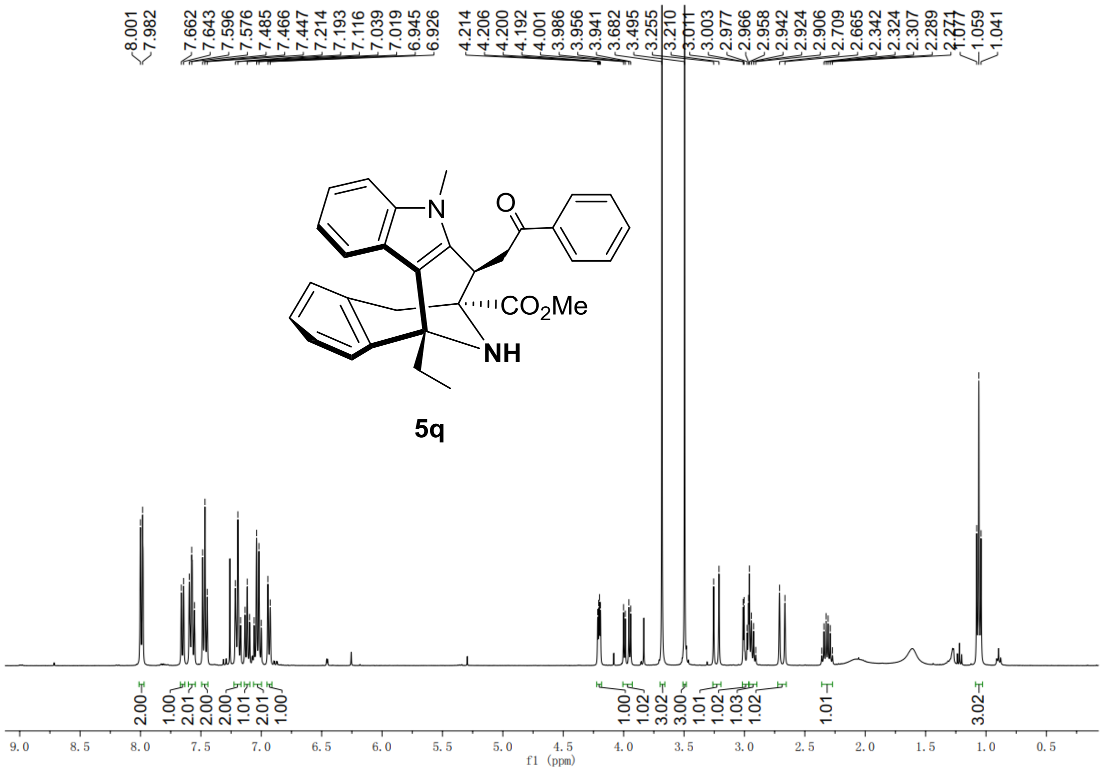

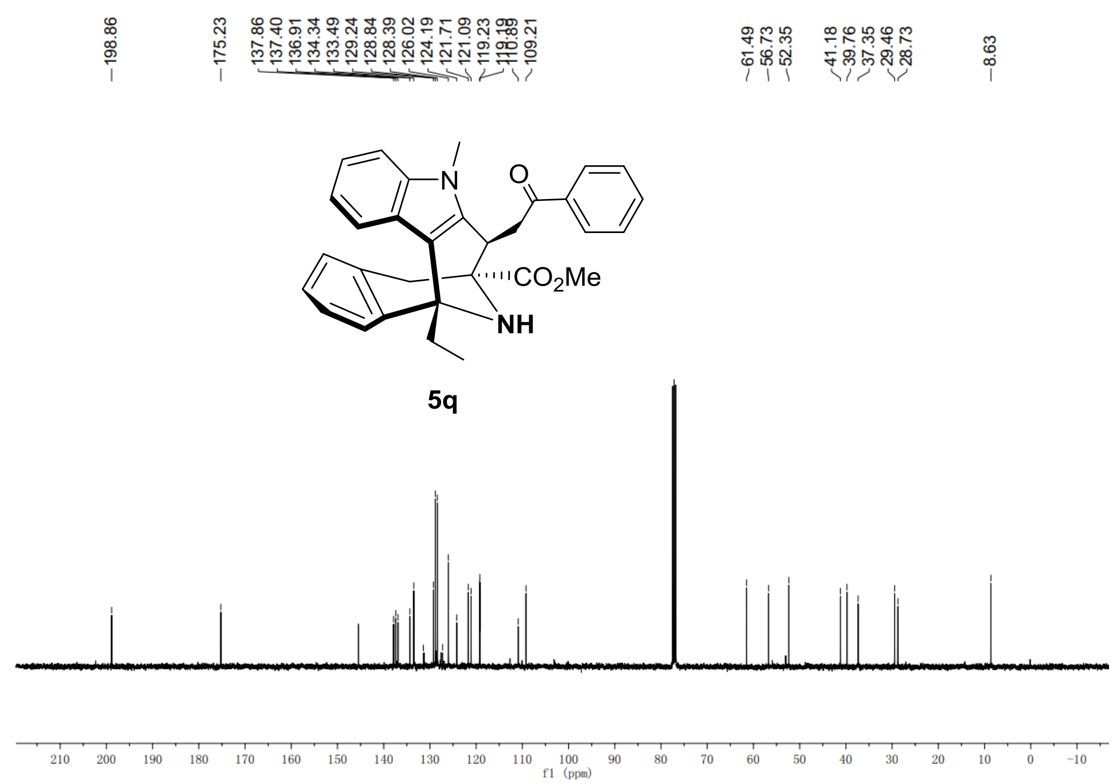


$^{1}$H-NMR and $^{13}$C-NMR of **5q**

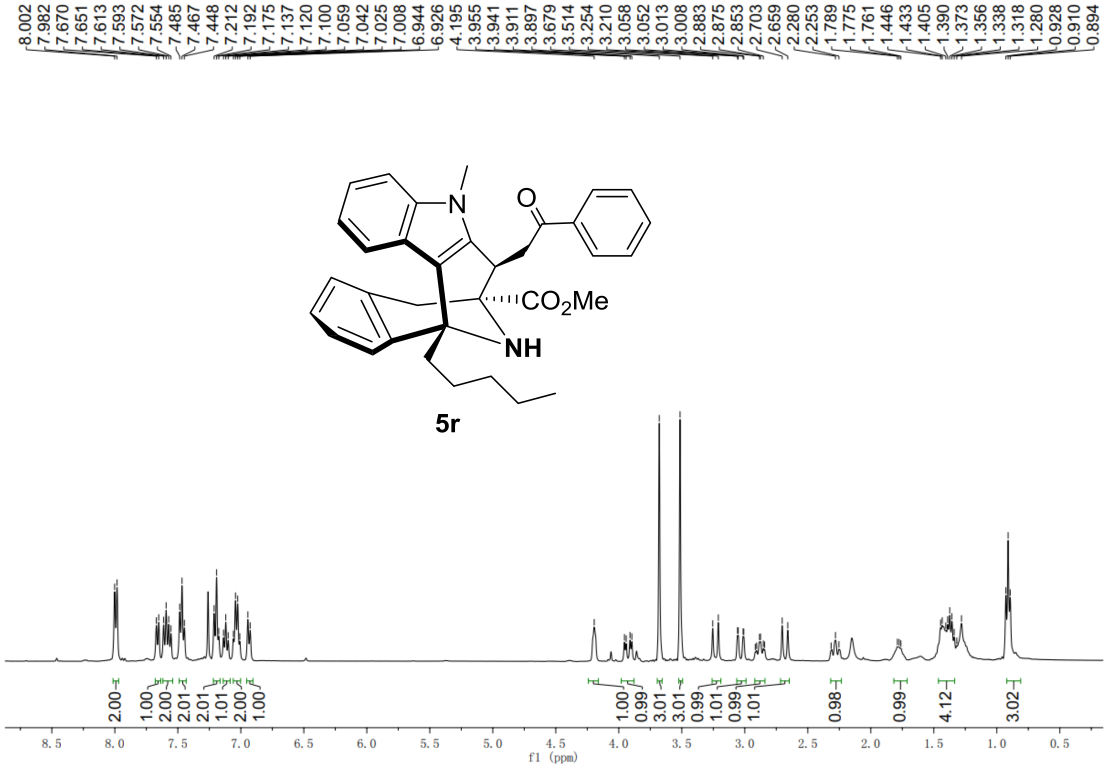

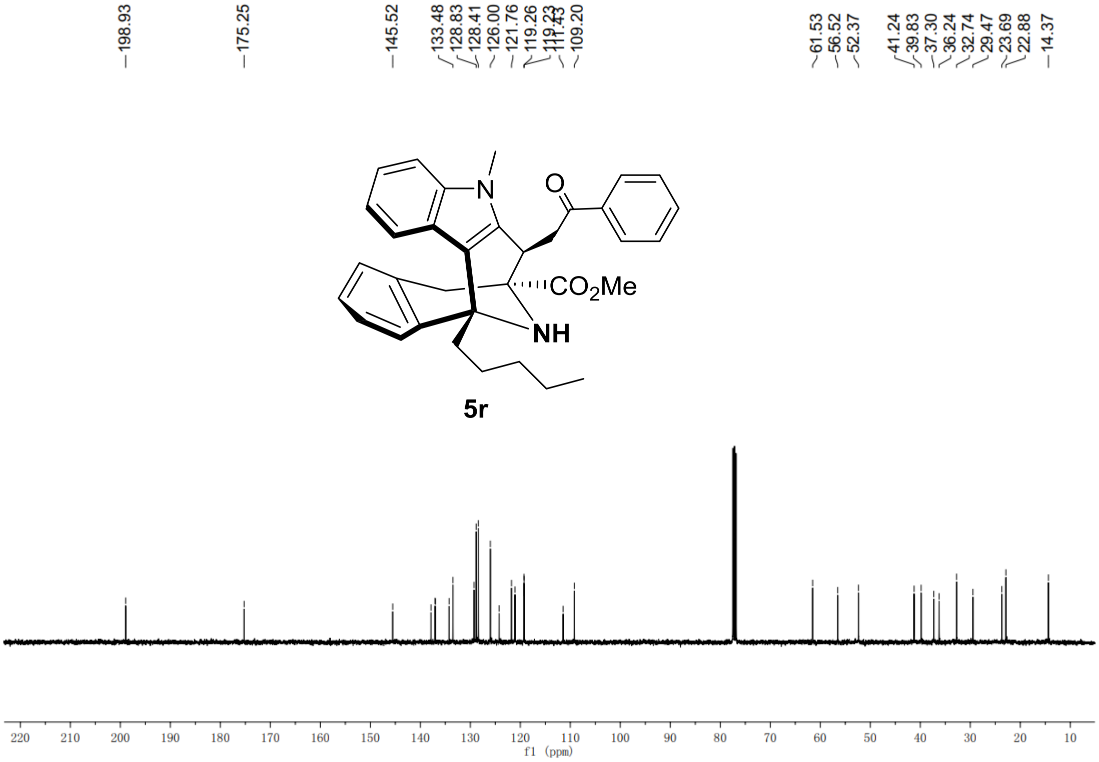


$^{1}$H-NMR and $^{13}$C-NMR of **5r**

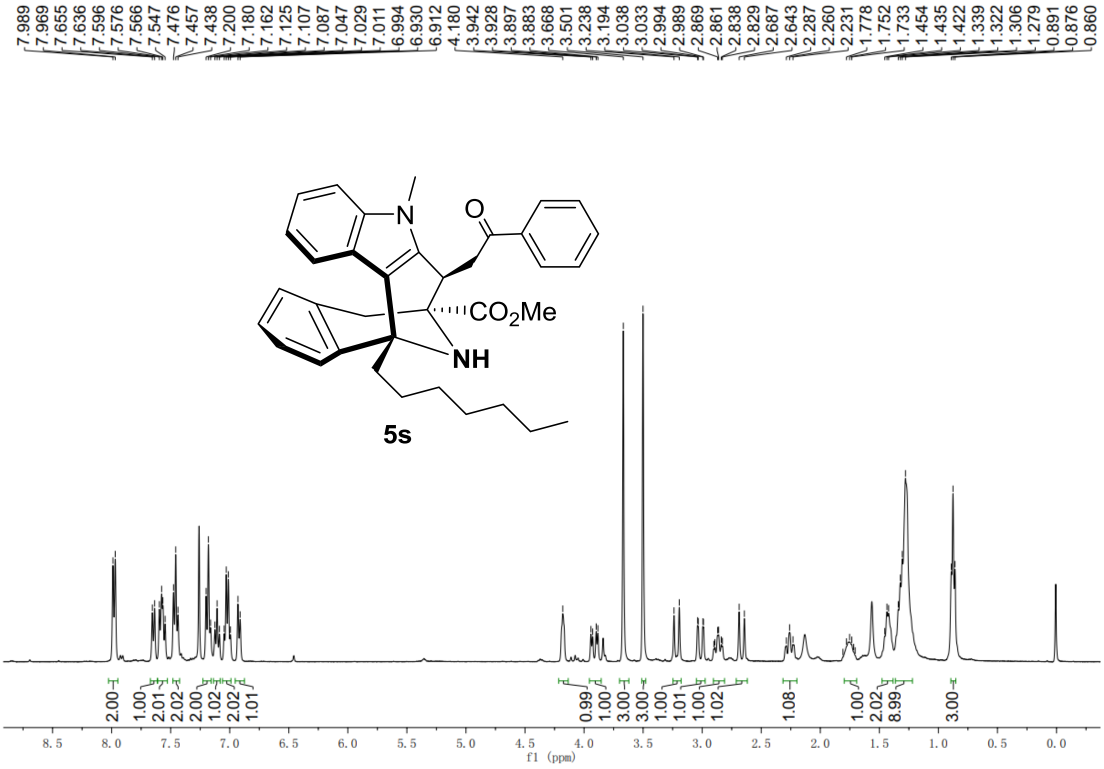

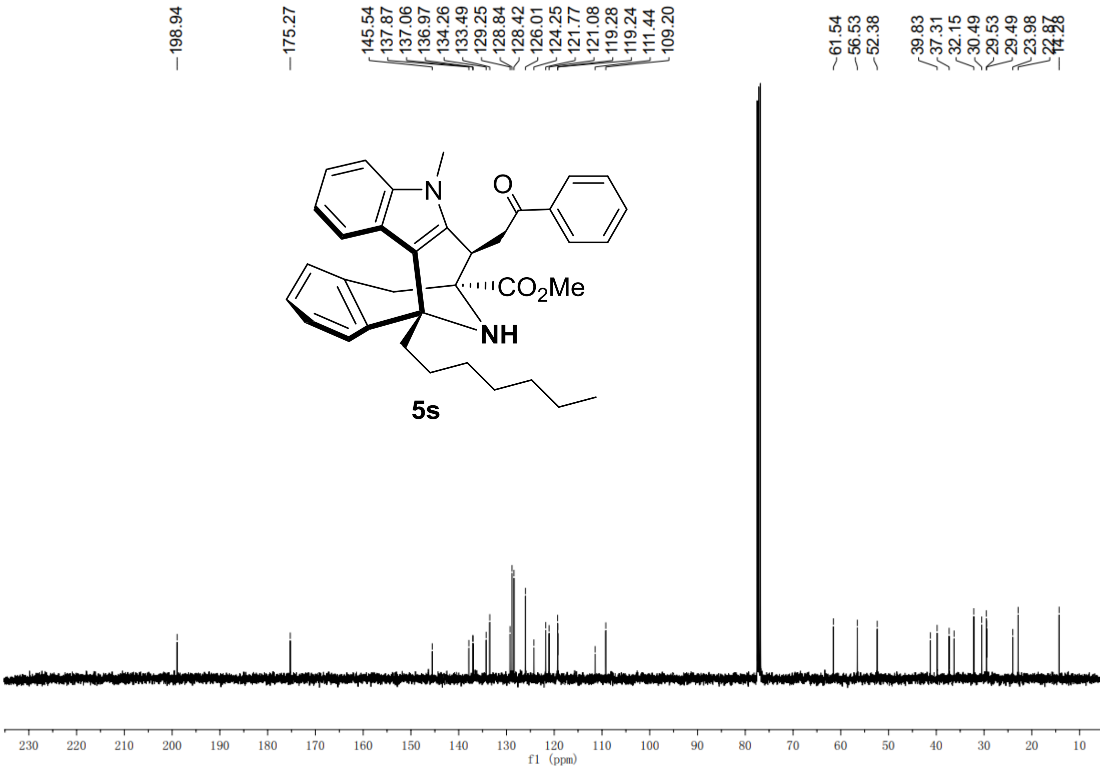


$^{1}$H-NMR and $^{13}$C-NMR of **5s**

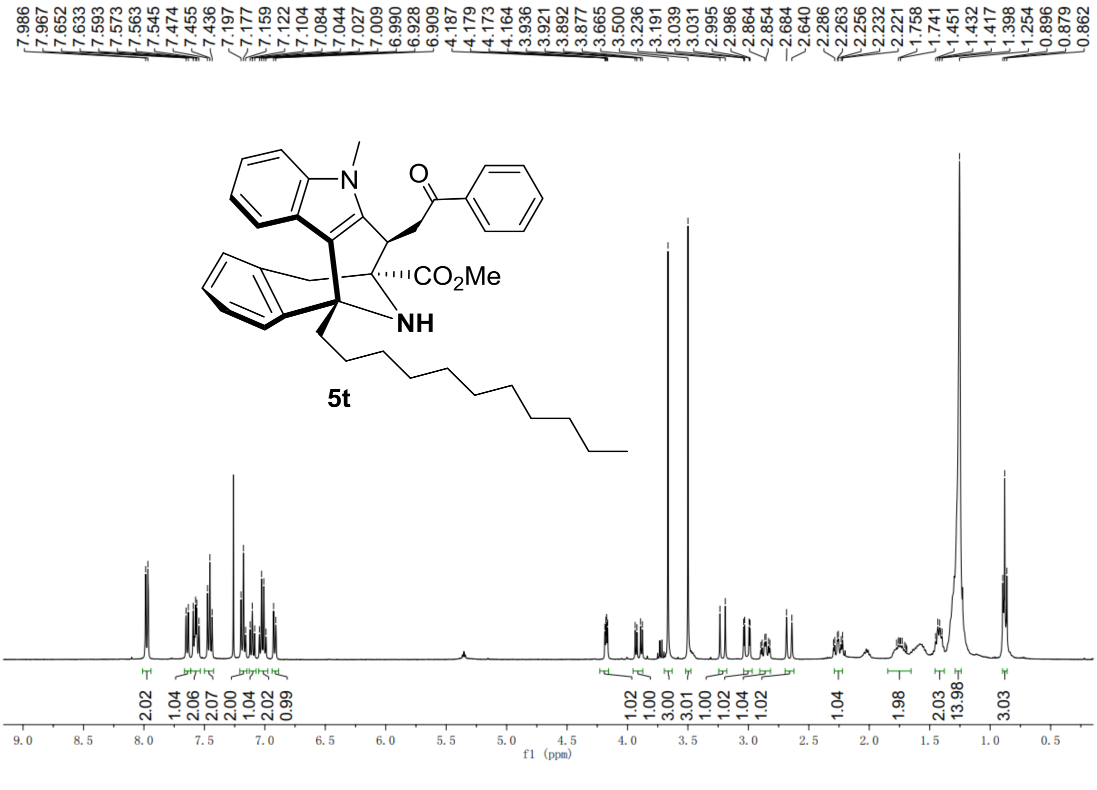

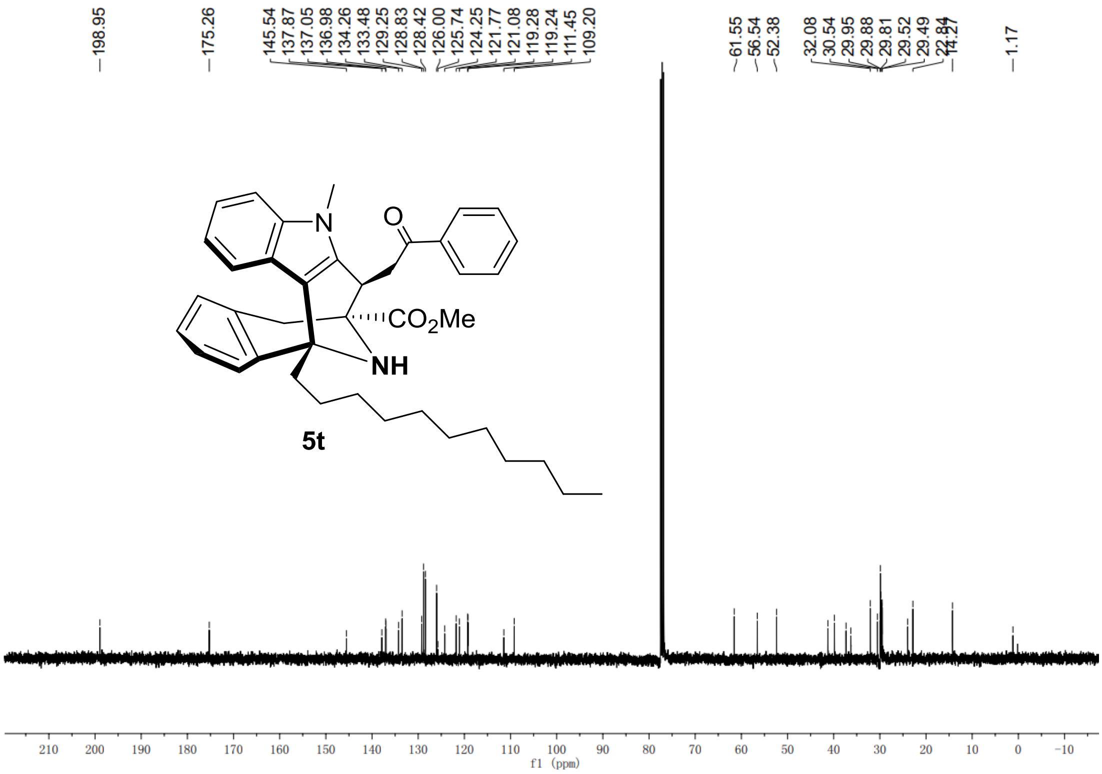


$^{1}$H-NMR and $^{13}$C-NMR of **5t**

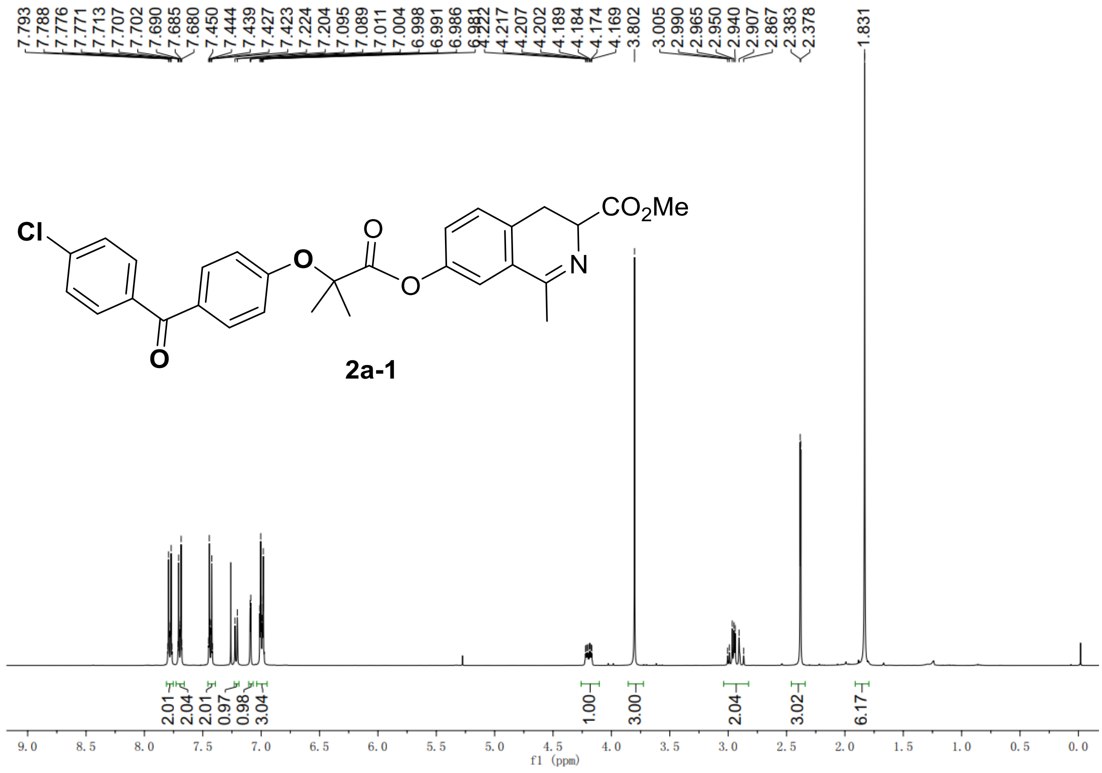

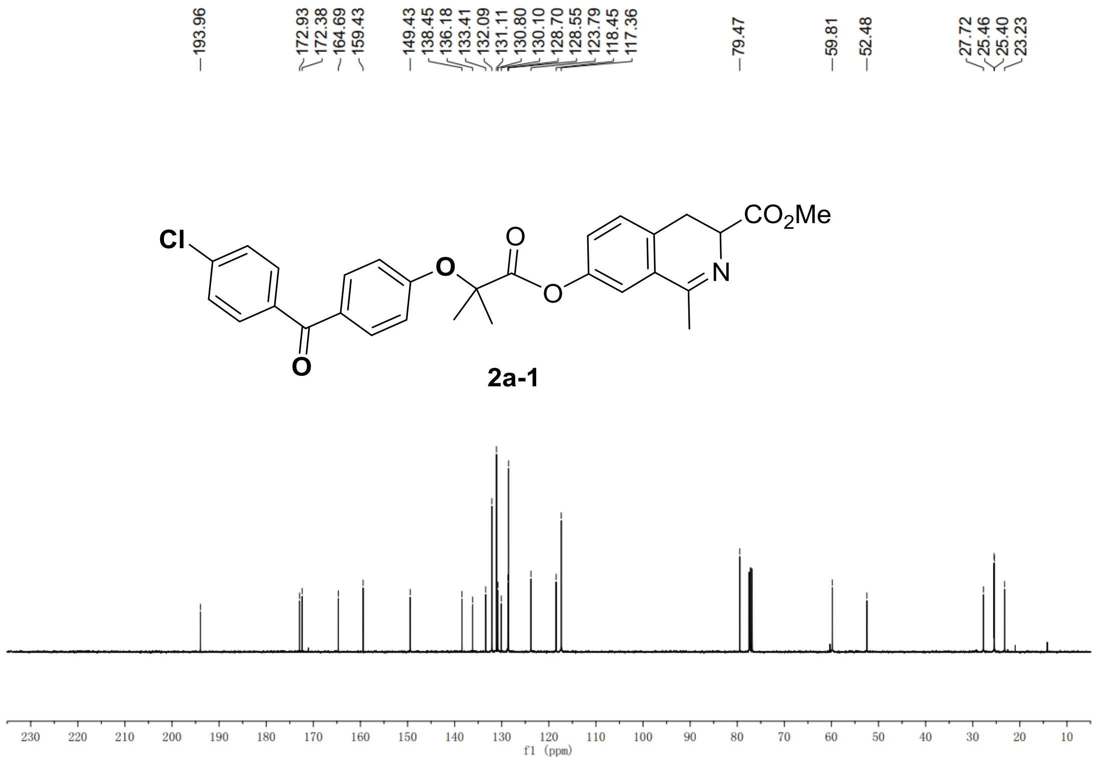


$^1$H-NMR and $^{13}$C-NMR of **2a-1**

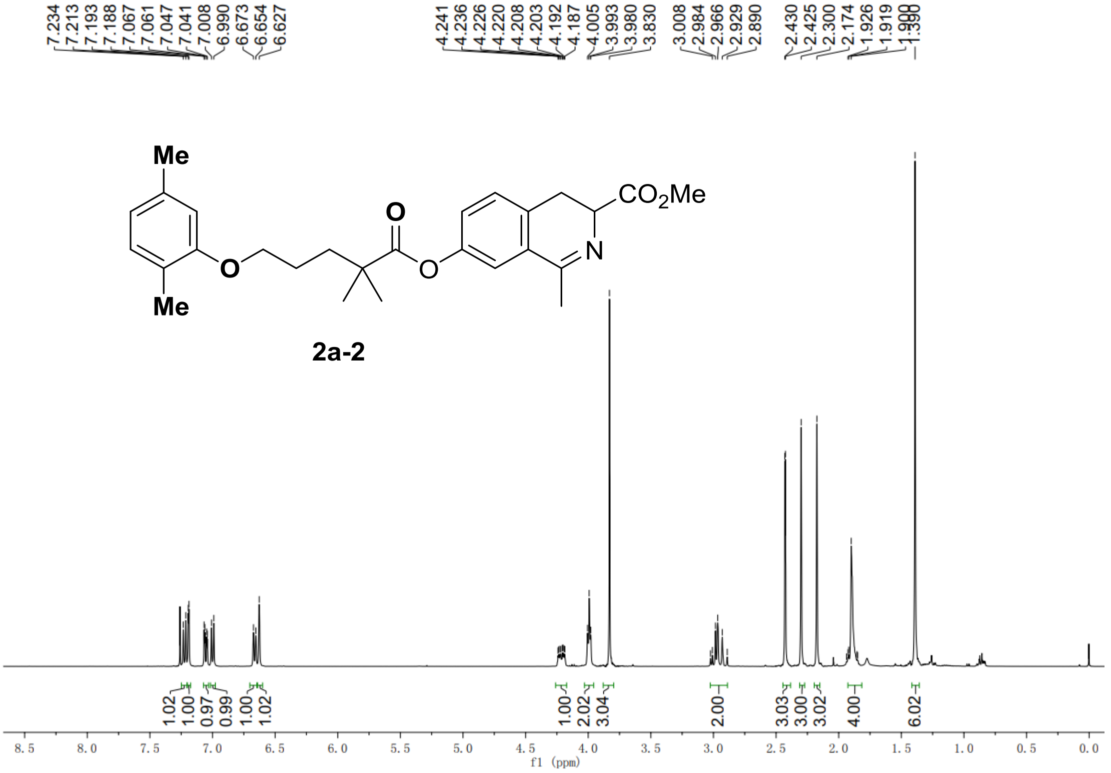

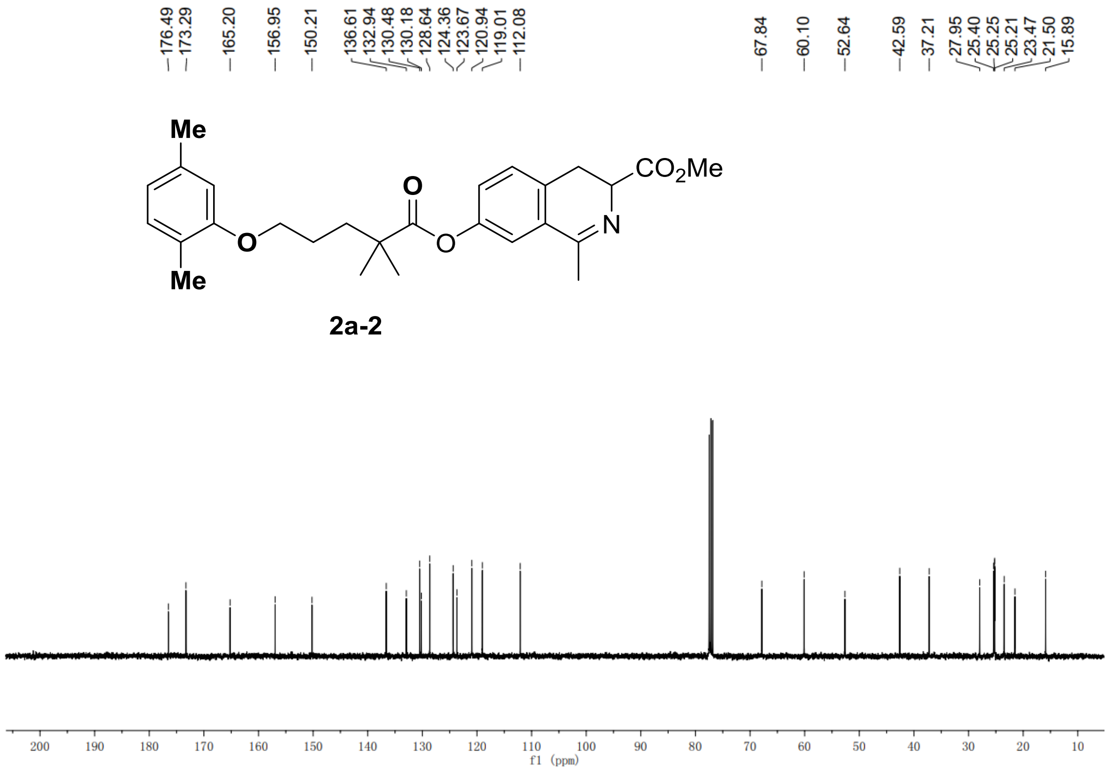


$^{1}$H-NMR and $^{13}$C-NMR of **2a-2**

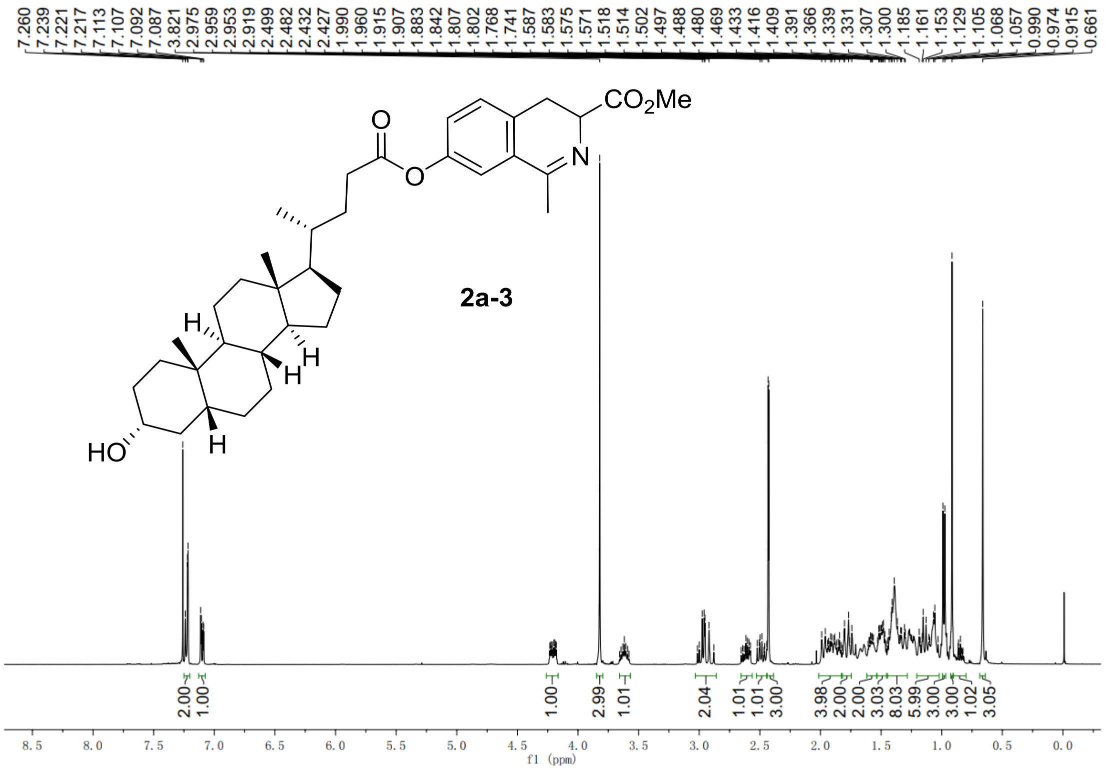

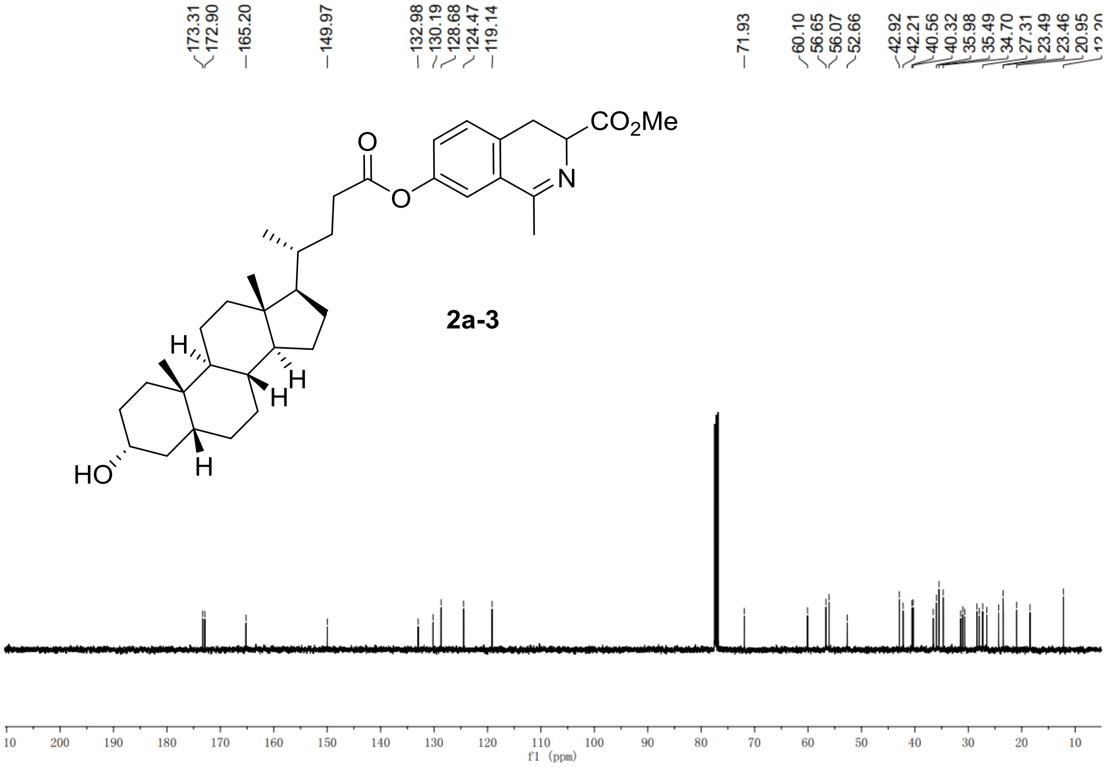


$^{1}$H-NMR and $^{13}$C-NMR of **2a-3**

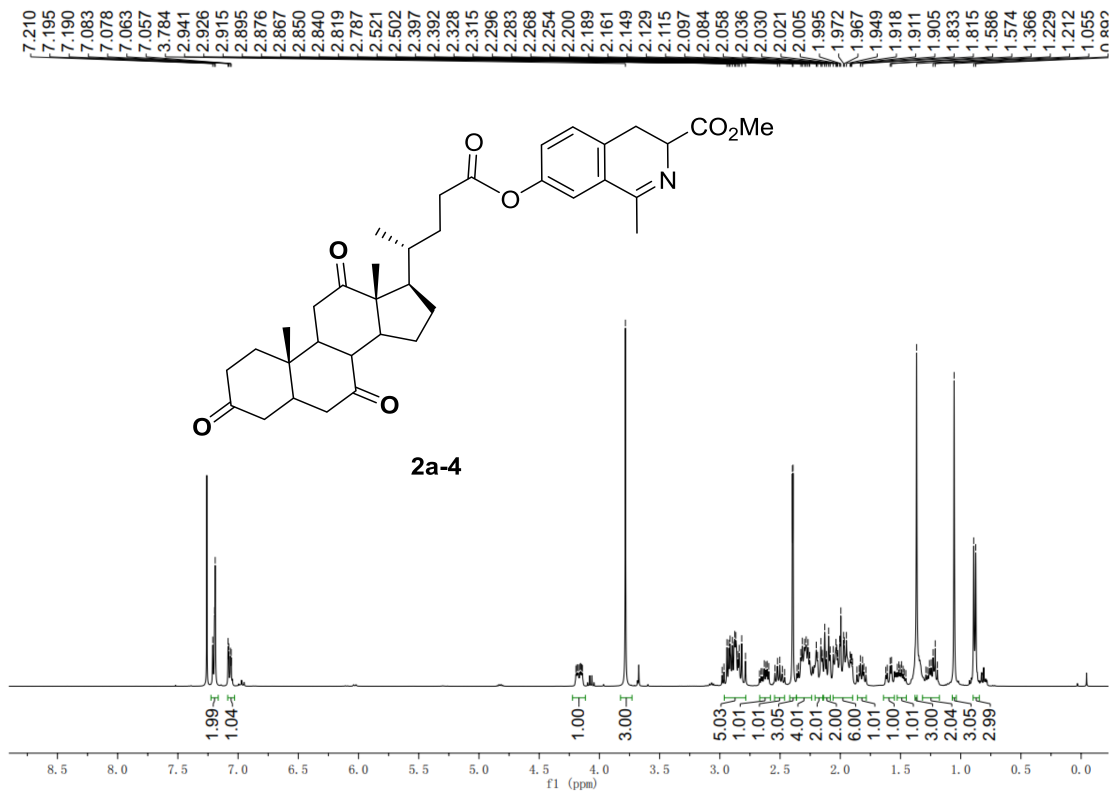

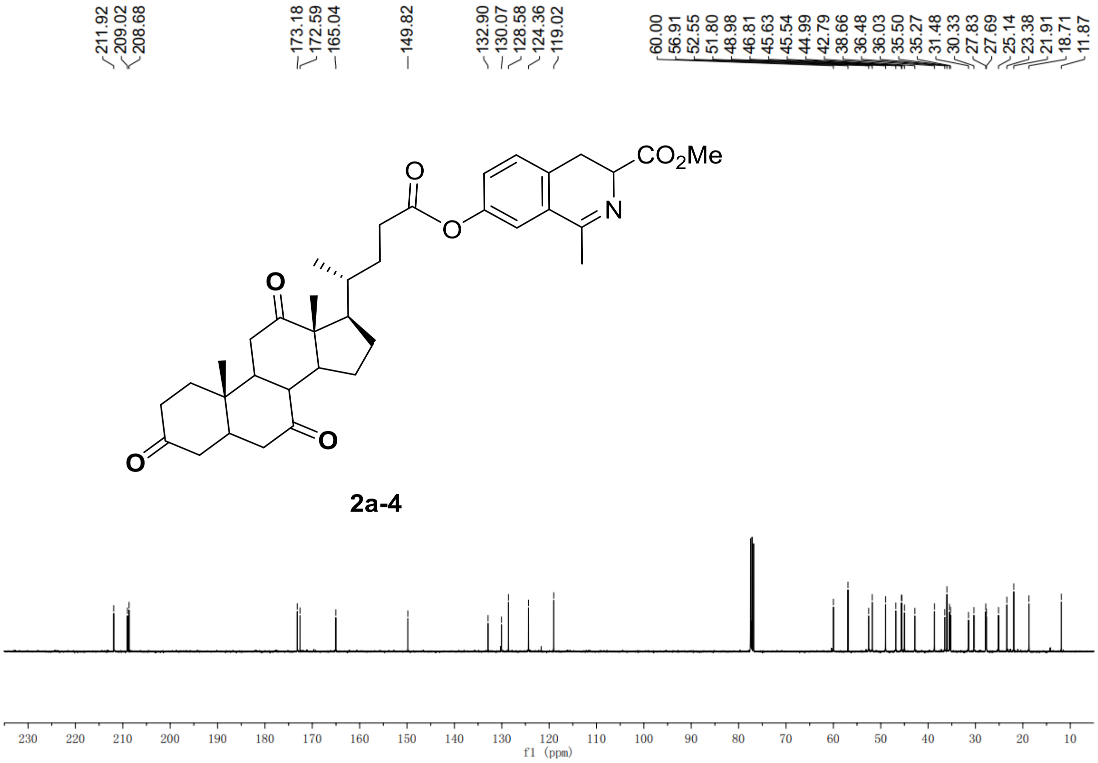


**2a-4**

$^{1}$H-NMR and $^{13}$C-NMR of **2a-4**

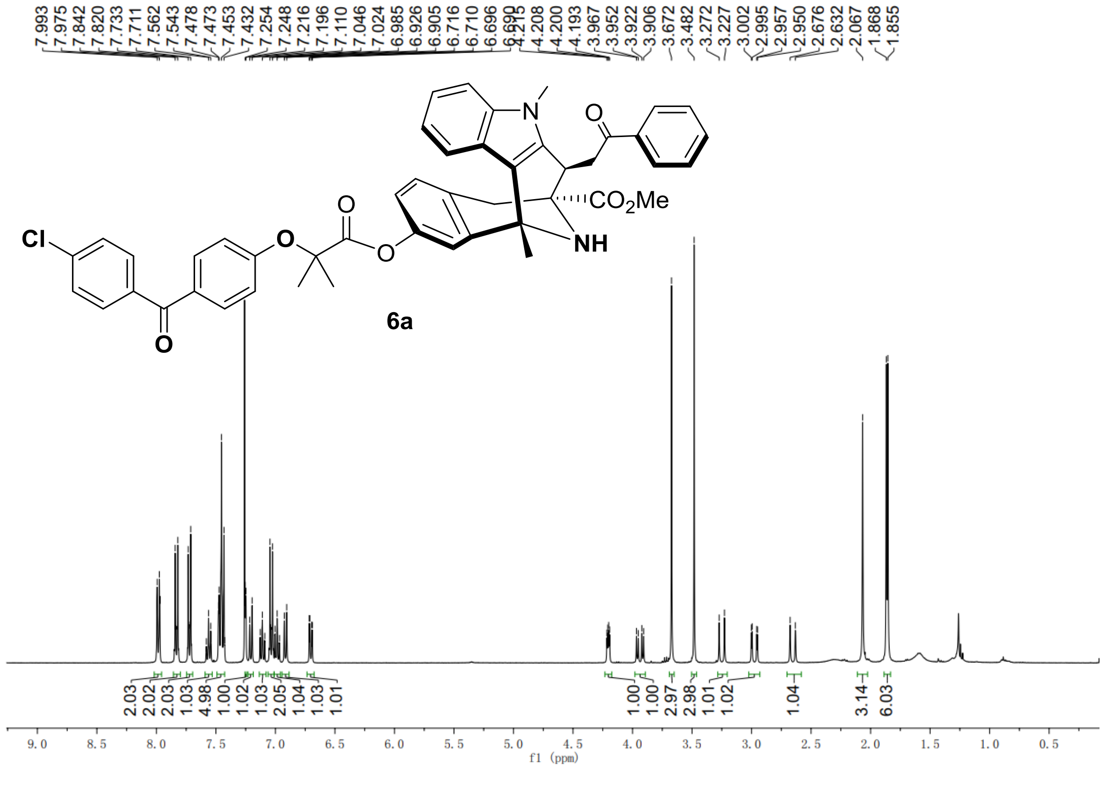


**6a**

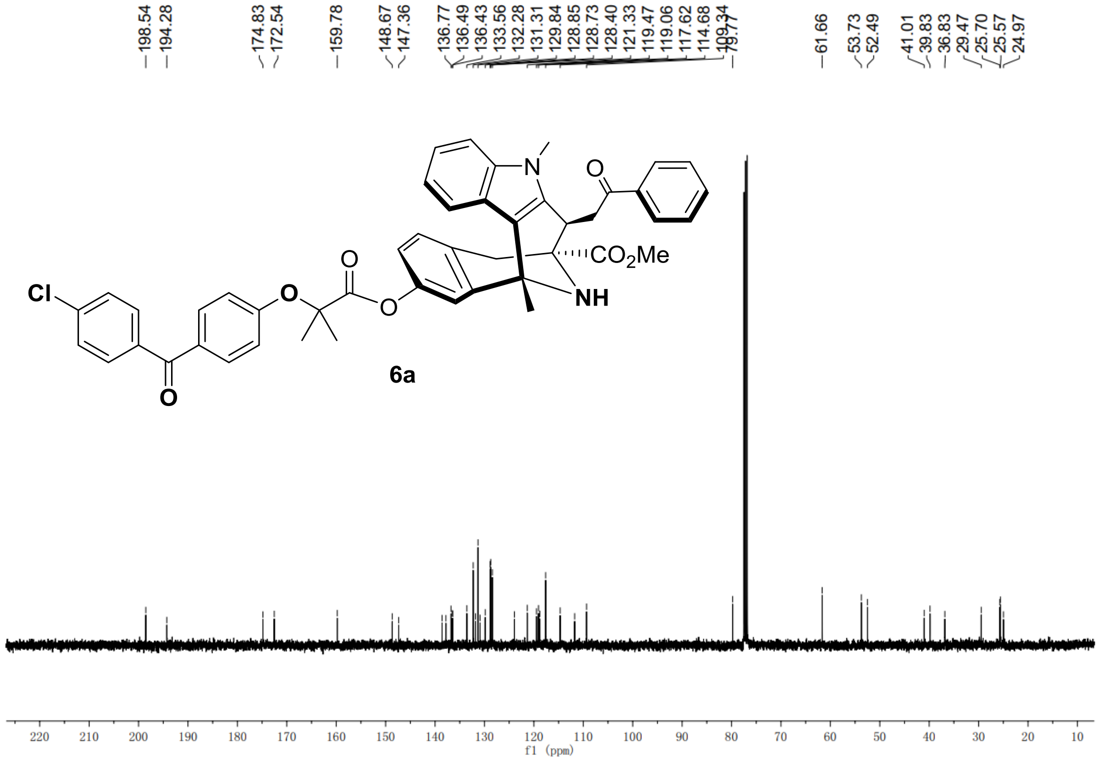


$^{1}$H-NMR and $^{13}$C-NMR of **6a**

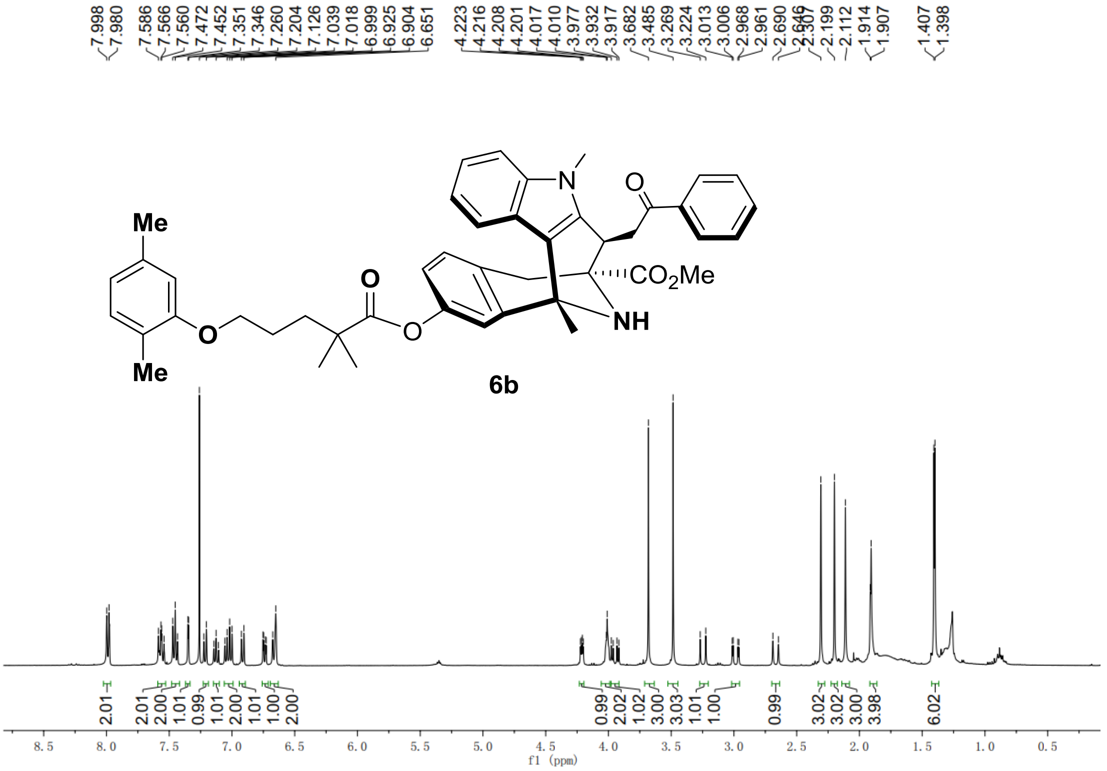

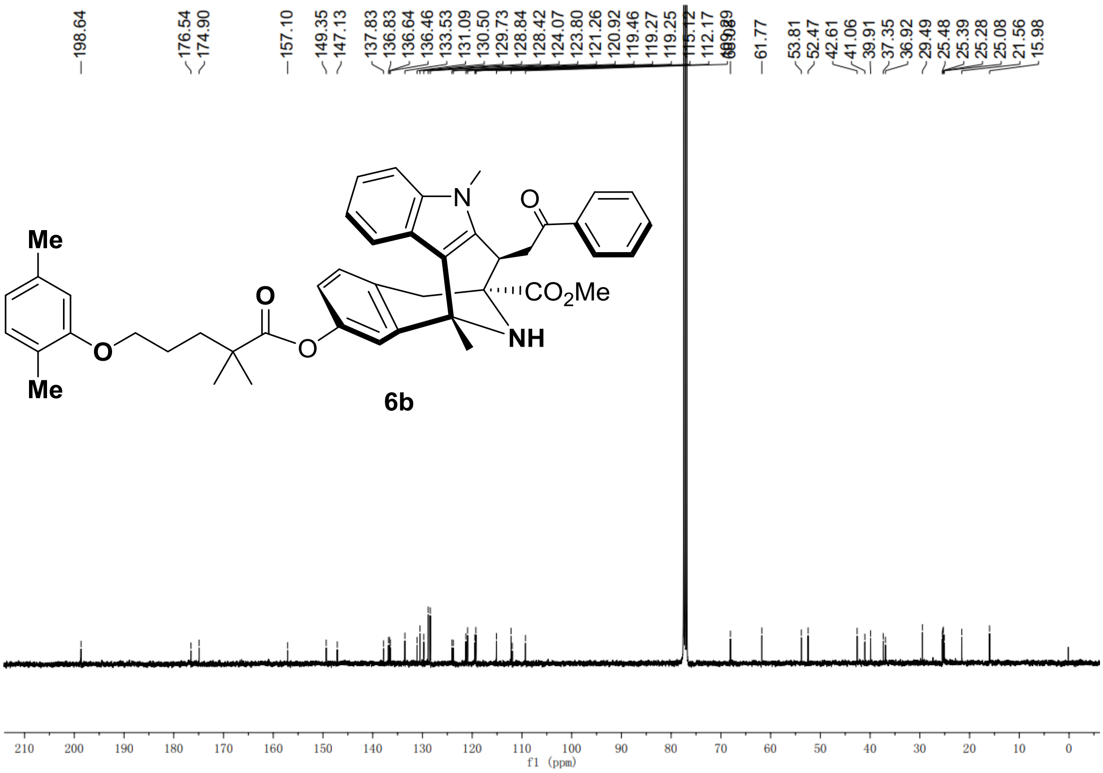


$^{1}$H-NMR and $^{13}$C-NMR of **6b**

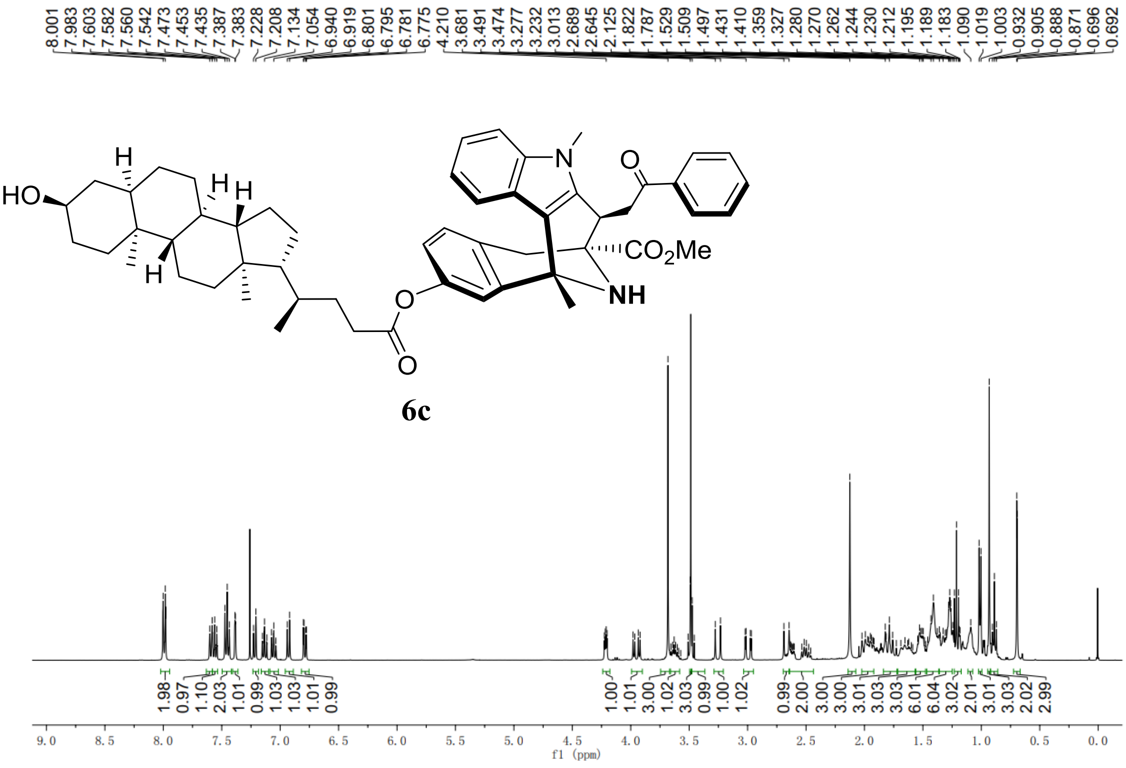

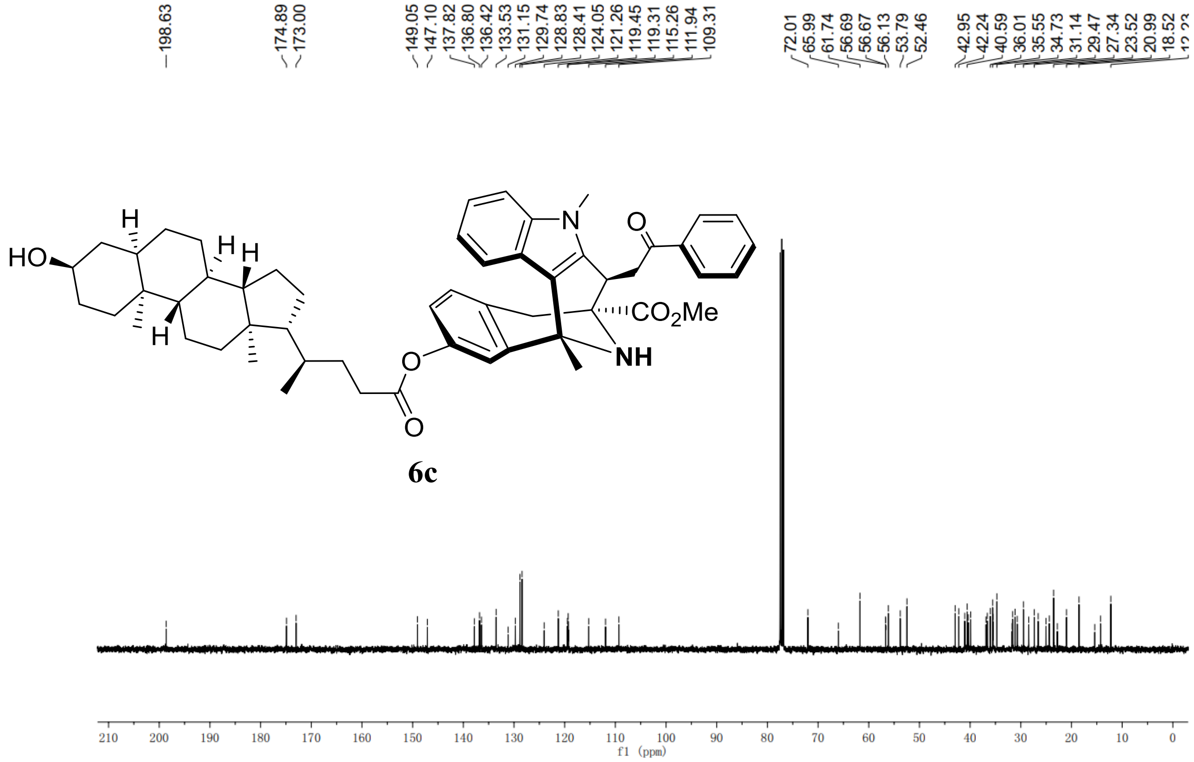


$^{1}$H-NMR and $^{13}$C-NMR of **6c**

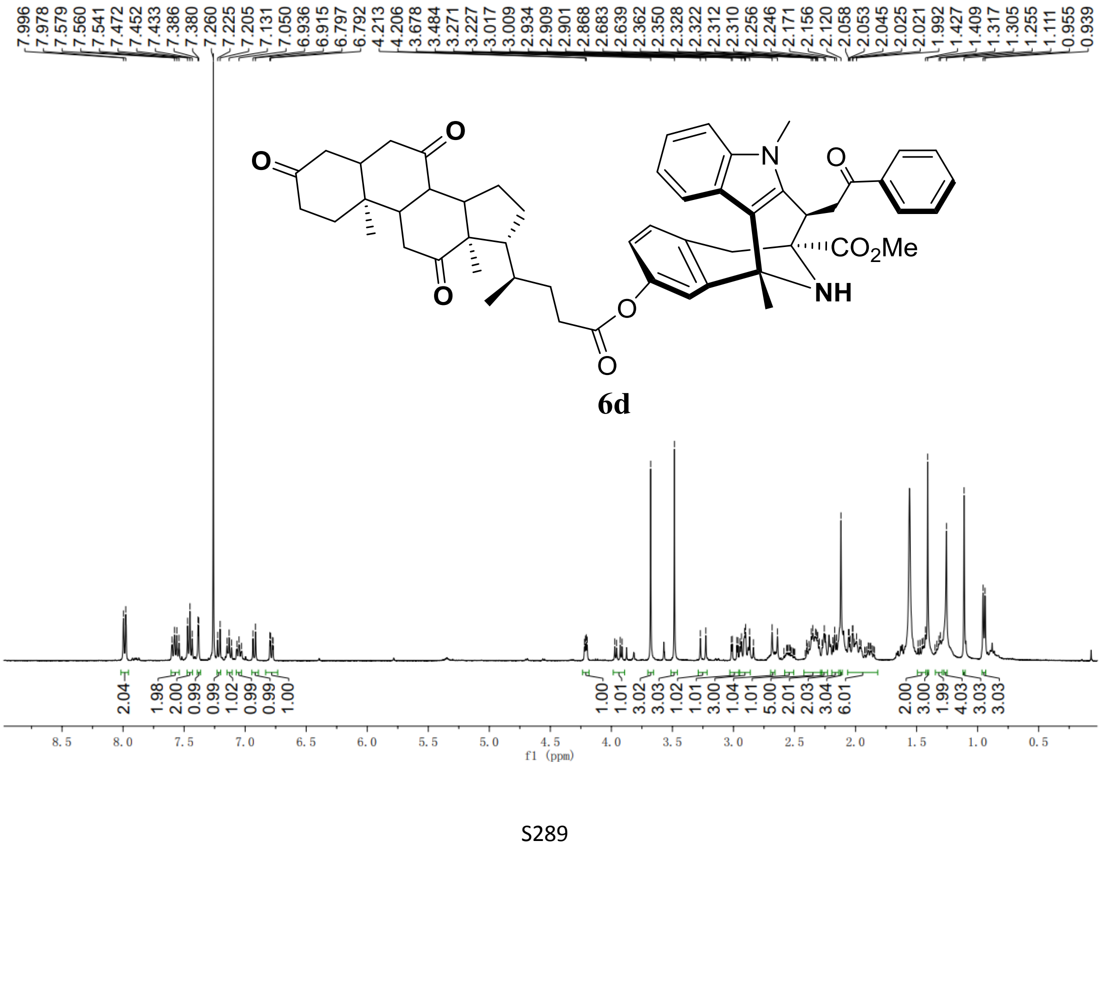

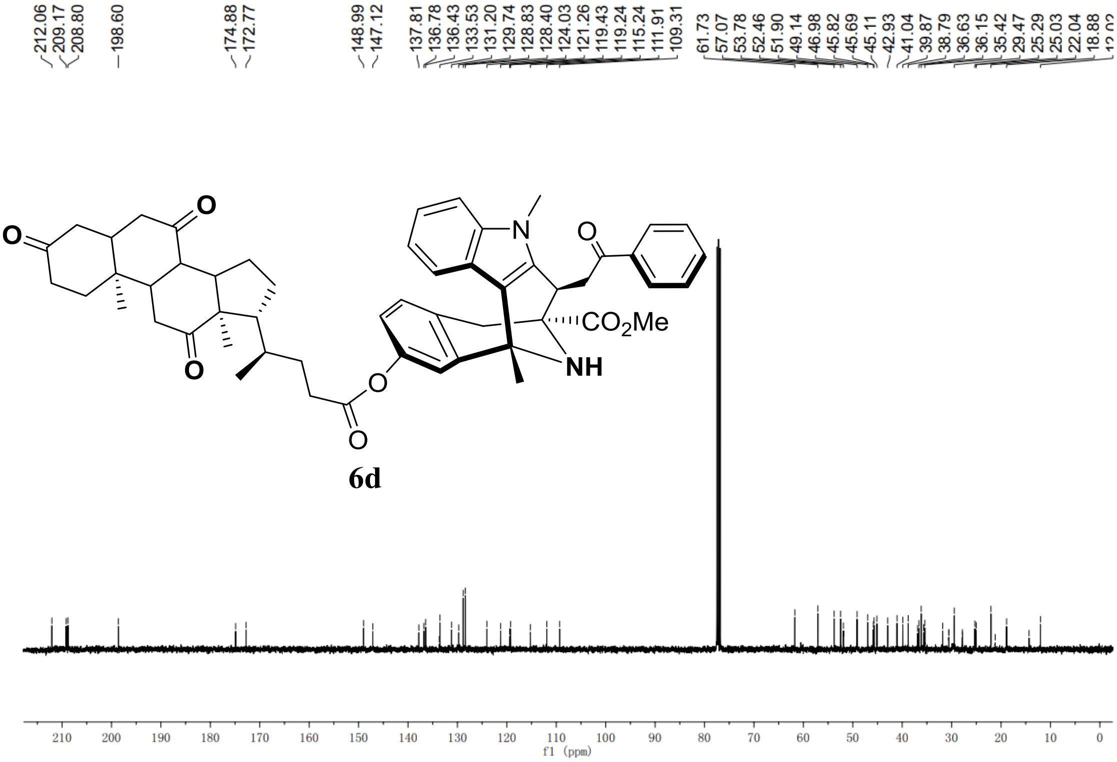


$^{1}$H-NMR and $^{13}$C-NMR of **6d**

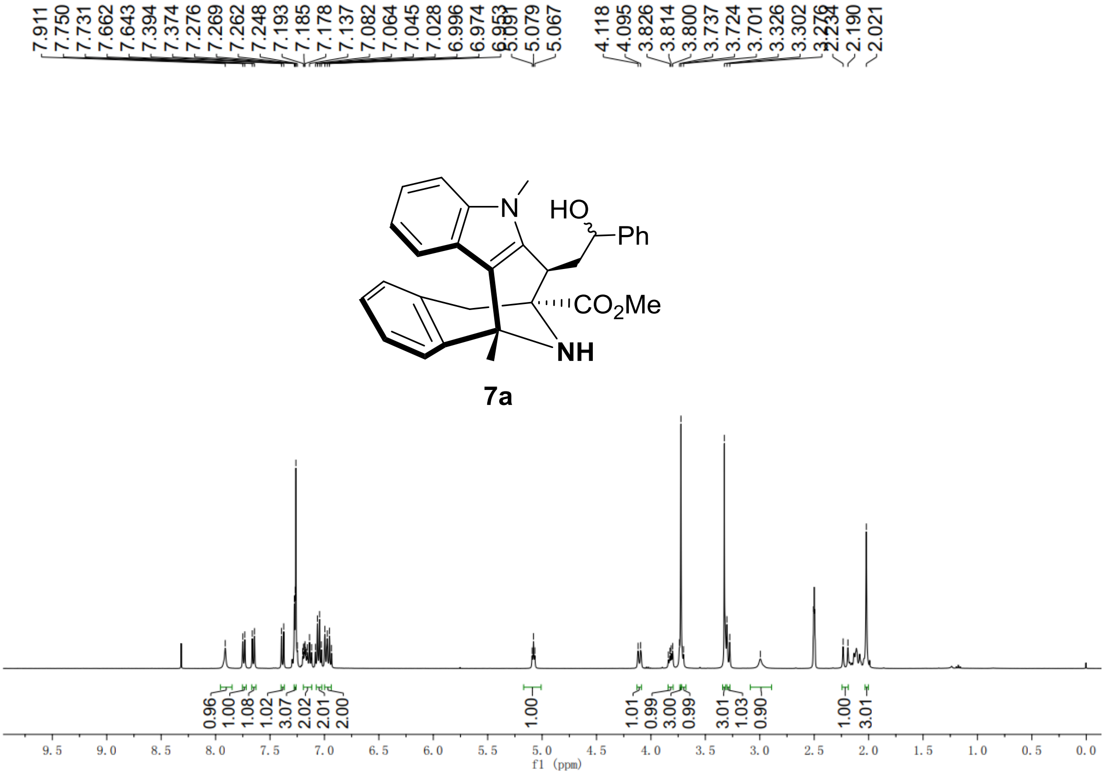

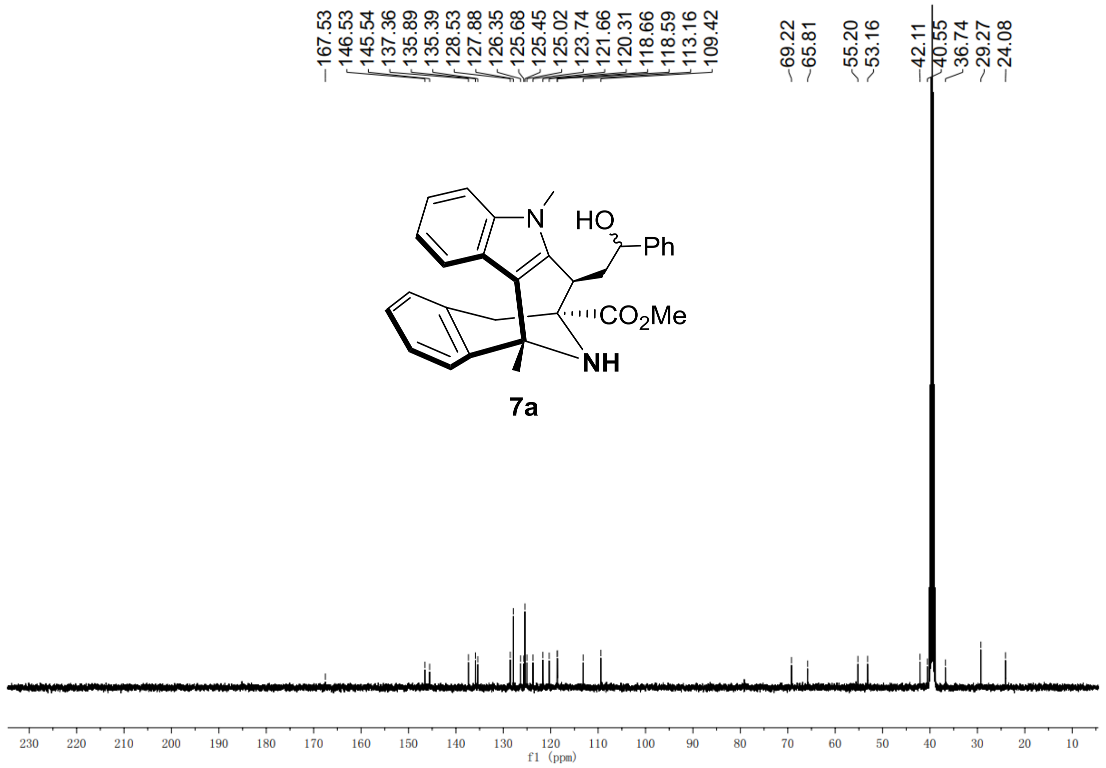


$^1$H-NMR and $^{13}$C-NMR of **7a**

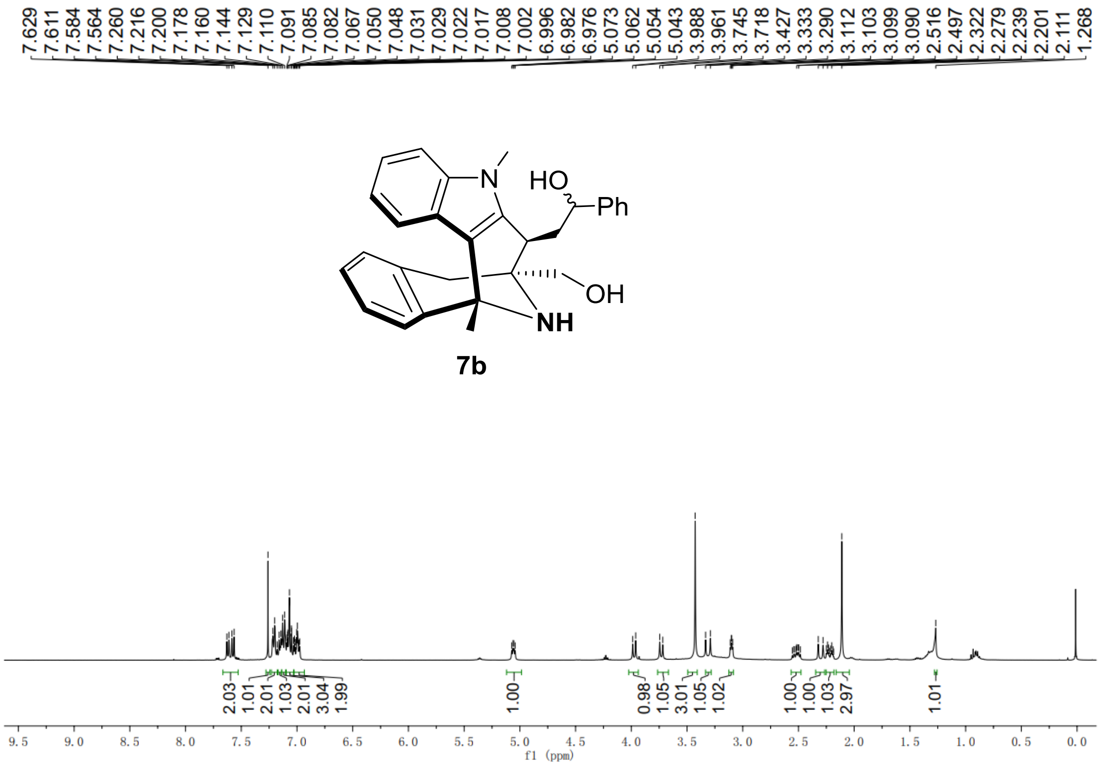

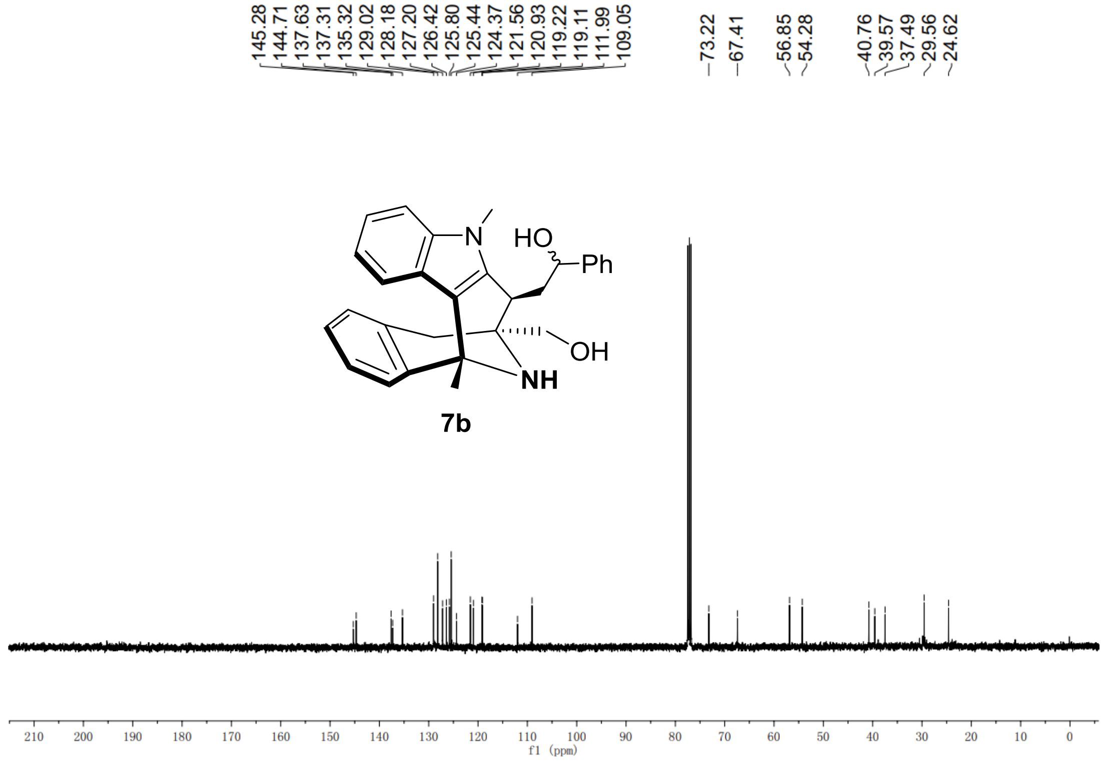


$^1$H-NMR and $^{13}$C-NMR of **7b**

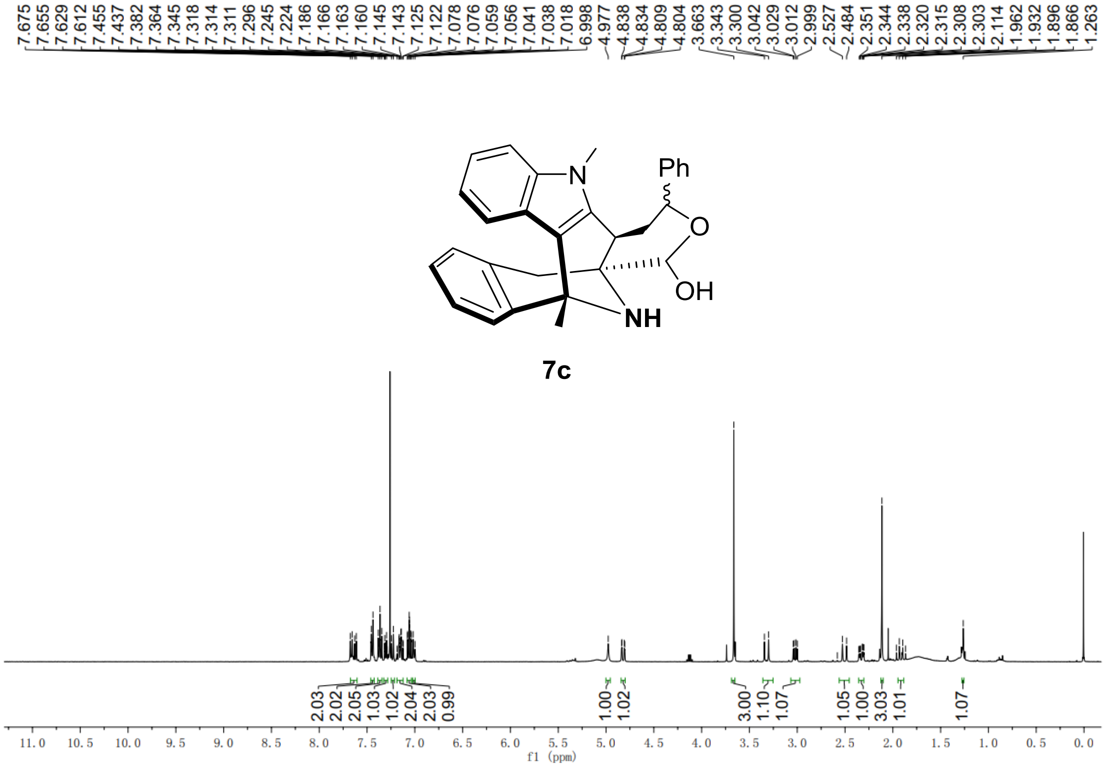

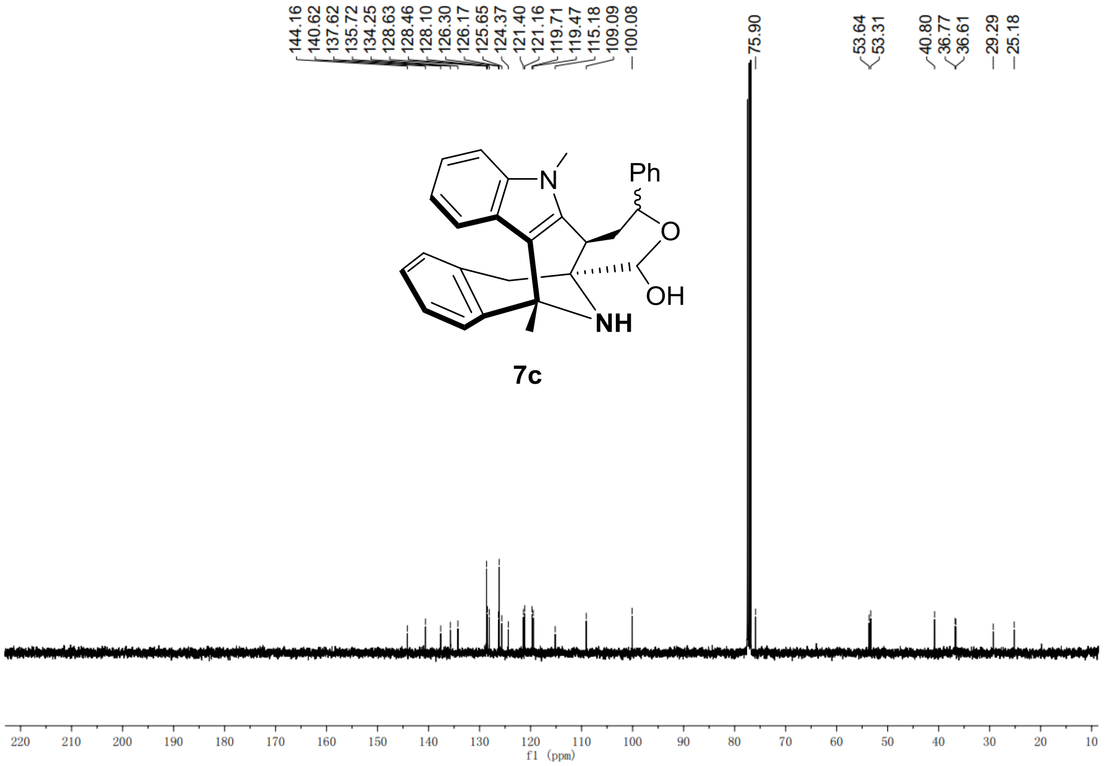


$^{1}$H-NMR and $^{13}$C-NMR of **7c**

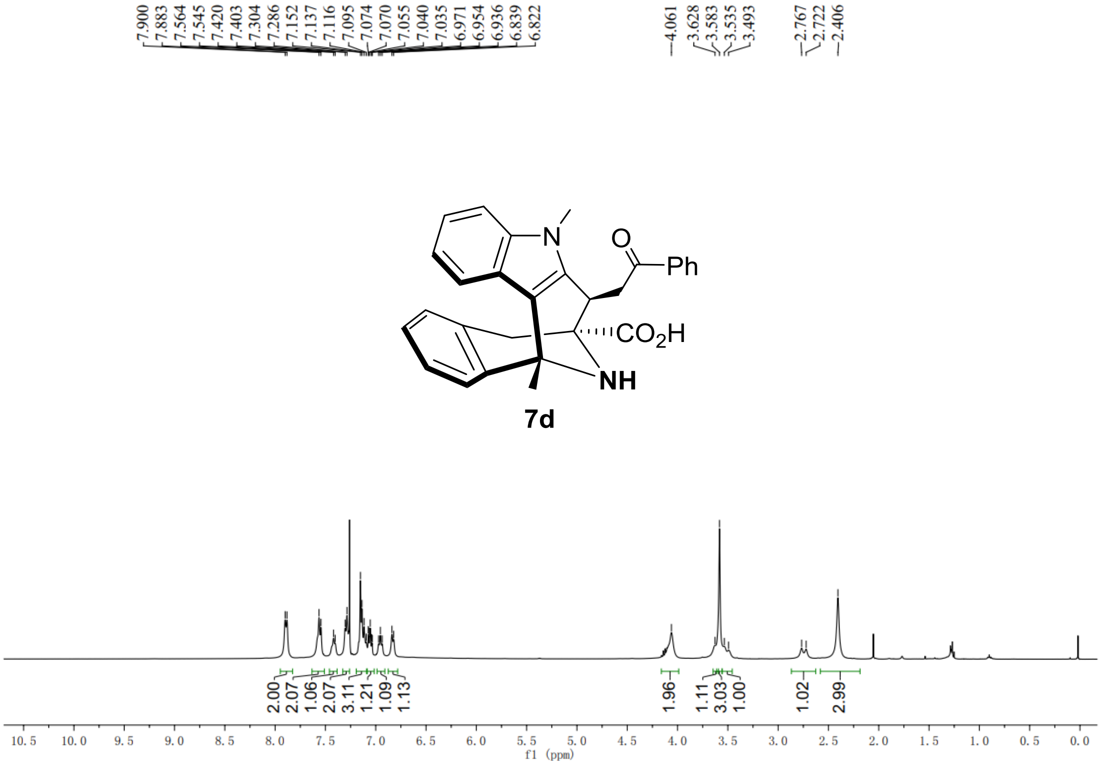

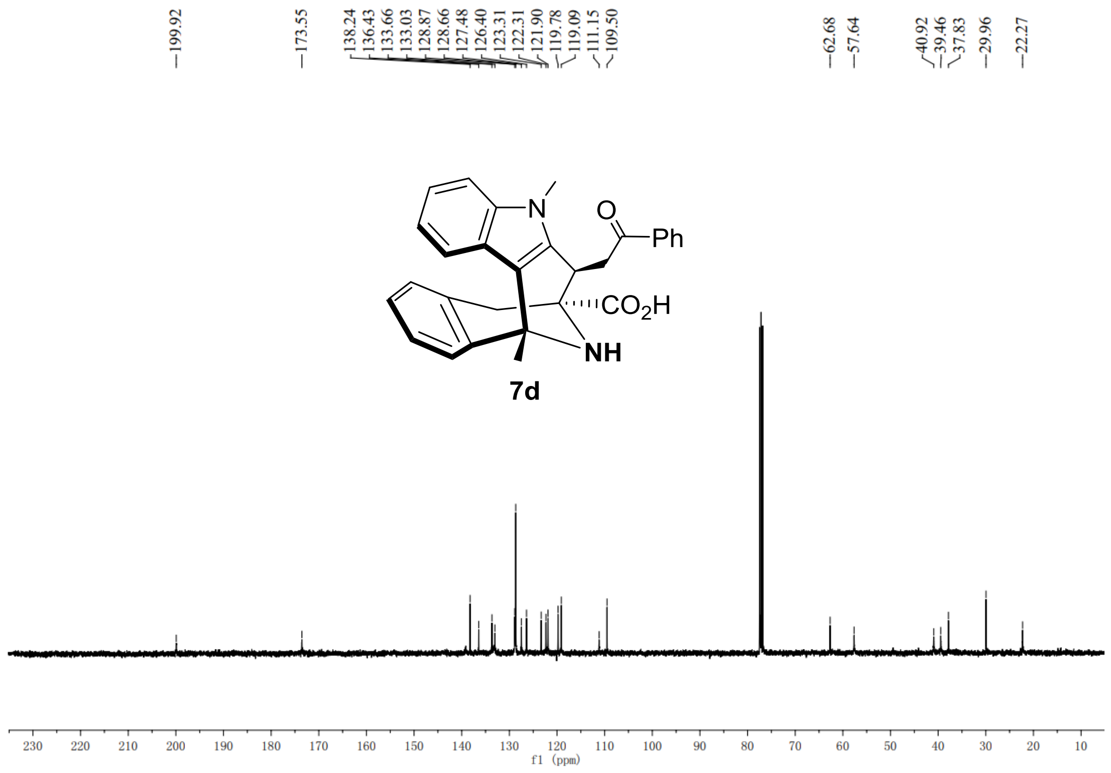

$^{1}$H-NMR and $^{13}$C-NMR of **7d**

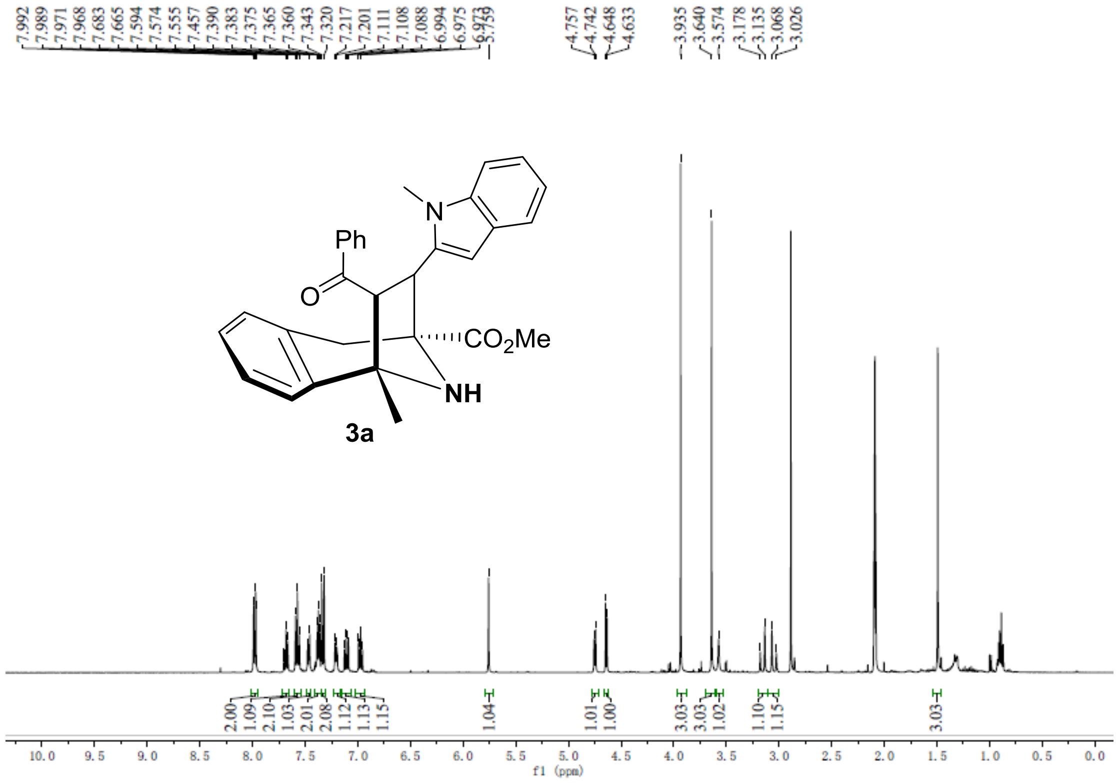

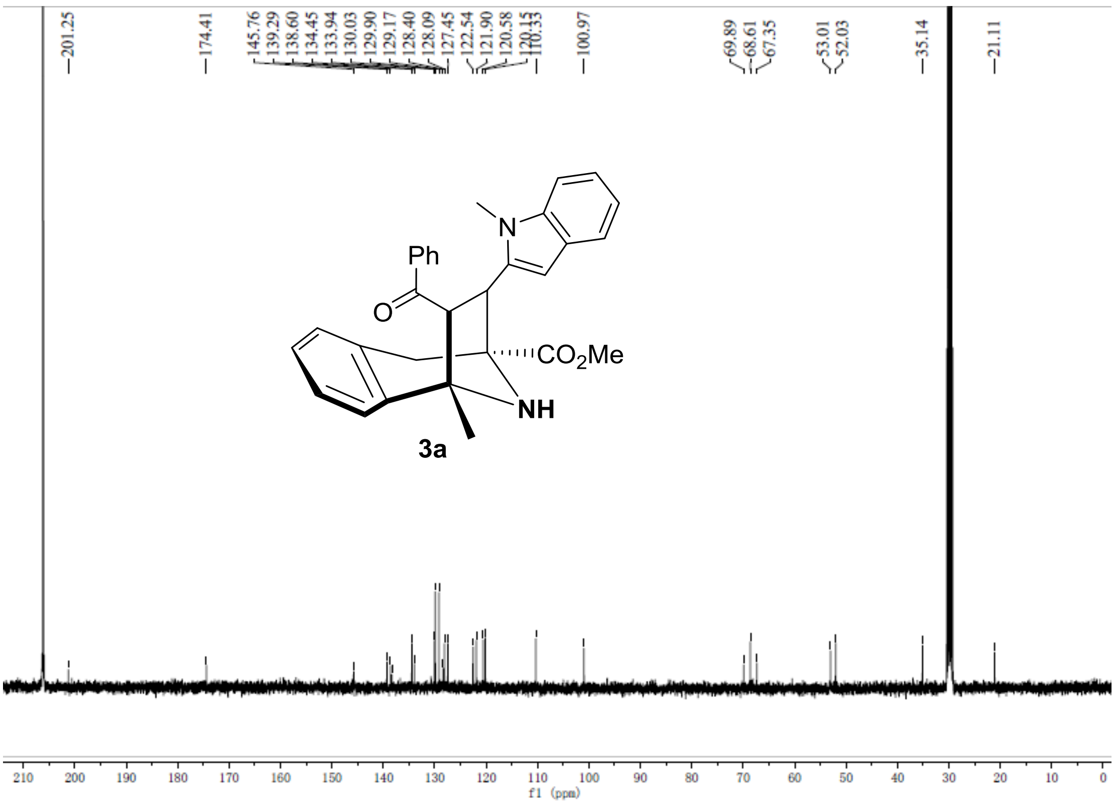


$^{1}$H-NMR and $^{13}$C-NMR of **3a**

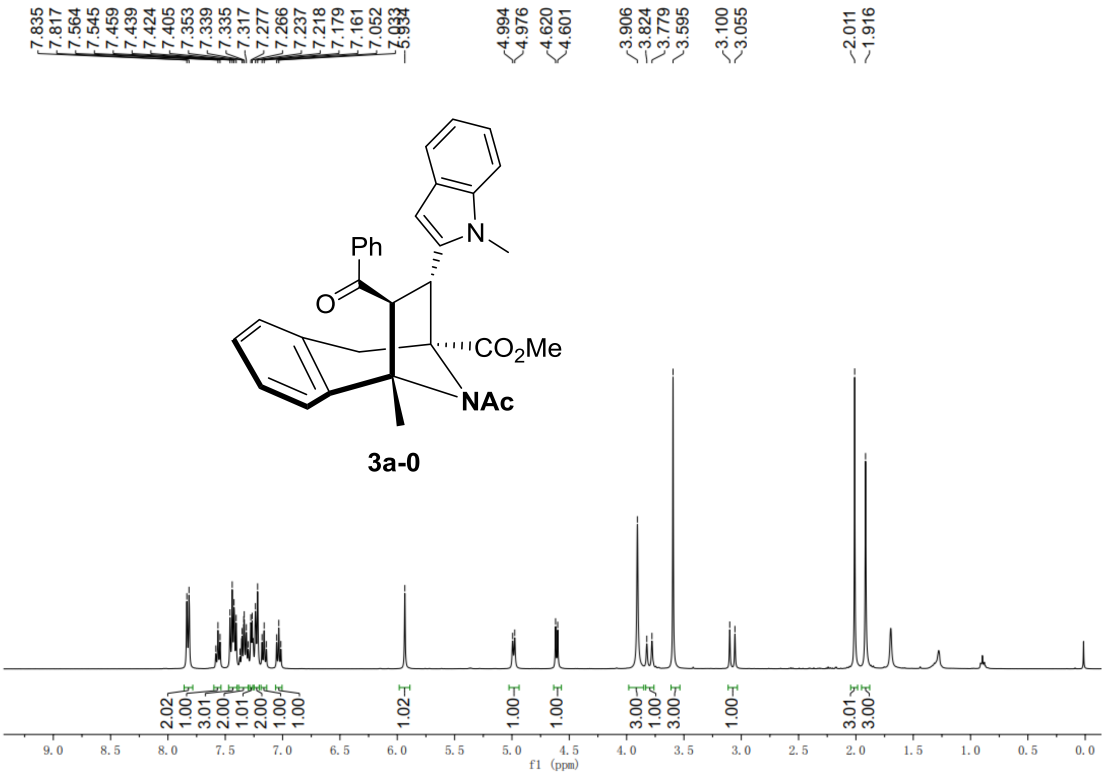

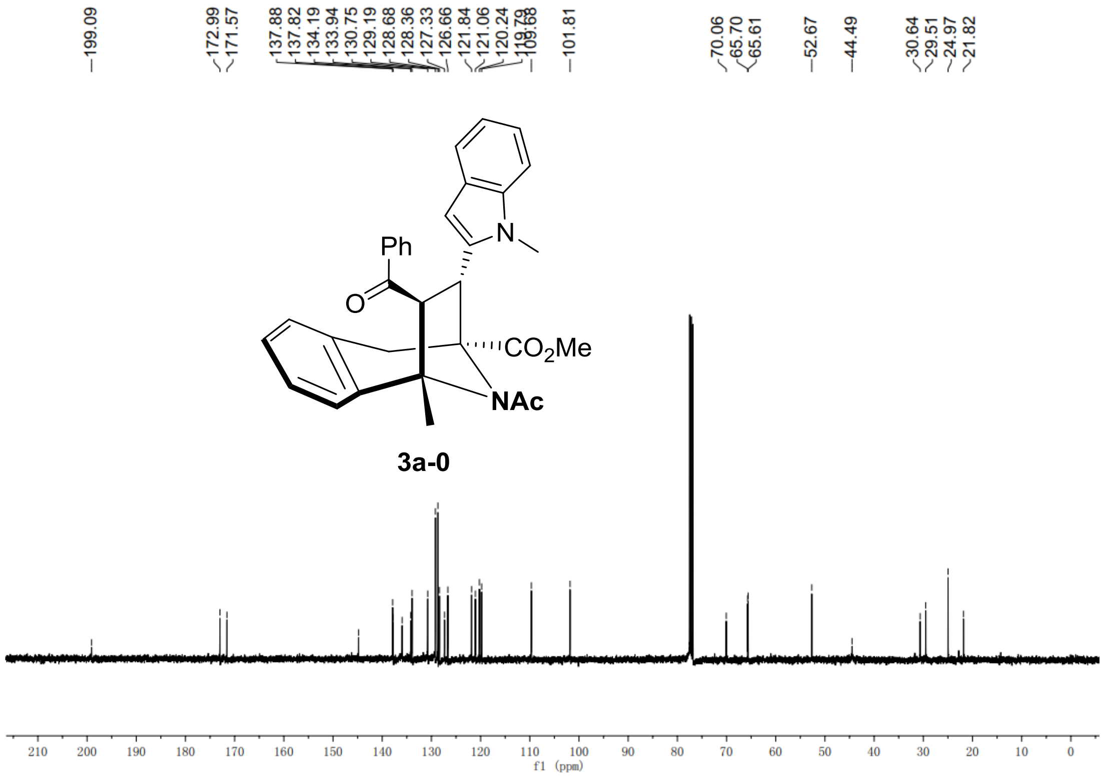


$^{1}$H-NMR and $^{13}$C-NMR of **3a-0**

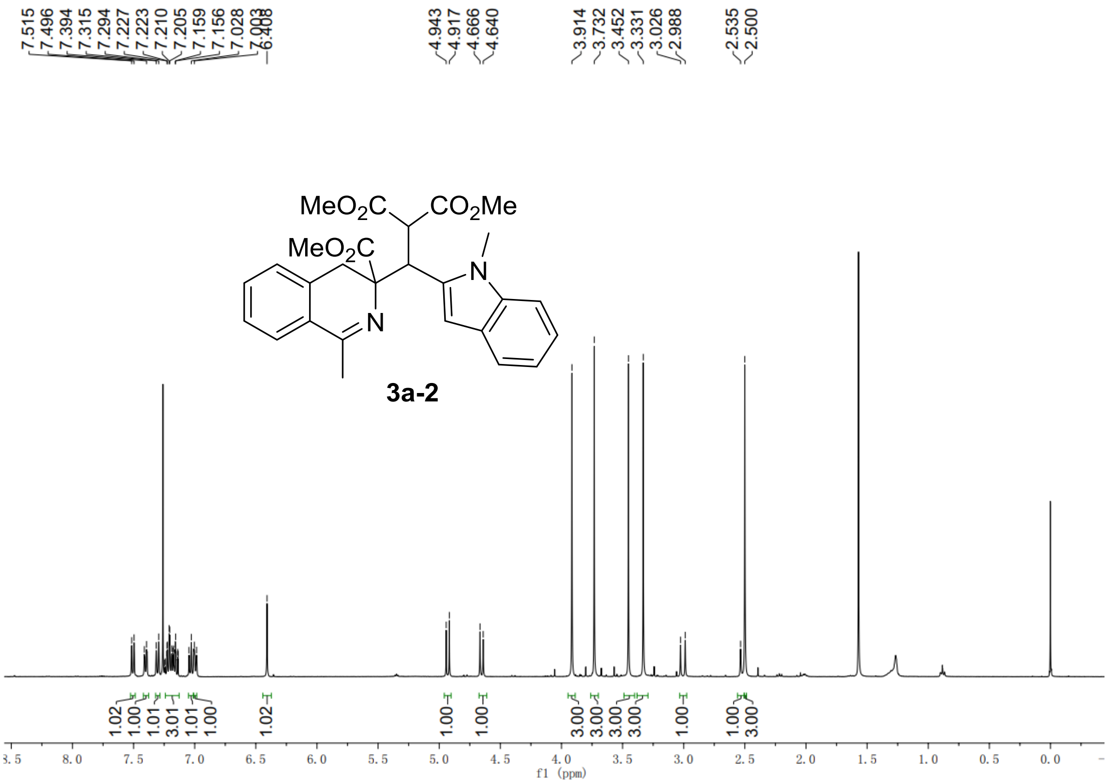

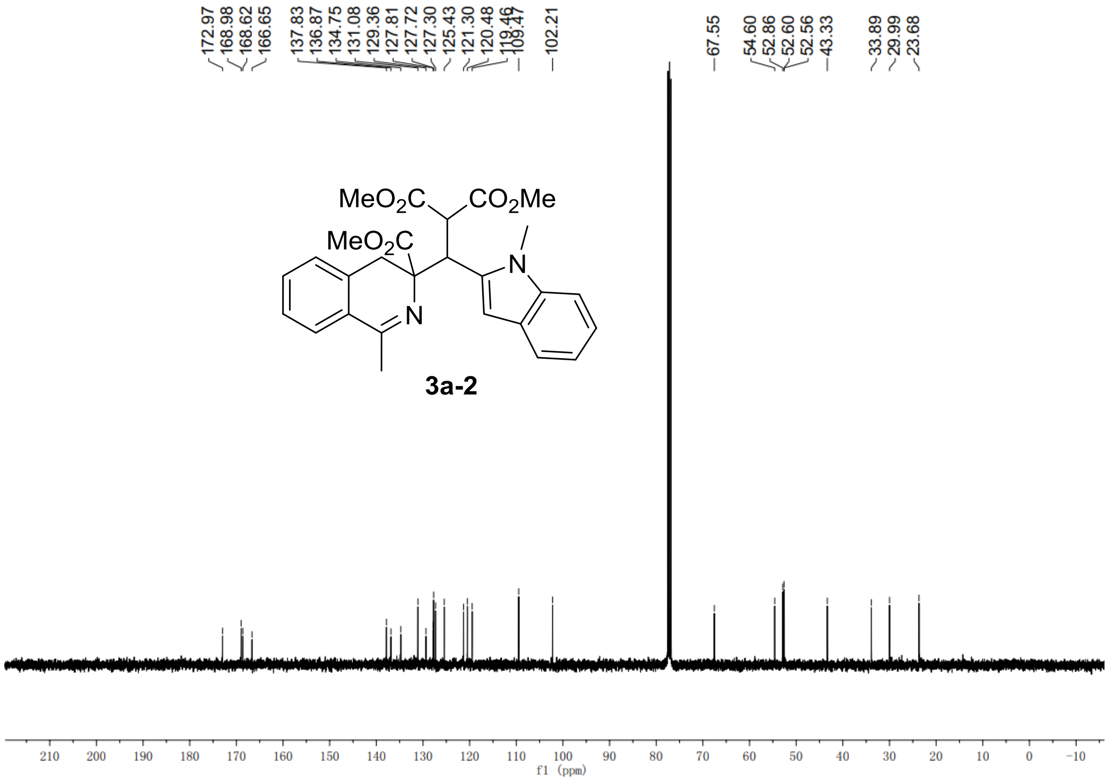


$^{1}$H-NMR and $^{13}$C-NMR of **3a-2**

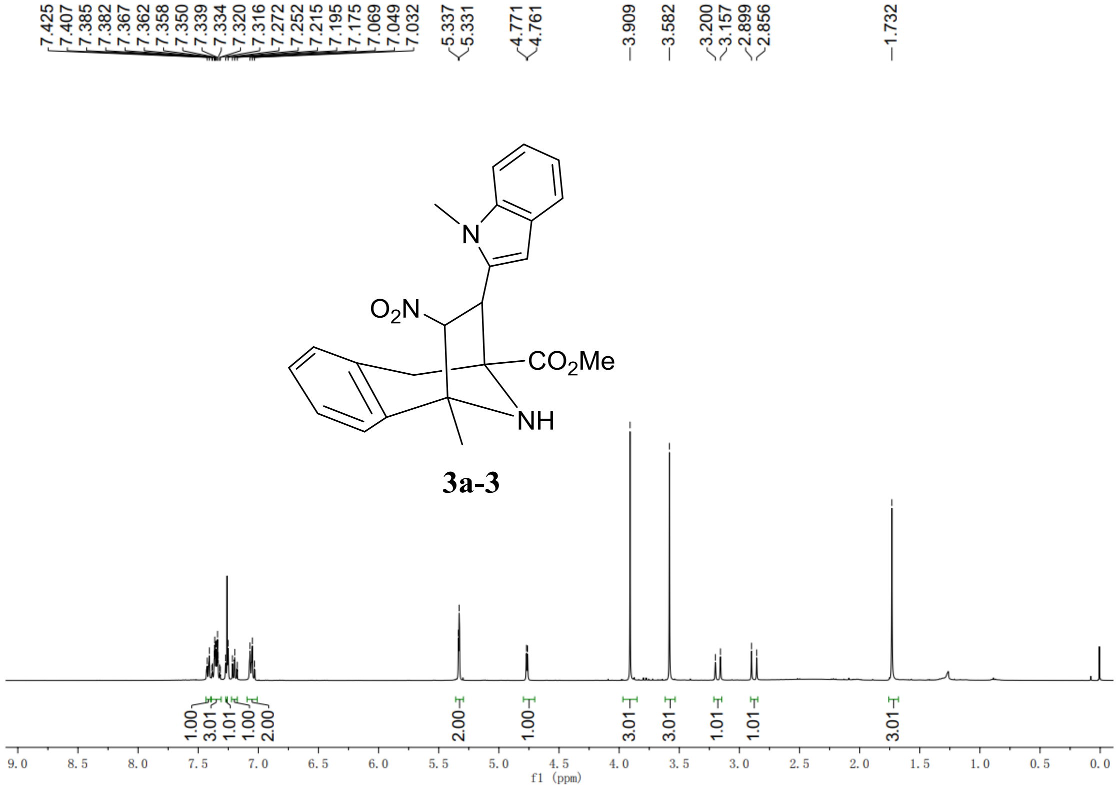

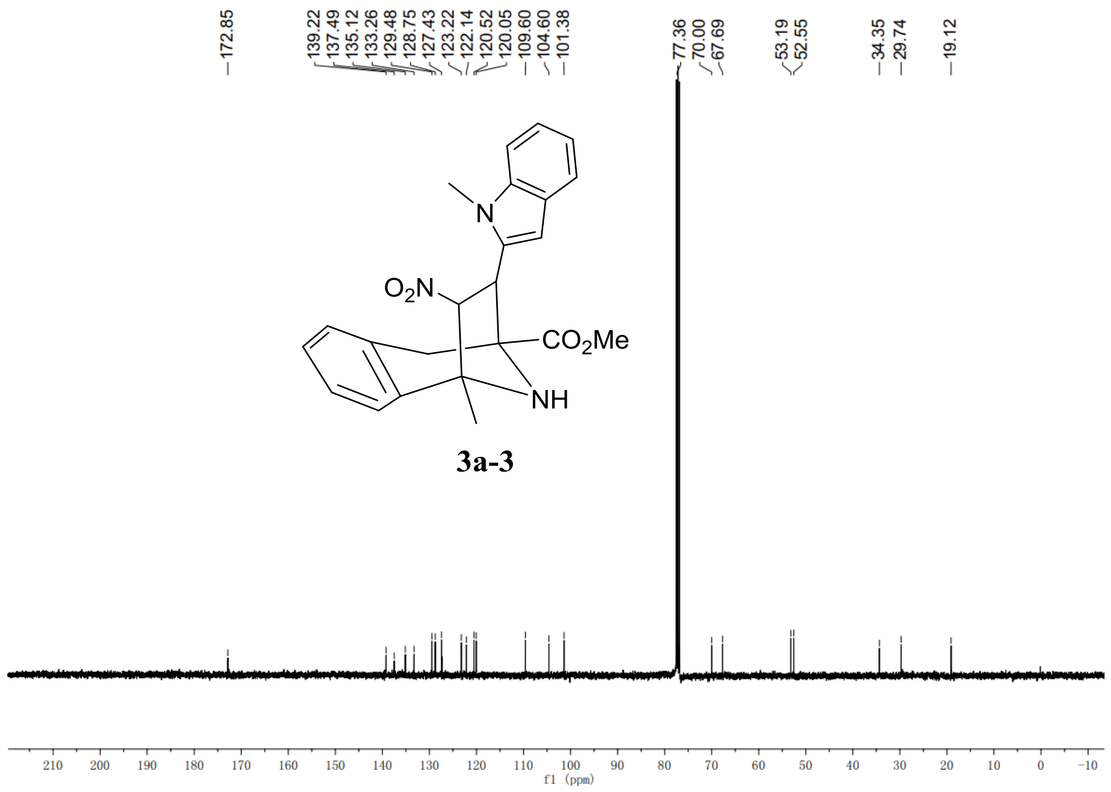

$^1$H-NMR and $^{13}$C-NMR of **3a-3**

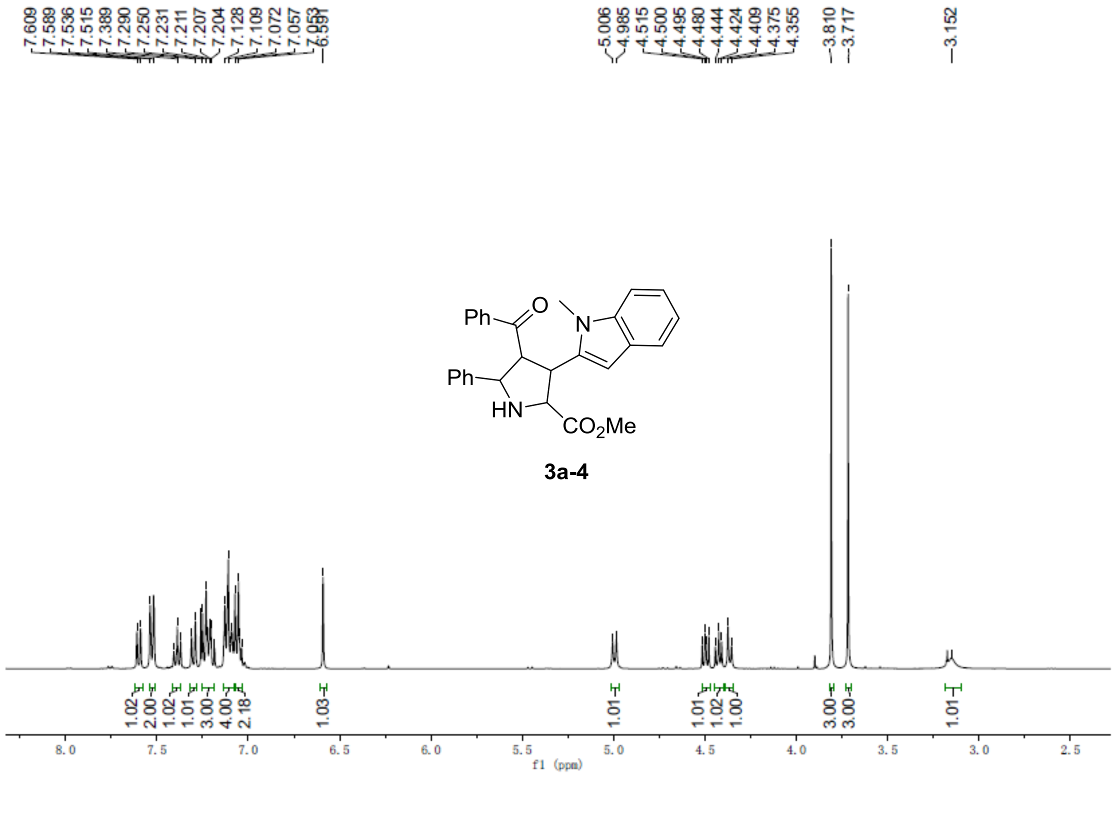

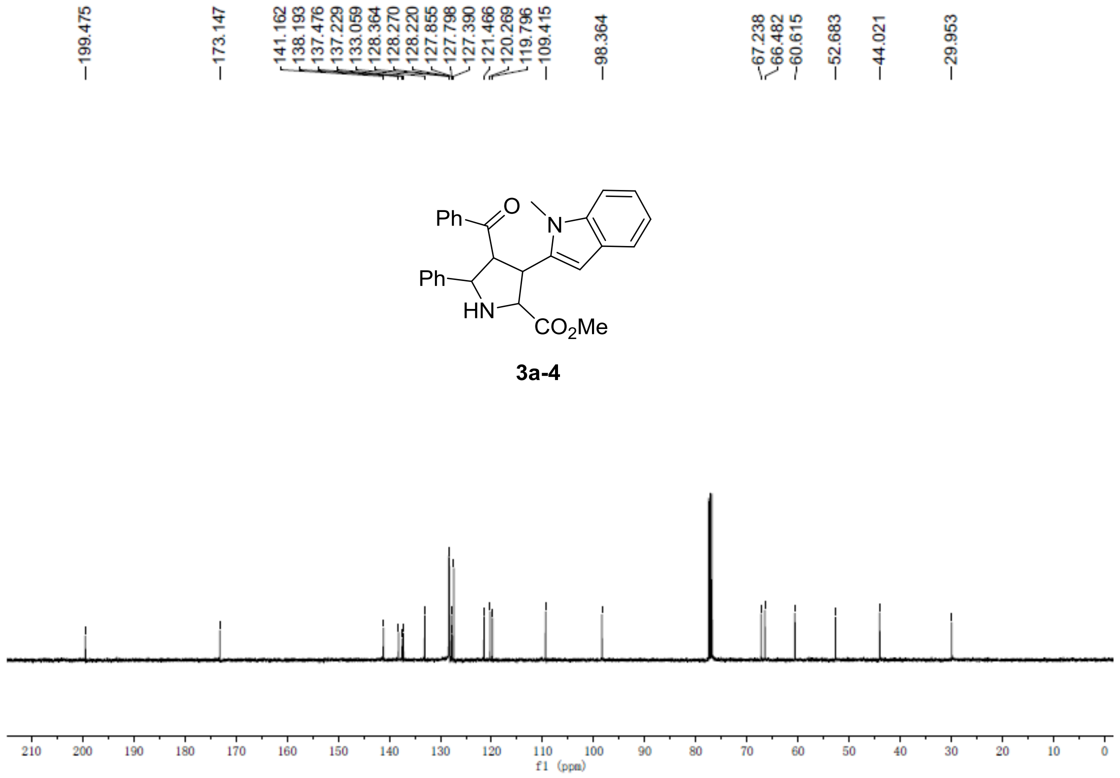


$^1$H-NMR and $^{13}$C-NMR of **3a-4**

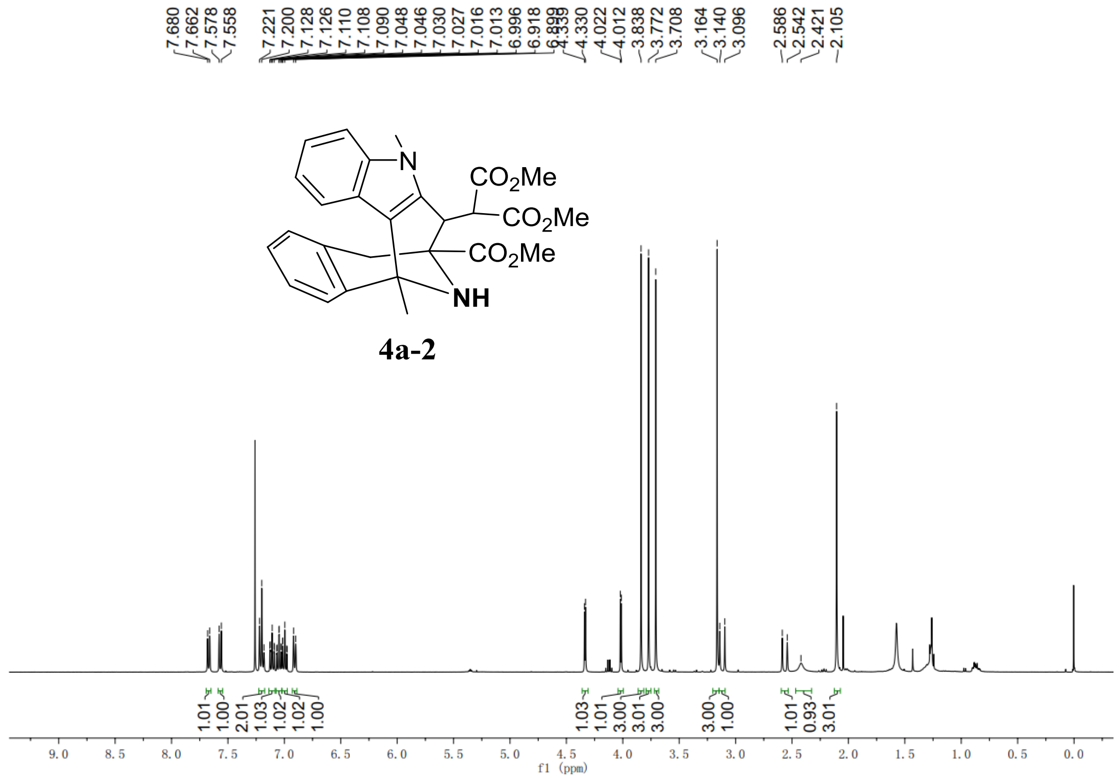

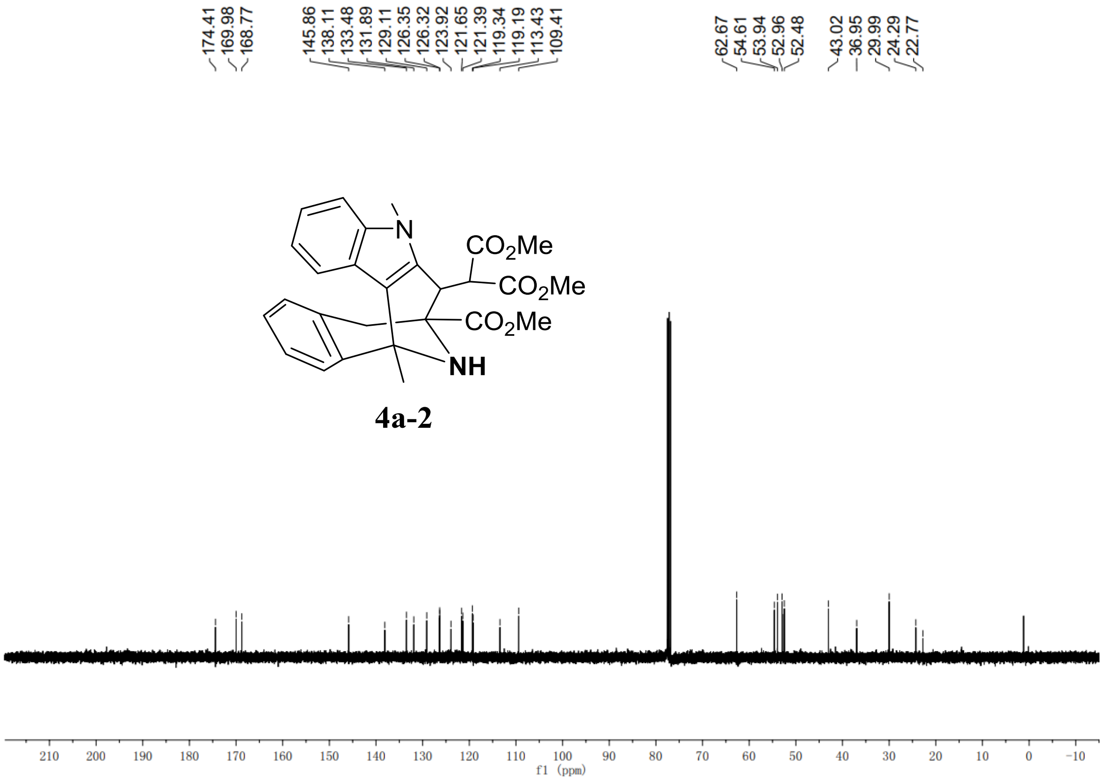


$^{1}$H-NMR and $^{13}$C-NMR of **4a-2**

## Supplementary References


[1] Rajasekar, S; Anbarasan, P. Tandem Rh(II) and Chiral Squaramide Relay Catalysis: Enantioselective Synthesis of Dihydro-β-carbolines via Insertion to C–H Bond and Aza-Michael Reaction. *Org. Lett.* **2019**, *21*, 3067.

[2] Tan., J.-P; Li, X;. Chen, Y; Rong, X; Zhu, L; Jiang, C; Xiao, K;. Wang, T. Highly Stereoselective Construction of Polycyclic Benzofused Tropane Scaffolds and Their Latent Bioactivities: Bifunctional Phosphonium Salt-enabled Cyclodearomatization Process. *Sci. China. Chem*, **2020**, *63*, 1091.

[3] a) Pan, J; Wu, J.-H; Zhang, H; Ren, X; Tan, J.-P; Zhu, L;. Zhang, H.-S; Jiang, C; Wang, T. Highly Enantioselective Synthesis of Fused Tri- and Tetrasubstituted Aziridines: aza-Darzens Reaction of Cyclic Imines with α-halogenated Ketones Catalyzed by Bifunctional Phosphonium Salt. *Angew. Chem., Int. Ed.*, **201**9, 58, 7425. b) Tan, J.-P; Yu, P; Wu, J.-H; Chen, Y; Pan, J; Jiang, C; Ren, X; Zhang, H.-S; Wang, T. Bifunctional Phosphonium Salt Directed Enantioselective Formal [4+ 1] Annulation of Hydroxyl-Substituted Quinone Methides with α-halogenated Ketones. *Org. Lett.*, **2019**, 21, 7298. c) Zhu, L; Ren, X; Du, J; Wu, J.-H; Tan,

J.-P; Che, J; Pan, J; Wang, T. Transition metal-free multicomponent reaction towards constructing chiral 2H-1,4-benzoxazine scaffolds. *Green Chem*. **2020**, 22, 7506.

[4]. Gaussian 16, Revision A.03, M. J. Frisch, G. W. Trucks, H. B. Schlegel, G. E. Scuseria, M. A. Robb, J. R. Cheeseman, G. Scalmani, V. Barone, G. A. Petersson, H. Nakatsuji, X. Li, M. Caricato, A. V. Marenich, J. Bloino, B. G. Janesko, R. Gomperts, B. Mennucci, H. P. Hratchian, J. V. Ortiz, A. F. Izmaylov, J. L. Sonnenberg, D. Williams-Young, F. Ding, F. Lipparini, F. Egidi, J. Goings, B. Peng, A. Petrone, T. Henderson, D. Ranasinghe, V. G. Zakrzewski, J. Gao, N. Rega, G. Zheng, W. Liang, M. Hada, M. Ehara, K. Toyota, R. Fukuda, J. Hasegawa, M. Ishida, T. Nakajima, Y. Honda, O. Kitao, H. Nakai, T. Vreven, K. Throssell, J. A. Montgomery, Jr., J. E. Peralta, F. Ogliaro, M. J. Bearpark, J. J. Heyd, E. N. Brothers, K. N. Kudin, V. N. Staroverov, T. A. Keith, R. Kobayashi, J. Normand, K. Raghavachari, A. P. Rendell, J. C. Burant, S. S. Iyengar, J. Tomasi, M. Cossi, J. M. Millam, M. Klene, C. Adamo, R. Cammi, J. W. Ochterski, R. L. Martin, K. Morokuma, O. Farkas, J. B. Foresman, and D. J. Fox, Gaussian, Inc., Wallingford CT, 2016.

[5]. Marenich, A. V; Cramer, C. J; Truhlar, D. G. Universal Solvation Model Based on Solute Electron Density and on a Continuum Model of the Solvent Defined by the Bulk Dielectric Constant and Atomic Surface Tensions. *J. Phys. Chem. B* **2009**, *113*, 6378-6396

[6]. Becke, A. D. Density-functional thermochemistry. III. The role of exact exchange. *J. Chem. Phys*. **1993**, *98*, 5648-5652.

[7]. Zhao, Y; Truhlar, D. G. The M06 Suite of Density Functionals for Main Group Thermochemistry, Thermochemical Kinetics, Noncovalent Interactions, Excited States, and Transition Elements: Two New Functionals and Systematic Testing of Four M06-Class Functionals and 12 Other Functionals. *Theor. Chem. Acc*. **2008**, *120*, 215-241.

[8] (a)Neese, F. Software update: the ORCA program system, version 4.0. Wiley Interdiscip. Rev.: Comput. Mol. Sci. 2018, 8, e1327 (b)Andrae, D.; Haeussermann, U.; Dolg, M.; Stoll, H.; Preuss, H. Energy-adjustedab initio pseudopotentials for the second and third row transition elements. *Theor. Chim. Acta* **1990**, *77*, 123. (c) Riplinger, C., Sandhoefer, B., Hansen, A.; Neese, F. Natural triple excitations in local coupled cluster calculations with pair natural orbitals. *J. Chem. Phys*. **2013**, *139*, 134101. (d) Weigend, F.; Ahlrichs, R. Balanced basis sets of split valence, triple zeta valence and quadruple zeta valence quality for H to Rn: Design and assessment of accuracy. *Phys. Chem. Chem. Phys*. **2005**, *7*, 3297.

[9]. Legault, C. Y. CYLview, 1.0 b, Université de Sherbrooke, Quebec (Canada), **2009**, (http://www.cylview.org).
