# Supplementary material for: Asymmetric synthesis of N-bridged [3.3.1] ring systems by phosphonium salt/Lewis acid relay catalysis
Source: Nat Commun. 2022 Jan 18;13:357. doi: 10.1038/s41467-022-28001-8 (PMC8766524; doi:10.1038/s41467-022-28001-8)
Supplement: Supplementary file 2 — Reporting Summary [file 41467_2022_28001_MOESM2_ESM.pdf]

nature portfolio

Corresponding author(s): Tianli Wang

Last updated by author(s): Nov 26, 2021

Reporting Summary

Nature Portfolio wishes to improve the reproducibility of the work that we publish. This form provides structure for consistency and transparency in reporting. For further information on Nature Portfolio policies, see our Editorial Policies and the Editorial Policy Checklist.

Statistics

For all statistical analyses, confirm that the following items are present in the figure legend, table legend, main text, or Methods section.

n/a	Confirmed	
☐	☒	The exact sample size (n) for each experimental group/condition, given as a discrete number and unit of measurement
☐	☒	A statement on whether measurements were taken from distinct samples or whether the same sample was measured repeatedly
☒	☐	The statistical test(s) used AND whether they are one- or two-sided<br>*Only common tests should be described solely by name; describe more complex techniques in the Methods section.*
☒	☐	A description of all covariates tested
☒	☐	A description of any assumptions or corrections, such as tests of normality and adjustment for multiple comparisons
☐	☒	A full description of the statistical parameters including central tendency (e.g. means) or other basic estimates (e.g. regression coefficient) AND variation (e.g. standard deviation) or associated estimates of uncertainty (e.g. confidence intervals)
☒	☐	For null hypothesis testing, the test statistic (e.g. F, t, r) with confidence intervals, effect sizes, degrees of freedom and P value noted<br>*Give P values as exact values whenever suitable.*
☒	☐	For Bayesian analysis, information on the choice of priors and Markov chain Monte Carlo settings
☒	☐	For hierarchical and complex designs, identification of the appropriate level for tests and full reporting of outcomes
☐	☒	Estimates of effect sizes (e.g. Cohen's d, Pearson's r), indicating how they were calculated

Our web collection on statistics for biologists contains articles on many of the points above.

Software and code

Policy information about availability of computer code

Data collection	Biotek Gen v5.2.0 was used to test cell survival viability. GaussView 6.0 Gaussian 16 Revision A.03 ORCA 4.2 CYLView 1.0 was used for the DFT study.
Data analysis	GraphPad Prism v8.4.3.was used to analyze mean, standard deviation and standard error of the mean and to draw curves in the graphs.

For manuscripts utilizing custom algorithms or software that are central to the research but not yet described in published literature, software must be made available to editors and reviewers. We strongly encourage code deposition in a community repository (e.g. GitHub). See the Nature Portfolio guidelines for submitting code & software for further information.

Data

Policy information about availability of data

All manuscripts must include a data availability statement. This statement should provide the following information, where applicable:

- Accession codes, unique identifiers, or web links for publicly available datasets
- A description of any restrictions on data availability
- For clinical datasets or third party data, please ensure that the statement adheres to our policy

Data relating to the characterization data of materials and products, general methods, optimization studies, mechanistic studies, mass spectrometry, HPLC and NMR spectra, computational studies and biological activity studies are available in the Supplementary Information. The X-ray crystallographic coordinates for structures of rac-3a, rac-4a, 3a, 4a, ent-4a, 3a-1 reported in this article have been deposited and available free of charge from the Cambridge Crystallographic Data Centre (CCDC), under deposition CCDC numbers 2089968, 2053966, 2015156, 2019560, 2047395 and 2041571, respectively. All other data are available from the corresponding author upon request.

Field-specific reporting

Please select the one below that is the best fit for your research. If you are not sure, read the appropriate sections before making your selection.

☒ Life sciences ☐ Behavioural & social sciences ☐ Ecological, evolutionary & environmental sciences

For a reference copy of the document with all sections, see nature.com/documents/nr-reporting-summary-flat.pdf

Life sciences study design

All studies must disclose on these points even when the disclosure is negative.

Sample size	All groups were designed with 3 biologically independent samples. It's sufficient for the bioactivity screens.
Data exclusions	No data was excluded from the analysis.
Replication	All groups were designed with 3 biologically independent samples. All attempts to replicate the experiments were successful.
Randomization	All cells were randomly and equally seeded into 96-well plates. Data processed by software, without interference from the participants.
Blinding	Our experiments involved only cytology experiments. All samples were measured and intervened by one Investigator.

Reporting for specific materials, systems and methods

We require information from authors about some types of materials, experimental systems and methods used in many studies. Here, indicate whether each material, system or method listed is relevant to your study. If you are not sure if a list item applies to your research, read the appropriate section before selecting a response.

Materials & experimental systems

n/a	Involved in the study	
☒	☐	Antibodies
☐	☒	Eukaryotic cell lines
☒	☐	Palaeontology and archaeology
☒	☐	Animals and other organisms
☒	☐	Human research participants
☒	☐	Clinical data
☒	☐	Dual use research of concern

Methods

n/a	Involved in the study	
☒	☐	ChIP-seq
☒	☐	Flow cytometry
☒	☐	MRI-based neuroimaging

Eukaryotic cell lines

Policy information about cell lines

Cell line source(s)	HCT116, A375, HELA, NCIN87, and HUVEC were obtained from the Cell Bank of the Chinese Academy of Sciences (Shanghai, China), and BB4 cell line (gefitinib-resistant non-small cell lung cancer PC-9 cells) was a gift from professor Qinghua Zhou's laboratory at West China Hospital, Sichuan University
Authentication	None of the cell lines were authenticated.
Mycoplasma contamination	All cell lines were tested negative for mycoplasma contamination.
Commonly misidentified lines (See ICLAC register)	No commonly misidentified cell lines was used in the study.
